# What Made the COVID-19 Pandemic Experience Worse in Communities in Northern Nigeria: Fuzzy Cognitive Mapping of Community Perceptions

**DOI:** 10.1177/2752535X251384522

**Published:** 2025-10-03

**Authors:** Ivan Sarmiento, Yagana Gidado, Hadiza Mudi, Altine Joga, Umaira Ansari, Sa’adatu Bello Kirfi, Mohammed Abbas Ibrahim, Neil Andersson, Anne Cockcroft

**Affiliations:** 1 CIET-PRAM, Department of Family Medicine, 5620 McGill University, Montréal, QC, Canada; 2 Grupo de Estudios en Sistemas Tradicionales de Salud, Escuela de Medicina y Ciencias de la Salud, Universidad del Rosario, Bogotá, Colombia; 3 Bauchi Chapter, Federation of Muslim Women Association in Nigeria (FOMWAN), Bauchi, Nigeria; 4 Centro de Investigación de Enfermedades Tropicales, 341132 Universidad Autónoma de Guerrero, Acapulco, México; 5 Ministry of Cooperatives and Small and Medium Enterprise Development, Bauchi, Nigeria

**Keywords:** participatory models, soft modelling, participatory research, social network analysis, community engagement, people living with disabilities

## Abstract

**Aims:**

Collate local perceptions of factors influencing experience of the COVID-19 pandemic in communities in Bauchi State, Northern Nigeria.

**Results:**

Fuzzy cognitive mapping (FCM) collated participant views of what made their experience worse during the COVID-19 pandemic. FCM uses concepts linked by weighted arrows to indicate perceived causal relationships. Higher weights indicate stronger influences; positive and negative signs indicate direct and inverse causal relationships, respectively. In late 2023, local facilitators collected 81 maps in urban, rural and remote communities, 11 with administrative officials, and four with vulnerable groups (388 participants in total). We created average maps for each stakeholder group. Facilitators inductively grouped factors into categories. We calculated the cumulative net influence (CNI) (range −1 to +1) of categories and identified important causes and outcomes within the network. The maps included 152 factors in 25 categories. *Hunger and lack of food* (CNI = 0.63) worsened pandemic experience the most, followed by *reduced businesses and jobs* (CNI = 0.40), causing economic disruption and threatening livelihoods. *Increased household conflicts* (CNI = 0.35) and *stress and mental health problems* (CNI = 0.30) were also prominent negative influences and intermediate outcomes in the network. *Lockdown* (CNI = 0.34) was the most important underlying cause of other causal categories.

**Conclusions:**

The maps depicted the interconnected impacts of the pandemic on community members. Participants confirmed the worst impacts were related to control measures exacerbating pre-existing economic challenges. These FCM findings will form part of the evidence shared with communities and policy makers to support co-design of strategies for pandemic recovery aligned with community needs and strengths.

## Introduction

The COVID-19 pandemic caused many excess deaths worldwide, directly through COVID-19 deaths and indirectly through increasing other causes of death.^1^ The pandemic had strong negative effects on health care and other health outcomes,^2^ economic and social inequalities,^3–5^ food production and availability,^6^ education,^7^ and the environment.^8^ It undermined the ability of many countries to achieve the Sustainable Developments Goals (SDGs) by 2030, particularly poverty eradication (SDG 1).^9^ The most vulnerable were hit hardest,^10,11^ especially in low- and middle-income countries, with loss of livelihoods, disruption of health and other services, and increasing costs of basic goods.^12^ The pandemic amplified existing inequalities and their impact^13^ including gender-based inequalities.^14^

By 3 October 2023, the Nigerian CDC reported 266,598 cases of COVID-19 and 3,155 deaths or 1.53 per 100,000.^15^ Bauchi State in Northern Nigeria, with a population of almost five million people, reported just 2,020 cases and 24 deaths by October 2022.^16^

COVID-19 recovery policies should address the underlying socioeconomic inequalities exacerbated by the pandemic.^17^ The UN Research Roadmap for COVID-19 recovery recommends leveraging local and traditional knowledge to develop community-tailored solutions, support their implementation, and evaluate outcomes.^18^ This approach seeks transformative change, where socioeconomic recovery can enhance equity, resilience, and sustainability. Promoting change among the most impacted communities should involve them in understanding the issue and designing and implementing solutions.

Our research team developed protocols to systematize and incorporate local and traditional knowledge into evidence-based decision-making in households, communities, and services using fuzzy cognitive mapping (FCM).^19–21^ In this paper, we report the results of using FCM in communities and with other stakeholders at different levels in Bauchi State, Nigeria, to systematize local knowledge about influences on the impact of COVID-19 in Bauchi communities. This is part of a larger initiative to guide community-informed COVID-19 recovery strategies in Bauchi. The FCM described here guided design of household survey instruments to measure the frequency and associations of factors important to the pandemic impact in Bauchi. The FCM and survey informed evidence-based dialogues between stakeholders to inform local and State COVID-19 recovery strategies.

## Methods

### Settings

Bauchi State in northern Nigeria has worse socio-economic indicators than the national average.^22^ The main economic activity is small-scale farming. Women and men in Bauchi have a life expectancy below 56 years,^23^ with basic literacy rates of 26% and 48%, respectively.^22^ Women often marry young and polygamy is common.^22^ In 2018, 18% of women reported experiencing violence during the last year.^22^ Infant mortality in the state (125 children per 1,000 live births) is among the highest in the country.^24^

The Ministry of Cooperatives and Small and Medium Enterprises Development in Bauchi implements the *Nigeria: Community Action (for) Resilience and Economic Stimulus Program* (NG-CARES), with funding from the World Bank,^25^ designed to support state government interventions targeting existing and newly vulnerable and poor households, farmers, and micro and small enterprises (MSEs) affected by the COVID-19 pandemic and economic crisis. NG-CARES aims to provide cash transfers and livelihood support to poor and vulnerable households; increase food security and safe functioning of food supply chains for poor households; and facilitate recovery of MSEs.

Our research engaged communities in Darazo local government area (LGA), which has a population of 450,000 people, mostly from the Hausa and Fulani ethnic groups, and almost all Muslim. The LGA has received internally displaced people fleeing insurgency and communal clashes in nearby states.

### Fuzzy Cognitive Mapping

We use fuzzy cognitive mapping (FCM)^19–21^ to clarify and collate local knowledge about causes of outcomes of concern. FCM offers a common and graphic language to compare and communicate different perspectives about causes, making it a valuable tool for participatory research.^20^ Maps illustrate connections (arrows) between perceived causes and outcomes (nodes) and strengths (weights) of these connections.^21^ Relationships can be positive (an increment of a cause leads to an increment of an outcome) or negative (an increase in a cause leads to a decrease in an outcome). Figure 1 shows an example of a fuzzy cognitive map created by a community group and the same map after digitization.

**Figure 1.**
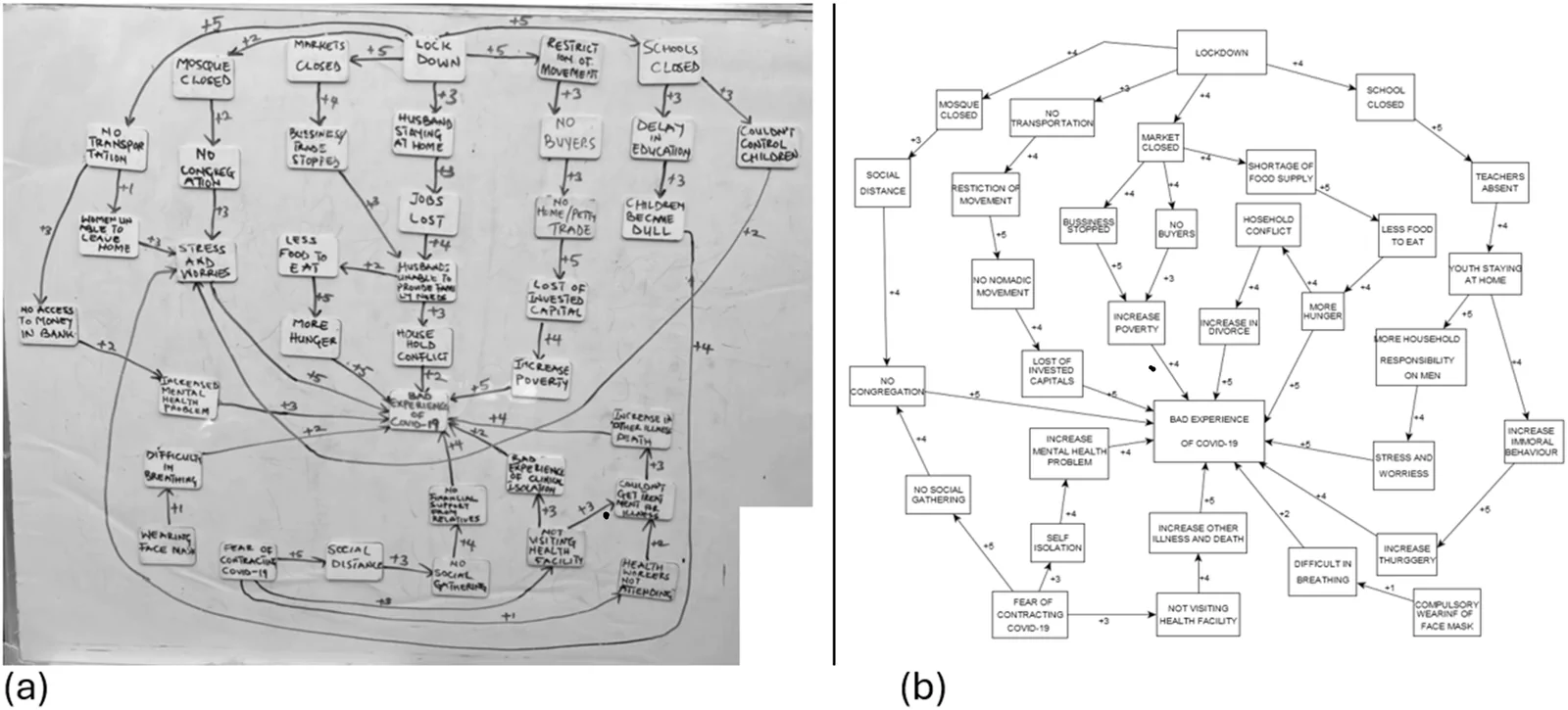
A fuzzy cognitive map created by a community group. Illustrative image of a stakeholder map to show the raw data and the digitized map. It shows all the nodes and arrows generated during the mapping session. This figure is only intended to give an idea of what a fuzzy cognitive map looks like. (a). We used metal whiteboards with concepts (nodes) written on magnetic tiles to facilitate moving concepts as the maps developed. Maps can also be created on paper with sticky labels. The facilitator photographs the completed map as the original data. (b). We created digital maps based on the photographs of the original maps.

### Drawing the Maps

The Bauchi research team ran FCM sessions between September and November 2023 with community groups and administrators at ward, LGA, and state levels. We selected three urban, three rural and three rural-remote communities across three wards in Darazo LGA for the mapping exercise. In each community, a purposive sample of nine groups made maps: young men, young women, adult men, adult women, older men, older women, male and female community leaders, and service providers (men and women together). Each mapping group included four participants with similar characteristics. Previous FCM experience^20^ suggests this group size encourages meaningful discussion while being readily manageable by facilitators. Community leaders agreed for their community to participate and encouraged community members to take part, and mapping participants gave oral informed consent.

Mapping sessions took place in communal spaces such as schools or clinics, supported by trained local Bauchi facilitators and reporters of the same gender as the group participants. The facilitator led the discussion and created the map on a whiteboard as directed by the participants. The reporter noted comments about the meaning of concepts, noting any disagreements and how they were resolved. The facilitator began by asking the group what factors they considered had worsened their experience during the COVID-19 pandemic. They wrote each response on a magnetic tile and put it on the whiteboard, arranging factors related to similar topics near one another. Once no more factors were forthcoming, the facilitator asked participants to identify the connections, direct or indirect, between factors (nodes) and the main outcome and drew these as arrows on the map. Participants then identified the strongest link, assigning it a weight of five, and the weakest link with a weight of one. They weighted other links on the map between these extremes. The facilitator confirmed with participants that the completed map represented their views, and, with their permission, photographed the map. Sessions were in Hausa, but facilitators wrote the labels in English, explaining them in Hausa as the participants linked the factors and weighted the links. To facilitate subsequent analysis of the maps, the field team created a list of “standard” factor labels, added to after each mapping session, which facilitators used in their labelling of factors in subsequent maps.

### Analysis of the Maps

Supplemental material 1 presents a flow chart with the steps in the analysis. We digitized maps using yEd software^26^ and created adjacency matrices, tables where the values in cells indicate the weights of relationships from factors in rows to each other factor in columns. We divided all original weights by five to normalize the values into the range −1 to 1. We standardized the labels of factors with the same meaning but different wording, building on the list of standard factor labels created during the mapping fieldwork.^27^ We calculated fuzzy transitive closure (TC) of the factor maps, identifying the strongest effect a factor could have on other factors through direct or indirect connections.^28^ We then combined the TC maps, calculating the average weight for each relationship across all the maps and separately for community groups of men and women, people with disabilities, rural, urban and remote communities, and community leaders and officials at different levels (Ward, LGA and State). The average weights resulted from adding the weights (positive or negative) of each relationship across all maps and dividing the total by the number of maps.

To facilitate communication of FCM findings, the research team condensed the average TC factor maps into category maps.^29^ They labelled tiles with each of the standard factor names, then placed these tiles in groups of similar concepts on a whiteboard. The process continued until all team members agreed on the contents of each group (category). Supplemental Material 2 lists each category and the corresponding standard factors. To calculate the cumulative net influences of each category -- arrows going from one category to another category -- we summed the weights of the individual factor-level relationships that contributed to the category-level relationship. To standardize this measure, we divided each sum by the highest absolute value obtained from different summations. Values closer to 1 or -1 indicate stronger influences, while those nearer to 0 are weaker.

Based on social network analysis, we calculated centrality measures^27,30^ to indicate the role of different categories in the map. Higher values of outgoing relationships (outdegree centrality) indicated more important perceived causes. Higher values of incoming relationships (indegree centrality) indicated more important perceived outcomes. We also counted the number of times a category was involved in direct and indirect relationships with the main outcome to identify important intermediaries for the effect of other categories. We also counted the number of times a category was the initial node of indirect and direct relationships affecting the main outcome. Categories that were often the initial node in a chain of relationships represent more distal (underlying) causes of the outcome.

To evaluate data saturation, we began with the map containing the most nodes and arranged the maps in sequence for each stakeholder group. We then counted the number of new nodes (causes) reported on each map in the sequence. Few new nodes in the last map indicates that additional maps would be unlikely to contain additional causes.

## Results

Fieldworkers conducted 96 mapping sessions, 81 with community groups involving 324 participants, 15 with special groups (officials, internally displaced and disabled people) at higher administrative levels (three Wards, the LGA and the state), involving 64 participants. Table 1 presents demographic characteristics of the map authors. Figure 1 shows an example of a community map, and Supplemental Material 3 contains all the original maps.

**Table 1.** Number of Maps and Characteristics of Participants in Mapping Groups.

Level	Maps				Participants			Mean age (yr)	Education			
	Total	Urban	Rural	Remote	Total	Male	Female		Secondary and above	Primary	Islamic school	None
STATE	4	4	0	0	20	11	9	53	20	0	0	0
Government officials	1	1	0	0	5	4	1	-				
Traditional leaders	1	1	0	0	5	5	0	-				
FOMWAN	1	1	0	0	5	0	5	-				
Media or civil society organisation	1	1	0	0	5	2	3	-				
LGA	**5**	**5**	**0**	**0**	**20**	**11**	**9**	**45**	**11**	**2**	**7**	**0**
LGA officials	1	1	0	0	4	3	1	-				
Disabled people	2	2	0	0	8	4	4	-				
Internally displaced people	2	2	0	0	8	4	4	-				
WARD	**6**	**6**	**0**	**0**	**24**	**12**	**12**	**46**	**15**	**2**	**7**	**0**
Ward leaders	6	6	0	0	24	12	12	-				
COMMUNITY	**81**	**27**	**27**	**27**	**324**	**170**	**154**	**44**	**97**	**38**	**181**	**8**
Traditional leaders	18	6	6	6	72	36	36	48				
Elderly	18	6	6	6	72	36	36	63				
Adult	18	6	6	6	72	36	36	40				
Youth	18	6	6	6	72	36	36	22				
Service providers	9	3	3	3	36	26	10	38				

1) All the community groups (except the service providers) and the groups of ward leaders, internally displaced people, and disabled people, were single gender groups. Community service providers, LGA officials and groups at state level were mixed gender groups. 2) The figures for mean age and education level are shown for all groups combined at each level.

The overall average map had 33 factors (range 25-50) and 131 relationships (range 75-258) after TC. Across all the maps, participants reported 152 unique factors that we grouped into 25 categories. Supplemental Material 2 shows the standard factors in each category. Supplemental Material 4 lists all the categories and indicates which maps mentioned them as causes of a bad experience of the COVID-19 pandemic. 13 categories were mentioned in all the maps.

Supplemental Material 5 shows the cumulative curve of the number of new nodes for each map in sequence. The first map for each new stakeholder group contained more new nodes. The number of new nodes decreased across maps; there were almost no new nodes in the last maps in sequence.

### Most Influential Categories

Figure 2 shows the average category map that covers all the 96 mapping sessions. For clarity, we have restricted the figure to the five strongest category-level influences on bad experience of the COVID-19 pandemic. Supplemental Material 6 shows the adjacency matrices with all relationships in the category maps. Table 2 shows the cumulative net influence of causal categories on the main outcome among different stakeholder groups. Nearly all stakeholder groups shared similar perceived causes of a bad experience, except for the groups of disabled people. For them, three additional categories emerged as important. Although category maps of all stakeholder groups mentioned reduced health services and increased illnesses and deaths (from causes other than clinical COVID), these categories were not among the five strongest causes in any group. We describe here the five most influential categories.

**Figure 2.**
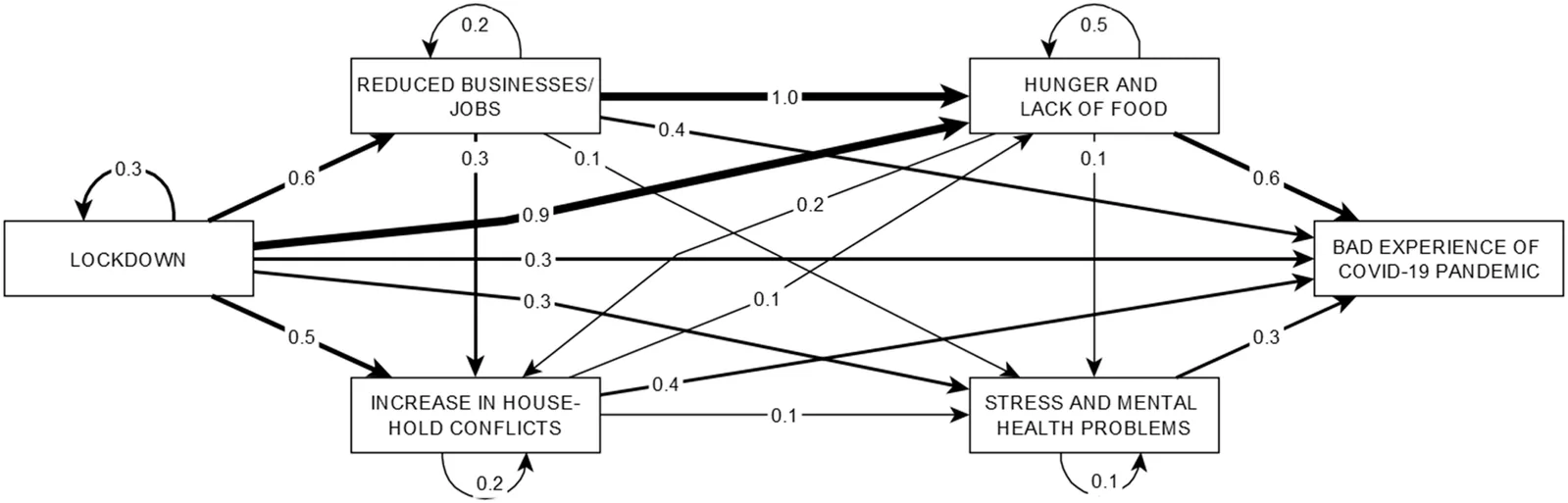
Restricted category map summarising all the maps. Category map, obtained from the combination of all 96 transitive closure maps, showing the five strongest influences on Bad Experience of COVID-19 pandemic. The thickness of lines is proportional to the cumulative influence of categories, where 1 or -1 are the highest influences on the map.

**Table 2.** Comparison of the Strongest Influences Across Category Maps From Different Stakeholders.

Category	All maps 96 maps		Female 41 maps		Male 41 maps		Com. 81 maps		Urban 27 maps		Rural 36 maps		Remote 18 maps		High L* 11 maps		Disab. 2 maps	
	P	CNI	P	CNI	P	CNI	P	CNI	P	CNI	P	CNI	P	CNI	P	CNI	P	CNI
Bad experience of COVID-19 pandemic		-			-			-		-		-		-		-		
Hunger and lack of food	**1**	0.6	**1**	0.5	**1**	0.7	**1**	0.6	**1**	0.5	**1**	0.6	**1**	0.7	**1**	0.6	**1**	0.4
Reduced businesses/jobs	**2**	0.4	**2**	0.4	**2**	0.4	**2**	0.4	**2**	0.4	**2**	0.4	**2**	0.4	**4**	0.3		0.1
Increase in household conflicts	**3**	0.4	**3**	0.3	**5**	0.3	**3**	0.3	**4**	0.3	**3**	0.4		0.3	**2**	0.4		0.2
Lockdown	**4**	0.3	**4**	0.3	**3**	0.4	**4**	0.3	**3**	0.3	**4**	0.3	**4**	0.4	**5**	0.3	**2**	0.2
Reduced education		0.3		0.2		0.3		0.3	**5**	0.3		0.3	**5**	0.3		0.2		0.2
Stress and mental health problems	**5**	0.3	**5**	0.3		0.3	**5**	0.3		0.2		0.3	**3**	0.4	**3**	0.3		0.2
Reduced religious activities		0.3		0.2	**4**	0.3		0.3		0.3	**5**	0.3		0.2		0.3		0.2
Fear and misconception of COVID-19		0.2		0.2		0.2		0.2		0.2		0.2		0.2		0.3	**2**	0.2
Problems with PPE		0.1		0.1		0.1		0.1		0.1		0.1		0.1		0.1	**4**	0.2
Reduced social contact/activities		0.1		0.1		0.2		0.1		0.1		0.1		0.2		0.2	**4**	0.2

P = position in the ranking of the five strongest influences. CNI = Cumulative net influence. Com. = All community members. High L. = staff working at higher administrative levels (State, LGA and Wards). Disab. = people with disabilities.

### Hunger and Lack of Food

All groups highlighted *hunger and lack of food* as the strongest negative influence (Table 2). This category encompasses a range of interacting issues contributing to food insecurity and malnutrition, including a shortage of food supply and an increased demand at home.

“In our neighbourhood, a husband had to sell all his belongings just to feed his family members. He could not get enough food to eat three times a day” (adult women from a rural community)

“As markets closed, people could not transact to earn money to buy food, even food was not available. This increased people’s hunger, this made us suffer” (adult men from an urban community)

Factors such as poverty, limiting access to essential resources, and reduced financial support from relatives exacerbated the struggle to meet basic needs. Men unable to provide for their families faced additional pressure, deepening the cycle of poverty and hunger (see self-pointing arrow in Figure 2). As shown in Figure 2, map creators saw hunger and lack of food as being due to reduced business and loss of jobs (CNI 1.0) and lockdown (CNI 0.9).

### Reduced Businesses or Loss of Jobs

Men and women both identified *reduced businesses or loss of jobs* as the second most important negative influence on their experience. Officers at higher administrative levels ranked it in fourth place. People lost their jobs and livelihoods when businesses and trade activities stopped. Less access to farm implements reduced agricultural productivity and poor harvests. The loss of home trade and street hawking devastated many households that relied on these activities for survival. Closure of markets and shops exacerbated this situation.

“Most people in Bauchi depend on business, so suspension of business affected them very seriously.” (service providers from an urban community)

“Begging is our major source of income. Due to no transport, we could not go out begging, which made us very poor.” (female disabled participant from an urban community)

“A lot of men lost their jobs due to lockdown. Some of us sold animals or foodstuffs at a giveaway price because we had no other income. There was no business, and the little we had, we spent. There was no money in circulation.” (service providers from a rural community)

### Increased household Conflicts

This category ranked as the third or fourth most influential category for almost all the stakeholders. It included factors disrupting harmony and stability of households. The categories of *increased household conflicts* and *stress and mental health problems* had high indegree centrality (they were important outcomes in the network), second only to the main outcome. Domestic violence was part of a toxic environment leading, for example, to more divorce. There was a breakdown of family structures: husbands ran away, wives ran away or were sent back to their parent’s home. Inability to control children and the presence of youth at home due to school closures added to the stress within the family. Reduced affection between couples, frequent, sometimes unwanted, sex, and more unwanted pregnancies further strained relationships. Men staying at home without work exacerbated these issues.

“Most couples couldn’t tolerate each other, they were inpatient with each other, fighting all the time.” (Female ward leaders)

“When a man lost his job, he would get frustrated and angry, which increased conflicts and resulted in divorce in most cases.” (male adults from an urban community)

### Lockdown

Lockdown had the highest outdegree centrality, indicating it was the most important cause in the network. It included restricted movement and reduced economic activity. Closed LGA and state borders halted travel. The suspension of transport services and the closure of filling stations disrupted commuting and reduced access to essential services. Restrictions on movement confined people to their homes, halting their regular activities. The lockdown also prevented men working away from home from returning home, separating families and causing additional stress.

“This lockdown was the main problem. It led to all the other problems. It resulted in no transport, one could not travel to town to visit relatives or for business. It took away all our jobs.” (men from a rural community)

“Sadly, we could not go to farm. We could not cultivate anything; we had shortages of food in our houses.” (male elders from a remote community)

“The lockdown is a very terrible thing. They closed the school, and our children had to stay at home. Our burden increased at home as the children numbers increased.” (disabled men from an urban community)

### Stress and Mental Health Problems

This category ranked fifth in the summary of all maps. It included contributing factors for psychological distress and emotional strain. Stress and worries were exacerbated by feeling socially isolated, which led to a sense of loneliness and disconnection. Isolation, combined with other pressures, led to an increase in mental health problems and even suicide. Parents struggled to meet family responsibilities, and both men and women experienced increased household duties.

“Almost all the male parents developed stress and worries due to additional household responsibilities, especially due to having kids at home all the time.” (male adults from a rural community)

### Intermediaries

Figure 3 shows the analysis of intermediaries based on 5,819 possible direct and indirect paths through which factors could lead to a bad experience. The four categories that were present most often on these paths were *hunger and lack of food* (64% or 3728/5819), *increase of household conflicts* (31% or 1790/5819), *stress and mental health problems* (30% or 1745/5819), and reduced business and jobs (22% or 1262/5819). On average, the paths involved three categories, with a range of two to nine. The most common starting point of paths towards the ultimate outcome was lockdown (26% or 1491/5819), *reduced business and jobs* (12% or 695/5819), hunger and lack of food (11% or 623/5819), *increased household conflicts* (8% or 172/5819) and *fear and misconceptions of COVID-19 (7% or 387/5819)*.

**Figure 3.**
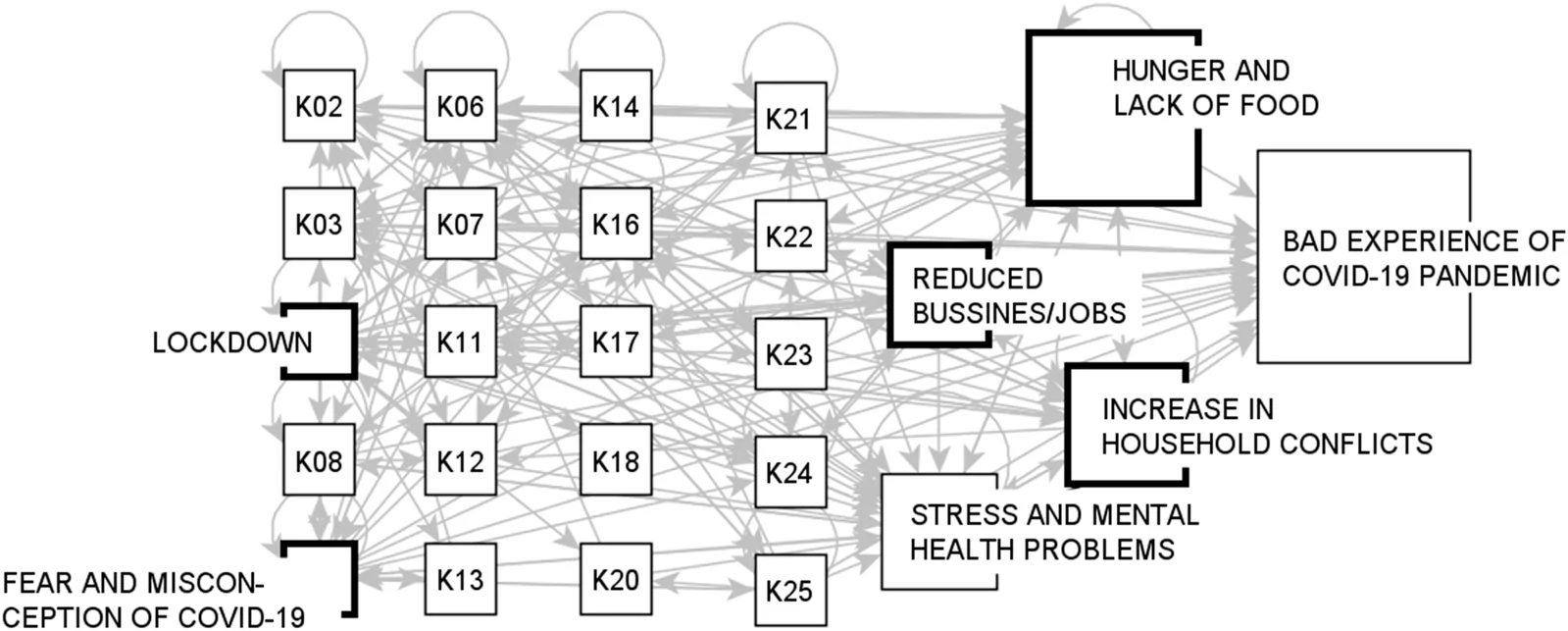
Category map of intermediaries for worse experience of COVID-19. 25 categories of causes ordered by the number of times they acted as intermediaries in the paths towards a bad experience of COVID-19. Bigger boxes indicate categories which were more frequently intermediaries on other causal pathways. The boxes with wider borders show categories more often initiating the path towards a bad experience. The names of the categories shown as codes are available in Supplemental Material 2.

## Discussion

All stakeholder fuzzy cognitive maps identified *hunger and lack of food* as the most influential cause of a bad experience during the COVID-19 pandemic. The second strongest category was *reduced businesses and jobs*, highlighting economic disruptions and their impact on livelihoods. *Increased household conflicts* and *stress and mental health problems* were also important causes. *Lockdown* was the major underlying cause of other causes. *Hunger and lack of food*, *stress and mental health problems*, and *household conflicts* were important intermediaries for the effects of other causes. Perceived causes were broadly similar across most stakeholder groups, but people with disabilities stressed concerns about disruption of their pre-pandemic support systems and difficulties with personal protective equipment.

A strength of this study is inclusion of a wide range of stakeholders. There is no formal procedure to calculate sample size (number of maps) for FCM; our concern was to involve many different groups to reflect plurality of experience. Our analysis of new nodes across maps suggests that we reached data saturation and additional maps would have identified few additional causes.

Darazo LGA has similar culture and economy to other parts of Bauchi State and nearby states; results of this study might be relevant throughout northern Nigeria. Local contextualization is a strength but limits generalizability to other settings. However, the process of valuing community knowledge using FCM might have much wider relevance. FCM is feasible, even in remote areas with limited resources, with non-literate participants, and those with visual or hearing loss. An important caveat is that stakeholder cognitive maps represent *perceived* causal relationships, a pivotal catalyst for community engagement, but do not necessarily confirm causality in a biological sense.

Most countries used lockdowns in some form to control the spread of infection during the COVID-19 pandemic. Evidence from richer countries indicates these measures were broadly effective.^31^ But restrictive lockdown measures had serious adverse effects, particularly economic effects, in low- and middle-income countries.^32^ From early in the pandemic, authors raised concerns about lockdowns worsening the already tenuous situation for poor populations in sub-Saharan Africa.^33–36^ The details of lockdown measures varied between countries in the region but there was little evidence of their effectiveness in preventing infections.^37^ Lockdowns reportedly led to conflicts with authorities in South Africa and Nigeria.^38,39^ The maps in our study clarify adverse effects of the COVID-19 lockdown in Bauchi, as experienced by community members. For participants in our study, the most important causes of their bad experience during the pandemic were related to economic stresses and food insecurity, with lockdown as the underlying cause of both.

Our study on community experiences contributes to broadening the knowledge base on pandemic impacts beyond health issues, as recommended in the UN research roadmap for COVID-19 recovery.^18^ Fuzzy cognitive maps provide a comprehensive picture of what communities consider important, expressed in their own terms. Additional quantitative studies could measure the impact of these factors. Intervention studies could apply strategies for informed recovery.

The FCM and its results described here are part of a larger participatory research program in Bauchi to inform evidence-based policies for COVID-19 recovery and support communities taking their own actions towards recovery. We used the FCM findings to design the instrument for a household survey that asked about issues important to the pandemic experience in the specific local context. The FCM and survey results are the evidential substrate for community dialogue groups. These groups are identifying their own priorities for action and interacting with representatives of the government bodies tasked with supporting pandemic recovery. We will report on the survey, the process of the dialogues between communities and government bodies, and the outcomes in a subsequent manuscript.

## Conclusions

Community members in Bauchi experienced the impacts of the COVID-19 pandemic as multifaceted and interconnected in domains other than health. They saw lockdown as the underlying cause of problems, and food insecurity was the key immediate concern. These community perceptions will inform COVID-19 recovery strategies that are pro-equity and respond to the needs and strengths of communities.

## Supplemental Material

Supplemental Material - What Made the COVID-19 Pandemic Experience Worse in Communities in Northern Nigeria: Fuzzy Cognitive Mapping of Community Perceptions Supplemental Material for What Made the COVID-19 Pandemic Experience Worse in Communities in Northern Nigeria: Fuzzy Cognitive Mapping of Community Perceptions by Ivan Sarmiento, Yagana Gidado, Hadiza Mudi, Altine Joga, Umaira Ansari, Sa’adatu Bello Kirfi, Mohammed Abbas Ibrahim, Neil Andersson, Anne Cockcroft in Community Health Equity Research & Policy.

## Data Availability

All the data used in this study are included in this publication*.
