# Supplementary material for: What Made the COVID-19 Pandemic Experience Worse in Communities in Northern Nigeria: Fuzzy Cognitive Mapping of Community Perceptions
Source: Community Health Equity Res Policy. 2025 Oct 3;47(2):125–34. doi: 10.1177/2752535X251384522 (PMC13752235; doi:10.1177/2752535X251384522)
Supplement: Supplementary file 1 [file sj-pdf-1-qch-10.1177_2752535X251384522.pdf]

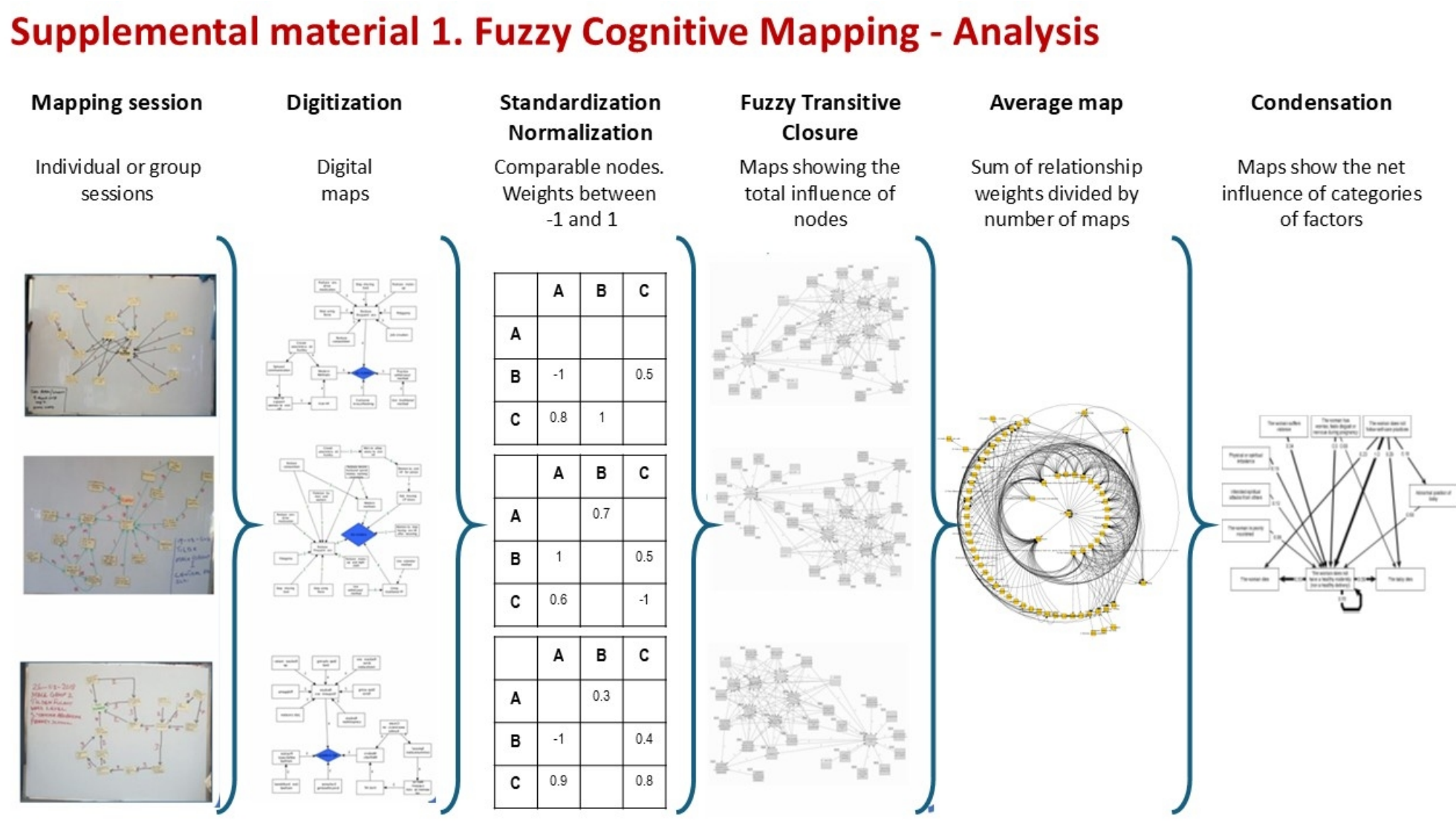

Supplementary material 1

338x190mm (96 x 96 DPI)

Supplementary material 2. List of unique factors across the maps and their corresponding categories.

Legend. The table presents the standard name of each factor and the category to which it belongs. Factors and categories have unique associated codes (F1 to F163 for factors and K01 to K25 for categories) to facilitate notation on tabular formats of the maps.

Standard factor	Code Factor	Category	Code Category
Bad experience of COVID-19 pandemic	F01	BAD EXPERIENCE OF COVID-19 PANDEMIC	K01
Bad use of social media	F04	INCREASE IN SOCIAL VICES	K02
Difficulty in resolving youth conflicts	F22		
Increase in cybercrime	F46		
Increase in fornication	F49		
Increased stealing	F53		
Increase in transactional sex	F54		
Increased child labour	F55		
Increased corruption	F56		
Increased crime	F57		
Increased drug-abuse	F58		
Increased gambling	F59		
Increased immorality	F60		
Increased kidnapping	F61		
Increased rape	F65		
Increased stealing	F67		
Increased thuggery	F69		
Banks closed or limited access	F05	LACK OF ACCESS TO MONEY	K03
No access to pension services	F102		
Loss of Hajj deposit	F81		
Lost invested capital	F82		
Borders closed	F06	LOCKDOWN	K04
No tourism	F119		
No transport	F120		
Restriction of movement	F140		
Filling station closed	F33		
LGA secretariat closed	F79		
Lockdown	F80		
Men couldn’t come back home	F86		
Businesses/trade stopped	F07	REDUCED BUSINESSES/JOBS	K05
No access to farm implements	F101		
No buyers	F104		

For Peer Review

No home/petty trade	F112		
No production of goods	F116		
Not going to farm	F123		
Poor harvest	F132		
Street hawking stopped	F146		
Decreased government revenue	F15		
Jobs lost	F70		
Markets/shops closed	F85		
Cannot communicate with husbands in city	F08	REDUCED SOCIAL CONTACT/ACTIVITIES	K06
No condolence visit	F105		
No entry at public places	F108		
Compulsory staying at home	F11		
No funerals	F110		
No social gatherings	F118		
No weddings	F121		
Temperature screened at public places	F150		
Women unable to leave home	F157		
Increased stigma/disparity for disabled people	F68		
Less affection between people	F76		
Less caregiver support	F77		
Cannot access maternal health services	F09	REDUCED HEALTH SERVICES	K07
Not visiting health facility	F124		
Could not get treatment for diseases	F14		
TBA is not attending delivery	F148		
Unable to buy medicines	F152		
Development partners not monitoring health facilities	F18		
Difficulty accessing care by disabled	F19		
Health facility closed	F37		
Health workers not available	F38		
Immunization stopped	F42		
Increase in self-medication	F50		
Lack of delivery kits for TBAs	F73		
Compulsory face mask/sanitizer	F10	PROBLEMS WITH PPE	K08
Not wearing face masks	F125		
Odour of sanitizer	F126		
Wearing face masks	F154		
Difficult breathing	F20		
Difficulty in lip reading	F21		
Difficulty with practising protective measures by disabled	F23		

For Peer Review

Lack of face masks/sanitizer at home	F74		
Constant media report of COVID-19	F12	FEAR AND MISCONCEPTION OF COVID-19	K09
People feeling insecure	F128		
Rumours on covid-19	F141		
Stigma against COVID patients	F145		
Fear of contracting COVID-19	F29		
Fear of death	F30		
Fear of each other	F31		
Lack of awareness on COVID-19	F71		
Misconception on covid vaccine	F89		
Physical abuse by wives	F129	INCREASE IN HOUSEHOLD CONFLICTS	K10
Could not control children	F13		
Reduced sex between couples	F137		
Wives ran away	F155		
Wives sent home	F156		
Youth/children staying at home	F158		
Domestic violence	F24		
Frequent sex	F36		
Household conflicts	F40		
Husbands ran away	F41		
Increase in divorce	F47		
Men staying at home	F87		
More unwanted pregnancies	F99		
No religious education	F117	REDUCED EDUCATION	K11
Poor performance by students	F133		
School/madrasah closed	F142		
Teachers absent	F149		
Two shifts in schools	F151		
Delay in education	F16		
Increase in school dropout	F51		
More burden on madrasah	F90		
Penalty for not wearing faces masks	F127	PENALTIES/PUNISHMENT	K12
Physical punishment	F131		
Delay in justice	F17		
Fear of being arrested	F27		
Fine payment	F34		
More imprisonment	F97		
Non-compliance with government policies	F122	CONFLICT BETWEEN GOVERNMENT AND CITIZENS	K13
Enforced COVID vaccination	F25		
No nomadic movement with animals	F113	ANIMAL/HERDERS PROBLEMS INCREASED	K14
Veterinary doctors not available	F153		

For Peer Review

Farmers/herders clash	F26		
Increase in animal death	F45		
Malnutrition in animals	F84		
Stress and worries	F147	STRESS AND MENTAL HEALTH PROBLEMS	K15
Feeling socially isolated	F32		
Increase in suicide	F52		
Increased mental health problems	F62		
More burden on parents	F91		
More household responsibility on men	F94		
More household responsibility on women	F95		
Physical injuries	F130	INCREASED ILLNESS/DEATH	K16
HIV/STI increased	F39		
Increased other illnesses/deaths	F63		
Increased reckless playing among youth	F66		
More maternal deaths/complications	F98		
No access to water supply	F103	REDUCED GOVERNMENT SUPPORT	K17
No government support	F111		
Improper implementation of projects	F43		
Increase in early marriage	F48	INCREASE IN EARLY MARRIAGE	K18
No financial support from relatives	F109	HUNGER AND LACK OF FOOD	K19
Poverty	F134		
Shortage of food supply	F143		
Increased poverty	F64		
Lack of basic needs	F72		
Lack of hygiene	F75		
Less food to eat	F78		
Malnutrition	F83		
Men unable to provide family needs	F88		
More demand for food at home	F93		
More hunger	F96		
No covid-19 medicines at health facilities	F107	LACK OF COVID-19 COMMODITIES	K20
More COVID-19 cases	F92		
Mosques/churches closed	F100	REDUCED RELIGIOUS ACTIVITIES	K21
No congregation	F106		
No pilgrimage	F114		
No preaching	F115		
Price hike	F135	PRICE HIKE	K22
Reduced physical fitness	F136	REDUCED PHYSICAL FITNESS	K23
Restricted physical activity	F139		
Restricted passengers in vehicles	F138	ENFORCED SOCIAL DISTANCE	K24

For Peer Review

Social distancing	F144		
Bad experience of clinical isolation	F02	FAULTY CLINICAL ISOLATION POLICIES	K25
Afraid to visit a health facility	F03		
Fear of clinical isolation	F28		
Forced COVID testing	F35		
Inadequate isolation centre	F44		

For Peer Review

Map code	From	To	From	To	W
Map1	F40	F47	Household	Increase in	0.2
Map1	F40	F01	Household	Bad experi	0.2
Map1	F47	F01	Increase in	Bad experi	0.6
Map1	F85	F82	Markets/sh	Lost invest	0.6
Map1	F85	F07	Markets/sh	Businesses	0.6
Map1	F85	F01	Markets/sh	Bad experi	0.6
Map1	F85	F64	Markets/sh	Increased	0.6
Map1	F85	F96	Markets/sh	More hung	0.6
Map1	F85	F78	Markets/sh	Less food t	0.6
Map1	F87	F40	Men stayin	Household	0.4
Map1	F87	F47	Men stayin	Increase in	0.2
Map1	F87	F01	Men stayin	Bad experi	0.2
Map1	F80	F40	Lockdown	Household	0.4
Map1	F80	F47	Lockdown	Increase in	0.2
Map1	F80	F85	Lockdown	Markets/sh	0.8
Map1	F80	F87	Lockdown	Men stayin	0.4
Map1	F80	F82	Lockdown	Lost invest	0.6
Map1	F80	F07	Lockdown	Businesses	0.6
Map1	F80	F01	Lockdown	Bad experi	0.8
Map1	F80	F64	Lockdown	Increased	0.6
Map1	F80	F62	Lockdown	Increased	0.8
Map1	F80	F16	Lockdown	Delay in ec	0.6
Map1	F80	F96	Lockdown	More hung	0.6
Map1	F80	F112	Lockdown	No home/p	0.6
Map1	F80	F78	Lockdown	Less food t	0.6
Map1	F80	F13	Lockdown	Could not	0.4
Map1	F80	F70	Lockdown	Jobs lost	0.8
Map1	F80	F143	Lockdown	Shortage o	0.6
Map1	F80	F142	Lockdown	School/ma	0.8
Map1	F80	F140	Lockdown	Restriction	0.6
Map1	F80	F147	Lockdown	Stress and	0.4
Map1	F80	F120	Lockdown	No transpo	0.4
Map1	F80	F123	Lockdown	Not going t	0.6
Map1	F80	F106	Lockdown	No congreg	0.4
Map1	F80	F104	Lockdown	No buyers	0.6
Map1	F80	F100	Lockdown	Mosques/c	0.8
Map1	F82	F01	Lost invest	Bad experi	0.6
Map1	F82	F64	Lost invest	Increased	0.6
Map1	F82	F96	Lost invest	More hung	0.6
Map1	F82	F78	Lost invest	Less food t	0.6
Map1	F07	F82	Businesses	Lost invest	1
Map1	F07	F01	Businesses	Bad experi	0.6
Map1	F07	F64	Businesses	Increased	0.6
Map1	F07	F96	Businesses	More hung	0.6
Map1	F07	F78	Businesses	Less food t	0.6
Map1	F29	F01	Fear of cor	Bad experi	0.6
Map1	F29	F63	Fear of cor	Increased	0.6
Map1	F29	F124	Fear of cor	Not visiting	0.8
Map1	F29	F31	Fear of cor	Fear of eac	0.4
Map1	F29	F14	Fear of cor	Could not	0.6
Map1	F64	F01	Increased	Bad experi	1
Map1	F64	F96	Increased	More hung	0.6
Map1	F64	F78	Increased	Less food t	0.8
Map1	F63	F01	Increased	Bad experi	0.8
Map1	F62	F01	Increased	Bad experi	1

For Peer Review

Map1	F16	F01	Delay in ed	Bad experi	0.8
Map1	F118	F01	No social g	Bad experi	0.4
Map1	F118	F62	No social g	Increased r	0.4
Map1	F118	F109	No social g	No financia	0.8
Map1	F96	F01	More hung	Bad experi	0.8
Map1	F112	F01	No home/p	Bad experi	0.6
Map1	F112	F64	No home/p	Increased p	0.6
Map1	F112	F96	No home/p	More hung	0.6
Map1	F112	F78	No home/p	Less food t	0.6
Map1	F124	F01	Not visiting	Bad experi	0.6
Map1	F124	F63	Not visiting	Increased c	0.6
Map1	F124	F14	Not visiting	Could not g	0.6
Map1	F31	F01	Fear of eac	Bad experi	0.6
Map1	F78	F01	Less food t	Bad experi	1
Map1	F78	F96	Less food t	More hung	0.6
Map1	F13	F01	Could not c	Bad experi	0.6
Map1	F13	F147	Could not c	Stress and	0.6
Map1	F70	F40	Jobs lost	Household	0.4
Map1	F70	F47	Jobs lost	Increase in	0.2
Map1	F70	F87	Jobs lost	Men stayin	0.4
Map1	F70	F01	Jobs lost	Bad experi	0.8
Map1	F70	F62	Jobs lost	Increased r	0.8
Map1	F14	F01	Could not g	Bad experi	0.8
Map1	F14	F63	Could not g	Increased c	1
Map1	F143	F01	Shortage o	Bad experi	1
Map1	F143	F96	Shortage o	More hung	0.6
Map1	F143	F78	Shortage o	Less food t	1
Map1	F142	F01	School/ma	Bad experi	0.6
Map1	F142	F16	School/ma	Delay in ec	0.6
Map1	F142	F13	School/ma	Could not c	0.4
Map1	F142	F147	School/ma	Stress and	0.4
Map1	F140	F40	Restriction	Household	0.4
Map1	F140	F47	Restriction	Increase in	0.2
Map1	F140	F87	Restriction	Men stayin	0.4
Map1	F140	F01	Restriction	Bad experi	0.6
Map1	F140	F64	Restriction	Increased p	0.6
Map1	F140	F62	Restriction	Increased r	0.6
Map1	F140	F96	Restriction	More hung	0.6
Map1	F140	F112	Restriction	No home/p	0.6
Map1	F140	F78	Restriction	Less food t	0.6
Map1	F140	F70	Restriction	Jobs lost	0.6
Map1	F140	F143	Restriction	Shortage o	0.6
Map1	F140	F120	Restriction	No transpo	0.4
Map1	F140	F123	Restriction	Not going t	0.8
Map1	F140	F104	Restriction	No buyers	0.6
Map1	F147	F01	Stress and	Bad experi	1
Map1	F120	F01	No transpo	Bad experi	0.6
Map1	F120	F96	No transpo	More hung	0.6
Map1	F120	F78	No transpo	Less food t	0.6
Map1	F120	F143	No transpo	Shortage o	0.6
Map1	F123	F01	Not going t	Bad experi	0.6
Map1	F123	F96	Not going t	More hung	0.6
Map1	F123	F78	Not going t	Less food t	0.6
Map1	F123	F143	Not going t	Shortage o	0.6
Map1	F144	F01	Social dista	Bad experi	0.4
Map1	F144	F62	Social dista	Increased r	0.4

For Peer Review

Map1	F144	F118	Social dista	No social g	0.6
Map1	F144	F109	Social dista	No financia	0.6
Map1	F109	F01	No financia	Bad experi	0.4
Map1	F109	F62	No financia	Increased r	0.4
Map1	F106	F01	No congreg	Bad experi	0.8
Map1	F106	F147	No congreg	Stress and	0.8
Map1	F104	F01	No buyers	Bad experi	0.6
Map1	F104	F64	No buyers	Increased p	0.6
Map1	F104	F96	No buyers	More hung	0.6
Map1	F104	F112	No buyers	No home/p	0.8
Map1	F104	F78	No buyers	Less food t	0.6
Map1	F100	F01	Mosques/c	Bad experi	0.4
Map1	F100	F147	Mosques/c	Stress and	0.4
Map1	F100	F106	Mosques/c	No congreg	0.4
Map2	F40	F47	Household	Increase in	0.2
Map2	F40	F01	Household	Bad experi	0.2
Map2	F40	F147	Household	Stress and	0.2
Map2	F47	F01	Increase in	Bad experi	0.6
Map2	F47	F147	Increase in	Stress and	0.6
Map2	F85	F40	Markets/sh	Household	0.4
Map2	F85	F47	Markets/sh	Increase in	0.2
Map2	F85	F87	Markets/sh	Men stayin	0.8
Map2	F85	F82	Markets/sh	Lost invest	0.8
Map2	F85	F07	Markets/sh	Businesses	0.8
Map2	F85	F01	Markets/sh	Bad experi	0.4
Map2	F85	F64	Markets/sh	Increased p	0.8
Map2	F85	F63	Markets/sh	Increased c	0.6
Map2	F85	F96	Markets/sh	More hung	0.8
Map2	F85	F70	Markets/sh	Jobs lost	0.8
Map2	F85	F147	Markets/sh	Stress and	0.2
Map2	F87	F40	Men stayin	Household	0.4
Map2	F87	F47	Men stayin	Increase in	0.2
Map2	F87	F01	Men stayin	Bad experi	0.4
Map2	F87	F64	Men stayin	Increased p	1
Map2	F87	F63	Men stayin	Increased c	0.6
Map2	F87	F96	Men stayin	More hung	0.8
Map2	F87	F147	Men stayin	Stress and	0.2
Map2	F80	F40	Lockdown	Household	0.4
Map2	F80	F47	Lockdown	Increase in	0.2
Map2	F80	F85	Lockdown	Markets/sh	1
Map2	F80	F87	Lockdown	Men stayin	0.8
Map2	F80	F82	Lockdown	Lost invest	0.8
Map2	F80	F38	Lockdown	Health wor	0.6
Map2	F80	F07	Lockdown	Businesses	0.8
Map2	F80	F01	Lockdown	Bad experi	0.8
Map2	F80	F64	Lockdown	Increased p	0.8
Map2	F80	F63	Lockdown	Increased c	0.6
Map2	F80	F121	Lockdown	No wedding	0.4
Map2	F80	F96	Lockdown	More hung	0.8
Map2	F80	F133	Lockdown	Poor perfor	0.6
Map2	F80	F142	Lockdown	School/mad	1
Map2	F80	F70	Lockdown	Jobs lost	0.8
Map2	F80	F13	Lockdown	Could not c	0.8
Map2	F80	F16	Lockdown	Delay in ec	1
Map2	F80	F124	Lockdown	Not visiting	0.6
Map2	F80	F140	Lockdown	Restriction	0.8

For Peer Review

Map2	F80	F147	Lockdown	Stress and	0.8
Map2	F80	F120	Lockdown	No transpo	0.6
Map2	F80	F106	Lockdown	No congreg	0.8
Map2	F80	F100	Lockdown	Mosques/c	0.8
Map2	F82	F01	Lost invest	Bad experi	0.4
Map2	F82	F64	Lost invest	Increased p	0.6
Map2	F82	F63	Lost invest	Increased c	0.6
Map2	F82	F96	Lost invest	More hung	0.6
Map2	F38	F01	Health wor	Bad experi	0.4
Map2	F38	F63	Health wor	Increased c	0.6
Map2	F38	F124	Health wor	Not visiting	0.6
Map2	F07	F40	Businesses	Household	0.4
Map2	F07	F47	Businesses	Increase in	0.2
Map2	F07	F87	Businesses	Men stayin	1
Map2	F07	F82	Businesses	Lost invest	0.8
Map2	F07	F01	Businesses	Bad experi	0.4
Map2	F07	F64	Businesses	Increased p	1
Map2	F07	F63	Businesses	Increased c	0.6
Map2	F07	F96	Businesses	More hung	0.8
Map2	F07	F147	Businesses	Stress and	0.2
Map2	F20	F01	Difficult bre	Bad experi	0.4
Map2	F29	F01	Fear of cor	Bad experi	0.6
Map2	F29	F31	Fear of cor	Fear of eac	0.8
Map2	F29	F147	Fear of cor	Stress and	0.6
Map2	F64	F01	Increased p	Bad experi	0.4
Map2	F64	F63	Increased p	Increased c	0.6
Map2	F64	F96	Increased p	More hung	0.8
Map2	F63	F01	Increased c	Bad experi	0.4
Map2	F121	F01	No wedding	Bad experi	0.6
Map2	F96	F01	More hung	Bad experi	0.4
Map2	F96	F63	More hung	Increased c	0.6
Map2	F133	F01	Poor perfor	Bad experi	0.6
Map2	F133	F147	Poor perfor	Stress and	0.6
Map2	F142	F01	School/ma	Bad experi	0.6
Map2	F142	F133	School/ma	Poor perfor	0.6
Map2	F142	F13	School/ma	Could not c	0.8
Map2	F142	F16	School/ma	Delay in ec	1
Map2	F142	F147	School/ma	Stress and	0.6
Map2	F70	F40	Jobs lost	Household	0.4
Map2	F70	F47	Jobs lost	Increase in	0.2
Map2	F70	F87	Jobs lost	Men stayin	0.8
Map2	F70	F82	Jobs lost	Lost invest	0.8
Map2	F70	F07	Jobs lost	Businesses	0.8
Map2	F70	F01	Jobs lost	Bad experi	0.4
Map2	F70	F64	Jobs lost	Increased p	0.8
Map2	F70	F63	Jobs lost	Increased c	0.6
Map2	F70	F96	Jobs lost	More hung	0.8
Map2	F70	F147	Jobs lost	Stress and	0.2
Map2	F31	F01	Fear of eac	Bad experi	0.6
Map2	F31	F147	Fear of eac	Stress and	0.6
Map2	F74	F01	Lack of fac	Bad experi	0.68
Map2	F13	F01	Could not c	Bad experi	0.6
Map2	F13	F147	Could not c	Stress and	0.6
Map2	F10	F01	Compulsor	Bad experi	0.6
Map2	F10	F74	Compulsor	Lack of fac	0.6
Map2	F16	F01	Delay in ec	Bad experi	0.6

For Peer Review

Map2	F16	F133	Delay in ed	Poor perfor	0.6
Map2	F16	F147	Delay in ed	Stress and	0.6
Map2	F124	F01	Not visiting	Bad experi	0.4
Map2	F124	F63	Not visiting	Increased (	0.6
Map2	F140	F01	Restriction	Bad experi	0.4
Map2	F140	F121	Restriction	No wedding	0.4
Map2	F147	F01	Stress and	Bad experi	1
Map2	F120	F40	No transpo	Household	0.4
Map2	F120	F47	No transpo	Increase in	0.2
Map2	F120	F87	No transpo	Men stayin	0.6
Map2	F120	F82	No transpo	Lost invest	0.6
Map2	F120	F07	No transpo	Businesses	0.6
Map2	F120	F01	No transpo	Bad experi	0.4
Map2	F120	F64	No transpo	Increased p	0.6
Map2	F120	F63	No transpo	Increased (	0.6
Map2	F120	F96	No transpo	More hung	0.6
Map2	F120	F70	No transpo	Jobs lost	0.6
Map2	F120	F147	No transpo	Stress and	0.2
Map2	F106	F01	No congreg	Bad experi	0.8
Map2	F106	F147	No congreg	Stress and	0.8
Map2	F100	F01	Mosques/c	Bad experi	0.8
Map2	F100	F147	Mosques/c	Stress and	0.8
Map2	F100	F106	Mosques/c	No congreg	1
Map3	F40	F01	Household	Bad experi	0.6
Map3	F40	F131	Household	Physical pu	0.6
Map3	F85	F82	Markets/sh	Lost invest	0.6
Map3	F85	F07	Markets/sh	Businesses	0.6
Map3	F85	F01	Markets/sh	Bad experi	0.6
Map3	F85	F64	Markets/sh	Increased p	0.6
Map3	F85	F70	Markets/sh	Jobs lost	0.6
Map3	F85	F96	Markets/sh	More hung	0.6
Map3	F85	F78	Markets/sh	Less food t	0.6
Map3	F85	F143	Markets/sh	Shortage o	0.8
Map3	F80	F40	Lockdown	Household	0.4
Map3	F80	F85	Lockdown	Markets/sh	0.8
Map3	F80	F82	Lockdown	Lost invest	0.6
Map3	F80	F07	Lockdown	Businesses	0.6
Map3	F80	F01	Lockdown	Bad experi	0.6
Map3	F80	F64	Lockdown	Increased p	0.6
Map3	F80	F63	Lockdown	Increased (	0.6
Map3	F80	F158	Lockdown	Youth/child	0.8
Map3	F80	F70	Lockdown	Jobs lost	0.6
Map3	F80	F96	Lockdown	More hung	0.6
Map3	F80	F133	Lockdown	Poor perfor	0.6
Map3	F80	F131	Lockdown	Physical pu	0.4
Map3	F80	F118	Lockdown	No social g	0.2
Map3	F80	F78	Lockdown	Less food t	0.6
Map3	F80	F38	Lockdown	Health wor	0.6
Map3	F80	F16	Lockdown	Delay in ec	0.6
Map3	F80	F143	Lockdown	Shortage o	0.8
Map3	F80	F142	Lockdown	School/ma	0.8
Map3	F80	F140	Lockdown	Restriction	0.8
Map3	F80	F147	Lockdown	Stress and	0.6
Map3	F80	F120	Lockdown	No transpo	0.6
Map3	F80	F144	Lockdown	Social dista	0.6
Map3	F80	F106	Lockdown	No congreg	0.6

For Peer Review

Map3	F80	F100	Lockdown	Mosques/c	0.6
Map3	F82	F01	Lost invest	Bad experi	0.8
Map3	F82	F64	Lost invest	Increased	0.8
Map3	F07	F82	Businesses	Lost invest	0.8
Map3	F07	F01	Businesses	Bad experi	0.8
Map3	F07	F64	Businesses	Increased	0.8
Map3	F07	F70	Businesses	Jobs lost	0.8
Map3	F20	F01	Difficult bre	Bad experi	0.4
Map3	F29	F20	Fear of cor	Difficult bre	0.4
Map3	F29	F01	Fear of cor	Bad experi	0.4
Map3	F29	F154	Fear of cor	Wearing fa	0.6
Map3	F64	F01	Increased	Bad experi	0.8
Map3	F63	F01	Increased	Bad experi	0.8
Map3	F154	F20	Wearing fa	Difficult bre	0.4
Map3	F154	F01	Wearing fa	Bad experi	0.4
Map3	F158	F40	Youth/child	Household	0.4
Map3	F158	F01	Youth/child	Bad experi	0.4
Map3	F158	F131	Youth/child	Physical pu	0.4
Map3	F70	F82	Jobs lost	Lost invest	0.8
Map3	F70	F01	Jobs lost	Bad experi	0.8
Map3	F70	F64	Jobs lost	Increased	0.8
Map3	F96	F01	More hung	Bad experi	1
Map3	F133	F01	Poor perfor	Bad experi	0.6
Map3	F133	F133	Poor perfor	Poor perfor	0.4
Map3	F133	F16	Poor perfor	Delay in ec	0.6
Map3	F131	F01	Physical pu	Bad experi	0.8
Map3	F118	F01	No social g	Bad experi	0.4
Map3	F118	F147	No social g	Stress and	0.4
Map3	F78	F01	Less food t	Bad experi	0.8
Map3	F78	F96	Less food t	More hung	0.8
Map3	F38	F01	Health wor	Bad experi	0.8
Map3	F38	F63	Health wor	Increased	0.8
Map3	F16	F01	Delay in ec	Bad experi	0.6
Map3	F143	F01	Shortage o	Bad experi	0.6
Map3	F143	F96	Shortage o	More hung	0.6
Map3	F143	F78	Shortage o	Less food t	0.6
Map3	F142	F40	School/ma	Household	0.4
Map3	F142	F01	School/ma	Bad experi	0.6
Map3	F142	F158	School/ma	Youth/child	0.8
Map3	F142	F133	School/ma	Poor perfor	0.6
Map3	F142	F131	School/ma	Physical pu	0.4
Map3	F142	F16	School/ma	Delay in ec	0.6
Map3	F140	F01	Restriction	Bad experi	0.6
Map3	F140	F63	Restriction	Increased	0.6
Map3	F140	F38	Restriction	Health wor	0.6
Map3	F140	F120	Restriction	No transpo	0.6
Map3	F147	F01	Stress and	Bad experi	0.8
Map3	F120	F01	No transpo	Bad experi	0.6
Map3	F120	F63	No transpo	Increased	0.6
Map3	F120	F38	No transpo	Health wor	0.6
Map3	F144	F01	Social dista	Bad experi	0.6
Map3	F144	F118	Social dista	No social g	0.2
Map3	F144	F147	Social dista	Stress and	0.6
Map3	F144	F106	Social dista	No congreg	0.8
Map3	F144	F100	Social dista	Mosques/c	1
Map3	F106	F01	No congreg	Bad experi	0.6

For Peer Review

Map3	F106	F147	No congreg	Stress and	0.6
Map3	F100	F01	Mosques/c	Bad experi	0.6
Map3	F100	F147	Mosques/c	Stress and	0.6
Map3	F100	F106	Mosques/c	No congreg	0.8
Map4	F41	F01	Husbands	Bad experi	0.4
Map4	F40	F47	Household	Increase in	0.4
Map4	F40	F01	Household	Bad experi	0.4
Map4	F40	F62	Household	Increased r	0.4
Map4	F88	F41	Men unable	Husbands	0.4
Map4	F88	F40	Men unable	Household	0.6
Map4	F88	F47	Men unable	Increase in	0.4
Map4	F88	F01	Men unable	Bad experi	0.4
Map4	F88	F62	Men unable	Increased r	0.4
Map4	F47	F01	Increase in	Bad experi	0.6
Map4	F85	F47	Markets/sh	Increase in	0.6
Map4	F85	F83	Markets/sh	Malnutrition	0.4
Map4	F85	F01	Markets/sh	Bad experi	0.6
Map4	F85	F63	Markets/sh	Increased c	0.6
Map4	F85	F96	Markets/sh	More hung	0.6
Map4	F85	F78	Markets/sh	Less food t	0.6
Map4	F85	F143	Markets/sh	Shortage o	0.6
Map4	F80	F41	Lockdown	Husbands	0.4
Map4	F80	F40	Lockdown	Household	0.6
Map4	F80	F88	Lockdown	Men unable	0.6
Map4	F80	F47	Lockdown	Increase in	0.6
Map4	F80	F85	Lockdown	Markets/sh	0.8
Map4	F80	F83	Lockdown	Malnutrition	0.4
Map4	F80	F01	Lockdown	Bad experi	0.6
Map4	F80	F69	Lockdown	Increased t	0.4
Map4	F80	F67	Lockdown	Increased s	0.4
Map4	F80	F64	Lockdown	Increased p	0.6
Map4	F80	F63	Lockdown	Increased c	0.6
Map4	F80	F62	Lockdown	Increased r	0.4
Map4	F80	F60	Lockdown	Increased i	0.4
Map4	F80	F158	Lockdown	Youth/child	0.8
Map4	F80	F16	Lockdown	Delay in ed	0.6
Map4	F80	F118	Lockdown	No social g	0.6
Map4	F80	F96	Lockdown	More hung	0.6
Map4	F80	F110	Lockdown	No funerals	0.4
Map4	F80	F54	Lockdown	Increase in	0.4
Map4	F80	F51	Lockdown	Increase in	0.4
Map4	F80	F58	Lockdown	Increased c	0.4
Map4	F80	F124	Lockdown	Not visiting	0.6
Map4	F80	F78	Lockdown	Less food t	0.6
Map4	F80	F70	Lockdown	Jobs lost	0.6
Map4	F80	F14	Lockdown	Could not g	0.8
Map4	F80	F143	Lockdown	Shortage o	0.6
Map4	F80	F142	Lockdown	School/mad	0.8
Map4	F80	F147	Lockdown	Stress and	0.4
Map4	F80	F120	Lockdown	No transpo	0.8
Map4	F80	F144	Lockdown	Social dista	0.8
Map4	F80	F107	Lockdown	No covid-1	0.8
Map4	F80	F106	Lockdown	No congreg	0.6
Map4	F83	F01	Malnutrition	Bad experi	0.4
Map4	F20	F01	Difficult bre	Bad experi	0.4
Map4	F69	F01	Increased t	Bad experi	0.4

For Peer Review

Map4	F69	F67	Increased t	Increased s	0.6
Map4	F69	F58	Increased t	Increased c	0.4
Map4	F67	F01	Increased s	Bad experi	0.4
Map4	F29	F20	Fear of cor	Difficult bre	0.4
Map4	F29	F01	Fear of cor	Bad experi	0.4
Map4	F29	F30	Fear of cor	Fear of dea	0.4
Map4	F29	F31	Fear of cor	Fear of eac	0.8
Map4	F29	F10	Fear of cor	Compulsor	0.4
Map4	F64	F41	Increased p	Husbands	0.4
Map4	F64	F40	Increased p	Household	0.6
Map4	F64	F88	Increased p	Men unable	0.6
Map4	F64	F47	Increased p	Increase in	0.4
Map4	F64	F01	Increased p	Bad experi	0.4
Map4	F64	F62	Increased p	Increased r	0.4
Map4	F63	F01	Increased c	Bad experi	0.6
Map4	F62	F01	Increased r	Bad experi	0.4
Map4	F60	F01	Increased i	Bad experi	0.4
Map4	F60	F54	Increased i	Increase in	0.4
Map4	F158	F01	Youth/child	Bad experi	0.6
Map4	F158	F60	Youth/child	Increased i	0.4
Map4	F158	F16	Youth/child	Delay in ec	0.6
Map4	F158	F54	Youth/child	Increase in	0.4
Map4	F16	F01	Delay in ec	Bad experi	0.6
Map4	F118	F01	No social g	Bad experi	0.4
Map4	F118	F147	No social g	Stress and	0.4
Map4	F96	F47	More hung	Increase in	0.6
Map4	F96	F83	More hung	Malnutritior	0.4
Map4	F96	F01	More hung	Bad experi	0.6
Map4	F96	F63	More hung	Increased c	0.6
Map4	F130	F01	Physical in	Bad experi	0.6
Map4	F110	F01	No funerals	Bad experi	0.4
Map4	F110	F147	No funerals	Stress and	0.4
Map4	F54	F01	Increase in	Bad experi	0.4
Map4	F51	F01	Increase in	Bad experi	0.4
Map4	F58	F01	Increased c	Bad experi	0.6
Map4	F124	F01	Not visiting	Bad experi	0.6
Map4	F124	F63	Not visiting	Increased c	0.8
Map4	F30	F01	Fear of dea	Bad experi	0.2
Map4	F31	F01	Fear of eac	Bad experi	0.2
Map4	F31	F30	Fear of eac	Fear of dea	0.4
Map4	F78	F47	Less food t	Increase in	0.6
Map4	F78	F83	Less food t	Malnutritior	0.4
Map4	F78	F01	Less food t	Bad experi	0.6
Map4	F78	F63	Less food t	Increased c	0.6
Map4	F78	F96	Less food t	More hung	0.6
Map4	F13	F01	Could not c	Bad experi	0.4
Map4	F13	F60	Could not c	Increased i	0.4
Map4	F13	F158	Could not c	Youth/child	0.4
Map4	F13	F16	Could not c	Delay in ec	0.4
Map4	F13	F130	Could not c	Physical in	0.4
Map4	F13	F54	Could not c	Increase in	0.4
Map4	F10	F20	Compulsor	Difficult bre	0.6
Map4	F10	F01	Compulsor	Bad experi	0.4
Map4	F70	F41	Jobs lost	Husbands	0.4
Map4	F70	F40	Jobs lost	Household	0.6
Map4	F70	F88	Jobs lost	Men unable	0.6

For Peer Review

Map4	F70	F47	Jobs lost	Increase in	0.4
Map4	F70	F01	Jobs lost	Bad experi	0.4
Map4	F70	F64	Jobs lost	Increased p	0.8
Map4	F70	F62	Jobs lost	Increased r	0.4
Map4	F14	F01	Could not g	Bad experi	0.6
Map4	F14	F63	Could not g	Increased c	0.6
Map4	F14	F124	Could not g	Not visiting	0.6
Map4	F143	F47	Shortage o	Increase in	0.6
Map4	F143	F83	Shortage o	Malnutrition	0.4
Map4	F143	F01	Shortage o	Bad experi	0.6
Map4	F143	F63	Shortage o	Increased c	0.6
Map4	F143	F96	Shortage o	More hung	0.6
Map4	F143	F78	Shortage o	Less food t	0.6
Map4	F142	F01	School/ma	Bad experi	0.6
Map4	F142	F69	School/ma	Increased t	0.4
Map4	F142	F67	School/ma	Increased s	0.4
Map4	F142	F60	School/ma	Increased i	0.4
Map4	F142	F158	School/ma	Youth/child	0.8
Map4	F142	F16	School/ma	Delay in ec	0.6
Map4	F142	F54	School/ma	Increase in	0.4
Map4	F142	F51	School/ma	Increase in	0.4
Map4	F142	F58	School/ma	Increased c	0.4
Map4	F147	F01	Stress and	Bad experi	1
Map4	F120	F01	No transpo	Bad experi	0.6
Map4	F120	F63	No transpo	Increased c	0.6
Map4	F120	F124	No transpo	Not visiting	0.6
Map4	F120	F14	No transpo	Could not g	0.8
Map4	F120	F107	No transpo	No covid-1	1
Map4	F144	F01	Social dista	Bad experi	0.4
Map4	F144	F118	Social dista	No social g	0.6
Map4	F144	F110	Social dista	No funerals	0.4
Map4	F144	F147	Social dista	Stress and	0.4
Map4	F144	F106	Social dista	No congreg	0.6
Map4	F107	F01	No covid-1	Bad experi	0.6
Map4	F107	F63	No covid-1	Increased c	0.6
Map4	F107	F124	No covid-1	Not visiting	0.6
Map4	F107	F14	No covid-1	Could not g	0.8
Map4	F106	F01	No congreg	Bad experi	0.4
Map4	F106	F118	No congreg	No social g	0.6
Map4	F106	F110	No congreg	No funerals	0.4
Map4	F106	F147	No congreg	Stress and	0.4
Map5	F40	F01	Household	Bad experi	0.8
Map5	F88	F40	Men unable	Household	0.8
Map5	F88	F01	Men unable	Bad experi	0.8
Map5	F85	F40	Markets/sh	Household	0.6
Map5	F85	F88	Markets/sh	Men unable	0.6
Map5	F85	F01	Markets/sh	Bad experi	0.6
Map5	F85	F70	Markets/sh	Jobs lost	0.6
Map5	F48	F01	Increase in	Bad experi	0.8
Map5	F80	F40	Lockdown	Household	0.6
Map5	F80	F88	Lockdown	Men unable	0.6
Map5	F80	F85	Lockdown	Markets/sh	0.8
Map5	F80	F48	Lockdown	Increase in	0.6
Map5	F80	F01	Lockdown	Bad experi	0.8
Map5	F80	F62	Lockdown	Increased r	0.8
Map5	F80	F60	Lockdown	Increased i	0.6

For Peer Review

Map5	F80	F158	Lockdown	Youth/child	0.6
Map5	F80	F70	Lockdown	Jobs lost	0.8
Map5	F80	F94	Lockdown	More hous	0.4
Map5	F80	F96	Lockdown	More hung	0.6
Map5	F80	F135	Lockdown	Price hike	0.6
Map5	F80	F51	Lockdown	Increase in	0.6
Map5	F80	F78	Lockdown	Less food t	0.6
Map5	F80	F16	Lockdown	Delay in ec	0.6
Map5	F80	F143	Lockdown	Shortage o	0.6
Map5	F80	F142	Lockdown	School/ma	0.8
Map5	F80	F147	Lockdown	Stress and	0.8
Map5	F80	F120	Lockdown	No transpo	1
Map5	F80	F123	Lockdown	Not going t	0.6
Map5	F80	F106	Lockdown	No congreg	0.8
Map5	F80	F100	Lockdown	Mosques/c	1
Map5	F20	F01	Difficult bre	Bad experi	0.4
Map5	F03	F01	Afraid to vi	Bad experi	0.6
Map5	F03	F63	Afraid to vi	Increased	0.6
Map5	F29	F01	Fear of cor	Bad experi	0.6
Map5	F29	F03	Fear of cor	Afraid to vi	0.8
Map5	F29	F63	Fear of cor	Increased	0.6
Map5	F28	F01	Fear of clin	Bad experi	0.4
Map5	F28	F03	Fear of clin	Afraid to vi	0.4
Map5	F28	F63	Fear of clin	Increased	0.4
Map5	F63	F01	Increased	Bad experi	0.8
Map5	F62	F01	Increased	Bad experi	0.6
Map5	F60	F01	Increased i	Bad experi	0.8
Map5	F154	F20	Wearing fa	Difficult bre	0.2
Map5	F154	F01	Wearing fa	Bad experi	0.2
Map5	F158	F48	Youth/child	Increase in	0.6
Map5	F158	F01	Youth/child	Bad experi	0.6
Map5	F158	F62	Youth/child	Increased	0.4
Map5	F158	F60	Youth/child	Increased i	0.6
Map5	F158	F94	Youth/child	More hous	0.4
Map5	F158	F51	Youth/child	Increase in	0.6
Map5	F158	F16	Youth/child	Delay in ec	0.6
Map5	F158	F147	Youth/child	Stress and	0.4
Map5	F70	F40	Jobs lost	Household	0.6
Map5	F70	F88	Jobs lost	Men unable	0.6
Map5	F70	F01	Jobs lost	Bad experi	0.6
Map5	F118	F01	No social g	Bad experi	0.8
Map5	F118	F62	No social g	Increased	0.8
Map5	F118	F147	No social g	Stress and	0.8
Map5	F118	F106	No social g	No congreg	0.8
Map5	F94	F01	More hous	Bad experi	0.6
Map5	F94	F62	More hous	Increased	0.6
Map5	F94	F147	More hous	Stress and	0.6
Map5	F96	F01	More hung	Bad experi	1
Map5	F135	F40	Price hike	Household	0.6
Map5	F135	F01	Price hike	Bad experi	0.6
Map5	F135	F96	Price hike	More hung	0.6
Map5	F135	F78	Price hike	Less food t	0.6
Map5	F51	F48	Increase in	Increase in	0.6
Map5	F51	F01	Increase in	Bad experi	0.6
Map5	F51	F60	Increase in	Increased i	0.6
Map5	F78	F40	Less food t	Household	0.6

For Peer Review

Map5	F78	F01	Less food t	Bad experi	0.8
Map5	F78	F96	Less food t	More hung	0.8
Map5	F16	F48	Delay in ed	Increase in	0.6
Map5	F16	F01	Delay in ed	Bad experi	0.6
Map5	F16	F60	Delay in ed	Increased i	0.6
Map5	F16	F51	Delay in ed	Increase in	0.8
Map5	F143	F40	Shortage o	Household	0.6
Map5	F143	F01	Shortage o	Bad experi	0.8
Map5	F143	F96	Shortage o	More hung	0.8
Map5	F143	F135	Shortage o	Price hike	0.8
Map5	F143	F78	Shortage o	Less food t	0.8
Map5	F142	F48	School/ma	Increase in	0.6
Map5	F142	F01	School/ma	Bad experi	0.6
Map5	F142	F62	School/ma	Increased r	0.4
Map5	F142	F60	School/ma	Increased i	0.6
Map5	F142	F158	School/ma	Youth/child	0.6
Map5	F142	F94	School/ma	More hous	0.4
Map5	F142	F51	School/ma	Increase in	0.6
Map5	F142	F16	School/ma	Delay in ed	0.6
Map5	F142	F147	School/ma	Stress and	0.4
Map5	F147	F01	Stress and	Bad experi	0.8
Map5	F147	F62	Stress and	Increased r	0.8
Map5	F120	F40	No transpo	Household	0.6
Map5	F120	F88	No transpo	Men unable	0.6
Map5	F120	F01	No transpo	Bad experi	0.6
Map5	F120	F70	No transpo	Jobs lost	0.8
Map5	F120	F96	No transpo	More hung	0.6
Map5	F120	F135	No transpo	Price hike	0.6
Map5	F120	F78	No transpo	Less food t	0.6
Map5	F120	F143	No transpo	Shortage o	0.6
Map5	F120	F123	No transpo	Not going t	0.6
Map5	F123	F40	Not going t	Household	0.6
Map5	F123	F88	Not going t	Men unable	0.4
Map5	F123	F01	Not going t	Bad experi	0.6
Map5	F123	F70	Not going t	Jobs lost	0.4
Map5	F123	F96	Not going t	More hung	0.6
Map5	F123	F135	Not going t	Price hike	0.6
Map5	F123	F78	Not going t	Less food t	0.6
Map5	F123	F143	Not going t	Shortage o	0.6
Map5	F144	F01	Social dista	Bad experi	0.6
Map5	F144	F62	Social dista	Increased r	0.6
Map5	F144	F118	Social dista	No social g	0.6
Map5	F144	F147	Social dista	Stress and	0.6
Map5	F144	F106	Social dista	No congreg	0.6
Map5	F106	F01	No congreg	Bad experi	0.8
Map5	F106	F62	No congreg	Increased r	0.8
Map5	F106	F147	No congreg	Stress and	0.8
Map5	F100	F01	Mosques/c	Bad experi	0.8
Map5	F100	F62	Mosques/c	Increased r	0.8
Map5	F100	F147	Mosques/c	Stress and	0.8
Map5	F100	F106	Mosques/c	No congreg	0.8
Map6	F40	F47	Household	Increase in	0.4
Map6	F40	F01	Household	Bad experi	0.4
Map6	F40	F156	Household	Wives sent	0.4
Map6	F40	F91	Household	More burde	0.4
Map6	F40	F147	Household	Stress and	0.4

For Peer Review

Map6	F88	F40	Men unabl	Household	0.6
Map6	F88	F47	Men unabl	Increase in	0.4
Map6	F88	F01	Men unabl	Bad experi	0.4
Map6	F88	F156	Men unabl	Wives sent	0.4
Map6	F88	F91	Men unabl	More burde	0.4
Map6	F88	F147	Men unabl	Stress and	0.4
Map6	F47	F01	Increase in	Bad experi	0.2
Map6	F85	F40	Markets/sh	Household	0.6
Map6	F85	F88	Markets/sh	Men unabl	0.6
Map6	F85	F47	Markets/sh	Increase in	0.4
Map6	F85	F82	Markets/sh	Lost invest	0.6
Map6	F85	F07	Markets/sh	Businesses	0.8
Map6	F85	F01	Markets/sh	Bad experi	0.6
Map6	F85	F64	Markets/sh	Increased p	0.6
Map6	F85	F156	Markets/sh	Wives sent	0.4
Map6	F85	F91	Markets/sh	More burde	0.4
Map6	F85	F147	Markets/sh	Stress and	0.6
Map6	F85	F121	Markets/sh	No wedding	0.6
Map6	F87	F40	Men stayin	Household	0.6
Map6	F87	F88	Men stayin	Men unabl	0.6
Map6	F87	F47	Men stayin	Increase in	0.4
Map6	F87	F01	Men stayin	Bad experi	0.4
Map6	F87	F156	Men stayin	Wives sent	0.4
Map6	F87	F91	Men stayin	More burde	0.4
Map6	F87	F147	Men stayin	Stress and	0.4
Map6	F80	F40	Lockdown	Household	0.6
Map6	F80	F88	Lockdown	Men unabl	0.6
Map6	F80	F47	Lockdown	Increase in	0.4
Map6	F80	F85	Lockdown	Markets/sh	0.8
Map6	F80	F87	Lockdown	Men stayin	1
Map6	F80	F82	Lockdown	Lost invest	0.6
Map6	F80	F04	Lockdown	Bad use of	0.7
Map6	F80	F07	Lockdown	Businesses	0.8
Map6	F80	F01	Lockdown	Bad experi	0.7
Map6	F80	F60	Lockdown	Increased i	0.7
Map6	F80	F64	Lockdown	Increased p	0.6
Map6	F80	F156	Lockdown	Wives sent	0.4
Map6	F80	F158	Lockdown	Youth/child	0.7
Map6	F80	F16	Lockdown	Delay in ec	0.6
Map6	F80	F91	Lockdown	More burde	0.4
Map6	F80	F147	Lockdown	Stress and	0.6
Map6	F80	F70	Lockdown	Jobs lost	0.6
Map6	F80	F142	Lockdown	School/ma	1
Map6	F80	F121	Lockdown	No wedding	0.6
Map6	F80	F106	Lockdown	No congreg	0.6
Map6	F80	F100	Lockdown	Mosques/c	1
Map6	F82	F01	Lost invest	Bad experi	0.8
Map6	F82	F64	Lost invest	Increased p	0.8
Map6	F82	F147	Lost invest	Stress and	0.6
Map6	F82	F121	Lost invest	No wedding	0.8
Map6	F04	F01	Bad use of	Bad experi	0.8
Map6	F04	F60	Bad use of	Increased i	0.8
Map6	F07	F40	Businesses	Household	0.6
Map6	F07	F88	Businesses	Men unabl	0.6
Map6	F07	F47	Businesses	Increase in	0.4
Map6	F07	F82	Businesses	Lost invest	0.6

For Peer Review

Map6	F07	F01	Businesses	Bad experi	0.6
Map6	F07	F64	Businesses	Increased p	0.6
Map6	F07	F156	Businesses	Wives sent	0.4
Map6	F07	F91	Businesses	More burde	0.4
Map6	F07	F147	Businesses	Stress and	0.6
Map6	F07	F121	Businesses	No wedding	0.6
Map6	F20	F01	Difficult bre	Bad experi	0.4
Map6	F03	F01	Afraid to vis	Bad experi	0.8
Map6	F03	F63	Afraid to vis	Increased c	0.8
Map6	F29	F01	Fear of cor	Bad experi	0.8
Map6	F29	F03	Fear of cor	Afraid to vis	0.8
Map6	F29	F63	Fear of cor	Increased c	0.8
Map6	F29	F31	Fear of cor	Fear of eac	0.2
Map6	F28	F01	Fear of clin	Bad experi	0.6
Map6	F28	F03	Fear of clin	Afraid to vis	0.6
Map6	F28	F63	Fear of clin	Increased c	0.6
Map6	F63	F01	Increased c	Bad experi	1
Map6	F60	F01	Increased i	Bad experi	0.8
Map6	F64	F01	Increased p	Bad experi	1
Map6	F64	F147	Increased p	Stress and	0.6
Map6	F64	F121	Increased p	No wedding	0.8
Map6	F156	F01	Wives sent	Bad experi	0.4
Map6	F156	F91	Wives sent	More burde	0.4
Map6	F156	F147	Wives sent	Stress and	0.4
Map6	F158	F04	Youth/child	Bad use of	0.8
Map6	F158	F01	Youth/child	Bad experi	0.8
Map6	F158	F60	Youth/child	Increased i	0.8
Map6	F158	F16	Youth/child	Delay in ec	0.6
Map6	F16	F01	Delay in ec	Bad experi	0.6
Map6	F91	F01	More burde	Bad experi	0.6
Map6	F91	F147	More burde	Stress and	0.6
Map6	F118	F01	No social g	Bad experi	0.6
Map6	F118	F64	No social g	Increased p	0.6
Map6	F118	F147	No social g	Stress and	0.6
Map6	F118	F121	No social g	No wedding	0.6
Map6	F96	F01	More hung	Bad experi	1
Map6	F96	F63	More hung	Increased c	1
Map6	F112	F82	No home/p	Lost invest	0.6
Map6	F112	F01	No home/p	Bad experi	0.6
Map6	F112	F64	No home/p	Increased p	0.6
Map6	F112	F147	No home/p	Stress and	0.6
Map6	F112	F121	No home/p	No wedding	0.6
Map6	F30	F01	Fear of dea	Bad experi	0.8
Map6	F30	F03	Fear of dea	Afraid to vis	0.8
Map6	F30	F29	Fear of dea	Fear of cor	0.8
Map6	F30	F63	Fear of dea	Increased c	0.8
Map6	F30	F31	Fear of dea	Fear of eac	0.2
Map6	F31	F01	Fear of eac	Bad experi	0.6
Map6	F78	F01	Less food t	Bad experi	0.6
Map6	F78	F63	Less food t	Increased c	0.6
Map6	F78	F96	Less food t	More hung	0.6
Map6	F10	F20	Compulsor	Difficult bre	0.2
Map6	F10	F01	Compulsor	Bad experi	0.2
Map6	F70	F40	Jobs lost	Household	0.6
Map6	F70	F88	Jobs lost	Men unable	0.6
Map6	F70	F47	Jobs lost	Increase in	0.4

For Peer Review

Map6	F70	F01	Jobs lost	Bad experi	0.4
Map6	F70	F156	Jobs lost	Wives sent	0.4
Map6	F70	F91	Jobs lost	More burde	0.4
Map6	F70	F147	Jobs lost	Stress and	0.4
Map6	F143	F01	Shortage o	Bad experi	0.6
Map6	F143	F63	Shortage o	Increased c	0.6
Map6	F143	F96	Shortage o	More hung	0.6
Map6	F143	F78	Shortage o	Less food t	0.8
Map6	F142	F04	School/mac	Bad use of	0.7
Map6	F142	F01	School/mac	Bad experi	0.7
Map6	F142	F60	School/mac	Increased i	0.7
Map6	F142	F158	School/mac	Youth/child	0.7
Map6	F142	F16	School/mac	Delay in ec	0.6
Map6	F140	F82	Restriction	Lost invest	0.4
Map6	F140	F01	Restriction	Bad experi	0.4
Map6	F140	F63	Restriction	Increased c	0.4
Map6	F140	F64	Restriction	Increased p	0.4
Map6	F140	F96	Restriction	More hung	0.4
Map6	F140	F112	Restriction	No home/p	0.4
Map6	F140	F78	Restriction	Less food t	0.4
Map6	F140	F147	Restriction	Stress and	0.4
Map6	F140	F143	Restriction	Shortage o	0.4
Map6	F140	F121	Restriction	No wedding	0.4
Map6	F140	F120	Restriction	No transpo	0.4
Map6	F140	F104	Restriction	No buyers	0.6
Map6	F121	F147	No wedding	Stress and	0.6
Map6	F120	F01	No transpo	Bad experi	0.6
Map6	F120	F63	No transpo	Increased c	0.6
Map6	F120	F96	No transpo	More hung	0.6
Map6	F120	F78	No transpo	Less food t	0.6
Map6	F120	F143	No transpo	Shortage o	0.6
Map6	F144	F01	Social dista	Bad experi	0.6
Map6	F144	F64	Social dista	Increased p	0.6
Map6	F144	F118	Social dista	No social g	0.8
Map6	F144	F147	Social dista	Stress and	0.6
Map6	F144	F121	Social dista	No wedding	0.6
Map6	F144	F106	Social dista	No congreg	0.6
Map6	F106	F147	No congreg	Stress and	0.8
Map6	F104	F82	No buyers	Lost invest	0.4
Map6	F104	F01	No buyers	Bad experi	0.4
Map6	F104	F64	No buyers	Increased p	0.4
Map6	F104	F112	No buyers	No home/p	0.4
Map6	F104	F147	No buyers	Stress and	0.4
Map6	F104	F121	No buyers	No wedding	0.4
Map6	F100	F147	Mosques/c	Stress and	0.6
Map6	F100	F106	Mosques/c	No congreg	0.6
Map7	F85	F01	Markets/sh	Bad experi	0.8
Map7	F85	F64	Markets/sh	Increased p	0.8
Map7	F85	F63	Markets/sh	Increased c	0.8
Map7	F85	F96	Markets/sh	More hung	0.8
Map7	F85	F78	Markets/sh	Less food t	0.8
Map7	F85	F70	Markets/sh	Jobs lost	1
Map7	F80	F85	Lockdown	Markets/sh	0.8
Map7	F80	F01	Lockdown	Bad experi	0.8
Map7	F80	F64	Lockdown	Increased p	0.8
Map7	F80	F63	Lockdown	Increased c	0.8

For Peer Review

Map7	F80	F158	Lockdown	Youth/child	1
Map7	F80	F96	Lockdown	More hung	0.8
Map7	F80	F114	Lockdown	No pilgrima	0.8
Map7	F80	F133	Lockdown	Poor perfor	0.6
Map7	F80	F110	Lockdown	No funerals	0.2
Map7	F80	F112	Lockdown	No home/p	0.6
Map7	F80	F135	Lockdown	Price hike	0.8
Map7	F80	F142	Lockdown	School/mad	1
Map7	F80	F118	Lockdown	No social g	0.2
Map7	F80	F78	Lockdown	Less food t	0.8
Map7	F80	F70	Lockdown	Jobs lost	0.8
Map7	F80	F147	Lockdown	Stress and	0.8
Map7	F80	F120	Lockdown	No transpo	1
Map7	F80	F106	Lockdown	No congreg	0.8
Map7	F80	F104	Lockdown	No buyers	0.6
Map7	F80	F100	Lockdown	Mosques/c	1
Map7	F74	F01	Lack of fac	Bad experi	0.8
Map7	F29	F74	Fear of cor	Lack of fac	0.6
Map7	F29	F01	Fear of cor	Bad experi	0.6
Map7	F29	F63	Fear of cor	Increased	0.6
Map7	F29	F28	Fear of cor	Fear of clin	0.6
Map7	F29	F32	Fear of cor	Feeling soc	0.6
Map7	F29	F38	Fear of cor	Health wor	0.6
Map7	F29	F10	Fear of cor	Compulsor	0.6
Map7	F29	F124	Fear of cor	Not visiting	0.4
Map7	F29	F128	Fear of cor	People fee	0.6
Map7	F64	F01	Increased	Bad experi	1
Map7	F64	F63	Increased	Increased	1
Map7	F64	F96	Increased	More hung	1
Map7	F64	F78	Increased	Less food t	1
Map7	F63	F01	Increased	Bad experi	1
Map7	F28	F01	Fear of clin	Bad experi	0.4
Map7	F28	F63	Fear of clin	Increased	0.4
Map7	F28	F124	Fear of clin	Not visiting	0.4
Map7	F158	F01	Youth/child	Bad experi	0.6
Map7	F158	F133	Youth/child	Poor perfor	0.6
Map7	F158	F147	Youth/child	Stress and	0.6
Map7	F96	F01	More hung	Bad experi	1
Map7	F96	F63	More hung	Increased	1
Map7	F114	F01	No pilgrima	Bad experi	0.6
Map7	F114	F147	No pilgrima	Stress and	0.6
Map7	F133	F01	Poor perfor	Bad experi	0.8
Map7	F133	F147	Poor perfor	Stress and	0.8
Map7	F110	F01	No funerals	Bad experi	0.8
Map7	F110	F147	No funerals	Stress and	0.8
Map7	F112	F01	No home/p	Bad experi	1
Map7	F112	F64	No home/p	Increased	1
Map7	F112	F63	No home/p	Increased	1
Map7	F112	F96	No home/p	More hung	1
Map7	F112	F78	No home/p	Less food t	1
Map7	F135	F01	Price hike	Bad experi	0.6
Map7	F135	F64	Price hike	Increased	0.6
Map7	F135	F63	Price hike	Increased	0.6
Map7	F135	F96	Price hike	More hung	0.6
Map7	F135	F112	Price hike	No home/p	0.6
Map7	F135	F78	Price hike	Less food t	0.6

For Peer Review

Map7	F135	F104	Price hike	No buyers	0.6
Map7	F142	F01	School/ma	Bad experi	0.6
Map7	F142	F158	School/ma	Youth/child	1
Map7	F142	F133	School/ma	Poor perfor	0.6
Map7	F142	F147	School/ma	Stress and	0.6
Map7	F32	F74	Feeling soc	Lack of fac	0.6
Map7	F32	F01	Feeling soc	Bad experi	0.6
Map7	F32	F63	Feeling soc	Increased	0.4
Map7	F32	F28	Feeling soc	Fear of clin	0.6
Map7	F32	F10	Feeling soc	Compulsor	0.6
Map7	F32	F124	Feeling soc	Not visiting	0.4
Map7	F32	F128	Feeling soc	People fee	1
Map7	F118	F01	No social g	Bad experi	0.6
Map7	F118	F110	No social g	No funerals	0.6
Map7	F118	F147	No social g	Stress and	0.6
Map7	F78	F01	Less food t	Bad experi	1
Map7	F78	F63	Less food t	Increased	1
Map7	F78	F96	Less food t	More hung	1
Map7	F38	F01	Health wor	Bad experi	0.8
Map7	F38	F63	Health wor	Increased	0.8
Map7	F10	F74	Compulsor	Lack of fac	0.6
Map7	F10	F01	Compulsor	Bad experi	0.6
Map7	F70	F01	Jobs lost	Bad experi	0.8
Map7	F70	F64	Jobs lost	Increased	0.8
Map7	F70	F63	Jobs lost	Increased	0.8
Map7	F70	F96	Jobs lost	More hung	0.8
Map7	F70	F78	Jobs lost	Less food t	0.8
Map7	F124	F01	Not visiting	Bad experi	0.8
Map7	F124	F63	Not visiting	Increased	0.8
Map7	F147	F01	Stress and	Bad experi	0.8
Map7	F120	F01	No transpo	Bad experi	0.6
Map7	F120	F64	No transpo	Increased	0.6
Map7	F120	F63	No transpo	Increased	0.6
Map7	F120	F96	No transpo	More hung	0.6
Map7	F120	F112	No transpo	No home/p	0.6
Map7	F120	F135	No transpo	Price hike	0.8
Map7	F120	F78	No transpo	Less food t	0.6
Map7	F120	F104	No transpo	No buyers	0.6
Map7	F128	F01	People feel	Bad experi	0.4
Map7	F128	F63	People feel	Increased	0.4
Map7	F128	F28	People feel	Fear of clin	0.6
Map7	F128	F124	People feel	Not visiting	0.4
Map7	F106	F01	No congreg	Bad experi	0.8
Map7	F106	F147	No congreg	Stress and	0.8
Map7	F106	F106	No congreg	No congreg	0.6
Map7	F104	F01	No buyers	Bad experi	0.8
Map7	F104	F64	No buyers	Increased	0.8
Map7	F104	F63	No buyers	Increased	0.8
Map7	F104	F96	No buyers	More hung	0.8
Map7	F104	F112	No buyers	No home/p	0.8
Map7	F104	F78	No buyers	Less food t	0.8
Map7	F100	F01	Mosques/c	Bad experi	0.8
Map7	F100	F147	Mosques/c	Stress and	0.8
Map7	F100	F106	Mosques/c	No congreg	0.8
Map8	F85	F83	Markets/sh	Malnutrition	0.4
Map8	F85	F01	Markets/sh	Bad experi	0.4

For Peer Review

Map8	F85	F64	Markets/sh	Increased	0.4
Map8	F85	F63	Markets/sh	Increased	0.4
Map8	F85	F96	Markets/sh	More hung	0.4
Map8	F85	F137	Markets/sh	Reduced s	0.4
Map8	F85	F135	Markets/sh	Price hike	0.4
Map8	F85	F78	Markets/sh	Less food t	0.4
Map8	F85	F70	Markets/sh	Jobs lost	0.6
Map8	F85	F143	Markets/sh	Shortage o	0.4
Map8	F80	F85	Lockdown	Markets/sh	0.4
Map8	F80	F83	Lockdown	Malnutritior	0.6
Map8	F80	F01	Lockdown	Bad experi	0.8
Map8	F80	F64	Lockdown	Increased	0.4
Map8	F80	F63	Lockdown	Increased	0.6
Map8	F80	F62	Lockdown	Increased	0.6
Map8	F80	F96	Lockdown	More hung	0.6
Map8	F80	F114	Lockdown	No pilgrima	0.6
Map8	F80	F115	Lockdown	No preachi	0.6
Map8	F80	F117	Lockdown	No religiou	0.6
Map8	F80	F136	Lockdown	Reduced p	0.4
Map8	F80	F137	Lockdown	Reduced s	0.6
Map8	F80	F135	Lockdown	Price hike	0.6
Map8	F80	F32	Lockdown	Feeling soc	0.4
Map8	F80	F78	Lockdown	Less food t	0.6
Map8	F80	F121	Lockdown	No wedding	0.4
Map8	F80	F70	Lockdown	Jobs lost	0.4
Map8	F80	F143	Lockdown	Shortage o	0.6
Map8	F80	F140	Lockdown	Restriction	0.8
Map8	F80	F147	Lockdown	Stress and	0.8
Map8	F80	F120	Lockdown	No transpo	1
Map8	F80	F144	Lockdown	Social dista	0.6
Map8	F80	F106	Lockdown	No congreg	0.8
Map8	F80	F102	Lockdown	No access	0.6
Map8	F80	F100	Lockdown	Mosques/c	1
Map8	F83	F01	Malnutritior	Bad experi	0.8
Map8	F83	F63	Malnutritior	Increased	0.6
Map8	F20	F01	Difficult bre	Bad experi	0.6
Map8	F29	F20	Fear of cor	Difficult bre	0.2
Map8	F29	F01	Fear of cor	Bad experi	0.2
Map8	F29	F154	Fear of cor	Wearing fa	0.4
Map8	F29	F31	Fear of cor	Fear of eac	0.4
Map8	F64	F01	Increased	Bad experi	0.7
Map8	F64	F64	Increased	Increased	0.4
Map8	F63	F01	Increased	Bad experi	0.8
Map8	F62	F01	Increased	Bad experi	0.8
Map8	F154	F20	Wearing fa	Difficult bre	0.2
Map8	F154	F01	Wearing fa	Bad experi	0.2
Map8	F96	F83	More hung	Malnutritior	0.6
Map8	F96	F01	More hung	Bad experi	0.6
Map8	F96	F63	More hung	Increased	0.6
Map8	F96	F96	More hung	More hung	0.6
Map8	F96	F137	More hung	Reduced s	0.6
Map8	F96	F135	More hung	Price hike	0.6
Map8	F96	F78	More hung	Less food t	0.6
Map8	F96	F143	More hung	Shortage o	0.6
Map8	F114	F01	No pilgrima	Bad experi	0.6
Map8	F114	F62	No pilgrima	Increased	0.6

For Peer Review

Map8	F115	F01	No preachi	Bad experi	0.4
Map8	F115	F62	No preachi	Increased r	0.4
Map8	F115	F147	No preachi	Stress and	0.4
Map8	F117	F01	No religiou	Bad experi	0.6
Map8	F117	F62	No religiou	Increased r	0.6
Map8	F117	F147	No religiou	Stress and	0.6
Map8	F136	F01	Reduced p	Bad experi	0.4
Map8	F136	F63	Reduced p	Increased c	0.4
Map8	F137	F01	Reduced s	Bad experi	0.6
Map8	F135	F01	Price hike	Bad experi	0.4
Map8	F135	F137	Price hike	Reduced s	0.4
Map8	F135	F78	Price hike	Less food t	0.8
Map8	F31	F20	Fear of eac	Difficult bre	0.2
Map8	F31	F01	Fear of eac	Bad experi	0.2
Map8	F31	F154	Fear of eac	Wearing fa	0.4
Map8	F32	F01	Feeling soc	Bad experi	0.6
Map8	F32	F62	Feeling soc	Increased r	0.6
Map8	F78	F01	Less food t	Bad experi	0.4
Map8	F78	F137	Less food t	Reduced s	0.4
Map8	F12	F01	Constant m	Bad experi	0.2
Map8	F12	F62	Constant m	Increased r	0.2
Map8	F12	F147	Constant m	Stress and	0.2
Map8	F121	F01	No wedding	Bad experi	0.2
Map8	F121	F62	No wedding	Increased r	0.2
Map8	F121	F147	No wedding	Stress and	0.2
Map8	F70	F01	Jobs lost	Bad experi	0.4
Map8	F70	F64	Jobs lost	Increased p	0.4
Map8	F143	F83	Shortage o	Malnutrition	0.6
Map8	F143	F01	Shortage o	Bad experi	0.8
Map8	F143	F63	Shortage o	Increased c	0.8
Map8	F143	F96	Shortage o	More hung	0.8
Map8	F143	F137	Shortage o	Reduced s	0.6
Map8	F143	F135	Shortage o	Price hike	0.6
Map8	F143	F78	Shortage o	Less food t	0.6
Map8	F143	F143	Shortage o	Shortage o	0.6
Map8	F141	F20	Rumors on	Difficult bre	0.2
Map8	F141	F01	Rumors on	Bad experi	0.2
Map8	F141	F154	Rumors on	Wearing fa	0.4
Map8	F141	F31	Rumors on	Fear of eac	0.4
Map8	F140	F01	Restriction	Bad experi	0.6
Map8	F140	F63	Restriction	Increased c	0.4
Map8	F140	F62	Restriction	Increased r	0.6
Map8	F140	F114	Restriction	No pilgrima	0.6
Map8	F140	F136	Restriction	Reduced p	0.4
Map8	F140	F121	Restriction	No wedding	0.4
Map8	F140	F147	Restriction	Stress and	0.6
Map8	F140	F102	Restriction	No access	0.6
Map8	F147	F01	Stress and	Bad experi	0.8
Map8	F147	F62	Stress and	Increased r	0.6
Map8	F120	F85	No transpo	Markets/sh	0.4
Map8	F120	F83	No transpo	Malnutrition	0.6
Map8	F120	F01	No transpo	Bad experi	0.6
Map8	F120	F64	No transpo	Increased p	0.4
Map8	F120	F63	No transpo	Increased c	0.6
Map8	F120	F96	No transpo	More hung	0.6
Map8	F120	F137	No transpo	Reduced s	0.6

For Peer Review

Map8	F120	F135	No transpo	Price hike	0.6
Map8	F120	F78	No transpo	Less food t	0.6
Map8	F120	F70	No transpo	Jobs lost	0.4
Map8	F120	F143	No transpo	Shortage o	0.6
Map8	F144	F01	Social dista	Bad experi	0.4
Map8	F144	F62	Social dista	Increased i	0.4
Map8	F144	F32	Social dista	Feeling soc	0.4
Map8	F111	F01	No governr	Bad experi	0.6
Map8	F111	F64	No governr	Increased p	0.6
Map8	F106	F01	No congreg	Bad experi	1
Map8	F106	F62	No congreg	Increased i	0.6
Map8	F106	F147	No congreg	Stress and	0.8
Map8	F102	F01	No access	Bad experi	0.6
Map8	F102	F62	No access	Increased i	0.6
Map8	F102	F147	No access	Stress and	0.6
Map8	F100	F01	Mosques/c	Bad experi	0.8
Map8	F100	F62	Mosques/c	Increased i	0.6
Map8	F100	F115	Mosques/c	No preachi	0.6
Map8	F100	F117	Mosques/c	No religiou	0.6
Map8	F100	F147	Mosques/c	Stress and	0.8
Map8	F100	F106	Mosques/c	No congreg	0.8
Map9	F40	F47	Household	Increase in	0.2
Map9	F40	F01	Household	Bad experi	0.2
Map9	F88	F40	Men unabl	Household	0.4
Map9	F88	F47	Men unabl	Increase in	0.2
Map9	F88	F01	Men unabl	Bad experi	0.2
Map9	F47	F01	Increase in	Bad experi	0.2
Map9	F49	F01	Increase in	Bad experi	0.2
Map9	F49	F99	Increase in	More unwa	0.2
Map9	F87	F40	Men stayin	Household	0.4
Map9	F87	F88	Men stayin	Men unabl	0.6
Map9	F87	F47	Men stayin	Increase in	0.2
Map9	F87	F01	Men stayin	Bad experi	0.2
Map9	F80	F40	Lockdown	Household	0.4
Map9	F80	F88	Lockdown	Men unabl	0.6
Map9	F80	F47	Lockdown	Increase in	0.2
Map9	F80	F49	Lockdown	Increase in	0.6
Map9	F80	F87	Lockdown	Men stayin	0.8
Map9	F80	F82	Lockdown	Lost invest	0.6
Map9	F80	F07	Lockdown	Businesses	0.6
Map9	F80	F01	Lockdown	Bad experi	0.8
Map9	F80	F69	Lockdown	Increased t	0.6
Map9	F80	F67	Lockdown	Increased s	0.6
Map9	F80	F65	Lockdown	Increased i	0.6
Map9	F80	F64	Lockdown	Increased p	0.6
Map9	F80	F63	Lockdown	Increased c	0.8
Map9	F80	F08	Lockdown	Cannot cor	0.8
Map9	F80	F60	Lockdown	Increased i	0.6
Map9	F80	F158	Lockdown	Youth/child	0.8
Map9	F80	F85	Lockdown	Markets/sh	0.8
Map9	F80	F96	Lockdown	More hung	0.6
Map9	F80	F130	Lockdown	Physical in	0.4
Map9	F80	F99	Lockdown	More unwa	0.2
Map9	F80	F124	Lockdown	Not visiting	0.8
Map9	F80	F58	Lockdown	Increased c	0.6
Map9	F80	F59	Lockdown	Increased g	0.6

For Peer Review

Map9	F80	F78	Lockdown	Less food t	0.6
Map9	F80	F133	Lockdown	Poor perfor	0.6
Map9	F80	F13	Lockdown	Could not c	0.6
Map9	F80	F16	Lockdown	Delay in ec	0.6
Map9	F80	F98	Lockdown	More mate	0.4
Map9	F80	F14	Lockdown	Could not g	0.8
Map9	F80	F142	Lockdown	School/mac	1
Map9	F80	F147	Lockdown	Stress and	0.2
Map9	F80	F120	Lockdown	No transpo	1
Map9	F80	F115	Lockdown	No preachi	0.6
Map9	F80	F106	Lockdown	No congreg	0.8
Map9	F80	F100	Lockdown	Mosques/c	1
Map9	F82	F01	Lost invest	Bad experi	0.6
Map9	F82	F64	Lost invest	Increased p	0.8
Map9	F82	F96	Lost invest	More hung	0.6
Map9	F82	F78	Lost invest	Less food t	0.6
Map9	F07	F82	Businesses	Lost invest	0.6
Map9	F07	F01	Businesses	Bad experi	0.6
Map9	F07	F64	Businesses	Increased p	0.6
Map9	F07	F96	Businesses	More hung	0.6
Map9	F07	F78	Businesses	Less food t	0.6
Map9	F20	F01	Difficult bre	Bad experi	0.2
Map9	F69	F01	Increased t	Bad experi	0.4
Map9	F67	F01	Increased s	Bad experi	0.6
Map9	F65	F01	Increased r	Bad experi	0.2
Map9	F65	F130	Increased r	Physical in	0.4
Map9	F64	F01	Increased p	Bad experi	0.6
Map9	F64	F96	Increased p	More hung	0.6
Map9	F64	F78	Increased p	Less food t	0.6
Map9	F63	F01	Increased c	Bad experi	1
Map9	F08	F01	Cannot cor	Bad experi	0.4
Map9	F08	F98	Cannot cor	More mate	0.4
Map9	F60	F49	Increased i	Increase in	0.6
Map9	F60	F01	Increased i	Bad experi	0.6
Map9	F60	F69	Increased i	Increased t	0.6
Map9	F60	F67	Increased i	Increased s	0.6
Map9	F60	F65	Increased i	Increased r	0.6
Map9	F60	F130	Increased i	Physical in	0.4
Map9	F60	F99	Increased i	More unwa	0.2
Map9	F60	F58	Increased i	Increased c	0.6
Map9	F60	F59	Increased i	Increased g	0.6
Map9	F158	F49	Youth/child	Increase in	0.6
Map9	F158	F01	Youth/child	Bad experi	0.6
Map9	F158	F69	Youth/child	Increased t	0.6
Map9	F158	F67	Youth/child	Increased s	0.6
Map9	F158	F65	Youth/child	Increased r	0.6
Map9	F158	F60	Youth/child	Increased i	0.6
Map9	F158	F130	Youth/child	Physical in	0.4
Map9	F158	F99	Youth/child	More unwa	0.2
Map9	F158	F58	Youth/child	Increased c	0.6
Map9	F158	F59	Youth/child	Increased g	0.6
Map9	F158	F133	Youth/child	Poor perfor	0.6
Map9	F158	F13	Youth/child	Could not c	0.6
Map9	F158	F16	Youth/child	Delay in ec	0.6
Map9	F85	F40	Markets/sh	Household	0.4
Map9	F85	F88	Markets/sh	Men unable	0.6

For Peer Review

Map9	F85	F47	Markets/sh	Increase in	0.2
Map9	F85	F87	Markets/sh	Men stayin	0.8
Map9	F85	F82	Markets/sh	Lost invest	0.6
Map9	F85	F07	Markets/sh	Businesses	0.6
Map9	F85	F01	Markets/sh	Bad experi	0.6
Map9	F85	F64	Markets/sh	Increased	0.6
Map9	F85	F96	Markets/sh	More hung	0.6
Map9	F85	F78	Markets/sh	Less food t	0.6
Map9	F96	F01	More hung	Bad experi	0.8
Map9	F118	F01	No social g	Bad experi	0.4
Map9	F118	F32	No social g	Feeling soc	0.6
Map9	F118	F147	No social g	Stress and	0.4
Map9	F130	F01	Physical in	Bad experi	0.2
Map9	F99	F01	More unwa	Bad experi	0.2
Map9	F124	F01	Not visiting	Bad experi	0.8
Map9	F124	F63	Not visiting	Increased	0.8
Map9	F124	F08	Not visiting	Cannot cor	0.8
Map9	F124	F98	Not visiting	More mate	0.4
Map9	F124	F14	Not visiting	Could not	0.8
Map9	F58	F01	Increased	Bad experi	0.8
Map9	F58	F69	Increased	Increased t	0.6
Map9	F58	F67	Increased	Increased	0.8
Map9	F59	F01	Increased	Bad experi	0.6
Map9	F59	F67	Increased	Increased	0.8
Map9	F32	F01	Feeling soc	Bad experi	0.4
Map9	F32	F147	Feeling soc	Stress and	0.4
Map9	F78	F01	Less food t	Bad experi	0.8
Map9	F78	F96	Less food t	More hung	0.8
Map9	F133	F01	Poor perfor	Bad experi	0.4
Map9	F74	F01	Lack of fac	Bad experi	0.4
Map9	F74	F125	Lack of fac	Not wearin	0.6
Map9	F74	F127	Lack of fac	Penalty for	0.6
Map9	F13	F49	Could not	Increase in	0.6
Map9	F13	F01	Could not	Bad experi	0.6
Map9	F13	F69	Could not	Increased t	0.6
Map9	F13	F67	Could not	Increased	0.6
Map9	F13	F65	Could not	Increased	0.6
Map9	F13	F60	Could not	Increased i	0.8
Map9	F13	F130	Could not	Physical in	0.4
Map9	F13	F99	Could not	More unwa	0.2
Map9	F13	F58	Could not	Increased	0.6
Map9	F13	F59	Could not	Increased	0.6
Map9	F10	F20	Compulsor	Difficult bre	0.4
Map9	F10	F01	Compulsor	Bad experi	0.4
Map9	F10	F74	Compulsor	Lack of fac	0.8
Map9	F10	F125	Compulsor	Not wearin	0.6
Map9	F10	F127	Compulsor	Penalty for	0.6
Map9	F16	F01	Delay in ec	Bad experi	0.4
Map9	F16	F133	Delay in ec	Poor perfor	0.6
Map9	F98	F01	More mate	Bad experi	0.4
Map9	F14	F01	Could not	Bad experi	0.8
Map9	F14	F63	Could not	Increased	0.8
Map9	F125	F01	Not wearin	Bad experi	0.4
Map9	F125	F127	Not wearin	Penalty for	0.6
Map9	F142	F49	School/ma	Increase in	0.6
Map9	F142	F01	School/ma	Bad experi	0.6

For Peer Review

Map9	F142	F69	School/ma	Increased t	0.6
Map9	F142	F67	School/ma	Increased s	0.6
Map9	F142	F65	School/ma	Increased r	0.6
Map9	F142	F60	School/ma	Increased i	0.6
Map9	F142	F158	School/ma	Youth/child	0.8
Map9	F142	F130	School/ma	Physical in	0.4
Map9	F142	F99	School/ma	More unwa	0.2
Map9	F142	F58	School/ma	Increased (	0.6
Map9	F142	F59	School/ma	Increased g	0.6
Map9	F142	F133	School/ma	Poor perfo	0.6
Map9	F142	F13	School/ma	Could not c	0.6
Map9	F142	F16	School/ma	Delay in ec	0.6
Map9	F127	F01	Penalty for	Bad experi	0.4
Map9	F147	F01	Stress and	Bad experi	0.6
Map9	F120	F01	No transpo	Bad experi	0.8
Map9	F120	F63	No transpo	Increased (	0.8
Map9	F120	F08	No transpo	Cannot cor	0.8
Map9	F120	F124	No transpo	Not visiting	0.8
Map9	F120	F98	No transpo	More mate	0.4
Map9	F120	F14	No transpo	Could not g	0.8
Map9	F144	F01	Social dista	Bad experi	0.4
Map9	F144	F118	Social dista	No social g	0.8
Map9	F144	F32	Social dista	Feeling soc	0.6
Map9	F144	F147	Social dista	Stress and	0.4
Map9	F115	F01	No preachi	Bad experi	0.2
Map9	F115	F147	No preachi	Stress and	0.2
Map9	F106	F01	No congreg	Bad experi	0.2
Map9	F106	F147	No congreg	Stress and	0.2
Map9	F106	F115	No congreg	No preachi	0.6
Map9	F100	F01	Mosques/c	Bad experi	0.2
Map9	F100	F147	Mosques/c	Stress and	0.2
Map9	F100	F115	Mosques/c	No preachi	0.6
Map9	F100	F106	Mosques/c	No congreg	0.8
Map10	F89	F01	Misconcep	Bad experi	0.8
Map10	F89	F63	Misconcep	Increased (	0.8
Map10	F89	F124	Misconcep	Not visiting	0.8
Map10	F85	F01	Markets/sh	Bad experi	0.6
Map10	F85	F64	Markets/sh	Increased p	0.6
Map10	F85	F96	Markets/sh	More hung	0.6
Map10	F85	F78	Markets/sh	Less food t	0.6
Map10	F85	F104	Markets/sh	No buyers	0.8
Map10	F118	F01	No social g	Bad experi	0.6
Map10	F118	F147	No social g	Stress and	0.6
Map10	F118	F106	No social g	No congreg	0.6
Map10	F80	F85	Lockdown	Markets/sh	1
Map10	F80	F118	Lockdown	No social g	0.2
Map10	F80	F82	Lockdown	Lost invest	0.6
Map10	F80	F01	Lockdown	Bad experi	0.6
Map10	F80	F64	Lockdown	Increased p	0.6
Map10	F80	F63	Lockdown	Increased (	0.6
Map10	F80	F16	Lockdown	Delay in ec	0.4
Map10	F80	F96	Lockdown	More hung	0.6
Map10	F80	F133	Lockdown	Poor perfo	0.8
Map10	F80	F51	Lockdown	Increase in	0.4
Map10	F80	F33	Lockdown	Filling stati	0.6
Map10	F80	F78	Lockdown	Less food t	0.6

For Peer Review

Map10	F80	F70	Lockdown	Jobs lost	0.6
Map10	F80	F142	Lockdown	School/ma	0.8
Map10	F80	F140	Lockdown	Restriction	0.6
Map10	F80	F147	Lockdown	Stress and	0.6
Map10	F80	F123	Lockdown	Not going t	0.6
Map10	F80	F144	Lockdown	Social dista	0.8
Map10	F80	F106	Lockdown	No congreg	0.6
Map10	F80	F104	Lockdown	No buyers	0.8
Map10	F80	F103	Lockdown	No access	0.6
Map10	F80	F100	Lockdown	Mosques/c	0.8
Map10	F82	F01	Lost invest	Bad experi	0.6
Map10	F82	F64	Lost invest	Increased p	0.6
Map10	F82	F96	Lost invest	More hung	0.6
Map10	F82	F78	Lost invest	Less food t	0.6
Map10	F20	F01	Difficult bre	Bad experi	1
Map10	F20	F63	Difficult bre	Increased c	0.8
Map10	F29	F20	Fear of cor	Difficult bre	0.6
Map10	F29	F01	Fear of cor	Bad experi	0.6
Map10	F29	F63	Fear of cor	Increased c	0.6
Map10	F29	F154	Fear of cor	Wearing fa	0.6
Map10	F29	F124	Fear of cor	Not visiting	0.6
Map10	F64	F01	Increased p	Bad experi	1
Map10	F64	F96	Increased p	More hung	0.6
Map10	F64	F78	Increased p	Less food t	0.6
Map10	F63	F01	Increased c	Bad experi	0.8
Map10	F154	F20	Wearing fa	Difficult bre	0.8
Map10	F154	F01	Wearing fa	Bad experi	0.8
Map10	F154	F63	Wearing fa	Increased c	0.8
Map10	F16	F01	Delay in ec	Bad experi	0.6
Map10	F16	F51	Delay in ec	Increase in	0.6
Map10	F96	F01	More hung	Bad experi	0.8
Map10	F133	F01	Poor perfor	Bad experi	0.4
Map10	F133	F16	Poor perfor	Delay in ec	0.4
Map10	F133	F51	Poor perfor	Increase in	0.4
Map10	F111	F01	No governr	Bad experi	0.8
Map10	F111	F96	No governr	More hung	0.8
Map10	F111	F78	No governr	Less food t	0.8
Map10	F51	F01	Increase in	Bad experi	0.8
Map10	F124	F01	Not visiting	Bad experi	0.8
Map10	F124	F63	Not visiting	Increased c	0.8
Map10	F33	F01	Filling stati	Bad experi	0.6
Map10	F33	F63	Filling stati	Increased c	0.6
Map10	F33	F103	Filling stati	No access	0.6
Map10	F78	F01	Less food t	Bad experi	0.8
Map10	F78	F96	Less food t	More hung	0.8
Map10	F70	F01	Jobs lost	Bad experi	0.8
Map10	F70	F64	Jobs lost	Increased p	0.8
Map10	F70	F96	Jobs lost	More hung	0.6
Map10	F70	F78	Jobs lost	Less food t	0.6
Map10	F142	F01	School/ma	Bad experi	0.4
Map10	F142	F16	School/ma	Delay in ec	0.4
Map10	F142	F133	School/ma	Poor perfor	0.8
Map10	F142	F51	School/ma	Increase in	0.4
Map10	F140	F82	Restriction	Lost invest	0.6
Map10	F140	F01	Restriction	Bad experi	0.6
Map10	F140	F64	Restriction	Increased p	0.6

For Peer Review

Map10	F140	F96	Restriction	More hung	0.6
Map10	F140	F78	Restriction	Less food t	0.6
Map10	F140	F70	Restriction	Jobs lost	0.6
Map10	F140	F123	Restriction	Not going t	0.8
Map10	F147	F01	Stress and	Bad experi	0.8
Map10	F123	F82	Not going t	Lost invest	0.6
Map10	F123	F01	Not going t	Bad experi	0.6
Map10	F123	F64	Not going t	Increased	0.6
Map10	F123	F96	Not going t	More hung	0.6
Map10	F123	F78	Not going t	Less food t	0.6
Map10	F144	F118	Social dista	No social g	0.2
Map10	F144	F01	Social dista	Bad experi	0.2
Map10	F144	F147	Social dista	Stress and	0.2
Map10	F144	F106	Social dista	No congreg	0.2
Map10	F106	F01	No congreg	Bad experi	1
Map10	F106	F147	No congreg	Stress and	0.8
Map10	F104	F01	No buyers	Bad experi	0.6
Map10	F104	F64	No buyers	Increased	0.6
Map10	F104	F96	No buyers	More hung	0.6
Map10	F104	F78	No buyers	Less food t	0.6
Map10	F103	F01	No access	Bad experi	0.8
Map10	F103	F63	No access	Increased	0.8
Map10	F103	F103	No access	No access	0.8
Map10	F100	F01	Mosques/c	Bad experi	0.6
Map10	F100	F147	Mosques/c	Stress and	0.6
Map10	F100	F106	Mosques/c	No congreg	0.6
Map11	F40	F47	Household	Increase in	0.4
Map11	F40	F01	Household	Bad experi	0.4
Map11	F88	F40	Men unable	Household	0.6
Map11	F88	F47	Men unable	Increase in	0.4
Map11	F88	F01	Men unable	Bad experi	0.4
Map11	F47	F01	Increase in	Bad experi	0.8
Map11	F85	F82	Markets/sh	Lost invest	0.6
Map11	F85	F07	Markets/sh	Businesses	0.6
Map11	F85	F01	Markets/sh	Bad experi	0.6
Map11	F85	F64	Markets/sh	Increased	0.6
Map11	F85	F96	Markets/sh	More hung	0.6
Map11	F85	F78	Markets/sh	Less food t	0.6
Map11	F87	F40	Men stayin	Household	0.6
Map11	F87	F88	Men stayin	Men unable	0.6
Map11	F87	F47	Men stayin	Increase in	0.4
Map11	F87	F01	Men stayin	Bad experi	0.6
Map11	F87	F64	Men stayin	Increased	0.6
Map11	F87	F96	Men stayin	More hung	0.6
Map11	F87	F78	Men stayin	Less food t	0.6
Map11	F87	F70	Men stayin	Jobs lost	0.6
Map11	F81	F01	Loss of Ha	Bad experi	0.8
Map11	F81	F64	Loss of Ha	Increased	0.6
Map11	F81	F96	Loss of Ha	More hung	0.6
Map11	F81	F78	Loss of Ha	Less food t	0.6
Map11	F81	F147	Loss of Ha	Stress and	0.8
Map11	F80	F40	Lockdown	Household	0.6
Map11	F80	F88	Lockdown	Men unable	0.6
Map11	F80	F47	Lockdown	Increase in	0.4
Map11	F80	F85	Lockdown	Markets/sh	0.8
Map11	F80	F87	Lockdown	Men stayin	0.8

For Peer Review

Map11	F80	F82	Lockdown	Lost invest	0.6
Map11	F80	F07	Lockdown	Businesses	0.6
Map11	F80	F01	Lockdown	Bad experi	0.8
Map11	F80	F64	Lockdown	Increased p	0.6
Map11	F80	F60	Lockdown	Increased i	0.6
Map11	F80	F158	Lockdown	Youth/child	0.8
Map11	F80	F16	Lockdown	Delay in ec	0.8
Map11	F80	F96	Lockdown	More hung	0.6
Map11	F80	F78	Lockdown	Less food t	0.6
Map11	F80	F115	Lockdown	No preachi	0.6
Map11	F80	F13	Lockdown	Could not c	0.6
Map11	F80	F70	Lockdown	Jobs lost	0.6
Map11	F80	F142	Lockdown	School/ma	1
Map11	F80	F147	Lockdown	Stress and	0.8
Map11	F80	F106	Lockdown	No congreg	0.6
Map11	F80	F100	Lockdown	Mosques/c	1
Map11	F82	F01	Lost invest	Bad experi	0.8
Map11	F82	F64	Lost invest	Increased p	0.8
Map11	F82	F96	Lost invest	More hung	0.8
Map11	F82	F78	Lost invest	Less food t	0.8
Map11	F07	F82	Businesses	Lost invest	1
Map11	F07	F01	Businesses	Bad experi	0.8
Map11	F07	F64	Businesses	Increased p	0.8
Map11	F07	F96	Businesses	More hung	0.8
Map11	F07	F78	Businesses	Less food t	0.8
Map11	F06	F81	Borders clo	Loss of Ha	0.6
Map11	F06	F01	Borders clo	Bad experi	0.6
Map11	F06	F64	Borders clo	Increased p	0.6
Map11	F06	F96	Borders clo	More hung	0.6
Map11	F06	F114	Borders clo	No pilgrima	1
Map11	F06	F78	Borders clo	Less food t	0.6
Map11	F06	F147	Borders clo	Stress and	0.6
Map11	F29	F01	Fear of cor	Bad experi	0.6
Map11	F29	F64	Fear of cor	Increased p	0.2
Map11	F29	F63	Fear of cor	Increased c	0.6
Map11	F29	F96	Fear of cor	More hung	0.2
Map11	F29	F31	Fear of cor	Fear of eac	0.4
Map11	F29	F78	Fear of cor	Less food t	0.2
Map11	F29	F14	Fear of cor	Could not g	0.6
Map11	F29	F147	Fear of cor	Stress and	0.2
Map11	F29	F105	Fear of cor	No condole	0.4
Map11	F64	F01	Increased p	Bad experi	0.8
Map11	F64	F96	Increased p	More hung	0.8
Map11	F64	F78	Increased p	Less food t	1
Map11	F63	F01	Increased c	Bad experi	1
Map11	F60	F01	Increased i	Bad experi	0.8
Map11	F158	F01	Youth/child	Bad experi	0.6
Map11	F158	F60	Youth/child	Increased i	0.6
Map11	F16	F01	Delay in ec	Bad experi	0.8
Map11	F16	F64	Delay in ec	Increased p	0.6
Map11	F16	F96	Delay in ec	More hung	0.6
Map11	F16	F78	Delay in ec	Less food t	0.6
Map11	F16	F147	Delay in ec	Stress and	1
Map11	F96	F01	More hung	Bad experi	1
Map11	F114	F81	No pilgrima	Loss of Ha	0.6
Map11	F114	F01	No pilgrima	Bad experi	0.6

For Peer Review

Map11	F114	F64	No pilgrima	Increased	0.6
Map11	F114	F96	No pilgrima	More hung	0.6
Map11	F114	F78	No pilgrima	Less food t	0.6
Map11	F114	F147	No pilgrima	Stress and	0.6
Map11	F118	F85	No social g	Markets/sh	0.8
Map11	F118	F82	No social g	Lost invest	0.6
Map11	F118	F07	No social g	Businesses	0.6
Map11	F118	F01	No social g	Bad experi	0.6
Map11	F118	F64	No social g	Increased	0.6
Map11	F118	F96	No social g	More hung	0.6
Map11	F118	F118	No social g	No social g	0.6
Map11	F118	F78	No social g	Less food t	0.6
Map11	F118	F147	No social g	Stress and	0.4
Map11	F118	F121	No social g	No weddin	0.4
Map11	F112	F82	No home/p	Lost invest	0.6
Map11	F112	F01	No home/p	Bad experi	0.6
Map11	F112	F64	No home/p	Increased	0.6
Map11	F112	F96	No home/p	More hung	0.6
Map11	F112	F78	No home/p	Less food t	0.6
Map11	F31	F01	Fear of eac	Bad experi	0.8
Map11	F78	F01	Less food t	Bad experi	0.8
Map11	F78	F96	Less food t	More hung	0.8
Map11	F115	F01	No preachi	Bad experi	0.6
Map11	F115	F64	No preachi	Increased	0.6
Map11	F115	F96	No preachi	More hung	0.6
Map11	F115	F78	No preachi	Less food t	0.6
Map11	F115	F147	No preachi	Stress and	0.6
Map11	F13	F01	Could not c	Bad experi	0.8
Map11	F70	F01	Jobs lost	Bad experi	0.8
Map11	F70	F64	Jobs lost	Increased	0.8
Map11	F70	F96	Jobs lost	More hung	0.8
Map11	F70	F78	Jobs lost	Less food t	0.8
Map11	F14	F01	Could not g	Bad experi	0.8
Map11	F14	F63	Could not g	Increased c	0.8
Map11	F14	F14	Could not g	Could not g	0.6
Map11	F142	F01	School/ma	Bad experi	0.8
Map11	F142	F64	School/ma	Increased	0.6
Map11	F142	F60	School/ma	Increased i	0.6
Map11	F142	F158	School/ma	Youth/child	0.8
Map11	F142	F16	School/ma	Delay in ec	0.8
Map11	F142	F96	School/ma	More hung	0.6
Map11	F142	F78	School/ma	Less food t	0.6
Map11	F142	F13	School/ma	Could not c	0.6
Map11	F142	F147	School/ma	Stress and	0.8
Map11	F140	F82	Restriction	Lost invest	0.4
Map11	F140	F01	Restriction	Bad experi	0.4
Map11	F140	F64	Restriction	Increased	0.4
Map11	F140	F96	Restriction	More hung	0.4
Map11	F140	F112	Restriction	No home/p	0.4
Map11	F140	F78	Restriction	Less food t	0.4
Map11	F140	F147	Restriction	Stress and	0.4
Map11	F140	F104	Restriction	No buyers	0.6
Map11	F147	F01	Stress and	Bad experi	0.8
Map11	F147	F64	Stress and	Increased	0.6
Map11	F147	F96	Stress and	More hung	0.6
Map11	F147	F78	Stress and	Less food t	0.6

For Peer Review

Map11	F121	F01	No weddin	Bad experi	0.8
Map11	F121	F64	No weddin	Increased p	0.6
Map11	F121	F96	No weddin	More hung	0.6
Map11	F121	F78	No weddin	Less food t	0.6
Map11	F121	F147	No weddin	Stress and	0.8
Map11	F106	F01	No congreg	Bad experi	0.8
Map11	F106	F64	No congreg	Increased p	0.6
Map11	F106	F96	No congreg	More hung	0.6
Map11	F106	F78	No congreg	Less food t	0.6
Map11	F106	F147	No congreg	Stress and	1
Map11	F105	F01	No condole	Bad experi	0.2
Map11	F105	F64	No condole	Increased p	0.2
Map11	F105	F96	No condole	More hung	0.2
Map11	F105	F78	No condole	Less food t	0.2
Map11	F105	F147	No condole	Stress and	0.2
Map11	F104	F82	No buyers	Lost invest	0.4
Map11	F104	F01	No buyers	Bad experi	0.4
Map11	F104	F64	No buyers	Increased p	0.4
Map11	F104	F96	No buyers	More hung	0.4
Map11	F104	F112	No buyers	No home/p	0.4
Map11	F104	F78	No buyers	Less food t	0.4
Map11	F100	F01	Mosques/c	Bad experi	0.6
Map11	F100	F64	Mosques/c	Increased p	0.6
Map11	F100	F96	Mosques/c	More hung	0.6
Map11	F100	F78	Mosques/c	Less food t	0.6
Map11	F100	F115	Mosques/c	No preachi	0.6
Map11	F100	F147	Mosques/c	Stress and	0.6
Map11	F100	F106	Mosques/c	No congreg	0.6
Map12	F40	F47	Household	Increase in	0.8
Map12	F40	F01	Household	Bad experi	0.8
Map12	F88	F40	Men unabl	Household	0.8
Map12	F88	F47	Men unabl	Increase in	0.8
Map12	F88	F01	Men unabl	Bad experi	0.8
Map12	F88	F96	Men unabl	More hung	0.6
Map12	F88	F78	Men unabl	Less food t	0.6
Map12	F47	F01	Increase in	Bad experi	1
Map12	F85	F82	Markets/sh	Lost invest	0.8
Map12	F85	F01	Markets/sh	Bad experi	0.8
Map12	F85	F64	Markets/sh	Increased p	0.8
Map12	F85	F147	Markets/sh	Stress and	0.6
Map12	F85	F121	Markets/sh	No weddin	0.6
Map12	F80	F40	Lockdown	Household	0.8
Map12	F80	F88	Lockdown	Men unabl	0.8
Map12	F80	F47	Lockdown	Increase in	0.8
Map12	F80	F85	Lockdown	Markets/sh	0.8
Map12	F80	F82	Lockdown	Lost invest	0.8
Map12	F80	F07	Lockdown	Businesses	1
Map12	F80	F01	Lockdown	Bad experi	0.8
Map12	F80	F67	Lockdown	Increased s	0.4
Map12	F80	F64	Lockdown	Increased p	0.8
Map12	F80	F60	Lockdown	Increased i	0.6
Map12	F80	F158	Lockdown	Youth/child	0.6
Map12	F80	F96	Lockdown	More hung	0.6
Map12	F80	F94	Lockdown	More hous	0.7
Map12	F80	F51	Lockdown	Increase in	0.7
Map12	F80	F78	Lockdown	Less food t	0.6

For Peer Review

Map12	F80	F147	Lockdown	Stress and	0.8
Map12	F80	F70	Lockdown	Jobs lost	0.8
Map12	F80	F142	Lockdown	School/ma	0.7
Map12	F80	F140	Lockdown	Restriction	1
Map12	F80	F121	Lockdown	No weddin	0.6
Map12	F80	F120	Lockdown	No transpo	0.8
Map12	F80	F123	Lockdown	Not going t	0.6
Map12	F80	F106	Lockdown	No congre	1
Map12	F80	F100	Lockdown	Mosques/c	1
Map12	F82	F01	Lost invest	Bad experi	0.8
Map12	F82	F64	Lost invest	Increased	1
Map12	F82	F147	Lost invest	Stress and	0.6
Map12	F82	F121	Lost invest	No weddin	0.6
Map12	F07	F40	Businesses	Household	0.8
Map12	F07	F88	Businesses	Men unable	0.8
Map12	F07	F47	Businesses	Increase in	0.8
Map12	F07	F01	Businesses	Bad experi	0.8
Map12	F07	F96	Businesses	More hung	0.6
Map12	F07	F78	Businesses	Less food t	0.6
Map12	F07	F70	Businesses	Jobs lost	0.8
Map12	F20	F01	Difficult bre	Bad experi	0.4
Map12	F67	F01	Increased	Bad experi	1
Map12	F64	F01	Increased	Bad experi	0.8
Map12	F63	F01	Increased	Bad experi	1
Map12	F60	F01	Increased i	Bad experi	0.4
Map12	F60	F67	Increased i	Increased	0.4
Map12	F158	F01	Youth/child	Bad experi	0.8
Map12	F158	F67	Youth/child	Increased	0.4
Map12	F158	F60	Youth/child	Increased i	0.6
Map12	F158	F94	Youth/child	More hous	1
Map12	F158	F147	Youth/child	Stress and	0.8
Map12	F96	F01	More hung	Bad experi	0.8
Map12	F94	F01	More hous	Bad experi	0.8
Map12	F94	F147	More hous	Stress and	0.8
Map12	F111	F01	No governn	Bad experi	0.8
Map12	F111	F67	No governn	Increased	0.4
Map12	F111	F60	No governn	Increased i	0.6
Map12	F111	F158	No governn	Youth/child	0.6
Map12	F111	F94	No governn	More hous	0.8
Map12	F111	F51	No governn	Increase in	0.8
Map12	F111	F147	No governn	Stress and	0.8
Map12	F111	F142	No governn	School/ma	0.8
Map12	F51	F01	Increase in	Bad experi	0.6
Map12	F51	F67	Increase in	Increased	0.4
Map12	F51	F60	Increase in	Increased i	0.6
Map12	F51	F158	Increase in	Youth/child	0.6
Map12	F51	F94	Increase in	More hous	0.6
Map12	F51	F147	Increase in	Stress and	0.6
Map12	F78	F01	Less food t	Bad experi	0.8
Map12	F78	F96	Less food t	More hung	0.8
Map12	F37	F01	Health facil	Bad experi	0.8
Map12	F37	F63	Health facil	Increased	0.8
Map12	F37	F38	Health facil	Health wor	0.8
Map12	F38	F01	Health wor	Bad experi	0.8
Map12	F38	F63	Health wor	Increased	0.8
Map12	F147	F01	Stress and	Bad experi	1

For Peer Review

Map12	F10	F20	Compulsor	Difficult bre	0.2
Map12	F10	F01	Compulsor	Bad experi	0.2
Map12	F70	F40	Jobs lost	Household	0.8
Map12	F70	F88	Jobs lost	Men unabl	0.8
Map12	F70	F47	Jobs lost	Increase in	0.8
Map12	F70	F01	Jobs lost	Bad experi	0.8
Map12	F70	F96	Jobs lost	More hung	0.6
Map12	F70	F78	Jobs lost	Less food t	0.6
Map12	F142	F01	School/ma	Bad experi	0.8
Map12	F142	F67	School/ma	Increased s	0.4
Map12	F142	F60	School/ma	Increased i	0.6
Map12	F142	F158	School/ma	Youth/child	0.6
Map12	F142	F94	School/ma	More hous	1
Map12	F142	F51	School/ma	Increase in	1
Map12	F142	F147	School/ma	Stress and	0.8
Map12	F140	F40	Restriction	Household	0.8
Map12	F140	F88	Restriction	Men unabl	0.8
Map12	F140	F47	Restriction	Increase in	0.8
Map12	F140	F85	Restriction	Markets/sh	0.8
Map12	F140	F82	Restriction	Lost invest	0.8
Map12	F140	F07	Restriction	Businesses	1
Map12	F140	F01	Restriction	Bad experi	0.8
Map12	F140	F64	Restriction	Increased p	0.8
Map12	F140	F96	Restriction	More hung	0.6
Map12	F140	F78	Restriction	Less food t	0.6
Map12	F140	F147	Restriction	Stress and	0.6
Map12	F140	F70	Restriction	Jobs lost	0.8
Map12	F140	F121	Restriction	No weddin	0.6
Map12	F140	F120	Restriction	No transpo	0.8
Map12	F140	F123	Restriction	Not going t	0.6
Map12	F121	F01	No weddin	Bad experi	0.8
Map12	F121	F147	No weddin	Stress and	0.8
Map12	F120	F40	No transpo	Household	0.4
Map12	F120	F88	No transpo	Men unabl	0.4
Map12	F120	F47	No transpo	Increase in	0.4
Map12	F120	F01	No transpo	Bad experi	0.6
Map12	F120	F96	No transpo	More hung	0.6
Map12	F120	F78	No transpo	Less food t	0.6
Map12	F120	F123	No transpo	Not going t	0.6
Map12	F123	F40	Not going t	Household	0.4
Map12	F123	F88	Not going t	Men unabl	0.4
Map12	F123	F47	Not going t	Increase in	0.4
Map12	F123	F01	Not going t	Bad experi	0.6
Map12	F123	F96	Not going t	More hung	0.6
Map12	F123	F78	Not going t	Less food t	0.6
Map12	F106	F01	No congreg	Bad experi	0.8
Map12	F106	F147	No congreg	Stress and	0.8
Map12	F100	F01	Mosques/c	Bad experi	0.8
Map12	F100	F147	Mosques/c	Stress and	0.8
Map12	F100	F106	Mosques/c	No congreg	1
Map13	F40	F47	Household	Increase in	0.6
Map13	F40	F01	Household	Bad experi	0.4
Map13	F88	F40	Men unabl	Household	0.6
Map13	F88	F47	Men unabl	Increase in	0.6
Map13	F88	F01	Men unabl	Bad experi	0.6
Map13	F88	F96	Men unabl	More hung	0.8

For Peer Review

Map13	F47	F01	Increase in	Bad experi	0.4
Map13	F85	F40	Markets/sh	Household	0.6
Map13	F85	F88	Markets/sh	Men unable	0.8
Map13	F85	F47	Markets/sh	Increase in	0.6
Map13	F85	F87	Markets/sh	Men stayin	1
Map13	F85	F01	Markets/sh	Bad experi	0.6
Map13	F85	F64	Markets/sh	Increased p	0.8
Map13	F85	F96	Markets/sh	More hung	0.8
Map13	F85	F98	Markets/sh	More mate	0.4
Map13	F85	F99	Markets/sh	More unwa	0.6
Map13	F85	F135	Markets/sh	Price hike	1
Map13	F85	F70	Markets/sh	Jobs lost	1
Map13	F87	F01	Men stayin	Bad experi	0.4
Map13	F87	F98	Men stayin	More mate	0.4
Map13	F87	F99	Men stayin	More unwa	0.6
Map13	F81	F01	Loss of Ha	Bad experi	0.4
Map13	F81	F147	Loss of Ha	Stress and	0.4
Map13	F80	F40	Lockdown	Household	0.6
Map13	F80	F88	Lockdown	Men unable	0.8
Map13	F80	F47	Lockdown	Increase in	0.6
Map13	F80	F85	Lockdown	Markets/sh	1
Map13	F80	F87	Lockdown	Men stayin	1
Map13	F80	F81	Lockdown	Loss of Ha	0.6
Map13	F80	F82	Lockdown	Lost invest	0.6
Map13	F80	F07	Lockdown	Businesses	0.8
Map13	F80	F01	Lockdown	Bad experi	0.8
Map13	F80	F64	Lockdown	Increased p	0.8
Map13	F80	F158	Lockdown	Youth/child	1
Map13	F80	F16	Lockdown	Delay in ec	0.6
Map13	F80	F114	Lockdown	No pilgrima	1
Map13	F80	F96	Lockdown	More hung	0.8
Map13	F80	F98	Lockdown	More mate	0.4
Map13	F80	F99	Lockdown	More unwa	0.6
Map13	F80	F135	Lockdown	Price hike	1
Map13	F80	F78	Lockdown	Less food t	0.6
Map13	F80	F13	Lockdown	Could not c	0.4
Map13	F80	F70	Lockdown	Jobs lost	1
Map13	F80	F142	Lockdown	School/ma	1
Map13	F80	F147	Lockdown	Stress and	0.8
Map13	F80	F120	Lockdown	No transpo	1
Map13	F80	F06	Lockdown	Borders clo	1
Map13	F80	F106	Lockdown	No congreg	0.8
Map13	F80	F100	Lockdown	Mosques/c	1
Map13	F82	F01	Lost invest	Bad experi	0.6
Map13	F82	F96	Lost invest	More hung	0.4
Map13	F82	F78	Lost invest	Less food t	0.6
Map13	F07	F82	Businesses	Lost invest	0.6
Map13	F07	F01	Businesses	Bad experi	0.6
Map13	F07	F96	Businesses	More hung	0.4
Map13	F07	F78	Businesses	Less food t	0.6
Map13	F20	F01	Difficult bre	Bad experi	0.4
Map13	F29	F01	Fear of cor	Bad experi	0.4
Map13	F29	F31	Fear of cor	Fear of eac	0.6
Map13	F64	F40	Increased p	Household	0.6
Map13	F64	F88	Increased p	Men unable	1
Map13	F64	F47	Increased p	Increase in	0.6

For Peer Review

Map13	F64	F01	Increased ¡	Bad experi	0.6
Map13	F64	F96	Increased ¡	More hung	0.8
Map13	F63	F01	Increased (	Bad experi	0.4
Map13	F158	F01	Youth/child	Bad experi	0.4
Map13	F158	F16	Youth/child	Delay in ec	0.6
Map13	F158	F13	Youth/child	Could not (	0.4
Map13	F158	F147	Youth/child	Stress and	0.4
Map13	F16	F01	Delay in ec	Bad experi	0.4
Map13	F16	F13	Delay in ec	Could not (	0.4
Map13	F16	F147	Delay in ec	Stress and	0.4
Map13	F118	F01	No social g	Bad experi	0.4
Map13	F118	F32	No social g	Feeling soc	0.4
Map13	F114	F81	No pilgrima	Loss of Ha	0.6
Map13	F114	F01	No pilgrima	Bad experi	0.4
Map13	F114	F147	No pilgrima	Stress and	0.4
Map13	F96	F01	More hung	Bad experi	0.6
Map13	F98	F01	More mate	Bad experi	0.4
Map13	F99	F01	More unwa	Bad experi	0.4
Map13	F99	F98	More unwa	More mate	0.4
Map13	F135	F40	Price hike	Household	0.6
Map13	F135	F88	Price hike	Men unable	0.8
Map13	F135	F47	Price hike	Increase in	0.6
Map13	F135	F01	Price hike	Bad experi	0.6
Map13	F135	F64	Price hike	Increased ¡	0.8
Map13	F135	F96	Price hike	More hung	0.8
Map13	F30	F01	Fear of dea	Bad experi	0.4
Map13	F30	F29	Fear of dea	Fear of cor	0.8
Map13	F30	F31	Fear of dea	Fear of eac	0.6
Map13	F31	F01	Fear of eac	Bad experi	0.4
Map13	F32	F01	Feeling soc	Bad experi	0.4
Map13	F78	F01	Less food t	Bad experi	0.6
Map13	F78	F96	Less food t	More hung	0.4
Map13	F13	F01	Could not (	Bad experi	0.4
Map13	F13	F147	Could not (	Stress and	0.4
Map13	F10	F20	Compulsor	Difficult bre	0.6
Map13	F10	F01	Compulsor	Bad experi	0.4
Map13	F70	F87	Jobs lost	Men stayin	1
Map13	F70	F01	Jobs lost	Bad experi	0.4
Map13	F70	F98	Jobs lost	More mate	0.4
Map13	F70	F99	Jobs lost	More unwa	0.6
Map13	F71	F01	Lack of aw	Bad experi	0.2
Map13	F71	F63	Lack of aw	Increased (	0.2
Map13	F71	F141	Lack of aw	Rumors on	0.2
Map13	F142	F01	School/ma	Bad experi	0.4
Map13	F142	F158	School/ma	Youth/child	1
Map13	F142	F16	School/ma	Delay in ec	0.6
Map13	F142	F13	School/ma	Could not (	0.4
Map13	F142	F147	School/ma	Stress and	0.4
Map13	F141	F01	Rumors on	Bad experi	0.4
Map13	F141	F63	Rumors on	Increased (	0.4
Map13	F147	F01	Stress and	Bad experi	0.8
Map13	F120	F82	No transpo	Lost invest	0.6
Map13	F120	F07	No transpo	Businesses	0.8
Map13	F120	F01	No transpo	Bad experi	0.6
Map13	F120	F96	No transpo	More hung	0.4
Map13	F120	F78	No transpo	Less food t	0.6

For Peer Review

Map13	F144	F01	Social dista	Bad experi	0.4
Map13	F144	F118	Social dista	No social g	1
Map13	F144	F32	Social dista	Feeling soc	0.4
Map13	F06	F81	Borders clc	Loss of Ha	0.6
Map13	F06	F01	Borders clc	Bad experi	0.4
Map13	F06	F114	Borders clc	No pilgrima	1
Map13	F06	F147	Borders clc	Stress and	0.4
Map13	F106	F01	No congreg	Bad experi	0.8
Map13	F106	F147	No congreg	Stress and	0.8
Map13	F100	F01	Mosques/c	Bad experi	0.8
Map13	F100	F147	Mosques/c	Stress and	0.8
Map13	F100	F106	Mosques/c	No congreg	0.8
Map14	F40	F47	Household	Increase in	0.2
Map14	F40	F01	Household	Bad experi	0.2
Map14	F88	F40	Men unable	Household	0.6
Map14	F88	F47	Men unable	Increase in	0.2
Map14	F88	F01	Men unable	Bad experi	0.6
Map14	F47	F01	Increase in	Bad experi	0.4
Map14	F85	F82	Markets/sh	Lost invest	0.8
Map14	F85	F07	Markets/sh	Businesses	0.8
Map14	F85	F01	Markets/sh	Bad experi	0.8
Map14	F85	F63	Markets/sh	Increased	0.6
Map14	F85	F96	Markets/sh	More hung	0.8
Map14	F85	F78	Markets/sh	Less food t	0.8
Map14	F80	F40	Lockdown	Household	0.6
Map14	F80	F88	Lockdown	Men unable	0.6
Map14	F80	F47	Lockdown	Increase in	0.2
Map14	F80	F85	Lockdown	Markets/sh	1
Map14	F80	F82	Lockdown	Lost invest	0.8
Map14	F80	F07	Lockdown	Businesses	0.8
Map14	F80	F01	Lockdown	Bad experi	0.8
Map14	F80	F64	Lockdown	Increased	0.6
Map14	F80	F63	Lockdown	Increased	0.6
Map14	F80	F62	Lockdown	Increased	0.6
Map14	F80	F158	Lockdown	Youth/child	0.8
Map14	F80	F96	Lockdown	More hung	0.8
Map14	F80	F110	Lockdown	No funerals	0.6
Map14	F80	F78	Lockdown	Less food t	0.8
Map14	F80	F13	Lockdown	Could not	0.6
Map14	F80	F142	Lockdown	School/ma	0.8
Map14	F80	F140	Lockdown	Restriction	0.6
Map14	F80	F147	Lockdown	Stress and	0.6
Map14	F80	F120	Lockdown	No transpo	0.8
Map14	F80	F149	Lockdown	Teachers a	0.8
Map14	F80	F106	Lockdown	No congreg	0.8
Map14	F80	F100	Lockdown	Mosques/c	1
Map14	F82	F01	Lost invest	Bad experi	0.8
Map14	F82	F63	Lost invest	Increased	0.6
Map14	F82	F96	Lost invest	More hung	1
Map14	F82	F78	Lost invest	Less food t	1
Map14	F07	F82	Businesses	Lost invest	0.8
Map14	F07	F01	Businesses	Bad experi	0.8
Map14	F07	F63	Businesses	Increased	0.6
Map14	F07	F96	Businesses	More hung	0.8
Map14	F07	F78	Businesses	Less food t	0.8
Map14	F20	F01	Difficult bre	Bad experi	0.4

For Peer Review

Map14	F29	F20	Fear of cor	Difficult bre	0.4
Map14	F29	F01	Fear of cor	Bad experi	0.6
Map14	F29	F30	Fear of cor	Fear of dea	0.8
Map14	F29	F31	Fear of cor	Fear of eac	0.8
Map14	F29	F10	Fear of cor	Compulsor	0.6
Map14	F64	F40	Increased	Household	0.6
Map14	F64	F88	Increased	Men unable	0.6
Map14	F64	F47	Increased	Increase in	0.2
Map14	F64	F01	Increased	Bad experi	0.6
Map14	F63	F01	Increased	Bad experi	0.6
Map14	F62	F01	Increased	Bad experi	0.4
Map14	F158	F01	Youth/child	Bad experi	0.4
Map14	F158	F13	Youth/child	Could not	0.6
Map14	F158	F147	Youth/child	Stress and	0.4
Map14	F96	F01	More hung	Bad experi	0.8
Map14	F96	F63	More hung	Increased	0.6
Map14	F110	F01	No funerals	Bad experi	0.4
Map14	F110	F62	No funerals	Increased	0.6
Map14	F30	F01	Fear of dea	Bad experi	0.6
Map14	F30	F31	Fear of dea	Fear of eac	0.8
Map14	F31	F01	Fear of eac	Bad experi	0.6
Map14	F78	F01	Less food t	Bad experi	0.8
Map14	F78	F63	Less food t	Increased	0.6
Map14	F78	F96	Less food t	More hung	1
Map14	F13	F01	Could not	Bad experi	0.4
Map14	F13	F147	Could not	Stress and	0.4
Map14	F10	F20	Compulsor	Difficult bre	0.4
Map14	F10	F01	Compulsor	Bad experi	0.4
Map14	F142	F01	School/ma	Bad experi	0.4
Map14	F142	F158	School/ma	Youth/child	1
Map14	F142	F13	School/ma	Could not	0.6
Map14	F142	F147	School/ma	Stress and	0.4
Map14	F140	F40	Restriction	Household	0.6
Map14	F140	F88	Restriction	Men unable	0.6
Map14	F140	F47	Restriction	Increase in	0.2
Map14	F140	F01	Restriction	Bad experi	0.6
Map14	F140	F64	Restriction	Increased	0.8
Map14	F147	F01	Stress and	Bad experi	0.6
Map14	F120	F01	No transpo	Bad experi	0.4
Map14	F120	F158	No transpo	Youth/child	0.8
Map14	F120	F13	No transpo	Could not	0.6
Map14	F120	F147	No transpo	Stress and	0.4
Map14	F120	F149	No transpo	Teachers a	0.8
Map14	F149	F01	Teachers a	Bad experi	0.4
Map14	F149	F158	Teachers a	Youth/child	1
Map14	F149	F13	Teachers a	Could not	0.6
Map14	F149	F147	Teachers a	Stress and	0.4
Map14	F106	F01	No congreg	Bad experi	0.6
Map14	F106	F147	No congreg	Stress and	0.6
Map14	F100	F01	Mosques/c	Bad experi	0.6
Map14	F100	F147	Mosques/c	Stress and	0.6
Map14	F100	F106	Mosques/c	No congreg	0.8
Map15	F88	F83	Men unable	Malnutrition	0.6
Map15	F88	F01	Men unable	Bad experi	0.4
Map15	F88	F96	Men unable	More hung	0.6
Map15	F85	F88	Markets/sh	Men unable	0.6

For Peer Review

Map15	F85	F83	Markets/sh	Malnutritior	0.6
Map15	F85	F82	Markets/sh	Lost invest	0.6
Map15	F85	F07	Markets/sh	Businesses	0.8
Map15	F85	F01	Markets/sh	Bad experi	0.6
Map15	F85	F64	Markets/sh	Increased p	0.6
Map15	F85	F96	Markets/sh	More hung	0.6
Map15	F80	F88	Lockdown	Men unable	0.8
Map15	F80	F85	Lockdown	Markets/sh	1
Map15	F80	F83	Lockdown	Malnutritior	0.6
Map15	F80	F82	Lockdown	Lost invest	0.6
Map15	F80	F07	Lockdown	Businesses	0.8
Map15	F80	F01	Lockdown	Bad experi	0.6
Map15	F80	F64	Lockdown	Increased p	0.6
Map15	F80	F63	Lockdown	Increased c	0.6
Map15	F80	F158	Lockdown	Youth/child	0.8
Map15	F80	F96	Lockdown	More hung	0.6
Map15	F80	F133	Lockdown	Poor perfor	0.6
Map15	F80	F32	Lockdown	Feeling soc	0.4
Map15	F80	F118	Lockdown	No social g	0.6
Map15	F80	F37	Lockdown	Health facil	0.8
Map15	F80	F13	Lockdown	Could not c	0.6
Map15	F80	F14	Lockdown	Could not g	0.8
Map15	F80	F142	Lockdown	School/mac	1
Map15	F80	F140	Lockdown	Restriction	0.8
Map15	F80	F147	Lockdown	Stress and	0.4
Map15	F80	F120	Lockdown	No transpo	0.8
Map15	F80	F115	Lockdown	No preachi	0.6
Map15	F80	F106	Lockdown	No congreg	0.8
Map15	F80	F100	Lockdown	Mosques/c	0.8
Map15	F83	F01	Malnutritior	Bad experi	0.4
Map15	F82	F83	Lost invest	Malnutritior	0.6
Map15	F82	F01	Lost invest	Bad experi	0.6
Map15	F82	F64	Lost invest	Increased p	0.8
Map15	F82	F96	Lost invest	More hung	0.8
Map15	F07	F88	Businesses	Men unable	0.6
Map15	F07	F83	Businesses	Malnutritior	0.6
Map15	F07	F82	Businesses	Lost invest	0.6
Map15	F07	F01	Businesses	Bad experi	0.6
Map15	F07	F64	Businesses	Increased p	0.6
Map15	F07	F96	Businesses	More hung	0.6
Map15	F20	F01	Difficult bre	Bad experi	0.2
Map15	F29	F01	Fear of cor	Bad experi	0.4
Map15	F29	F30	Fear of cor	Fear of dea	0.6
Map15	F29	F31	Fear of cor	Fear of eac	0.4
Map15	F64	F83	Increased p	Malnutritior	0.6
Map15	F64	F01	Increased p	Bad experi	0.6
Map15	F64	F96	Increased p	More hung	0.8
Map15	F63	F01	Increased c	Bad experi	0.6
Map15	F158	F01	Youth/child	Bad experi	0.4
Map15	F158	F133	Youth/child	Poor perfor	0.6
Map15	F158	F13	Youth/child	Could not c	0.6
Map15	F158	F147	Youth/child	Stress and	0.4
Map15	F96	F83	More hung	Malnutritior	0.6
Map15	F96	F01	More hung	Bad experi	0.4
Map15	F133	F01	Poor perfor	Bad experi	0.4
Map15	F133	F147	Poor perfor	Stress and	0.4

For Peer Review

Map15	F30	F01	Fear of dea	Bad experi	0.2
Map15	F31	F01	Fear of eac	Bad experi	0.4
Map15	F32	F01	Feeling soc	Bad experi	0.4
Map15	F118	F01	No social g	Bad experi	0.4
Map15	F118	F32	No social g	Feeling soc	0.4
Map15	F37	F01	Health facil	Bad experi	0.6
Map15	F37	F63	Health facil	Increased (	0.6
Map15	F37	F14	Health facil	Could not g	0.8
Map15	F13	F01	Could not c	Bad experi	0.4
Map15	F13	F147	Could not c	Stress and	0.4
Map15	F10	F20	Compulsor	Difficult bre	0.4
Map15	F10	F01	Compulsor	Bad experi	0.2
Map15	F14	F01	Could not g	Bad experi	0.6
Map15	F14	F63	Could not g	Increased (	0.6
Map15	F142	F01	School/mad	Bad experi	0.4
Map15	F142	F158	School/mad	Youth/child	0.8
Map15	F142	F133	School/mad	Poor perfor	0.6
Map15	F142	F13	School/mad	Could not c	0.6
Map15	F142	F147	School/mad	Stress and	0.4
Map15	F140	F88	Restriction	Men unable	0.8
Map15	F140	F83	Restriction	Malnutrition	0.6
Map15	F140	F01	Restriction	Bad experi	0.4
Map15	F140	F96	Restriction	More hung	0.6
Map15	F140	F32	Restriction	Feeling soc	0.4
Map15	F140	F118	Restriction	No social g	0.6
Map15	F140	F120	Restriction	No transpo	0.8
Map15	F147	F01	Stress and	Bad experi	0.6
Map15	F120	F88	No transpo	Men unable	0.8
Map15	F120	F83	No transpo	Malnutrition	0.6
Map15	F120	F01	No transpo	Bad experi	0.4
Map15	F120	F96	No transpo	More hung	0.6
Map15	F115	F01	No preachi	Bad experi	0.4
Map15	F115	F147	No preachi	Stress and	0.4
Map15	F106	F01	No congreg	Bad experi	0.4
Map15	F106	F147	No congreg	Stress and	0.4
Map15	F106	F115	No congreg	No preachi	0.6
Map15	F100	F01	Mosques/c	Bad experi	0.4
Map15	F100	F147	Mosques/c	Stress and	0.4
Map15	F100	F115	Mosques/c	No preachi	0.6
Map15	F100	F106	Mosques/c	No congreg	0.8
Map16	F40	F47	Household	Increase in	0.4
Map16	F40	F01	Household	Bad experi	0.4
Map16	F47	F01	Increase in	Bad experi	0.4
Map16	F85	F82	Markets/sh	Lost invest	0.8
Map16	F85	F07	Markets/sh	Businesses	1
Map16	F85	F01	Markets/sh	Bad experi	0.8
Map16	F85	F64	Markets/sh	Increased p	0.8
Map16	F85	F96	Markets/sh	More hung	0.8
Map16	F87	F40	Men stayin	Household	0.6
Map16	F87	F47	Men stayin	Increase in	0.4
Map16	F87	F01	Men stayin	Bad experi	0.4
Map16	F87	F95	Men stayin	More hous	0.4
Map16	F87	F147	Men stayin	Stress and	0.4
Map16	F81	F01	Loss of Ha	Bad experi	0.4
Map16	F81	F62	Loss of Ha	Increased r	0.6
Map16	F80	F40	Lockdown	Household	0.6

For Peer Review

Map16	F80	F47	Lockdown	Increase in	0.4
Map16	F80	F85	Lockdown	Markets/sh	1
Map16	F80	F87	Lockdown	Men stayin	0.8
Map16	F80	F81	Lockdown	Loss of Ha	0.6
Map16	F80	F82	Lockdown	Lost invest	0.8
Map16	F80	F07	Lockdown	Businesses	1
Map16	F80	F01	Lockdown	Bad experi	0.8
Map16	F80	F64	Lockdown	Increased p	0.8
Map16	F80	F63	Lockdown	Increased c	0.6
Map16	F80	F62	Lockdown	Increased r	0.6
Map16	F80	F157	Lockdown	Women un	0.6
Map16	F80	F158	Lockdown	Youth/child	0.8
Map16	F80	F16	Lockdown	Delay in ec	0.6
Map16	F80	F118	Lockdown	No social g	0.6
Map16	F80	F95	Lockdown	More hous	0.4
Map16	F80	F114	Lockdown	No pilgrima	0.8
Map16	F80	F96	Lockdown	More hung	0.8
Map16	F80	F112	Lockdown	No home/p	0.6
Map16	F80	F32	Lockdown	Feeling soc	0.6
Map16	F80	F37	Lockdown	Health facil	0.6
Map16	F80	F13	Lockdown	Could not c	0.8
Map16	F80	F70	Lockdown	Jobs lost	0.8
Map16	F80	F14	Lockdown	Could not g	0.6
Map16	F80	F142	Lockdown	School/ma	0.8
Map16	F80	F140	Lockdown	Restriction	0.8
Map16	F80	F147	Lockdown	Stress and	0.6
Map16	F80	F120	Lockdown	No transpo	0.6
Map16	F80	F106	Lockdown	No congreg	0.6
Map16	F80	F104	Lockdown	No buyers	0.6
Map16	F80	F100	Lockdown	Mosques/c	0.6
Map16	F82	F01	Lost invest	Bad experi	0.8
Map16	F82	F64	Lost invest	Increased p	0.8
Map16	F82	F96	Lost invest	More hung	0.8
Map16	F07	F82	Businesses	Lost invest	0.8
Map16	F07	F01	Businesses	Bad experi	0.8
Map16	F07	F64	Businesses	Increased p	0.8
Map16	F07	F96	Businesses	More hung	0.8
Map16	F20	F01	Difficult bre	Bad experi	0.2
Map16	F29	F01	Fear of cor	Bad experi	0.4
Map16	F29	F31	Fear of cor	Fear of eac	0.6
Map16	F64	F01	Increased p	Bad experi	1
Map16	F64	F96	Increased p	More hung	0.8
Map16	F63	F01	Increased c	Bad experi	0.4
Map16	F62	F01	Increased r	Bad experi	0.4
Map16	F157	F01	Women un	Bad experi	0.4
Map16	F157	F118	Women un	No social g	0.6
Map16	F157	F32	Women un	Feeling soc	0.6
Map16	F158	F01	Youth/child	Bad experi	0.6
Map16	F158	F16	Youth/child	Delay in ec	0.6
Map16	F158	F13	Youth/child	Could not c	0.8
Map16	F158	F147	Youth/child	Stress and	0.6
Map16	F16	F01	Delay in ec	Bad experi	0.8
Map16	F16	F147	Delay in ec	Stress and	0.8
Map16	F118	F01	No social g	Bad experi	0.4
Map16	F118	F32	No social g	Feeling soc	0.6
Map16	F95	F40	More hous	Household	0.8

For Peer Review

Map16	F95	F47	More hous	Increase in	0.4
Map16	F95	F01	More hous	Bad experi	0.6
Map16	F95	F147	More hous	Stress and	0.6
Map16	F114	F81	No pilgrima	Loss of Ha	0.6
Map16	F114	F01	No pilgrima	Bad experi	0.4
Map16	F114	F62	No pilgrima	Increased r	0.6
Map16	F96	F01	More hung	Bad experi	0.8
Map16	F112	F82	No home/p	Lost invest	0.6
Map16	F112	F01	No home/p	Bad experi	0.6
Map16	F112	F64	No home/p	Increased p	0.6
Map16	F112	F96	No home/p	More hung	0.6
Map16	F31	F01	Fear of eac	Bad experi	0.4
Map16	F32	F01	Feeling soc	Bad experi	0.4
Map16	F37	F01	Health facil	Bad experi	0.4
Map16	F37	F63	Health facil	Increased c	0.6
Map16	F37	F14	Health facil	Could not g	0.8
Map16	F13	F01	Could not c	Bad experi	0.6
Map16	F13	F147	Could not c	Stress and	0.6
Map16	F10	F20	Compulsor	Difficult bre	0.6
Map16	F10	F01	Compulsor	Bad experi	0.2
Map16	F70	F40	Jobs lost	Household	0.6
Map16	F70	F47	Jobs lost	Increase in	0.4
Map16	F70	F87	Jobs lost	Men stayin	0.8
Map16	F70	F01	Jobs lost	Bad experi	0.4
Map16	F70	F95	Jobs lost	More hous	0.4
Map16	F70	F147	Jobs lost	Stress and	0.4
Map16	F14	F01	Could not g	Bad experi	0.4
Map16	F14	F63	Could not g	Increased c	0.6
Map16	F142	F01	School/ma	Bad experi	0.6
Map16	F142	F158	School/ma	Youth/child	0.8
Map16	F142	F16	School/ma	Delay in ec	0.6
Map16	F142	F13	School/ma	Could not c	0.8
Map16	F142	F147	School/ma	Stress and	0.6
Map16	F140	F82	Restriction	Lost invest	0.6
Map16	F140	F01	Restriction	Bad experi	0.6
Map16	F140	F64	Restriction	Increased p	0.6
Map16	F140	F157	Restriction	Women un	0.6
Map16	F140	F118	Restriction	No social g	0.6
Map16	F140	F96	Restriction	More hung	0.6
Map16	F140	F112	Restriction	No home/p	0.6
Map16	F140	F32	Restriction	Feeling soc	0.6
Map16	F140	F120	Restriction	No transpo	0.6
Map16	F140	F104	Restriction	No buyers	0.6
Map16	F147	F01	Stress and	Bad experi	0.8
Map16	F120	F01	No transpo	Bad experi	0.4
Map16	F120	F157	No transpo	Women un	0.6
Map16	F120	F118	No transpo	No social g	0.6
Map16	F120	F32	No transpo	Feeling soc	0.6
Map16	F106	F01	No congreg	Bad experi	0.6
Map16	F104	F82	No buyers	Lost invest	0.6
Map16	F104	F01	No buyers	Bad experi	0.6
Map16	F104	F64	No buyers	Increased p	0.6
Map16	F104	F96	No buyers	More hung	0.6
Map16	F104	F112	No buyers	No home/p	0.6
Map16	F100	F01	Mosques/c	Bad experi	0.6
Map16	F100	F106	Mosques/c	No congreg	0.6

For Peer Review

Map17	F40	F47	Household	Increase in	0.8
Map17	F40	F01	Household	Bad experi	0.8
Map17	F88	F40	Men unabl	Household	0.6
Map17	F88	F47	Men unabl	Increase in	0.6
Map17	F88	F01	Men unabl	Bad experi	0.6
Map17	F47	F01	Increase in	Bad experi	0.6
Map17	F85	F40	Markets/sh	Household	0.6
Map17	F85	F88	Markets/sh	Men unabl	0.6
Map17	F85	F47	Markets/sh	Increase in	0.6
Map17	F85	F87	Markets/sh	Men stayin	0.6
Map17	F85	F82	Markets/sh	Lost invest	0.8
Map17	F85	F07	Markets/sh	Businesses	0.8
Map17	F85	F01	Markets/sh	Bad experi	0.8
Map17	F85	F64	Markets/sh	Increased p	0.8
Map17	F85	F96	Markets/sh	More hung	0.8
Map17	F85	F132	Markets/sh	Poor harve	0.6
Map17	F85	F78	Markets/sh	Less food t	0.8
Map17	F85	F70	Markets/sh	Jobs lost	0.6
Map17	F85	F101	Markets/sh	No access	0.6
Map17	F87	F40	Men stayin	Household	0.6
Map17	F87	F88	Men stayin	Men unabl	0.8
Map17	F87	F47	Men stayin	Increase in	0.6
Map17	F87	F01	Men stayin	Bad experi	0.6
Map17	F80	F40	Lockdown	Household	0.6
Map17	F80	F88	Lockdown	Men unabl	0.6
Map17	F80	F47	Lockdown	Increase in	0.6
Map17	F80	F85	Lockdown	Markets/sh	1
Map17	F80	F87	Lockdown	Men stayin	0.6
Map17	F80	F82	Lockdown	Lost invest	0.8
Map17	F80	F07	Lockdown	Businesses	0.8
Map17	F80	F01	Lockdown	Bad experi	0.8
Map17	F80	F64	Lockdown	Increased p	0.8
Map17	F80	F60	Lockdown	Increased i	0.4
Map17	F80	F158	Lockdown	Youth/child	0.6
Map17	F80	F16	Lockdown	Delay in ec	0.6
Map17	F80	F96	Lockdown	More hung	0.8
Map17	F80	F132	Lockdown	Poor harve	0.6
Map17	F80	F133	Lockdown	Poor perfor	0.4
Map17	F80	F130	Lockdown	Physical in	0.4
Map17	F80	F51	Lockdown	Increase in	0.4
Map17	F80	F78	Lockdown	Less food t	0.8
Map17	F80	F70	Lockdown	Jobs lost	0.6
Map17	F80	F142	Lockdown	School/ma	0.8
Map17	F80	F147	Lockdown	Stress and	0.6
Map17	F80	F120	Lockdown	No transpo	0.6
Map17	F80	F144	Lockdown	Social dista	0.6
Map17	F80	F106	Lockdown	No congreg	0.6
Map17	F80	F101	Lockdown	No access	0.6
Map17	F80	F100	Lockdown	Mosques/c	0.8
Map17	F82	F01	Lost invest	Bad experi	0.8
Map17	F82	F64	Lost invest	Increased p	0.8
Map17	F82	F96	Lost invest	More hung	0.8
Map17	F82	F78	Lost invest	Less food t	0.8
Map17	F07	F40	Businesses	Household	0.6
Map17	F07	F88	Businesses	Men unabl	0.6
Map17	F07	F47	Businesses	Increase in	0.6

For Peer Review

Map17	F07	F87	Businesses	Men stayin	0.6
Map17	F07	F82	Businesses	Lost invest	1
Map17	F07	F01	Businesses	Bad experi	0.8
Map17	F07	F64	Businesses	Increased p	0.8
Map17	F07	F96	Businesses	More hung	0.8
Map17	F07	F78	Businesses	Less food t	0.8
Map17	F07	F70	Businesses	Jobs lost	0.6
Map17	F20	F01	Difficult bre	Bad experi	0.4
Map17	F29	F20	Fear of cor	Difficult bre	0.2
Map17	F29	F01	Fear of cor	Bad experi	0.2
Map17	F29	F31	Fear of cor	Fear of eac	0.8
Map17	F29	F10	Fear of cor	Compulsor	0.4
Map17	F64	F01	Increased p	Bad experi	1
Map17	F63	F01	Increased (	Bad experi	1
Map17	F60	F01	Increased i	Bad experi	0.6
Map17	F60	F130	Increased i	Physical in	0.4
Map17	F60	F147	Increased i	Stress and	0.6
Map17	F158	F01	Youth/child	Bad experi	0.4
Map17	F158	F60	Youth/child	Increased i	0.4
Map17	F158	F16	Youth/child	Delay in ec	0.6
Map17	F158	F133	Youth/child	Poor perfor	0.4
Map17	F158	F130	Youth/child	Physical in	0.4
Map17	F158	F51	Youth/child	Increase in	0.4
Map17	F158	F147	Youth/child	Stress and	0.4
Map17	F16	F01	Delay in ec	Bad experi	0.4
Map17	F16	F60	Delay in ec	Increased i	0.4
Map17	F16	F133	Delay in ec	Poor perfor	0.4
Map17	F16	F130	Delay in ec	Physical in	0.4
Map17	F16	F51	Delay in ec	Increase in	0.4
Map17	F16	F147	Delay in ec	Stress and	0.4
Map17	F96	F01	More hung	Bad experi	1
Map17	F132	F82	Poor harve	Lost invest	0.6
Map17	F132	F01	Poor harve	Bad experi	0.8
Map17	F132	F64	Poor harve	Increased p	0.6
Map17	F132	F96	Poor harve	More hung	0.8
Map17	F132	F78	Poor harve	Less food t	0.8
Map17	F133	F01	Poor perfor	Bad experi	0.6
Map17	F133	F60	Poor perfor	Increased i	0.6
Map17	F133	F130	Poor perfor	Physical in	0.4
Map17	F133	F51	Poor perfor	Increase in	0.6
Map17	F133	F147	Poor perfor	Stress and	0.6
Map17	F130	F01	Physical in	Bad experi	0.8
Map17	F130	F147	Physical in	Stress and	0.8
Map17	F51	F01	Increase in	Bad experi	0.8
Map17	F51	F60	Increase in	Increased i	0.6
Map17	F51	F130	Increase in	Physical in	0.4
Map17	F51	F147	Increase in	Stress and	0.8
Map17	F31	F20	Fear of eac	Difficult bre	0.2
Map17	F31	F01	Fear of eac	Bad experi	0.2
Map17	F31	F10	Fear of eac	Compulsor	0.4
Map17	F78	F01	Less food t	Bad experi	0.8
Map17	F78	F96	Less food t	More hung	0.8
Map17	F38	F01	Health wor	Bad experi	0.8
Map17	F38	F63	Health wor	Increased (	0.8
Map17	F38	F14	Health wor	Could not g	0.8
Map17	F10	F20	Compulsor	Difficult bre	0.2

For Peer Review

Map17	F10	F01	Compulsor	Bad experi	0.2
Map17	F70	F40	Jobs lost	Household	0.6
Map17	F70	F88	Jobs lost	Men unabl	0.8
Map17	F70	F47	Jobs lost	Increase in	0.6
Map17	F70	F87	Jobs lost	Men stayin	0.8
Map17	F70	F01	Jobs lost	Bad experi	0.6
Map17	F14	F01	Could not g	Bad experi	0.8
Map17	F14	F63	Could not g	Increased	0.8
Map17	F142	F01	School/ma	Bad experi	0.4
Map17	F142	F60	School/ma	Increased i	0.4
Map17	F142	F158	School/ma	Youth/child	0.6
Map17	F142	F16	School/ma	Delay in ed	0.6
Map17	F142	F133	School/ma	Poor perfor	0.4
Map17	F142	F130	School/ma	Physical inj	0.4
Map17	F142	F51	School/ma	Increase in	0.4
Map17	F142	F147	School/ma	Stress and	0.4
Map17	F147	F01	Stress and	Bad experi	1
Map17	F120	F40	No transpo	Household	0.6
Map17	F120	F88	No transpo	Men unabl	0.6
Map17	F120	F47	No transpo	Increase in	0.6
Map17	F120	F87	No transpo	Men stayin	0.6
Map17	F120	F82	No transpo	Lost invest	0.8
Map17	F120	F07	No transpo	Businesses	0.8
Map17	F120	F01	No transpo	Bad experi	0.8
Map17	F120	F64	No transpo	Increased	0.8
Map17	F120	F96	No transpo	More hung	0.8
Map17	F120	F78	No transpo	Less food t	0.8
Map17	F120	F70	No transpo	Jobs lost	0.6
Map17	F144	F01	Social dista	Bad experi	0.6
Map17	F144	F147	Social dista	Stress and	0.6
Map17	F144	F106	Social dista	No congreg	0.8
Map17	F106	F01	No congreg	Bad experi	0.6
Map17	F106	F147	No congreg	Stress and	0.6
Map17	F101	F82	No access	Lost invest	0.6
Map17	F101	F01	No access	Bad experi	0.6
Map17	F101	F64	No access	Increased	0.6
Map17	F101	F96	No access	More hung	0.6
Map17	F101	F132	No access	Poor harve	0.6
Map17	F101	F78	No access	Less food t	0.6
Map17	F100	F01	Mosques/c	Bad experi	0.6
Map17	F100	F147	Mosques/c	Stress and	0.6
Map17	F100	F144	Mosques/c	Social dista	0.6
Map17	F100	F106	Mosques/c	No congreg	0.6
Map18	F85	F82	Markets/sh	Lost invest	0.6
Map18	F85	F07	Markets/sh	Businesses	0.6
Map18	F85	F01	Markets/sh	Bad experi	0.8
Map18	F85	F64	Markets/sh	Increased	0.6
Map18	F85	F63	Markets/sh	Increased	0.6
Map18	F85	F96	Markets/sh	More hung	0.8
Map18	F85	F135	Markets/sh	Price hike	0.8
Map18	F85	F78	Markets/sh	Less food t	0.8
Map18	F85	F143	Markets/sh	Shortage o	0.8
Map18	F80	F85	Lockdown	Markets/sh	0.8
Map18	F80	F82	Lockdown	Lost invest	0.6
Map18	F80	F07	Lockdown	Businesses	0.6
Map18	F80	F01	Lockdown	Bad experi	0.8

For Peer Review

Map18	F80	F64	Lockdown	Increased	0.6
Map18	F80	F63	Lockdown	Increased	0.6
Map18	F80	F62	Lockdown	Increased	0.6
Map18	F80	F158	Lockdown	Youth/child	0.6
Map18	F80	F16	Lockdown	Delay in ec	0.6
Map18	F80	F90	Lockdown	More burde	0.6
Map18	F80	F96	Lockdown	More hung	0.8
Map18	F80	F133	Lockdown	Poor perfor	0.6
Map18	F80	F130	Lockdown	Physical in	0.4
Map18	F80	F110	Lockdown	No funerals	0.8
Map18	F80	F135	Lockdown	Price hike	0.8
Map18	F80	F124	Lockdown	Not visiting	0.6
Map18	F80	F32	Lockdown	Feeling soc	0.6
Map18	F80	F78	Lockdown	Less food t	0.8
Map18	F80	F38	Lockdown	Health wor	0.6
Map18	F80	F13	Lockdown	Could not	0.4
Map18	F80	F70	Lockdown	Jobs lost	0.6
Map18	F80	F98	Lockdown	More mate	0.4
Map18	F80	F14	Lockdown	Could not g	0.6
Map18	F80	F73	Lockdown	Lack of del	0.4
Map18	F80	F143	Lockdown	Shortage o	0.8
Map18	F80	F142	Lockdown	School/ma	0.8
Map18	F80	F147	Lockdown	Stress and	0.8
Map18	F80	F120	Lockdown	No transpo	0.8
Map18	F80	F144	Lockdown	Social dista	0.6
Map18	F80	F148	Lockdown	TBA not att	0.4
Map18	F80	F106	Lockdown	No congreg	0.8
Map18	F80	F100	Lockdown	Mosques/c	1
Map18	F82	F01	Lost invest	Bad experi	0.6
Map18	F82	F64	Lost invest	Increased	0.6
Map18	F07	F82	Businesses	Lost invest	0.6
Map18	F07	F01	Businesses	Bad experi	0.6
Map18	F07	F64	Businesses	Increased	0.6
Map18	F20	F01	Difficult bre	Bad experi	0.4
Map18	F29	F20	Fear of cor	Difficult bre	0.2
Map18	F29	F01	Fear of cor	Bad experi	0.4
Map18	F29	F63	Fear of cor	Increased	0.4
Map18	F29	F124	Fear of cor	Not visiting	0.4
Map18	F29	F10	Fear of cor	Compulsor	0.4
Map18	F64	F01	Increased	Bad experi	0.8
Map18	F63	F01	Increased	Bad experi	0.8
Map18	F62	F01	Increased	Bad experi	0.6
Map18	F158	F01	Youth/child	Bad experi	0.4
Map18	F158	F130	Youth/child	Physical in	0.4
Map18	F158	F13	Youth/child	Could not	0.4
Map18	F16	F01	Delay in ec	Bad experi	0.6
Map18	F16	F133	Delay in ec	Poor perfor	0.8
Map18	F90	F01	More burde	Bad experi	0.6
Map18	F90	F147	More burde	Stress and	0.6
Map18	F96	F01	More hung	Bad experi	0.8
Map18	F96	F63	More hung	Increased	0.6
Map18	F133	F01	Poor perfor	Bad experi	0.6
Map18	F130	F01	Physical in	Bad experi	0.6
Map18	F110	F01	No funerals	Bad experi	0.6
Map18	F110	F147	No funerals	Stress and	0.6
Map18	F135	F01	Price hike	Bad experi	0.8

For Peer Review

Map18	F135	F63	Price hike	Increased (	0.6
Map18	F135	F96	Price hike	More hung	0.8
Map18	F135	F78	Price hike	Less food t	0.8
Map18	F124	F01	Not visiting	Bad experi	0.6
Map18	F124	F63	Not visiting	Increased (	0.6
Map18	F32	F01	Feeling soc	Bad experi	0.6
Map18	F32	F62	Feeling soc	Increased r	0.8
Map18	F78	F01	Less food t	Bad experi	0.8
Map18	F78	F63	Less food t	Increased (	0.6
Map18	F78	F96	Less food t	More hung	0.8
Map18	F38	F01	Health wor	Bad experi	0.6
Map18	F38	F63	Health wor	Increased (	0.6
Map18	F38	F14	Health wor	Could not g	0.6
Map18	F13	F01	Could not c	Bad experi	0.6
Map18	F13	F130	Could not c	Physical in	0.6
Map18	F10	F20	Compulsor	Difficult bre	0.2
Map18	F10	F01	Compulsor	Bad experi	0.2
Map18	F70	F01	Jobs lost	Bad experi	0.6
Map18	F70	F90	Jobs lost	More burde	0.6
Map18	F70	F147	Jobs lost	Stress and	0.6
Map18	F98	F01	More mate	Bad experi	0.4
Map18	F14	F01	Could not g	Bad experi	0.6
Map18	F14	F63	Could not g	Increased (	0.6
Map18	F73	F01	Lack of del	Bad experi	0.4
Map18	F73	F98	Lack of del	More mate	0.4
Map18	F73	F148	Lack of del	TBA not att	0.4
Map18	F143	F01	Shortage o	Bad experi	0.8
Map18	F143	F63	Shortage o	Increased (	0.6
Map18	F143	F96	Shortage o	More hung	0.8
Map18	F143	F135	Shortage o	Price hike	0.8
Map18	F143	F78	Shortage o	Less food t	0.8
Map18	F142	F01	School/ma	Bad experi	0.6
Map18	F142	F158	School/ma	Youth/child	0.6
Map18	F142	F16	School/ma	Delay in ec	0.6
Map18	F142	F90	School/ma	More burde	0.6
Map18	F142	F133	School/ma	Poor perfor	0.6
Map18	F142	F130	School/ma	Physical in	0.4
Map18	F142	F13	School/ma	Could not c	0.4
Map18	F142	F70	School/ma	Jobs lost	0.6
Map18	F142	F147	School/ma	Stress and	0.6
Map18	F147	F01	Stress and	Bad experi	0.6
Map18	F120	F01	No transpo	Bad experi	0.8
Map18	F120	F63	No transpo	Increased (	0.6
Map18	F120	F96	No transpo	More hung	0.8
Map18	F120	F135	No transpo	Price hike	0.8
Map18	F120	F124	No transpo	Not visiting	0.6
Map18	F120	F78	No transpo	Less food t	0.8
Map18	F120	F38	No transpo	Health wor	0.6
Map18	F120	F98	No transpo	More mate	0.4
Map18	F120	F14	No transpo	Could not g	0.6
Map18	F120	F73	No transpo	Lack of del	0.4
Map18	F120	F143	No transpo	Shortage o	0.8
Map18	F120	F148	No transpo	TBA not att	0.4
Map18	F144	F01	Social dista	Bad experi	0.6
Map18	F144	F62	Social dista	Increased r	0.6
Map18	F144	F32	Social dista	Feeling soc	0.6

For Peer Review

Map18	F148	F01	TBA not at	Bad experi	0.4
Map18	F148	F98	TBA not at	More mate	0.4
Map18	F106	F01	No congreg	Bad experi	0.6
Map18	F106	F62	No congreg	Increased r	0.6
Map18	F106	F32	No congreg	Feeling soc	0.6
Map18	F106	F147	No congreg	Stress and	0.8
Map18	F106	F144	No congreg	Social dista	0.6
Map18	F100	F01	Mosques/c	Bad experi	0.6
Map18	F100	F62	Mosques/c	Increased r	0.6
Map18	F100	F32	Mosques/c	Feeling soc	0.6
Map18	F100	F147	Mosques/c	Stress and	0.8
Map18	F100	F144	Mosques/c	Social dista	0.6
Map18	F100	F106	Mosques/c	No congreg	0.8
Map19	F40	F47	Household	Increase in	0.4
Map19	F40	F01	Household	Bad experi	0.4
Map19	F88	F40	Men unable	Household	0.6
Map19	F88	F47	Men unable	Increase in	0.4
Map19	F88	F01	Men unable	Bad experi	0.4
Map19	F47	F01	Increase in	Bad experi	0.4
Map19	F85	F40	Markets/sh	Household	0.6
Map19	F85	F88	Markets/sh	Men unable	0.8
Map19	F85	F47	Markets/sh	Increase in	0.4
Map19	F85	F87	Markets/sh	Men stayin	0.8
Map19	F85	F83	Markets/sh	Malnutrition	0.6
Map19	F85	F07	Markets/sh	Businesses	0.8
Map19	F85	F01	Markets/sh	Bad experi	0.6
Map19	F85	F64	Markets/sh	Increased p	0.8
Map19	F85	F63	Markets/sh	Increased c	0.6
Map19	F85	F96	Markets/sh	More hung	0.8
Map19	F85	F98	Markets/sh	More mate	0.4
Map19	F85	F78	Markets/sh	Less food t	0.8
Map19	F85	F70	Markets/sh	Jobs lost	0.8
Map19	F85	F99	Markets/sh	More unwa	0.6
Map19	F87	F40	Men stayin	Household	0.6
Map19	F87	F88	Men stayin	Men unable	1
Map19	F87	F47	Men stayin	Increase in	0.4
Map19	F87	F01	Men stayin	Bad experi	0.4
Map19	F87	F98	Men stayin	More mate	0.4
Map19	F87	F99	Men stayin	More unwa	0.6
Map19	F80	F40	Lockdown	Household	0.6
Map19	F80	F88	Lockdown	Men unable	0.8
Map19	F80	F47	Lockdown	Increase in	0.4
Map19	F80	F85	Lockdown	Markets/sh	1
Map19	F80	F87	Lockdown	Men stayin	0.8
Map19	F80	F83	Lockdown	Malnutrition	0.6
Map19	F80	F07	Lockdown	Businesses	0.8
Map19	F80	F01	Lockdown	Bad experi	0.6
Map19	F80	F67	Lockdown	Increased s	0.6
Map19	F80	F64	Lockdown	Increased p	0.8
Map19	F80	F63	Lockdown	Increased c	0.6
Map19	F80	F62	Lockdown	Increased r	0.6
Map19	F80	F60	Lockdown	Increased i	0.6
Map19	F80	F158	Lockdown	Youth/child	0.8
Map19	F80	F16	Lockdown	Delay in ec	0.8
Map19	F80	F96	Lockdown	More hung	0.8
Map19	F80	F118	Lockdown	No social g	0.8

For Peer Review

Map19	F80	F98	Lockdown	More mate	0.4
Map19	F80	F131	Lockdown	Physical pu	0.4
Map19	F80	F54	Lockdown	Increase in	0.6
Map19	F80	F58	Lockdown	Increased (	0.6
Map19	F80	F32	Lockdown	Feeling soc	0.6
Map19	F80	F78	Lockdown	Less food t	0.8
Map19	F80	F133	Lockdown	Poor perfor	0.6
Map19	F80	F39	Lockdown	HIV/STI inc	0.4
Map19	F80	F76	Lockdown	Less affect	0.6
Map19	F80	F70	Lockdown	Jobs lost	0.8
Map19	F80	F142	Lockdown	School/mar	1
Map19	F80	F99	Lockdown	More unwa	0.6
Map19	F80	F147	Lockdown	Stress and	0.6
Map19	F80	F120	Lockdown	No transpo	0.8
Map19	F80	F106	Lockdown	No congreg	0.8
Map19	F80	F100	Lockdown	Mosques/c	1
Map19	F83	F01	Malnutritior	Bad experi	0.6
Map19	F83	F63	Malnutritior	Increased (	0.6
Map19	F07	F83	Businesses	Malnutritior	0.6
Map19	F07	F01	Businesses	Bad experi	0.6
Map19	F07	F64	Businesses	Increased p	0.8
Map19	F07	F63	Businesses	Increased (	0.6
Map19	F07	F96	Businesses	More hung	0.8
Map19	F07	F78	Businesses	Less food t	0.8
Map19	F20	F01	Difficult bre	Bad experi	0.4
Map19	F67	F01	Increased s	Bad experi	0.4
Map19	F67	F131	Increased s	Physical pu	0.4
Map19	F29	F01	Fear of cor	Bad experi	0.6
Map19	F29	F63	Fear of cor	Increased (	0.6
Map19	F29	F31	Fear of cor	Fear of eac	0.6
Map19	F29	F38	Fear of cor	Health wor	0.6
Map19	F29	F14	Fear of cor	Could not g	0.6
Map19	F64	F83	Increased p	Malnutritior	0.6
Map19	F64	F01	Increased p	Bad experi	0.6
Map19	F64	F63	Increased p	Increased (	0.6
Map19	F64	F96	Increased p	More hung	0.8
Map19	F64	F78	Increased p	Less food t	0.8
Map19	F63	F01	Increased (	Bad experi	0.6
Map19	F62	F01	Increased r	Bad experi	0.4
Map19	F60	F01	Increased i	Bad experi	0.4
Map19	F60	F67	Increased i	Increased s	0.6
Map19	F60	F62	Increased i	Increased r	0.4
Map19	F60	F131	Increased i	Physical pu	0.4
Map19	F60	F54	Increased i	Increase in	0.6
Map19	F60	F58	Increased i	Increased (	0.6
Map19	F60	F39	Increased i	HIV/STI inc	0.4
Map19	F158	F01	Youth/child	Bad experi	0.6
Map19	F158	F67	Youth/child	Increased s	0.6
Map19	F158	F62	Youth/child	Increased r	0.4
Map19	F158	F60	Youth/child	Increased i	0.6
Map19	F158	F16	Youth/child	Delay in ec	0.8
Map19	F158	F131	Youth/child	Physical pu	0.4
Map19	F158	F54	Youth/child	Increase in	0.6
Map19	F158	F58	Youth/child	Increased (	0.6
Map19	F158	F133	Youth/child	Poor perfor	0.6
Map19	F158	F39	Youth/child	HIV/STI inc	0.4

For Peer Review

Map19	F158	F147	Youth/child	Stress and	0.4
Map19	F16	F01	Delay in ec	Bad experi	0.6
Map19	F16	F133	Delay in ec	Poor perfor	0.6
Map19	F16	F147	Delay in ec	Stress and	0.4
Map19	F96	F83	More hung	Malnutritior	0.6
Map19	F96	F01	More hung	Bad experi	0.6
Map19	F96	F63	More hung	Increased (	0.6
Map19	F118	F01	No social g	Bad experi	0.4
Map19	F118	F62	No social g	Increased r	0.6
Map19	F118	F32	No social g	Feeling soc	0.6
Map19	F98	F01	More mate	Bad experi	0.4
Map19	F131	F01	Physical pu	Bad experi	0.4
Map19	F54	F01	Increase in	Bad experi	0.4
Map19	F54	F62	Increase in	Increased r	0.4
Map19	F54	F39	Increase in	HIV/STI inc	0.4
Map19	F58	F01	Increased (	Bad experi	0.4
Map19	F58	F67	Increased (	Increased s	0.6
Map19	F58	F131	Increased (	Physical pu	0.4
Map19	F30	F01	Fear of dea	Bad experi	0.6
Map19	F30	F29	Fear of dea	Fear of cor	0.8
Map19	F30	F63	Fear of dea	Increased (	0.6
Map19	F30	F31	Fear of dea	Fear of eac	0.6
Map19	F30	F38	Fear of dea	Health wor	0.6
Map19	F30	F14	Fear of dea	Could not g	0.6
Map19	F31	F01	Fear of eac	Bad experi	0.4
Map19	F32	F01	Feeling soc	Bad experi	0.4
Map19	F32	F62	Feeling soc	Increased r	0.6
Map19	F78	F83	Less food t	Malnutritior	0.6
Map19	F78	F01	Less food t	Bad experi	0.6
Map19	F78	F63	Less food t	Increased (	0.6
Map19	F78	F96	Less food t	More hung	1
Map19	F133	F01	Poor perfor	Bad experi	0.6
Map19	F133	F147	Poor perfor	Stress and	0.4
Map19	F38	F01	Health wor	Bad experi	0.6
Map19	F38	F63	Health wor	Increased (	0.6
Map19	F38	F14	Health wor	Could not g	0.6
Map19	F39	F01	HIV/STI inc	Bad experi	0.4
Map19	F39	F62	HIV/STI inc	Increased r	0.4
Map19	F76	F01	Less affect	Bad experi	0.4
Map19	F76	F62	Less affect	Increased r	0.4
Map19	F76	F32	Less affect	Feeling soc	0.4
Map19	F70	F40	Jobs lost	Household	0.6
Map19	F70	F88	Jobs lost	Men unable	0.8
Map19	F70	F47	Jobs lost	Increase in	0.4
Map19	F70	F87	Jobs lost	Men stayin	0.8
Map19	F70	F01	Jobs lost	Bad experi	0.4
Map19	F70	F98	Jobs lost	More mate	0.4
Map19	F70	F99	Jobs lost	More unwa	0.6
Map19	F14	F01	Could not g	Bad experi	0.6
Map19	F14	F63	Could not g	Increased (	0.6
Map19	F142	F01	School/ma	Bad experi	0.6
Map19	F142	F67	School/ma	Increased s	0.6
Map19	F142	F62	School/ma	Increased r	0.4
Map19	F142	F60	School/ma	Increased i	0.6
Map19	F142	F158	School/ma	Youth/child	0.8
Map19	F142	F16	School/ma	Delay in ec	0.8

For Peer Review

Map19	F142	F131	School/ma	Physical pu	0.4
Map19	F142	F54	School/ma	Increase in	0.6
Map19	F142	F58	School/ma	Increased (	0.6
Map19	F142	F133	School/ma	Poor perfor	0.6
Map19	F142	F39	School/ma	HIV/STI inc	0.4
Map19	F142	F147	School/ma	Stress and	0.4
Map19	F99	F01	More unwa	Bad experi	0.4
Map19	F99	F98	More unwa	More mate	0.4
Map19	F147	F01	Stress and	Bad experi	0.6
Map19	F120	F01	No transpo	Bad experi	0.4
Map19	F120	F62	No transpo	Increased r	0.4
Map19	F120	F32	No transpo	Feeling soc	0.4
Map19	F120	F76	No transpo	Less affect	0.6
Map19	F10	F20	Compulsor	Difficult bre	0.2
Map19	F10	F01	Compulsor	Bad experi	0.2
Map19	F106	F01	No congreg	Bad experi	0.6
Map19	F106	F147	No congreg	Stress and	0.6
Map19	F100	F01	Mosques/c	Bad experi	0.6
Map19	F100	F147	Mosques/c	Stress and	0.6
Map19	F100	F106	Mosques/c	No congreg	0.8
Map20	F85	F01	Markets/sh	Bad experi	0.8
Map20	F85	F64	Markets/sh	Increased p	0.8
Map20	F85	F63	Markets/sh	Increased (	0.6
Map20	F85	F96	Markets/sh	More hung	0.6
Map20	F85	F78	Markets/sh	Less food t	0.6
Map20	F85	F143	Markets/sh	Shortage o	0.8
Map20	F85	F104	Markets/sh	No buyers	0.6
Map20	F80	F85	Lockdown	Markets/sh	0.6
Map20	F80	F82	Lockdown	Lost invest	0.6
Map20	F80	F01	Lockdown	Bad experi	0.6
Map20	F80	F26	Lockdown	Farmers/he	0.6
Map20	F80	F64	Lockdown	Increased p	0.6
Map20	F80	F63	Lockdown	Increased (	0.6
Map20	F80	F62	Lockdown	Increased r	0.4
Map20	F80	F96	Lockdown	More hung	0.6
Map20	F80	F113	Lockdown	No nomadi	0.8
Map20	F80	F32	Lockdown	Feeling soc	0.4
Map20	F80	F34	Lockdown	Fine payme	0.6
Map20	F80	F78	Lockdown	Less food t	0.6
Map20	F80	F16	Lockdown	Delay in ec	0.6
Map20	F80	F143	Lockdown	Shortage o	0.6
Map20	F80	F142	Lockdown	School/ma	1
Map20	F80	F140	Lockdown	Restriction	0.8
Map20	F80	F147	Lockdown	Stress and	0.6
Map20	F80	F120	Lockdown	No transpo	0.4
Map20	F80	F144	Lockdown	Social dista	0.4
Map20	F80	F149	Lockdown	Teachers a	0.6
Map20	F80	F106	Lockdown	No congreg	1
Map20	F80	F104	Lockdown	No buyers	0.6
Map20	F80	F100	Lockdown	Mosques/c	1
Map20	F82	F01	Lost invest	Bad experi	1
Map20	F20	F01	Difficult bre	Bad experi	0.4
Map20	F26	F01	Farmers/he	Bad experi	0.8
Map20	F26	F34	Farmers/he	Fine payme	0.8
Map20	F29	F20	Fear of cor	Difficult bre	0.2
Map20	F29	F01	Fear of cor	Bad experi	0.4

For Peer Review

Map20	F29	F62	Fear of cor	Increased r	0.2
Map20	F29	F154	Fear of cor	Wearing fa	0.2
Map20	F29	F118	Fear of cor	No social g	0.6
Map20	F29	F10	Fear of cor	Compulsor	0.8
Map20	F29	F147	Fear of cor	Stress and	0.4
Map20	F29	F106	Fear of cor	No congreg	0.4
Map20	F64	F01	Increased p	Bad experi	1
Map20	F63	F01	Increased c	Bad experi	0.8
Map20	F62	F01	Increased r	Bad experi	0.6
Map20	F154	F20	Wearing fa	Difficult bre	0.6
Map20	F154	F01	Wearing fa	Bad experi	0.4
Map20	F118	F01	No social g	Bad experi	0.4
Map20	F118	F62	No social g	Increased r	0.2
Map20	F118	F147	No social g	Stress and	0.4
Map20	F118	F106	No social g	No congreg	0.4
Map20	F96	F01	More hung	Bad experi	0.8
Map20	F96	F63	More hung	Increased c	0.8
Map20	F111	F01	No governr	Bad experi	0.6
Map20	F111	F63	No governr	Increased c	0.6
Map20	F111	F96	No governr	More hung	0.6
Map20	F111	F78	No governr	Less food t	0.8
Map20	F113	F82	No nomadi	Lost invest	0.6
Map20	F113	F01	No nomadi	Bad experi	0.6
Map20	F113	F26	No nomadi	Farmers/he	0.6
Map20	F113	F34	No nomadi	Fine payme	0.6
Map20	F30	F01	Fear of dea	Bad experi	0.6
Map20	F30	F147	Fear of dea	Stress and	0.6
Map20	F32	F01	Feeling soc	Bad experi	0.6
Map20	F32	F62	Feeling soc	Increased r	0.6
Map20	F34	F01	Fine payme	Bad experi	0.8
Map20	F78	F01	Less food t	Bad experi	0.6
Map20	F78	F63	Less food t	Increased c	0.6
Map20	F78	F96	Less food t	More hung	0.6
Map20	F10	F20	Compulsor	Difficult bre	0.2
Map20	F10	F01	Compulsor	Bad experi	0.2
Map20	F10	F154	Compulsor	Wearing fa	0.2
Map20	F16	F01	Delay in ec	Bad experi	1
Map20	F16	F147	Delay in ec	Stress and	0.6
Map20	F143	F01	Shortage o	Bad experi	0.6
Map20	F143	F63	Shortage o	Increased c	0.6
Map20	F143	F96	Shortage o	More hung	0.6
Map20	F143	F78	Shortage o	Less food t	0.6
Map20	F142	F01	School/ma	Bad experi	0.6
Map20	F142	F16	School/ma	Delay in ec	0.6
Map20	F142	F147	School/ma	Stress and	0.6
Map20	F142	F149	School/ma	Teachers a	0.6
Map20	F141	F01	Rumors on	Bad experi	0.8
Map20	F141	F147	Rumors on	Stress and	0.8
Map20	F140	F82	Restriction	Lost invest	0.6
Map20	F140	F01	Restriction	Bad experi	0.6
Map20	F140	F26	Restriction	Farmers/he	0.6
Map20	F140	F113	Restriction	No nomadi	1
Map20	F140	F34	Restriction	Fine payme	0.6
Map20	F147	F01	Stress and	Bad experi	1
Map20	F120	F01	No transpo	Bad experi	0.6
Map20	F120	F63	No transpo	Increased c	0.6

For Peer Review

Map20	F120	F96	No transpo	More hung	0.6
Map20	F120	F78	No transpo	Less food t	0.6
Map20	F120	F143	No transpo	Shortage o	0.6
Map20	F144	F01	Social dista	Bad experi	0.6
Map20	F144	F62	Social dista	Increased r	0.6
Map20	F144	F32	Social dista	Feeling soc	0.8
Map20	F149	F01	Teachers a	Bad experi	1
Map20	F149	F16	Teachers a	Delay in ec	1
Map20	F149	F147	Teachers a	Stress and	0.6
Map20	F106	F01	No congreg	Bad experi	0.6
Map20	F106	F147	No congreg	Stress and	0.6
Map20	F104	F01	No buyers	Bad experi	0.6
Map20	F104	F64	No buyers	Increased p	0.6
Map20	F100	F01	Mosques/c	Bad experi	0.6
Map20	F100	F62	Mosques/c	Increased r	0.4
Map20	F100	F32	Mosques/c	Feeling soc	0.4
Map20	F100	F147	Mosques/c	Stress and	0.6
Map20	F100	F144	Mosques/c	Social dista	0.4
Map20	F100	F106	Mosques/c	No congreg	1
Map21	F88	F83	Men unable	Malnutritior	0.6
Map21	F88	F01	Men unable	Bad experi	0.8
Map21	F88	F96	Men unable	More hung	0.8
Map21	F88	F78	Men unable	Less food t	0.8
Map21	F85	F83	Markets/sh	Malnutritior	0.6
Map21	F85	F82	Markets/sh	Lost invest	0.6
Map21	F85	F01	Markets/sh	Bad experi	0.6
Map21	F85	F64	Markets/sh	Increased p	0.6
Map21	F85	F96	Markets/sh	More hung	0.6
Map21	F85	F112	Markets/sh	No home/p	0.6
Map21	F86	F88	Men couldr	Men unable	1
Map21	F86	F83	Men couldr	Malnutritior	0.6
Map21	F86	F01	Men couldr	Bad experi	0.8
Map21	F86	F96	Men couldr	More hung	0.8
Map21	F86	F78	Men couldr	Less food t	0.8
Map21	F86	F147	Men couldr	Stress and	0.4
Map21	F80	F88	Lockdown	Men unable	0.8
Map21	F80	F85	Lockdown	Markets/sh	0.8
Map21	F80	F86	Lockdown	Men couldr	0.8
Map21	F80	F83	Lockdown	Malnutritior	0.6
Map21	F80	F82	Lockdown	Lost invest	0.6
Map21	F80	F01	Lockdown	Bad experi	0.8
Map21	F80	F29	Lockdown	Fear of cor	0.6
Map21	F80	F64	Lockdown	Increased p	0.6
Map21	F80	F63	Lockdown	Increased c	0.4
Map21	F80	F62	Lockdown	Increased r	0.4
Map21	F80	F158	Lockdown	Youth/child	0.6
Map21	F80	F118	Lockdown	No social g	0.4
Map21	F80	F96	Lockdown	More hung	0.8
Map21	F80	F112	Lockdown	No home/p	0.6
Map21	F80	F142	Lockdown	School/ma	0.6
Map21	F80	F31	Lockdown	Fear of eac	0.6
Map21	F80	F32	Lockdown	Feeling soc	0.4
Map21	F80	F78	Lockdown	Less food t	0.8
Map21	F80	F13	Lockdown	Could not c	0.4
Map21	F80	F124	Lockdown	Not visiting	0.6
Map21	F80	F147	Lockdown	Stress and	0.4

For Peer Review

Map21	F80	F120	Lockdown	No transpo	0.8
Map21	F80	F144	Lockdown	Social dista	0.6
Map21	F80	F149	Lockdown	Teachers a	0.8
Map21	F80	F106	Lockdown	No congreg	0.2
Map21	F83	F01	Malnutritior	Bad experi	0.6
Map21	F82	F83	Lost invest	Malnutritior	0.6
Map21	F82	F01	Lost invest	Bad experi	0.8
Map21	F82	F64	Lost invest	Increased	0.8
Map21	F82	F96	Lost invest	More hung	0.8
Map21	F29	F01	Fear of cor	Bad experi	0.4
Map21	F29	F63	Fear of cor	Increased	0.4
Map21	F29	F62	Fear of cor	Increased	0.4
Map21	F29	F118	Fear of cor	No social g	0.4
Map21	F29	F31	Fear of cor	Fear of eac	0.8
Map21	F29	F32	Fear of cor	Feeling soc	0.4
Map21	F29	F124	Fear of cor	Not visiting	0.6
Map21	F29	F144	Fear of cor	Social dista	0.6
Map21	F64	F83	Increased	Malnutritior	0.6
Map21	F64	F01	Increased	Bad experi	1
Map21	F64	F96	Increased	More hung	1
Map21	F63	F01	Increased	Bad experi	0.6
Map21	F62	F01	Increased	Bad experi	0.6
Map21	F158	F01	Youth/child	Bad experi	0.4
Map21	F158	F13	Youth/child	Could not	0.4
Map21	F158	F147	Youth/child	Stress and	0.4
Map21	F118	F01	No social g	Bad experi	0.4
Map21	F118	F62	No social g	Increased	0.4
Map21	F118	F32	No social g	Feeling soc	0.4
Map21	F96	F83	More hung	Malnutritior	0.6
Map21	F96	F01	More hung	Bad experi	1
Map21	F112	F83	No home/p	Malnutritior	0.6
Map21	F112	F82	No home/p	Lost invest	0.8
Map21	F112	F01	No home/p	Bad experi	0.8
Map21	F112	F64	No home/p	Increased	0.8
Map21	F112	F96	No home/p	More hung	0.8
Map21	F142	F01	School/ma	Bad experi	0.4
Map21	F142	F158	School/ma	Youth/child	0.6
Map21	F142	F13	School/ma	Could not	0.4
Map21	F142	F147	School/ma	Stress and	0.4
Map21	F31	F01	Fear of eac	Bad experi	0.4
Map21	F31	F62	Fear of eac	Increased	0.4
Map21	F31	F118	Fear of eac	No social g	0.4
Map21	F31	F32	Fear of eac	Feeling soc	0.4
Map21	F31	F144	Fear of eac	Social dista	0.6
Map21	F32	F01	Feeling soc	Bad experi	0.4
Map21	F32	F62	Feeling soc	Increased	0.4
Map21	F78	F83	Less food t	Malnutritior	0.6
Map21	F78	F01	Less food t	Bad experi	1
Map21	F78	F96	Less food t	More hung	1
Map21	F13	F01	Could not	Bad experi	0.6
Map21	F13	F147	Could not	Stress and	0.6
Map21	F124	F01	Not visiting	Bad experi	0.4
Map21	F124	F63	Not visiting	Increased	0.4
Map21	F147	F01	Stress and	Bad experi	0.6
Map21	F120	F88	No transpo	Men unable	0.8
Map21	F120	F86	No transpo	Men couldr	0.8

For Peer Review

Map21	F120	F83	No transpo	Malnutrition	0.6
Map21	F120	F01	No transpo	Bad experi	0.8
Map21	F120	F63	No transpo	Increased (	0.4
Map21	F120	F96	No transpo	More hung	0.8
Map21	F120	F78	No transpo	Less food t	0.8
Map21	F120	F124	No transpo	Not visiting	0.6
Map21	F120	F147	No transpo	Stress and	0.4
Map21	F144	F01	Social dista	Bad experi	0.4
Map21	F144	F62	Social dista	Increased r	0.4
Map21	F144	F118	Social dista	No social g	0.4
Map21	F144	F32	Social dista	Feeling soc	0.4
Map21	F149	F01	Teachers a	Bad experi	0.4
Map21	F149	F29	Teachers a	Fear of cor	0.6
Map21	F149	F63	Teachers a	Increased (	0.4
Map21	F149	F62	Teachers a	Increased r	0.4
Map21	F149	F158	Teachers a	Youth/child	0.6
Map21	F149	F118	Teachers a	No social g	0.4
Map21	F149	F142	Teachers a	School/mad	0.6
Map21	F149	F31	Teachers a	Fear of eac	0.6
Map21	F149	F32	Teachers a	Feeling soc	0.4
Map21	F149	F13	Teachers a	Could not c	0.4
Map21	F149	F124	Teachers a	Not visiting	0.6
Map21	F149	F147	Teachers a	Stress and	0.4
Map21	F149	F144	Teachers a	Social dista	0.6
Map21	F106	F01	No congreg	Bad experi	0.6
Map21	F106	F147	No congreg	Stress and	0.6
Map22	F85	F82	Markets/sh	Lost invest	0.6
Map22	F85	F07	Markets/sh	Businesses	0.6
Map22	F85	F01	Markets/sh	Bad experi	0.6
Map22	F85	F64	Markets/sh	Increased p	0.6
Map22	F85	F63	Markets/sh	Increased (	0.6
Map22	F85	F96	Markets/sh	More hung	0.6
Map22	F85	F78	Markets/sh	Less food t	0.6
Map22	F85	F147	Markets/sh	Stress and	0.6
Map22	F86	F01	Men couldr	Bad experi	0.8
Map22	F86	F147	Men couldr	Stress and	1
Map22	F80	F85	Lockdown	Markets/sh	0.6
Map22	F80	F86	Lockdown	Men couldr	1
Map22	F80	F82	Lockdown	Lost invest	0.6
Map22	F80	F07	Lockdown	Businesses	0.6
Map22	F80	F01	Lockdown	Bad experi	0.8
Map22	F80	F64	Lockdown	Increased p	0.6
Map22	F80	F63	Lockdown	Increased (	0.6
Map22	F80	F158	Lockdown	Youth/child	0.6
Map22	F80	F96	Lockdown	More hung	0.6
Map22	F80	F142	Lockdown	School/mad	0.8
Map22	F80	F78	Lockdown	Less food t	0.6
Map22	F80	F13	Lockdown	Could not c	0.6
Map22	F80	F147	Lockdown	Stress and	1
Map22	F80	F120	Lockdown	No transpo	1
Map22	F80	F149	Lockdown	Teachers a	1
Map22	F82	F01	Lost invest	Bad experi	0.8
Map22	F82	F64	Lost invest	Increased p	0.6
Map22	F82	F63	Lost invest	Increased (	0.6
Map22	F82	F96	Lost invest	More hung	0.6
Map22	F82	F78	Lost invest	Less food t	0.6

For Peer Review

Map22	F82	F147	Lost invest	Stress and	0.8
Map22	F07	F82	Businesses	Lost invest	0.6
Map22	F07	F01	Businesses	Bad experi	0.6
Map22	F07	F64	Businesses	Increased	0.8
Map22	F07	F63	Businesses	Increased	0.6
Map22	F07	F96	Businesses	More hung	0.6
Map22	F07	F78	Businesses	Less food t	0.6
Map22	F07	F147	Businesses	Stress and	0.6
Map22	F29	F01	Fear of cor	Bad experi	0.4
Map22	F29	F63	Fear of cor	Increased	0.4
Map22	F29	F62	Fear of cor	Increased	0.4
Map22	F29	F08	Fear of cor	Cannot cor	0.2
Map22	F29	F118	Fear of cor	No social g	0.6
Map22	F29	F98	Fear of cor	More mate	0.2
Map22	F29	F31	Fear of cor	Fear of eac	0.4
Map22	F29	F32	Fear of cor	Feeling soc	0.4
Map22	F29	F124	Fear of cor	Not visiting	0.4
Map22	F29	F144	Fear of cor	Social dista	0.6
Map22	F64	F01	Increased	Bad experi	0.6
Map22	F64	F63	Increased	Increased	0.6
Map22	F64	F96	Increased	More hung	0.6
Map22	F64	F78	Increased	Less food t	0.6
Map22	F64	F147	Increased	Stress and	0.4
Map22	F63	F01	Increased	Bad experi	0.4
Map22	F62	F01	Increased	Bad experi	0.4
Map22	F158	F01	Youth/child	Bad experi	0.6
Map22	F158	F13	Youth/child	Could not	0.6
Map22	F158	F147	Youth/child	Stress and	0.6
Map22	F08	F01	Cannot cor	Bad experi	0.4
Map22	F08	F98	Cannot cor	More mate	0.8
Map22	F118	F01	No social g	Bad experi	0.4
Map22	F118	F62	No social g	Increased	0.4
Map22	F118	F32	No social g	Feeling soc	0.4
Map22	F96	F01	More hung	Bad experi	1
Map22	F96	F63	More hung	Increased	0.6
Map22	F98	F01	More mate	Bad experi	0.4
Map22	F142	F01	School/ma	Bad experi	0.6
Map22	F142	F158	School/ma	Youth/child	0.6
Map22	F142	F13	School/ma	Could not	0.6
Map22	F142	F147	School/ma	Stress and	0.6
Map22	F31	F01	Fear of eac	Bad experi	0.2
Map22	F32	F01	Feeling soc	Bad experi	0.4
Map22	F32	F62	Feeling soc	Increased	0.4
Map22	F78	F01	Less food t	Bad experi	0.6
Map22	F78	F63	Less food t	Increased	0.6
Map22	F78	F96	Less food t	More hung	0.6
Map22	F13	F01	Could not	Bad experi	0.8
Map22	F13	F147	Could not	Stress and	0.8
Map22	F124	F01	Not visiting	Bad experi	0.4
Map22	F124	F63	Not visiting	Increased	0.4
Map22	F124	F08	Not visiting	Cannot cor	0.2
Map22	F124	F98	Not visiting	More mate	0.2
Map22	F147	F01	Stress and	Bad experi	0.8
Map22	F120	F86	No transpo	Men couldr	1
Map22	F120	F01	No transpo	Bad experi	0.8
Map22	F120	F147	No transpo	Stress and	1

For Peer Review

Map22	F144	F01	Social dista	Bad experi	0.4
Map22	F144	F62	Social dista	Increased r	0.4
Map22	F144	F118	Social dista	No social g	0.6
Map22	F144	F32	Social dista	Feeling soc	0.4
Map22	F149	F01	Teachers a	Bad experi	0.6
Map22	F149	F158	Teachers a	Youth/child	0.6
Map22	F149	F142	Teachers a	School/mad	0.8
Map22	F149	F13	Teachers a	Could not c	0.6
Map22	F149	F147	Teachers a	Stress and	0.6
Map23	F40	F47	Household	Increase in	0.8
Map23	F40	F01	Household	Bad experi	0.8
Map23	F47	F01	Increase in	Bad experi	1
Map23	F85	F40	Markets/sh	Household	0.8
Map23	F85	F47	Markets/sh	Increase in	0.8
Map23	F85	F07	Markets/sh	Businesses	0.8
Map23	F85	F01	Markets/sh	Bad experi	0.8
Map23	F85	F64	Markets/sh	Increased p	0.8
Map23	F85	F96	Markets/sh	More hung	0.8
Map23	F85	F78	Markets/sh	Less food t	0.8
Map23	F85	F143	Markets/sh	Shortage o	0.8
Map23	F85	F104	Markets/sh	No buyers	0.8
Map23	F80	F40	Lockdown	Household	0.8
Map23	F80	F47	Lockdown	Increase in	0.8
Map23	F80	F85	Lockdown	Markets/sh	0.8
Map23	F80	F82	Lockdown	Lost invest	0.6
Map23	F80	F07	Lockdown	Businesses	0.8
Map23	F80	F01	Lockdown	Bad experi	0.8
Map23	F80	F69	Lockdown	Increased t	0.8
Map23	F80	F64	Lockdown	Increased p	0.8
Map23	F80	F60	Lockdown	Increased i	0.8
Map23	F80	F158	Lockdown	Youth/child	0.8
Map23	F80	F94	Lockdown	More hous	0.6
Map23	F80	F96	Lockdown	More hung	0.8
Map23	F80	F113	Lockdown	No nomadi	0.6
Map23	F80	F78	Lockdown	Less food t	0.8
Map23	F80	F143	Lockdown	Shortage o	0.8
Map23	F80	F142	Lockdown	School/mad	0.8
Map23	F80	F140	Lockdown	Restriction	0.6
Map23	F80	F147	Lockdown	Stress and	0.6
Map23	F80	F120	Lockdown	No transpo	0.6
Map23	F80	F144	Lockdown	Social dista	0.6
Map23	F80	F149	Lockdown	Teachers a	0.8
Map23	F80	F106	Lockdown	No congreg	0.6
Map23	F80	F104	Lockdown	No buyers	0.8
Map23	F80	F100	Lockdown	Mosques/c	0.8
Map23	F82	F01	Lost invest	Bad experi	1
Map23	F07	F01	Businesses	Bad experi	0.8
Map23	F07	F64	Businesses	Increased p	1
Map23	F20	F01	Difficult bre	Bad experi	0.4
Map23	F69	F01	Increased t	Bad experi	0.8
Map23	F29	F01	Fear of cor	Bad experi	0.8
Map23	F29	F63	Fear of cor	Increased c	0.6
Map23	F29	F62	Fear of cor	Increased r	0.6
Map23	F29	F118	Fear of cor	No social g	1
Map23	F29	F124	Fear of cor	Not visiting	0.6
Map23	F29	F32	Fear of cor	Feeling soc	0.6

Map23	F29	F106	Fear of cor	No congreg	0.8
Map23	F64	F01	Increased p	Bad experi	0.8
Map23	F63	F01	Increased c	Bad experi	1
Map23	F62	F01	Increased r	Bad experi	0.8
Map23	F60	F01	Increased i	Bad experi	0.8
Map23	F60	F69	Increased i	Increased t	1
Map23	F158	F01	Youth/child	Bad experi	0.8
Map23	F158	F69	Youth/child	Increased t	0.8
Map23	F158	F60	Youth/child	Increased i	0.8
Map23	F158	F94	Youth/child	More house	0.6
Map23	F158	F147	Youth/child	Stress and	0.6
Map23	F118	F01	No social g	Bad experi	0.8
Map23	F118	F106	No social g	No congreg	0.8
Map23	F94	F01	More house	Bad experi	0.8
Map23	F94	F147	More house	Stress and	0.8
Map23	F96	F40	More hung	Household	0.8
Map23	F96	F47	More hung	Increase in	0.8
Map23	F96	F01	More hung	Bad experi	1
Map23	F113	F82	No nomadi	Lost invest	0.8
Map23	F113	F01	No nomadi	Bad experi	0.8
Map23	F124	F01	Not visiting	Bad experi	0.8
Map23	F124	F63	Not visiting	Increased c	0.8
Map23	F32	F01	Feeling soc	Bad experi	0.8
Map23	F32	F62	Feeling soc	Increased r	0.8
Map23	F78	F40	Less food t	Household	0.8
Map23	F78	F47	Less food t	Increase in	0.8
Map23	F78	F01	Less food t	Bad experi	0.8
Map23	F78	F96	Less food t	More hung	0.8
Map23	F10	F20	Compulsor	Difficult bre	0.2
Map23	F10	F01	Compulsor	Bad experi	0.2
Map23	F143	F40	Shortage o	Household	0.8
Map23	F143	F47	Shortage o	Increase in	0.8
Map23	F143	F01	Shortage o	Bad experi	0.8
Map23	F143	F96	Shortage o	More hung	0.8
Map23	F143	F78	Shortage o	Less food t	1
Map23	F142	F01	School/ma	Bad experi	0.8
Map23	F142	F69	School/ma	Increased t	0.8
Map23	F142	F60	School/ma	Increased i	0.8
Map23	F142	F158	School/ma	Youth/child	0.8
Map23	F142	F94	School/ma	More house	0.6
Map23	F142	F147	School/ma	Stress and	0.6
Map23	F142	F149	School/ma	Teachers a	1
Map23	F140	F82	Restriction	Lost invest	0.8
Map23	F140	F01	Restriction	Bad experi	0.8
Map23	F140	F113	Restriction	No nomadi	1
Map23	F147	F01	Stress and	Bad experi	1
Map23	F120	F82	No transpo	Lost invest	0.8
Map23	F120	F01	No transpo	Bad experi	0.8
Map23	F120	F113	No transpo	No nomadi	0.8
Map23	F120	F140	No transpo	Restriction	0.8
Map23	F144	F01	Social dista	Bad experi	0.8
Map23	F144	F106	Social dista	No congreg	0.8
Map23	F149	F01	Teachers a	Bad experi	0.8
Map23	F149	F69	Teachers a	Increased t	0.8
Map23	F149	F60	Teachers a	Increased i	0.8
Map23	F149	F158	Teachers a	Youth/child	0.8

For Peer Review

Map23	F149	F94	Teachers a	More hous	0.6
Map23	F149	F147	Teachers a	Stress and	0.6
Map23	F106	F01	No congreg	Bad experi	1
Map23	F104	F01	No buyers	Bad experi	0.6
Map23	F104	F64	No buyers	Increased p	0.6
Map23	F100	F01	Mosques/c	Bad experi	0.6
Map23	F100	F144	Mosques/c	Social dista	0.6
Map23	F100	F106	Mosques/c	No congreg	0.6
Map24	F44	F01	Inadequate	Bad experi	0.2
Map24	F44	F92	Inadequate	More COVI	0.2
Map24	F44	F147	Inadequate	Stress and	0.2
Map24	F85	F82	Markets/sh	Lost invest	0.4
Map24	F85	F01	Markets/sh	Bad experi	0.6
Map24	F85	F64	Markets/sh	Increased p	0.8
Map24	F85	F70	Markets/sh	Jobs lost	0.8
Map24	F85	F104	Markets/sh	No buyers	0.4
Map24	F80	F85	Lockdown	Markets/sh	0.6
Map24	F80	F82	Lockdown	Lost invest	0.4
Map24	F80	F01	Lockdown	Bad experi	0.6
Map24	F80	F64	Lockdown	Increased p	0.6
Map24	F80	F63	Lockdown	Increased c	0.4
Map24	F80	F70	Lockdown	Jobs lost	0.6
Map24	F80	F96	Lockdown	More hung	0.4
Map24	F80	F135	Lockdown	Price hike	0.6
Map24	F80	F32	Lockdown	Feeling soc	0.6
Map24	F80	F78	Lockdown	Less food t	0.6
Map24	F80	F16	Lockdown	Delay in ec	0.6
Map24	F80	F143	Lockdown	Shortage o	0.6
Map24	F80	F142	Lockdown	School/mac	0.8
Map24	F80	F147	Lockdown	Stress and	0.6
Map24	F80	F120	Lockdown	No transpo	0.8
Map24	F80	F123	Lockdown	Not going t	0.4
Map24	F80	F144	Lockdown	Social dista	0.6
Map24	F80	F149	Lockdown	Teachers a	0.6
Map24	F80	F106	Lockdown	No congreg	0.6
Map24	F80	F104	Lockdown	No buyers	0.4
Map24	F80	F100	Lockdown	Mosques/c	0.6
Map24	F82	F01	Lost invest	Bad experi	0.6
Map24	F82	F64	Lost invest	Increased p	0.6
Map24	F20	F01	Difficult bre	Bad experi	0.6
Map24	F29	F20	Fear of cor	Difficult bre	0.4
Map24	F29	F01	Fear of cor	Bad experi	0.4
Map24	F29	F154	Fear of cor	Wearing fa	0.6
Map24	F29	F118	Fear of cor	No social g	0.4
Map24	F29	F147	Fear of cor	Stress and	0.4
Map24	F64	F01	Increased p	Bad experi	0.6
Map24	F63	F01	Increased c	Bad experi	0.8
Map24	F154	F20	Wearing fa	Difficult bre	0.4
Map24	F154	F01	Wearing fa	Bad experi	0.4
Map24	F70	F01	Jobs lost	Bad experi	0.6
Map24	F70	F64	Jobs lost	Increased p	1
Map24	F92	F01	More COVI	Bad experi	0.8
Map24	F92	F147	More COVI	Stress and	0.8
Map24	F118	F01	No social g	Bad experi	0.6
Map24	F118	F147	No social g	Stress and	0.6
Map24	F96	F01	More hung	Bad experi	0.8

For Peer Review

Map24	F96	F63	More hung	Increased	0.4
Map24	F96	F96	More hung	More hung	0.4
Map24	F96	F135	More hung	Price hike	0.4
Map24	F96	F78	More hung	Less food t	0.4
Map24	F96	F143	More hung	Shortage o	0.4
Map24	F96	F123	More hung	Not going t	1
Map24	F111	F44	No governr	Inadequate	0.6
Map24	F111	F01	No governr	Bad experi	0.4
Map24	F111	F64	No governr	Increased	0.4
Map24	F111	F92	No governr	More COVI	0.2
Map24	F111	F147	No governr	Stress and	0.2
Map24	F135	F01	Price hike	Bad experi	0.4
Map24	F135	F63	Price hike	Increased	0.4
Map24	F135	F96	Price hike	More hung	0.4
Map24	F135	F135	Price hike	Price hike	0.4
Map24	F135	F78	Price hike	Less food t	0.6
Map24	F135	F143	Price hike	Shortage o	0.4
Map24	F135	F123	Price hike	Not going t	0.4
Map24	F32	F01	Feeling soc	Bad experi	0.8
Map24	F32	F147	Feeling soc	Stress and	0.8
Map24	F78	F01	Less food t	Bad experi	0.4
Map24	F78	F63	Less food t	Increased	0.4
Map24	F78	F96	Less food t	More hung	0.4
Map24	F78	F135	Less food t	Price hike	0.4
Map24	F78	F78	Less food t	Less food t	0.4
Map24	F78	F143	Less food t	Shortage o	0.4
Map24	F78	F123	Less food t	Not going t	0.4
Map24	F16	F01	Delay in ec	Bad experi	1
Map24	F16	F147	Delay in ec	Stress and	0.6
Map24	F143	F01	Shortage o	Bad experi	0.4
Map24	F143	F63	Shortage o	Increased	0.4
Map24	F143	F96	Shortage o	More hung	0.4
Map24	F143	F135	Shortage o	Price hike	0.8
Map24	F143	F78	Shortage o	Less food t	0.6
Map24	F143	F143	Shortage o	Shortage o	0.4
Map24	F143	F123	Shortage o	Not going t	0.4
Map24	F142	F01	School/ma	Bad experi	0.6
Map24	F142	F16	School/ma	Delay in ec	0.6
Map24	F142	F147	School/ma	Stress and	0.6
Map24	F142	F149	School/ma	Teachers a	0.6
Map24	F147	F01	Stress and	Bad experi	1
Map24	F120	F01	No transpo	Bad experi	0.4
Map24	F120	F63	No transpo	Increased	0.4
Map24	F120	F96	No transpo	More hung	0.4
Map24	F120	F135	No transpo	Price hike	0.6
Map24	F120	F78	No transpo	Less food t	0.6
Map24	F120	F143	No transpo	Shortage o	0.6
Map24	F120	F123	No transpo	Not going t	0.4
Map24	F123	F01	Not going t	Bad experi	0.4
Map24	F123	F63	Not going t	Increased	0.4
Map24	F123	F96	Not going t	More hung	0.4
Map24	F123	F135	Not going t	Price hike	0.4
Map24	F123	F78	Not going t	Less food t	0.4
Map24	F123	F143	Not going t	Shortage o	0.4
Map24	F123	F123	Not going t	Not going t	0.4
Map24	F144	F01	Social dista	Bad experi	0.6

For Peer Review

Map24	F144	F32	Social dista	Feeling soc	0.6
Map24	F144	F147	Social dista	Stress and	0.6
Map24	F149	F01	Teachers a	Bad experi	0.8
Map24	F149	F16	Teachers a	Delay in ec	0.8
Map24	F149	F147	Teachers a	Stress and	0.6
Map24	F106	F01	No congreg	Bad experi	0.6
Map24	F106	F147	No congreg	Stress and	0.6
Map24	F104	F82	No buyers	Lost invest	0.8
Map24	F104	F01	No buyers	Bad experi	0.6
Map24	F104	F64	No buyers	Increased p	0.6
Map24	F100	F01	Mosques/c	Bad experi	0.6
Map24	F100	F32	Mosques/c	Feeling soc	0.6
Map24	F100	F147	Mosques/c	Stress and	0.6
Map24	F100	F144	Mosques/c	Social dista	0.6
Map24	F100	F106	Mosques/c	No congreg	0.8
Map25	F40	F01	Household	Bad experi	0.8
Map25	F40	F147	Household	Stress and	0.8
Map25	F89	F01	Misconcep	Bad experi	0.4
Map25	F89	F29	Misconcep	Fear of cor	0.8
Map25	F89	F31	Misconcep	Fear of eac	0.8
Map25	F89	F76	Misconcep	Less affect	0.6
Map25	F85	F40	Markets/sh	Household	0.6
Map25	F85	F87	Markets/sh	Men stayin	0.6
Map25	F85	F82	Markets/sh	Lost invest	0.4
Map25	F85	F07	Markets/sh	Businesses	0.6
Map25	F85	F01	Markets/sh	Bad experi	0.6
Map25	F85	F64	Markets/sh	Increased p	0.6
Map25	F85	F118	Markets/sh	No social g	0.6
Map25	F85	F78	Markets/sh	Less food t	0.6
Map25	F85	F70	Markets/sh	Jobs lost	0.6
Map25	F85	F147	Markets/sh	Stress and	0.6
Map25	F85	F144	Markets/sh	Social dista	0.6
Map25	F87	F40	Men stayin	Household	0.8
Map25	F87	F87	Men stayin	Men stayin	0.6
Map25	F87	F82	Men stayin	Lost invest	0.4
Map25	F87	F07	Men stayin	Businesses	0.6
Map25	F87	F01	Men stayin	Bad experi	0.8
Map25	F87	F64	Men stayin	Increased p	0.6
Map25	F87	F78	Men stayin	Less food t	0.6
Map25	F87	F70	Men stayin	Jobs lost	0.6
Map25	F87	F147	Men stayin	Stress and	0.8
Map25	F80	F40	Lockdown	Household	0.6
Map25	F80	F85	Lockdown	Markets/sh	0.6
Map25	F80	F87	Lockdown	Men stayin	0.6
Map25	F80	F82	Lockdown	Lost invest	0.4
Map25	F80	F07	Lockdown	Businesses	0.6
Map25	F80	F01	Lockdown	Bad experi	0.8
Map25	F80	F67	Lockdown	Increased s	0.4
Map25	F80	F64	Lockdown	Increased p	0.6
Map25	F80	F65	Lockdown	Increased r	0.4
Map25	F80	F158	Lockdown	Youth/child	0.8
Map25	F80	F16	Lockdown	Delay in ec	0.8
Map25	F80	F118	Lockdown	No social g	0.6
Map25	F80	F133	Lockdown	Poor perfor	0.6
Map25	F80	F130	Lockdown	Physical in	0.4
Map25	F80	F99	Lockdown	More unwa	0.2

For Peer Review

Map25	F80	F58	Lockdown	Increased (	0.4
Map25	F80	F78	Lockdown	Less food t	0.8
Map25	F80	F70	Lockdown	Jobs lost	0.6
Map25	F80	F142	Lockdown	School/ma(	1
Map25	F80	F147	Lockdown	Stress and	0.8
Map25	F80	F146	Lockdown	Street haw	0.6
Map25	F80	F123	Lockdown	Not going t	0.6
Map25	F80	F144	Lockdown	Social dista	0.6
Map25	F80	F106	Lockdown	No congreg	0.8
Map25	F80	F101	Lockdown	No access	0.8
Map25	F80	F100	Lockdown	Mosques/c	0.8
Map25	F82	F01	Lost invest	Bad experi	0.6
Map25	F82	F64	Lost invest	Increased p	0.6
Map25	F07	F40	Businesses	Household	0.6
Map25	F07	F87	Businesses	Men stayin	0.6
Map25	F07	F82	Businesses	Lost invest	0.4
Map25	F07	F07	Businesses	Businesses	0.6
Map25	F07	F01	Businesses	Bad experi	0.8
Map25	F07	F64	Businesses	Increased p	0.8
Map25	F07	F78	Businesses	Less food t	0.8
Map25	F07	F70	Businesses	Jobs lost	0.6
Map25	F07	F147	Businesses	Stress and	0.6
Map25	F20	F01	Difficult bre	Bad experi	0.6
Map25	F67	F01	Increased s	Bad experi	0.4
Map25	F67	F130	Increased s	Physical in	0.4
Map25	F29	F01	Fear of cor	Bad experi	0.4
Map25	F29	F31	Fear of cor	Fear of eac	0.8
Map25	F29	F76	Fear of cor	Less affect	0.6
Map25	F64	F01	Increased p	Bad experi	0.8
Map25	F65	F01	Increased r	Bad experi	0.2
Map25	F65	F99	Increased r	More unwa	0.2
Map25	F154	F20	Wearing fa	Difficult bre	0.8
Map25	F154	F01	Wearing fa	Bad experi	0.6
Map25	F158	F01	Youth/child	Bad experi	0.4
Map25	F158	F67	Youth/child	Increased s	0.4
Map25	F158	F65	Youth/child	Increased r	0.4
Map25	F158	F130	Youth/child	Physical in	0.4
Map25	F158	F99	Youth/child	More unwa	0.2
Map25	F158	F58	Youth/child	Increased (	0.4
Map25	F16	F01	Delay in ed	Bad experi	0.4
Map25	F16	F133	Delay in ed	Poor perfor	0.6
Map25	F118	F40	No social g	Household	0.6
Map25	F118	F87	No social g	Men stayin	0.6
Map25	F118	F82	No social g	Lost invest	0.4
Map25	F118	F07	No social g	Businesses	0.6
Map25	F118	F01	No social g	Bad experi	0.6
Map25	F118	F64	No social g	Increased p	0.6
Map25	F118	F78	No social g	Less food t	0.6
Map25	F118	F70	No social g	Jobs lost	0.6
Map25	F118	F147	No social g	Stress and	0.6
Map25	F133	F01	Poor perfor	Bad experi	0.4
Map25	F130	F01	Physical in	Bad experi	0.4
Map25	F99	F01	More unwa	Bad experi	0.2
Map25	F112	F01	No home/p	Bad experi	0.6
Map25	F112	F64	No home/p	Increased p	0.6
Map25	F135	F40	Price hike	Household	0.6

For Peer Review

Map25	F135	F87	Price hike	Men stayin	0.6
Map25	F135	F82	Price hike	Lost invest	0.4
Map25	F135	F07	Price hike	Businesses	0.6
Map25	F135	F01	Price hike	Bad experi	0.6
Map25	F135	F64	Price hike	Increased p	0.6
Map25	F135	F78	Price hike	Less food t	0.6
Map25	F135	F70	Price hike	Jobs lost	0.6
Map25	F135	F147	Price hike	Stress and	0.6
Map25	F58	F01	Increased (	Bad experi	0.4
Map25	F58	F67	Increased (	Increased s	0.6
Map25	F58	F65	Increased (	Increased r	0.4
Map25	F58	F130	Increased (	Physical in	0.4
Map25	F58	F99	Increased (	More unwa	0.2
Map25	F31	F01	Fear of eat	Bad experi	0.4
Map25	F31	F76	Fear of eat	Less affect	0.6
Map25	F32	F01	Feeling soc	Bad experi	0.6
Map25	F32	F147	Feeling soc	Stress and	0.6
Map25	F78	F01	Less food t	Bad experi	1
Map25	F76	F01	Less affect	Bad experi	0.4
Map25	F70	F82	Jobs lost	Lost invest	0.4
Map25	F70	F01	Jobs lost	Bad experi	0.4
Map25	F70	F64	Jobs lost	Increased p	0.4
Map25	F142	F01	School/ma	Bad experi	0.4
Map25	F142	F67	School/ma	Increased s	0.4
Map25	F142	F65	School/ma	Increased r	0.4
Map25	F142	F158	School/ma	Youth/child	0.8
Map25	F142	F16	School/ma	Delay in ec	0.8
Map25	F142	F133	School/ma	Poor perfor	0.6
Map25	F142	F130	School/ma	Physical in	0.4
Map25	F142	F99	School/ma	More unwa	0.2
Map25	F142	F58	School/ma	Increased (	0.4
Map25	F147	F01	Stress and	Bad experi	0.8
Map25	F146	F01	Street haw	Bad experi	0.6
Map25	F146	F64	Street haw	Increased p	0.6
Map25	F123	F01	Not going t	Bad experi	0.8
Map25	F123	F78	Not going t	Less food t	0.8
Map25	F144	F40	Social dista	Household	0.6
Map25	F144	F87	Social dista	Men stayin	0.6
Map25	F144	F82	Social dista	Lost invest	0.4
Map25	F144	F07	Social dista	Businesses	0.6
Map25	F144	F01	Social dista	Bad experi	0.6
Map25	F144	F64	Social dista	Increased p	0.6
Map25	F144	F118	Social dista	No social g	0.8
Map25	F144	F78	Social dista	Less food t	0.6
Map25	F144	F70	Social dista	Jobs lost	0.6
Map25	F144	F147	Social dista	Stress and	0.6
Map25	F10	F20	Compulsor	Difficult bre	0.6
Map25	F10	F01	Compulsor	Bad experi	0.6
Map25	F10	F154	Compulsor	Wearing fa	0.6
Map25	F106	F01	No congreg	Bad experi	0.8
Map25	F106	F147	No congreg	Stress and	0.8
Map25	F101	F01	No access	Bad experi	0.8
Map25	F101	F78	No access	Less food t	0.8
Map25	F101	F123	No access	Not going t	0.6
Map25	F100	F01	Mosques/c	Bad experi	0.8
Map25	F100	F147	Mosques/c	Stress and	0.8

For Peer Review

Map25	F100	F106	Mosques/c	No congreg	0.8
Map26	F40	F47	Household	Increase in	1
Map26	F40	F01	Household	Bad experi	1
Map26	F47	F01	Increase in	Bad experi	1
Map26	F85	F40	Markets/sh	Household	0.6
Map26	F85	F47	Markets/sh	Increase in	0.6
Map26	F85	F07	Markets/sh	Businesses	0.6
Map26	F85	F01	Markets/sh	Bad experi	0.6
Map26	F85	F67	Markets/sh	Increased s	0.6
Map26	F85	F63	Markets/sh	Increased (	0.6
Map26	F85	F96	Markets/sh	More hung	0.6
Map26	F85	F78	Markets/sh	Less food t	0.6
Map26	F85	F143	Markets/sh	Shortage o	0.8
Map26	F80	F40	Lockdown	Household	0.6
Map26	F80	F47	Lockdown	Increase in	0.6
Map26	F80	F85	Lockdown	Markets/sh	0.8
Map26	F80	F07	Lockdown	Businesses	0.6
Map26	F80	F01	Lockdown	Bad experi	0.6
Map26	F80	F67	Lockdown	Increased s	0.6
Map26	F80	F63	Lockdown	Increased (	0.6
Map26	F80	F114	Lockdown	No pilgrima	0.8
Map26	F80	F96	Lockdown	More hung	0.6
Map26	F80	F32	Lockdown	Feeling soc	0.4
Map26	F80	F78	Lockdown	Less food t	0.6
Map26	F80	F75	Lockdown	Lack of hyg	0.4
Map26	F80	F77	Lockdown	Less careg	0.6
Map26	F80	F70	Lockdown	Jobs lost	0.6
Map26	F80	F72	Lockdown	Lack of bas	0.4
Map26	F80	F143	Lockdown	Shortage o	0.8
Map26	F80	F147	Lockdown	Stress and	0.6
Map26	F80	F120	Lockdown	No transpo	1
Map26	F80	F144	Lockdown	Social dista	0.6
Map26	F80	F109	Lockdown	No financia	0.4
Map26	F80	F106	Lockdown	No congreg	0.4
Map26	F80	F100	Lockdown	Mosques/c	1
Map26	F07	F40	Businesses	Household	0.6
Map26	F07	F47	Businesses	Increase in	0.6
Map26	F07	F01	Businesses	Bad experi	0.6
Map26	F07	F78	Businesses	Less food t	0.6
Map26	F20	F01	Difficult bre	Bad experi	0.8
Map26	F67	F01	Increased s	Bad experi	0.6
Map26	F29	F20	Fear of cor	Difficult bre	0.4
Map26	F29	F01	Fear of cor	Bad experi	0.4
Map26	F29	F62	Fear of cor	Increased r	0.4
Map26	F29	F154	Fear of cor	Wearing fa	0.6
Map26	F29	F118	Fear of cor	No social g	0.6
Map26	F64	F01	Increased p	Bad experi	0.8
Map26	F63	F01	Increased (	Bad experi	0.6
Map26	F62	F01	Increased r	Bad experi	0.8
Map26	F154	F20	Wearing fa	Difficult bre	0.4
Map26	F154	F01	Wearing fa	Bad experi	0.4
Map26	F118	F01	No social g	Bad experi	0.4
Map26	F118	F62	No social g	Increased r	0.4
Map26	F114	F01	No pilgrima	Bad experi	0.2
Map26	F114	F147	No pilgrima	Stress and	0.2
Map26	F96	F01	More hung	Bad experi	0.6

For Peer Review

Map26	F96	F67	More hung	Increased s	0.6
Map26	F96	F63	More hung	Increased c	0.8
Map26	F111	F01	No governr	Bad experi	0.6
Map26	F111	F64	No governr	Increased p	0.6
Map26	F31	F20	Fear of eac	Difficult bre	0.4
Map26	F31	F01	Fear of eac	Bad experi	0.4
Map26	F31	F154	Fear of eac	Wearing fa	0.4
Map26	F32	F01	Feeling soc	Bad experi	0.6
Map26	F32	F147	Feeling soc	Stress and	0.6
Map26	F78	F40	Less food t	Household	1
Map26	F78	F47	Less food t	Increase in	1
Map26	F78	F01	Less food t	Bad experi	1
Map26	F75	F01	Lack of hyg	Bad experi	0.8
Map26	F77	F01	Less careg	Bad experi	0.8
Map26	F77	F147	Less careg	Stress and	0.8
Map26	F70	F01	Jobs lost	Bad experi	0.4
Map26	F70	F75	Jobs lost	Lack of hyg	0.4
Map26	F70	F72	Jobs lost	Lack of bas	0.4
Map26	F72	F01	Lack of bas	Bad experi	0.6
Map26	F72	F75	Lack of bas	Lack of hyg	0.6
Map26	F143	F40	Shortage o	Household	0.4
Map26	F143	F47	Shortage o	Increase in	0.4
Map26	F143	F01	Shortage o	Bad experi	0.6
Map26	F143	F67	Shortage o	Increased s	0.6
Map26	F143	F63	Shortage o	Increased c	0.6
Map26	F143	F96	Shortage o	More hung	0.6
Map26	F143	F78	Shortage o	Less food t	0.4
Map26	F124	F01	Not visiting	Bad experi	0.6
Map26	F124	F63	Not visiting	Increased c	0.8
Map26	F141	F20	Rumors on	Difficult bre	0.4
Map26	F141	F01	Rumors on	Bad experi	0.4
Map26	F141	F29	Rumors on	Fear of cor	0.6
Map26	F141	F62	Rumors on	Increased r	0.4
Map26	F141	F154	Rumors on	Wearing fa	0.6
Map26	F141	F118	Rumors on	No social g	0.6
Map26	F147	F01	Stress and	Bad experi	0.8
Map26	F120	F01	No transpo	Bad experi	0.4
Map26	F120	F75	No transpo	Lack of hyg	0.4
Map26	F120	F70	No transpo	Jobs lost	0.6
Map26	F120	F72	No transpo	Lack of bas	0.4
Map26	F120	F109	No transpo	No financia	0.4
Map26	F144	F01	Social dista	Bad experi	0.8
Map26	F144	F32	Social dista	Feeling soc	0.4
Map26	F144	F77	Social dista	Less careg	0.8
Map26	F144	F147	Social dista	Stress and	0.8
Map26	F109	F01	No financia	Bad experi	0.4
Map26	F109	F75	No financia	Lack of hyg	0.4
Map26	F106	F01	No congreg	Bad experi	0.4
Map26	F106	F147	No congreg	Stress and	0.4
Map26	F100	F01	Mosques/c	Bad experi	0.6
Map26	F100	F32	Mosques/c	Feeling soc	0.4
Map26	F100	F77	Mosques/c	Less careg	0.6
Map26	F100	F147	Mosques/c	Stress and	0.6
Map26	F100	F144	Mosques/c	Social dista	0.6
Map26	F100	F106	Mosques/c	No congreg	0.4
Map27	F40	F47	Household	Increase in	0.8

For Peer Review

Map27	F40	F01	Household	Bad experi	0.8
Map27	F47	F01	Increase in	Bad experi	1
Map27	F85	F40	Markets/sh	Household	0.8
Map27	F85	F47	Markets/sh	Increase in	0.8
Map27	F85	F01	Markets/sh	Bad experi	0.8
Map27	F85	F96	Markets/sh	More hung	0.6
Map27	F85	F78	Markets/sh	Less food t	0.8
Map27	F85	F143	Markets/sh	Shortage o	0.8
Map27	F80	F40	Lockdown	Household	0.8
Map27	F80	F47	Lockdown	Increase in	0.8
Map27	F80	F85	Lockdown	Markets/sh	0.8
Map27	F80	F82	Lockdown	Lost invest	0.8
Map27	F80	F01	Lockdown	Bad experi	0.8
Map27	F80	F67	Lockdown	Increased s	0.8
Map27	F80	F64	Lockdown	Increased p	0.8
Map27	F80	F70	Lockdown	Jobs lost	0.8
Map27	F80	F118	Lockdown	No social g	0.4
Map27	F80	F96	Lockdown	More hung	0.6
Map27	F80	F130	Lockdown	Physical in	0.8
Map27	F80	F51	Lockdown	Increase in	0.6
Map27	F80	F32	Lockdown	Feeling soc	0.4
Map27	F80	F78	Lockdown	Less food t	0.8
Map27	F80	F121	Lockdown	No wedding	0.4
Map27	F80	F16	Lockdown	Delay in ec	0.6
Map27	F80	F143	Lockdown	Shortage o	0.8
Map27	F80	F142	Lockdown	School/ma	0.8
Map27	F80	F147	Lockdown	Stress and	0.4
Map27	F80	F120	Lockdown	No transpo	0.8
Map27	F80	F144	Lockdown	Social dista	0.4
Map27	F80	F106	Lockdown	No congreg	0.4
Map27	F80	F100	Lockdown	Mosques/c	0.8
Map27	F82	F01	Lost invest	Bad experi	0.8
Map27	F82	F64	Lost invest	Increased p	0.8
Map27	F20	F01	Difficult bre	Bad experi	0.6
Map27	F20	F63	Difficult bre	Increased c	0.6
Map27	F67	F01	Increased s	Bad experi	1
Map27	F67	F130	Increased s	Physical in	1
Map27	F29	F20	Fear of cor	Difficult bre	0.2
Map27	F29	F01	Fear of cor	Bad experi	0.2
Map27	F29	F63	Fear of cor	Increased c	0.2
Map27	F29	F154	Fear of cor	Wearing fa	0.6
Map27	F64	F01	Increased p	Bad experi	1
Map27	F63	F01	Increased c	Bad experi	0.8
Map27	F154	F20	Wearing fa	Difficult bre	0.2
Map27	F154	F01	Wearing fa	Bad experi	0.2
Map27	F154	F63	Wearing fa	Increased c	0.2
Map27	F70	F82	Jobs lost	Lost invest	0.8
Map27	F70	F01	Jobs lost	Bad experi	0.8
Map27	F70	F67	Jobs lost	Increased s	0.8
Map27	F70	F64	Jobs lost	Increased p	0.8
Map27	F70	F130	Jobs lost	Physical in	0.8
Map27	F118	F01	No social g	Bad experi	0.6
Map27	F118	F121	No social g	No wedding	0.6
Map27	F118	F147	No social g	Stress and	0.6
Map27	F96	F01	More hung	Bad experi	1
Map27	F130	F01	Physical in	Bad experi	1

For Peer Review

Map27	F130	F130	Physical in	Physical in	0.8
Map27	F51	F01	Increase in	Bad experi	0.8
Map27	F32	F01	Feeling soc	Bad experi	0.6
Map27	F32	F147	Feeling soc	Stress and	0.6
Map27	F78	F40	Less food t	Household	0.8
Map27	F78	F47	Less food t	Increase in	0.8
Map27	F78	F01	Less food t	Bad experi	0.8
Map27	F78	F96	Less food t	More hung	0.6
Map27	F121	F01	No wedding	Bad experi	0.8
Map27	F121	F147	No wedding	Stress and	0.8
Map27	F16	F01	Delay in ed	Bad experi	0.8
Map27	F16	F51	Delay in ed	Increase in	0.8
Map27	F143	F40	Shortage o	Household	0.8
Map27	F143	F47	Shortage o	Increase in	0.8
Map27	F143	F01	Shortage o	Bad experi	0.8
Map27	F143	F96	Shortage o	More hung	0.6
Map27	F143	F78	Shortage o	Less food t	0.8
Map27	F142	F01	School/ma	Bad experi	0.6
Map27	F142	F51	School/ma	Increase in	0.6
Map27	F142	F16	School/ma	Delay in ed	0.6
Map27	F147	F01	Stress and	Bad experi	1
Map27	F120	F40	No transpo	Household	0.8
Map27	F120	F47	No transpo	Increase in	0.8
Map27	F120	F82	No transpo	Lost invest	0.8
Map27	F120	F01	No transpo	Bad experi	0.8
Map27	F120	F67	No transpo	Increased s	0.8
Map27	F120	F64	No transpo	Increased p	0.8
Map27	F120	F70	No transpo	Jobs lost	1
Map27	F120	F96	No transpo	More hung	0.6
Map27	F120	F130	No transpo	Physical in	0.8
Map27	F120	F78	No transpo	Less food t	0.8
Map27	F120	F143	No transpo	Shortage o	0.8
Map27	F144	F01	Social dista	Bad experi	0.6
Map27	F144	F118	Social dista	No social g	0.8
Map27	F144	F32	Social dista	Feeling soc	0.4
Map27	F144	F121	Social dista	No wedding	0.6
Map27	F144	F147	Social dista	Stress and	0.6
Map27	F144	F106	Social dista	No congreg	0.6
Map27	F106	F01	No congreg	Bad experi	1
Map27	F106	F147	No congreg	Stress and	1
Map27	F100	F01	Mosques/c	Bad experi	0.4
Map27	F100	F118	Mosques/c	No social g	0.4
Map27	F100	F32	Mosques/c	Feeling soc	0.4
Map27	F100	F121	Mosques/c	No wedding	0.4
Map27	F100	F147	Mosques/c	Stress and	0.4
Map27	F100	F144	Mosques/c	Social dista	0.4
Map27	F100	F106	Mosques/c	No congreg	0.4
Map28	F40	F47	Household	Increase in	0.6
Map28	F40	F01	Household	Bad experi	0.6
Map28	F47	F01	Increase in	Bad experi	0.8
Map28	F85	F82	Markets/sh	Lost invest	0.6
Map28	F85	F07	Markets/sh	Businesses	0.6
Map28	F85	F01	Markets/sh	Bad experi	0.6
Map28	F85	F64	Markets/sh	Increased p	0.6
Map28	F87	F40	Men stayin	Household	0.8
Map28	F87	F88	Men stayin	Men unable	0.8

For Peer Review

Map28	F87	F47	Men stayin	Increase in	0.6
Map28	F87	F87	Men stayin	Men stayin	0.4
Map28	F87	F01	Men stayin	Bad experi	0.6
Map28	F87	F70	Men stayin	Jobs lost	0.8
Map28	F87	F95	Men stayin	More hous	0.8
Map28	F82	F82	Lost invest	Lost invest	1
Map28	F82	F01	Lost invest	Bad experi	1
Map28	F82	F64	Lost invest	Increased	1
Map28	F07	F82	Businesses	Lost invest	1
Map28	F07	F01	Businesses	Bad experi	1
Map28	F07	F64	Businesses	Increased	1
Map28	F69	F82	Increased t	Lost invest	0.4
Map28	F69	F01	Increased t	Bad experi	0.4
Map28	F69	F64	Increased t	Increased	0.4
Map28	F29	F01	Fear of cor	Bad experi	0.8
Map28	F29	F63	Fear of cor	Increased	0.4
Map28	F29	F31	Fear of cor	Fear of eac	0.8
Map28	F29	F124	Fear of cor	Not visiting	0.4
Map28	F64	F82	Increased	Lost invest	1
Map28	F64	F01	Increased	Bad experi	1
Map28	F64	F64	Increased	Increased	1
Map28	F63	F01	Increased	Bad experi	0.8
Map28	F157	F01	Women un	Bad experi	0.2
Map28	F157	F118	Women un	No social g	0.2
Map28	F157	F147	Women un	Stress and	0.2
Map28	F70	F40	Jobs lost	Household	0.4
Map28	F70	F88	Jobs lost	Men unable	0.8
Map28	F70	F47	Jobs lost	Increase in	0.4
Map28	F70	F87	Jobs lost	Men stayin	0.4
Map28	F70	F01	Jobs lost	Bad experi	0.4
Map28	F70	F70	Jobs lost	Jobs lost	0.4
Map28	F70	F95	Jobs lost	More hous	0.4
Map28	F96	F82	More hung	Lost invest	0.4
Map28	F96	F01	More hung	Bad experi	1
Map28	F96	F69	More hung	Increased t	0.4
Map28	F96	F64	More hung	Increased	0.4
Map28	F95	F40	More hous	Household	0.8
Map28	F95	F47	More hous	Increase in	0.6
Map28	F95	F01	More hous	Bad experi	0.6
Map28	F115	F01	No preachi	Bad experi	0.6
Map28	F115	F147	No preachi	Stress and	0.6
Map28	F112	F82	No home/p	Lost invest	1
Map28	F112	F01	No home/p	Bad experi	1
Map28	F112	F64	No home/p	Increased	1
Map28	F142	F01	School/ma	Bad experi	0.8
Map28	F142	F13	School/ma	Could not	0.8
Map28	F142	F16	School/ma	Delay in ec	1
Map28	F142	F147	School/ma	Stress and	0.6
Map28	F31	F01	Fear of eac	Bad experi	1
Map28	F118	F01	No social g	Bad experi	0.4
Map28	F118	F147	No social g	Stress and	0.4
Map28	F78	F82	Less food t	Lost invest	0.4
Map28	F78	F01	Less food t	Bad experi	0.8
Map28	F78	F69	Less food t	Increased t	0.4
Map28	F78	F64	Less food t	Increased	0.4
Map28	F78	F96	Less food t	More hung	0.8

For Peer Review

Map28	F13	F01	Could not c	Bad experi	0.6
Map28	F13	F147	Could not c	Stress and	0.6
Map28	F16	F01	Delay in ec	Bad experi	0.8
Map28	F143	F82	Shortage o	Lost invest	0.4
Map28	F143	F01	Shortage o	Bad experi	0.6
Map28	F143	F69	Shortage o	Increased t	0.4
Map28	F143	F64	Shortage o	Increased p	0.4
Map28	F143	F96	Shortage o	More hung	0.6
Map28	F143	F78	Shortage o	Less food t	0.6
Map28	F124	F01	Not visiting	Bad experi	0.6
Map28	F124	F63	Not visiting	Increased c	0.6
Map28	F140	F40	Restriction	Household	0.4
Map28	F140	F88	Restriction	Men unable	0.4
Map28	F140	F47	Restriction	Increase in	0.4
Map28	F140	F87	Restriction	Men stayin	0.4
Map28	F140	F82	Restriction	Lost invest	0.6
Map28	F140	F01	Restriction	Bad experi	0.6
Map28	F140	F69	Restriction	Increased t	0.4
Map28	F140	F64	Restriction	Increased p	0.6
Map28	F140	F157	Restriction	Women un	0.4
Map28	F140	F70	Restriction	Jobs lost	0.4
Map28	F140	F96	Restriction	More hung	0.4
Map28	F140	F95	Restriction	More hous	0.4
Map28	F140	F112	Restriction	No home/p	0.6
Map28	F140	F118	Restriction	No social g	0.2
Map28	F140	F78	Restriction	Less food t	0.4
Map28	F140	F143	Restriction	Shortage o	0.4
Map28	F140	F147	Restriction	Stress and	0.2
Map28	F140	F120	Restriction	No transpo	0.4
Map28	F140	F104	Restriction	No buyers	0.8
Map28	F147	F01	Stress and	Bad experi	1
Map28	F120	F82	No transpo	Lost invest	0.4
Map28	F120	F01	No transpo	Bad experi	0.6
Map28	F120	F69	No transpo	Increased t	0.4
Map28	F120	F64	No transpo	Increased p	0.4
Map28	F120	F96	No transpo	More hung	0.6
Map28	F120	F78	No transpo	Less food t	0.6
Map28	F120	F143	No transpo	Shortage o	0.6
Map28	F144	F85	Social dista	Markets/sh	1
Map28	F144	F82	Social dista	Lost invest	0.6
Map28	F144	F07	Social dista	Businesses	0.6
Map28	F144	F01	Social dista	Bad experi	0.8
Map28	F144	F64	Social dista	Increased p	0.6
Map28	F144	F115	Social dista	No preachi	0.2
Map28	F144	F142	Social dista	School/mac	0.8
Map28	F144	F13	Social dista	Could not c	0.8
Map28	F144	F16	Social dista	Delay in ec	0.8
Map28	F144	F147	Social dista	Stress and	0.6
Map28	F144	F106	Social dista	No congreg	0.2
Map28	F144	F100	Social dista	Mosques/c	0.2
Map28	F106	F01	No congreg	Bad experi	0.8
Map28	F106	F147	No congreg	Stress and	0.8
Map28	F104	F82	No buyers	Lost invest	0.6
Map28	F104	F01	No buyers	Bad experi	0.6
Map28	F104	F64	No buyers	Increased p	0.6
Map28	F104	F112	No buyers	No home/p	0.6

For Peer Review

Map28	F100	F01	Mosques/c	Bad experi	0.6
Map28	F100	F115	Mosques/c	No preachi	0.8
Map28	F100	F147	Mosques/c	Stress and	0.6
Map28	F100	F106	Mosques/c	No congreg	0.6
Map29	F40	F47	Household	Increase in	0.6
Map29	F40	F01	Household	Bad experi	0.6
Map29	F47	F01	Increase in	Bad experi	0.8
Map29	F85	F40	Markets/sh	Household	0.6
Map29	F85	F47	Markets/sh	Increase in	0.6
Map29	F85	F87	Markets/sh	Men stayin	0.6
Map29	F85	F82	Markets/sh	Lost invest	0.8
Map29	F85	F07	Markets/sh	Businesses	1
Map29	F85	F01	Markets/sh	Bad experi	0.8
Map29	F85	F64	Markets/sh	Increased p	0.8
Map29	F85	F96	Markets/sh	More hung	0.6
Map29	F85	F78	Markets/sh	Less food t	0.6
Map29	F87	F40	Men stayin	Household	0.6
Map29	F87	F47	Men stayin	Increase in	0.6
Map29	F87	F01	Men stayin	Bad experi	0.6
Map29	F80	F40	Lockdown	Household	0.6
Map29	F80	F47	Lockdown	Increase in	0.6
Map29	F80	F85	Lockdown	Markets/sh	1
Map29	F80	F87	Lockdown	Men stayin	0.6
Map29	F80	F82	Lockdown	Lost invest	0.8
Map29	F80	F07	Lockdown	Businesses	1
Map29	F80	F01	Lockdown	Bad experi	0.8
Map29	F80	F69	Lockdown	Increased t	0.4
Map29	F80	F67	Lockdown	Increased s	0.6
Map29	F80	F64	Lockdown	Increased p	0.8
Map29	F80	F63	Lockdown	Increased c	0.4
Map29	F80	F60	Lockdown	Increased i	0.6
Map29	F80	F158	Lockdown	Youth/child	0.6
Map29	F80	F96	Lockdown	More hung	0.6
Map29	F80	F115	Lockdown	No preachi	0.2
Map29	F80	F130	Lockdown	Physical in	0.4
Map29	F80	F78	Lockdown	Less food t	0.6
Map29	F80	F38	Lockdown	Health wor	0.4
Map29	F80	F16	Lockdown	Delay in ec	0.8
Map29	F80	F142	Lockdown	School/ma	0.8
Map29	F80	F147	Lockdown	Stress and	0.8
Map29	F80	F106	Lockdown	No congreg	1
Map29	F80	F65	Lockdown	Increased r	0.4
Map29	F80	F100	Lockdown	Mosques/c	1
Map29	F82	F01	Lost invest	Bad experi	0.8
Map29	F82	F64	Lost invest	Increased p	0.8
Map29	F82	F96	Lost invest	More hung	0.6
Map29	F82	F78	Lost invest	Less food t	0.6
Map29	F07	F82	Businesses	Lost invest	0.8
Map29	F07	F01	Businesses	Bad experi	0.8
Map29	F07	F64	Businesses	Increased p	0.8
Map29	F07	F96	Businesses	More hung	0.6
Map29	F07	F78	Businesses	Less food t	0.6
Map29	F20	F01	Difficult bre	Bad experi	0.2
Map29	F69	F01	Increased t	Bad experi	0.4
Map29	F69	F63	Increased t	Increased c	0.4
Map29	F69	F130	Increased t	Physical in	0.4

For Peer Review

Map29	F69	F38	Increased t	Health wor	0.4
Map29	F67	F01	Increased s	Bad experi	0.4
Map29	F29	F01	Fear of cor	Bad experi	0.6
Map29	F29	F63	Fear of cor	Increased c	0.6
Map29	F29	F08	Fear of cor	Cannot cor	0.6
Map29	F29	F124	Fear of cor	Not visiting	0.6
Map29	F29	F31	Fear of cor	Fear of eac	0.6
Map29	F29	F38	Fear of cor	Health wor	0.4
Map29	F29	F98	Fear of cor	More mate	0.6
Map29	F64	F01	Increased p	Bad experi	1
Map29	F64	F96	Increased p	More hung	0.6
Map29	F64	F78	Increased p	Less food t	0.6
Map29	F63	F01	Increased c	Bad experi	1
Map29	F63	F38	Increased c	Health wor	0.4
Map29	F08	F01	Cannot cor	Bad experi	0.6
Map29	F08	F98	Cannot cor	More mate	0.6
Map29	F60	F01	Increased i	Bad experi	0.4
Map29	F60	F69	Increased i	Increased t	0.4
Map29	F60	F67	Increased i	Increased s	0.6
Map29	F60	F63	Increased i	Increased c	0.4
Map29	F60	F130	Increased i	Physical in	0.4
Map29	F60	F38	Increased i	Health wor	0.4
Map29	F60	F65	Increased i	Increased r	0.4
Map29	F158	F01	Youth/child	Bad experi	0.4
Map29	F158	F69	Youth/child	Increased t	0.4
Map29	F158	F67	Youth/child	Increased s	0.6
Map29	F158	F63	Youth/child	Increased c	0.4
Map29	F158	F60	Youth/child	Increased i	0.6
Map29	F158	F130	Youth/child	Physical in	0.4
Map29	F158	F38	Youth/child	Health wor	0.4
Map29	F158	F65	Youth/child	Increased r	0.4
Map29	F138	F01	Restricted	Bad experi	0.8
Map29	F138	F135	Restricted	Price hike	0.8
Map29	F96	F01	More hung	Bad experi	0.8
Map29	F115	F01	No preachi	Bad experi	0.2
Map29	F115	F147	No preachi	Stress and	0.2
Map29	F130	F01	Physical in	Bad experi	0.4
Map29	F130	F63	Physical in	Increased c	0.4
Map29	F130	F38	Physical in	Health wor	0.4
Map29	F135	F01	Price hike	Bad experi	0.8
Map29	F124	F01	Not visiting	Bad experi	0.8
Map29	F124	F63	Not visiting	Increased c	0.8
Map29	F124	F08	Not visiting	Cannot cor	0.6
Map29	F124	F38	Not visiting	Health wor	0.4
Map29	F124	F98	Not visiting	More mate	0.6
Map29	F31	F01	Fear of eac	Bad experi	0.4
Map29	F31	F63	Fear of eac	Increased c	0.4
Map29	F31	F08	Fear of eac	Cannot cor	0.4
Map29	F31	F124	Fear of eac	Not visiting	0.4
Map29	F31	F38	Fear of eac	Health wor	0.4
Map29	F31	F98	Fear of eac	More mate	0.4
Map29	F78	F01	Less food t	Bad experi	0.6
Map29	F78	F96	Less food t	More hung	0.6
Map29	F10	F20	Compulsor	Difficult bre	0.4
Map29	F10	F01	Compulsor	Bad experi	0.4
Map29	F10	F63	Compulsor	Increased c	0.4

For Peer Review

Map29	F10	F38	Compulsor	Health wor	0.4
Map29	F10	F127	Compulsor	Penalty for	0.4
Map29	F10	F108	Compulsor	No entry at	0.4
Map29	F16	F01	Delay in ec	Bad experi	0.6
Map29	F98	F01	More mate	Bad experi	0.6
Map29	F142	F01	School/ma	Bad experi	0.6
Map29	F142	F69	School/ma	Increased t	0.4
Map29	F142	F67	School/ma	Increased s	0.6
Map29	F142	F63	School/ma	Increased c	0.4
Map29	F142	F60	School/ma	Increased i	0.6
Map29	F142	F158	School/ma	Youth/child	0.6
Map29	F142	F130	School/ma	Physical in	0.4
Map29	F142	F38	School/ma	Health wor	0.4
Map29	F142	F16	School/ma	Delay in ec	0.8
Map29	F142	F65	School/ma	Increased r	0.4
Map29	F127	F01	Penalty for	Bad experi	0.2
Map29	F147	F01	Stress and	Bad experi	0.8
Map29	F144	F01	Social dista	Bad experi	0.4
Map29	F144	F138	Social dista	Restricted	0.4
Map29	F144	F135	Social dista	Price hike	0.4
Map29	F108	F01	No entry at	Bad experi	0.6
Map29	F108	F63	No entry at	Increased c	0.6
Map29	F108	F38	No entry at	Health wor	0.4
Map29	F106	F01	No congreg	Bad experi	0.8
Map29	F106	F147	No congreg	Stress and	0.8
Map29	F65	F01	Increased r	Bad experi	0.2
Map29	F65	F63	Increased r	Increased c	0.2
Map29	F65	F130	Increased r	Physical in	0.2
Map29	F65	F38	Increased r	Health wor	0.2
Map29	F100	F01	Mosques/c	Bad experi	0.8
Map29	F100	F115	Mosques/c	No preachi	0.2
Map29	F100	F147	Mosques/c	Stress and	0.8
Map29	F100	F106	Mosques/c	No congreg	1
Map30	F40	F47	Household	Increase in	0.6
Map30	F40	F01	Household	Bad experi	0.6
Map30	F88	F40	Men unable	Household	0.6
Map30	F88	F47	Men unable	Increase in	0.6
Map30	F88	F01	Men unable	Bad experi	0.6
Map30	F47	F01	Increase in	Bad experi	0.6
Map30	F85	F40	Markets/sh	Household	0.6
Map30	F85	F88	Markets/sh	Men unable	0.6
Map30	F85	F47	Markets/sh	Increase in	0.6
Map30	F85	F82	Markets/sh	Lost invest	0.6
Map30	F85	F07	Markets/sh	Businesses	0.8
Map30	F85	F01	Markets/sh	Bad experi	0.6
Map30	F85	F64	Markets/sh	Increased p	0.6
Map30	F85	F96	Markets/sh	More hung	0.6
Map30	F85	F78	Markets/sh	Less food t	0.6
Map30	F85	F146	Markets/sh	Street haw	0.6
Map30	F87	F40	Men stayin	Household	0.6
Map30	F87	F88	Men stayin	Men unable	0.8
Map30	F87	F47	Men stayin	Increase in	0.6
Map30	F87	F01	Men stayin	Bad experi	0.6
Map30	F87	F98	Men stayin	More mate	0.4
Map30	F87	F99	Men stayin	More unwa	0.6
Map30	F80	F40	Lockdown	Household	0.6

For Peer Review

Map30	F80	F88	Lockdown	Men unable	0.8
Map30	F80	F47	Lockdown	Increase in	0.6
Map30	F80	F85	Lockdown	Markets/sh	1
Map30	F80	F87	Lockdown	Men stayin	0.8
Map30	F80	F82	Lockdown	Lost invest	0.6
Map30	F80	F07	Lockdown	Businesses	0.8
Map30	F80	F01	Lockdown	Bad experi	0.6
Map30	F80	F64	Lockdown	Increased p	0.6
Map30	F80	F63	Lockdown	Increased c	0.6
Map30	F80	F158	Lockdown	Youth/child	0.6
Map30	F80	F96	Lockdown	More hung	0.6
Map30	F80	F95	Lockdown	More hous	0.6
Map30	F80	F118	Lockdown	No social g	1
Map30	F80	F98	Lockdown	More mate	0.6
Map30	F80	F99	Lockdown	More unwa	0.6
Map30	F80	F32	Lockdown	Feeling soc	0.6
Map30	F80	F78	Lockdown	Less food t	0.6
Map30	F80	F115	Lockdown	No preachi	0.8
Map30	F80	F13	Lockdown	Could not c	0.6
Map30	F80	F120	Lockdown	No transpo	0.8
Map30	F80	F124	Lockdown	Not visiting	0.6
Map30	F80	F140	Lockdown	Restriction	0.6
Map30	F80	F147	Lockdown	Stress and	0.6
Map30	F80	F146	Lockdown	Street haw	0.6
Map30	F80	F144	Lockdown	Social dista	0.8
Map30	F80	F106	Lockdown	No congreg	0.8
Map30	F80	F100	Lockdown	Mosques/c	1
Map30	F82	F40	Lost invest	Household	0.6
Map30	F82	F88	Lost invest	Men unable	0.8
Map30	F82	F47	Lost invest	Increase in	0.6
Map30	F82	F01	Lost invest	Bad experi	0.8
Map30	F82	F64	Lost invest	Increased p	1
Map30	F82	F96	Lost invest	More hung	0.8
Map30	F82	F78	Lost invest	Less food t	0.8
Map30	F07	F40	Businesses	Household	0.6
Map30	F07	F88	Businesses	Men unable	0.6
Map30	F07	F47	Businesses	Increase in	0.6
Map30	F07	F82	Businesses	Lost invest	0.6
Map30	F07	F01	Businesses	Bad experi	0.6
Map30	F07	F64	Businesses	Increased p	0.6
Map30	F07	F96	Businesses	More hung	0.6
Map30	F07	F78	Businesses	Less food t	0.6
Map30	F07	F146	Businesses	Street haw	0.6
Map30	F20	F01	Difficult bre	Bad experi	0.2
Map30	F29	F01	Fear of cor	Bad experi	0.6
Map30	F29	F98	Fear of cor	More mate	0.6
Map30	F29	F38	Fear of cor	Health wor	0.6
Map30	F64	F40	Increased p	Household	0.6
Map30	F64	F88	Increased p	Men unable	0.8
Map30	F64	F47	Increased p	Increase in	0.6
Map30	F64	F01	Increased p	Bad experi	0.8
Map30	F64	F96	Increased p	More hung	0.8
Map30	F64	F78	Increased p	Less food t	0.8
Map30	F63	F01	Increased c	Bad experi	0.8
Map30	F158	F01	Youth/child	Bad experi	0.6
Map30	F158	F95	Youth/child	More hous	0.6

For Peer Review

Map30	F158	F13	Youth/child	Could not c	0.8
Map30	F158	F147	Youth/child	Stress and	0.6
Map30	F96	F01	More hung	Bad experi	1
Map30	F95	F01	More hous	Bad experi	0.6
Map30	F95	F147	More hous	Stress and	0.6
Map30	F118	F01	No social g	Bad experi	0.6
Map30	F118	F63	No social g	Increased c	0.6
Map30	F118	F98	No social g	More mate	0.6
Map30	F118	F120	No social g	No transpo	0.8
Map30	F118	F124	No social g	Not visiting	0.6
Map30	F98	F01	More mate	Bad experi	0.6
Map30	F99	F01	More unwa	Bad experi	0.4
Map30	F99	F98	More unwa	More mate	0.4
Map30	F32	F01	Feeling soc	Bad experi	0.4
Map30	F32	F147	Feeling soc	Stress and	0.4
Map30	F78	F01	Less food t	Bad experi	0.8
Map30	F78	F96	Less food t	More hung	0.8
Map30	F115	F01	No preachi	Bad experi	0.6
Map30	F115	F147	No preachi	Stress and	0.6
Map30	F38	F01	Health wor	Bad experi	0.6
Map30	F38	F98	Health wor	More mate	0.8
Map30	F13	F01	Could not c	Bad experi	0.6
Map30	F13	F147	Could not c	Stress and	0.6
Map30	F10	F20	Compulsor	Difficult bre	0.4
Map30	F10	F01	Compulsor	Bad experi	0.2
Map30	F120	F01	No transpo	Bad experi	0.6
Map30	F120	F63	No transpo	Increased c	0.6
Map30	F120	F98	No transpo	More mate	0.6
Map30	F120	F124	No transpo	Not visiting	0.6
Map30	F124	F01	Not visiting	Bad experi	0.6
Map30	F124	F63	Not visiting	Increased c	0.6
Map30	F124	F98	Not visiting	More mate	0.6
Map30	F140	F01	Restriction	Bad experi	0.4
Map30	F140	F32	Restriction	Feeling soc	0.6
Map30	F140	F147	Restriction	Stress and	0.4
Map30	F147	F01	Stress and	Bad experi	0.6
Map30	F146	F40	Street haw	Household	0.6
Map30	F146	F88	Street haw	Men unable	0.8
Map30	F146	F47	Street haw	Increase in	0.6
Map30	F146	F01	Street haw	Bad experi	0.8
Map30	F146	F64	Street haw	Increased p	1
Map30	F146	F96	Street haw	More hung	0.8
Map30	F146	F78	Street haw	Less food t	0.8
Map30	F144	F01	Social dista	Bad experi	0.4
Map30	F144	F32	Social dista	Feeling soc	0.6
Map30	F144	F140	Social dista	Restriction	0.6
Map30	F144	F147	Social dista	Stress and	0.4
Map30	F106	F01	No congreg	Bad experi	0.6
Map30	F106	F115	No congreg	No preachi	0.8
Map30	F106	F147	No congreg	Stress and	0.6
Map30	F100	F01	Mosques/c	Bad experi	0.6
Map30	F100	F115	Mosques/c	No preachi	0.8
Map30	F100	F147	Mosques/c	Stress and	0.6
Map30	F100	F106	Mosques/c	No congreg	0.8
Map31	F85	F83	Markets/sh	Malnutrition	0.6
Map31	F85	F01	Markets/sh	Bad experi	0.8

For Peer Review

Map31	F85	F64	Markets/sh	Increased (	0.8
Map31	F85	F63	Markets/sh	Increased (	0.6
Map31	F85	F62	Markets/sh	Increased r	0.6
Map31	F85	F152	Markets/sh	Unable to b	0.8
Map31	F85	F96	Markets/sh	More hung	0.8
Map31	F85	F110	Markets/sh	No funerals	0.6
Map31	F85	F112	Markets/sh	No home/p	1
Map31	F85	F78	Markets/sh	Less food t	0.8
Map31	F85	F14	Markets/sh	Could not g	0.6
Map31	F85	F147	Markets/sh	Stress and	0.6
Map31	F80	F85	Lockdown	Markets/sh	0.8
Map31	F80	F83	Lockdown	Malnutritior	0.6
Map31	F80	F06	Lockdown	Borders clc	1
Map31	F80	F01	Lockdown	Bad experi	0.8
Map31	F80	F64	Lockdown	Increased (	0.8
Map31	F80	F63	Lockdown	Increased (	0.6
Map31	F80	F62	Lockdown	Increased r	0.6
Map31	F80	F152	Lockdown	Unable to b	0.8
Map31	F80	F118	Lockdown	No social g	0.6
Map31	F80	F114	Lockdown	No pilgrima	0.2
Map31	F80	F96	Lockdown	More hung	0.8
Map31	F80	F110	Lockdown	No funerals	0.6
Map31	F80	F112	Lockdown	No home/p	0.8
Map31	F80	F32	Lockdown	Feeling soc	0.6
Map31	F80	F78	Lockdown	Less food t	0.8
Map31	F80	F14	Lockdown	Could not g	0.6
Map31	F80	F147	Lockdown	Stress and	0.8
Map31	F80	F144	Lockdown	Social dista	0.8
Map31	F80	F106	Lockdown	No congreg	0.8
Map31	F80	F100	Lockdown	Mosques/c	1
Map31	F83	F01	Malnutritior	Bad experi	0.8
Map31	F83	F63	Malnutritior	Increased (	0.4
Map31	F83	F62	Malnutritior	Increased r	0.4
Map31	F83	F110	Malnutritior	No funerals	0.4
Map31	F83	F147	Malnutritior	Stress and	0.4
Map31	F06	F01	Borders clc	Bad experi	0.2
Map31	F06	F62	Borders clc	Increased r	0.2
Map31	F06	F114	Borders clc	No pilgrima	0.2
Map31	F06	F147	Borders clc	Stress and	0.2
Map31	F29	F01	Fear of cor	Bad experi	0.6
Map31	F29	F30	Fear of cor	Fear of dea	0.8
Map31	F29	F31	Fear of cor	Fear of eac	0.6
Map31	F64	F83	Increased (	Malnutritior	0.6
Map31	F64	F01	Increased (	Bad experi	0.8
Map31	F64	F63	Increased (	Increased (	0.6
Map31	F64	F62	Increased (	Increased r	0.6
Map31	F64	F152	Increased (	Unable to b	0.8
Map31	F64	F96	Increased (	More hung	0.8
Map31	F64	F110	Increased (	No funerals	0.6
Map31	F64	F78	Increased (	Less food t	0.8
Map31	F64	F14	Increased (	Could not g	0.6
Map31	F64	F147	Increased (	Stress and	0.6
Map31	F63	F01	Increased (	Bad experi	0.8
Map31	F63	F62	Increased (	Increased r	0.8
Map31	F63	F110	Increased (	No funerals	0.8
Map31	F63	F147	Increased (	Stress and	0.6

For Peer Review

Map31	F62	F01	Increased r	Bad experi	0.8
Map31	F152	F01	Unable to b	Bad experi	0.6
Map31	F152	F63	Unable to b	Increased c	0.6
Map31	F152	F62	Unable to b	Increased r	0.6
Map31	F152	F110	Unable to b	No funerals	0.6
Map31	F152	F14	Unable to b	Could not g	0.6
Map31	F152	F147	Unable to b	Stress and	0.6
Map31	F118	F01	No social g	Bad experi	0.4
Map31	F118	F32	No social g	Feeling soc	0.6
Map31	F114	F01	No pilgrima	Bad experi	0.6
Map31	F114	F62	No pilgrima	Increased r	0.6
Map31	F114	F147	No pilgrima	Stress and	0.8
Map31	F96	F83	More hung	Malnutrition	0.6
Map31	F96	F01	More hung	Bad experi	1
Map31	F96	F63	More hung	Increased c	0.4
Map31	F96	F62	More hung	Increased r	0.4
Map31	F96	F110	More hung	No funerals	0.4
Map31	F96	F147	More hung	Stress and	0.4
Map31	F110	F01	No funerals	Bad experi	0.8
Map31	F110	F62	No funerals	Increased r	0.8
Map31	F110	F147	No funerals	Stress and	0.6
Map31	F111	F83	No governm	Malnutrition	0.4
Map31	F111	F01	No governm	Bad experi	0.4
Map31	F111	F64	No governm	Increased p	0.4
Map31	F111	F63	No governm	Increased c	0.4
Map31	F111	F62	No governm	Increased r	0.4
Map31	F111	F152	No governm	Unable to b	0.4
Map31	F111	F96	No governm	More hung	0.4
Map31	F111	F110	No governm	No funerals	0.4
Map31	F111	F78	No governm	Less food t	0.4
Map31	F111	F14	No governm	Could not g	0.4
Map31	F111	F147	No governm	Stress and	0.4
Map31	F112	F83	No home/p	Malnutrition	0.6
Map31	F112	F01	No home/p	Bad experi	0.8
Map31	F112	F64	No home/p	Increased p	0.8
Map31	F112	F63	No home/p	Increased c	0.6
Map31	F112	F62	No home/p	Increased r	0.6
Map31	F112	F152	No home/p	Unable to b	0.8
Map31	F112	F96	No home/p	More hung	0.8
Map31	F112	F110	No home/p	No funerals	0.6
Map31	F112	F78	No home/p	Less food t	0.8
Map31	F112	F14	No home/p	Could not g	0.6
Map31	F112	F147	No home/p	Stress and	0.6
Map31	F30	F01	Fear of dea	Bad experi	0.6
Map31	F31	F01	Fear of eac	Bad experi	0.4
Map31	F32	F01	Feeling soc	Bad experi	0.4
Map31	F78	F83	Less food t	Malnutrition	0.6
Map31	F78	F01	Less food t	Bad experi	0.8
Map31	F78	F63	Less food t	Increased c	0.4
Map31	F78	F62	Less food t	Increased r	0.4
Map31	F78	F96	Less food t	More hung	0.8
Map31	F78	F110	Less food t	No funerals	0.4
Map31	F78	F147	Less food t	Stress and	0.4
Map31	F71	F01	Lack of aw	Bad experi	0.6
Map31	F71	F29	Lack of aw	Fear of cor	0.8
Map31	F71	F30	Lack of aw	Fear of dea	0.8

For Peer Review

Map31	F71	F31	Lack of aw	Fear of eac	0.6
Map31	F71	F71	Lack of aw	Lack of aw	0.8
Map31	F14	F01	Could not g	Bad experi	0.6
Map31	F14	F63	Could not g	Increased d	0.6
Map31	F14	F62	Could not g	Increased r	0.6
Map31	F14	F110	Could not g	No funerals	0.6
Map31	F14	F147	Could not g	Stress and	0.6
Map31	F147	F01	Stress and	Bad experi	0.6
Map31	F147	F62	Stress and	Increased r	0.6
Map31	F144	F01	Social dista	Bad experi	0.4
Map31	F144	F118	Social dista	No social g	0.6
Map31	F144	F32	Social dista	Feeling soc	0.6
Map31	F106	F01	No congreg	Bad experi	0.6
Map31	F106	F62	No congreg	Increased r	0.6
Map31	F106	F147	No congreg	Stress and	1
Map31	F100	F01	Mosques/c	Bad experi	0.6
Map31	F100	F62	Mosques/c	Increased r	0.6
Map31	F100	F147	Mosques/c	Stress and	0.8
Map31	F100	F106	Mosques/c	No congreg	0.8
Map32	F40	F01	Household	Bad experi	0.6
Map32	F40	F155	Household	Wives ran a	0.6
Map32	F85	F40	Markets/sh	Household	0.6
Map32	F85	F83	Markets/sh	Malnutrition	0.6
Map32	F85	F82	Markets/sh	Lost invest	0.8
Map32	F85	F07	Markets/sh	Businesses	0.8
Map32	F85	F01	Markets/sh	Bad experi	0.8
Map32	F85	F64	Markets/sh	Increased p	0.8
Map32	F85	F155	Markets/sh	Wives ran a	0.6
Map32	F85	F96	Markets/sh	More hung	0.6
Map32	F85	F135	Markets/sh	Price hike	0.8
Map32	F85	F78	Markets/sh	Less food t	0.8
Map32	F85	F143	Markets/sh	Shortage o	0.8
Map32	F81	F01	Loss of Ha	Bad experi	0.6
Map32	F81	F63	Loss of Ha	Increased d	0.6
Map32	F81	F147	Loss of Ha	Stress and	0.6
Map32	F80	F40	Lockdown	Household	0.6
Map32	F80	F85	Lockdown	Markets/sh	0.6
Map32	F80	F81	Lockdown	Loss of Ha	0.8
Map32	F80	F83	Lockdown	Malnutrition	0.6
Map32	F80	F82	Lockdown	Lost invest	0.6
Map32	F80	F07	Lockdown	Businesses	0.6
Map32	F80	F01	Lockdown	Bad experi	0.6
Map32	F80	F64	Lockdown	Increased p	0.6
Map32	F80	F63	Lockdown	Increased d	0.6
Map32	F80	F155	Lockdown	Wives ran a	0.6
Map32	F80	F158	Lockdown	Youth/child	0.4
Map32	F80	F94	Lockdown	More hous	0.4
Map32	F80	F96	Lockdown	More hung	0.6
Map32	F80	F110	Lockdown	No funerals	0.6
Map32	F80	F135	Lockdown	Price hike	0.6
Map32	F80	F114	Lockdown	No pilgrima	1
Map32	F80	F78	Lockdown	Less food t	0.6
Map32	F80	F16	Lockdown	Delay in ed	0.4
Map32	F80	F143	Lockdown	Shortage o	0.6
Map32	F80	F142	Lockdown	School/mad	0.4
Map32	F80	F147	Lockdown	Stress and	0.6

For Peer Review

Map32	F80	F106	Lockdown	No congreg	1
Map32	F80	F100	Lockdown	Mosques/c	1
Map32	F83	F01	Malnutritior	Bad experi	1
Map32	F82	F01	Lost invest	Bad experi	1
Map32	F82	F64	Lost invest	Increased p	1
Map32	F07	F82	Businesses	Lost invest	1
Map32	F07	F01	Businesses	Bad experi	1
Map32	F07	F64	Businesses	Increased p	1
Map32	F20	F01	Difficult bre	Bad experi	0.4
Map32	F29	F20	Fear of cor	Difficult bre	0.2
Map32	F29	F01	Fear of cor	Bad experi	0.4
Map32	F29	F63	Fear of cor	Increased (	0.4
Map32	F29	F62	Fear of cor	Increased r	0.4
Map32	F29	F118	Fear of cor	No social g	0.4
Map32	F29	F32	Fear of cor	Feeling soc	0.4
Map32	F29	F10	Fear of cor	Compulsor	0.4
Map32	F29	F147	Fear of cor	Stress and	0.4
Map32	F29	F31	Fear of cor	Fear of eac	0.6
Map32	F29	F144	Fear of cor	Social dista	0.4
Map32	F29	F106	Fear of cor	No congreg	0.4
Map32	F64	F01	Increased p	Bad experi	1
Map32	F63	F01	Increased (	Bad experi	0.8
Map32	F62	F01	Increased r	Bad experi	1
Map32	F155	F01	Wives ran a	Bad experi	0.8
Map32	F158	F01	Youth/child	Bad experi	0.6
Map32	F158	F94	Youth/child	More hous	0.6
Map32	F158	F16	Youth/child	Delay in ec	0.4
Map32	F118	F01	No social g	Bad experi	0.8
Map32	F118	F63	No social g	Increased (	0.6
Map32	F118	F147	No social g	Stress and	0.8
Map32	F94	F01	More hous	Bad experi	0.8
Map32	F132	F01	Poor harve	Bad experi	0.8
Map32	F96	F83	More hung	Malnutritior	0.8
Map32	F96	F01	More hung	Bad experi	0.8
Map32	F110	F01	No funerals	Bad experi	0.8
Map32	F110	F63	No funerals	Increased (	0.6
Map32	F110	F147	No funerals	Stress and	0.8
Map32	F111	F01	No governr	Bad experi	0.8
Map32	F111	F132	No governr	Poor harve	0.8
Map32	F111	F101	No governr	No access	1
Map32	F135	F01	Price hike	Bad experi	1
Map32	F124	F01	Not visiting	Bad experi	0.8
Map32	F124	F63	Not visiting	Increased (	0.8
Map32	F114	F81	No pilgrima	Loss of Ha	0.8
Map32	F114	F01	No pilgrima	Bad experi	0.6
Map32	F114	F63	No pilgrima	Increased (	0.6
Map32	F114	F147	No pilgrima	Stress and	0.6
Map32	F32	F01	Feeling soc	Bad experi	0.6
Map32	F32	F62	Feeling soc	Increased r	0.6
Map32	F78	F40	Less food t	Household	0.6
Map32	F78	F83	Less food t	Malnutritior	0.6
Map32	F78	F01	Less food t	Bad experi	0.6
Map32	F78	F155	Less food t	Wives ran a	0.6
Map32	F78	F96	Less food t	More hung	0.6
Map32	F10	F20	Compulsor	Difficult bre	0.2
Map32	F10	F01	Compulsor	Bad experi	0.2

For Peer Review

Map32	F16	F01	Delay in ed	Bad experi	0.6
Map32	F143	F40	Shortage o	Household	0.6
Map32	F143	F83	Shortage o	Malnutritior	0.6
Map32	F143	F01	Shortage o	Bad experi	0.8
Map32	F143	F155	Shortage o	Wives ran a	0.6
Map32	F143	F96	Shortage o	More hung	0.6
Map32	F143	F135	Shortage o	Price hike	0.8
Map32	F143	F78	Shortage o	Less food t	0.8
Map32	F142	F01	School/ma	Bad experi	0.5
Map32	F142	F158	School/ma	Youth/child	0.5
Map32	F142	F94	School/ma	More hous	0.5
Map32	F142	F16	School/ma	Delay in ec	0.4
Map32	F147	F01	Stress and	Bad experi	1
Map32	F147	F63	Stress and	Increased (	0.6
Map32	F120	F40	No transpo	Household	0.6
Map32	F120	F83	No transpo	Malnutritior	0.6
Map32	F120	F01	No transpo	Bad experi	0.6
Map32	F120	F63	No transpo	Increased (	0.6
Map32	F120	F155	No transpo	Wives ran a	0.6
Map32	F120	F96	No transpo	More hung	0.6
Map32	F120	F135	No transpo	Price hike	0.6
Map32	F120	F124	No transpo	Not visiting	0.6
Map32	F120	F78	No transpo	Less food t	0.6
Map32	F120	F143	No transpo	Shortage o	0.6
Map32	F31	F20	Fear of eac	Difficult bre	0.2
Map32	F31	F01	Fear of eac	Bad experi	0.2
Map32	F31	F10	Fear of eac	Compulsor	0.4
Map32	F144	F01	Social dista	Bad experi	0.6
Map32	F144	F63	Social dista	Increased (	0.4
Map32	F144	F62	Social dista	Increased r	0.6
Map32	F144	F118	Social dista	No social g	0.4
Map32	F144	F32	Social dista	Feeling soc	0.6
Map32	F144	F147	Social dista	Stress and	0.4
Map32	F144	F106	Social dista	No congreg	0.8
Map32	F101	F01	No access	Bad experi	0.8
Map32	F101	F132	No access	Poor harve	0.8
Map32	F100	F106	Mosques/c	No congreg	1
Map33	F40	F47	Household	Increase in	0.4
Map33	F40	F01	Household	Bad experi	0.2
Map33	F47	F01	Increase in	Bad experi	0.2
Map33	F85	F40	Markets/sh	Household	0.6
Map33	F85	F47	Markets/sh	Increase in	0.4
Map33	F85	F87	Markets/sh	Men stayin	0.8
Map33	F85	F82	Markets/sh	Lost invest	0.8
Map33	F85	F01	Markets/sh	Bad experi	0.8
Map33	F85	F64	Markets/sh	Increased p	0.8
Map33	F85	F112	Markets/sh	No home/p	0.6
Map33	F87	F40	Men stayin	Household	0.6
Map33	F87	F47	Men stayin	Increase in	0.4
Map33	F87	F82	Men stayin	Lost invest	0.8
Map33	F87	F01	Men stayin	Bad experi	0.8
Map33	F87	F64	Men stayin	Increased p	0.8
Map33	F80	F40	Lockdown	Household	0.6
Map33	F80	F47	Lockdown	Increase in	0.4
Map33	F80	F85	Lockdown	Markets/sh	0.6
Map33	F80	F87	Lockdown	Men stayin	0.6

For Peer Review

Map33	F80	F82	Lockdown	Lost invest	0.6
Map33	F80	F01	Lockdown	Bad experi	0.8
Map33	F80	F64	Lockdown	Increased p	0.6
Map33	F80	F63	Lockdown	Increased c	0.6
Map33	F80	F158	Lockdown	Youth/child	0.8
Map33	F80	F118	Lockdown	No social g	0.6
Map33	F80	F96	Lockdown	More hung	0.6
Map33	F80	F130	Lockdown	Physical in	0.4
Map33	F80	F112	Lockdown	No home/p	0.6
Map33	F80	F135	Lockdown	Price hike	0.8
Map33	F80	F32	Lockdown	Feeling soc	0.6
Map33	F80	F78	Lockdown	Less food t	0.8
Map33	F80	F13	Lockdown	Could not c	0.6
Map33	F80	F16	Lockdown	Delay in ec	0.8
Map33	F80	F142	Lockdown	School/ma	0.8
Map33	F80	F140	Lockdown	Restriction	0.6
Map33	F80	F147	Lockdown	Stress and	0.8
Map33	F80	F106	Lockdown	No congreg	0.8
Map33	F80	F100	Lockdown	Mosques/c	0.8
Map33	F82	F01	Lost invest	Bad experi	0.8
Map33	F82	F64	Lost invest	Increased p	0.8
Map33	F20	F01	Difficult bre	Bad experi	0.4
Map33	F29	F20	Fear of cor	Difficult bre	0.2
Map33	F29	F01	Fear of cor	Bad experi	0.2
Map33	F29	F10	Fear of cor	Compulsor	0.4
Map33	F64	F01	Increased p	Bad experi	1
Map33	F63	F01	Increased c	Bad experi	0.4
Map33	F158	F01	Youth/child	Bad experi	0.8
Map33	F158	F16	Youth/child	Delay in ec	0.8
Map33	F118	F01	No social g	Bad experi	0.6
Map33	F118	F32	No social g	Feeling soc	0.8
Map33	F96	F01	More hung	Bad experi	0.4
Map33	F96	F63	More hung	Increased c	0.6
Map33	F130	F01	Physical in	Bad experi	0.6
Map33	F112	F82	No home/p	Lost invest	0.6
Map33	F112	F01	No home/p	Bad experi	0.6
Map33	F112	F64	No home/p	Increased p	0.6
Map33	F135	F01	Price hike	Bad experi	0.4
Map33	F135	F63	Price hike	Increased c	0.6
Map33	F135	F96	Price hike	More hung	0.6
Map33	F135	F78	Price hike	Less food t	0.8
Map33	F32	F01	Feeling soc	Bad experi	0.6
Map33	F78	F01	Less food t	Bad experi	0.4
Map33	F78	F63	Less food t	Increased c	0.6
Map33	F78	F96	Less food t	More hung	0.6
Map33	F13	F01	Could not c	Bad experi	0.4
Map33	F13	F130	Could not c	Physical in	0.4
Map33	F10	F20	Compulsor	Difficult bre	0.2
Map33	F10	F01	Compulsor	Bad experi	0.2
Map33	F16	F01	Delay in ec	Bad experi	0.8
Map33	F142	F01	School/ma	Bad experi	0.8
Map33	F142	F158	School/ma	Youth/child	0.8
Map33	F142	F130	School/ma	Physical in	0.4
Map33	F142	F13	School/ma	Could not c	0.6
Map33	F142	F16	School/ma	Delay in ec	0.8
Map33	F140	F01	Restriction	Bad experi	0.6

For Peer Review

Map33	F140	F118	Restriction	No social g	0.8
Map33	F140	F32	Restriction	Feeling soc	0.8
Map33	F147	F01	Stress and	Bad experi	1
Map33	F106	F01	No congreg	Bad experi	0.8
Map33	F106	F147	No congreg	Stress and	0.8
Map33	F100	F01	Mosques/c	Bad experi	0.8
Map33	F100	F147	Mosques/c	Stress and	0.8
Map33	F100	F106	Mosques/c	No congreg	0.8
Map34	F85	F07	Markets/sh	Businesses	0.8
Map34	F85	F01	Markets/sh	Bad experi	0.6
Map34	F85	F67	Markets/sh	Increased s	0.4
Map34	F85	F64	Markets/sh	Increased p	0.6
Map34	F85	F96	Markets/sh	More hung	0.6
Map34	F85	F131	Markets/sh	Physical pu	0.4
Map34	F85	F78	Markets/sh	Less food t	0.6
Map34	F87	F82	Men stayin	Lost invest	0.6
Map34	F87	F01	Men stayin	Bad experi	0.6
Map34	F87	F67	Men stayin	Increased s	0.4
Map34	F87	F64	Men stayin	Increased p	0.6
Map34	F87	F96	Men stayin	More hung	0.6
Map34	F87	F131	Men stayin	Physical pu	0.4
Map34	F87	F78	Men stayin	Less food t	0.6
Map34	F87	F70	Men stayin	Jobs lost	0.6
Map34	F80	F85	Lockdown	Markets/sh	1
Map34	F80	F87	Lockdown	Men stayin	0.8
Map34	F80	F82	Lockdown	Lost invest	0.6
Map34	F80	F07	Lockdown	Businesses	0.8
Map34	F80	F01	Lockdown	Bad experi	0.6
Map34	F80	F67	Lockdown	Increased s	0.4
Map34	F80	F64	Lockdown	Increased p	0.6
Map34	F80	F158	Lockdown	Youth/child	0.6
Map34	F80	F16	Lockdown	Delay in ec	0.6
Map34	F80	F118	Lockdown	No social g	0.6
Map34	F80	F96	Lockdown	More hung	0.6
Map34	F80	F131	Lockdown	Physical pu	0.4
Map34	F80	F51	Lockdown	Increase in	0.6
Map34	F80	F78	Lockdown	Less food t	0.6
Map34	F80	F121	Lockdown	No wedding	0.4
Map34	F80	F76	Lockdown	Less affect	0.6
Map34	F80	F70	Lockdown	Jobs lost	0.6
Map34	F80	F142	Lockdown	School/ma	1
Map34	F80	F140	Lockdown	Restriction	0.8
Map34	F80	F147	Lockdown	Stress and	0.6
Map34	F80	F120	Lockdown	No transpo	0.6
Map34	F80	F144	Lockdown	Social dista	0.6
Map34	F80	F106	Lockdown	No congreg	0.6
Map34	F80	F100	Lockdown	Mosques/c	0.6
Map34	F82	F01	Lost invest	Bad experi	0.6
Map34	F82	F67	Lost invest	Increased s	0.4
Map34	F82	F64	Lost invest	Increased p	1
Map34	F82	F96	Lost invest	More hung	0.6
Map34	F82	F131	Lost invest	Physical pu	0.4
Map34	F82	F78	Lost invest	Less food t	0.8
Map34	F07	F01	Businesses	Bad experi	0.6
Map34	F07	F67	Businesses	Increased s	0.4
Map34	F07	F64	Businesses	Increased p	0.6

For Peer Review

Map34	F07	F96	Businesses	More hung	0.6
Map34	F07	F131	Businesses	Physical pu	0.4
Map34	F07	F78	Businesses	Less food t	0.6
Map34	F20	F01	Difficult bre	Bad experi	0.6
Map34	F20	F63	Difficult bre	Increased (	1
Map34	F67	F01	Increased s	Bad experi	0.2
Map34	F67	F131	Increased s	Physical pu	0.8
Map34	F29	F20	Fear of cor	Difficult bre	0.4
Map34	F29	F01	Fear of cor	Bad experi	0.6
Map34	F29	F63	Fear of cor	Increased (	0.6
Map34	F29	F154	Fear of cor	Wearing fa	0.8
Map34	F29	F118	Fear of cor	No social g	0.6
Map34	F29	F124	Fear of cor	Not visiting	0.6
Map34	F29	F31	Fear of cor	Fear of eac	0.6
Map34	F29	F38	Fear of cor	Health wor	0.6
Map34	F29	F121	Fear of cor	No wedding	0.4
Map34	F29	F76	Fear of cor	Less affect	0.6
Map34	F29	F147	Fear of cor	Stress and	0.4
Map34	F64	F01	Increased p	Bad experi	0.6
Map34	F64	F67	Increased p	Increased s	0.4
Map34	F64	F96	Increased p	More hung	0.6
Map34	F64	F131	Increased p	Physical pu	0.4
Map34	F64	F78	Increased p	Less food t	0.8
Map34	F63	F01	Increased (	Bad experi	0.6
Map34	F154	F20	Wearing fa	Difficult bre	0.4
Map34	F154	F01	Wearing fa	Bad experi	0.4
Map34	F154	F63	Wearing fa	Increased (	0.4
Map34	F158	F82	Youth/child	Lost invest	0.6
Map34	F158	F01	Youth/child	Bad experi	0.6
Map34	F158	F67	Youth/child	Increased s	0.4
Map34	F158	F64	Youth/child	Increased p	0.8
Map34	F158	F16	Youth/child	Delay in ec	0.6
Map34	F158	F96	Youth/child	More hung	0.6
Map34	F158	F131	Youth/child	Physical pu	0.4
Map34	F158	F51	Youth/child	Increase in	0.6
Map34	F158	F78	Youth/child	Less food t	0.8
Map34	F158	F70	Youth/child	Jobs lost	0.8
Map34	F16	F01	Delay in ec	Bad experi	0.8
Map34	F16	F51	Delay in ec	Increase in	0.8
Map34	F118	F01	No social g	Bad experi	0.8
Map34	F118	F121	No social g	No wedding	0.4
Map34	F118	F76	No social g	Less affect	0.8
Map34	F118	F147	No social g	Stress and	0.4
Map34	F96	F01	More hung	Bad experi	1
Map34	F131	F01	Physical pu	Bad experi	0.2
Map34	F51	F01	Increase in	Bad experi	0.8
Map34	F124	F01	Not visiting	Bad experi	0.6
Map34	F124	F63	Not visiting	Increased (	0.8
Map34	F31	F01	Fear of eac	Bad experi	0.6
Map34	F31	F63	Fear of eac	Increased (	0.8
Map34	F31	F124	Fear of eac	Not visiting	0.8
Map34	F31	F38	Fear of eac	Health wor	0.6
Map34	F78	F01	Less food t	Bad experi	0.6
Map34	F78	F96	Less food t	More hung	0.6
Map34	F38	F01	Health wor	Bad experi	0.6
Map34	F38	F63	Health wor	Increased (	0.8

For Peer Review

Map34	F121	F01	No weddin	Bad experi	0.6
Map34	F121	F147	No weddin	Stress and	0.6
Map34	F76	F01	Less affect	Bad experi	0.8
Map34	F70	F82	Jobs lost	Lost invest	0.6
Map34	F70	F01	Jobs lost	Bad experi	0.6
Map34	F70	F67	Jobs lost	Increased s	0.4
Map34	F70	F64	Jobs lost	Increased p	0.8
Map34	F70	F96	Jobs lost	More hung	0.6
Map34	F70	F131	Jobs lost	Physical pu	0.4
Map34	F70	F78	Jobs lost	Less food t	0.8
Map34	F142	F82	School/ma	Lost invest	0.6
Map34	F142	F01	School/ma	Bad experi	0.6
Map34	F142	F67	School/ma	Increased s	0.4
Map34	F142	F64	School/ma	Increased p	0.6
Map34	F142	F158	School/ma	Youth/child	0.6
Map34	F142	F16	School/ma	Delay in ed	0.6
Map34	F142	F118	School/ma	No social g	0.6
Map34	F142	F96	School/ma	More hung	0.6
Map34	F142	F131	School/ma	Physical pu	0.4
Map34	F142	F51	School/ma	Increase in	0.6
Map34	F142	F78	School/ma	Less food t	0.6
Map34	F142	F121	School/ma	No weddin	0.4
Map34	F142	F76	School/ma	Less affect	0.6
Map34	F142	F70	School/ma	Jobs lost	0.6
Map34	F142	F147	School/ma	Stress and	0.6
Map34	F142	F144	School/ma	Social dista	0.6
Map34	F142	F106	School/ma	No congreg	0.6
Map34	F142	F100	School/ma	Mosques/c	0.6
Map34	F140	F87	Restriction	Men stayin	0.8
Map34	F140	F82	Restriction	Lost invest	0.6
Map34	F140	F01	Restriction	Bad experi	0.6
Map34	F140	F67	Restriction	Increased s	0.4
Map34	F140	F64	Restriction	Increased p	0.6
Map34	F140	F96	Restriction	More hung	0.6
Map34	F140	F131	Restriction	Physical pu	0.4
Map34	F140	F78	Restriction	Less food t	0.6
Map34	F140	F70	Restriction	Jobs lost	0.6
Map34	F147	F01	Stress and	Bad experi	1
Map34	F120	F01	No transpo	Bad experi	0.6
Map34	F120	F118	No transpo	No social g	0.6
Map34	F120	F121	No transpo	No weddin	0.4
Map34	F120	F76	No transpo	Less affect	0.6
Map34	F120	F147	No transpo	Stress and	0.4
Map34	F144	F01	Social dista	Bad experi	0.8
Map34	F144	F118	Social dista	No social g	0.6
Map34	F144	F121	Social dista	No weddin	0.4
Map34	F144	F76	Social dista	Less affect	0.6
Map34	F144	F147	Social dista	Stress and	0.8
Map34	F144	F106	Social dista	No congreg	0.8
Map34	F144	F100	Social dista	Mosques/c	0.8
Map34	F108	F20	No entry at	Difficult bre	0.4
Map34	F108	F01	No entry at	Bad experi	0.4
Map34	F108	F63	No entry at	Increased c	0.4
Map34	F108	F154	No entry at	Wearing fa	1
Map34	F106	F01	No congreg	Bad experi	0.8
Map34	F106	F147	No congreg	Stress and	0.8

For Peer Review

Map34	F100	F01	Mosques/c	Bad experi	0.8
Map34	F100	F118	Mosques/c	No social g	0.6
Map34	F100	F121	Mosques/c	No weddin	0.4
Map34	F100	F76	Mosques/c	Less affect	0.6
Map34	F100	F147	Mosques/c	Stress and	0.8
Map34	F100	F106	Mosques/c	No congreg	1
Map35	F40	F47	Household	Increase in	0.4
Map35	F40	F01	Household	Bad experi	0.4
Map35	F88	F40	Men unabl	Household	0.6
Map35	F88	F47	Men unabl	Increase in	0.4
Map35	F88	F01	Men unabl	Bad experi	0.6
Map35	F88	F95	Men unabl	More hous	0.8
Map35	F47	F01	Increase in	Bad experi	0.4
Map35	F85	F40	Markets/sh	Household	0.6
Map35	F85	F88	Markets/sh	Men unabl	0.8
Map35	F85	F47	Markets/sh	Increase in	0.4
Map35	F85	F87	Markets/sh	Men stayin	0.8
Map35	F85	F82	Markets/sh	Lost invest	0.6
Map35	F85	F07	Markets/sh	Businesses	0.8
Map35	F85	F01	Markets/sh	Bad experi	0.6
Map35	F85	F64	Markets/sh	Increased	0.6
Map35	F85	F63	Markets/sh	Increased	0.6
Map35	F85	F95	Markets/sh	More hous	0.8
Map35	F85	F96	Markets/sh	More hung	0.6
Map35	F85	F135	Markets/sh	Price hike	0.6
Map35	F85	F78	Markets/sh	Less food t	0.6
Map35	F85	F143	Markets/sh	Shortage o	0.6
Map35	F87	F40	Men stayin	Household	0.6
Map35	F87	F88	Men stayin	Men unabl	0.8
Map35	F87	F47	Men stayin	Increase in	0.4
Map35	F87	F01	Men stayin	Bad experi	0.6
Map35	F87	F95	Men stayin	More hous	0.8
Map35	F80	F40	Lockdown	Household	0.6
Map35	F80	F88	Lockdown	Men unabl	0.8
Map35	F80	F47	Lockdown	Increase in	0.4
Map35	F80	F85	Lockdown	Markets/sh	0.8
Map35	F80	F87	Lockdown	Men stayin	1
Map35	F80	F82	Lockdown	Lost invest	0.6
Map35	F80	F07	Lockdown	Businesses	0.8
Map35	F80	F01	Lockdown	Bad experi	0.8
Map35	F80	F64	Lockdown	Increased	0.6
Map35	F80	F63	Lockdown	Increased	0.6
Map35	F80	F157	Lockdown	Women un	0.6
Map35	F80	F158	Lockdown	Youth/child	0.8
Map35	F80	F118	Lockdown	No social g	0.6
Map35	F80	F95	Lockdown	More hous	0.8
Map35	F80	F96	Lockdown	More hung	0.8
Map35	F80	F135	Lockdown	Price hike	0.8
Map35	F80	F32	Lockdown	Feeling soc	0.4
Map35	F80	F78	Lockdown	Less food t	0.8
Map35	F80	F37	Lockdown	Health facil	0.8
Map35	F80	F38	Lockdown	Health wor	0.6
Map35	F80	F13	Lockdown	Could not c	0.6
Map35	F80	F143	Lockdown	Shortage o	0.8
Map35	F80	F142	Lockdown	School/ma	1
Map35	F80	F147	Lockdown	Stress and	0.4

For Peer Review

Map35	F80	F120	Lockdown	No transpo	0.8
Map35	F82	F01	Lost invest	Bad experi	0.6
Map35	F82	F64	Lost invest	Increased p	0.6
Map35	F82	F63	Lost invest	Increased c	0.6
Map35	F82	F96	Lost invest	More hung	0.6
Map35	F07	F82	Businesses	Lost invest	0.6
Map35	F07	F01	Businesses	Bad experi	0.6
Map35	F07	F64	Businesses	Increased p	0.6
Map35	F07	F63	Businesses	Increased c	0.6
Map35	F07	F96	Businesses	More hung	0.6
Map35	F07	F135	Businesses	Price hike	0.6
Map35	F07	F78	Businesses	Less food t	0.6
Map35	F07	F143	Businesses	Shortage o	0.6
Map35	F20	F01	Difficult bre	Bad experi	0.2
Map35	F29	F01	Fear of cor	Bad experi	0.4
Map35	F29	F31	Fear of cor	Fear of eac	0.6
Map35	F64	F01	Increased p	Bad experi	0.8
Map35	F64	F63	Increased p	Increased c	0.6
Map35	F64	F96	Increased p	More hung	0.8
Map35	F63	F01	Increased c	Bad experi	0.4
Map35	F157	F01	Women un	Bad experi	0.4
Map35	F157	F118	Women un	No social g	0.6
Map35	F157	F32	Women un	Feeling soc	0.4
Map35	F158	F01	Youth/child	Bad experi	0.4
Map35	F158	F13	Youth/child	Could not c	0.6
Map35	F158	F147	Youth/child	Stress and	0.4
Map35	F118	F01	No social g	Bad experi	0.4
Map35	F118	F32	No social g	Feeling soc	0.4
Map35	F95	F01	More hous	Bad experi	0.6
Map35	F96	F01	More hung	Bad experi	1
Map35	F96	F63	More hung	Increased c	0.6
Map35	F135	F01	Price hike	Bad experi	0.8
Map35	F135	F63	Price hike	Increased c	0.6
Map35	F135	F96	Price hike	More hung	0.8
Map35	F135	F78	Price hike	Less food t	0.8
Map35	F31	F01	Fear of eac	Bad experi	0.4
Map35	F32	F01	Feeling soc	Bad experi	0.4
Map35	F78	F01	Less food t	Bad experi	0.8
Map35	F78	F63	Less food t	Increased c	0.6
Map35	F78	F96	Less food t	More hung	0.8
Map35	F37	F01	Health facil	Bad experi	0.4
Map35	F37	F63	Health facil	Increased c	0.4
Map35	F37	F38	Health facil	Health wor	0.6
Map35	F38	F01	Health wor	Bad experi	0.4
Map35	F38	F63	Health wor	Increased c	0.4
Map35	F13	F01	Could not c	Bad experi	0.4
Map35	F13	F147	Could not c	Stress and	0.4
Map35	F10	F20	Compulsor	Difficult bre	0.4
Map35	F10	F01	Compulsor	Bad experi	0.2
Map35	F143	F01	Shortage o	Bad experi	0.8
Map35	F143	F63	Shortage o	Increased c	0.6
Map35	F143	F96	Shortage o	More hung	0.8
Map35	F143	F135	Shortage o	Price hike	1
Map35	F143	F78	Shortage o	Less food t	0.8
Map35	F142	F01	School/ma	Bad experi	0.4
Map35	F142	F158	School/ma	Youth/child	0.8

For Peer Review

Map35	F142	F13	School/ma	Could not c	0.6
Map35	F142	F147	School/ma	Stress and	0.4
Map35	F147	F01	Stress and	Bad experi	0.4
Map35	F120	F01	No transpo	Bad experi	0.8
Map35	F120	F63	No transpo	Increased c	0.6
Map35	F120	F157	No transpo	Women un	0.6
Map35	F120	F118	No transpo	No social g	0.6
Map35	F120	F96	No transpo	More hung	0.8
Map35	F120	F135	No transpo	Price hike	0.8
Map35	F120	F32	No transpo	Feeling soc	0.4
Map35	F120	F78	No transpo	Less food t	0.8
Map35	F120	F37	No transpo	Health facil	0.8
Map35	F120	F38	No transpo	Health wor	0.6
Map35	F120	F143	No transpo	Shortage o	0.8
Map35	F144	F01	Social dista	Bad experi	0.4
Map35	F144	F118	Social dista	No social g	0.6
Map35	F144	F32	Social dista	Feeling soc	0.4
Map36	F40	F47	Household	Increase in	0.2
Map36	F40	F01	Household	Bad experi	0.2
Map36	F88	F40	Men unable	Household	0.6
Map36	F88	F47	Men unable	Increase in	0.2
Map36	F88	F01	Men unable	Bad experi	0.2
Map36	F47	F01	Increase in	Bad experi	0.2
Map36	F85	F40	Markets/sh	Household	0.6
Map36	F85	F88	Markets/sh	Men unable	0.8
Map36	F85	F47	Markets/sh	Increase in	0.2
Map36	F85	F87	Markets/sh	Men stayin	0.8
Map36	F85	F82	Markets/sh	Lost invest	0.6
Map36	F85	F07	Markets/sh	Businesses	0.8
Map36	F85	F01	Markets/sh	Bad experi	0.8
Map36	F85	F64	Markets/sh	Increased p	0.6
Map36	F85	F96	Markets/sh	More hung	0.8
Map36	F85	F135	Markets/sh	Price hike	0.8
Map36	F85	F143	Markets/sh	Shortage o	0.8
Map36	F87	F40	Men stayin	Household	0.6
Map36	F87	F88	Men stayin	Men unable	1
Map36	F87	F47	Men stayin	Increase in	0.2
Map36	F87	F01	Men stayin	Bad experi	0.2
Map36	F80	F40	Lockdown	Household	0.6
Map36	F80	F88	Lockdown	Men unable	0.8
Map36	F80	F47	Lockdown	Increase in	0.2
Map36	F80	F85	Lockdown	Markets/sh	1
Map36	F80	F87	Lockdown	Men stayin	0.8
Map36	F80	F82	Lockdown	Lost invest	0.6
Map36	F80	F07	Lockdown	Businesses	0.8
Map36	F80	F01	Lockdown	Bad experi	0.8
Map36	F80	F64	Lockdown	Increased p	0.6
Map36	F80	F63	Lockdown	Increased c	0.6
Map36	F80	F158	Lockdown	Youth/child	1
Map36	F80	F70	Lockdown	Jobs lost	1
Map36	F80	F96	Lockdown	More hung	0.8
Map36	F80	F133	Lockdown	Poor perfor	0.6
Map36	F80	F135	Lockdown	Price hike	0.8
Map36	F80	F37	Lockdown	Health facil	0.6
Map36	F80	F38	Lockdown	Health wor	0.6
Map36	F80	F13	Lockdown	Could not c	0.6

For Peer Review

Map36	F80	F16	Lockdown	Delay in ec	1
Map36	F80	F143	Lockdown	Shortage o	0.8
Map36	F80	F142	Lockdown	School/mac	1
Map36	F80	F147	Lockdown	Stress and	0.6
Map36	F80	F120	Lockdown	No transpo	0.6
Map36	F82	F01	Lost invest	Bad experi	0.8
Map36	F82	F64	Lost invest	Increased p	0.8
Map36	F82	F96	Lost invest	More hung	0.6
Map36	F07	F40	Businesses	Household	0.6
Map36	F07	F88	Businesses	Men unable	1
Map36	F07	F47	Businesses	Increase in	0.2
Map36	F07	F87	Businesses	Men stayin	1
Map36	F07	F82	Businesses	Lost invest	0.6
Map36	F07	F01	Businesses	Bad experi	0.6
Map36	F07	F64	Businesses	Increased p	0.6
Map36	F07	F96	Businesses	More hung	0.6
Map36	F20	F01	Difficult bre	Bad experi	0.2
Map36	F29	F01	Fear of cor	Bad experi	0.4
Map36	F29	F31	Fear of cor	Fear of eac	0.6
Map36	F64	F01	Increased p	Bad experi	0.8
Map36	F64	F96	Increased p	More hung	0.6
Map36	F63	F01	Increased c	Bad experi	1
Map36	F158	F01	Youth/child	Bad experi	0.6
Map36	F158	F13	Youth/child	Could not c	0.6
Map36	F158	F147	Youth/child	Stress and	0.6
Map36	F70	F40	Jobs lost	Household	0.4
Map36	F70	F88	Jobs lost	Men unable	0.4
Map36	F70	F47	Jobs lost	Increase in	0.2
Map36	F70	F87	Jobs lost	Men stayin	0.4
Map36	F70	F01	Jobs lost	Bad experi	0.2
Map36	F96	F01	More hung	Bad experi	1
Map36	F133	F01	Poor perfor	Bad experi	0.2
Map36	F133	F147	Poor perfor	Stress and	0.2
Map36	F135	F01	Price hike	Bad experi	0.8
Map36	F135	F96	Price hike	More hung	0.8
Map36	F31	F01	Fear of eac	Bad experi	0.4
Map36	F32	F01	Feeling soc	Bad experi	0.4
Map36	F118	F01	No social g	Bad experi	0.4
Map36	F118	F32	No social g	Feeling soc	0.4
Map36	F37	F01	Health facil	Bad experi	0.8
Map36	F37	F63	Health facil	Increased c	0.8
Map36	F37	F38	Health facil	Health wor	0.8
Map36	F38	F01	Health wor	Bad experi	0.8
Map36	F38	F63	Health wor	Increased c	0.8
Map36	F13	F01	Could not c	Bad experi	0.6
Map36	F13	F147	Could not c	Stress and	0.6
Map36	F10	F20	Compulsor	Difficult bre	0.4
Map36	F10	F01	Compulsor	Bad experi	0.2
Map36	F16	F01	Delay in ec	Bad experi	0.2
Map36	F16	F133	Delay in ec	Poor perfor	0.6
Map36	F16	F147	Delay in ec	Stress and	0.2
Map36	F143	F01	Shortage o	Bad experi	0.8
Map36	F143	F96	Shortage o	More hung	0.8
Map36	F143	F135	Shortage o	Price hike	1
Map36	F142	F01	School/mac	Bad experi	0.6
Map36	F142	F158	School/mac	Youth/child	1

For Peer Review

Map36	F142	F133	School/ma	Poor perfo	0.6
Map36	F142	F13	School/ma	Could not	0.6
Map36	F142	F16	School/ma	Delay in ec	1
Map36	F142	F147	School/ma	Stress and	0.6
Map36	F147	F01	Stress and	Bad experi	1
Map36	F120	F01	No transpo	Bad experi	0.6
Map36	F120	F63	No transpo	Increased	0.6
Map36	F120	F37	No transpo	Health facil	0.6
Map36	F120	F38	No transpo	Health wor	0.6
Map36	F144	F01	Social dista	Bad experi	0.4
Map36	F144	F32	Social dista	Feeling soc	0.4
Map36	F144	F118	Social dista	No social g	0.6
Map37	F40	F47	Household	Increase in	0.6
Map37	F40	F01	Household	Bad experi	0.6
Map37	F88	F40	Men unable	Household	0.6
Map37	F88	F47	Men unable	Increase in	0.6
Map37	F88	F01	Men unable	Bad experi	0.6
Map37	F47	F01	Increase in	Bad experi	0.4
Map37	F85	F40	Markets/sh	Household	0.6
Map37	F85	F88	Markets/sh	Men unable	0.8
Map37	F85	F47	Markets/sh	Increase in	0.6
Map37	F85	F83	Markets/sh	Malnutrition	0.6
Map37	F85	F82	Markets/sh	Lost invest	0.6
Map37	F85	F07	Markets/sh	Businesses	0.8
Map37	F85	F01	Markets/sh	Bad experi	0.8
Map37	F85	F64	Markets/sh	Increased	0.6
Map37	F85	F96	Markets/sh	More hung	0.8
Map37	F85	F132	Markets/sh	Poor harve	0.8
Map37	F85	F135	Markets/sh	Price hike	0.8
Map37	F85	F78	Markets/sh	Less food t	0.8
Map37	F85	F70	Markets/sh	Jobs lost	0.8
Map37	F85	F143	Markets/sh	Shortage o	0.8
Map37	F85	F147	Markets/sh	Stress and	0.8
Map37	F85	F101	Markets/sh	No access	1
Map37	F80	F40	Lockdown	Household	0.6
Map37	F80	F88	Lockdown	Men unable	0.8
Map37	F80	F47	Lockdown	Increase in	0.6
Map37	F80	F85	Lockdown	Markets/sh	0.8
Map37	F80	F83	Lockdown	Malnutrition	0.6
Map37	F80	F82	Lockdown	Lost invest	0.6
Map37	F80	F07	Lockdown	Businesses	0.8
Map37	F80	F01	Lockdown	Bad experi	0.8
Map37	F80	F67	Lockdown	Increased s	0.4
Map37	F80	F64	Lockdown	Increased	0.6
Map37	F80	F60	Lockdown	Increased i	0.6
Map37	F80	F158	Lockdown	Youth/child	0.6
Map37	F80	F16	Lockdown	Delay in ec	0.6
Map37	F80	F96	Lockdown	More hung	0.8
Map37	F80	F132	Lockdown	Poor harve	0.8
Map37	F80	F130	Lockdown	Physical in	0.4
Map37	F80	F135	Lockdown	Price hike	0.8
Map37	F80	F114	Lockdown	No pilgrima	0.4
Map37	F80	F78	Lockdown	Less food t	0.8
Map37	F80	F70	Lockdown	Jobs lost	0.8
Map37	F80	F143	Lockdown	Shortage o	0.8
Map37	F80	F142	Lockdown	School/ma	1

For Peer Review

Map37	F80	F147	Lockdown	Stress and	0.8
Map37	F80	F144	Lockdown	Social dista	0.6
Map37	F80	F106	Lockdown	No congreg	0.8
Map37	F80	F101	Lockdown	No access	0.8
Map37	F80	F100	Lockdown	Mosques/c	0.8
Map37	F83	F01	Malnutritior	Bad experi	0.8
Map37	F82	F01	Lost invest	Bad experi	0.8
Map37	F82	F64	Lost invest	Increased p	0.8
Map37	F07	F40	Businesses	Household	0.6
Map37	F07	F88	Businesses	Men unable	0.8
Map37	F07	F47	Businesses	Increase in	0.6
Map37	F07	F82	Businesses	Lost invest	0.6
Map37	F07	F01	Businesses	Bad experi	0.6
Map37	F07	F64	Businesses	Increased p	0.6
Map37	F07	F70	Businesses	Jobs lost	0.8
Map37	F20	F01	Difficult bre	Bad experi	0.4
Map37	F67	F01	Increased s	Bad experi	0.6
Map37	F67	F147	Increased s	Stress and	0.6
Map37	F29	F20	Fear of cor	Difficult bre	0.4
Map37	F29	F01	Fear of cor	Bad experi	0.8
Map37	F29	F63	Fear of cor	Increased c	0.8
Map37	F29	F38	Fear of cor	Health wor	0.8
Map37	F29	F10	Fear of cor	Compulsor	0.4
Map37	F29	F31	Fear of cor	Fear of eac	0.4
Map37	F64	F01	Increased p	Bad experi	0.8
Map37	F63	F01	Increased c	Bad experi	1
Map37	F60	F01	Increased i	Bad experi	0.4
Map37	F60	F67	Increased i	Increased s	0.4
Map37	F60	F130	Increased i	Physical in	0.4
Map37	F60	F147	Increased i	Stress and	0.4
Map37	F158	F01	Youth/child	Bad experi	0.4
Map37	F158	F67	Youth/child	Increased s	0.4
Map37	F158	F60	Youth/child	Increased i	0.6
Map37	F158	F16	Youth/child	Delay in ec	0.8
Map37	F158	F130	Youth/child	Physical in	0.4
Map37	F158	F147	Youth/child	Stress and	0.4
Map37	F16	F01	Delay in ec	Bad experi	0.4
Map37	F16	F67	Delay in ec	Increased s	0.4
Map37	F16	F60	Delay in ec	Increased i	0.6
Map37	F16	F130	Delay in ec	Physical in	0.4
Map37	F16	F147	Delay in ec	Stress and	0.4
Map37	F96	F83	More hung	Malnutritior	0.6
Map37	F96	F01	More hung	Bad experi	0.8
Map37	F132	F83	Poor harve	Malnutritior	0.6
Map37	F132	F01	Poor harve	Bad experi	0.8
Map37	F132	F96	Poor harve	More hung	1
Map37	F132	F78	Poor harve	Less food t	1
Map37	F130	F01	Physical in	Bad experi	0.6
Map37	F130	F147	Physical in	Stress and	0.6
Map37	F135	F83	Price hike	Malnutritior	0.6
Map37	F135	F01	Price hike	Bad experi	1
Map37	F135	F96	Price hike	More hung	0.8
Map37	F135	F78	Price hike	Less food t	0.8
Map37	F135	F147	Price hike	Stress and	0.8
Map37	F114	F01	No pilgrima	Bad experi	0.2
Map37	F114	F147	No pilgrima	Stress and	0.2

For Peer Review

Map37	F78	F83	Less food t	Malnutritior	0.6
Map37	F78	F01	Less food t	Bad experi	0.8
Map37	F78	F96	Less food t	More hung	1
Map37	F38	F01	Health wor	Bad experi	0.8
Map37	F38	F63	Health wor	Increased (	0.8
Map37	F10	F20	Compulsor	Difficult bre	0.4
Map37	F10	F01	Compulsor	Bad experi	0.4
Map37	F70	F40	Jobs lost	Household	0.6
Map37	F70	F88	Jobs lost	Men unable	0.8
Map37	F70	F47	Jobs lost	Increase in	0.6
Map37	F70	F01	Jobs lost	Bad experi	0.6
Map37	F143	F83	Shortage o	Malnutritior	0.6
Map37	F143	F01	Shortage o	Bad experi	0.8
Map37	F143	F96	Shortage o	More hung	0.8
Map37	F143	F135	Shortage o	Price hike	0.8
Map37	F143	F78	Shortage o	Less food t	0.8
Map37	F143	F147	Shortage o	Stress and	0.8
Map37	F142	F01	School/ma	Bad experi	0.4
Map37	F142	F67	School/ma	Increased s	0.4
Map37	F142	F60	School/ma	Increased i	0.6
Map37	F142	F158	School/ma	Youth/child	0.6
Map37	F142	F16	School/ma	Delay in ec	0.6
Map37	F142	F130	School/ma	Physical in	0.4
Map37	F142	F147	School/ma	Stress and	0.4
Map37	F147	F01	Stress and	Bad experi	1
Map37	F31	F20	Fear of eac	Difficult bre	0.4
Map37	F31	F01	Fear of eac	Bad experi	0.4
Map37	F31	F10	Fear of eac	Compulsor	0.6
Map37	F144	F83	Social dista	Malnutritior	0.6
Map37	F144	F01	Social dista	Bad experi	0.6
Map37	F144	F96	Social dista	More hung	0.6
Map37	F144	F135	Social dista	Price hike	0.6
Map37	F144	F78	Social dista	Less food t	0.6
Map37	F144	F147	Social dista	Stress and	0.6
Map37	F106	F01	No congreg	Bad experi	1
Map37	F106	F147	No congreg	Stress and	1
Map37	F101	F83	No access	Malnutritior	0.6
Map37	F101	F01	No access	Bad experi	0.8
Map37	F101	F96	No access	More hung	0.8
Map37	F101	F132	No access	Poor harve	0.8
Map37	F101	F78	No access	Less food t	0.8
Map37	F100	F01	Mosques/c	Bad experi	1
Map37	F100	F147	Mosques/c	Stress and	1
Map37	F100	F106	Mosques/c	No congreg	1
Map38	F40	F47	Household	Increase in	0.6
Map38	F40	F01	Household	Bad experi	0.6
Map38	F88	F40	Men unable	Household	0.6
Map38	F88	F47	Men unable	Increase in	0.6
Map38	F88	F01	Men unable	Bad experi	0.6
Map38	F88	F95	Men unable	More hous	0.8
Map38	F47	F01	Increase in	Bad experi	0.6
Map38	F85	F40	Markets/sh	Household	0.6
Map38	F85	F88	Markets/sh	Men unable	0.6
Map38	F85	F47	Markets/sh	Increase in	0.6
Map38	F85	F87	Markets/sh	Men stayin	0.8
Map38	F85	F82	Markets/sh	Lost invest	0.6

For Peer Review

Map38	F85	F07	Markets/sh	Businesses	0.8
Map38	F85	F01	Markets/sh	Bad experi	0.6
Map38	F85	F64	Markets/sh	Increased p	0.6
Map38	F85	F08	Markets/sh	Cannot cor	0.4
Map38	F85	F96	Markets/sh	More hung	0.6
Map38	F85	F95	Markets/sh	More hous	0.6
Map38	F85	F98	Markets/sh	More mate	0.4
Map38	F87	F40	Men stayin	Household	0.6
Map38	F87	F88	Men stayin	Men unabl	0.6
Map38	F87	F47	Men stayin	Increase in	0.6
Map38	F87	F01	Men stayin	Bad experi	0.6
Map38	F87	F95	Men stayin	More hous	0.6
Map38	F80	F40	Lockdown	Household	0.6
Map38	F80	F88	Lockdown	Men unabl	0.6
Map38	F80	F47	Lockdown	Increase in	0.6
Map38	F80	F85	Lockdown	Markets/sh	0.8
Map38	F80	F87	Lockdown	Men stayin	0.8
Map38	F80	F82	Lockdown	Lost invest	0.6
Map38	F80	F07	Lockdown	Businesses	0.8
Map38	F80	F01	Lockdown	Bad experi	0.6
Map38	F80	F69	Lockdown	Increased t	0.6
Map38	F80	F67	Lockdown	Increased s	0.6
Map38	F80	F64	Lockdown	Increased p	0.6
Map38	F80	F63	Lockdown	Increased c	0.6
Map38	F80	F08	Lockdown	Cannot cor	0.6
Map38	F80	F60	Lockdown	Increased i	0.6
Map38	F80	F158	Lockdown	Youth/child	0.8
Map38	F80	F96	Lockdown	More hung	0.6
Map38	F80	F94	Lockdown	More hous	0.4
Map38	F80	F95	Lockdown	More hous	0.6
Map38	F80	F98	Lockdown	More mate	0.6
Map38	F80	F135	Lockdown	Price hike	0.6
Map38	F80	F124	Lockdown	Not visiting	0.8
Map38	F80	F58	Lockdown	Increased c	0.6
Map38	F80	F133	Lockdown	Poor perfor	0.4
Map38	F80	F13	Lockdown	Could not c	0.6
Map38	F80	F16	Lockdown	Delay in ec	0.6
Map38	F80	F14	Lockdown	Could not g	0.8
Map38	F80	F143	Lockdown	Shortage o	0.6
Map38	F80	F142	Lockdown	School/ma	1
Map38	F80	F147	Lockdown	Stress and	0.4
Map38	F80	F120	Lockdown	No transpo	0.8
Map38	F80	F115	Lockdown	No preachi	0.6
Map38	F80	F106	Lockdown	No congreg	0.8
Map38	F80	F100	Lockdown	Mosques/c	1
Map38	F82	F01	Lost invest	Bad experi	0.6
Map38	F82	F64	Lost invest	Increased p	0.6
Map38	F82	F08	Lost invest	Cannot cor	0.4
Map38	F82	F96	Lost invest	More hung	0.6
Map38	F82	F98	Lost invest	More mate	0.4
Map38	F07	F82	Businesses	Lost invest	0.6
Map38	F07	F01	Businesses	Bad experi	0.6
Map38	F07	F64	Businesses	Increased p	0.6
Map38	F07	F08	Businesses	Cannot cor	0.4
Map38	F07	F96	Businesses	More hung	0.6
Map38	F07	F98	Businesses	More mate	0.4

For Peer Review

Map38	F20	F01	Difficult bre	Bad experi	0.2
Map38	F69	F01	Increased t	Bad experi	0.4
Map38	F67	F01	Increased s	Bad experi	0.6
Map38	F29	F20	Fear of cor	Difficult bre	0.6
Map38	F29	F01	Fear of cor	Bad experi	0.4
Map38	F29	F31	Fear of cor	Fear of eac	0.6
Map38	F29	F74	Fear of cor	Lack of fac	0.4
Map38	F29	F10	Fear of cor	Compulsor	0.6
Map38	F64	F01	Increased p	Bad experi	0.8
Map38	F64	F08	Increased p	Cannot cor	0.4
Map38	F64	F96	Increased p	More hung	0.6
Map38	F64	F98	Increased p	More mate	0.4
Map38	F63	F01	Increased c	Bad experi	0.6
Map38	F08	F01	Cannot cor	Bad experi	0.6
Map38	F08	F98	Cannot cor	More mate	0.6
Map38	F60	F01	Increased i	Bad experi	0.6
Map38	F60	F69	Increased i	Increased t	0.6
Map38	F60	F67	Increased i	Increased s	0.8
Map38	F60	F58	Increased i	Increased c	0.8
Map38	F158	F01	Youth/child	Bad experi	0.6
Map38	F158	F69	Youth/child	Increased t	0.6
Map38	F158	F67	Youth/child	Increased s	0.6
Map38	F158	F60	Youth/child	Increased i	0.6
Map38	F158	F94	Youth/child	More hous	0.4
Map38	F158	F58	Youth/child	Increased c	0.6
Map38	F158	F133	Youth/child	Poor perfor	0.4
Map38	F158	F13	Youth/child	Could not c	0.6
Map38	F158	F16	Youth/child	Delay in ec	0.6
Map38	F96	F01	More hung	Bad experi	1
Map38	F95	F40	More hous	Household	0.6
Map38	F95	F47	More hous	Increase in	0.6
Map38	F95	F01	More hous	Bad experi	0.6
Map38	F118	F01	No social g	Bad experi	0.4
Map38	F118	F32	No social g	Feeling soc	0.6
Map38	F98	F01	More mate	Bad experi	0.6
Map38	F135	F01	Price hike	Bad experi	0.4
Map38	F135	F96	Price hike	More hung	0.4
Map38	F124	F01	Not visiting	Bad experi	0.6
Map38	F124	F63	Not visiting	Increased c	0.6
Map38	F124	F08	Not visiting	Cannot cor	0.6
Map38	F124	F98	Not visiting	More mate	0.6
Map38	F124	F14	Not visiting	Could not g	0.8
Map38	F58	F01	Increased c	Bad experi	0.6
Map38	F58	F69	Increased c	Increased t	0.6
Map38	F31	F01	Fear of eac	Bad experi	0.4
Map38	F32	F01	Feeling soc	Bad experi	0.4
Map38	F133	F01	Poor perfor	Bad experi	0.4
Map38	F74	F01	Lack of fac	Bad experi	0.4
Map38	F13	F01	Could not c	Bad experi	0.6
Map38	F13	F69	Could not c	Increased t	0.6
Map38	F13	F67	Could not c	Increased s	0.6
Map38	F13	F60	Could not c	Increased i	0.6
Map38	F13	F94	Could not c	More hous	0.4
Map38	F13	F58	Could not c	Increased c	0.6
Map38	F10	F20	Compulsor	Difficult bre	0.6
Map38	F10	F01	Compulsor	Bad experi	0.4

For Peer Review

Map38	F10	F74	Compulsor	Lack of fac	0.4
Map38	F16	F01	Delay in ed	Bad experi	0.4
Map38	F16	F133	Delay in ed	Poor perfor	0.4
Map38	F14	F01	Could not g	Bad experi	0.6
Map38	F14	F63	Could not g	Increased c	0.6
Map38	F143	F01	Shortage o	Bad experi	0.4
Map38	F143	F96	Shortage o	More hung	0.4
Map38	F143	F135	Shortage o	Price hike	0.6
Map38	F142	F01	School/ma	Bad experi	0.6
Map38	F142	F69	School/ma	Increased t	0.6
Map38	F142	F67	School/ma	Increased s	0.6
Map38	F142	F60	School/ma	Increased i	0.6
Map38	F142	F158	School/ma	Youth/child	0.8
Map38	F142	F94	School/ma	More hous	0.4
Map38	F142	F58	School/ma	Increased c	0.6
Map38	F142	F133	School/ma	Poor perfor	0.4
Map38	F142	F13	School/ma	Could not c	0.6
Map38	F142	F16	School/ma	Delay in ed	0.6
Map38	F147	F01	Stress and	Bad experi	0.6
Map38	F120	F01	No transpo	Bad experi	0.6
Map38	F120	F63	No transpo	Increased c	0.6
Map38	F120	F08	No transpo	Cannot cor	0.6
Map38	F120	F96	No transpo	More hung	0.4
Map38	F120	F98	No transpo	More mate	0.6
Map38	F120	F135	No transpo	Price hike	0.6
Map38	F120	F124	No transpo	Not visiting	0.8
Map38	F120	F14	No transpo	Could not g	0.8
Map38	F120	F143	No transpo	Shortage o	0.6
Map38	F144	F01	Social dista	Bad experi	0.4
Map38	F144	F118	Social dista	No social g	0.6
Map38	F144	F32	Social dista	Feeling soc	0.6
Map38	F144	F147	Social dista	Stress and	0.4
Map38	F144	F115	Social dista	No preachi	0.6
Map38	F144	F106	Social dista	No congreg	0.8
Map38	F144	F100	Social dista	Mosques/c	0.8
Map38	F115	F01	No preachi	Bad experi	0.4
Map38	F115	F147	No preachi	Stress and	0.4
Map38	F106	F01	No congreg	Bad experi	0.4
Map38	F106	F147	No congreg	Stress and	0.4
Map38	F106	F115	No congreg	No preachi	0.6
Map38	F100	F01	Mosques/c	Bad experi	0.4
Map38	F100	F147	Mosques/c	Stress and	0.4
Map38	F100	F115	Mosques/c	No preachi	0.6
Map38	F100	F106	Mosques/c	No congreg	0.8
Map39	F40	F47	Household	Increase in	1
Map39	F40	F01	Household	Bad experi	0.6
Map39	F47	F01	Increase in	Bad experi	0.6
Map39	F85	F82	Markets/sh	Lost invest	0.8
Map39	F85	F07	Markets/sh	Businesses	0.8
Map39	F85	F01	Markets/sh	Bad experi	0.8
Map39	F85	F64	Markets/sh	Increased p	0.8
Map39	F81	F01	Loss of Ha	Bad experi	0.6
Map39	F81	F147	Loss of Ha	Stress and	0.6
Map39	F80	F85	Lockdown	Markets/sh	0.8
Map39	F80	F81	Lockdown	Loss of Ha	0.4
Map39	F80	F82	Lockdown	Lost invest	0.8

For Peer Review

Map39	F80	F07	Lockdown	Businesses	0.8
Map39	F80	F01	Lockdown	Bad experi	0.8
Map39	F80	F64	Lockdown	Increased p	0.8
Map39	F80	F62	Lockdown	Increased r	0.4
Map39	F80	F158	Lockdown	Youth/child	1
Map39	F80	F114	Lockdown	No pilgrima	0.6
Map39	F80	F133	Lockdown	Poor perfor	0.8
Map39	F80	F130	Lockdown	Physical in	0.6
Map39	F80	F135	Lockdown	Price hike	0.6
Map39	F80	F51	Lockdown	Increase in	0.8
Map39	F80	F32	Lockdown	Feeling soc	0.4
Map39	F80	F13	Lockdown	Could not c	0.4
Map39	F80	F16	Lockdown	Delay in ec	0.8
Map39	F80	F143	Lockdown	Shortage o	0.6
Map39	F80	F142	Lockdown	School/mac	0.8
Map39	F80	F140	Lockdown	Restriction	0.8
Map39	F80	F147	Lockdown	Stress and	0.8
Map39	F80	F120	Lockdown	No transpo	0.8
Map39	F80	F144	Lockdown	Social dista	0.6
Map39	F80	F106	Lockdown	No congreg	1
Map39	F80	F100	Lockdown	Mosques/c	1
Map39	F82	F01	Lost invest	Bad experi	0.8
Map39	F82	F64	Lost invest	Increased p	0.8
Map39	F07	F82	Businesses	Lost invest	0.8
Map39	F07	F01	Businesses	Bad experi	0.8
Map39	F07	F64	Businesses	Increased p	0.8
Map39	F20	F01	Difficult bre	Bad experi	0.4
Map39	F29	F20	Fear of cor	Difficult bre	0.2
Map39	F29	F01	Fear of cor	Bad experi	0.4
Map39	F29	F62	Fear of cor	Increased r	0.4
Map39	F29	F31	Fear of cor	Fear of eac	0.6
Map39	F29	F32	Fear of cor	Feeling soc	0.4
Map39	F29	F10	Fear of cor	Compulsor	0.8
Map39	F29	F144	Fear of cor	Social dista	0.6
Map39	F64	F01	Increased p	Bad experi	1
Map39	F63	F01	Increased c	Bad experi	1
Map39	F62	F01	Increased r	Bad experi	1
Map39	F158	F01	Youth/child	Bad experi	0.4
Map39	F158	F130	Youth/child	Physical in	0.6
Map39	F158	F13	Youth/child	Could not c	0.4
Map39	F96	F40	More hung	Household	1
Map39	F96	F47	More hung	Increase in	1
Map39	F96	F01	More hung	Bad experi	0.8
Map39	F96	F63	More hung	Increased c	0.8
Map39	F114	F81	No pilgrima	Loss of Ha	0.4
Map39	F114	F01	No pilgrima	Bad experi	0.4
Map39	F114	F147	No pilgrima	Stress and	0.4
Map39	F133	F01	Poor perfor	Bad experi	0.6
Map39	F130	F01	Physical in	Bad experi	0.4
Map39	F130	F13	Physical in	Could not c	0.4
Map39	F135	F01	Price hike	Bad experi	1
Map39	F51	F01	Increase in	Bad experi	0.6
Map39	F31	F20	Fear of eac	Difficult bre	0.2
Map39	F31	F01	Fear of eac	Bad experi	0.2
Map39	F31	F10	Fear of eac	Compulsor	0.4
Map39	F32	F01	Feeling soc	Bad experi	0.6

For Peer Review

Map39	F32	F62	Feeling soc	Increased r	0.6
Map39	F78	F40	Less food t	Household	0.8
Map39	F78	F47	Less food t	Increase in	0.8
Map39	F78	F01	Less food t	Bad experi	0.6
Map39	F78	F63	Less food t	Increased c	0.6
Map39	F78	F96	Less food t	More hunge	0.6
Map39	F13	F01	Could not c	Bad experi	0.4
Map39	F10	F20	Compulsor	Difficult bre	0.2
Map39	F10	F01	Compulsor	Bad experi	0.2
Map39	F16	F01	Delay in ed	Bad experi	0.6
Map39	F16	F133	Delay in ed	Poor perfor	1
Map39	F16	F51	Delay in ed	Increase in	0.8
Map39	F143	F01	Shortage o	Bad experi	0.8
Map39	F143	F135	Shortage o	Price hike	0.8
Map39	F142	F01	School/mad	Bad experi	1
Map39	F142	F158	School/mad	Youth/child	1
Map39	F142	F133	School/mad	Poor perfor	0.8
Map39	F142	F130	School/mad	Physical inj	0.6
Map39	F142	F51	School/mad	Increase in	0.8
Map39	F142	F13	School/mad	Could not c	0.4
Map39	F142	F16	School/mad	Delay in ed	0.8
Map39	F142	F147	School/mad	Stress and	1
Map39	F140	F01	Restriction	Bad experi	0.6
Map39	F140	F140	Restriction	Restriction	1
Map39	F140	F147	Restriction	Stress and	0.6
Map39	F147	F01	Stress and	Bad experi	1
Map39	F120	F85	No transpo	Markets/sh	1
Map39	F120	F82	No transpo	Lost invest	0.8
Map39	F120	F07	No transpo	Businesses	0.8
Map39	F120	F01	No transpo	Bad experi	0.8
Map39	F120	F64	No transpo	Increased p	0.8
Map39	F120	F135	No transpo	Price hike	0.6
Map39	F120	F143	No transpo	Shortage o	0.6
Map39	F144	F01	Social dista	Bad experi	0.4
Map39	F144	F62	Social dista	Increased r	0.4
Map39	F144	F32	Social dista	Feeling soc	0.4
Map39	F106	F01	No congreg	Bad experi	0.4
Map39	F106	F62	No congreg	Increased r	0.4
Map39	F106	F32	No congreg	Feeling soc	0.4
Map39	F106	F144	No congreg	Social dista	0.6
Map39	F100	F01	Mosques/c	Bad experi	0.4
Map39	F100	F62	Mosques/c	Increased r	0.4
Map39	F100	F32	Mosques/c	Feeling soc	0.4
Map39	F100	F144	Mosques/c	Social dista	0.4
Map40	F20	F01	Difficult bre	Bad experi	0.4
Map40	F69	F67	Increased t	Increased s	0.6
Map40	F69	F130	Increased t	Physical inj	0.6
Map40	F69	F01	Increased t	Bad experi	0.4
Map40	F69	F65	Increased t	Increased r	0.4
Map40	F69	F58	Increased t	Increased c	0.4
Map40	F24	F01	Domestic v	Bad experi	0.4
Map40	F67	F01	Increased s	Bad experi	0.4
Map40	F29	F63	Fear of cor	Increased c	0.4
Map40	F29	F38	Fear of cor	Health wor	0.4
Map40	F29	F01	Fear of cor	Bad experi	0.4
Map40	F29	F03	Fear of cor	Afraid to vis	0.4

For Peer Review

Map40	F64	F01	Increased p	Bad experi	0.6
Map40	F63	F01	Increased c	Bad experi	0.8
Map40	F62	F01	Increased r	Bad experi	0.4
Map40	F139	F63	Restricted	Increased c	0.4
Map40	F139	F136	Restricted	Reduced p	0.4
Map40	F139	F01	Restricted	Bad experi	0.4
Map40	F96	F01	More hung	Bad experi	0.8
Map40	F133	F01	Poor perfor	Bad experi	0.4
Map40	F130	F01	Physical inj	Bad experi	0.4
Map40	F136	F63	Reduced p	Increased c	0.4
Map40	F136	F01	Reduced p	Bad experi	0.4
Map40	F135	F96	Price hike	More hung	0.6
Map40	F135	F78	Price hike	Less food t	0.6
Map40	F135	F01	Price hike	Bad experi	0.6
Map40	F31	F29	Fear of eac	Fear of cor	0.8
Map40	F31	F63	Fear of eac	Increased c	0.4
Map40	F31	F38	Fear of eac	Health wor	0.4
Map40	F31	F01	Fear of eac	Bad experi	0.4
Map40	F31	F03	Fear of eac	Afraid to vi	0.4
Map40	F31	F145	Fear of eac	Stigma aga	0.6
Map40	F32	F62	Feeling soc	Increased r	0.6
Map40	F32	F01	Feeling soc	Bad experi	0.4
Map40	F78	F96	Less food t	More hung	0.8
Map40	F78	F01	Less food t	Bad experi	0.8
Map40	F38	F01	Health wor	Bad experi	0.6
Map40	F73	F63	Lack of del	Increased c	0.6
Map40	F73	F01	Lack of del	Bad experi	0.6
Map40	F120	F63	No transpo	Increased c	0.6
Map40	F120	F96	No transpo	More hung	0.6
Map40	F120	F135	No transpo	Price hike	0.6
Map40	F120	F78	No transpo	Less food t	0.6
Map40	F120	F73	No transpo	Lack of del	0.8
Map40	F120	F01	No transpo	Bad experi	0.6
Map40	F120	F143	No transpo	Shortage o	0.6
Map40	F40	F24	Household	Domestic v	0.4
Map40	F40	F01	Household	Bad experi	0.4
Map40	F88	F24	Men unable	Domestic v	0.4
Map40	F88	F40	Men unable	Household	0.4
Map40	F88	F01	Men unable	Bad experi	0.4
Map40	F85	F24	Markets/sh	Domestic v	0.4
Map40	F85	F64	Markets/sh	Increased p	0.8
Map40	F85	F40	Markets/sh	Household	0.4
Map40	F85	F88	Markets/sh	Men unable	0.6
Map40	F85	F87	Markets/sh	Men stayin	0.8
Map40	F85	F82	Markets/sh	Lost invest	0.8
Map40	F85	F07	Markets/sh	Businesses	0.8
Map40	F85	F01	Markets/sh	Bad experi	0.6
Map40	F87	F24	Men stayin	Domestic v	0.4
Map40	F87	F40	Men stayin	Household	0.4
Map40	F87	F88	Men stayin	Men unable	0.6
Map40	F87	F01	Men stayin	Bad experi	0.4
Map40	F80	F69	Lockdown	Increased t	0.6
Map40	F80	F24	Lockdown	Domestic v	0.4
Map40	F80	F67	Lockdown	Increased s	0.6
Map40	F80	F64	Lockdown	Increased p	0.8
Map40	F80	F63	Lockdown	Increased c	0.6

For Peer Review

Map40	F80	F96	Lockdown	More hung	0.6
Map40	F80	F133	Lockdown	Poor perfor	0.4
Map40	F80	F130	Lockdown	Physical in	0.6
Map40	F80	F135	Lockdown	Price hike	0.6
Map40	F80	F78	Lockdown	Less food t	0.6
Map40	F80	F73	Lockdown	Lack of del	0.6
Map40	F80	F120	Lockdown	No transpo	0.6
Map40	F80	F40	Lockdown	Household	0.4
Map40	F80	F88	Lockdown	Men unable	0.6
Map40	F80	F85	Lockdown	Markets/sh	1
Map40	F80	F87	Lockdown	Men stayin	0.8
Map40	F80	F82	Lockdown	Lost invest	0.8
Map40	F80	F07	Lockdown	Businesses	0.8
Map40	F80	F01	Lockdown	Bad experi	0.6
Map40	F80	F65	Lockdown	Increased r	0.4
Map40	F80	F158	Lockdown	Youth/child	0.8
Map40	F80	F115	Lockdown	No preachi	0.8
Map40	F80	F55	Lockdown	Increased c	0.4
Map40	F80	F58	Lockdown	Increased c	0.4
Map40	F80	F13	Lockdown	Could not c	0.4
Map40	F80	F16	Lockdown	Delay in ed	0.4
Map40	F80	F143	Lockdown	Shortage o	0.6
Map40	F80	F142	Lockdown	School/mac	1
Map40	F80	F147	Lockdown	Stress and	0.6
Map40	F80	F106	Lockdown	No congreg	0.8
Map40	F80	F100	Lockdown	Mosques/c	0.8
Map40	F82	F64	Lost invest	Increased p	0.8
Map40	F82	F01	Lost invest	Bad experi	0.6
Map40	F07	F24	Businesses	Domestic v	0.4
Map40	F07	F64	Businesses	Increased p	0.8
Map40	F07	F40	Businesses	Household	0.4
Map40	F07	F88	Businesses	Men unable	0.6
Map40	F07	F87	Businesses	Men stayin	0.8
Map40	F07	F82	Businesses	Lost invest	0.8
Map40	F07	F01	Businesses	Bad experi	0.6
Map40	F03	F63	Afraid to vi	Increased c	0.6
Map40	F03	F01	Afraid to vi	Bad experi	0.6
Map40	F02	F63	Bad experi	Increased c	0.6
Map40	F02	F01	Bad experi	Bad experi	0.6
Map40	F02	F03	Bad experi	Afraid to vi	0.8
Map40	F65	F01	Increased r	Bad experi	0.2
Map40	F158	F133	Youth/child	Poor perfor	0.4
Map40	F158	F130	Youth/child	Physical in	0.4
Map40	F158	F01	Youth/child	Bad experi	0.4
Map40	F158	F55	Youth/child	Increased c	0.4
Map40	F158	F13	Youth/child	Could not c	0.4
Map40	F158	F16	Youth/child	Delay in ed	0.4
Map40	F118	F62	No social g	Increased r	0.6
Map40	F118	F32	No social g	Feeling soc	0.8
Map40	F118	F01	No social g	Bad experi	0.4
Map40	F115	F01	No preachi	Bad experi	0.6
Map40	F115	F147	No preachi	Stress and	0.6
Map40	F55	F01	Increased c	Bad experi	0.2
Map40	F58	F01	Increased c	Bad experi	0.4
Map40	F13	F130	Could not c	Physical in	0.4
Map40	F13	F01	Could not c	Bad experi	0.4

For Peer Review

Map40	F10	F20	Compulsor	Difficult bre	0.2
Map40	F10	F01	Compulsor	Bad experi	0.2
Map40	F16	F133	Delay in ec	Poor perfor	0.4
Map40	F16	F01	Delay in ec	Bad experi	0.4
Map40	F143	F96	Shortage o	More hung	0.6
Map40	F143	F135	Shortage o	Price hike	0.6
Map40	F143	F78	Shortage o	Less food t	0.6
Map40	F143	F01	Shortage o	Bad experi	0.6
Map40	F142	F69	School/ma	Increased t	0.6
Map40	F142	F67	School/ma	Increased s	0.6
Map40	F142	F133	School/ma	Poor perfor	0.4
Map40	F142	F130	School/ma	Physical in	0.6
Map40	F142	F01	School/ma	Bad experi	0.4
Map40	F142	F65	School/ma	Increased r	0.4
Map40	F142	F158	School/ma	Youth/child	0.8
Map40	F142	F55	School/ma	Increased c	0.4
Map40	F142	F58	School/ma	Increased c	0.4
Map40	F142	F13	School/ma	Could not c	0.4
Map40	F142	F16	School/ma	Delay in ec	0.4
Map40	F141	F29	Rumors on	Fear of cor	0.8
Map40	F141	F63	Rumors on	Increased c	0.4
Map40	F141	F38	Rumors on	Health wor	0.4
Map40	F141	F01	Rumors on	Bad experi	0.4
Map40	F141	F03	Rumors on	Afraid to vi	0.4
Map40	F147	F01	Stress and	Bad experi	1
Map40	F145	F01	Stigma aga	Bad experi	0.4
Map40	F144	F62	Social dista	Increased r	0.6
Map40	F144	F32	Social dista	Feeling soc	0.8
Map40	F144	F01	Social dista	Bad experi	0.4
Map40	F144	F118	Social dista	No social g	0.8
Map40	F106	F01	No congreg	Bad experi	0.6
Map40	F106	F147	No congreg	Stress and	0.6
Map40	F100	F01	Mosques/c	Bad experi	0.6
Map40	F100	F115	Mosques/c	No preachi	0.8
Map40	F100	F147	Mosques/c	Stress and	0.6
Map40	F100	F106	Mosques/c	No congreg	0.8
Map41	F85	F01	Markets/sh	Bad experi	0.6
Map41	F85	F96	Markets/sh	More hung	0.6
Map41	F85	F116	Markets/sh	No product	0.4
Map41	F85	F78	Markets/sh	Less food t	0.6
Map41	F81	F01	Loss of Ha	Bad experi	0.2
Map41	F80	F85	Lockdown	Markets/sh	0.8
Map41	F80	F81	Lockdown	Loss of Ha	0.2
Map41	F80	F06	Lockdown	Borders clo	0.6
Map41	F80	F01	Lockdown	Bad experi	0.6
Map41	F80	F64	Lockdown	Increased p	0.6
Map41	F80	F63	Lockdown	Increased c	0.4
Map41	F80	F60	Lockdown	Increased i	0.6
Map41	F80	F158	Lockdown	Youth/child	0.4
Map41	F80	F97	Lockdown	More impris	0.6
Map41	F80	F114	Lockdown	No pilgrima	0.4
Map41	F80	F96	Lockdown	More hung	0.6
Map41	F80	F116	Lockdown	No product	0.8
Map41	F80	F53	Lockdown	Increased s	0.6
Map41	F80	F58	Lockdown	Increased c	0.6
Map41	F80	F78	Lockdown	Less food t	0.8

For Peer Review

Map41	F80	F16	Lockdown	Delay in ec	0.4
Map41	F80	F17	Lockdown	Delay in jus	0.4
Map41	F80	F142	Lockdown	School/mad	0.8
Map41	F80	F140	Lockdown	Restriction	0.8
Map41	F80	F147	Lockdown	Stress and	0.4
Map41	F80	F120	Lockdown	No transpo	0.6
Map41	F80	F123	Lockdown	Not going t	0.8
Map41	F80	F128	Lockdown	People fee	0.6
Map41	F80	F106	Lockdown	No congreg	0.6
Map41	F80	F100	Lockdown	Mosques/c	0.8
Map41	F05	F01	Banks clos	Bad experi	0.4
Map41	F05	F63	Banks clos	Increased c	0.6
Map41	F05	F147	Banks clos	Stress and	0.8
Map41	F06	F81	Borders clo	Loss of Ha	0.2
Map41	F06	F01	Borders clo	Bad experi	0.2
Map41	F06	F114	Borders clo	No pilgrima	0.4
Map41	F29	F01	Fear of cor	Bad experi	0.4
Map41	F29	F63	Fear of cor	Increased c	0.6
Map41	F29	F10	Fear of cor	Compulsor	0.6
Map41	F29	F141	Fear of cor	Rumors on	0.8
Map41	F29	F147	Fear of cor	Stress and	0.6
Map41	F64	F01	Increased p	Bad experi	0.8
Map41	F64	F96	Increased p	More hung	0.8
Map41	F63	F01	Increased c	Bad experi	0.4
Map41	F60	F01	Increased i	Bad experi	0.2
Map41	F60	F97	Increased i	More impris	0.8
Map41	F60	F53	Increased i	Increased s	0.8
Map41	F60	F58	Increased i	Increased c	0.6
Map41	F60	F17	Increased i	Delay in jus	0.4
Map41	F158	F01	Youth/child	Bad experi	0.6
Map41	F158	F16	Youth/child	Delay in ec	0.6
Map41	F118	F05	No social g	Banks clos	0.6
Map41	F118	F01	No social g	Bad experi	0.4
Map41	F118	F63	No social g	Increased c	0.6
Map41	F118	F147	No social g	Stress and	0.6
Map41	F97	F01	More impris	Bad experi	0.2
Map41	F97	F17	More impris	Delay in jus	0.4
Map41	F114	F81	No pilgrima	Loss of Ha	0.2
Map41	F114	F01	No pilgrima	Bad experi	0.2
Map41	F96	F01	More hung	Bad experi	1
Map41	F116	F01	No product	Bad experi	0.6
Map41	F116	F96	No product	More hung	0.6
Map41	F116	F78	No product	Less food t	0.8
Map41	F53	F01	Increased s	Bad experi	0.2
Map41	F53	F97	Increased s	More impris	1
Map41	F53	F17	Increased s	Delay in jus	0.4
Map41	F58	F01	Increased c	Bad experi	0.2
Map41	F58	F97	Increased c	More impris	0.6
Map41	F58	F17	Increased c	Delay in jus	0.4
Map41	F78	F01	Less food t	Bad experi	0.6
Map41	F78	F96	Less food t	More hung	0.6
Map41	F10	F01	Compulsor	Bad experi	0.4
Map41	F10	F63	Compulsor	Increased c	0.6
Map41	F10	F147	Compulsor	Stress and	0.8
Map41	F16	F01	Delay in ec	Bad experi	0.8
Map41	F17	F01	Delay in jus	Bad experi	0.2

For Peer Review

Map41	F142	F01	School/ma	Bad experi	0.4
Map41	F142	F158	School/ma	Youth/child	0.4
Map41	F142	F16	School/ma	Delay in ec	0.4
Map41	F141	F01	Rumors on	Bad experi	0.4
Map41	F141	F63	Rumors on	Increased (	0.6
Map41	F141	F147	Rumors on	Stress and	0.6
Map41	F140	F85	Restriction	Markets/sh	1
Map41	F140	F01	Restriction	Bad experi	0.6
Map41	F140	F64	Restriction	Increased p	0.6
Map41	F140	F60	Restriction	Increased i	0.6
Map41	F140	F97	Restriction	More impri	0.6
Map41	F140	F96	Restriction	More hung	0.6
Map41	F140	F116	Restriction	No product	0.8
Map41	F140	F53	Restriction	Increased s	0.6
Map41	F140	F58	Restriction	Increased (	0.6
Map41	F140	F78	Restriction	Less food t	0.8
Map41	F140	F17	Restriction	Delay in jus	0.4
Map41	F140	F120	Restriction	No transpo	0.6
Map41	F140	F123	Restriction	Not going t	1
Map41	F140	F128	Restriction	People fee	0.6
Map41	F147	F01	Stress and	Bad experi	0.4
Map41	F147	F63	Stress and	Increased (	0.6
Map41	F120	F01	No transpo	Bad experi	0.6
Map41	F120	F96	No transpo	More hung	0.6
Map41	F120	F78	No transpo	Less food t	0.6
Map41	F123	F01	Not going t	Bad experi	0.6
Map41	F123	F64	Not going t	Increased p	0.6
Map41	F123	F96	Not going t	More hung	0.6
Map41	F123	F78	Not going t	Less food t	0.8
Map41	F128	F01	People feel	Bad experi	0.2
Map41	F128	F60	People feel	Increased i	0.8
Map41	F128	F97	People feel	More impri	0.8
Map41	F128	F53	People feel	Increased s	0.8
Map41	F128	F58	People feel	Increased (	0.6
Map41	F128	F17	People feel	Delay in jus	0.4
Map41	F106	F01	No congreg	Bad experi	0.4
Map41	F106	F63	No congreg	Increased (	0.4
Map41	F106	F147	No congreg	Stress and	0.4
Map41	F100	F01	Mosques/c	Bad experi	0.4
Map41	F100	F63	Mosques/c	Increased (	0.4
Map41	F100	F147	Mosques/c	Stress and	0.4
Map41	F100	F106	Mosques/c	No congreg	0.6
Map42	F40	F47	Household	Increase in	0.6
Map42	F40	F01	Household	Bad experi	0.4
Map42	F88	F40	Men unable	Household	0.4
Map42	F88	F47	Men unable	Increase in	0.4
Map42	F88	F01	Men unable	Bad experi	0.4
Map42	F47	F01	Increase in	Bad experi	0.4
Map42	F85	F40	Markets/sh	Household	0.6
Map42	F85	F47	Markets/sh	Increase in	0.6
Map42	F85	F83	Markets/sh	Malnutritior	0.6
Map42	F85	F82	Markets/sh	Lost invest	0.6
Map42	F85	F01	Markets/sh	Bad experi	0.6
Map42	F85	F64	Markets/sh	Increased p	0.6
Map42	F85	F63	Markets/sh	Increased (	0.6
Map42	F85	F96	Markets/sh	More hung	0.6

For Peer Review

Map42	F85	F135	Markets/sh	Price hike	0.6
Map42	F85	F78	Markets/sh	Less food t	0.6
Map42	F85	F143	Markets/sh	Shortage o	0.6
Map42	F87	F40	Men stayin	Household	0.4
Map42	F87	F88	Men stayin	Men unable	0.6
Map42	F87	F47	Men stayin	Increase in	0.4
Map42	F87	F01	Men stayin	Bad experi	0.4
Map42	F80	F40	Lockdown	Household	0.6
Map42	F80	F88	Lockdown	Men unable	0.6
Map42	F80	F47	Lockdown	Increase in	0.6
Map42	F80	F85	Lockdown	Markets/sh	0.8
Map42	F80	F87	Lockdown	Men stayin	0.6
Map42	F80	F83	Lockdown	Malnutrition	0.6
Map42	F80	F82	Lockdown	Lost invest	0.6
Map42	F80	F01	Lockdown	Bad experi	0.6
Map42	F80	F64	Lockdown	Increased p	0.6
Map42	F80	F63	Lockdown	Increased c	0.6
Map42	F80	F62	Lockdown	Increased r	0.6
Map42	F80	F151	Lockdown	Two shifts	0.8
Map42	F80	F158	Lockdown	Youth/child	0.8
Map42	F80	F16	Lockdown	Delay in ec	0.6
Map42	F80	F96	Lockdown	More hung	0.6
Map42	F80	F135	Lockdown	Price hike	0.6
Map42	F80	F32	Lockdown	Feeling soc	0.4
Map42	F80	F78	Lockdown	Less food t	0.6
Map42	F80	F70	Lockdown	Jobs lost	0.8
Map42	F80	F143	Lockdown	Shortage o	0.6
Map42	F80	F142	Lockdown	School/mac	0.8
Map42	F80	F147	Lockdown	Stress and	0.6
Map42	F80	F120	Lockdown	No transpo	0.8
Map42	F80	F144	Lockdown	Social dista	0.8
Map42	F80	F106	Lockdown	No congreg	0.8
Map42	F80	F100	Lockdown	Mosques/c	1
Map42	F83	F01	Malnutrition	Bad experi	0.6
Map42	F83	F63	Malnutrition	Increased c	0.6
Map42	F82	F01	Lost invest	Bad experi	0.6
Map42	F82	F64	Lost invest	Increased p	0.6
Map42	F20	F01	Difficult bre	Bad experi	0.2
Map42	F29	F01	Fear of cor	Bad experi	0.4
Map42	F29	F30	Fear of cor	Fear of dea	0.4
Map42	F29	F31	Fear of cor	Fear of eac	0.4
Map42	F64	F01	Increased p	Bad experi	0.6
Map42	F63	F01	Increased c	Bad experi	0.8
Map42	F62	F01	Increased r	Bad experi	0.8
Map42	F151	F01	Two shifts i	Bad experi	0.6
Map42	F151	F62	Two shifts i	Increased r	0.6
Map42	F151	F151	Two shifts i	Two shifts	0.8
Map42	F151	F147	Two shifts i	Stress and	0.6
Map42	F158	F01	Youth/child	Bad experi	0.6
Map42	F158	F16	Youth/child	Delay in ec	0.6
Map42	F16	F01	Delay in ec	Bad experi	0.8
Map42	F96	F01	More hung	Bad experi	1
Map42	F96	F63	More hung	Increased c	0.6
Map42	F135	F40	Price hike	Household	0.6
Map42	F135	F47	Price hike	Increase in	0.6
Map42	F135	F83	Price hike	Malnutrition	0.8

For Peer Review

Map42	F135	F01	Price hike	Bad experi	0.6
Map42	F135	F63	Price hike	Increased (	0.6
Map42	F135	F96	Price hike	More hung	0.6
Map42	F135	F78	Price hike	Less food t	0.8
Map42	F30	F01	Fear of dea	Bad experi	0.4
Map42	F31	F01	Fear of eac	Bad experi	0.4
Map42	F31	F30	Fear of eac	Fear of dea	0.6
Map42	F32	F01	Feeling soc	Bad experi	0.8
Map42	F32	F62	Feeling soc	Increased r	0.8
Map42	F78	F40	Less food t	Household	0.6
Map42	F78	F47	Less food t	Increase in	0.6
Map42	F78	F83	Less food t	Malnutritior	0.8
Map42	F78	F01	Less food t	Bad experi	0.6
Map42	F78	F63	Less food t	Increased (	0.6
Map42	F78	F96	Less food t	More hung	0.6
Map42	F10	F20	Compulsor	Difficult bre	0.4
Map42	F10	F01	Compulsor	Bad experi	0.2
Map42	F10	F126	Compulsor	Odour of sa	0.4
Map42	F70	F40	Jobs lost	Household	0.4
Map42	F70	F88	Jobs lost	Men unable	0.6
Map42	F70	F47	Jobs lost	Increase in	0.4
Map42	F70	F87	Jobs lost	Men stayin	0.6
Map42	F70	F01	Jobs lost	Bad experi	0.4
Map42	F70	F64	Jobs lost	Increased p	0.4
Map42	F143	F40	Shortage o	Household	0.4
Map42	F143	F47	Shortage o	Increase in	0.4
Map42	F143	F83	Shortage o	Malnutritior	0.4
Map42	F143	F01	Shortage o	Bad experi	0.4
Map42	F143	F63	Shortage o	Increased (	0.4
Map42	F143	F96	Shortage o	More hung	0.4
Map42	F143	F78	Shortage o	Less food t	0.4
Map42	F142	F01	School/ma	Bad experi	0.6
Map42	F142	F158	School/ma	Youth/child	0.8
Map42	F142	F16	School/ma	Delay in ec	0.6
Map42	F126	F01	Odour of sa	Bad experi	0.2
Map42	F147	F01	Stress and	Bad experi	0.8
Map42	F147	F62	Stress and	Increased r	0.6
Map42	F120	F40	No transpo	Household	0.4
Map42	F120	F47	No transpo	Increase in	0.4
Map42	F120	F83	No transpo	Malnutritior	0.4
Map42	F120	F01	No transpo	Bad experi	0.4
Map42	F120	F63	No transpo	Increased (	0.4
Map42	F120	F96	No transpo	More hung	0.4
Map42	F120	F78	No transpo	Less food t	0.4
Map42	F120	F143	No transpo	Shortage o	0.6
Map42	F144	F01	Social dista	Bad experi	0.6
Map42	F144	F62	Social dista	Increased r	0.6
Map42	F144	F151	Social dista	Two shifts	0.8
Map42	F144	F32	Social dista	Feeling soc	0.4
Map42	F144	F147	Social dista	Stress and	0.6
Map42	F144	F106	Social dista	No congreg	0.8
Map42	F106	F01	No congreg	Bad experi	0.6
Map42	F106	F62	No congreg	Increased r	0.6
Map42	F106	F147	No congreg	Stress and	0.6
Map42	F100	F01	Mosques/c	Bad experi	0.6
Map42	F100	F62	Mosques/c	Increased r	0.6

For Peer Review

Map42	F100	F147	Mosques/c	Stress and	0.6
Map42	F100	F106	Mosques/c	No congreg	0.6
Map43	F40	F47	Household	Increase in	0.6
Map43	F40	F01	Household	Bad experi	0.6
Map43	F40	F137	Household	Reduced s	0.8
Map43	F47	F01	Increase in	Bad experi	0.6
Map43	F85	F82	Markets/sh	Lost invest	0.8
Map43	F85	F07	Markets/sh	Businesses	0.8
Map43	F85	F01	Markets/sh	Bad experi	0.8
Map43	F85	F64	Markets/sh	Increased p	0.8
Map43	F85	F96	Markets/sh	More hung	0.8
Map43	F85	F112	Markets/sh	No home/p	0.8
Map43	F85	F78	Markets/sh	Less food t	0.8
Map43	F87	F40	Men stayin	Household	0.8
Map43	F87	F47	Men stayin	Increase in	0.6
Map43	F87	F01	Men stayin	Bad experi	0.6
Map43	F87	F137	Men stayin	Reduced s	0.8
Map43	F80	F40	Lockdown	Household	0.8
Map43	F80	F47	Lockdown	Increase in	0.6
Map43	F80	F85	Lockdown	Markets/sh	1
Map43	F80	F87	Lockdown	Men stayin	1
Map43	F80	F82	Lockdown	Lost invest	0.8
Map43	F80	F07	Lockdown	Businesses	0.8
Map43	F80	F01	Lockdown	Bad experi	0.8
Map43	F80	F64	Lockdown	Increased p	0.8
Map43	F80	F62	Lockdown	Increased r	0.6
Map43	F80	F157	Lockdown	Women un	0.6
Map43	F80	F158	Lockdown	Youth/child	0.6
Map43	F80	F118	Lockdown	No social g	0.6
Map43	F80	F96	Lockdown	More hung	0.8
Map43	F80	F137	Lockdown	Reduced s	0.8
Map43	F80	F112	Lockdown	No home/p	0.8
Map43	F80	F78	Lockdown	Less food t	0.8
Map43	F80	F13	Lockdown	Could not c	0.6
Map43	F80	F16	Lockdown	Delay in ec	0.6
Map43	F80	F142	Lockdown	School/ma	0.8
Map43	F80	F140	Lockdown	Restriction	1
Map43	F80	F147	Lockdown	Stress and	0.6
Map43	F80	F120	Lockdown	No transpo	0.6
Map43	F80	F149	Lockdown	Teachers a	0.6
Map43	F80	F106	Lockdown	No congreg	0.6
Map43	F80	F100	Lockdown	Mosques/c	0.8
Map43	F82	F01	Lost invest	Bad experi	0.8
Map43	F82	F64	Lost invest	Increased p	0.8
Map43	F82	F96	Lost invest	More hung	0.8
Map43	F82	F78	Lost invest	Less food t	0.8
Map43	F07	F82	Businesses	Lost invest	0.8
Map43	F07	F01	Businesses	Bad experi	0.8
Map43	F07	F64	Businesses	Increased p	0.8
Map43	F07	F96	Businesses	More hung	0.8
Map43	F07	F112	Businesses	No home/p	0.8
Map43	F07	F78	Businesses	Less food t	0.8
Map43	F02	F01	Bad experi	Bad experi	0.6
Map43	F29	F01	Fear of cor	Bad experi	0.6
Map43	F29	F31	Fear of cor	Fear of eac	0.6
Map43	F64	F01	Increased p	Bad experi	0.8

For Peer Review

Map43	F64	F96	Increased ‖	More hung‖	0.8
Map43	F64	F78	Increased ‖	Less food t	0.8
Map43	F62	F01	Increased ı	Bad experi	0.6
Map43	F157	F01	Women un	Bad experi	0.4
Map43	F157	F118	Women un	No social g	0.6
Map43	F158	F01	Youth/child	Bad experi	0.6
Map43	F158	F13	Youth/child	Could not c	0.6
Map43	F158	F16	Youth/child	Delay in ec	0.8
Map43	F158	F147	Youth/child	Stress and	0.8
Map43	F118	F01	No social g	Bad experi	0.4
Map43	F96	F01	More hung	Bad experi	0.6
Map43	F137	F47	Reduced s	Increase in	0.6
Map43	F137	F01	Reduced s	Bad experi	0.6
Map43	F112	F82	No home/p	Lost invest	1
Map43	F112	F01	No home/p	Bad experi	0.8
Map43	F112	F64	No home/p	Increased ‖	0.8
Map43	F112	F96	No home/p	More hung	0.8
Map43	F112	F78	No home/p	Less food t	0.8
Map43	F124	F01	Not visiting	Bad experi	0.6
Map43	F124	F02	Not visiting	Bad experi	0.8
Map43	F31	F01	Fear of eac	Bad experi	0.8
Map43	F78	F01	Less food t	Bad experi	0.6
Map43	F78	F96	Less food t	More hung	0.8
Map43	F74	F01	Lack of fac	Bad experi	0.4
Map43	F13	F01	Could not c	Bad experi	0.6
Map43	F13	F147	Could not c	Stress and	0.8
Map43	F10	F01	Compulsor	Bad experi	0.2
Map43	F10	F74	Compulsor	Lack of fac	0.2
Map43	F10	F108	Compulsor	No entry at	0.2
Map43	F16	F01	Delay in ec	Bad experi	0.6
Map43	F16	F147	Delay in ec	Stress and	1
Map43	F142	F01	School/ma	Bad experi	0.6
Map43	F142	F158	School/ma	Youth/child	0.6
Map43	F142	F13	School/ma	Could not c	0.6
Map43	F142	F16	School/ma	Delay in ec	0.6
Map43	F142	F147	School/ma	Stress and	0.6
Map43	F140	F01	Restriction	Bad experi	0.6
Map43	F140	F158	Restriction	Youth/child	0.6
Map43	F140	F13	Restriction	Could not c	0.6
Map43	F140	F16	Restriction	Delay in ec	0.6
Map43	F140	F142	Restriction	School/ma	0.6
Map43	F140	F147	Restriction	Stress and	0.6
Map43	F140	F120	Restriction	No transpo	0.6
Map43	F140	F149	Restriction	Teachers a	0.6
Map43	F147	F01	Stress and	Bad experi	0.6
Map43	F120	F01	No transpo	Bad experi	0.6
Map43	F120	F158	No transpo	Youth/child	0.6
Map43	F120	F13	No transpo	Could not c	0.6
Map43	F120	F16	No transpo	Delay in ec	0.6
Map43	F120	F142	No transpo	School/ma	0.6
Map43	F120	F147	No transpo	Stress and	0.6
Map43	F120	F149	No transpo	Teachers a	0.6
Map43	F144	F01	Social dista	Bad experi	0.4
Map43	F144	F118	Social dista	No social g	0.4
Map43	F149	F01	Teachers a	Bad experi	0.6
Map43	F149	F158	Teachers a	Youth/child	0.6

For Peer Review

Map43	F149	F13	Teachers a	Could not c	0.6
Map43	F149	F16	Teachers a	Delay in ec	0.6
Map43	F149	F142	Teachers a	School/mad	0.6
Map43	F149	F147	Teachers a	Stress and	0.6
Map43	F108	F01	No entry at	Bad experi	0.4
Map43	F108	F74	No entry at	Lack of fac	0.4
Map43	F106	F01	No congreg	Bad experi	0.6
Map43	F106	F62	No congreg	Increased r	0.8
Map43	F100	F01	Mosques/c	Bad experi	0.6
Map43	F100	F62	Mosques/c	Increased r	0.6
Map43	F100	F106	Mosques/c	No congreg	0.6
Map44	F40	F47	Household	Increase in	0.2
Map44	F40	F01	Household	Bad experi	0.2
Map44	F88	F40	Men unable	Household	0.6
Map44	F88	F47	Men unable	Increase in	0.2
Map44	F88	F01	Men unable	Bad experi	0.4
Map44	F88	F96	Men unable	More hung	0.4
Map44	F88	F78	Men unable	Less food t	0.4
Map44	F88	F143	Men unable	Shortage o	0.4
Map44	F47	F01	Increase in	Bad experi	0.6
Map44	F85	F40	Markets/sh	Household	0.4
Map44	F85	F47	Markets/sh	Increase in	0.2
Map44	F85	F82	Markets/sh	Lost invest	0.8
Map44	F85	F07	Markets/sh	Businesses	0.8
Map44	F85	F01	Markets/sh	Bad experi	0.8
Map44	F85	F64	Markets/sh	Increased p	0.8
Map44	F85	F63	Markets/sh	Increased c	0.6
Map44	F85	F96	Markets/sh	More hung	0.6
Map44	F85	F135	Markets/sh	Price hike	0.8
Map44	F85	F124	Markets/sh	Not visiting	0.8
Map44	F85	F78	Markets/sh	Less food t	0.6
Map44	F85	F14	Markets/sh	Could not g	0.8
Map44	F85	F143	Markets/sh	Shortage o	0.6
Map44	F87	F40	Men stayin	Household	0.6
Map44	F87	F88	Men stayin	Men unable	0.6
Map44	F87	F47	Men stayin	Increase in	0.2
Map44	F87	F01	Men stayin	Bad experi	0.4
Map44	F87	F96	Men stayin	More hung	0.4
Map44	F87	F78	Men stayin	Less food t	0.4
Map44	F87	F70	Men stayin	Jobs lost	0.6
Map44	F87	F143	Men stayin	Shortage o	0.4
Map44	F80	F40	Lockdown	Household	0.4
Map44	F80	F88	Lockdown	Men unable	0.4
Map44	F80	F47	Lockdown	Increase in	0.2
Map44	F80	F85	Lockdown	Markets/sh	1
Map44	F80	F87	Lockdown	Men stayin	0.4
Map44	F80	F82	Lockdown	Lost invest	0.8
Map44	F80	F07	Lockdown	Businesses	0.8
Map44	F80	F01	Lockdown	Bad experi	0.8
Map44	F80	F64	Lockdown	Increased p	0.8
Map44	F80	F63	Lockdown	Increased c	0.6
Map44	F80	F157	Lockdown	Women un	0.2
Map44	F80	F16	Lockdown	Delay in ec	0.6
Map44	F80	F118	Lockdown	No social g	0.2
Map44	F80	F96	Lockdown	More hung	0.6
Map44	F80	F135	Lockdown	Price hike	0.8

For Peer Review

Map44	F80	F124	Lockdown	Not visiting	0.8
Map44	F80	F78	Lockdown	Less food t	0.6
Map44	F80	F13	Lockdown	Could not c	0.4
Map44	F80	F70	Lockdown	Jobs lost	0.4
Map44	F80	F14	Lockdown	Could not g	0.8
Map44	F80	F143	Lockdown	Shortage o	0.6
Map44	F80	F142	Lockdown	School/ma	0.6
Map44	F80	F140	Lockdown	Restriction	0.6
Map44	F80	F147	Lockdown	Stress and	0.6
Map44	F80	F120	Lockdown	No transpo	0.8
Map44	F80	F149	Lockdown	Teachers a	0.6
Map44	F80	F109	Lockdown	No financia	0.2
Map44	F80	F106	Lockdown	No congreg	0.8
Map44	F80	F100	Lockdown	Mosques/c	0.8
Map44	F82	F01	Lost invest	Bad experi	0.8
Map44	F82	F64	Lost invest	Increased p	0.8
Map44	F82	F63	Lost invest	Increased c	0.6
Map44	F82	F96	Lost invest	More hung	0.6
Map44	F82	F124	Lost invest	Not visiting	0.8
Map44	F82	F78	Lost invest	Less food t	0.6
Map44	F82	F14	Lost invest	Could not g	0.8
Map44	F07	F82	Businesses	Lost invest	1
Map44	F07	F01	Businesses	Bad experi	0.8
Map44	F07	F64	Businesses	Increased p	1
Map44	F07	F63	Businesses	Increased c	0.6
Map44	F07	F96	Businesses	More hung	0.6
Map44	F07	F124	Businesses	Not visiting	0.8
Map44	F07	F78	Businesses	Less food t	0.6
Map44	F07	F14	Businesses	Could not g	0.8
Map44	F20	F01	Difficult bre	Bad experi	0.4
Map44	F64	F01	Increased p	Bad experi	0.8
Map44	F64	F63	Increased p	Increased c	0.6
Map44	F64	F96	Increased p	More hung	0.6
Map44	F64	F124	Increased p	Not visiting	0.8
Map44	F64	F78	Increased p	Less food t	0.6
Map44	F64	F14	Increased p	Could not g	0.8
Map44	F63	F01	Increased c	Bad experi	1
Map44	F154	F20	Wearing fa	Difficult bre	0.8
Map44	F154	F01	Wearing fa	Bad experi	0.4
Map44	F157	F01	Women un	Bad experi	0.4
Map44	F157	F118	Women un	No social g	0.4
Map44	F157	F147	Women un	Stress and	0.4
Map44	F157	F109	Women un	No financia	0.4
Map44	F16	F01	Delay in ed	Bad experi	0.6
Map44	F118	F01	No social g	Bad experi	0.6
Map44	F118	F147	No social g	Stress and	0.6
Map44	F118	F109	No social g	No financia	0.6
Map44	F96	F01	More hung	Bad experi	0.8
Map44	F135	F40	Price hike	Household	0.4
Map44	F135	F47	Price hike	Increase in	0.2
Map44	F135	F01	Price hike	Bad experi	0.6
Map44	F135	F96	Price hike	More hung	0.6
Map44	F135	F78	Price hike	Less food t	0.6
Map44	F135	F143	Price hike	Shortage o	0.6
Map44	F124	F01	Not visiting	Bad experi	0.6
Map44	F124	F63	Not visiting	Increased c	0.6

For Peer Review

Map44	F124	F14	Not visiting	Could not g	1
Map44	F78	F01	Less food t	Bad experi	1
Map44	F78	F96	Less food t	More hung	0.6
Map44	F13	F01	Could not c	Bad experi	0.8
Map44	F13	F147	Could not c	Stress and	0.8
Map44	F10	F20	Compulsor	Difficult bre	0.8
Map44	F10	F01	Compulsor	Bad experi	0.6
Map44	F10	F63	Compulsor	Increased c	0.6
Map44	F10	F154	Compulsor	Wearing fa	0.8
Map44	F10	F14	Compulsor	Could not g	0.6
Map44	F10	F108	Compulsor	No entry at	0.8
Map44	F70	F40	Jobs lost	Household	0.6
Map44	F70	F88	Jobs lost	Men unable	0.8
Map44	F70	F47	Jobs lost	Increase in	0.2
Map44	F70	F01	Jobs lost	Bad experi	0.4
Map44	F70	F96	Jobs lost	More hung	0.4
Map44	F70	F78	Jobs lost	Less food t	0.4
Map44	F70	F143	Jobs lost	Shortage o	0.4
Map44	F14	F01	Could not g	Bad experi	0.6
Map44	F14	F63	Could not g	Increased c	0.6
Map44	F143	F40	Shortage o	Household	0.4
Map44	F143	F47	Shortage o	Increase in	0.2
Map44	F143	F01	Shortage o	Bad experi	0.8
Map44	F143	F96	Shortage o	More hung	0.6
Map44	F143	F78	Shortage o	Less food t	0.8
Map44	F142	F01	School/ma	Bad experi	0.6
Map44	F142	F16	School/ma	Delay in ec	0.8
Map44	F142	F13	School/ma	Could not c	0.4
Map44	F142	F147	School/ma	Stress and	0.4
Map44	F140	F01	Restriction	Bad experi	0.2
Map44	F140	F157	Restriction	Women un	0.2
Map44	F140	F118	Restriction	No social g	0.2
Map44	F140	F147	Restriction	Stress and	0.2
Map44	F140	F109	Restriction	No financia	0.2
Map44	F147	F01	Stress and	Bad experi	1
Map44	F120	F01	No transpo	Bad experi	0.4
Map44	F120	F16	No transpo	Delay in ec	0.4
Map44	F120	F13	No transpo	Could not c	0.4
Map44	F120	F142	No transpo	School/ma	0.4
Map44	F120	F147	No transpo	Stress and	0.4
Map44	F120	F149	No transpo	Teachers a	0.6
Map44	F144	F01	Social dista	Bad experi	0.6
Map44	F144	F118	Social dista	No social g	0.6
Map44	F144	F147	Social dista	Stress and	0.6
Map44	F144	F109	Social dista	No financia	0.6
Map44	F149	F01	Teachers a	Bad experi	0.4
Map44	F149	F16	Teachers a	Delay in ec	0.4
Map44	F149	F13	Teachers a	Could not c	0.4
Map44	F149	F142	Teachers a	School/ma	0.4
Map44	F149	F147	Teachers a	Stress and	0.4
Map44	F109	F01	No financia	Bad experi	0.8
Map44	F109	F147	No financia	Stress and	0.8
Map44	F108	F01	No entry at	Bad experi	0.6
Map44	F108	F63	No entry at	Increased c	0.6
Map44	F108	F14	No entry at	Could not g	0.6
Map44	F106	F01	No congreg	Bad experi	0.6

For Peer Review

Map44	F106	F147	No congreg	Stress and	0.6
Map44	F100	F01	Mosques/c	Bad experi	0.6
Map44	F100	F147	Mosques/c	Stress and	0.6
Map44	F100	F106	Mosques/c	No congreg	1
Map45	F85	F82	Markets/sh	Lost invest	0.6
Map45	F85	F01	Markets/sh	Bad experi	0.4
Map45	F85	F67	Markets/sh	Increased s	0.6
Map45	F85	F64	Markets/sh	Increased p	0.6
Map45	F85	F62	Markets/sh	Increased r	0.6
Map45	F85	F97	Markets/sh	More impris	0.6
Map45	F85	F96	Markets/sh	More hung	0.4
Map45	F85	F78	Markets/sh	Less food t	0.6
Map45	F80	F85	Lockdown	Markets/sh	1
Map45	F80	F82	Lockdown	Lost invest	0.6
Map45	F80	F05	Lockdown	Banks clos	0.4
Map45	F80	F20	Lockdown	Difficult bre	0.4
Map45	F80	F01	Lockdown	Bad experi	0.6
Map45	F80	F67	Lockdown	Increased s	0.6
Map45	F80	F64	Lockdown	Increased p	0.6
Map45	F80	F63	Lockdown	Increased c	0.6
Map45	F80	F62	Lockdown	Increased r	0.6
Map45	F80	F154	Lockdown	Wearing fa	0.6
Map45	F80	F118	Lockdown	No social g	0.6
Map45	F80	F97	Lockdown	More impris	0.6
Map45	F80	F96	Lockdown	More hung	0.4
Map45	F80	F78	Lockdown	Less food t	0.6
Map45	F80	F38	Lockdown	Health wor	0.6
Map45	F80	F121	Lockdown	No wedding	0.4
Map45	F80	F10	Lockdown	Compulsor	0.4
Map45	F80	F14	Lockdown	Could not g	0.6
Map45	F80	F140	Lockdown	Restriction	1
Map45	F80	F147	Lockdown	Stress and	0.4
Map45	F80	F144	Lockdown	Social dista	0.8
Map45	F80	F108	Lockdown	No entry at	0.6
Map45	F80	F106	Lockdown	No congreg	0.6
Map45	F80	F100	Lockdown	Mosques/c	0.8
Map45	F82	F01	Lost invest	Bad experi	0.4
Map45	F82	F67	Lost invest	Increased s	0.6
Map45	F82	F64	Lost invest	Increased p	0.8
Map45	F82	F62	Lost invest	Increased r	0.6
Map45	F82	F97	Lost invest	More impris	0.6
Map45	F82	F96	Lost invest	More hung	0.4
Map45	F82	F78	Lost invest	Less food t	0.8
Map45	F05	F20	Banks clos	Difficult bre	0.4
Map45	F05	F01	Banks clos	Bad experi	0.4
Map45	F05	F154	Banks clos	Wearing fa	0.6
Map45	F05	F10	Banks clos	Compulsor	0.6
Map45	F05	F147	Banks clos	Stress and	0.2
Map45	F20	F01	Difficult bre	Bad experi	0.6
Map45	F67	F01	Increased s	Bad experi	0.4
Map45	F67	F62	Increased s	Increased r	0.6
Map45	F67	F97	Increased s	More impris	0.6
Map45	F29	F01	Fear of cor	Bad experi	0.4
Map45	F29	F118	Fear of cor	No social g	0.6
Map45	F29	F31	Fear of cor	Fear of eac	0.6
Map45	F29	F121	Fear of cor	No wedding	0.4

For Peer Review

Map45	F29	F147	Fear of cor	Stress and	0.4
Map45	F64	F01	Increased	Bad experi	0.4
Map45	F64	F67	Increased	Increased	0.6
Map45	F64	F62	Increased	Increased	0.6
Map45	F64	F97	Increased	More impris	0.6
Map45	F64	F96	Increased	More hung	0.4
Map45	F64	F78	Increased	Less food t	0.8
Map45	F63	F01	Increased	Bad experi	0.8
Map45	F62	F01	Increased	Bad experi	0.4
Map45	F154	F20	Wearing fa	Difficult bre	0.4
Map45	F154	F01	Wearing fa	Bad experi	0.4
Map45	F118	F01	No social g	Bad experi	0.4
Map45	F118	F121	No social g	No wedding	0.4
Map45	F118	F147	No social g	Stress and	0.4
Map45	F97	F01	More impris	Bad experi	0.4
Map45	F97	F62	More impris	Increased	0.8
Map45	F96	F01	More hung	Bad experi	1
Map45	F30	F01	Fear of dea	Bad experi	0.4
Map45	F30	F29	Fear of dea	Fear of cor	0.8
Map45	F30	F118	Fear of dea	No social g	0.6
Map45	F30	F31	Fear of dea	Fear of eac	0.6
Map45	F30	F121	Fear of dea	No wedding	0.4
Map45	F30	F147	Fear of dea	Stress and	0.4
Map45	F31	F01	Fear of eac	Bad experi	0.4
Map45	F31	F118	Fear of eac	No social g	0.6
Map45	F31	F121	Fear of eac	No wedding	0.4
Map45	F31	F147	Fear of eac	Stress and	0.4
Map45	F78	F01	Less food t	Bad experi	0.4
Map45	F78	F96	Less food t	More hung	0.4
Map45	F38	F01	Health wor	Bad experi	0.8
Map45	F38	F63	Health wor	Increased	0.8
Map45	F38	F14	Health wor	Could not	0.8
Map45	F121	F01	No wedding	Bad experi	0.6
Map45	F121	F147	No wedding	Stress and	0.6
Map45	F10	F01	Compulsor	Bad experi	0.2
Map45	F10	F147	Compulsor	Stress and	0.2
Map45	F14	F01	Could not	Bad experi	0.8
Map45	F14	F63	Could not	Increased	1
Map45	F141	F01	Rumors on	Bad experi	0.4
Map45	F141	F29	Rumors on	Fear of cor	1
Map45	F141	F118	Rumors on	No social g	0.6
Map45	F141	F31	Rumors on	Fear of eac	0.6
Map45	F141	F121	Rumors on	No wedding	0.4
Map45	F141	F147	Rumors on	Stress and	0.4
Map45	F140	F01	Restriction	Bad experi	0.6
Map45	F140	F63	Restriction	Increased	0.6
Map45	F140	F38	Restriction	Health wor	0.6
Map45	F140	F14	Restriction	Could not	0.6
Map45	F147	F01	Stress and	Bad experi	0.8
Map45	F144	F05	Social dista	Banks clos	0.4
Map45	F144	F20	Social dista	Difficult bre	0.4
Map45	F144	F01	Social dista	Bad experi	0.6
Map45	F144	F154	Social dista	Wearing fa	0.6
Map45	F144	F118	Social dista	No social g	0.6
Map45	F144	F121	Social dista	No wedding	0.4
Map45	F144	F10	Social dista	Compulsor	0.4

For Peer Review

Map45	F144	F147	Social dista	Stress and	0.4
Map45	F144	F108	Social dista	No entry at	0.6
Map45	F144	F106	Social dista	No congreg	0.6
Map45	F144	F100	Social dista	Mosques/c	0.8
Map45	F108	F20	No entry at	Difficult bre	0.4
Map45	F108	F01	No entry at	Bad experi	0.4
Map45	F108	F154	No entry at	Wearing fa	0.6
Map45	F106	F01	No congreg	Bad experi	1
Map45	F100	F01	Mosques/c	Bad experi	0.6
Map45	F100	F106	Mosques/c	No congreg	0.6
Map46	F88	F83	Men unable	Malnutrition	0.4
Map46	F88	F01	Men unable	Bad experi	0.6
Map46	F88	F63	Men unable	Increased	0.8
Map46	F88	F96	Men unable	More hung	0.8
Map46	F85	F83	Markets/sh	Malnutrition	0.4
Map46	F85	F82	Markets/sh	Lost invest	0.6
Map46	F85	F01	Markets/sh	Bad experi	0.6
Map46	F85	F64	Markets/sh	Increased	0.6
Map46	F85	F63	Markets/sh	Increased	0.6
Map46	F85	F96	Markets/sh	More hung	0.6
Map46	F85	F112	Markets/sh	No home/p	0.8
Map46	F85	F135	Markets/sh	Price hike	0.6
Map46	F85	F143	Markets/sh	Shortage o	0.8
Map46	F80	F88	Lockdown	Men unable	0.8
Map46	F80	F85	Lockdown	Markets/sh	1
Map46	F80	F83	Lockdown	Malnutrition	0.4
Map46	F80	F82	Lockdown	Lost invest	0.6
Map46	F80	F01	Lockdown	Bad experi	0.6
Map46	F80	F64	Lockdown	Increased	0.6
Map46	F80	F63	Lockdown	Increased	0.8
Map46	F80	F158	Lockdown	Youth/child	0.8
Map46	F80	F96	Lockdown	More hung	0.8
Map46	F80	F112	Lockdown	No home/p	0.8
Map46	F80	F135	Lockdown	Price hike	0.6
Map46	F80	F13	Lockdown	Could not c	0.4
Map46	F80	F143	Lockdown	Shortage o	0.8
Map46	F80	F142	Lockdown	School/ma	1
Map46	F80	F140	Lockdown	Restriction	0.8
Map46	F80	F147	Lockdown	Stress and	0.4
Map46	F80	F120	Lockdown	No transpo	0.8
Map46	F83	F01	Malnutrition	Bad experi	0.4
Map46	F83	F63	Malnutrition	Increased	0.4
Map46	F82	F83	Lost invest	Malnutrition	0.4
Map46	F82	F01	Lost invest	Bad experi	0.6
Map46	F82	F64	Lost invest	Increased	0.6
Map46	F82	F63	Lost invest	Increased	0.6
Map46	F82	F96	Lost invest	More hung	0.6
Map46	F20	F01	Difficult bre	Bad experi	0.2
Map46	F29	F20	Fear of cor	Difficult bre	0.4
Map46	F29	F01	Fear of cor	Bad experi	0.2
Map46	F29	F30	Fear of cor	Fear of dea	0.6
Map46	F29	F31	Fear of cor	Fear of eac	0.4
Map46	F29	F10	Fear of cor	Compulsor	0.6
Map46	F64	F83	Increased	Malnutrition	0.4
Map46	F64	F01	Increased	Bad experi	0.8
Map46	F64	F63	Increased	Increased	0.8

For Peer Review

Map46	F64	F96	Increased p	More hung	0.8
Map46	F63	F01	Increased c	Bad experi	0.6
Map46	F62	F01	Increased r	Bad experi	0.4
Map46	F158	F01	Youth/child	Bad experi	0.4
Map46	F158	F13	Youth/child	Could not c	0.4
Map46	F158	F147	Youth/child	Stress and	0.4
Map46	F96	F83	More hung	Malnutrition	0.4
Map46	F96	F01	More hung	Bad experi	0.6
Map46	F96	F63	More hung	Increased c	0.8
Map46	F115	F01	No preachi	Bad experi	0.4
Map46	F115	F147	No preachi	Stress and	0.4
Map46	F110	F01	No funerals	Bad experi	0.4
Map46	F110	F62	No funerals	Increased r	0.4
Map46	F112	F83	No home/p	Malnutrition	0.4
Map46	F112	F82	No home/p	Lost invest	0.6
Map46	F112	F01	No home/p	Bad experi	0.6
Map46	F112	F64	No home/p	Increased p	0.6
Map46	F112	F63	No home/p	Increased c	0.6
Map46	F112	F96	No home/p	More hung	0.6
Map46	F135	F83	Price hike	Malnutrition	0.4
Map46	F135	F01	Price hike	Bad experi	0.8
Map46	F135	F64	Price hike	Increased p	0.8
Map46	F135	F63	Price hike	Increased c	0.8
Map46	F135	F96	Price hike	More hung	0.8
Map46	F30	F01	Fear of dea	Bad experi	0.2
Map46	F30	F31	Fear of dea	Fear of eac	0.4
Map46	F31	F01	Fear of eac	Bad experi	0.2
Map46	F32	F01	Feeling soc	Bad experi	0.4
Map46	F118	F01	No social g	Bad experi	0.4
Map46	F118	F32	No social g	Feeling soc	0.4
Map46	F13	F01	Could not c	Bad experi	0.4
Map46	F13	F147	Could not c	Stress and	0.4
Map46	F10	F20	Compulsor	Difficult bre	0.4
Map46	F10	F01	Compulsor	Bad experi	0.2
Map46	F143	F83	Shortage o	Malnutrition	0.4
Map46	F143	F01	Shortage o	Bad experi	0.6
Map46	F143	F64	Shortage o	Increased p	0.6
Map46	F143	F63	Shortage o	Increased c	0.6
Map46	F143	F96	Shortage o	More hung	0.6
Map46	F143	F135	Shortage o	Price hike	0.6
Map46	F142	F01	School/ma	Bad experi	0.4
Map46	F142	F158	School/ma	Youth/child	0.8
Map46	F142	F13	School/ma	Could not c	0.4
Map46	F142	F147	School/ma	Stress and	0.4
Map46	F140	F88	Restriction	Men unable	0.8
Map46	F140	F83	Restriction	Malnutrition	0.4
Map46	F140	F01	Restriction	Bad experi	0.6
Map46	F140	F64	Restriction	Increased p	0.6
Map46	F140	F63	Restriction	Increased c	0.8
Map46	F140	F96	Restriction	More hung	0.8
Map46	F140	F135	Restriction	Price hike	0.6
Map46	F140	F143	Restriction	Shortage o	0.6
Map46	F140	F120	Restriction	No transpo	0.8
Map46	F147	F01	Stress and	Bad experi	0.4
Map46	F120	F88	No transpo	Men unable	1
Map46	F120	F83	No transpo	Malnutrition	0.4

For Peer Review

Map46	F120	F01	No transpo	Bad experi	0.6
Map46	F120	F64	No transpo	Increased p	0.6
Map46	F120	F63	No transpo	Increased c	0.8
Map46	F120	F96	No transpo	More hung	0.8
Map46	F120	F135	No transpo	Price hike	0.6
Map46	F120	F143	No transpo	Shortage o	0.6
Map46	F144	F88	Social dista	Men unable	0.8
Map46	F144	F83	Social dista	Malnutrition	0.4
Map46	F144	F01	Social dista	Bad experi	0.6
Map46	F144	F64	Social dista	Increased p	0.6
Map46	F144	F63	Social dista	Increased c	0.8
Map46	F144	F62	Social dista	Increased r	0.4
Map46	F144	F96	Social dista	More hung	0.8
Map46	F144	F115	Social dista	No preachi	0.4
Map46	F144	F110	Social dista	No funerals	0.6
Map46	F144	F135	Social dista	Price hike	0.6
Map46	F144	F32	Social dista	Feeling soc	0.4
Map46	F144	F118	Social dista	No social g	0.8
Map46	F144	F143	Social dista	Shortage o	0.6
Map46	F144	F147	Social dista	Stress and	0.4
Map46	F144	F120	Social dista	No transpo	0.8
Map46	F144	F106	Social dista	No congreg	0.6
Map46	F144	F100	Social dista	Mosques/c	0.8
Map46	F106	F01	No congreg	Bad experi	0.4
Map46	F106	F115	No congreg	No preachi	0.4
Map46	F106	F147	No congreg	Stress and	0.4
Map46	F100	F01	Mosques/c	Bad experi	0.4
Map46	F100	F115	Mosques/c	No preachi	0.4
Map46	F100	F147	Mosques/c	Stress and	0.4
Map46	F100	F106	Mosques/c	No congreg	0.6
Map47	F40	F01	Household	Bad experi	0.6
Map47	F42	F01	Immunizati	Bad experi	0.6
Map47	F88	F40	Men unable	Household	0.6
Map47	F88	F01	Men unable	Bad experi	0.6
Map47	F85	F40	Markets/sh	Household	0.6
Map47	F85	F88	Markets/sh	Men unable	0.6
Map47	F85	F87	Markets/sh	Men stayin	0.6
Map47	F85	F82	Markets/sh	Lost invest	0.6
Map47	F85	F07	Markets/sh	Businesses	0.6
Map47	F85	F01	Markets/sh	Bad experi	0.6
Map47	F85	F67	Markets/sh	Increased s	0.6
Map47	F85	F64	Markets/sh	Increased p	0.6
Map47	F87	F40	Men stayin	Household	0.6
Map47	F87	F88	Men stayin	Men unable	0.6
Map47	F87	F01	Men stayin	Bad experi	0.6
Map47	F80	F40	Lockdown	Household	0.6
Map47	F80	F88	Lockdown	Men unable	0.6
Map47	F80	F85	Lockdown	Markets/sh	0.6
Map47	F80	F87	Lockdown	Men stayin	0.8
Map47	F80	F83	Lockdown	Malnutrition	0.6
Map47	F80	F82	Lockdown	Lost invest	0.6
Map47	F80	F07	Lockdown	Businesses	0.6
Map47	F80	F01	Lockdown	Bad experi	0.6
Map47	F80	F69	Lockdown	Increased t	0.6
Map47	F80	F67	Lockdown	Increased s	0.6
Map47	F80	F64	Lockdown	Increased p	0.6

For Peer Review

Map47	F80	F63	Lockdown	Increased (	0.6
Map47	F80	F158	Lockdown	Youth/child	0.8
Map47	F80	F96	Lockdown	More hung	0.6
Map47	F80	F115	Lockdown	No preachi	0.6
Map47	F80	F78	Lockdown	Less food t	0.6
Map47	F80	F16	Lockdown	Delay in ec	0.6
Map47	F80	F143	Lockdown	Shortage o	0.6
Map47	F80	F142	Lockdown	School/ma	0.8
Map47	F80	F140	Lockdown	Restriction	0.8
Map47	F80	F147	Lockdown	Stress and	0.6
Map47	F80	F120	Lockdown	No transpo	0.8
Map47	F80	F100	Lockdown	Mosques/c	0.6
Map47	F83	F01	Malnutritior	Bad experi	0.8
Map47	F82	F01	Lost invest	Bad experi	0.6
Map47	F82	F67	Lost invest	Increased s	0.6
Map47	F82	F64	Lost invest	Increased p	0.6
Map47	F07	F40	Businesses	Household	0.6
Map47	F07	F88	Businesses	Men unable	0.6
Map47	F07	F87	Businesses	Men stayin	0.8
Map47	F07	F82	Businesses	Lost invest	0.6
Map47	F07	F01	Businesses	Bad experi	0.6
Map47	F07	F67	Businesses	Increased s	0.6
Map47	F07	F64	Businesses	Increased p	0.6
Map47	F20	F01	Difficult bre	Bad experi	0.4
Map47	F69	F01	Increased t	Bad experi	0.4
Map47	F67	F01	Increased s	Bad experi	0.4
Map47	F29	F20	Fear of cor	Difficult bre	0.2
Map47	F29	F01	Fear of cor	Bad experi	0.4
Map47	F29	F10	Fear of cor	Compulsor	0.4
Map47	F29	F31	Fear of cor	Fear of eac	0.6
Map47	F29	F76	Fear of cor	Less affect	0.4
Map47	F64	F01	Increased p	Bad experi	0.8
Map47	F64	F67	Increased p	Increased s	0.6
Map47	F63	F01	Increased (	Bad experi	0.8
Map47	F10	F20	Compulsor	Difficult bre	0.2
Map47	F10	F01	Compulsor	Bad experi	0.2
Map47	F158	F01	Youth/child	Bad experi	0.6
Map47	F158	F69	Youth/child	Increased t	0.6
Map47	F158	F16	Youth/child	Delay in ec	0.6
Map47	F96	F83	More hung	Malnutritior	0.8
Map47	F96	F01	More hung	Bad experi	0.8
Map47	F96	F63	More hung	Increased (	0.8
Map47	F115	F01	No preachi	Bad experi	0.8
Map47	F115	F147	No preachi	Stress and	0.8
Map47	F31	F01	Fear of eac	Bad experi	0.4
Map47	F31	F76	Fear of eac	Less affect	0.4
Map47	F78	F83	Less food t	Malnutritior	0.8
Map47	F78	F01	Less food t	Bad experi	0.8
Map47	F78	F63	Less food t	Increased (	0.8
Map47	F78	F96	Less food t	More hung	1
Map47	F37	F42	Health facil	Immunizati	0.6
Map47	F37	F01	Health facil	Bad experi	0.6
Map47	F37	F14	Health facil	Could not g	0.6
Map47	F76	F01	Less affect	Bad experi	0.4
Map47	F16	F01	Delay in ec	Bad experi	0.6
Map47	F14	F01	Could not g	Bad experi	0.8

For Peer Review

Map47	F143	F83	Shortage o	Malnutritior	0.8
Map47	F143	F01	Shortage o	Bad experi	0.8
Map47	F143	F63	Shortage o	Increased (	0.8
Map47	F143	F96	Shortage o	More hung	0.8
Map47	F143	F78	Shortage o	Less food t	0.8
Map47	F142	F01	School/ma	Bad experi	0.6
Map47	F142	F69	School/ma	Increased t	0.6
Map47	F142	F158	School/ma	Youth/child	0.8
Map47	F142	F16	School/ma	Delay in ec	0.6
Map47	F140	F83	Restriction	Malnutritior	0.6
Map47	F140	F01	Restriction	Bad experi	0.6
Map47	F140	F63	Restriction	Increased (	0.6
Map47	F140	F96	Restriction	More hung	0.6
Map47	F140	F78	Restriction	Less food t	0.6
Map47	F140	F143	Restriction	Shortage o	0.6
Map47	F140	F120	Restriction	No transpo	0.8
Map47	F147	F01	Stress and	Bad experi	0.8
Map47	F120	F83	No transpo	Malnutritior	0.6
Map47	F120	F01	No transpo	Bad experi	0.6
Map47	F120	F63	No transpo	Increased (	0.6
Map47	F120	F96	No transpo	More hung	0.6
Map47	F120	F78	No transpo	Less food t	0.6
Map47	F120	F143	No transpo	Shortage o	0.6
Map47	F100	F01	Mosques/c	Bad experi	0.8
Map47	F100	F115	Mosques/c	No preachi	1
Map47	F100	F147	Mosques/c	Stress and	0.8
Map48	F40	F47	Household	Increase in	0.4
Map48	F40	F01	Household	Bad experi	0.8
Map48	F88	F40	Men unabl	Household	0.6
Map48	F88	F47	Men unabl	Increase in	0.4
Map48	F88	F01	Men unabl	Bad experi	0.6
Map48	F47	F01	Increase in	Bad experi	0.6
Map48	F85	F40	Markets/sh	Household	0.6
Map48	F85	F88	Markets/sh	Men unabl	0.6
Map48	F85	F47	Markets/sh	Increase in	0.4
Map48	F85	F07	Markets/sh	Businesses	0.8
Map48	F85	F01	Markets/sh	Bad experi	0.8
Map48	F85	F64	Markets/sh	Increased		0.8
Map48	F85	F96	Markets/sh	More hung	0.6
Map48	F85	F132	Markets/sh	Poor harve	0.6
Map48	F85	F78	Markets/sh	Less food t	0.6
Map48	F85	F101	Markets/sh	No access	0.8
Map48	F80	F40	Lockdown	Household	0.6
Map48	F80	F88	Lockdown	Men unabl	0.6
Map48	F80	F47	Lockdown	Increase in	0.4
Map48	F80	F85	Lockdown	Markets/sh	0.6
Map48	F80	F07	Lockdown	Businesses	0.6
Map48	F80	F01	Lockdown	Bad experi	0.8
Map48	F80	F67	Lockdown	Increased s	0.6
Map48	F80	F64	Lockdown	Increased		0.6
Map48	F80	F60	Lockdown	Increased i	0.6
Map48	F80	F158	Lockdown	Youth/child	0.8
Map48	F80	F70	Lockdown	Jobs lost	0.4
Map48	F80	F96	Lockdown	More hung	0.6
Map48	F80	F132	Lockdown	Poor harve	0.6
Map48	F80	F133	Lockdown	Poor perfor	0.6

For Peer Review

Map48	F80	F51	Lockdown	Increase in	0.4
Map48	F80	F78	Lockdown	Less food t	0.6
Map48	F80	F16	Lockdown	Delay in ec	0.8
Map48	F80	F142	Lockdown	School/mac	0.8
Map48	F80	F147	Lockdown	Stress and	0.8
Map48	F80	F120	Lockdown	No transpo	0.6
Map48	F80	F106	Lockdown	No congreg	0.8
Map48	F80	F101	Lockdown	No access	0.6
Map48	F80	F100	Lockdown	Mosques/c	0.8
Map48	F07	F40	Businesses	Household	0.6
Map48	F07	F88	Businesses	Men unable	0.6
Map48	F07	F47	Businesses	Increase in	0.4
Map48	F07	F01	Businesses	Bad experi	0.8
Map48	F07	F64	Businesses	Increased p	0.8
Map48	F20	F01	Difficult bre	Bad experi	0.4
Map48	F67	F01	Increased s	Bad experi	1
Map48	F67	F147	Increased s	Stress and	1
Map48	F29	F20	Fear of cor	Difficult bre	0.2
Map48	F29	F01	Fear of cor	Bad experi	0.8
Map48	F29	F63	Fear of cor	Increased c	0.8
Map48	F29	F31	Fear of cor	Fear of eac	0.6
Map48	F29	F38	Fear of cor	Health wor	0.8
Map48	F29	F10	Fear of cor	Compulsor	0.6
Map48	F64	F01	Increased p	Bad experi	1
Map48	F63	F01	Increased c	Bad experi	1
Map48	F60	F01	Increased i	Bad experi	0.8
Map48	F60	F67	Increased i	Increased s	0.8
Map48	F60	F147	Increased i	Stress and	0.8
Map48	F158	F01	Youth/child	Bad experi	0.6
Map48	F158	F67	Youth/child	Increased s	0.6
Map48	F158	F60	Youth/child	Increased i	0.6
Map48	F158	F133	Youth/child	Poor perfor	0.6
Map48	F158	F51	Youth/child	Increase in	0.4
Map48	F158	F16	Youth/child	Delay in ec	0.8
Map48	F158	F147	Youth/child	Stress and	0.6
Map48	F70	F40	Jobs lost	Household	0.6
Map48	F70	F88	Jobs lost	Men unable	0.6
Map48	F70	F47	Jobs lost	Increase in	0.4
Map48	F70	F01	Jobs lost	Bad experi	0.6
Map48	F96	F01	More hung	Bad experi	1
Map48	F132	F01	Poor harve	Bad experi	0.6
Map48	F132	F96	Poor harve	More hung	0.6
Map48	F132	F78	Poor harve	Less food t	0.6
Map48	F133	F01	Poor perfor	Bad experi	0.4
Map48	F133	F67	Poor perfor	Increased s	0.4
Map48	F133	F60	Poor perfor	Increased i	0.4
Map48	F133	F51	Poor perfor	Increase in	0.4
Map48	F133	F147	Poor perfor	Stress and	0.4
Map48	F51	F01	Increase in	Bad experi	0.8
Map48	F51	F67	Increase in	Increased s	0.8
Map48	F51	F60	Increase in	Increased i	0.8
Map48	F51	F147	Increase in	Stress and	0.8
Map48	F31	F20	Fear of eac	Difficult bre	0.2
Map48	F31	F01	Fear of eac	Bad experi	0.2
Map48	F31	F10	Fear of eac	Compulsor	0.6
Map48	F78	F01	Less food t	Bad experi	0.8

For Peer Review

Map48	F78	F96	Less food t	More hung	0.8
Map48	F38	F01	Health wor	Bad experi	0.8
Map48	F38	F63	Health wor	Increased c	0.8
Map48	F10	F20	Compulsor	Difficult bre	0.2
Map48	F10	F01	Compulsor	Bad experi	0.2
Map48	F16	F01	Delay in ec	Bad experi	0.4
Map48	F16	F67	Delay in ec	Increased s	0.4
Map48	F16	F60	Delay in ec	Increased i	0.4
Map48	F16	F133	Delay in ec	Poor perfor	0.6
Map48	F16	F51	Delay in ec	Increase in	0.4
Map48	F16	F147	Delay in ec	Stress and	0.4
Map48	F142	F01	School/ma	Bad experi	0.6
Map48	F142	F67	School/ma	Increased s	0.6
Map48	F142	F60	School/ma	Increased i	0.6
Map48	F142	F158	School/ma	Youth/child	0.8
Map48	F142	F133	School/ma	Poor perfor	0.6
Map48	F142	F51	School/ma	Increase in	0.4
Map48	F142	F16	School/ma	Delay in ec	0.8
Map48	F142	F147	School/ma	Stress and	0.6
Map48	F147	F01	Stress and	Bad experi	1
Map48	F120	F40	No transpo	Household	0.4
Map48	F120	F88	No transpo	Men unable	0.4
Map48	F120	F47	No transpo	Increase in	0.4
Map48	F120	F01	No transpo	Bad experi	0.4
Map48	F120	F70	No transpo	Jobs lost	0.4
Map48	F106	F01	No congreg	Bad experi	1
Map48	F106	F147	No congreg	Stress and	1
Map48	F101	F01	No access	Bad experi	0.6
Map48	F101	F96	No access	More hung	0.6
Map48	F101	F132	No access	Poor harve	0.6
Map48	F101	F78	No access	Less food t	0.6
Map48	F100	F01	Mosques/c	Bad experi	1
Map48	F100	F147	Mosques/c	Stress and	1
Map48	F100	F106	Mosques/c	No congreg	1
Map49	F40	F47	Household	Increase in	0.4
Map49	F40	F01	Household	Bad experi	0.4
Map49	F47	F01	Increase in	Bad experi	0.6
Map49	F85	F40	Markets/sh	Household	0.6
Map49	F85	F47	Markets/sh	Increase in	0.4
Map49	F85	F87	Markets/sh	Men stayin	0.6
Map49	F85	F83	Markets/sh	Malnutritior	0.6
Map49	F85	F82	Markets/sh	Lost invest	0.6
Map49	F85	F07	Markets/sh	Businesses	1
Map49	F85	F01	Markets/sh	Bad experi	0.6
Map49	F85	F64	Markets/sh	Increased p	0.6
Map49	F85	F63	Markets/sh	Increased c	0.4
Map49	F85	F96	Markets/sh	More hung	0.6
Map49	F85	F78	Markets/sh	Less food t	0.6
Map49	F87	F40	Men stayin	Household	0.6
Map49	F87	F47	Men stayin	Increase in	0.4
Map49	F87	F01	Men stayin	Bad experi	0.4
Map49	F80	F40	Lockdown	Household	0.6
Map49	F80	F47	Lockdown	Increase in	0.4
Map49	F80	F85	Lockdown	Markets/sh	1
Map49	F80	F87	Lockdown	Men stayin	0.6
Map49	F80	F83	Lockdown	Malnutritior	0.6

For Peer Review

Map49	F80	F82	Lockdown	Lost invest	0.6
Map49	F80	F07	Lockdown	Businesses	1
Map49	F80	F01	Lockdown	Bad experi	0.6
Map49	F80	F64	Lockdown	Increased p	0.6
Map49	F80	F63	Lockdown	Increased c	0.6
Map49	F80	F158	Lockdown	Youth/child	1
Map49	F80	F96	Lockdown	More hung	0.6
Map49	F80	F135	Lockdown	Price hike	1
Map49	F80	F142	Lockdown	School/ma	1
Map49	F80	F78	Lockdown	Less food t	0.6
Map49	F80	F13	Lockdown	Could not c	0.6
Map49	F80	F14	Lockdown	Could not g	0.8
Map49	F80	F143	Lockdown	Shortage o	0.6
Map49	F80	F124	Lockdown	Not visiting	0.8
Map49	F80	F147	Lockdown	Stress and	0.4
Map49	F80	F120	Lockdown	No transpo	0.8
Map49	F83	F01	Malnutrition	Bad experi	0.4
Map49	F83	F63	Malnutrition	Increased c	0.4
Map49	F82	F83	Lost invest	Malnutrition	0.6
Map49	F82	F01	Lost invest	Bad experi	0.8
Map49	F82	F64	Lost invest	Increased p	0.8
Map49	F82	F63	Lost invest	Increased c	0.4
Map49	F82	F96	Lost invest	More hung	0.8
Map49	F82	F78	Lost invest	Less food t	0.8
Map49	F07	F83	Businesses	Malnutrition	0.6
Map49	F07	F82	Businesses	Lost invest	0.6
Map49	F07	F01	Businesses	Bad experi	0.6
Map49	F07	F64	Businesses	Increased p	0.6
Map49	F07	F63	Businesses	Increased c	0.4
Map49	F07	F96	Businesses	More hung	0.6
Map49	F07	F78	Businesses	Less food t	0.6
Map49	F29	F01	Fear of cor	Bad experi	0.4
Map49	F29	F31	Fear of cor	Fear of eac	0.8
Map49	F64	F83	Increased p	Malnutrition	0.6
Map49	F64	F01	Increased p	Bad experi	0.8
Map49	F64	F63	Increased p	Increased c	0.4
Map49	F64	F96	Increased p	More hung	0.8
Map49	F64	F78	Increased p	Less food t	0.8
Map49	F63	F01	Increased c	Bad experi	0.6
Map49	F158	F01	Youth/child	Bad experi	0.4
Map49	F158	F13	Youth/child	Could not c	0.6
Map49	F158	F147	Youth/child	Stress and	0.4
Map49	F118	F01	No social g	Bad experi	0.2
Map49	F118	F32	No social g	Feeling soc	0.2
Map49	F96	F83	More hung	Malnutrition	0.6
Map49	F96	F01	More hung	Bad experi	0.6
Map49	F96	F63	More hung	Increased c	0.4
Map49	F135	F83	Price hike	Malnutrition	0.6
Map49	F135	F01	Price hike	Bad experi	0.6
Map49	F135	F63	Price hike	Increased c	0.4
Map49	F135	F96	Price hike	More hung	0.6
Map49	F135	F143	Price hike	Shortage o	0.6
Map49	F142	F01	School/ma	Bad experi	0.4
Map49	F142	F158	School/ma	Youth/child	1
Map49	F142	F13	School/ma	Could not c	0.6
Map49	F142	F147	School/ma	Stress and	0.4

For Peer Review

Map49	F31	F01	Fear of eac	Bad experi	0.4
Map49	F32	F01	Feeling soc	Bad experi	0.2
Map49	F78	F83	Less food t	Malnutrition	0.6
Map49	F78	F01	Less food t	Bad experi	0.8
Map49	F78	F63	Less food t	Increased (	0.4
Map49	F78	F96	Less food t	More hung	1
Map49	F13	F01	Could not c	Bad experi	0.4
Map49	F13	F147	Could not c	Stress and	0.4
Map49	F14	F01	Could not g	Bad experi	0.6
Map49	F14	F63	Could not g	Increased (	0.6
Map49	F143	F83	Shortage o	Malnutrition	0.6
Map49	F143	F01	Shortage o	Bad experi	0.6
Map49	F143	F63	Shortage o	Increased (	0.4
Map49	F143	F96	Shortage o	More hung	0.8
Map49	F124	F01	Not visiting	Bad experi	0.6
Map49	F124	F63	Not visiting	Increased (	0.6
Map49	F124	F14	Not visiting	Could not g	0.8
Map49	F147	F01	Stress and	Bad experi	1
Map49	F120	F01	No transpo	Bad experi	0.6
Map49	F120	F63	No transpo	Increased (	0.6
Map49	F120	F14	No transpo	Could not g	0.8
Map49	F120	F124	No transpo	Not visiting	0.8
Map49	F144	F01	Social dista	Bad experi	0.2
Map49	F144	F118	Social dista	No social g	0.4
Map49	F144	F32	Social dista	Feeling soc	0.2
Map50	F40	F47	Household	Increase in	0.6
Map50	F40	F01	Household	Bad experi	0.4
Map50	F42	F01	Immunizati	Bad experi	0.4
Map50	F42	F63	Immunizati	Increased (	0.4
Map50	F47	F01	Increase in	Bad experi	0.4
Map50	F85	F83	Markets/sh	Malnutrition	0.6
Map50	F85	F82	Markets/sh	Lost invest	0.8
Map50	F85	F07	Markets/sh	Businesses	0.8
Map50	F85	F01	Markets/sh	Bad experi	0.8
Map50	F85	F75	Markets/sh	Lack of hyg	0.6
Map50	F85	F64	Markets/sh	Increased p	0.8
Map50	F85	F63	Markets/sh	Increased (	0.6
Map50	F85	F78	Markets/sh	Less food t	0.8
Map50	F85	F72	Markets/sh	Lack of bas	0.8
Map50	F87	F40	Men stayin	Household	0.8
Map50	F87	F47	Men stayin	Increase in	0.6
Map50	F87	F01	Men stayin	Bad experi	0.4
Map50	F81	F01	Loss of Ha	Bad experi	0.6
Map50	F81	F62	Loss of Ha	Increased r	0.6
Map50	F80	F40	Lockdown	Household	0.6
Map50	F80	F47	Lockdown	Increase in	0.6
Map50	F80	F85	Lockdown	Markets/sh	1
Map50	F80	F87	Lockdown	Men stayin	0.6
Map50	F80	F81	Lockdown	Loss of Ha	0.2
Map50	F80	F83	Lockdown	Malnutrition	0.6
Map50	F80	F82	Lockdown	Lost invest	0.8
Map50	F80	F07	Lockdown	Businesses	0.8
Map50	F80	F01	Lockdown	Bad experi	0.8
Map50	F80	F75	Lockdown	Lack of hyg	0.6
Map50	F80	F64	Lockdown	Increased p	0.8
Map50	F80	F63	Lockdown	Increased (	0.6

For Peer Review

Map50	F80	F62	Lockdown	Increased r	0.2
Map50	F80	F16	Lockdown	Delay in ec	0.6
Map50	F80	F114	Lockdown	No pilgrima	0.2
Map50	F80	F112	Lockdown	No home/p	0.8
Map50	F80	F135	Lockdown	Price hike	0.8
Map50	F80	F78	Lockdown	Less food t	0.8
Map50	F80	F13	Lockdown	Could not c	0.6
Map50	F80	F70	Lockdown	Jobs lost	0.6
Map50	F80	F72	Lockdown	Lack of bas	0.8
Map50	F80	F143	Lockdown	Shortage o	0.8
Map50	F80	F142	Lockdown	School/mad	0.8
Map50	F80	F140	Lockdown	Restriction	0.8
Map50	F80	F147	Lockdown	Stress and	0.6
Map50	F80	F120	Lockdown	No transpo	0.8
Map50	F80	F106	Lockdown	No congreg	0.6
Map50	F80	F104	Lockdown	No buyers	0.8
Map50	F80	F100	Lockdown	Mosques/c	0.8
Map50	F83	F01	Malnutritior	Bad experi	0.8
Map50	F82	F83	Lost invest	Malnutritior	0.6
Map50	F82	F01	Lost invest	Bad experi	1
Map50	F82	F75	Lost invest	Lack of hyg	0.6
Map50	F82	F64	Lost invest	Increased p	1
Map50	F82	F63	Lost invest	Increased c	0.6
Map50	F82	F78	Lost invest	Less food t	0.8
Map50	F82	F72	Lost invest	Lack of bas	1
Map50	F07	F83	Businesses	Malnutritior	0.6
Map50	F07	F82	Businesses	Lost invest	0.8
Map50	F07	F01	Businesses	Bad experi	1
Map50	F07	F75	Businesses	Lack of hyg	0.6
Map50	F07	F64	Businesses	Increased p	1
Map50	F07	F63	Businesses	Increased c	0.6
Map50	F07	F78	Businesses	Less food t	0.8
Map50	F07	F72	Businesses	Lack of bas	1
Map50	F75	F01	Lack of hyg	Bad experi	0.8
Map50	F75	F63	Lack of hyg	Increased c	0.8
Map50	F29	F42	Fear of cor	Immunizati	0.6
Map50	F29	F01	Fear of cor	Bad experi	0.8
Map50	F29	F63	Fear of cor	Increased c	0.6
Map50	F29	F14	Fear of cor	Could not g	0.6
Map50	F29	F124	Fear of cor	Not visiting	0.4
Map50	F29	F31	Fear of cor	Fear of eac	0.8
Map50	F29	F38	Fear of cor	Health wor	0.6
Map50	F64	F83	Increased p	Malnutritior	0.6
Map50	F64	F01	Increased p	Bad experi	1
Map50	F64	F75	Increased p	Lack of hyg	0.6
Map50	F64	F63	Increased p	Increased c	0.6
Map50	F64	F78	Increased p	Less food t	0.8
Map50	F64	F72	Increased p	Lack of bas	1
Map50	F63	F01	Increased c	Bad experi	1
Map50	F62	F01	Increased r	Bad experi	0.8
Map50	F16	F01	Delay in ec	Bad experi	0.8
Map50	F16	F147	Delay in ec	Stress and	0.8
Map50	F118	F01	No social g	Bad experi	0.6
Map50	F118	F147	No social g	Stress and	0.6
Map50	F118	F121	No social g	No wedding	0.6
Map50	F14	F01	Could not g	Bad experi	0.8

For Peer Review

Map50	F14	F63	Could not g	Increased c	0.8
Map50	F114	F81	No pilgrima	Loss of Ha	0.4
Map50	F114	F01	No pilgrima	Bad experi	0.4
Map50	F114	F62	No pilgrima	Increased r	0.4
Map50	F112	F83	No home/p	Malnutrition	0.6
Map50	F112	F82	No home/p	Lost invest	0.6
Map50	F112	F01	No home/p	Bad experi	0.6
Map50	F112	F75	No home/p	Lack of hyg	0.6
Map50	F112	F64	No home/p	Increased p	0.6
Map50	F112	F63	No home/p	Increased c	0.6
Map50	F112	F78	No home/p	Less food t	0.6
Map50	F112	F72	No home/p	Lack of bas	0.6
Map50	F135	F83	Price hike	Malnutrition	0.6
Map50	F135	F01	Price hike	Bad experi	0.8
Map50	F135	F78	Price hike	Less food t	0.8
Map50	F124	F01	Not visiting	Bad experi	0.6
Map50	F124	F63	Not visiting	Increased c	0.6
Map50	F124	F14	Not visiting	Could not g	0.6
Map50	F31	F01	Fear of eac	Bad experi	0.8
Map50	F78	F83	Less food t	Malnutrition	0.6
Map50	F78	F01	Less food t	Bad experi	1
Map50	F38	F42	Health wor	Immunizati	0.6
Map50	F38	F01	Health wor	Bad experi	0.6
Map50	F38	F63	Health wor	Increased c	0.6
Map50	F38	F14	Health wor	Could not g	0.6
Map50	F13	F01	Could not c	Bad experi	0.4
Map50	F13	F147	Could not c	Stress and	0.4
Map50	F70	F83	Jobs lost	Malnutrition	0.6
Map50	F70	F01	Jobs lost	Bad experi	0.8
Map50	F70	F75	Jobs lost	Lack of hyg	0.6
Map50	F70	F64	Jobs lost	Increased p	0.8
Map50	F70	F63	Jobs lost	Increased c	0.6
Map50	F70	F78	Jobs lost	Less food t	0.8
Map50	F70	F72	Jobs lost	Lack of bas	0.8
Map50	F72	F01	Lack of bas	Bad experi	0.6
Map50	F72	F75	Lack of bas	Lack of hyg	0.6
Map50	F72	F63	Lack of bas	Increased c	0.6
Map50	F143	F83	Shortage o	Malnutrition	0.6
Map50	F143	F01	Shortage o	Bad experi	0.8
Map50	F143	F135	Shortage o	Price hike	0.8
Map50	F143	F78	Shortage o	Less food t	0.8
Map50	F142	F01	School/ma	Bad experi	0.6
Map50	F142	F16	School/ma	Delay in ec	0.6
Map50	F142	F13	School/ma	Could not c	0.6
Map50	F142	F147	School/ma	Stress and	0.6
Map50	F140	F83	Restriction	Malnutrition	0.6
Map50	F140	F82	Restriction	Lost invest	0.6
Map50	F140	F01	Restriction	Bad experi	0.6
Map50	F140	F75	Restriction	Lack of hyg	0.6
Map50	F140	F64	Restriction	Increased p	0.6
Map50	F140	F63	Restriction	Increased c	0.6
Map50	F140	F112	Restriction	No home/p	0.8
Map50	F140	F78	Restriction	Less food t	0.6
Map50	F140	F72	Restriction	Lack of bas	0.6
Map50	F140	F104	Restriction	No buyers	0.8
Map50	F147	F01	Stress and	Bad experi	1

For Peer Review

Map50	F120	F83	No transpo	Malnutritior	0.6
Map50	F120	F82	No transpo	Lost invest	0.6
Map50	F120	F01	No transpo	Bad experi	0.8
Map50	F120	F75	No transpo	Lack of hyg	0.6
Map50	F120	F64	No transpo	Increased	0.6
Map50	F120	F63	No transpo	Increased	0.6
Map50	F120	F112	No transpo	No home/p	0.6
Map50	F120	F135	No transpo	Price hike	0.8
Map50	F120	F78	No transpo	Less food t	0.8
Map50	F120	F72	No transpo	Lack of bas	0.6
Map50	F120	F143	No transpo	Shortage o	1
Map50	F120	F140	No transpo	Restriction	0.6
Map50	F120	F104	No transpo	No buyers	0.6
Map50	F144	F01	Social dista	Bad experi	0.6
Map50	F144	F118	Social dista	No social g	0.8
Map50	F144	F147	Social dista	Stress and	0.6
Map50	F144	F121	Social dista	No wedding	0.6
Map50	F121	F01	No wedding	Bad experi	0.8
Map50	F121	F147	No wedding	Stress and	0.8
Map50	F106	F01	No congreg	Bad experi	0.4
Map50	F106	F147	No congreg	Stress and	0.4
Map50	F104	F83	No buyers	Malnutritior	0.6
Map50	F104	F82	No buyers	Lost invest	0.6
Map50	F104	F01	No buyers	Bad experi	0.6
Map50	F104	F75	No buyers	Lack of hyg	0.6
Map50	F104	F64	No buyers	Increased	0.6
Map50	F104	F63	No buyers	Increased	0.6
Map50	F104	F112	No buyers	No home/p	0.8
Map50	F104	F78	No buyers	Less food t	0.6
Map50	F104	F72	No buyers	Lack of bas	0.6
Map50	F100	F01	Mosques/c	Bad experi	0.4
Map50	F100	F147	Mosques/c	Stress and	0.4
Map50	F100	F106	Mosques/c	No congreg	0.6
Map51	F40	F47	Household	Increase in	0.6
Map51	F40	F01	Household	Bad experi	0.6
Map51	F47	F01	Increase in	Bad experi	1
Map51	F85	F40	Markets/sh	Household	0.6
Map51	F85	F47	Markets/sh	Increase in	0.6
Map51	F85	F87	Markets/sh	Men stayin	0.6
Map51	F85	F82	Markets/sh	Lost invest	0.6
Map51	F85	F07	Markets/sh	Businesses	0.8
Map51	F85	F01	Markets/sh	Bad experi	0.6
Map51	F85	F64	Markets/sh	Increased	0.6
Map51	F85	F62	Markets/sh	Increased	0.6
Map51	F85	F118	Markets/sh	No social g	0.6
Map51	F85	F75	Markets/sh	Lack of hyg	0.6
Map51	F85	F70	Markets/sh	Jobs lost	0.8
Map51	F85	F72	Markets/sh	Lack of bas	0.6
Map51	F85	F147	Markets/sh	Stress and	0.6
Map51	F85	F106	Markets/sh	No congreg	0.6
Map51	F87	F40	Men stayin	Household	0.6
Map51	F87	F47	Men stayin	Increase in	0.6
Map51	F87	F01	Men stayin	Bad experi	0.6
Map51	F80	F40	Lockdown	Household	0.6
Map51	F80	F47	Lockdown	Increase in	0.6
Map51	F80	F85	Lockdown	Markets/sh	0.8

For Peer Review

Map51	F80	F87	Lockdown	Men stayin	0.6
Map51	F80	F83	Lockdown	Malnutritior	0.6
Map51	F80	F82	Lockdown	Lost invest	0.6
Map51	F80	F07	Lockdown	Businesses	0.8
Map51	F80	F01	Lockdown	Bad experi	0.8
Map51	F80	F64	Lockdown	Increased p	0.6
Map51	F80	F63	Lockdown	Increased c	0.6
Map51	F80	F62	Lockdown	Increased r	0.8
Map51	F80	F158	Lockdown	Youth/child	0.4
Map51	F80	F16	Lockdown	Delay in ec	0.4
Map51	F80	F96	Lockdown	More hung	0.6
Map51	F80	F133	Lockdown	Poor perfor	0.4
Map51	F80	F135	Lockdown	Price hike	0.6
Map51	F80	F118	Lockdown	No social g	0.6
Map51	F80	F78	Lockdown	Less food t	0.6
Map51	F80	F75	Lockdown	Lack of hyg	0.6
Map51	F80	F70	Lockdown	Jobs lost	0.8
Map51	F80	F72	Lockdown	Lack of bas	0.6
Map51	F80	F143	Lockdown	Shortage o	0.6
Map51	F80	F142	Lockdown	School/mac	0.5
Map51	F80	F147	Lockdown	Stress and	0.8
Map51	F80	F120	Lockdown	No transpo	0.8
Map51	F80	F106	Lockdown	No congreg	1
Map51	F83	F01	Malnutritior	Bad experi	0.6
Map51	F82	F47	Lost invest	Increase in	0.6
Map51	F82	F01	Lost invest	Bad experi	1
Map51	F82	F64	Lost invest	Increased p	1
Map51	F82	F62	Lost invest	Increased r	0.6
Map51	F82	F118	Lost invest	No social g	0.6
Map51	F82	F147	Lost invest	Stress and	0.6
Map51	F82	F106	Lost invest	No congreg	0.6
Map51	F07	F40	Businesses	Household	0.6
Map51	F07	F47	Businesses	Increase in	0.6
Map51	F07	F87	Businesses	Men stayin	0.6
Map51	F07	F82	Businesses	Lost invest	0.6
Map51	F07	F01	Businesses	Bad experi	0.6
Map51	F07	F64	Businesses	Increased p	0.6
Map51	F07	F62	Businesses	Increased r	0.6
Map51	F07	F118	Businesses	No social g	0.6
Map51	F07	F75	Businesses	Lack of hyg	0.6
Map51	F07	F70	Businesses	Jobs lost	0.8
Map51	F07	F72	Businesses	Lack of bas	0.6
Map51	F07	F147	Businesses	Stress and	0.6
Map51	F07	F106	Businesses	No congreg	0.6
Map51	F29	F01	Fear of cor	Bad experi	0.4
Map51	F29	F62	Fear of cor	Increased r	0.4
Map51	F29	F30	Fear of cor	Fear of dea	0.6
Map51	F29	F32	Fear of cor	Feeling soc	0.4
Map51	F29	F144	Fear of cor	Social dista	0.4
Map51	F64	F47	Increased p	Increase in	0.6
Map51	F64	F01	Increased p	Bad experi	1
Map51	F64	F62	Increased p	Increased r	0.6
Map51	F64	F118	Increased p	No social g	0.6
Map51	F64	F147	Increased p	Stress and	0.6
Map51	F64	F106	Increased p	No congreg	0.6
Map51	F63	F01	Increased c	Bad experi	0.8

For Peer Review

Map51	F62	F01	Increased r	Bad experi	0.8
Map51	F158	F01	Youth/child	Bad experi	0.6
Map51	F158	F16	Youth/child	Delay in ec	0.6
Map51	F158	F133	Youth/child	Poor perfor	0.6
Map51	F16	F01	Delay in ec	Bad experi	0.8
Map51	F96	F01	More hung	Bad experi	1
Map51	F96	F63	More hung	Increased c	0.8
Map51	F133	F01	Poor perfor	Bad experi	0.8
Map51	F135	F83	Price hike	Malnutrition	0.6
Map51	F135	F01	Price hike	Bad experi	0.6
Map51	F135	F63	Price hike	Increased c	0.6
Map51	F135	F96	Price hike	More hung	0.6
Map51	F135	F78	Price hike	Less food t	0.6
Map51	F30	F01	Fear of dea	Bad experi	0.2
Map51	F31	F01	Fear of eac	Bad experi	0.4
Map51	F31	F29	Fear of eac	Fear of cor	0.4
Map51	F31	F62	Fear of eac	Increased r	0.4
Map51	F31	F30	Fear of eac	Fear of dea	0.4
Map51	F31	F32	Fear of eac	Feeling soc	0.4
Map51	F31	F144	Fear of eac	Social dista	0.4
Map51	F32	F01	Feeling soc	Bad experi	0.6
Map51	F32	F62	Feeling soc	Increased r	0.6
Map51	F118	F01	No social g	Bad experi	1
Map51	F118	F62	No social g	Increased r	0.8
Map51	F118	F147	No social g	Stress and	0.8
Map51	F118	F106	No social g	No congreg	1
Map51	F78	F83	Less food t	Malnutrition	0.6
Map51	F78	F01	Less food t	Bad experi	0.8
Map51	F78	F63	Less food t	Increased c	0.8
Map51	F78	F96	Less food t	More hung	0.8
Map51	F75	F01	Lack of hyg	Bad experi	0.4
Map51	F70	F40	Jobs lost	Household	0.6
Map51	F70	F47	Jobs lost	Increase in	0.6
Map51	F70	F87	Jobs lost	Men stayin	0.6
Map51	F70	F01	Jobs lost	Bad experi	0.6
Map51	F70	F64	Jobs lost	Increased p	0.6
Map51	F70	F62	Jobs lost	Increased r	0.6
Map51	F70	F118	Jobs lost	No social g	0.6
Map51	F70	F147	Jobs lost	Stress and	0.6
Map51	F70	F106	Jobs lost	No congreg	0.6
Map51	F72	F01	Lack of bas	Bad experi	0.4
Map51	F72	F75	Lack of bas	Lack of hyg	0.6
Map51	F143	F83	Shortage o	Malnutrition	0.6
Map51	F143	F01	Shortage o	Bad experi	0.6
Map51	F143	F63	Shortage o	Increased c	0.6
Map51	F143	F62	Shortage o	Increased r	0.6
Map51	F143	F96	Shortage o	More hung	0.6
Map51	F143	F135	Shortage o	Price hike	0.8
Map51	F143	F78	Shortage o	Less food t	0.6
Map51	F143	F143	Shortage o	Shortage o	1
Map51	F143	F147	Shortage o	Stress and	0.6
Map51	F142	F01	School/ma	Bad experi	0.4
Map51	F142	F158	School/ma	Youth/child	0.4
Map51	F142	F16	School/ma	Delay in ec	0.4
Map51	F142	F133	School/ma	Poor perfor	0.4
Map51	F147	F01	Stress and	Bad experi	0.8

For Peer Review

Map51	F147	F62	Stress and	Increased r	0.8
Map51	F120	F83	No transpo	Malnutritior	0.6
Map51	F120	F01	No transpo	Bad experi	0.6
Map51	F120	F63	No transpo	Increased c	0.6
Map51	F120	F62	No transpo	Increased r	0.6
Map51	F120	F96	No transpo	More hung	0.6
Map51	F120	F135	No transpo	Price hike	0.6
Map51	F120	F78	No transpo	Less food t	0.6
Map51	F120	F143	No transpo	Shortage o	0.6
Map51	F120	F147	No transpo	Stress and	0.6
Map51	F144	F01	Social dista	Bad experi	0.4
Map51	F144	F62	Social dista	Increased r	0.4
Map51	F144	F32	Social dista	Feeling soc	0.4
Map51	F106	F01	No congreg	Bad experi	0.8
Map51	F106	F62	No congreg	Increased r	0.8
Map51	F106	F147	No congreg	Stress and	0.8
Map52	F40	F01	Household	Bad experi	0.4
Map52	F88	F40	Men unable	Household	0.6
Map52	F88	F01	Men unable	Bad experi	0.4
Map52	F85	F82	Markets/sh	Lost invest	0.6
Map52	F85	F07	Markets/sh	Businesses	0.6
Map52	F85	F01	Markets/sh	Bad experi	0.6
Map52	F85	F64	Markets/sh	Increased p	0.6
Map52	F85	F63	Markets/sh	Increased c	0.6
Map52	F85	F118	Markets/sh	No social g	0.4
Map52	F85	F96	Markets/sh	More hung	0.6
Map52	F85	F32	Markets/sh	Feeling soc	0.4
Map52	F85	F78	Markets/sh	Less food t	0.6
Map52	F87	F40	Men stayin	Household	0.6
Map52	F87	F88	Men stayin	Men unable	0.6
Map52	F87	F01	Men stayin	Bad experi	0.4
Map52	F87	F98	Men stayin	More mate	0.4
Map52	F87	F99	Men stayin	More unwa	0.6
Map52	F80	F40	Lockdown	Household	0.6
Map52	F80	F88	Lockdown	Men unable	0.6
Map52	F80	F85	Lockdown	Markets/sh	0.8
Map52	F80	F87	Lockdown	Men stayin	0.6
Map52	F80	F82	Lockdown	Lost invest	0.6
Map52	F80	F07	Lockdown	Businesses	0.6
Map52	F80	F01	Lockdown	Bad experi	0.6
Map52	F80	F64	Lockdown	Increased p	0.6
Map52	F80	F63	Lockdown	Increased c	0.6
Map52	F80	F158	Lockdown	Youth/child	0.8
Map52	F80	F118	Lockdown	No social g	0.4
Map52	F80	F96	Lockdown	More hung	0.6
Map52	F80	F98	Lockdown	More mate	0.4
Map52	F80	F99	Lockdown	More unwa	0.6
Map52	F80	F135	Lockdown	Price hike	0.6
Map52	F80	F32	Lockdown	Feeling soc	0.4
Map52	F80	F78	Lockdown	Less food t	0.6
Map52	F80	F13	Lockdown	Could not c	0.6
Map52	F80	F70	Lockdown	Jobs lost	0.6
Map52	F80	F143	Lockdown	Shortage o	0.6
Map52	F80	F142	Lockdown	School/ma	1
Map52	F80	F140	Lockdown	Restriction	0.6
Map52	F80	F147	Lockdown	Stress and	0.4

For Peer Review

Map52	F80	F120	Lockdown	No transpo	0.6
Map52	F82	F01	Lost invest	Bad experi	0.8
Map52	F82	F64	Lost invest	Increased	0.8
Map52	F82	F63	Lost invest	Increased	0.6
Map52	F82	F118	Lost invest	No social g	0.4
Map52	F82	F96	Lost invest	More hung	0.8
Map52	F82	F32	Lost invest	Feeling soc	0.4
Map52	F82	F78	Lost invest	Less food t	0.8
Map52	F07	F82	Businesses	Lost invest	0.6
Map52	F07	F01	Businesses	Bad experi	0.8
Map52	F07	F64	Businesses	Increased	0.6
Map52	F07	F63	Businesses	Increased	0.6
Map52	F07	F118	Businesses	No social g	0.4
Map52	F07	F96	Businesses	More hung	0.8
Map52	F07	F32	Businesses	Feeling soc	0.4
Map52	F07	F78	Businesses	Less food t	0.8
Map52	F20	F01	Difficult bre	Bad experi	0.2
Map52	F29	F20	Fear of cor	Difficult bre	0.4
Map52	F29	F01	Fear of cor	Bad experi	0.4
Map52	F29	F118	Fear of cor	No social g	0.6
Map52	F29	F30	Fear of cor	Fear of dea	0.6
Map52	F29	F32	Fear of cor	Feeling soc	0.4
Map52	F29	F10	Fear of cor	Compulsor	0.6
Map52	F64	F01	Increased	Bad experi	1
Map52	F64	F63	Increased	Increased	0.6
Map52	F64	F118	Increased	No social g	0.4
Map52	F64	F96	Increased	More hung	0.8
Map52	F64	F32	Increased	Feeling soc	0.4
Map52	F64	F78	Increased	Less food t	0.8
Map52	F63	F01	Increased	Bad experi	0.4
Map52	F158	F01	Youth/child	Bad experi	0.2
Map52	F158	F13	Youth/child	Could not	0.6
Map52	F158	F147	Youth/child	Stress and	0.4
Map52	F118	F01	No social g	Bad experi	0.4
Map52	F118	F32	No social g	Feeling soc	0.4
Map52	F96	F01	More hung	Bad experi	0.8
Map52	F96	F63	More hung	Increased	0.6
Map52	F98	F01	More mate	Bad experi	0.4
Map52	F99	F01	More unwa	Bad experi	0.4
Map52	F99	F98	More unwa	More mate	0.4
Map52	F135	F01	Price hike	Bad experi	1
Map52	F135	F64	Price hike	Increased	1
Map52	F135	F63	Price hike	Increased	0.6
Map52	F135	F118	Price hike	No social g	0.4
Map52	F135	F96	Price hike	More hung	0.8
Map52	F135	F32	Price hike	Feeling soc	0.4
Map52	F135	F78	Price hike	Less food t	0.8
Map52	F30	F01	Fear of dea	Bad experi	0.4
Map52	F32	F01	Feeling soc	Bad experi	0.4
Map52	F78	F01	Less food t	Bad experi	0.8
Map52	F78	F63	Less food t	Increased	0.6
Map52	F78	F96	Less food t	More hung	1
Map52	F13	F01	Could not	Bad experi	0.2
Map52	F13	F147	Could not	Stress and	0.4
Map52	F10	F20	Compulsor	Difficult bre	0.4
Map52	F10	F01	Compulsor	Bad experi	0.2

For Peer Review

Map52	F70	F40	Jobs lost	Household	0.6
Map52	F70	F88	Jobs lost	Men unable	0.6
Map52	F70	F87	Jobs lost	Men stayin	0.6
Map52	F70	F01	Jobs lost	Bad experi	0.4
Map52	F70	F98	Jobs lost	More mate	0.4
Map52	F70	F99	Jobs lost	More unwa	0.6
Map52	F143	F01	Shortage o	Bad experi	0.8
Map52	F143	F64	Shortage o	Increased	0.8
Map52	F143	F63	Shortage o	Increased	0.6
Map52	F143	F118	Shortage o	No social g	0.4
Map52	F143	F96	Shortage o	More hung	0.8
Map52	F143	F135	Shortage o	Price hike	0.8
Map52	F143	F32	Shortage o	Feeling soc	0.4
Map52	F143	F78	Shortage o	Less food t	0.8
Map52	F142	F01	School/ma	Bad experi	0.2
Map52	F142	F158	School/ma	Youth/child	0.8
Map52	F142	F13	School/ma	Could not c	0.6
Map52	F142	F147	School/ma	Stress and	0.4
Map52	F140	F82	Restriction	Lost invest	0.6
Map52	F140	F07	Restriction	Businesses	0.6
Map52	F140	F01	Restriction	Bad experi	0.6
Map52	F140	F64	Restriction	Increased	0.6
Map52	F140	F63	Restriction	Increased	0.6
Map52	F140	F118	Restriction	No social g	0.4
Map52	F140	F96	Restriction	More hung	0.6
Map52	F140	F135	Restriction	Price hike	0.6
Map52	F140	F32	Restriction	Feeling soc	0.4
Map52	F140	F78	Restriction	Less food t	0.6
Map52	F140	F143	Restriction	Shortage o	0.6
Map52	F140	F120	Restriction	No transpo	0.6
Map52	F147	F01	Stress and	Bad experi	0.2
Map52	F120	F82	No transpo	Lost invest	0.6
Map52	F120	F07	No transpo	Businesses	0.8
Map52	F120	F01	No transpo	Bad experi	0.8
Map52	F120	F64	No transpo	Increased	0.8
Map52	F120	F63	No transpo	Increased	0.6
Map52	F120	F118	No transpo	No social g	0.4
Map52	F120	F96	No transpo	More hung	0.8
Map52	F120	F135	No transpo	Price hike	0.8
Map52	F120	F32	No transpo	Feeling soc	0.4
Map52	F120	F78	No transpo	Less food t	0.8
Map52	F120	F143	No transpo	Shortage o	0.8
Map52	F144	F82	Social dista	Lost invest	0.6
Map52	F144	F07	Social dista	Businesses	0.6
Map52	F144	F01	Social dista	Bad experi	0.6
Map52	F144	F64	Social dista	Increased	0.6
Map52	F144	F63	Social dista	Increased	0.6
Map52	F144	F118	Social dista	No social g	0.6
Map52	F144	F96	Social dista	More hung	0.6
Map52	F144	F135	Social dista	Price hike	0.6
Map52	F144	F32	Social dista	Feeling soc	0.4
Map52	F144	F78	Social dista	Less food t	0.6
Map52	F144	F143	Social dista	Shortage o	0.6
Map52	F144	F120	Social dista	No transpo	0.6
Map53	F40	F47	Household	Increase in	0.4
Map53	F40	F01	Household	Bad experi	0.4

For Peer Review

Map53	F47	F01	Increase in	Bad experi	0.6
Map53	F85	F40	Markets/sh	Household	0.4
Map53	F85	F47	Markets/sh	Increase in	0.4
Map53	F85	F87	Markets/sh	Men stayin	0.4
Map53	F85	F82	Markets/sh	Lost invest	0.6
Map53	F85	F07	Markets/sh	Businesses	0.8
Map53	F85	F01	Markets/sh	Bad experi	0.6
Map53	F85	F64	Markets/sh	Increased p	0.6
Map53	F85	F70	Markets/sh	Jobs lost	0.6
Map53	F87	F40	Men stayin	Household	0.6
Map53	F87	F47	Men stayin	Increase in	0.4
Map53	F87	F01	Men stayin	Bad experi	0.4
Map53	F81	F01	Loss of Ha	Bad experi	0.4
Map53	F81	F147	Loss of Ha	Stress and	0.4
Map53	F80	F40	Lockdown	Household	0.6
Map53	F80	F47	Lockdown	Increase in	0.4
Map53	F80	F85	Lockdown	Markets/sh	1
Map53	F80	F87	Lockdown	Men stayin	0.4
Map53	F80	F81	Lockdown	Loss of Ha	0.6
Map53	F80	F83	Lockdown	Malnutrition	0.6
Map53	F80	F82	Lockdown	Lost invest	0.6
Map53	F80	F07	Lockdown	Businesses	0.8
Map53	F80	F01	Lockdown	Bad experi	0.8
Map53	F80	F64	Lockdown	Increased p	0.6
Map53	F80	F63	Lockdown	Increased c	0.6
Map53	F80	F158	Lockdown	Youth/child	0.4
Map53	F80	F16	Lockdown	Delay in ec	0.4
Map53	F80	F119	Lockdown	No tourism	0.6
Map53	F80	F132	Lockdown	Poor harve	0.6
Map53	F80	F96	Lockdown	More hung	0.6
Map53	F80	F135	Lockdown	Price hike	0.6
Map53	F80	F114	Lockdown	No pilgrima	0.8
Map53	F80	F78	Lockdown	Less food t	0.6
Map53	F80	F70	Lockdown	Jobs lost	0.6
Map53	F80	F143	Lockdown	Shortage o	0.6
Map53	F80	F142	Lockdown	School/ma	0.6
Map53	F80	F147	Lockdown	Stress and	0.8
Map53	F80	F120	Lockdown	No transpo	0.6
Map53	F80	F106	Lockdown	No congreg	0.8
Map53	F80	F101	Lockdown	No access	0.6
Map53	F80	F100	Lockdown	Mosques/c	0.8
Map53	F83	F01	Malnutrition	Bad experi	0.6
Map53	F83	F63	Malnutrition	Increased c	0.6
Map53	F82	F01	Lost invest	Bad experi	0.8
Map53	F82	F64	Lost invest	Increased p	0.8
Map53	F07	F40	Businesses	Household	0.4
Map53	F07	F47	Businesses	Increase in	0.4
Map53	F07	F87	Businesses	Men stayin	0.4
Map53	F07	F82	Businesses	Lost invest	0.6
Map53	F07	F01	Businesses	Bad experi	0.6
Map53	F07	F64	Businesses	Increased p	0.6
Map53	F07	F70	Businesses	Jobs lost	0.6
Map53	F20	F01	Difficult bre	Bad experi	0.2
Map53	F29	F20	Fear of cor	Difficult bre	0.4
Map53	F29	F01	Fear of cor	Bad experi	0.8
Map53	F29	F118	Fear of cor	No social g	1

For Peer Review

Map53	F29	F119	Fear of cor	No tourism	0.4
Map53	F29	F30	Fear of cor	Fear of dea	0.6
Map53	F29	F10	Fear of cor	Compulsor	0.4
Map53	F29	F147	Fear of cor	Stress and	1
Map53	F29	F31	Fear of cor	Fear of eac	0.4
Map53	F64	F01	Increased (	Bad experi	1
Map53	F63	F01	Increased (	Bad experi	1
Map53	F158	F01	Youth/child	Bad experi	0.6
Map53	F158	F16	Youth/child	Delay in ec	0.6
Map53	F16	F01	Delay in ec	Bad experi	1
Map53	F118	F01	No social g	Bad experi	0.8
Map53	F118	F147	No social g	Stress and	1
Map53	F119	F01	No tourism	Bad experi	0.4
Map53	F119	F147	No tourism	Stress and	0.4
Map53	F132	F01	Poor harve	Bad experi	1
Map53	F96	F40	More hung	Household	0.8
Map53	F96	F47	More hung	Increase in	0.4
Map53	F96	F83	More hung	Malnutritior	0.6
Map53	F96	F01	More hung	Bad experi	0.8
Map53	F96	F63	More hung	Increased (	0.8
Map53	F111	F40	No governr	Household	0.4
Map53	F111	F47	No governr	Increase in	0.4
Map53	F111	F83	No governr	Malnutritior	0.4
Map53	F111	F01	No governr	Bad experi	0.4
Map53	F111	F63	No governr	Increased (	0.4
Map53	F111	F96	No governr	More hung	0.4
Map53	F111	F78	No governr	Less food t	0.4
Map53	F135	F40	Price hike	Household	0.8
Map53	F135	F47	Price hike	Increase in	0.4
Map53	F135	F83	Price hike	Malnutritior	0.6
Map53	F135	F01	Price hike	Bad experi	1
Map53	F135	F63	Price hike	Increased (	0.8
Map53	F135	F96	Price hike	More hung	0.8
Map53	F135	F78	Price hike	Less food t	0.8
Map53	F30	F01	Fear of dea	Bad experi	0.6
Map53	F114	F81	No pilgrima	Loss of Ha	0.6
Map53	F114	F01	No pilgrima	Bad experi	0.4
Map53	F114	F147	No pilgrima	Stress and	0.4
Map53	F78	F40	Less food t	Household	0.8
Map53	F78	F47	Less food t	Increase in	0.4
Map53	F78	F83	Less food t	Malnutritior	0.6
Map53	F78	F01	Less food t	Bad experi	0.8
Map53	F78	F63	Less food t	Increased (	0.8
Map53	F78	F96	Less food t	More hung	0.8
Map53	F10	F20	Compulsor	Difficult bre	0.6
Map53	F10	F01	Compulsor	Bad experi	0.2
Map53	F70	F40	Jobs lost	Household	0.4
Map53	F70	F47	Jobs lost	Increase in	0.4
Map53	F70	F87	Jobs lost	Men stayin	0.4
Map53	F70	F01	Jobs lost	Bad experi	0.4
Map53	F143	F40	Shortage o	Household	0.8
Map53	F143	F47	Shortage o	Increase in	0.4
Map53	F143	F83	Shortage o	Malnutritior	0.6
Map53	F143	F01	Shortage o	Bad experi	0.8
Map53	F143	F63	Shortage o	Increased (	0.8
Map53	F143	F96	Shortage o	More hung	0.8

For Peer Review

Map53	F143	F135	Shortage o	Price hike	0.8
Map53	F143	F78	Shortage o	Less food t	0.8
Map53	F142	F01	School/ma	Bad experi	0.4
Map53	F142	F158	School/ma	Youth/child	0.4
Map53	F142	F16	School/ma	Delay in ec	0.4
Map53	F147	F01	Stress and	Bad experi	0.8
Map53	F120	F40	No transpo	Household	0.6
Map53	F120	F47	No transpo	Increase in	0.4
Map53	F120	F83	No transpo	Malnutritior	0.6
Map53	F120	F01	No transpo	Bad experi	0.8
Map53	F120	F63	No transpo	Increased (	0.6
Map53	F120	F119	No transpo	No tourism	0.6
Map53	F120	F132	No transpo	Poor harve	0.8
Map53	F120	F96	No transpo	More hung	0.6
Map53	F120	F135	No transpo	Price hike	0.6
Map53	F120	F78	No transpo	Less food t	0.6
Map53	F120	F143	No transpo	Shortage o	0.6
Map53	F120	F147	No transpo	Stress and	0.4
Map53	F120	F101	No transpo	No access	1
Map53	F31	F01	Fear of eac	Bad experi	0.8
Map53	F106	F01	No congreg	Bad experi	0.8
Map53	F106	F147	No congreg	Stress and	0.8
Map53	F101	F01	No access	Bad experi	0.8
Map53	F101	F132	No access	Poor harve	0.8
Map53	F100	F01	Mosques/c	Bad experi	0.8
Map53	F100	F147	Mosques/c	Stress and	0.8
Map53	F100	F106	Mosques/c	No congreg	1
Map54	F40	F47	Household	Increase in	0.6
Map54	F40	F01	Household	Bad experi	0.6
Map54	F88	F40	Men unable	Household	0.6
Map54	F88	F47	Men unable	Increase in	0.6
Map54	F88	F01	Men unable	Bad experi	0.6
Map54	F47	F01	Increase in	Bad experi	0.6
Map54	F85	F40	Markets/sh	Household	0.6
Map54	F85	F88	Markets/sh	Men unable	0.6
Map54	F85	F47	Markets/sh	Increase in	0.6
Map54	F85	F07	Markets/sh	Businesses	0.8
Map54	F85	F01	Markets/sh	Bad experi	0.8
Map54	F85	F64	Markets/sh	Increased p	0.8
Map54	F85	F52	Markets/sh	Increase in	0.2
Map54	F85	F70	Markets/sh	Jobs lost	0.8
Map54	F80	F40	Lockdown	Household	0.6
Map54	F80	F88	Lockdown	Men unable	0.6
Map54	F80	F47	Lockdown	Increase in	0.6
Map54	F80	F85	Lockdown	Markets/sh	0.8
Map54	F80	F83	Lockdown	Malnutritior	0.6
Map54	F80	F07	Lockdown	Businesses	0.8
Map54	F80	F01	Lockdown	Bad experi	0.8
Map54	F80	F64	Lockdown	Increased p	0.8
Map54	F80	F63	Lockdown	Increased (	0.4
Map54	F80	F60	Lockdown	Increased i	0.6
Map54	F80	F158	Lockdown	Youth/child	0.8
Map54	F80	F16	Lockdown	Delay in ec	0.7
Map54	F80	F96	Lockdown	More hung	0.6
Map54	F80	F118	Lockdown	No social g	0.8
Map54	F80	F99	Lockdown	More unwa	0.4

For Peer Review

Map54	F80	F110	Lockdown	No funerals	0.4
Map54	F80	F135	Lockdown	Price hike	0.6
Map54	F80	F52	Lockdown	Increase in	0.2
Map54	F80	F78	Lockdown	Less food t	0.6
Map54	F80	F133	Lockdown	Poor perfor	0.4
Map54	F80	F70	Lockdown	Jobs lost	0.8
Map54	F80	F143	Lockdown	Shortage o	0.6
Map54	F80	F142	Lockdown	School/ma	0.8
Map54	F80	F147	Lockdown	Stress and	0.4
Map54	F80	F120	Lockdown	No transpo	0.8
Map54	F80	F115	Lockdown	No preachi	0.8
Map54	F80	F106	Lockdown	No congreg	0.8
Map54	F83	F01	Malnutritior	Bad experi	0.4
Map54	F05	F01	Banks clos	Bad experi	0.6
Map54	F05	F63	Banks clos	Increased	0.4
Map54	F05	F147	Banks clos	Stress and	0.6
Map54	F07	F40	Businesses	Household	0.6
Map54	F07	F88	Businesses	Men unable	0.6
Map54	F07	F47	Businesses	Increase in	0.6
Map54	F07	F01	Businesses	Bad experi	0.8
Map54	F07	F64	Businesses	Increased	0.8
Map54	F07	F52	Businesses	Increase in	0.2
Map54	F07	F70	Businesses	Jobs lost	0.8
Map54	F20	F01	Difficult bre	Bad experi	0.4
Map54	F20	F63	Difficult bre	Increased	0.4
Map54	F29	F05	Fear of cor	Banks clos	0.6
Map54	F29	F20	Fear of cor	Difficult bre	0.6
Map54	F29	F01	Fear of cor	Bad experi	1
Map54	F29	F63	Fear of cor	Increased	0.4
Map54	F29	F62	Fear of cor	Increased	0.4
Map54	F29	F52	Fear of cor	Increase in	0.2
Map54	F29	F30	Fear of cor	Fear of dea	1
Map54	F29	F31	Fear of cor	Fear of eac	0.8
Map54	F29	F32	Fear of cor	Feeling soc	0.6
Map54	F29	F10	Fear of cor	Compulsor	0.8
Map54	F29	F147	Fear of cor	Stress and	0.6
Map54	F29	F145	Fear of cor	Stigma aga	1
Map54	F29	F144	Fear of cor	Social dista	0.8
Map54	F64	F40	Increased	Household	0.6
Map54	F64	F47	Increased	Increase in	0.6
Map54	F64	F01	Increased	Bad experi	1
Map54	F64	F52	Increased	Increase in	0.2
Map54	F63	F01	Increased	Bad experi	0.4
Map54	F62	F01	Increased	Bad experi	0.6
Map54	F62	F52	Increased	Increase in	0.2
Map54	F60	F01	Increased i	Bad experi	0.4
Map54	F60	F99	Increased i	More unwa	0.4
Map54	F158	F01	Youth/child	Bad experi	0.6
Map54	F158	F60	Youth/child	Increased i	0.6
Map54	F158	F16	Youth/child	Delay in ec	0.7
Map54	F158	F99	Youth/child	More unwa	0.4
Map54	F158	F133	Youth/child	Poor perfor	0.4
Map54	F16	F01	Delay in ec	Bad experi	0.6
Map54	F16	F133	Delay in ec	Poor perfor	0.4
Map54	F96	F40	More hung	Household	0.8
Map54	F96	F47	More hung	Increase in	0.6

For Peer Review

Map54	F96	F83	More hung	Malnutritior	0.6
Map54	F96	F01	More hung	Bad experi	1
Map54	F118	F01	No social g	Bad experi	0.4
Map54	F118	F63	No social g	Increased (	0.4
Map54	F118	F147	No social g	Stress and	0.4
Map54	F118	F115	No social g	No preachi	0.8
Map54	F118	F106	No social g	No congreg	0.8
Map54	F99	F01	More unwa	Bad experi	0.4
Map54	F110	F01	No funerals	Bad experi	0.2
Map54	F110	F63	No funerals	Increased (	0.2
Map54	F110	F147	No funerals	Stress and	0.2
Map54	F135	F40	Price hike	Household	0.6
Map54	F135	F47	Price hike	Increase in	0.6
Map54	F135	F83	Price hike	Malnutritior	0.6
Map54	F135	F01	Price hike	Bad experi	0.6
Map54	F135	F96	Price hike	More hung	0.6
Map54	F135	F78	Price hike	Less food t	0.6
Map54	F52	F01	Increase in	Bad experi	0.2
Map54	F30	F01	Fear of dea	Bad experi	1
Map54	F30	F145	Fear of dea	Stigma aga	0.8
Map54	F31	F01	Fear of eac	Bad experi	0.8
Map54	F32	F01	Feeling soc	Bad experi	0.4
Map54	F32	F62	Feeling soc	Increased r	0.4
Map54	F32	F52	Feeling soc	Increase in	0.2
Map54	F78	F40	Less food t	Household	0.8
Map54	F78	F47	Less food t	Increase in	0.6
Map54	F78	F83	Less food t	Malnutritior	0.6
Map54	F78	F01	Less food t	Bad experi	0.8
Map54	F78	F96	Less food t	More hung	0.8
Map54	F133	F01	Poor perfor	Bad experi	0.4
Map54	F10	F20	Compulsor	Difficult bre	0.6
Map54	F10	F01	Compulsor	Bad experi	0.4
Map54	F10	F63	Compulsor	Increased (	0.4
Map54	F70	F40	Jobs lost	Household	0.6
Map54	F70	F88	Jobs lost	Men unable	0.6
Map54	F70	F47	Jobs lost	Increase in	0.6
Map54	F70	F01	Jobs lost	Bad experi	0.8
Map54	F70	F64	Jobs lost	Increased p	0.8
Map54	F70	F52	Jobs lost	Increase in	0.2
Map54	F143	F40	Shortage o	Household	0.6
Map54	F143	F47	Shortage o	Increase in	0.6
Map54	F143	F83	Shortage o	Malnutritior	0.6
Map54	F143	F01	Shortage o	Bad experi	0.6
Map54	F143	F96	Shortage o	More hung	0.6
Map54	F143	F135	Shortage o	Price hike	0.6
Map54	F143	F78	Shortage o	Less food t	0.6
Map54	F142	F01	School/ma	Bad experi	0.6
Map54	F142	F60	School/ma	Increased i	0.6
Map54	F142	F158	School/ma	Youth/child	0.8
Map54	F142	F16	School/ma	Delay in ec	0.7
Map54	F142	F99	School/ma	More unwa	0.4
Map54	F142	F133	School/ma	Poor perfor	0.4
Map54	F147	F01	Stress and	Bad experi	0.6
Map54	F147	F63	Stress and	Increased (	0.4
Map54	F120	F40	No transpo	Household	0.6
Map54	F120	F47	No transpo	Increase in	0.6

For Peer Review

Map54	F120	F83	No transpo	Malnutritior	0.6
Map54	F120	F01	No transpo	Bad experi	0.6
Map54	F120	F96	No transpo	More hung	0.6
Map54	F120	F135	No transpo	Price hike	0.6
Map54	F120	F78	No transpo	Less food t	0.6
Map54	F120	F143	No transpo	Shortage o	0.6
Map54	F145	F01	Stigma aga	Bad experi	0.8
Map54	F144	F05	Social dista	Banks clos	0.6
Map54	F144	F01	Social dista	Bad experi	0.6
Map54	F144	F63	Social dista	Increased (	0.4
Map54	F144	F62	Social dista	Increased r	0.4
Map54	F144	F52	Social dista	Increase in	0.2
Map54	F144	F32	Social dista	Feeling soc	0.6
Map54	F144	F147	Social dista	Stress and	0.6
Map54	F115	F01	No preachi	Bad experi	0.4
Map54	F115	F63	No preachi	Increased (	0.4
Map54	F115	F147	No preachi	Stress and	0.4
Map54	F106	F01	No congreg	Bad experi	0.4
Map54	F106	F63	No congreg	Increased (	0.4
Map54	F106	F147	No congreg	Stress and	0.4
Map54	F106	F115	No congreg	No preachi	0.8
Map55	F20	F01	Difficult bre	Bad experi	0.2
Map55	F69	F130	Increased t	Physical in	0.4
Map55	F69	F01	Increased t	Bad experi	0.4
Map55	F29	F20	Fear of cor	Difficult bre	0.4
Map55	F29	F63	Fear of cor	Increased (	0.6
Map55	F29	F31	Fear of cor	Fear of eac	0.8
Map55	F29	F98	Fear of cor	More mate	0.6
Map55	F29	F124	Fear of cor	Not visiting	0.8
Map55	F29	F01	Fear of cor	Bad experi	0.6
Map55	F29	F10	Fear of cor	Compulsor	0.6
Map55	F64	F96	Increased p	More hung	0.6
Map55	F64	F83	Increased p	Malnutritior	0.4
Map55	F64	F01	Increased p	Bad experi	0.6
Map55	F63	F01	Increased (	Bad experi	0.6
Map55	F62	F01	Increased r	Bad experi	0.4
Map55	F60	F69	Increased i	Increased t	0.4
Map55	F60	F130	Increased i	Physical in	0.4
Map55	F60	F99	Increased i	More unwa	0.4
Map55	F60	F39	Increased i	HIV/STI inc	0.4
Map55	F60	F01	Increased i	Bad experi	0.6
Map55	F60	F65	Increased i	Increased r	0.6
Map55	F60	F57	Increased i	Increased (	0.8
Map55	F60	F58	Increased i	Increased (	0.6
Map55	F96	F83	More hung	Malnutritior	0.4
Map55	F96	F01	More hung	Bad experi	0.8
Map55	F95	F40	More hous	Household	0.4
Map55	F95	F47	More hous	Increase in	0.4
Map55	F95	F01	More hous	Bad experi	0.4
Map55	F130	F01	Physical in	Bad experi	0.4
Map55	F99	F01	More unwa	Bad experi	0.4
Map55	F135	F96	Price hike	More hung	0.4
Map55	F135	F83	Price hike	Malnutritior	0.4
Map55	F135	F01	Price hike	Bad experi	0.4
Map55	F31	F20	Fear of eac	Difficult bre	0.4
Map55	F31	F01	Fear of eac	Bad experi	0.4

For Peer Review

Map55	F31	F10	Fear of eac	Compulsor	0.6
Map55	F32	F62	Feeling soc	Increased r	0.4
Map55	F32	F01	Feeling soc	Bad experi	0.4
Map55	F32	F147	Feeling soc	Stress and	0.6
Map55	F78	F96	Less food t	More hung	0.8
Map55	F78	F83	Less food t	Malnutrition	0.4
Map55	F78	F01	Less food t	Bad experi	0.8
Map55	F39	F01	HIV/STI inc	Bad experi	0.4
Map55	F70	F96	Jobs lost	More hung	0.6
Map55	F70	F95	Jobs lost	More hous	0.4
Map55	F70	F135	Jobs lost	Price hike	0.6
Map55	F70	F78	Jobs lost	Less food t	0.6
Map55	F70	F40	Jobs lost	Household	0.6
Map55	F70	F88	Jobs lost	Men unable	0.6
Map55	F70	F47	Jobs lost	Increase in	0.4
Map55	F70	F87	Jobs lost	Men stayin	0.8
Map55	F70	F83	Jobs lost	Malnutrition	0.4
Map55	F70	F01	Jobs lost	Bad experi	0.6
Map55	F70	F143	Jobs lost	Shortage o	0.8
Map55	F98	F01	More mate	Bad experi	0.2
Map55	F124	F63	Not visiting	Increased c	0.6
Map55	F124	F98	Not visiting	More mate	0.6
Map55	F124	F01	Not visiting	Bad experi	0.6
Map55	F120	F64	No transpo	Increased p	0.4
Map55	F120	F96	No transpo	More hung	0.6
Map55	F120	F135	No transpo	Price hike	0.6
Map55	F120	F78	No transpo	Less food t	0.6
Map55	F120	F83	No transpo	Malnutrition	0.4
Map55	F120	F82	No transpo	Lost invest	0.6
Map55	F120	F07	No transpo	Businesses	0.6
Map55	F120	F01	No transpo	Bad experi	0.6
Map55	F120	F143	No transpo	Shortage o	0.8
Map55	F40	F47	Household	Increase in	0.4
Map55	F40	F01	Household	Bad experi	0.4
Map55	F88	F95	Men unable	More hous	0.4
Map55	F88	F40	Men unable	Household	0.6
Map55	F88	F47	Men unable	Increase in	0.4
Map55	F88	F01	Men unable	Bad experi	0.4
Map55	F47	F01	Increase in	Bad experi	0.4
Map55	F85	F64	Markets/sh	Increased p	0.4
Map55	F85	F96	Markets/sh	More hung	0.6
Map55	F85	F135	Markets/sh	Price hike	0.6
Map55	F85	F78	Markets/sh	Less food t	0.6
Map55	F85	F83	Markets/sh	Malnutrition	0.4
Map55	F85	F82	Markets/sh	Lost invest	0.6
Map55	F85	F07	Markets/sh	Businesses	0.8
Map55	F85	F01	Markets/sh	Bad experi	0.6
Map55	F85	F143	Markets/sh	Shortage o	0.6
Map55	F87	F95	Men stayin	More hous	0.4
Map55	F87	F40	Men stayin	Household	0.6
Map55	F87	F88	Men stayin	Men unable	0.6
Map55	F87	F47	Men stayin	Increase in	0.4
Map55	F87	F01	Men stayin	Bad experi	0.4
Map55	F81	F01	Loss of Ha	Bad experi	0.2
Map55	F80	F69	Lockdown	Increased t	0.4
Map55	F80	F64	Lockdown	Increased p	0.4

For Peer Review

Map55	F80	F60	Lockdown	Increased i	0.6
Map55	F80	F96	Lockdown	More hung	0.6
Map55	F80	F95	Lockdown	More hous	0.4
Map55	F80	F130	Lockdown	Physical in	0.4
Map55	F80	F99	Lockdown	More unwa	0.4
Map55	F80	F135	Lockdown	Price hike	0.6
Map55	F80	F78	Lockdown	Less food t	0.6
Map55	F80	F39	Lockdown	HIV/STI inc	0.4
Map55	F80	F70	Lockdown	Jobs lost	0.4
Map55	F80	F120	Lockdown	No transpo	0.8
Map55	F80	F40	Lockdown	Household	0.6
Map55	F80	F88	Lockdown	Men unable	0.6
Map55	F80	F47	Lockdown	Increase in	0.4
Map55	F80	F85	Lockdown	Markets/sh	0.8
Map55	F80	F87	Lockdown	Men stayin	0.8
Map55	F80	F81	Lockdown	Loss of Ha	0.6
Map55	F80	F83	Lockdown	Malnutritior	0.4
Map55	F80	F82	Lockdown	Lost invest	0.6
Map55	F80	F07	Lockdown	Businesses	0.8
Map55	F80	F06	Lockdown	Borders clc	1
Map55	F80	F01	Lockdown	Bad experi	0.6
Map55	F80	F65	Lockdown	Increased r	0.6
Map55	F80	F158	Lockdown	Youth/child	1
Map55	F80	F114	Lockdown	No pilgrima	1
Map55	F80	F115	Lockdown	No preachi	0.6
Map55	F80	F57	Lockdown	Increased c	0.6
Map55	F80	F58	Lockdown	Increased c	0.6
Map55	F80	F13	Lockdown	Could not c	0.6
Map55	F80	F16	Lockdown	Delay in ec	0.6
Map55	F80	F143	Lockdown	Shortage o	0.8
Map55	F80	F142	Lockdown	School/mac	1
Map55	F80	F140	Lockdown	Restriction	0.8
Map55	F80	F106	Lockdown	No congreg	0.8
Map55	F80	F100	Lockdown	Mosques/c	0.8
Map55	F83	F01	Malnutritior	Bad experi	0.4
Map55	F82	F64	Lost invest	Increased p	0.4
Map55	F82	F96	Lost invest	More hung	0.4
Map55	F82	F83	Lost invest	Malnutritior	0.4
Map55	F82	F01	Lost invest	Bad experi	0.4
Map55	F07	F64	Businesses	Increased p	0.4
Map55	F07	F96	Businesses	More hung	0.6
Map55	F07	F135	Businesses	Price hike	0.6
Map55	F07	F78	Businesses	Less food t	0.6
Map55	F07	F83	Businesses	Malnutritior	0.4
Map55	F07	F82	Businesses	Lost invest	0.6
Map55	F07	F01	Businesses	Bad experi	0.6
Map55	F07	F143	Businesses	Shortage o	0.6
Map55	F06	F81	Borders clc	Loss of Ha	0.6
Map55	F06	F01	Borders clc	Bad experi	0.2
Map55	F06	F114	Borders clc	No pilgrima	1
Map55	F65	F99	Increased r	More unwa	0.4
Map55	F65	F39	Increased r	HIV/STI inc	0.4
Map55	F65	F01	Increased r	Bad experi	0.4
Map55	F158	F69	Youth/child	Increased t	0.4
Map55	F158	F60	Youth/child	Increased i	0.6
Map55	F158	F130	Youth/child	Physical in	0.4

For Peer Review

Map55	F158	F99	Youth/child	More unwa	0.4
Map55	F158	F39	Youth/child	HIV/STI inc	0.4
Map55	F158	F01	Youth/child	Bad experi	0.6
Map55	F158	F65	Youth/child	Increased r	0.6
Map55	F158	F57	Youth/child	Increased (	0.6
Map55	F158	F58	Youth/child	Increased (	0.6
Map55	F158	F13	Youth/child	Could not c	0.6
Map55	F158	F16	Youth/child	Delay in ec	0.6
Map55	F118	F62	No social g	Increased r	0.4
Map55	F118	F32	No social g	Feeling soc	0.6
Map55	F118	F01	No social g	Bad experi	0.4
Map55	F118	F147	No social g	Stress and	0.6
Map55	F114	F81	No pilgrima	Loss of Ha	0.6
Map55	F114	F01	No pilgrima	Bad experi	0.2
Map55	F115	F69	No preachi	Increased t	0.4
Map55	F115	F60	No preachi	Increased i	0.4
Map55	F115	F130	No preachi	Physical in	0.4
Map55	F115	F99	No preachi	More unwa	0.4
Map55	F115	F39	No preachi	HIV/STI inc	0.4
Map55	F115	F01	No preachi	Bad experi	0.4
Map55	F115	F65	No preachi	Increased r	0.4
Map55	F115	F57	No preachi	Increased (	0.4
Map55	F115	F58	No preachi	Increased (	0.4
Map55	F57	F01	Increased (	Bad experi	0.6
Map55	F58	F69	Increased (	Increased t	0.4
Map55	F58	F130	Increased (	Physical in	0.4
Map55	F58	F01	Increased (	Bad experi	0.4
Map55	F13	F69	Could not c	Increased t	0.4
Map55	F13	F60	Could not c	Increased i	0.8
Map55	F13	F130	Could not c	Physical in	0.4
Map55	F13	F99	Could not c	More unwa	0.4
Map55	F13	F39	Could not c	HIV/STI inc	0.4
Map55	F13	F01	Could not c	Bad experi	0.6
Map55	F13	F65	Could not c	Increased r	0.6
Map55	F13	F57	Could not c	Increased (	0.8
Map55	F13	F58	Could not c	Increased (	0.6
Map55	F10	F20	Compulsor	Difficult bre	0.4
Map55	F10	F01	Compulsor	Bad experi	0.2
Map55	F16	F01	Delay in ec	Bad experi	0.6
Map55	F143	F96	Shortage o	More hung	0.6
Map55	F143	F135	Shortage o	Price hike	0.6
Map55	F143	F78	Shortage o	Less food t	0.6
Map55	F143	F83	Shortage o	Malnutritior	0.4
Map55	F143	F01	Shortage o	Bad experi	0.6
Map55	F142	F69	School/ma	Increased t	0.4
Map55	F142	F60	School/ma	Increased i	0.6
Map55	F142	F130	School/ma	Physical in	0.4
Map55	F142	F99	School/ma	More unwa	0.4
Map55	F142	F39	School/ma	HIV/STI inc	0.4
Map55	F142	F01	School/ma	Bad experi	0.6
Map55	F142	F65	School/ma	Increased r	0.6
Map55	F142	F158	School/ma	Youth/child	1
Map55	F142	F57	School/ma	Increased (	0.6
Map55	F142	F58	School/ma	Increased (	0.6
Map55	F142	F13	School/ma	Could not c	0.6
Map55	F142	F16	School/ma	Delay in ec	0.6

For Peer Review

Map55	F140	F64	Restriction	Increased p	0.4
Map55	F140	F96	Restriction	More hung	0.6
Map55	F140	F95	Restriction	More house	0.4
Map55	F140	F135	Restriction	Price hike	0.6
Map55	F140	F78	Restriction	Less food t	0.6
Map55	F140	F70	Restriction	Jobs lost	0.4
Map55	F140	F120	Restriction	No transpo	0.8
Map55	F140	F40	Restriction	Household	0.4
Map55	F140	F88	Restriction	Men unable	0.4
Map55	F140	F47	Restriction	Increase in	0.4
Map55	F140	F87	Restriction	Men stayin	0.4
Map55	F140	F83	Restriction	Malnutrition	0.4
Map55	F140	F82	Restriction	Lost invest	0.6
Map55	F140	F07	Restriction	Businesses	0.6
Map55	F140	F01	Restriction	Bad experi	0.6
Map55	F140	F143	Restriction	Shortage o	0.8
Map55	F147	F62	Stress and	Increased r	0.4
Map55	F147	F01	Stress and	Bad experi	0.4
Map55	F144	F69	Social dista	Increased t	0.4
Map55	F144	F62	Social dista	Increased r	0.4
Map55	F144	F60	Social dista	Increased i	0.4
Map55	F144	F130	Social dista	Physical in	0.4
Map55	F144	F99	Social dista	More unwa	0.4
Map55	F144	F32	Social dista	Feeling soc	0.6
Map55	F144	F39	Social dista	HIV/STI inc	0.4
Map55	F144	F01	Social dista	Bad experi	0.4
Map55	F144	F65	Social dista	Increased r	0.4
Map55	F144	F118	Social dista	No social g	0.8
Map55	F144	F115	Social dista	No preachi	0.6
Map55	F144	F57	Social dista	Increased c	0.4
Map55	F144	F58	Social dista	Increased c	0.4
Map55	F144	F147	Social dista	Stress and	0.6
Map55	F144	F106	Social dista	No congreg	0.8
Map55	F144	F100	Social dista	Mosques/c	0.8
Map55	F106	F69	No congreg	Increased t	0.4
Map55	F106	F60	No congreg	Increased i	0.4
Map55	F106	F130	No congreg	Physical in	0.4
Map55	F106	F99	No congreg	More unwa	0.4
Map55	F106	F39	No congreg	HIV/STI inc	0.4
Map55	F106	F01	No congreg	Bad experi	0.4
Map55	F106	F65	No congreg	Increased r	0.4
Map55	F106	F115	No congreg	No preachi	0.6
Map55	F106	F57	No congreg	Increased c	0.4
Map55	F106	F58	No congreg	Increased c	0.4
Map55	F100	F69	Mosques/c	Increased t	0.4
Map55	F100	F60	Mosques/c	Increased i	0.4
Map55	F100	F130	Mosques/c	Physical in	0.4
Map55	F100	F99	Mosques/c	More unwa	0.4
Map55	F100	F39	Mosques/c	HIV/STI inc	0.4
Map55	F100	F01	Mosques/c	Bad experi	0.4
Map55	F100	F65	Mosques/c	Increased r	0.4
Map55	F100	F115	Mosques/c	No preachi	0.6
Map55	F100	F57	Mosques/c	Increased c	0.4
Map55	F100	F58	Mosques/c	Increased c	0.4
Map55	F100	F106	Mosques/c	No congreg	0.8
Map56	F20	F01	Difficult bre	Bad experi	0.4

For Peer Review

Map56	F24	F47	Domestic v	Increase in	0.6
Map56	F24	F01	Domestic v	Bad experi	0.4
Map56	F24	F65	Domestic v	Increased r	0.4
Map56	F67	F01	Increased s	Bad experi	1
Map56	F29	F63	Fear of cor	Increased c	0.8
Map56	F29	F31	Fear of cor	Fear of eac	1
Map56	F29	F38	Fear of cor	Health wor	0.8
Map56	F29	F76	Fear of cor	Less affect	1
Map56	F29	F124	Fear of cor	Not visiting	1
Map56	F29	F01	Fear of cor	Bad experi	0.8
Map56	F29	F14	Fear of cor	Could not g	0.8
Map56	F28	F63	Fear of clin	Increased c	0.8
Map56	F28	F124	Fear of clin	Not visiting	1
Map56	F28	F01	Fear of clin	Bad experi	0.8
Map56	F28	F14	Fear of clin	Could not g	0.8
Map56	F63	F01	Increased c	Bad experi	0.8
Map56	F62	F01	Increased r	Bad experi	1
Map56	F62	F147	Increased r	Stress and	0.4
Map56	F60	F67	Increased i	Increased s	0.4
Map56	F60	F128	Increased i	People fee	0.2
Map56	F60	F46	Increased i	Increase in	0.2
Map56	F60	F01	Increased i	Bad experi	0.8
Map56	F60	F147	Increased i	Stress and	0.8
Map56	F96	F01	More hung	Bad experi	1
Map56	F135	F96	Price hike	More hung	0.6
Map56	F135	F78	Price hike	Less food t	0.6
Map56	F135	F01	Price hike	Bad experi	0.6
Map56	F31	F76	Fear of eac	Less affect	1
Map56	F31	F01	Fear of eac	Bad experi	0.8
Map56	F32	F01	Feeling soc	Bad experi	0.6
Map56	F78	F96	Less food t	More hung	0.8
Map56	F78	F01	Less food t	Bad experi	0.8
Map56	F35	F63	Forced CO	Increased c	0.8
Map56	F35	F124	Forced CO	Not visiting	1
Map56	F35	F01	Forced CO	Bad experi	0.8
Map56	F35	F14	Forced CO	Could not g	0.8
Map56	F38	F63	Health wor	Increased c	0.6
Map56	F38	F01	Health wor	Bad experi	0.6
Map56	F38	F14	Health wor	Could not g	0.6
Map56	F76	F01	Less affect	Bad experi	0.8
Map56	F70	F24	Jobs lost	Domestic v	0.8
Map56	F70	F40	Jobs lost	Household	0.8
Map56	F70	F88	Jobs lost	Men unable	0.8
Map56	F70	F47	Jobs lost	Increase in	0.6
Map56	F70	F87	Jobs lost	Men stayin	1
Map56	F70	F01	Jobs lost	Bad experi	0.4
Map56	F70	F65	Jobs lost	Increased r	0.4
Map56	F124	F63	Not visiting	Increased c	0.8
Map56	F124	F01	Not visiting	Bad experi	0.8
Map56	F124	F14	Not visiting	Could not g	0.8
Map56	F120	F96	No transpo	More hung	0.6
Map56	F120	F135	No transpo	Price hike	0.6
Map56	F120	F78	No transpo	Less food t	0.6
Map56	F120	F01	No transpo	Bad experi	0.6
Map56	F120	F143	No transpo	Shortage o	0.8
Map56	F128	F01	People feel	Bad experi	0.4

For Peer Review

Map56	F40	F24	Household	Domestic v	0.8
Map56	F40	F47	Household	Increase in	0.6
Map56	F40	F01	Household	Bad experi	0.4
Map56	F40	F65	Household	Increased r	0.4
Map56	F88	F24	Men unabl	Domestic v	0.8
Map56	F88	F40	Men unabl	Household	0.8
Map56	F88	F47	Men unabl	Increase in	0.6
Map56	F88	F01	Men unabl	Bad experi	0.4
Map56	F88	F65	Men unabl	Increased r	0.4
Map56	F47	F01	Increase in	Bad experi	0.4
Map56	F47	F65	Increase in	Increased r	0.4
Map56	F46	F128	Increase in	People fee	0.2
Map56	F46	F01	Increase in	Bad experi	0.2
Map56	F85	F96	Markets/sh	More hung	0.6
Map56	F85	F135	Markets/sh	Price hike	0.6
Map56	F85	F78	Markets/sh	Less food t	0.6
Map56	F85	F82	Markets/sh	Lost invest	0.8
Map56	F85	F07	Markets/sh	Businesses	0.8
Map56	F85	F01	Markets/sh	Bad experi	0.8
Map56	F85	F64	Markets/sh	Increased p	0.8
Map56	F85	F143	Markets/sh	Shortage o	0.6
Map56	F87	F24	Men stayin	Domestic v	0.8
Map56	F87	F40	Men stayin	Household	0.8
Map56	F87	F88	Men stayin	Men unabl	0.8
Map56	F87	F47	Men stayin	Increase in	0.6
Map56	F87	F01	Men stayin	Bad experi	0.4
Map56	F87	F65	Men stayin	Increased r	0.4
Map56	F81	F62	Loss of Ha	Increased r	0.8
Map56	F81	F01	Loss of Ha	Bad experi	0.8
Map56	F81	F147	Loss of Ha	Stress and	0.4
Map56	F80	F24	Lockdown	Domestic v	0.8
Map56	F80	F67	Lockdown	Increased s	0.4
Map56	F80	F62	Lockdown	Increased r	0.4
Map56	F80	F60	Lockdown	Increased i	0.6
Map56	F80	F96	Lockdown	More hung	0.6
Map56	F80	F135	Lockdown	Price hike	0.6
Map56	F80	F78	Lockdown	Less food t	0.6
Map56	F80	F70	Lockdown	Jobs lost	0.6
Map56	F80	F120	Lockdown	No transpo	1
Map56	F80	F128	Lockdown	People fee	0.2
Map56	F80	F40	Lockdown	Household	0.8
Map56	F80	F88	Lockdown	Men unabl	0.8
Map56	F80	F47	Lockdown	Increase in	0.6
Map56	F80	F46	Lockdown	Increase in	0.2
Map56	F80	F85	Lockdown	Markets/sh	1
Map56	F80	F87	Lockdown	Men stayin	1
Map56	F80	F81	Lockdown	Loss of Ha	0.4
Map56	F80	F82	Lockdown	Lost invest	0.8
Map56	F80	F05	Lockdown	Banks clos	0.6
Map56	F80	F07	Lockdown	Businesses	0.8
Map56	F80	F06	Lockdown	Borders clo	0.4
Map56	F80	F01	Lockdown	Bad experi	0.8
Map56	F80	F65	Lockdown	Increased r	0.4
Map56	F80	F64	Lockdown	Increased p	0.8
Map56	F80	F158	Lockdown	Youth/child	0.7
Map56	F80	F114	Lockdown	No pilgrima	0.4

For Peer Review

Map56	F80	F16	Lockdown	Delay in ec	0.7
Map56	F80	F143	Lockdown	Shortage o	0.8
Map56	F80	F142	Lockdown	School/mai	0.7
Map56	F80	F140	Lockdown	Restriction	1
Map56	F80	F147	Lockdown	Stress and	0.7
Map56	F80	F106	Lockdown	No congreg	0.6
Map56	F80	F100	Lockdown	Mosques/c	0.6
Map56	F82	F01	Lost invest	Bad experi	0.8
Map56	F82	F64	Lost invest	Increased p	0.8
Map56	F05	F01	Banks clos	Bad experi	0.6
Map56	F05	F64	Banks clos	Increased p	0.6
Map56	F05	F147	Banks clos	Stress and	0.6
Map56	F07	F82	Businesses	Lost invest	0.8
Map56	F07	F01	Businesses	Bad experi	0.8
Map56	F07	F64	Businesses	Increased p	0.8
Map56	F06	F62	Borders clo	Increased r	0.6
Map56	F06	F81	Borders clo	Loss of Ha	0.6
Map56	F06	F01	Borders clo	Bad experi	0.6
Map56	F06	F114	Borders clo	No pilgrima	0.6
Map56	F06	F147	Borders clo	Stress and	0.4
Map56	F65	F01	Increased r	Bad experi	0.2
Map56	F64	F01	Increased p	Bad experi	0.8
Map56	F154	F20	Wearing fa	Difficult bre	0.6
Map56	F154	F01	Wearing fa	Bad experi	0.4
Map56	F158	F67	Youth/child	Increased s	0.4
Map56	F158	F60	Youth/child	Increased i	0.6
Map56	F158	F128	Youth/child	People fee	0.2
Map56	F158	F46	Youth/child	Increase in	0.2
Map56	F158	F01	Youth/child	Bad experi	0.6
Map56	F158	F147	Youth/child	Stress and	0.6
Map56	F118	F32	No social g	Feeling soc	0.6
Map56	F118	F01	No social g	Bad experi	0.6
Map56	F114	F62	No pilgrima	Increased r	0.8
Map56	F114	F81	No pilgrima	Loss of Ha	0.8
Map56	F114	F01	No pilgrima	Bad experi	0.8
Map56	F114	F147	No pilgrima	Stress and	0.4
Map56	F110	F01	No funerals	Bad experi	0.6
Map56	F110	F147	No funerals	Stress and	0.6
Map56	F10	F20	Compulsor	Difficult bre	0.6
Map56	F10	F01	Compulsor	Bad experi	0.4
Map56	F10	F154	Compulsor	Wearing fa	0.8
Map56	F16	F01	Delay in ec	Bad experi	0.8
Map56	F16	F147	Delay in ec	Stress and	0.8
Map56	F14	F63	Could not g	Increased c	0.8
Map56	F14	F01	Could not g	Bad experi	0.8
Map56	F143	F96	Shortage o	More hung	0.6
Map56	F143	F135	Shortage o	Price hike	0.6
Map56	F143	F78	Shortage o	Less food t	0.6
Map56	F143	F01	Shortage o	Bad experi	0.6
Map56	F142	F67	School/mai	Increased s	0.4
Map56	F142	F62	School/mai	Increased r	0.4
Map56	F142	F60	School/mai	Increased i	0.6
Map56	F142	F128	School/mai	People fee	0.2
Map56	F142	F46	School/mai	Increase in	0.2
Map56	F142	F81	School/mai	Loss of Ha	0.4
Map56	F142	F06	School/mai	Borders clo	0.4

For Peer Review

Map56	F142	F01	School/ma	Bad experi	0.8
Map56	F142	F158	School/ma	Youth/child	1
Map56	F142	F114	School/ma	No pilgrima	0.4
Map56	F142	F16	School/ma	Delay in ec	1
Map56	F142	F147	School/ma	Stress and	0.8
Map56	F140	F24	Restriction	Domestic v	0.8
Map56	F140	F96	Restriction	More hung	0.6
Map56	F140	F135	Restriction	Price hike	0.6
Map56	F140	F78	Restriction	Less food t	0.6
Map56	F140	F70	Restriction	Jobs lost	0.6
Map56	F140	F120	Restriction	No transpo	1
Map56	F140	F40	Restriction	Household	0.8
Map56	F140	F88	Restriction	Men unabl	0.8
Map56	F140	F47	Restriction	Increase in	0.6
Map56	F140	F87	Restriction	Men stayin	1
Map56	F140	F01	Restriction	Bad experi	0.6
Map56	F140	F65	Restriction	Increased r	0.4
Map56	F140	F143	Restriction	Shortage o	0.8
Map56	F147	F01	Stress and	Bad experi	1
Map56	F144	F32	Social dista	Feeling soc	0.4
Map56	F144	F05	Social dista	Banks clos	0.6
Map56	F144	F01	Social dista	Bad experi	0.6
Map56	F144	F64	Social dista	Increased p	0.6
Map56	F144	F118	Social dista	No social g	0.4
Map56	F144	F110	Social dista	No funerals	0.8
Map56	F144	F147	Social dista	Stress and	0.6
Map56	F106	F01	No congreg	Bad experi	0.8
Map56	F106	F147	No congreg	Stress and	0.8
Map56	F100	F01	Mosques/c	Bad experi	0.6
Map56	F100	F147	Mosques/c	Stress and	0.6
Map56	F100	F106	Mosques/c	No congreg	0.6
Map57	F88	F01	Men unabl	Bad experi	0.8
Map57	F88	F63	Men unabl	Increased c	0.4
Map57	F88	F78	Men unabl	Less food t	0.8
Map57	F85	F01	Markets/sh	Bad experi	0.8
Map57	F85	F63	Markets/sh	Increased c	0.4
Map57	F85	F112	Markets/sh	No home/p	0.8
Map57	F85	F135	Markets/sh	Price hike	0.8
Map57	F85	F78	Markets/sh	Less food t	0.8
Map57	F80	F88	Lockdown	Men unabl	0.8
Map57	F80	F85	Lockdown	Markets/sh	0.8
Map57	F80	F01	Lockdown	Bad experi	0.8
Map57	F80	F63	Lockdown	Increased c	0.4
Map57	F80	F158	Lockdown	Youth/child	0.6
Map57	F80	F114	Lockdown	No pilgrima	0.6
Map57	F80	F133	Lockdown	Poor perfor	0.6
Map57	F80	F112	Lockdown	No home/p	0.8
Map57	F80	F135	Lockdown	Price hike	0.8
Map57	F80	F78	Lockdown	Less food t	0.8
Map57	F80	F13	Lockdown	Could not c	0.6
Map57	F80	F16	Lockdown	Delay in ec	0.8
Map57	F80	F142	Lockdown	School/ma	0.8
Map57	F80	F140	Lockdown	Restriction	0.8
Map57	F80	F147	Lockdown	Stress and	0.6
Map57	F80	F06	Lockdown	Borders clo	0.8
Map57	F20	F01	Difficult bre	Bad experi	0.4

For Peer Review

Map57	F29	F01	Fear of cor	Bad experi	0.6
Map57	F29	F118	Fear of cor	No social g	0.2
Map57	F29	F30	Fear of cor	Fear of dea	0.8
Map57	F29	F147	Fear of cor	Stress and	0.6
Map57	F29	F144	Fear of cor	Social dista	0.2
Map57	F29	F106	Fear of cor	No congreg	0.2
Map57	F63	F01	Increased (	Bad experi	0.2
Map57	F154	F20	Wearing fa	Difficult bre	0.2
Map57	F154	F01	Wearing fa	Bad experi	0.2
Map57	F158	F01	Youth/child	Bad experi	0.6
Map57	F158	F13	Youth/child	Could not c	0.6
Map57	F158	F147	Youth/child	Stress and	0.6
Map57	F118	F01	No social g	Bad experi	0.6
Map57	F118	F147	No social g	Stress and	0.4
Map57	F118	F106	No social g	No congreg	0.6
Map57	F114	F01	No pilgrima	Bad experi	0.6
Map57	F114	F147	No pilgrima	Stress and	0.6
Map57	F133	F01	Poor perfor	Bad experi	0.4
Map57	F133	F147	Poor perfor	Stress and	0.4
Map57	F112	F01	No home/p	Bad experi	0.8
Map57	F112	F63	No home/p	Increased (	0.4
Map57	F112	F135	No home/p	Price hike	0.8
Map57	F112	F78	No home/p	Less food t	0.8
Map57	F135	F01	Price hike	Bad experi	0.8
Map57	F135	F63	Price hike	Increased (	0.4
Map57	F135	F78	Price hike	Less food t	1
Map57	F30	F01	Fear of dea	Bad experi	0.6
Map57	F78	F01	Less food t	Bad experi	0.8
Map57	F78	F63	Less food t	Increased (	0.4
Map57	F37	F01	Health facil	Bad experi	0.2
Map57	F37	F63	Health facil	Increased (	0.6
Map57	F37	F14	Health facil	Could not g	0.6
Map57	F13	F01	Could not c	Bad experi	0.6
Map57	F13	F147	Could not c	Stress and	0.6
Map57	F16	F01	Delay in ed	Bad experi	0.4
Map57	F16	F133	Delay in ed	Poor perfor	0.6
Map57	F16	F147	Delay in ed	Stress and	0.4
Map57	F14	F01	Could not g	Bad experi	0.2
Map57	F14	F63	Could not g	Increased (	0.8
Map57	F142	F01	School/ma	Bad experi	0.6
Map57	F142	F158	School/ma	Youth/child	0.6
Map57	F142	F133	School/ma	Poor perfor	0.6
Map57	F142	F13	School/ma	Could not c	0.6
Map57	F142	F16	School/ma	Delay in ec	0.8
Map57	F142	F147	School/ma	Stress and	0.6
Map57	F140	F88	Restriction	Men unable	1
Map57	F140	F01	Restriction	Bad experi	0.8
Map57	F140	F63	Restriction	Increased (	0.4
Map57	F140	F78	Restriction	Less food t	0.8
Map57	F147	F01	Stress and	Bad experi	0.8
Map57	F144	F01	Social dista	Bad experi	0.6
Map57	F144	F118	Social dista	No social g	0.6
Map57	F144	F147	Social dista	Stress and	0.4
Map57	F144	F106	Social dista	No congreg	0.6
Map57	F06	F01	Borders clc	Bad experi	0.6
Map57	F06	F114	Borders clc	No pilgrima	0.6

For Peer Review

Map57	F06	F147	Borders clc	Stress and	0.6
Map57	F106	F01	No congreg	Bad experi	0.6
Map58	F85	F83	Markets/sh	Malnutritior	0.6
Map58	F85	F07	Markets/sh	Businesses	0.8
Map58	F85	F01	Markets/sh	Bad experi	0.8
Map58	F85	F64	Markets/sh	Increased p	0.8
Map58	F85	F63	Markets/sh	Increased c	0.4
Map58	F85	F96	Markets/sh	More hung	0.8
Map58	F80	F85	Lockdown	Markets/sh	1
Map58	F80	F83	Lockdown	Malnutritior	0.6
Map58	F80	F07	Lockdown	Businesses	0.8
Map58	F80	F01	Lockdown	Bad experi	0.8
Map58	F80	F64	Lockdown	Increased p	0.8
Map58	F80	F63	Lockdown	Increased c	0.6
Map58	F80	F158	Lockdown	Youth/child	0.8
Map58	F80	F96	Lockdown	More hung	0.8
Map58	F80	F114	Lockdown	No pilgrima	0.8
Map58	F80	F133	Lockdown	Poor perfor	0.6
Map58	F80	F32	Lockdown	Feeling soc	0.4
Map58	F80	F118	Lockdown	No social g	0.6
Map58	F80	F37	Lockdown	Health facil	0.6
Map58	F80	F13	Lockdown	Could not c	0.6
Map58	F80	F14	Lockdown	Could not g	0.6
Map58	F80	F142	Lockdown	School/mac	1
Map58	F80	F140	Lockdown	Restriction	0.6
Map58	F80	F147	Lockdown	Stress and	0.6
Map58	F80	F06	Lockdown	Borders clc	1
Map58	F80	F106	Lockdown	No congreg	0.6
Map58	F80	F100	Lockdown	Mosques/c	1
Map58	F83	F01	Malnutritior	Bad experi	0.6
Map58	F83	F63	Malnutritior	Increased c	0.4
Map58	F07	F83	Businesses	Malnutritior	0.6
Map58	F07	F01	Businesses	Bad experi	0.8
Map58	F07	F64	Businesses	Increased p	0.8
Map58	F07	F63	Businesses	Increased c	0.4
Map58	F07	F96	Businesses	More hung	0.8
Map58	F20	F01	Difficult bre	Bad experi	0.2
Map58	F29	F01	Fear of cor	Bad experi	0.8
Map58	F29	F30	Fear of cor	Fear of dea	0.8
Map58	F29	F31	Fear of cor	Fear of eac	0.6
Map58	F64	F83	Increased p	Malnutritior	0.6
Map58	F64	F01	Increased p	Bad experi	0.8
Map58	F64	F63	Increased p	Increased c	0.4
Map58	F64	F96	Increased p	More hung	0.8
Map58	F63	F01	Increased c	Bad experi	0.4
Map58	F158	F01	Youth/child	Bad experi	0.4
Map58	F158	F133	Youth/child	Poor perfor	0.6
Map58	F158	F13	Youth/child	Could not c	0.6
Map58	F158	F147	Youth/child	Stress and	0.6
Map58	F96	F83	More hung	Malnutritior	0.6
Map58	F96	F01	More hung	Bad experi	1
Map58	F96	F63	More hung	Increased c	0.4
Map58	F114	F01	No pilgrima	Bad experi	0.4
Map58	F114	F147	No pilgrima	Stress and	0.4
Map58	F133	F01	Poor perfor	Bad experi	0.4
Map58	F133	F147	Poor perfor	Stress and	0.4

For Peer Review

Map58	F30	F01	Fear of dea	Bad experi	1
Map58	F30	F31	Fear of dea	Fear of eac	0.6
Map58	F31	F01	Fear of eac	Bad experi	0.4
Map58	F32	F01	Feeling soc	Bad experi	0.4
Map58	F118	F01	No social g	Bad experi	0.4
Map58	F118	F32	No social g	Feeling soc	0.4
Map58	F37	F01	Health facil	Bad experi	0.4
Map58	F37	F63	Health facil	Increased c	0.8
Map58	F37	F14	Health facil	Could not g	0.8
Map58	F13	F01	Could not c	Bad experi	0.4
Map58	F13	F147	Could not c	Stress and	0.6
Map58	F10	F20	Compulsor	Difficult bre	0.8
Map58	F10	F01	Compulsor	Bad experi	0.2
Map58	F14	F01	Could not g	Bad experi	0.4
Map58	F14	F63	Could not g	Increased c	0.8
Map58	F142	F01	School/ma	Bad experi	0.4
Map58	F142	F158	School/ma	Youth/child	0.8
Map58	F142	F133	School/ma	Poor perfor	0.6
Map58	F142	F13	School/ma	Could not c	0.6
Map58	F142	F147	School/ma	Stress and	0.6
Map58	F140	F01	Restriction	Bad experi	0.4
Map58	F140	F32	Restriction	Feeling soc	0.4
Map58	F140	F118	Restriction	No social g	0.8
Map58	F147	F01	Stress and	Bad experi	0.4
Map58	F06	F01	Borders clo	Bad experi	0.4
Map58	F06	F114	Borders clo	No pilgrima	0.8
Map58	F06	F147	Borders clo	Stress and	0.4
Map58	F106	F01	No congreg	Bad experi	0.2
Map58	F100	F01	Mosques/c	Bad experi	0.2
Map58	F100	F106	Mosques/c	No congreg	0.6
Map59	F40	F47	Household	Increase in	0.4
Map59	F40	F01	Household	Bad experi	0.4
Map59	F40	F137	Household	Reduced s	0.6
Map59	F47	F01	Increase in	Bad experi	0.4
Map59	F85	F82	Markets/sh	Lost invest	0.6
Map59	F85	F07	Markets/sh	Businesses	0.8
Map59	F85	F01	Markets/sh	Bad experi	0.6
Map59	F85	F64	Markets/sh	Increased p	0.6
Map59	F85	F63	Markets/sh	Increased c	0.6
Map59	F85	F96	Markets/sh	More hung	0.6
Map59	F87	F40	Men stayin	Household	0.6
Map59	F87	F47	Men stayin	Increase in	0.4
Map59	F87	F01	Men stayin	Bad experi	0.4
Map59	F87	F137	Men stayin	Reduced s	0.6
Map59	F80	F40	Lockdown	Household	0.6
Map59	F80	F47	Lockdown	Increase in	0.4
Map59	F80	F85	Lockdown	Markets/sh	1
Map59	F80	F87	Lockdown	Men stayin	1
Map59	F80	F82	Lockdown	Lost invest	0.6
Map59	F80	F07	Lockdown	Businesses	0.8
Map59	F80	F01	Lockdown	Bad experi	0.6
Map59	F80	F64	Lockdown	Increased p	0.6
Map59	F80	F63	Lockdown	Increased c	0.6
Map59	F80	F62	Lockdown	Increased r	0.4
Map59	F80	F157	Lockdown	Women un	0.8
Map59	F80	F158	Lockdown	Youth/child	0.8

For Peer Review

Map59	F80	F96	Lockdown	More hung	0.6
Map59	F80	F95	Lockdown	More hous	0.6
Map59	F80	F118	Lockdown	No social g	0.6
Map59	F80	F98	Lockdown	More mate	0.8
Map59	F80	F137	Lockdown	Reduced s	0.6
Map59	F80	F32	Lockdown	Feeling soc	0.4
Map59	F80	F133	Lockdown	Poor perfor	0.6
Map59	F80	F38	Lockdown	Health wor	0.8
Map59	F80	F16	Lockdown	Delay in ed	0.8
Map59	F80	F142	Lockdown	School/mad	1
Map59	F80	F140	Lockdown	Restriction	0.8
Map59	F80	F147	Lockdown	Stress and	0.6
Map59	F80	F120	Lockdown	No transpo	0.8
Map59	F80	F115	Lockdown	No preachi	0.6
Map59	F80	F106	Lockdown	No congreg	0.8
Map59	F80	F100	Lockdown	Mosques/c	1
Map59	F82	F01	Lost invest	Bad experi	0.8
Map59	F82	F64	Lost invest	Increased p	0.8
Map59	F82	F63	Lost invest	Increased c	0.6
Map59	F82	F96	Lost invest	More hung	0.8
Map59	F07	F82	Businesses	Lost invest	0.6
Map59	F07	F01	Businesses	Bad experi	0.6
Map59	F07	F64	Businesses	Increased p	0.6
Map59	F07	F63	Businesses	Increased c	0.6
Map59	F07	F96	Businesses	More hung	0.6
Map59	F20	F01	Difficult bre	Bad experi	0.2
Map59	F29	F20	Fear of cor	Difficult bre	0.4
Map59	F29	F01	Fear of cor	Bad experi	0.6
Map59	F29	F154	Fear of cor	Wearing fa	0.6
Map59	F29	F30	Fear of cor	Fear of dea	0.8
Map59	F64	F01	Increased p	Bad experi	0.8
Map59	F64	F63	Increased p	Increased c	0.6
Map59	F64	F96	Increased p	More hung	0.8
Map59	F63	F01	Increased c	Bad experi	0.4
Map59	F62	F01	Increased r	Bad experi	0.4
Map59	F154	F20	Wearing fa	Difficult bre	0.4
Map59	F154	F01	Wearing fa	Bad experi	0.2
Map59	F157	F01	Women un	Bad experi	0.2
Map59	F157	F118	Women un	No social g	0.6
Map59	F157	F32	Women un	Feeling soc	0.4
Map59	F158	F01	Youth/child	Bad experi	0.6
Map59	F158	F95	Youth/child	More hous	0.6
Map59	F158	F133	Youth/child	Poor perfor	0.6
Map59	F158	F16	Youth/child	Delay in ed	0.8
Map59	F158	F147	Youth/child	Stress and	0.6
Map59	F96	F01	More hung	Bad experi	1
Map59	F96	F63	More hung	Increased c	0.6
Map59	F95	F01	More hous	Bad experi	0.4
Map59	F95	F147	More hous	Stress and	0.4
Map59	F118	F01	No social g	Bad experi	0.2
Map59	F118	F32	No social g	Feeling soc	0.4
Map59	F98	F01	More mate	Bad experi	0.6
Map59	F137	F47	Reduced s	Increase in	0.4
Map59	F137	F01	Reduced s	Bad experi	0.4
Map59	F30	F01	Fear of dea	Bad experi	0.6
Map59	F32	F01	Feeling soc	Bad experi	0.2

For Peer Review

Map59	F133	F01	Poor perfo	Bad experi	0.6
Map59	F133	F147	Poor perfo	Stress and	0.6
Map59	F38	F01	Health wor	Bad experi	0.6
Map59	F38	F98	Health wor	More mate	0.8
Map59	F16	F01	Delay in ed	Bad experi	0.6
Map59	F16	F133	Delay in ed	Poor perfo	0.6
Map59	F16	F147	Delay in ed	Stress and	0.6
Map59	F142	F01	School/ma	Bad experi	0.6
Map59	F142	F158	School/ma	Youth/child	0.8
Map59	F142	F95	School/ma	More hous	0.6
Map59	F142	F133	School/ma	Poor perfo	0.6
Map59	F142	F16	School/ma	Delay in ed	0.8
Map59	F142	F147	School/ma	Stress and	0.6
Map59	F140	F01	Restriction	Bad experi	0.6
Map59	F140	F157	Restriction	Women un	0.8
Map59	F140	F118	Restriction	No social g	0.6
Map59	F140	F98	Restriction	More mate	0.8
Map59	F140	F32	Restriction	Feeling soc	0.4
Map59	F140	F38	Restriction	Health wor	1
Map59	F140	F120	Restriction	No transpo	1
Map59	F147	F01	Stress and	Bad experi	0.6
Map59	F120	F01	No transpo	Bad experi	0.6
Map59	F120	F157	No transpo	Women un	0.8
Map59	F120	F118	No transpo	No social g	0.6
Map59	F120	F98	No transpo	More mate	0.8
Map59	F120	F32	No transpo	Feeling soc	0.4
Map59	F120	F38	No transpo	Health wor	1
Map59	F115	F01	No preachi	Bad experi	0.4
Map59	F115	F62	No preachi	Increased r	0.4
Map59	F106	F01	No congreg	Bad experi	0.4
Map59	F106	F62	No congreg	Increased r	0.4
Map59	F106	F115	No congreg	No preachi	0.6
Map59	F100	F01	Mosques/c	Bad experi	0.4
Map59	F100	F62	Mosques/c	Increased r	0.4
Map59	F100	F115	Mosques/c	No preachi	0.6
Map59	F100	F106	Mosques/c	No congreg	0.8
Map60	F40	F47	Household	Increase in	0.8
Map60	F40	F01	Household	Bad experi	0.8
Map60	F88	F40	Men unabl	Household	0.4
Map60	F88	F47	Men unabl	Increase in	0.4
Map60	F88	F01	Men unabl	Bad experi	0.4
Map60	F47	F01	Increase in	Bad experi	1
Map60	F85	F40	Markets/sh	Household	0.4
Map60	F85	F88	Markets/sh	Men unabl	0.8
Map60	F85	F47	Markets/sh	Increase in	0.4
Map60	F85	F87	Markets/sh	Men stayin	0.8
Map60	F85	F83	Markets/sh	Malnutritior	0.4
Map60	F85	F82	Markets/sh	Lost invest	0.8
Map60	F85	F07	Markets/sh	Businesses	1
Map60	F85	F01	Markets/sh	Bad experi	0.4
Map60	F85	F64	Markets/sh	Increased p	0.8
Map60	F85	F96	Markets/sh	More hung	0.4
Map60	F85	F132	Markets/sh	Poor harve	0.4
Map60	F85	F78	Markets/sh	Less food t	0.4
Map60	F85	F70	Markets/sh	Jobs lost	0.8
Map60	F85	F101	Markets/sh	No access	0.4

For Peer Review

Map60	F87	F40	Men stayin	Household	0.4
Map60	F87	F88	Men stayin	Men unabl	0.8
Map60	F87	F47	Men stayin	Increase in	0.4
Map60	F87	F01	Men stayin	Bad experi	0.4
Map60	F80	F40	Lockdown	Household	0.4
Map60	F80	F88	Lockdown	Men unabl	0.8
Map60	F80	F47	Lockdown	Increase in	0.4
Map60	F80	F85	Lockdown	Markets/sh	0.8
Map60	F80	F87	Lockdown	Men stayin	0.8
Map60	F80	F83	Lockdown	Malnutritior	0.4
Map60	F80	F82	Lockdown	Lost invest	0.8
Map60	F80	F07	Lockdown	Businesses	0.8
Map60	F80	F01	Lockdown	Bad experi	0.8
Map60	F80	F67	Lockdown	Increased s	0.6
Map60	F80	F64	Lockdown	Increased p	0.8
Map60	F80	F60	Lockdown	Increased i	0.6
Map60	F80	F158	Lockdown	Youth/child	0.6
Map60	F80	F96	Lockdown	More hung	0.4
Map60	F80	F132	Lockdown	Poor harve	0.4
Map60	F80	F51	Lockdown	Increase in	0.6
Map60	F80	F58	Lockdown	Increased c	0.6
Map60	F80	F78	Lockdown	Less food t	0.4
Map60	F80	F70	Lockdown	Jobs lost	0.8
Map60	F80	F142	Lockdown	School/ma	0.6
Map60	F80	F147	Lockdown	Stress and	0.8
Map60	F80	F144	Lockdown	Social dista	0.8
Map60	F80	F106	Lockdown	No congreg	0.8
Map60	F80	F101	Lockdown	No access	0.4
Map60	F80	F100	Lockdown	Mosques/c	0.8
Map60	F83	F01	Malnutritior	Bad experi	0.8
Map60	F82	F40	Lost invest	Household	0.4
Map60	F82	F88	Lost invest	Men unabl	0.6
Map60	F82	F47	Lost invest	Increase in	0.4
Map60	F82	F01	Lost invest	Bad experi	0.4
Map60	F82	F64	Lost invest	Increased p	0.8
Map60	F07	F40	Businesses	Household	0.4
Map60	F07	F88	Businesses	Men unabl	0.8
Map60	F07	F47	Businesses	Increase in	0.4
Map60	F07	F87	Businesses	Men stayin	0.8
Map60	F07	F82	Businesses	Lost invest	0.8
Map60	F07	F01	Businesses	Bad experi	0.4
Map60	F07	F64	Businesses	Increased p	0.8
Map60	F07	F70	Businesses	Jobs lost	0.8
Map60	F20	F01	Difficult bre	Bad experi	0.6
Map60	F67	F01	Increased s	Bad experi	1
Map60	F67	F147	Increased s	Stress and	1
Map60	F29	F20	Fear of cor	Difficult bre	0.2
Map60	F29	F01	Fear of cor	Bad experi	0.6
Map60	F29	F63	Fear of cor	Increased c	0.6
Map60	F29	F38	Fear of cor	Health wor	0.6
Map60	F29	F10	Fear of cor	Compulsor	0.4
Map60	F64	F40	Increased p	Household	0.4
Map60	F64	F88	Increased p	Men unabl	0.6
Map60	F64	F47	Increased p	Increase in	0.4
Map60	F64	F01	Increased p	Bad experi	0.4
Map60	F63	F01	Increased c	Bad experi	1

For Peer Review

Map60	F60	F01	Increased i	Bad experi	0.8
Map60	F60	F67	Increased i	Increased s	0.8
Map60	F60	F58	Increased i	Increased c	0.8
Map60	F60	F147	Increased i	Stress and	0.8
Map60	F158	F01	Youth/child	Bad experi	0.6
Map60	F158	F67	Youth/child	Increased s	0.6
Map60	F158	F60	Youth/child	Increased i	0.6
Map60	F158	F51	Youth/child	Increase in	0.6
Map60	F158	F58	Youth/child	Increased c	0.6
Map60	F158	F147	Youth/child	Stress and	0.6
Map60	F96	F83	More hung	Malnutrition	0.8
Map60	F96	F01	More hung	Bad experi	1
Map60	F132	F40	Poor harve	Household	0.4
Map60	F132	F88	Poor harve	Men unable	0.6
Map60	F132	F47	Poor harve	Increase in	0.4
Map60	F132	F83	Poor harve	Malnutrition	0.6
Map60	F132	F82	Poor harve	Lost invest	0.8
Map60	F132	F01	Poor harve	Bad experi	0.6
Map60	F132	F64	Poor harve	Increased p	0.8
Map60	F132	F96	Poor harve	More hung	0.6
Map60	F132	F78	Poor harve	Less food t	0.6
Map60	F51	F01	Increase in	Bad experi	0.6
Map60	F51	F67	Increase in	Increased s	0.6
Map60	F51	F60	Increase in	Increased i	0.6
Map60	F51	F58	Increase in	Increased c	0.6
Map60	F51	F147	Increase in	Stress and	0.6
Map60	F58	F01	Increased c	Bad experi	0.8
Map60	F58	F67	Increased c	Increased s	0.8
Map60	F58	F147	Increased c	Stress and	0.8
Map60	F78	F83	Less food t	Malnutrition	0.8
Map60	F78	F01	Less food t	Bad experi	0.8
Map60	F78	F96	Less food t	More hung	0.8
Map60	F38	F01	Health wor	Bad experi	0.8
Map60	F38	F63	Health wor	Increased c	0.8
Map60	F10	F20	Compulsor	Difficult bre	0.2
Map60	F10	F01	Compulsor	Bad experi	0.2
Map60	F70	F40	Jobs lost	Household	0.4
Map60	F70	F88	Jobs lost	Men unable	0.8
Map60	F70	F47	Jobs lost	Increase in	0.4
Map60	F70	F87	Jobs lost	Men stayin	0.8
Map60	F70	F01	Jobs lost	Bad experi	0.4
Map60	F70	F64	Jobs lost	Increased p	0.6
Map60	F142	F01	School/ma	Bad experi	0.6
Map60	F142	F67	School/ma	Increased s	0.6
Map60	F142	F60	School/ma	Increased i	0.6
Map60	F142	F158	School/ma	Youth/child	0.8
Map60	F142	F51	School/ma	Increase in	0.6
Map60	F142	F58	School/ma	Increased c	0.6
Map60	F142	F147	School/ma	Stress and	0.6
Map60	F147	F01	Stress and	Bad experi	1
Map60	F144	F01	Social dista	Bad experi	0.8
Map60	F144	F147	Social dista	Stress and	0.8
Map60	F144	F106	Social dista	No congreg	0.8
Map60	F106	F01	No congreg	Bad experi	1
Map60	F106	F147	No congreg	Stress and	1
Map60	F101	F40	No access	Household	0.4

For Peer Review

Map60	F101	F88	No access	Men unabl	0.4
Map60	F101	F47	No access	Increase in	0.4
Map60	F101	F83	No access	Malnutritior	0.4
Map60	F101	F82	No access	Lost invest	0.4
Map60	F101	F01	No access	Bad experi	0.4
Map60	F101	F64	No access	Increased p	0.4
Map60	F101	F96	No access	More hung	0.4
Map60	F101	F132	No access	Poor harve	0.4
Map60	F101	F78	No access	Less food t	0.4
Map60	F100	F01	Mosques/c	Bad experi	0.8
Map60	F100	F147	Mosques/c	Stress and	0.8
Map60	F100	F144	Mosques/c	Social dista	0.8
Map60	F100	F106	Mosques/c	No congreg	0.8
Map61	F40	F47	Household	Increase in	0.8
Map61	F40	F01	Household	Bad experi	0.8
Map61	F47	F01	Increase in	Bad experi	0.8
Map61	F85	F40	Markets/sh	Household	0.6
Map61	F85	F47	Markets/sh	Increase in	0.6
Map61	F85	F82	Markets/sh	Lost invest	0.6
Map61	F85	F07	Markets/sh	Businesses	0.6
Map61	F85	F01	Markets/sh	Bad experi	0.6
Map61	F85	F64	Markets/sh	Increased p	0.6
Map61	F85	F63	Markets/sh	Increased c	0.6
Map61	F85	F155	Markets/sh	Wives ran a	0.6
Map61	F85	F96	Markets/sh	More hung	0.6
Map61	F85	F135	Markets/sh	Price hike	0.6
Map61	F85	F78	Markets/sh	Less food t	0.6
Map61	F85	F143	Markets/sh	Shortage o	0.6
Map61	F80	F40	Lockdown	Household	0.6
Map61	F80	F47	Lockdown	Increase in	0.6
Map61	F80	F85	Lockdown	Markets/sh	1
Map61	F80	F82	Lockdown	Lost invest	0.6
Map61	F80	F07	Lockdown	Businesses	0.6
Map61	F80	F01	Lockdown	Bad experi	0.8
Map61	F80	F64	Lockdown	Increased p	0.6
Map61	F80	F63	Lockdown	Increased c	0.6
Map61	F80	F62	Lockdown	Increased r	0.8
Map61	F80	F155	Lockdown	Wives ran a	0.6
Map61	F80	F158	Lockdown	Youth/child	0.8
Map61	F80	F96	Lockdown	More hung	0.6
Map61	F80	F110	Lockdown	No funerals	1
Map61	F80	F135	Lockdown	Price hike	0.6
Map61	F80	F124	Lockdown	Not visiting	0.6
Map61	F80	F58	Lockdown	Increased c	0.6
Map61	F80	F32	Lockdown	Feeling soc	0.8
Map61	F80	F78	Lockdown	Less food t	0.6
Map61	F80	F16	Lockdown	Delay in ec	0.6
Map61	F80	F143	Lockdown	Shortage o	0.6
Map61	F80	F142	Lockdown	School/mac	0.8
Map61	F80	F147	Lockdown	Stress and	0.8
Map61	F80	F120	Lockdown	No transpo	0.6
Map61	F80	F144	Lockdown	Social dista	0.8
Map61	F80	F106	Lockdown	No congreg	0.8
Map61	F82	F01	Lost invest	Bad experi	0.8
Map61	F82	F64	Lost invest	Increased p	0.8
Map61	F07	F82	Businesses	Lost invest	0.8

For Peer Review

Map61	F07	F01	Businesses	Bad experi	0.8
Map61	F07	F64	Businesses	Increased p	0.8
Map61	F20	F01	Difficult bre	Bad experi	0.8
Map61	F29	F20	Fear of cor	Difficult bre	0.6
Map61	F29	F01	Fear of cor	Bad experi	0.6
Map61	F29	F30	Fear of cor	Fear of dea	0.4
Map61	F29	F31	Fear of cor	Fear of eac	0.6
Map61	F29	F10	Fear of cor	Compulsor	0.6
Map61	F64	F01	Increased p	Bad experi	1
Map61	F63	F01	Increased (	Bad experi	1
Map61	F62	F01	Increased r	Bad experi	1
Map61	F155	F01	Wives ran a	Bad experi	1
Map61	F158	F01	Youth/child	Bad experi	0.6
Map61	F158	F58	Youth/child	Increased (	0.6
Map61	F158	F16	Youth/child	Delay in ec	0.6
Map61	F96	F01	More hung	Bad experi	1
Map61	F96	F63	More hung	Increased (	1
Map61	F96	F155	More hung	Wives ran a	0.8
Map61	F115	F01	No preachi	Bad experi	0.6
Map61	F115	F62	No preachi	Increased r	0.6
Map61	F115	F147	No preachi	Stress and	0.6
Map61	F110	F01	No funerals	Bad experi	0.6
Map61	F110	F62	No funerals	Increased r	0.6
Map61	F110	F147	No funerals	Stress and	0.6
Map61	F135	F40	Price hike	Household	0.6
Map61	F135	F47	Price hike	Increase in	0.6
Map61	F135	F01	Price hike	Bad experi	0.8
Map61	F135	F63	Price hike	Increased (	0.8
Map61	F135	F155	Price hike	Wives ran a	0.8
Map61	F135	F96	Price hike	More hung	0.8
Map61	F135	F78	Price hike	Less food t	0.8
Map61	F124	F01	Not visiting	Bad experi	0.8
Map61	F124	F63	Not visiting	Increased (	0.8
Map61	F58	F01	Increased (	Bad experi	1
Map61	F30	F01	Fear of dea	Bad experi	0.6
Map61	F31	F20	Fear of eac	Difficult bre	0.6
Map61	F31	F01	Fear of eac	Bad experi	0.6
Map61	F31	F30	Fear of eac	Fear of dea	0.4
Map61	F31	F10	Fear of eac	Compulsor	0.6
Map61	F32	F01	Feeling soc	Bad experi	1
Map61	F32	F62	Feeling soc	Increased r	1
Map61	F78	F40	Less food t	Household	0.6
Map61	F78	F47	Less food t	Increase in	0.6
Map61	F78	F01	Less food t	Bad experi	1
Map61	F78	F63	Less food t	Increased (	0.8
Map61	F78	F155	Less food t	Wives ran a	0.8
Map61	F78	F96	Less food t	More hung	0.8
Map61	F121	F01	No wedding	Bad experi	0.2
Map61	F121	F62	No wedding	Increased r	0.2
Map61	F121	F147	No wedding	Stress and	0.2
Map61	F10	F20	Compulsor	Difficult bre	0.6
Map61	F10	F01	Compulsor	Bad experi	0.6
Map61	F16	F01	Delay in ec	Bad experi	0.6
Map61	F143	F40	Shortage o	Household	0.6
Map61	F143	F47	Shortage o	Increase in	0.6
Map61	F143	F01	Shortage o	Bad experi	0.8

For Peer Review

Map61	F143	F63	Shortage o	Increased	0.8
Map61	F143	F155	Shortage o	Wives ran	0.8
Map61	F143	F96	Shortage o	More hung	0.8
Map61	F143	F135	Shortage o	Price hike	1
Map61	F143	F78	Shortage o	Less food t	0.8
Map61	F142	F01	School/ma	Bad experi	0.6
Map61	F142	F158	School/ma	Youth/child	1
Map61	F142	F58	School/ma	Increased	0.6
Map61	F142	F16	School/ma	Delay in ec	0.6
Map61	F147	F01	Stress and	Bad experi	1
Map61	F147	F62	Stress and	Increased	0.6
Map61	F120	F01	No transpo	Bad experi	0.6
Map61	F120	F63	No transpo	Increased	0.6
Map61	F120	F124	No transpo	Not visiting	0.6
Map61	F144	F01	Social dista	Bad experi	0.8
Map61	F144	F62	Social dista	Increased	0.8
Map61	F144	F32	Social dista	Feeling soc	0.8
Map61	F144	F147	Social dista	Stress and	0.8
Map61	F144	F106	Social dista	No congreg	1
Map61	F106	F01	No congreg	Bad experi	0.8
Map61	F106	F62	No congreg	Increased	0.6
Map61	F106	F147	No congreg	Stress and	0.8
Map62	F88	F01	Men unabl	Bad experi	0.6
Map62	F88	F96	Men unabl	More hung	0.6
Map62	F88	F78	Men unabl	Less food t	0.6
Map62	F85	F88	Markets/sh	Men unabl	0.6
Map62	F85	F87	Markets/sh	Men stayin	0.6
Map62	F85	F01	Markets/sh	Bad experi	0.6
Map62	F85	F67	Markets/sh	Increased	0.6
Map62	F85	F64	Markets/sh	Increased	0.6
Map62	F85	F158	Markets/sh	Youth/child	0.6
Map62	F85	F96	Markets/sh	More hung	0.6
Map62	F85	F130	Markets/sh	Physical in	0.6
Map62	F85	F78	Markets/sh	Less food t	0.6
Map62	F85	F70	Markets/sh	Jobs lost	0.6
Map62	F87	F88	Men stayin	Men unabl	0.8
Map62	F87	F01	Men stayin	Bad experi	0.8
Map62	F87	F64	Men stayin	Increased	0.8
Map62	F87	F96	Men stayin	More hung	0.6
Map62	F87	F78	Men stayin	Less food t	0.6
Map62	F80	F88	Lockdown	Men unabl	0.6
Map62	F80	F85	Lockdown	Markets/sh	0.8
Map62	F80	F87	Lockdown	Men stayin	0.6
Map62	F80	F01	Lockdown	Bad experi	0.8
Map62	F80	F67	Lockdown	Increased	0.6
Map62	F80	F66	Lockdown	Increased	0.6
Map62	F80	F64	Lockdown	Increased	0.6
Map62	F80	F63	Lockdown	Increased	0.4
Map62	F80	F158	Lockdown	Youth/child	0.6
Map62	F80	F16	Lockdown	Delay in ec	0.8
Map62	F80	F96	Lockdown	More hung	0.6
Map62	F80	F133	Lockdown	Poor perfor	0.8
Map62	F80	F130	Lockdown	Physical in	0.6
Map62	F80	F99	Lockdown	More unwa	0.4
Map62	F80	F112	Lockdown	No home/p	0.6
Map62	F80	F54	Lockdown	Increase in	0.4

For Peer Review

Map62	F80	F118	Lockdown	No social g	0.6
Map62	F80	F78	Lockdown	Less food t	0.6
Map62	F80	F70	Lockdown	Jobs lost	0.6
Map62	F80	F142	Lockdown	School/ma	0.8
Map62	F80	F140	Lockdown	Restriction	0.6
Map62	F80	F147	Lockdown	Stress and	0.8
Map62	F80	F144	Lockdown	Social dista	0.8
Map62	F80	F106	Lockdown	No congreg	0.8
Map62	F80	F104	Lockdown	No buyers	0.6
Map62	F80	F100	Lockdown	Mosques/c	0.8
Map62	F67	F01	Increased s	Bad experi	0.6
Map62	F67	F130	Increased s	Physical in	0.8
Map62	F66	F01	Increased r	Bad experi	0.6
Map62	F66	F130	Increased r	Physical in	0.6
Map62	F29	F01	Fear of cor	Bad experi	0.2
Map62	F29	F150	Fear of cor	Temperatu	0.4
Map62	F29	F31	Fear of cor	Fear of eac	0.6
Map62	F29	F147	Fear of cor	Stress and	0.2
Map62	F64	F88	Increased p	Men unable	0.6
Map62	F64	F01	Increased p	Bad experi	1
Map62	F64	F96	Increased p	More hung	0.6
Map62	F64	F78	Increased p	Less food t	0.6
Map62	F63	F01	Increased c	Bad experi	0.8
Map62	F150	F01	Temperatu	Bad experi	0.2
Map62	F150	F147	Temperatu	Stress and	0.2
Map62	F158	F01	Youth/child	Bad experi	0.6
Map62	F158	F67	Youth/child	Increased s	0.6
Map62	F158	F130	Youth/child	Physical in	0.6
Map62	F158	F70	Youth/child	Jobs lost	0.6
Map62	F16	F01	Delay in ed	Bad experi	0.4
Map62	F16	F133	Delay in ed	Poor perfor	0.8
Map62	F96	F01	More hung	Bad experi	1
Map62	F133	F01	Poor perfor	Bad experi	0.4
Map62	F130	F01	Physical in	Bad experi	0.6
Map62	F99	F01	More unwa	Bad experi	0.6
Map62	F112	F01	No home/p	Bad experi	0.4
Map62	F112	F63	No home/p	Increased c	0.4
Map62	F112	F99	No home/p	More unwa	0.4
Map62	F112	F54	No home/p	Increase in	0.4
Map62	F54	F01	Increase in	Bad experi	0.6
Map62	F54	F63	Increase in	Increased c	0.6
Map62	F54	F99	Increase in	More unwa	0.8
Map62	F31	F01	Fear of eac	Bad experi	0.2
Map62	F31	F150	Fear of eac	Temperatu	0.4
Map62	F31	F147	Fear of eac	Stress and	0.2
Map62	F118	F01	No social g	Bad experi	1
Map62	F118	F147	No social g	Stress and	1
Map62	F78	F01	Less food t	Bad experi	0.8
Map62	F78	F96	Less food t	More hung	0.8
Map62	F70	F01	Jobs lost	Bad experi	0.6
Map62	F70	F67	Jobs lost	Increased s	0.6
Map62	F70	F130	Jobs lost	Physical in	0.6
Map62	F142	F01	School/ma	Bad experi	0.6
Map62	F142	F67	School/ma	Increased s	0.6
Map62	F142	F66	School/ma	Increased r	0.6
Map62	F142	F158	School/ma	Youth/child	0.6

For Peer Review

Map62	F142	F16	School/ma	Delay in ec	1
Map62	F142	F133	School/ma	Poor perfor	0.8
Map62	F142	F130	School/ma	Physical in	0.6
Map62	F142	F70	School/ma	Jobs lost	0.6
Map62	F140	F88	Restriction	Men unable	0.8
Map62	F140	F87	Restriction	Men stayin	1
Map62	F140	F01	Restriction	Bad experi	0.8
Map62	F140	F64	Restriction	Increased p	0.8
Map62	F140	F96	Restriction	More hung	0.6
Map62	F140	F78	Restriction	Less food t	0.6
Map62	F147	F01	Stress and	Bad experi	1
Map62	F144	F01	Social dista	Bad experi	0.6
Map62	F144	F63	Social dista	Increased c	0.4
Map62	F144	F99	Social dista	More unwa	0.4
Map62	F144	F112	Social dista	No home/p	0.6
Map62	F144	F54	Social dista	Increase in	0.4
Map62	F144	F118	Social dista	No social g	0.6
Map62	F144	F147	Social dista	Stress and	0.6
Map62	F144	F104	Social dista	No buyers	0.6
Map62	F106	F01	No congreg	Bad experi	0.8
Map62	F106	F147	No congreg	Stress and	0.8
Map62	F104	F01	No buyers	Bad experi	0.4
Map62	F104	F63	No buyers	Increased c	0.4
Map62	F104	F99	No buyers	More unwa	0.4
Map62	F104	F112	No buyers	No home/p	0.8
Map62	F104	F54	No buyers	Increase in	0.4
Map62	F100	F01	Mosques/c	Bad experi	0.8
Map62	F100	F147	Mosques/c	Stress and	0.8
Map62	F100	F106	Mosques/c	No congreg	1
Map63	F85	F07	Markets/sh	Businesses	0.6
Map63	F85	F01	Markets/sh	Bad experi	0.8
Map63	F85	F64	Markets/sh	Increased p	0.8
Map63	F85	F63	Markets/sh	Increased c	0.8
Map63	F85	F152	Markets/sh	Unable to b	0.8
Map63	F85	F96	Markets/sh	More hung	0.8
Map63	F85	F78	Markets/sh	Less food t	0.8
Map63	F85	F74	Markets/sh	Lack of fac	0.2
Map63	F85	F10	Markets/sh	Compulsor	0.4
Map63	F85	F70	Markets/sh	Jobs lost	0.8
Map63	F85	F143	Markets/sh	Shortage o	0.8
Map63	F85	F147	Markets/sh	Stress and	0.4
Map63	F85	F144	Markets/sh	Social dista	0.4
Map63	F85	F106	Markets/sh	No congreg	0.4
Map63	F81	F01	Loss of Ha	Bad experi	0.8
Map63	F81	F62	Loss of Ha	Increased r	0.8
Map63	F80	F85	Lockdown	Markets/sh	0.6
Map63	F80	F81	Lockdown	Loss of Ha	0.6
Map63	F80	F07	Lockdown	Businesses	0.6
Map63	F80	F06	Lockdown	Borders clo	1
Map63	F80	F01	Lockdown	Bad experi	0.6
Map63	F80	F64	Lockdown	Increased p	0.6
Map63	F80	F63	Lockdown	Increased c	0.6
Map63	F80	F62	Lockdown	Increased r	0.6
Map63	F80	F152	Lockdown	Unable to b	0.6
Map63	F80	F158	Lockdown	Youth/child	0.8
Map63	F80	F16	Lockdown	Delay in ec	0.8

For Peer Review

Map63	F80	F96	Lockdown	More hung	0.6
Map63	F80	F114	Lockdown	No pilgrima	1
Map63	F80	F118	Lockdown	No social g	0.6
Map63	F80	F110	Lockdown	No funerals	0.6
Map63	F80	F78	Lockdown	Less food t	0.6
Map63	F80	F133	Lockdown	Poor perfor	0.6
Map63	F80	F74	Lockdown	Lack of fac	0.2
Map63	F80	F121	Lockdown	No weddin	0.6
Map63	F80	F10	Lockdown	Compulsor	0.4
Map63	F80	F70	Lockdown	Jobs lost	0.6
Map63	F80	F143	Lockdown	Shortage o	0.6
Map63	F80	F142	Lockdown	School/ma	1
Map63	F80	F140	Lockdown	Restriction	0.8
Map63	F80	F147	Lockdown	Stress and	0.4
Map63	F80	F144	Lockdown	Social dista	0.4
Map63	F80	F106	Lockdown	No congreg	0.4
Map63	F38	F01	Health wor	Bad experi	0.6
Map63	F38	F63	Health wor	Increased	0.6
Map63	F38	F14	Health wor	Could not g	0.6
Map63	F07	F01	Businesses	Bad experi	0.8
Map63	F07	F64	Businesses	Increased p	0.8
Map63	F07	F63	Businesses	Increased	0.8
Map63	F07	F152	Businesses	Unable to b	0.8
Map63	F07	F96	Businesses	More hung	0.8
Map63	F07	F78	Businesses	Less food t	0.8
Map63	F06	F81	Borders clo	Loss of Ha	0.6
Map63	F06	F06	Borders clo	Borders clo	0.8
Map63	F06	F01	Borders clo	Bad experi	0.6
Map63	F06	F62	Borders clo	Increased	0.6
Map63	F06	F114	Borders clo	No pilgrima	1
Map63	F29	F38	Fear of cor	Health wor	0.4
Map63	F29	F01	Fear of cor	Bad experi	0.4
Map63	F29	F63	Fear of cor	Increased	0.4
Map63	F29	F31	Fear of cor	Fear of eac	0.6
Map63	F29	F14	Fear of cor	Could not g	0.4
Map63	F29	F147	Fear of cor	Stress and	0.4
Map63	F29	F128	Fear of cor	People fee	1
Map63	F64	F01	Increased p	Bad experi	0.8
Map63	F64	F63	Increased p	Increased	0.8
Map63	F64	F152	Increased p	Unable to b	0.8
Map63	F64	F96	Increased p	More hung	1
Map63	F64	F78	Increased p	Less food t	1
Map63	F63	F01	Increased	Bad experi	1
Map63	F62	F01	Increased	Bad experi	0.8
Map63	F152	F01	Unable to b	Bad experi	1
Map63	F152	F63	Unable to b	Increased	1
Map63	F158	F01	Youth/child	Bad experi	0.6
Map63	F158	F16	Youth/child	Delay in ec	0.8
Map63	F158	F133	Youth/child	Poor perfor	0.6
Map63	F16	F01	Delay in ec	Bad experi	0.6
Map63	F16	F133	Delay in ec	Poor perfor	0.6
Map63	F96	F01	More hung	Bad experi	0.6
Map63	F96	F63	More hung	Increased	0.6
Map63	F114	F81	No pilgrima	Loss of Ha	0.6
Map63	F114	F01	No pilgrima	Bad experi	0.6
Map63	F114	F62	No pilgrima	Increased	0.6

For Peer Review

Map63	F118	F01	No social g	Bad experi	0.6
Map63	F118	F62	No social g	Increased r	0.6
Map63	F118	F110	No social g	No funerals	0.6
Map63	F118	F121	No social g	No wedding	0.6
Map63	F110	F01	No funerals	Bad experi	0.6
Map63	F110	F62	No funerals	Increased r	0.6
Map63	F31	F01	Fear of eac	Bad experi	0.4
Map63	F31	F147	Fear of eac	Stress and	0.4
Map63	F78	F01	Less food t	Bad experi	0.6
Map63	F78	F63	Less food t	Increased c	0.6
Map63	F78	F96	Less food t	More hung	1
Map63	F133	F01	Poor perfor	Bad experi	0.6
Map63	F74	F01	Lack of fac	Bad experi	0.4
Map63	F74	F147	Lack of fac	Stress and	0.4
Map63	F121	F01	No wedding	Bad experi	0.8
Map63	F121	F62	No wedding	Increased r	0.8
Map63	F10	F01	Compulsor	Bad experi	0.2
Map63	F10	F74	Compulsor	Lack of fac	0.2
Map63	F10	F147	Compulsor	Stress and	0.2
Map63	F70	F01	Jobs lost	Bad experi	0.8
Map63	F70	F64	Jobs lost	Increased p	0.8
Map63	F70	F63	Jobs lost	Increased c	0.8
Map63	F70	F152	Jobs lost	Unable to b	0.8
Map63	F70	F96	Jobs lost	More hung	0.8
Map63	F70	F78	Jobs lost	Less food t	0.8
Map63	F14	F01	Could not g	Bad experi	0.8
Map63	F14	F63	Could not g	Increased c	0.8
Map63	F143	F01	Shortage o	Bad experi	0.6
Map63	F143	F63	Shortage o	Increased c	0.6
Map63	F143	F96	Shortage o	More hung	0.8
Map63	F143	F78	Shortage o	Less food t	0.8
Map63	F142	F01	School/ma	Bad experi	0.6
Map63	F142	F158	School/ma	Youth/child	0.8
Map63	F142	F16	School/ma	Delay in ec	0.8
Map63	F142	F133	School/ma	Poor perfor	0.6
Map63	F140	F01	Restriction	Bad experi	0.6
Map63	F140	F62	Restriction	Increased r	0.6
Map63	F140	F118	Restriction	No social g	0.6
Map63	F140	F110	Restriction	No funerals	0.6
Map63	F140	F121	Restriction	No wedding	0.6
Map63	F147	F01	Stress and	Bad experi	0.8
Map63	F144	F01	Social dista	Bad experi	0.6
Map63	F144	F147	Social dista	Stress and	0.6
Map63	F128	F01	People feel	Bad experi	0.4
Map63	F128	F31	People feel	Fear of eac	0.6
Map63	F128	F147	People feel	Stress and	0.4
Map63	F106	F01	No congreg	Bad experi	0.6
Map63	F106	F74	No congreg	Lack of fac	0.2
Map63	F106	F10	No congreg	Compulsor	0.4
Map63	F106	F147	No congreg	Stress and	0.6
Map63	F106	F144	No congreg	Social dista	0.6
Map64	F40	F47	Household	Increase in	0.8
Map64	F40	F01	Household	Bad experi	0.6
Map64	F47	F01	Increase in	Bad experi	0.6
Map64	F85	F82	Markets/sh	Lost invest	0.8
Map64	F85	F07	Markets/sh	Businesses	0.8

For Peer Review

Map64	F85	F01	Markets/sh	Bad experi	0.8
Map64	F85	F64	Markets/sh	Increased p	0.8
Map64	F85	F62	Markets/sh	Increased r	0.8
Map64	F85	F111	Markets/sh	No governn	0.8
Map64	F87	F40	Men stayin	Household	0.6
Map64	F87	F47	Men stayin	Increase in	0.6
Map64	F87	F01	Men stayin	Bad experi	0.6
Map64	F80	F40	Lockdown	Household	0.6
Map64	F80	F47	Lockdown	Increase in	0.6
Map64	F80	F83	Lockdown	Malnutritior	0.6
Map64	F80	F22	Lockdown	Difficulty in	0.4
Map64	F80	F01	Lockdown	Bad experi	0.6
Map64	F80	F63	Lockdown	Increased c	0.6
Map64	F80	F62	Lockdown	Increased r	0.6
Map64	F80	F158	Lockdown	Youth/child	0.4
Map64	F80	F16	Lockdown	Delay in ec	0.4
Map64	F80	F96	Lockdown	More hunge	0.6
Map64	F80	F130	Lockdown	Physical in	0.4
Map64	F80	F110	Lockdown	No funerals	0.8
Map64	F80	F135	Lockdown	Price hike	0.6
Map64	F80	F50	Lockdown	Increase in	0.6
Map64	F80	F124	Lockdown	Not visiting	1
Map64	F80	F78	Lockdown	Less food t	0.6
Map64	F80	F133	Lockdown	Poor perfor	0.4
Map64	F80	F98	Lockdown	More mate	0.6
Map64	F80	F143	Lockdown	Shortage o	0.6
Map64	F80	F142	Lockdown	School/mac	0.6
Map64	F80	F147	Lockdown	Stress and	0.6
Map64	F80	F120	Lockdown	No transpo	1
Map64	F80	F106	Lockdown	No congreg	0.4
Map64	F83	F01	Malnutritior	Bad experi	0.8
Map64	F83	F63	Malnutritior	Increased c	0.8
Map64	F82	F01	Lost invest	Bad experi	0.8
Map64	F82	F64	Lost invest	Increased p	1
Map64	F82	F62	Lost invest	Increased r	0.8
Map64	F82	F111	Lost invest	No governn	0.8
Map64	F22	F01	Difficulty in	Bad experi	0.8
Map64	F22	F62	Difficulty in	Increased r	0.8
Map64	F22	F147	Difficulty in	Stress and	0.8
Map64	F22	F106	Difficulty in	No congreg	1
Map64	F07	F82	Businesses	Lost invest	0.8
Map64	F07	F01	Businesses	Bad experi	0.8
Map64	F07	F64	Businesses	Increased p	0.8
Map64	F07	F62	Businesses	Increased r	0.8
Map64	F07	F111	Businesses	No governn	0.8
Map64	F20	F01	Difficult bre	Bad experi	0.2
Map64	F29	F20	Fear of cor	Difficult bre	0.4
Map64	F29	F01	Fear of cor	Bad experi	0.8
Map64	F29	F62	Fear of cor	Increased r	0.6
Map64	F29	F30	Fear of cor	Fear of dea	0.4
Map64	F29	F31	Fear of cor	Fear of eac	0.8
Map64	F29	F32	Fear of cor	Feeling soc	0.6
Map64	F29	F10	Fear of cor	Compulsor	0.4
Map64	F29	F147	Fear of cor	Stress and	0.6
Map64	F29	F144	Fear of cor	Social dista	1
Map64	F64	F01	Increased p	Bad experi	0.8

For Peer Review

Map64	F64	F62	Increased p	Increased r	0.8
Map64	F64	F111	Increased p	No governr	0.8
Map64	F63	F01	Increased c	Bad experi	1
Map64	F62	F01	Increased r	Bad experi	0.8
Map64	F158	F22	Youth/child	Difficulty in	0.4
Map64	F158	F01	Youth/child	Bad experi	0.6
Map64	F158	F62	Youth/child	Increased r	0.4
Map64	F158	F16	Youth/child	Delay in ec	0.6
Map64	F158	F130	Youth/child	Physical inj	0.6
Map64	F158	F133	Youth/child	Poor perfor	0.4
Map64	F158	F147	Youth/child	Stress and	0.4
Map64	F158	F106	Youth/child	No congreg	0.4
Map64	F16	F01	Delay in ec	Bad experi	0.8
Map64	F96	F40	More hung	Household	0.8
Map64	F96	F47	More hung	Increase in	0.8
Map64	F96	F01	More hung	Bad experi	1
Map64	F118	F01	No social g	Bad experi	0.6
Map64	F118	F62	No social g	Increased r	0.6
Map64	F118	F147	No social g	Stress and	0.6
Map64	F118	F106	No social g	No congreg	0.6
Map64	F130	F22	Physical inj	Difficulty in	0.4
Map64	F130	F01	Physical inj	Bad experi	0.4
Map64	F130	F62	Physical inj	Increased r	0.4
Map64	F130	F147	Physical inj	Stress and	0.4
Map64	F130	F106	Physical inj	No congreg	0.4
Map64	F110	F01	No funerals	Bad experi	0.6
Map64	F110	F62	No funerals	Increased r	0.6
Map64	F110	F147	No funerals	Stress and	0.6
Map64	F111	F01	No governr	Bad experi	0.8
Map64	F135	F40	Price hike	Household	0.6
Map64	F135	F47	Price hike	Increase in	0.6
Map64	F135	F83	Price hike	Malnutritior	0.6
Map64	F135	F01	Price hike	Bad experi	0.6
Map64	F135	F63	Price hike	Increased c	0.6
Map64	F135	F96	Price hike	More hung	0.6
Map64	F135	F78	Price hike	Less food t	0.6
Map64	F124	F01	Not visiting	Bad experi	0.6
Map64	F124	F50	Not visiting	Increase in	0.6
Map64	F124	F98	Not visiting	More mate	0.6
Map64	F30	F01	Fear of dea	Bad experi	0.6
Map64	F31	F01	Fear of eac	Bad experi	1
Map64	F32	F01	Feeling soc	Bad experi	1
Map64	F32	F62	Feeling soc	Increased r	0.8
Map64	F32	F147	Feeling soc	Stress and	1
Map64	F78	F40	Less food t	Household	0.8
Map64	F78	F47	Less food t	Increase in	0.8
Map64	F78	F83	Less food t	Malnutritior	0.8
Map64	F78	F01	Less food t	Bad experi	0.8
Map64	F78	F63	Less food t	Increased c	0.8
Map64	F78	F96	Less food t	More hung	0.8
Map64	F133	F01	Poor perfor	Bad experi	0.8
Map64	F10	F20	Compulsor	Difficult bre	1
Map64	F10	F01	Compulsor	Bad experi	0.2
Map64	F70	F40	Jobs lost	Household	0.6
Map64	F70	F47	Jobs lost	Increase in	0.6
Map64	F70	F87	Jobs lost	Men stayin	1

For Peer Review

Map64	F70	F01	Jobs lost	Bad experi	0.6
Map64	F98	F01	More mate	Bad experi	0.8
Map64	F143	F40	Shortage o	Household	0.6
Map64	F143	F47	Shortage o	Increase in	0.6
Map64	F143	F83	Shortage o	Malnutrition	0.6
Map64	F143	F01	Shortage o	Bad experi	0.6
Map64	F143	F63	Shortage o	Increased (	0.6
Map64	F143	F96	Shortage o	More hung	0.6
Map64	F143	F135	Shortage o	Price hike	0.8
Map64	F143	F78	Shortage o	Less food t	0.6
Map64	F142	F22	School/ma	Difficulty in	0.4
Map64	F142	F01	School/ma	Bad experi	0.4
Map64	F142	F62	School/ma	Increased r	0.4
Map64	F142	F158	School/ma	Youth/child	0.4
Map64	F142	F16	School/ma	Delay in ec	0.4
Map64	F142	F130	School/ma	Physical in	0.4
Map64	F142	F133	School/ma	Poor perfor	0.4
Map64	F142	F147	School/ma	Stress and	0.4
Map64	F142	F106	School/ma	No congreg	0.4
Map64	F147	F01	Stress and	Bad experi	1
Map64	F147	F62	Stress and	Increased r	0.8
Map64	F120	F40	No transpo	Household	0.6
Map64	F120	F47	No transpo	Increase in	0.6
Map64	F120	F83	No transpo	Malnutrition	0.6
Map64	F120	F01	No transpo	Bad experi	0.6
Map64	F120	F63	No transpo	Increased (	0.6
Map64	F120	F96	No transpo	More hung	0.6
Map64	F120	F135	No transpo	Price hike	0.6
Map64	F120	F50	No transpo	Increase in	0.6
Map64	F120	F124	No transpo	Not visiting	0.8
Map64	F120	F78	No transpo	Less food t	0.6
Map64	F120	F98	No transpo	More mate	0.6
Map64	F120	F143	No transpo	Shortage o	0.6
Map64	F144	F01	Social dista	Bad experi	0.6
Map64	F144	F62	Social dista	Increased r	0.6
Map64	F144	F32	Social dista	Feeling soc	0.6
Map64	F144	F147	Social dista	Stress and	0.6
Map64	F106	F01	No congreg	Bad experi	0.8
Map64	F106	F62	No congreg	Increased r	0.8
Map64	F106	F147	No congreg	Stress and	0.8
Map65	F40	F47	Household	Increase in	0.6
Map65	F40	F01	Household	Bad experi	0.6
Map65	F40	F62	Household	Increased r	0.4
Map65	F88	F40	Men unable	Household	0.6
Map65	F88	F47	Men unable	Increase in	0.6
Map65	F88	F01	Men unable	Bad experi	0.6
Map65	F88	F62	Men unable	Increased r	0.6
Map65	F88	F95	Men unable	More hous	1
Map65	F47	F01	Increase in	Bad experi	0.4
Map65	F47	F62	Increase in	Increased r	0.4
Map65	F85	F82	Markets/sh	Lost invest	0.8
Map65	F85	F07	Markets/sh	Businesses	0.8
Map65	F85	F01	Markets/sh	Bad experi	0.6
Map65	F85	F64	Markets/sh	Increased p	0.6
Map65	F85	F63	Markets/sh	Increased (	0.6
Map65	F85	F96	Markets/sh	More hung	0.6

For Peer Review

Map65	F87	F40	Men stayin	Household	0.6
Map65	F87	F88	Men stayin	Men unable	0.8
Map65	F87	F47	Men stayin	Increase in	0.6
Map65	F87	F01	Men stayin	Bad experi	0.6
Map65	F87	F62	Men stayin	Increased r	0.6
Map65	F87	F95	Men stayin	More house	0.8
Map65	F87	F98	Men stayin	More mate	0.6
Map65	F87	F99	Men stayin	More unwa	0.6
Map65	F80	F40	Lockdown	Household	0.6
Map65	F80	F88	Lockdown	Men unable	0.8
Map65	F80	F47	Lockdown	Increase in	0.6
Map65	F80	F85	Lockdown	Markets/sh	0.8
Map65	F80	F87	Lockdown	Men stayin	0.8
Map65	F80	F82	Lockdown	Lost invest	0.8
Map65	F80	F07	Lockdown	Businesses	0.8
Map65	F80	F01	Lockdown	Bad experi	0.6
Map65	F80	F64	Lockdown	Increased p	0.6
Map65	F80	F63	Lockdown	Increased c	0.6
Map65	F80	F62	Lockdown	Increased r	0.6
Map65	F80	F157	Lockdown	Women un	0.8
Map65	F80	F158	Lockdown	Youth/child	0.8
Map65	F80	F96	Lockdown	More hunge	0.6
Map65	F80	F95	Lockdown	More house	0.8
Map65	F80	F118	Lockdown	No social g	0.6
Map65	F80	F98	Lockdown	More mate	0.6
Map65	F80	F99	Lockdown	More unwa	0.6
Map65	F80	F32	Lockdown	Feeling soc	0.4
Map65	F80	F133	Lockdown	Poor perfor	0.4
Map65	F80	F38	Lockdown	Health wor	0.8
Map65	F80	F13	Lockdown	Could not c	0.6
Map65	F80	F16	Lockdown	Delay in ed	0.6
Map65	F80	F142	Lockdown	School/mac	1
Map65	F80	F140	Lockdown	Restriction	1
Map65	F80	F147	Lockdown	Stress and	0.6
Map65	F80	F120	Lockdown	No transpo	0.8
Map65	F82	F01	Lost invest	Bad experi	0.6
Map65	F82	F64	Lost invest	Increased p	0.6
Map65	F82	F63	Lost invest	Increased c	0.6
Map65	F82	F96	Lost invest	More hunge	0.6
Map65	F07	F82	Businesses	Lost invest	0.8
Map65	F07	F01	Businesses	Bad experi	0.6
Map65	F07	F64	Businesses	Increased p	0.6
Map65	F07	F63	Businesses	Increased c	0.6
Map65	F07	F96	Businesses	More hunge	0.6
Map65	F20	F01	Difficult bre	Bad experi	0.2
Map65	F29	F01	Fear of cor	Bad experi	0.4
Map65	F29	F30	Fear of cor	Fear of dea	0.6
Map65	F29	F31	Fear of cor	Fear of eac	0.6
Map65	F64	F01	Increased p	Bad experi	0.6
Map65	F64	F63	Increased p	Increased c	0.6
Map65	F64	F96	Increased p	More hunge	0.6
Map65	F63	F01	Increased c	Bad experi	0.4
Map65	F62	F01	Increased r	Bad experi	0.6
Map65	F157	F01	Women un	Bad experi	0.2
Map65	F157	F118	Women un	No social g	0.6
Map65	F157	F32	Women un	Feeling soc	0.4

For Peer Review

Map65	F158	F01	Youth/child	Bad experi	0.6
Map65	F158	F13	Youth/child	Could not c	0.6
Map65	F158	F147	Youth/child	Stress and	0.6
Map65	F96	F01	More hung	Bad experi	1
Map65	F96	F63	More hung	Increased c	0.6
Map65	F95	F01	More hous	Bad experi	0.6
Map65	F95	F62	More hous	Increased r	0.6
Map65	F118	F01	No social g	Bad experi	0.2
Map65	F118	F32	No social g	Feeling soc	0.4
Map65	F98	F01	More mate	Bad experi	0.6
Map65	F99	F01	More unwa	Bad experi	0.8
Map65	F99	F98	More unwa	More mate	0.6
Map65	F30	F01	Fear of dea	Bad experi	0.4
Map65	F31	F01	Fear of eac	Bad experi	0.4
Map65	F32	F01	Feeling soc	Bad experi	0.2
Map65	F133	F01	Poor perfor	Bad experi	0.4
Map65	F133	F147	Poor perfor	Stress and	0.4
Map65	F38	F01	Health wor	Bad experi	0.6
Map65	F38	F98	Health wor	More mate	0.6
Map65	F13	F01	Could not c	Bad experi	0.6
Map65	F13	F147	Could not c	Stress and	0.6
Map65	F10	F20	Compulsor	Difficult bre	0.4
Map65	F10	F01	Compulsor	Bad experi	0.2
Map65	F16	F01	Delay in ec	Bad experi	0.4
Map65	F16	F133	Delay in ec	Poor perfor	0.4
Map65	F16	F147	Delay in ec	Stress and	0.4
Map65	F142	F01	School/ma	Bad experi	0.6
Map65	F142	F158	School/ma	Youth/child	0.8
Map65	F142	F133	School/ma	Poor perfor	0.4
Map65	F142	F13	School/ma	Could not c	0.6
Map65	F142	F16	School/ma	Delay in ec	0.6
Map65	F142	F147	School/ma	Stress and	0.6
Map65	F140	F01	Restriction	Bad experi	0.6
Map65	F140	F157	Restriction	Women un	0.8
Map65	F140	F118	Restriction	No social g	0.6
Map65	F140	F98	Restriction	More mate	0.6
Map65	F140	F32	Restriction	Feeling soc	0.4
Map65	F140	F38	Restriction	Health wor	0.8
Map65	F140	F120	Restriction	No transpo	0.8
Map65	F147	F01	Stress and	Bad experi	0.6
Map65	F120	F01	No transpo	Bad experi	0.6
Map65	F120	F157	No transpo	Women un	0.8
Map65	F120	F118	No transpo	No social g	0.6
Map65	F120	F98	No transpo	More mate	0.6
Map65	F120	F32	No transpo	Feeling soc	0.4
Map65	F120	F38	No transpo	Health wor	0.8
Map66	F40	F47	Household	Increase in	0.2
Map66	F40	F01	Household	Bad experi	0.2
Map66	F88	F40	Men unable	Household	0.4
Map66	F88	F47	Men unable	Increase in	0.2
Map66	F88	F01	Men unable	Bad experi	0.8
Map66	F88	F63	Men unable	Increased c	0.6
Map66	F88	F96	Men unable	More hung	0.8
Map66	F47	F01	Increase in	Bad experi	0.4
Map66	F85	F40	Markets/sh	Household	0.4
Map66	F85	F88	Markets/sh	Men unable	0.8

For Peer Review

Map66	F85	F47	Markets/sh	Increase in	0.2
Map66	F85	F87	Markets/sh	Men stayin	0.8
Map66	F85	F82	Markets/sh	Lost invest	0.6
Map66	F85	F07	Markets/sh	Businesses	1
Map66	F85	F01	Markets/sh	Bad experi	0.8
Map66	F85	F64	Markets/sh	Increased	0.6
Map66	F85	F63	Markets/sh	Increased	0.6
Map66	F85	F70	Markets/sh	Jobs lost	1
Map66	F85	F96	Markets/sh	More hung	0.8
Map66	F87	F40	Men stayin	Household	0.4
Map66	F87	F88	Men stayin	Men unable	1
Map66	F87	F47	Men stayin	Increase in	0.2
Map66	F87	F01	Men stayin	Bad experi	0.8
Map66	F87	F63	Men stayin	Increased	0.6
Map66	F87	F96	Men stayin	More hung	0.8
Map66	F80	F40	Lockdown	Household	0.4
Map66	F80	F88	Lockdown	Men unable	0.8
Map66	F80	F47	Lockdown	Increase in	0.2
Map66	F80	F85	Lockdown	Markets/sh	1
Map66	F80	F87	Lockdown	Men stayin	0.8
Map66	F80	F82	Lockdown	Lost invest	0.6
Map66	F80	F07	Lockdown	Businesses	1
Map66	F80	F01	Lockdown	Bad experi	0.8
Map66	F80	F64	Lockdown	Increased	0.6
Map66	F80	F63	Lockdown	Increased	0.8
Map66	F80	F70	Lockdown	Jobs lost	1
Map66	F80	F96	Lockdown	More hung	0.8
Map66	F80	F133	Lockdown	Poor perfor	0.6
Map66	F80	F135	Lockdown	Price hike	1
Map66	F80	F37	Lockdown	Health facil	0.8
Map66	F80	F38	Lockdown	Health wor	0.6
Map66	F80	F13	Lockdown	Could not	0.8
Map66	F80	F16	Lockdown	Delay in ec	1
Map66	F80	F14	Lockdown	Could not	0.8
Map66	F80	F142	Lockdown	School/ma	1
Map66	F80	F140	Lockdown	Restriction	0.8
Map66	F80	F147	Lockdown	Stress and	0.6
Map66	F80	F120	Lockdown	No transpo	0.6
Map66	F80	F144	Lockdown	Social dista	0.6
Map66	F80	F106	Lockdown	No congreg	0.8
Map66	F80	F100	Lockdown	Mosques/c	0.8
Map66	F82	F01	Lost invest	Bad experi	0.8
Map66	F82	F64	Lost invest	Increased	0.8
Map66	F82	F63	Lost invest	Increased	0.6
Map66	F82	F96	Lost invest	More hung	0.8
Map66	F07	F82	Businesses	Lost invest	0.6
Map66	F07	F01	Businesses	Bad experi	0.6
Map66	F07	F64	Businesses	Increased	0.6
Map66	F07	F63	Businesses	Increased	0.6
Map66	F07	F96	Businesses	More hung	0.6
Map66	F20	F01	Difficult bre	Bad experi	0.4
Map66	F29	F01	Fear of cor	Bad experi	0.6
Map66	F29	F30	Fear of cor	Fear of dea	0.8
Map66	F29	F31	Fear of cor	Fear of eac	0.8
Map66	F64	F01	Increased	Bad experi	1
Map66	F64	F63	Increased	Increased	0.6

For Peer Review

Map66	F64	F96	Increased p	More hung	1
Map66	F63	F01	Increased c	Bad experi	0.4
Map66	F70	F40	Jobs lost	Household	0.4
Map66	F70	F88	Jobs lost	Men unable	0.8
Map66	F70	F47	Jobs lost	Increase in	0.2
Map66	F70	F87	Jobs lost	Men stayin	0.8
Map66	F70	F01	Jobs lost	Bad experi	0.8
Map66	F70	F63	Jobs lost	Increased c	0.6
Map66	F70	F96	Jobs lost	More hung	0.8
Map66	F96	F01	More hung	Bad experi	1
Map66	F96	F63	More hung	Increased c	0.6
Map66	F133	F01	Poor perfor	Bad experi	0.6
Map66	F133	F147	Poor perfor	Stress and	0.6
Map66	F135	F01	Price hike	Bad experi	0.8
Map66	F135	F63	Price hike	Increased c	0.6
Map66	F135	F96	Price hike	More hung	0.6
Map66	F30	F01	Fear of dea	Bad experi	0.6
Map66	F31	F01	Fear of eac	Bad experi	0.6
Map66	F37	F01	Health facil	Bad experi	0.4
Map66	F37	F63	Health facil	Increased c	0.8
Map66	F37	F14	Health facil	Could not g	0.8
Map66	F38	F01	Health wor	Bad experi	0.4
Map66	F38	F63	Health wor	Increased c	0.6
Map66	F38	F14	Health wor	Could not g	0.6
Map66	F13	F01	Could not c	Bad experi	0.6
Map66	F13	F147	Could not c	Stress and	0.6
Map66	F10	F20	Compulsor	Difficult bre	0.4
Map66	F10	F01	Compulsor	Bad experi	0.4
Map66	F16	F01	Delay in ec	Bad experi	0.6
Map66	F16	F133	Delay in ec	Poor perfor	0.6
Map66	F16	F147	Delay in ec	Stress and	0.6
Map66	F14	F01	Could not g	Bad experi	0.4
Map66	F14	F63	Could not g	Increased c	0.8
Map66	F142	F01	School/ma	Bad experi	0.6
Map66	F142	F133	School/ma	Poor perfor	0.6
Map66	F142	F13	School/ma	Could not c	0.8
Map66	F142	F16	School/ma	Delay in ec	1
Map66	F142	F147	School/ma	Stress and	0.6
Map66	F140	F40	Restriction	Household	0.4
Map66	F140	F88	Restriction	Men unable	0.4
Map66	F140	F47	Restriction	Increase in	0.2
Map66	F140	F87	Restriction	Men stayin	0.4
Map66	F140	F01	Restriction	Bad experi	0.4
Map66	F140	F63	Restriction	Increased c	0.6
Map66	F140	F96	Restriction	More hung	0.4
Map66	F140	F38	Restriction	Health wor	0.6
Map66	F140	F14	Restriction	Could not g	0.6
Map66	F140	F120	Restriction	No transpo	0.6
Map66	F147	F01	Stress and	Bad experi	1
Map66	F120	F40	No transpo	Household	0.4
Map66	F120	F88	No transpo	Men unable	0.4
Map66	F120	F47	No transpo	Increase in	0.2
Map66	F120	F87	No transpo	Men stayin	0.4
Map66	F120	F01	No transpo	Bad experi	0.4
Map66	F120	F63	No transpo	Increased c	0.6
Map66	F120	F96	No transpo	More hung	0.4

For Peer Review

Map66	F120	F38	No transpo	Health wor	0.6
Map66	F120	F14	No transpo	Could not g	0.6
Map66	F144	F01	Social dista	Bad experi	0.8
Map66	F144	F147	Social dista	Stress and	0.6
Map66	F106	F01	No congreg	Bad experi	0.6
Map66	F106	F147	No congreg	Stress and	0.6
Map66	F106	F144	No congreg	Social dista	0.6
Map66	F100	F01	Mosques/c	Bad experi	0.6
Map66	F100	F147	Mosques/c	Stress and	0.6
Map66	F100	F144	Mosques/c	Social dista	0.6
Map66	F100	F106	Mosques/c	No congreg	0.8
Map67	F40	F47	Household	Increase in	0.6
Map67	F40	F01	Household	Bad experi	0.6
Map67	F40	F147	Household	Stress and	0.6
Map67	F47	F01	Increase in	Bad experi	0.8
Map67	F47	F147	Increase in	Stress and	0.8
Map67	F85	F40	Markets/sh	Household	0.4
Map67	F85	F47	Markets/sh	Increase in	0.4
Map67	F85	F87	Markets/sh	Men stayin	0.6
Map67	F85	F01	Markets/sh	Bad experi	0.6
Map67	F85	F67	Markets/sh	Increased s	0.6
Map67	F85	F64	Markets/sh	Increased p	0.6
Map67	F85	F70	Markets/sh	Jobs lost	0.6
Map67	F85	F96	Markets/sh	More hung	0.6
Map67	F85	F78	Markets/sh	Less food t	0.6
Map67	F85	F72	Markets/sh	Lack of bas	0.6
Map67	F85	F147	Markets/sh	Stress and	0.4
Map67	F87	F40	Men stayin	Household	0.4
Map67	F87	F47	Men stayin	Increase in	0.4
Map67	F87	F01	Men stayin	Bad experi	0.6
Map67	F87	F96	Men stayin	More hung	0.6
Map67	F87	F78	Men stayin	Less food t	0.6
Map67	F87	F72	Men stayin	Lack of bas	0.6
Map67	F87	F147	Men stayin	Stress and	0.4
Map67	F80	F40	Lockdown	Household	0.4
Map67	F80	F47	Lockdown	Increase in	0.4
Map67	F80	F85	Lockdown	Markets/sh	0.8
Map67	F80	F87	Lockdown	Men stayin	0.6
Map67	F80	F01	Lockdown	Bad experi	0.8
Map67	F80	F67	Lockdown	Increased s	0.6
Map67	F80	F64	Lockdown	Increased p	0.6
Map67	F80	F62	Lockdown	Increased r	0.4
Map67	F80	F70	Lockdown	Jobs lost	0.6
Map67	F80	F96	Lockdown	More hung	0.6
Map67	F80	F115	Lockdown	No preachi	0.8
Map67	F80	F118	Lockdown	No social g	0.4
Map67	F80	F78	Lockdown	Less food t	0.6
Map67	F80	F76	Lockdown	Less affect	0.4
Map67	F80	F16	Lockdown	Delay in ec	1
Map67	F80	F72	Lockdown	Lack of bas	0.6
Map67	F80	F142	Lockdown	School/ma	1
Map67	F80	F147	Lockdown	Stress and	0.8
Map67	F80	F123	Lockdown	Not going t	0.6
Map67	F80	F144	Lockdown	Social dista	0.8
Map67	F80	F106	Lockdown	No congreg	0.8
Map67	F80	F100	Lockdown	Mosques/c	0.8

For Peer Review

Map67	F67	F01	Increased s	Bad experi	0.4
Map67	F29	F01	Fear of cor	Bad experi	0.6
Map67	F29	F62	Fear of cor	Increased r	0.6
Map67	F29	F31	Fear of cor	Fear of eac	0.6
Map67	F29	F118	Fear of cor	No social g	0.6
Map67	F29	F76	Fear of cor	Less affect	0.6
Map67	F64	F01	Increased p	Bad experi	0.6
Map67	F64	F67	Increased p	Increased s	0.8
Map67	F64	F96	Increased p	More hung	0.6
Map67	F64	F78	Increased p	Less food t	0.6
Map67	F62	F01	Increased r	Bad experi	0.6
Map67	F70	F40	Jobs lost	Household	0.4
Map67	F70	F47	Jobs lost	Increase in	0.4
Map67	F70	F87	Jobs lost	Men stayin	0.8
Map67	F70	F01	Jobs lost	Bad experi	0.6
Map67	F70	F67	Jobs lost	Increased s	0.8
Map67	F70	F64	Jobs lost	Increased p	0.8
Map67	F70	F96	Jobs lost	More hung	0.6
Map67	F70	F78	Jobs lost	Less food t	0.6
Map67	F70	F72	Jobs lost	Lack of bas	0.6
Map67	F70	F147	Jobs lost	Stress and	0.4
Map67	F96	F01	More hung	Bad experi	1
Map67	F115	F01	No preachi	Bad experi	0.8
Map67	F115	F147	No preachi	Stress and	0.8
Map67	F31	F01	Fear of eac	Bad experi	0.6
Map67	F31	F62	Fear of eac	Increased r	0.6
Map67	F31	F118	Fear of eac	No social g	0.6
Map67	F31	F76	Fear of eac	Less affect	0.6
Map67	F118	F01	No social g	Bad experi	0.6
Map67	F118	F62	No social g	Increased r	0.6
Map67	F118	F76	No social g	Less affect	0.6
Map67	F78	F01	Less food t	Bad experi	0.6
Map67	F78	F96	Less food t	More hung	0.6
Map67	F76	F01	Less affect	Bad experi	0.6
Map67	F76	F62	Less affect	Increased r	0.8
Map67	F16	F01	Delay in ec	Bad experi	0.8
Map67	F72	F40	Lack of bas	Household	0.4
Map67	F72	F47	Lack of bas	Increase in	0.4
Map67	F72	F01	Lack of bas	Bad experi	0.6
Map67	F72	F96	Lack of bas	More hung	0.6
Map67	F72	F78	Lack of bas	Less food t	0.8
Map67	F72	F147	Lack of bas	Stress and	0.4
Map67	F142	F01	School/ma	Bad experi	0.8
Map67	F142	F16	School/ma	Delay in ec	1
Map67	F147	F01	Stress and	Bad experi	1
Map67	F123	F01	Not going t	Bad experi	0.6
Map67	F123	F67	Not going t	Increased s	0.6
Map67	F123	F64	Not going t	Increased p	0.6
Map67	F123	F96	Not going t	More hung	0.6
Map67	F123	F78	Not going t	Less food t	0.6
Map67	F144	F01	Social dista	Bad experi	0.8
Map67	F144	F62	Social dista	Increased r	0.4
Map67	F144	F115	Social dista	No preachi	0.8
Map67	F144	F118	Social dista	No social g	0.4
Map67	F144	F76	Social dista	Less affect	0.4
Map67	F144	F147	Social dista	Stress and	0.8

For Peer Review

Map67	F144	F106	Social dista	No congreg	1
Map67	F144	F100	Social dista	Mosques/c	1
Map67	F106	F01	No congreg	Bad experi	0.8
Map67	F100	F01	Mosques/c	Bad experi	0.8
Map67	F100	F115	Mosques/c	No preachi	0.8
Map67	F100	F147	Mosques/c	Stress and	0.8
Map67	F100	F106	Mosques/c	No congreg	1
Map68	F85	F83	Markets/sh	Malnutritior	0.4
Map68	F85	F82	Markets/sh	Lost invest	0.6
Map68	F85	F07	Markets/sh	Businesses	0.6
Map68	F85	F01	Markets/sh	Bad experi	0.6
Map68	F85	F64	Markets/sh	Increased p	0.6
Map68	F85	F63	Markets/sh	Increased (	0.4
Map68	F85	F96	Markets/sh	More hung	0.4
Map68	F85	F135	Markets/sh	Price hike	0.4
Map68	F85	F78	Markets/sh	Less food t	0.4
Map68	F85	F70	Markets/sh	Jobs lost	0.6
Map68	F85	F143	Markets/sh	Shortage o	0.6
Map68	F80	F85	Lockdown	Markets/sh	0.8
Map68	F80	F83	Lockdown	Malnutritior	0.6
Map68	F80	F82	Lockdown	Lost invest	0.6
Map68	F80	F07	Lockdown	Businesses	0.6
Map68	F80	F01	Lockdown	Bad experi	0.6
Map68	F80	F64	Lockdown	Increased p	0.6
Map68	F80	F63	Lockdown	Increased (	0.6
Map68	F80	F16	Lockdown	Delay in ec	0.6
Map68	F80	F118	Lockdown	No social g	0.4
Map68	F80	F96	Lockdown	More hung	0.6
Map68	F80	F135	Lockdown	Price hike	0.8
Map68	F80	F142	Lockdown	School/mad	0.6
Map68	F80	F78	Lockdown	Less food t	0.6
Map68	F80	F70	Lockdown	Jobs lost	0.6
Map68	F80	F143	Lockdown	Shortage o	0.6
Map68	F80	F124	Lockdown	Not visiting	0.4
Map68	F80	F140	Lockdown	Restriction	1
Map68	F80	F147	Lockdown	Stress and	0.6
Map68	F80	F120	Lockdown	No transpo	0.6
Map68	F80	F144	Lockdown	Social dista	0.6
Map68	F80	F106	Lockdown	No congreg	0.6
Map68	F80	F100	Lockdown	Mosques/c	0.6
Map68	F83	F01	Malnutritior	Bad experi	0.8
Map68	F83	F63	Malnutritior	Increased (	0.8
Map68	F82	F01	Lost invest	Bad experi	0.6
Map68	F82	F64	Lost invest	Increased p	0.6
Map68	F07	F82	Businesses	Lost invest	0.6
Map68	F07	F01	Businesses	Bad experi	0.6
Map68	F07	F64	Businesses	Increased p	0.6
Map68	F07	F70	Businesses	Jobs lost	0.8
Map68	F20	F01	Difficult bre	Bad experi	0.4
Map68	F29	F82	Fear of cor	Lost invest	0.4
Map68	F29	F07	Fear of cor	Businesses	0.4
Map68	F29	F01	Fear of cor	Bad experi	0.4
Map68	F29	F64	Fear of cor	Increased p	0.4
Map68	F29	F31	Fear of cor	Fear of eac	0.4
Map68	F29	F70	Fear of cor	Jobs lost	0.4
Map68	F64	F01	Increased p	Bad experi	0.6

For Peer Review

Map68	F63	F01	Increased (	Bad experi	1
Map68	F16	F01	Delay in ec	Bad experi	0.8
Map68	F118	F01	No social g	Bad experi	0.4
Map68	F118	F147	No social g	Stress and	0.4
Map68	F96	F83	More hung	Malnutritior	0.8
Map68	F96	F01	More hung	Bad experi	0.8
Map68	F96	F63	More hung	Increased (	0.8
Map68	F135	F83	Price hike	Malnutritior	0.6
Map68	F135	F01	Price hike	Bad experi	0.6
Map68	F135	F63	Price hike	Increased (	0.6
Map68	F135	F96	Price hike	More hung	0.6
Map68	F135	F135	Price hike	Price hike	0.4
Map68	F135	F78	Price hike	Less food t	0.6
Map68	F135	F143	Price hike	Shortage o	0.6
Map68	F142	F01	School/ma	Bad experi	0.6
Map68	F142	F16	School/ma	Delay in ec	0.6
Map68	F31	F82	Fear of eac	Lost invest	0.6
Map68	F31	F07	Fear of eac	Businesses	0.6
Map68	F31	F01	Fear of eac	Bad experi	0.6
Map68	F31	F64	Fear of eac	Increased p	0.6
Map68	F31	F70	Fear of eac	Jobs lost	0.6
Map68	F78	F83	Less food t	Malnutritior	0.8
Map68	F78	F01	Less food t	Bad experi	0.8
Map68	F78	F63	Less food t	Increased (	0.8
Map68	F78	F96	Less food t	More hung	0.8
Map68	F10	F20	Compulsor	Difficult bre	0.2
Map68	F10	F01	Compulsor	Bad experi	0.2
Map68	F70	F82	Jobs lost	Lost invest	0.6
Map68	F70	F01	Jobs lost	Bad experi	0.6
Map68	F70	F64	Jobs lost	Increased p	0.6
Map68	F143	F83	Shortage o	Malnutritior	0.4
Map68	F143	F01	Shortage o	Bad experi	0.4
Map68	F143	F63	Shortage o	Increased (	0.4
Map68	F143	F96	Shortage o	More hung	0.4
Map68	F143	F135	Shortage o	Price hike	0.4
Map68	F143	F78	Shortage o	Less food t	0.4
Map68	F143	F143	Shortage o	Shortage o	0.4
Map68	F124	F01	Not visiting	Bad experi	0.6
Map68	F124	F63	Not visiting	Increased (	0.6
Map68	F140	F01	Restriction	Bad experi	0.4
Map68	F140	F63	Restriction	Increased (	0.4
Map68	F140	F124	Restriction	Not visiting	0.4
Map68	F140	F120	Restriction	No transpo	0.6
Map68	F147	F01	Stress and	Bad experi	0.8
Map68	F120	F01	No transpo	Bad experi	0.4
Map68	F120	F63	No transpo	Increased (	0.4
Map68	F120	F124	No transpo	Not visiting	0.4
Map68	F144	F01	Social dista	Bad experi	0.8
Map68	F144	F118	Social dista	No social g	0.4
Map68	F144	F147	Social dista	Stress and	0.8
Map68	F144	F106	Social dista	No congreg	0.8
Map68	F144	F100	Social dista	Mosques/c	0.8
Map68	F106	F01	No congreg	Bad experi	0.8
Map68	F106	F147	No congreg	Stress and	0.8
Map68	F100	F01	Mosques/c	Bad experi	0.8
Map68	F100	F147	Mosques/c	Stress and	0.8

For Peer Review

Map68	F100	F106	Mosques/c	No congreg	0.8
Map69	F88	F01	Men unable	Bad experi	0.4
Map69	F88	F62	Men unable	Increased r	0.4
Map69	F88	F95	Men unable	More hous	0.4
Map69	F85	F07	Markets/sh	Businesses	0.8
Map69	F85	F01	Markets/sh	Bad experi	0.8
Map69	F85	F64	Markets/sh	Increased p	0.8
Map69	F85	F96	Markets/sh	More hung	0.6
Map69	F85	F78	Markets/sh	Less food t	0.6
Map69	F86	F01	Men couldn	Bad experi	0.6
Map69	F86	F147	Men couldn	Stress and	0.6
Map69	F80	F88	Lockdown	Men unable	0.6
Map69	F80	F85	Lockdown	Markets/sh	0.8
Map69	F80	F86	Lockdown	Men couldn	0.8
Map69	F80	F07	Lockdown	Businesses	0.8
Map69	F80	F01	Lockdown	Bad experi	0.8
Map69	F80	F64	Lockdown	Increased p	0.8
Map69	F80	F63	Lockdown	Increased c	0.6
Map69	F80	F62	Lockdown	Increased r	0.8
Map69	F80	F158	Lockdown	Youth/child	0.8
Map69	F80	F09	Lockdown	Cannot acc	0.8
Map69	F80	F96	Lockdown	More hung	0.6
Map69	F80	F95	Lockdown	More hous	0.8
Map69	F80	F133	Lockdown	Poor perfor	0.8
Map69	F80	F142	Lockdown	School/ma	0.8
Map69	F80	F118	Lockdown	No social g	0.2
Map69	F80	F78	Lockdown	Less food t	0.6
Map69	F80	F13	Lockdown	Could not c	0.8
Map69	F80	F70	Lockdown	Jobs lost	0.8
Map69	F80	F124	Lockdown	Not visiting	0.6
Map69	F80	F147	Lockdown	Stress and	0.6
Map69	F80	F120	Lockdown	No transpo	0.8
Map69	F80	F149	Lockdown	Teachers a	0.8
Map69	F07	F01	Businesses	Bad experi	0.8
Map69	F07	F64	Businesses	Increased p	0.8
Map69	F29	F01	Fear of cor	Bad experi	0.6
Map69	F29	F63	Fear of cor	Increased c	0.4
Map69	F29	F31	Fear of cor	Fear of eac	0.6
Map69	F29	F124	Fear of cor	Not visiting	0.4
Map69	F64	F01	Increased p	Bad experi	1
Map69	F63	F01	Increased c	Bad experi	0.6
Map69	F62	F01	Increased r	Bad experi	0.8
Map69	F158	F01	Youth/child	Bad experi	0.6
Map69	F158	F13	Youth/child	Could not c	1
Map69	F158	F147	Youth/child	Stress and	0.6
Map69	F09	F01	Cannot acc	Bad experi	0.8
Map69	F09	F62	Cannot acc	Increased r	0.8
Map69	F09	F95	Cannot acc	More hous	0.8
Map69	F96	F01	More hung	Bad experi	1
Map69	F95	F01	More hous	Bad experi	0.6
Map69	F95	F62	More hous	Increased r	0.6
Map69	F133	F01	Poor perfor	Bad experi	0.8
Map69	F142	F01	School/ma	Bad experi	0.8
Map69	F142	F158	School/ma	Youth/child	0.8
Map69	F142	F133	School/ma	Poor perfor	1
Map69	F142	F13	School/ma	Could not c	0.8

For Peer Review

Map69	F142	F147	School/ma	Stress and	0.6
Map69	F31	F01	Fear of eac	Bad experi	0.8
Map69	F118	F01	No social g	Bad experi	0.2
Map69	F118	F147	No social g	Stress and	0.2
Map69	F78	F01	Less food t	Bad experi	0.8
Map69	F78	F96	Less food t	More hung	0.8
Map69	F12	F01	Constant m	Bad experi	0.4
Map69	F12	F29	Constant m	Fear of cor	0.4
Map69	F12	F63	Constant m	Increased (	0.4
Map69	F12	F31	Constant m	Fear of eac	0.4
Map69	F12	F124	Constant m	Not visiting	0.4
Map69	F13	F01	Could not c	Bad experi	0.6
Map69	F13	F147	Could not c	Stress and	0.6
Map69	F70	F88	Jobs lost	Men unable	0.6
Map69	F70	F01	Jobs lost	Bad experi	0.6
Map69	F70	F64	Jobs lost	Increased p	0.6
Map69	F70	F62	Jobs lost	Increased r	0.4
Map69	F70	F95	Jobs lost	More house	0.4
Map69	F124	F01	Not visiting	Bad experi	0.6
Map69	F124	F63	Not visiting	Increased (	0.6
Map69	F147	F01	Stress and	Bad experi	0.8
Map69	F120	F86	No transpo	Men couldr	1
Map69	F120	F01	No transpo	Bad experi	0.8
Map69	F120	F63	No transpo	Increased (	0.6
Map69	F120	F158	No transpo	Youth/child	0.8
Map69	F120	F133	No transpo	Poor perfor	0.8
Map69	F120	F142	No transpo	School/ma	0.8
Map69	F120	F13	No transpo	Could not c	0.8
Map69	F120	F124	No transpo	Not visiting	0.6
Map69	F120	F147	No transpo	Stress and	0.6
Map69	F120	F149	No transpo	Teachers a	0.8
Map69	F149	F01	Teachers a	Bad experi	0.8
Map69	F149	F158	Teachers a	Youth/child	0.8
Map69	F149	F133	Teachers a	Poor perfor	0.8
Map69	F149	F142	Teachers a	School/ma	0.8
Map69	F149	F13	Teachers a	Could not c	0.8
Map69	F149	F147	Teachers a	Stress and	0.6
Map70	F85	F83	Markets/sh	Malnutritior	0.6
Map70	F85	F07	Markets/sh	Businesses	0.8
Map70	F85	F01	Markets/sh	Bad experi	0.8
Map70	F85	F64	Markets/sh	Increased p	0.8
Map70	F85	F63	Markets/sh	Increased (	0.6
Map70	F85	F96	Markets/sh	More hung	0.8
Map70	F85	F135	Markets/sh	Price hike	0.8
Map70	F85	F78	Markets/sh	Less food t	0.8
Map70	F85	F147	Markets/sh	Stress and	0.4
Map70	F85	F104	Markets/sh	No buyers	0.6
Map70	F80	F85	Lockdown	Markets/sh	0.8
Map70	F80	F83	Lockdown	Malnutritior	0.6
Map70	F80	F07	Lockdown	Businesses	0.8
Map70	F80	F01	Lockdown	Bad experi	0.8
Map70	F80	F64	Lockdown	Increased p	0.8
Map70	F80	F63	Lockdown	Increased (	0.6
Map70	F80	F96	Lockdown	More hung	0.8
Map70	F80	F112	Lockdown	No home/p	0.6
Map70	F80	F135	Lockdown	Price hike	0.8

For Peer Review

Map70	F80	F142	Lockdown	School/ma	0.8
Map70	F80	F78	Lockdown	Less food t	0.8
Map70	F80	F147	Lockdown	Stress and	0.6
Map70	F80	F16	Lockdown	Delay in ec	0.6
Map70	F80	F140	Lockdown	Restriction	0.6
Map70	F80	F149	Lockdown	Teachers a	0.8
Map70	F80	F104	Lockdown	No buyers	0.6
Map70	F83	F01	Malnutritior	Bad experi	0.6
Map70	F83	F63	Malnutritior	Increased	0.6
Map70	F07	F83	Businesses	Malnutritior	0.6
Map70	F07	F01	Businesses	Bad experi	0.8
Map70	F07	F64	Businesses	Increased	0.8
Map70	F07	F63	Businesses	Increased	0.6
Map70	F07	F96	Businesses	More hung	0.8
Map70	F07	F135	Businesses	Price hike	1
Map70	F07	F78	Businesses	Less food t	0.8
Map70	F07	F147	Businesses	Stress and	0.4
Map70	F07	F104	Businesses	No buyers	0.6
Map70	F20	F01	Difficult bre	Bad experi	0.6
Map70	F20	F147	Difficult bre	Stress and	0.6
Map70	F29	F20	Fear of cor	Difficult bre	0.6
Map70	F29	F01	Fear of cor	Bad experi	0.8
Map70	F29	F63	Fear of cor	Increased	0.8
Map70	F29	F31	Fear of cor	Fear of eac	0.6
Map70	F29	F38	Fear of cor	Health wor	0.8
Map70	F29	F147	Fear of cor	Stress and	0.6
Map70	F29	F10	Fear of cor	Compulsor	0.6
Map70	F29	F124	Fear of cor	Not visiting	0.8
Map70	F64	F83	Increased	Malnutritior	0.6
Map70	F64	F01	Increased	Bad experi	1
Map70	F64	F63	Increased	Increased	0.6
Map70	F64	F96	Increased	More hung	0.8
Map70	F64	F78	Increased	Less food t	1
Map70	F64	F147	Increased	Stress and	0.4
Map70	F64	F104	Increased	No buyers	0.6
Map70	F63	F01	Increased	Bad experi	0.8
Map70	F62	F01	Increased	Bad experi	0.6
Map70	F118	F01	No social g	Bad experi	0.2
Map70	F118	F62	No social g	Increased	0.2
Map70	F118	F121	No social g	No wedding	0.2
Map70	F96	F83	More hung	Malnutritior	0.6
Map70	F96	F01	More hung	Bad experi	0.8
Map70	F96	F63	More hung	Increased	0.6
Map70	F112	F01	No home/p	Bad experi	0.4
Map70	F112	F147	No home/p	Stress and	0.4
Map70	F112	F104	No home/p	No buyers	0.4
Map70	F135	F83	Price hike	Malnutritior	0.6
Map70	F135	F01	Price hike	Bad experi	0.8
Map70	F135	F64	Price hike	Increased	0.8
Map70	F135	F63	Price hike	Increased	0.6
Map70	F135	F96	Price hike	More hung	0.8
Map70	F135	F78	Price hike	Less food t	0.8
Map70	F135	F147	Price hike	Stress and	0.4
Map70	F135	F104	Price hike	No buyers	0.6
Map70	F142	F01	School/ma	Bad experi	0.6
Map70	F142	F147	School/ma	Stress and	0.6

For Peer Review

Map70	F142	F16	School/mac	Delay in ec	0.6
Map70	F142	F149	School/mac	Teachers a	1
Map70	F31	F20	Fear of eac	Difficult bre	0.6
Map70	F31	F01	Fear of eac	Bad experi	0.6
Map70	F31	F147	Fear of eac	Stress and	0.6
Map70	F31	F10	Fear of eac	Compulsor	0.6
Map70	F78	F83	Less food t	Malnutritior	0.6
Map70	F78	F01	Less food t	Bad experi	0.8
Map70	F78	F63	Less food t	Increased c	0.6
Map70	F78	F96	Less food t	More hung	0.8
Map70	F38	F01	Health wor	Bad experi	0.6
Map70	F38	F63	Health wor	Increased c	0.6
Map70	F147	F01	Stress and	Bad experi	0.6
Map70	F10	F20	Compulsor	Difficult bre	0.6
Map70	F10	F01	Compulsor	Bad experi	0.6
Map70	F10	F147	Compulsor	Stress and	0.6
Map70	F16	F01	Delay in ec	Bad experi	0.6
Map70	F16	F147	Delay in ec	Stress and	0.8
Map70	F124	F01	Not visiting	Bad experi	0.8
Map70	F124	F63	Not visiting	Increased c	0.8
Map70	F140	F01	Restriction	Bad experi	0.4
Map70	F140	F112	Restriction	No home/p	0.6
Map70	F140	F147	Restriction	Stress and	0.4
Map70	F140	F104	Restriction	No buyers	0.4
Map70	F121	F01	No wedding	Bad experi	0.4
Map70	F121	F62	No wedding	Increased r	0.4
Map70	F144	F01	Social dista	Bad experi	0.2
Map70	F144	F62	Social dista	Increased r	0.2
Map70	F144	F118	Social dista	No social g	0.6
Map70	F144	F121	Social dista	No wedding	0.2
Map70	F149	F01	Teachers a	Bad experi	0.6
Map70	F149	F147	Teachers a	Stress and	0.6
Map70	F149	F16	Teachers a	Delay in ec	0.6
Map70	F104	F01	No buyers	Bad experi	0.4
Map70	F104	F147	No buyers	Stress and	0.4
Map71	F40	F47	Household	Increase in	0.8
Map71	F40	F01	Household	Bad experi	0.6
Map71	F47	F01	Increase in	Bad experi	0.6
Map71	F80	F40	Lockdown	Household	0.6
Map71	F80	F47	Lockdown	Increase in	0.6
Map71	F80	F82	Lockdown	Lost invest	0.6
Map71	F80	F07	Lockdown	Businesses	0.6
Map71	F80	F06	Lockdown	Borders clo	0.8
Map71	F80	F01	Lockdown	Bad experi	0.6
Map71	F80	F64	Lockdown	Increased p	0.6
Map71	F80	F158	Lockdown	Youth/child	1
Map71	F80	F16	Lockdown	Delay in ec	0.8
Map71	F80	F96	Lockdown	More hung	0.6
Map71	F80	F114	Lockdown	No pilgrima	0.8
Map71	F80	F135	Lockdown	Price hike	0.6
Map71	F80	F78	Lockdown	Less food t	0.6
Map71	F80	F70	Lockdown	Jobs lost	0.8
Map71	F80	F72	Lockdown	Lack of bas	0.6
Map71	F80	F142	Lockdown	School/mac	1
Map71	F80	F140	Lockdown	Restriction	0.8
Map71	F80	F147	Lockdown	Stress and	0.6

For Peer Review

Map71	F80	F123	Lockdown	Not going t	0.8
Map71	F80	F106	Lockdown	No congreg	0.6
Map71	F80	F113	Lockdown	No nomadi	0.6
Map71	F80	F100	Lockdown	Mosques/c	0.6
Map71	F82	F01	Lost invest	Bad experi	0.6
Map71	F82	F64	Lost invest	Increased p	0.6
Map71	F07	F40	Businesses	Household	0.6
Map71	F07	F47	Businesses	Increase in	0.6
Map71	F07	F82	Businesses	Lost invest	0.8
Map71	F07	F01	Businesses	Bad experi	0.6
Map71	F07	F64	Businesses	Increased p	0.6
Map71	F07	F135	Businesses	Price hike	0.8
Map71	F07	F72	Businesses	Lack of bas	0.8
Map71	F06	F06	Borders clo	Borders clo	0.6
Map71	F06	F01	Borders clo	Bad experi	0.4
Map71	F06	F114	Borders clo	No pilgrima	0.8
Map71	F06	F147	Borders clo	Stress and	0.4
Map71	F29	F40	Fear of cor	Household	0.4
Map71	F29	F47	Fear of cor	Increase in	0.4
Map71	F29	F01	Fear of cor	Bad experi	0.6
Map71	F29	F63	Fear of cor	Increased c	0.4
Map71	F29	F152	Fear of cor	Unable to b	0.4
Map71	F29	F158	Fear of cor	Youth/child	0.6
Map71	F29	F16	Fear of cor	Delay in ec	0.6
Map71	F29	F118	Fear of cor	No social g	1
Map71	F29	F110	Fear of cor	No funerals	0.4
Map71	F29	F124	Fear of cor	Not visiting	0.4
Map71	F29	F115	Fear of cor	No preachi	0.8
Map71	F29	F38	Fear of cor	Health wor	0.4
Map71	F29	F70	Fear of cor	Jobs lost	0.6
Map71	F29	F72	Fear of cor	Lack of bas	0.4
Map71	F29	F142	Fear of cor	School/mad	0.6
Map71	F29	F147	Fear of cor	Stress and	0.6
Map71	F29	F109	Fear of cor	No financia	0.4
Map71	F29	F103	Fear of cor	No access	0.6
Map71	F64	F01	Increased p	Bad experi	1
Map71	F63	F01	Increased c	Bad experi	0.8
Map71	F152	F01	Unable to b	Bad experi	0.6
Map71	F152	F63	Unable to b	Increased c	0.6
Map71	F158	F40	Youth/child	Household	0.4
Map71	F158	F47	Youth/child	Increase in	0.4
Map71	F158	F01	Youth/child	Bad experi	0.4
Map71	F158	F72	Youth/child	Lack of bas	0.4
Map71	F16	F01	Delay in ec	Bad experi	0.6
Map71	F96	F01	More hung	Bad experi	1
Map71	F114	F01	No pilgrima	Bad experi	0.4
Map71	F114	F147	No pilgrima	Stress and	0.4
Map71	F118	F01	No social g	Bad experi	0.6
Map71	F118	F110	No social g	No funerals	0.4
Map71	F118	F115	No social g	No preachi	0.8
Map71	F118	F147	No social g	Stress and	0.6
Map71	F118	F103	No social g	No access	0.6
Map71	F110	F01	No funerals	Bad experi	0.2
Map71	F135	F40	Price hike	Household	0.6
Map71	F135	F47	Price hike	Increase in	0.6
Map71	F135	F01	Price hike	Bad experi	0.6

Map71	F135	F72	Price hike	Lack of bas	1
Map71	F124	F01	Not visiting	Bad experi	0.6
Map71	F124	F63	Not visiting	Increased (	0.6
Map71	F124	F152	Not visiting	Unable to b	0.8
Map71	F78	F01	Less food t	Bad experi	0.8
Map71	F78	F96	Less food t	More hung	0.8
Map71	F115	F01	No preachi	Bad experi	0.6
Map71	F38	F01	Health wor	Bad experi	0.6
Map71	F38	F63	Health wor	Increased (	0.6
Map71	F38	F152	Health wor	Unable to b	0.8
Map71	F38	F124	Health wor	Not visiting	1
Map71	F70	F40	Jobs lost	Household	0.4
Map71	F70	F47	Jobs lost	Increase in	0.4
Map71	F70	F01	Jobs lost	Bad experi	0.6
Map71	F70	F158	Jobs lost	Youth/child	0.8
Map71	F70	F16	Jobs lost	Delay in ec	0.8
Map71	F70	F72	Jobs lost	Lack of bas	0.4
Map71	F70	F142	Jobs lost	School/ma	0.8
Map71	F72	F40	Lack of bas	Household	0.6
Map71	F72	F47	Lack of bas	Increase in	0.6
Map71	F72	F01	Lack of bas	Bad experi	0.6
Map71	F142	F40	School/ma	Household	0.4
Map71	F142	F47	School/ma	Increase in	0.4
Map71	F142	F01	School/ma	Bad experi	0.6
Map71	F142	F158	School/ma	Youth/child	1
Map71	F142	F16	School/ma	Delay in ec	0.8
Map71	F142	F72	School/ma	Lack of bas	0.4
Map71	F140	F40	Restriction	Household	0.6
Map71	F140	F47	Restriction	Increase in	0.6
Map71	F140	F82	Restriction	Lost invest	0.6
Map71	F140	F07	Restriction	Businesses	0.6
Map71	F140	F01	Restriction	Bad experi	0.6
Map71	F140	F64	Restriction	Increased p	0.6
Map71	F140	F158	Restriction	Youth/child	0.8
Map71	F140	F16	Restriction	Delay in ec	0.8
Map71	F140	F96	Restriction	More hung	0.6
Map71	F140	F135	Restriction	Price hike	0.6
Map71	F140	F78	Restriction	Less food t	0.6
Map71	F140	F70	Restriction	Jobs lost	0.8
Map71	F140	F72	Restriction	Lack of bas	0.6
Map71	F140	F142	Restriction	School/ma	0.8
Map71	F140	F123	Restriction	Not going t	0.8
Map71	F140	F113	Restriction	No nomadi	0.6
Map71	F147	F01	Stress and	Bad experi	0.8
Map71	F123	F01	Not going t	Bad experi	0.6
Map71	F123	F96	Not going t	More hung	0.6
Map71	F123	F78	Not going t	Less food t	0.6
Map71	F109	F01	No financia	Bad experi	0.2
Map71	F109	F63	No financia	Increased (	0.2
Map71	F109	F152	No financia	Unable to b	0.2
Map71	F106	F01	No congreg	Bad experi	0.8
Map71	F106	F147	No congreg	Stress and	1
Map71	F113	F01	No nomadi	Bad experi	0.8
Map71	F113	F96	No nomadi	More hung	0.8
Map71	F113	F78	No nomadi	Less food t	1
Map71	F103	F01	No access	Bad experi	0.6

For Peer Review

Map71	F103	F147	No access	Stress and	0.6
Map71	F100	F01	Mosques/c	Bad experi	0.8
Map71	F100	F147	Mosques/c	Stress and	1
Map71	F100	F106	Mosques/c	No congreg	1
Map72	F85	F83	Markets/sh	Malnutrition	0.6
Map72	F85	F07	Markets/sh	Businesses	0.6
Map72	F85	F01	Markets/sh	Bad experi	0.6
Map72	F85	F64	Markets/sh	Increased p	0.6
Map72	F85	F63	Markets/sh	Increased c	0.6
Map72	F85	F152	Markets/sh	Unable to b	0.6
Map72	F85	F96	Markets/sh	More hung	0.6
Map72	F85	F147	Markets/sh	Stress and	0.4
Map72	F80	F85	Lockdown	Markets/sh	0.8
Map72	F80	F83	Lockdown	Malnutrition	0.6
Map72	F80	F07	Lockdown	Businesses	0.6
Map72	F80	F01	Lockdown	Bad experi	0.6
Map72	F80	F64	Lockdown	Increased p	0.6
Map72	F80	F63	Lockdown	Increased c	0.6
Map72	F80	F62	Lockdown	Increased r	0.6
Map72	F80	F152	Lockdown	Unable to b	0.6
Map72	F80	F118	Lockdown	No social g	0.6
Map72	F80	F96	Lockdown	More hung	0.6
Map72	F80	F110	Lockdown	No funerals	0.6
Map72	F80	F112	Lockdown	No home/p	0.4
Map72	F80	F147	Lockdown	Stress and	0.6
Map72	F80	F70	Lockdown	Jobs lost	0.6
Map72	F80	F143	Lockdown	Shortage o	0.6
Map72	F80	F140	Lockdown	Restriction	0.8
Map72	F80	F121	Lockdown	No wedding	0.6
Map72	F80	F123	Lockdown	Not going t	0.6
Map72	F80	F106	Lockdown	No congreg	0.6
Map72	F80	F104	Lockdown	No buyers	0.4
Map72	F80	F100	Lockdown	Mosques/c	0.6
Map72	F83	F01	Malnutrition	Bad experi	0.6
Map72	F83	F63	Malnutrition	Increased c	0.6
Map72	F07	F83	Businesses	Malnutrition	0.6
Map72	F07	F01	Businesses	Bad experi	0.8
Map72	F07	F64	Businesses	Increased p	0.8
Map72	F07	F63	Businesses	Increased c	0.8
Map72	F07	F152	Businesses	Unable to b	0.8
Map72	F07	F96	Businesses	More hung	0.8
Map72	F07	F147	Businesses	Stress and	0.4
Map72	F29	F83	Fear of cor	Malnutrition	0.2
Map72	F29	F01	Fear of cor	Bad experi	0.6
Map72	F29	F64	Fear of cor	Increased p	0.2
Map72	F29	F63	Fear of cor	Increased c	0.2
Map72	F29	F152	Fear of cor	Unable to b	0.2
Map72	F29	F96	Fear of cor	More hung	0.2
Map72	F29	F31	Fear of cor	Fear of eac	0.8
Map72	F29	F74	Fear of cor	Lack of fac	0.2
Map72	F29	F147	Fear of cor	Stress and	0.6
Map72	F29	F10	Fear of cor	Compulsor	0.6
Map72	F29	F144	Fear of cor	Social dista	0.6
Map72	F29	F106	Fear of cor	No congreg	0.6
Map72	F64	F83	Increased p	Malnutrition	0.6
Map72	F64	F01	Increased p	Bad experi	0.8

For Peer Review

Map72	F64	F64	Increased p	Increased p	0.2
Map72	F64	F63	Increased p	Increased c	0.8
Map72	F64	F152	Increased p	Unable to b	0.8
Map72	F64	F96	Increased p	More hung	1
Map72	F64	F147	Increased p	Stress and	0.4
Map72	F63	F01	Increased c	Bad experi	1
Map72	F62	F01	Increased r	Bad experi	0.8
Map72	F152	F01	Unable to b	Bad experi	1
Map72	F152	F63	Unable to b	Increased c	1
Map72	F118	F01	No social g	Bad experi	0.6
Map72	F118	F62	No social g	Increased r	0.6
Map72	F118	F110	No social g	No funerals	0.6
Map72	F118	F121	No social g	No wedding	0.8
Map72	F96	F83	More hung	Malnutrition	0.6
Map72	F96	F01	More hung	Bad experi	0.6
Map72	F96	F63	More hung	Increased c	0.6
Map72	F110	F01	No funerals	Bad experi	0.4
Map72	F110	F62	No funerals	Increased r	0.4
Map72	F112	F83	No home/p	Malnutrition	0.2
Map72	F112	F01	No home/p	Bad experi	0.2
Map72	F112	F64	No home/p	Increased p	0.2
Map72	F112	F63	No home/p	Increased c	0.2
Map72	F112	F152	No home/p	Unable to b	0.2
Map72	F112	F96	No home/p	More hung	0.2
Map72	F112	F147	No home/p	Stress and	0.2
Map72	F112	F104	No home/p	No buyers	0.4
Map72	F31	F83	Fear of eac	Malnutrition	0.2
Map72	F31	F01	Fear of eac	Bad experi	0.2
Map72	F31	F64	Fear of eac	Increased p	0.2
Map72	F31	F63	Fear of eac	Increased c	0.2
Map72	F31	F152	Fear of eac	Unable to b	0.2
Map72	F31	F96	Fear of eac	More hung	0.2
Map72	F31	F74	Fear of eac	Lack of fac	0.2
Map72	F31	F147	Fear of eac	Stress and	0.2
Map72	F31	F10	Fear of eac	Compulsor	0.6
Map72	F74	F83	Lack of fac	Malnutrition	0.4
Map72	F74	F01	Lack of fac	Bad experi	0.4
Map72	F74	F64	Lack of fac	Increased p	0.4
Map72	F74	F63	Lack of fac	Increased c	0.4
Map72	F74	F152	Lack of fac	Unable to b	0.4
Map72	F74	F96	Lack of fac	More hung	0.4
Map72	F74	F147	Lack of fac	Stress and	0.4
Map72	F147	F01	Stress and	Bad experi	1
Map72	F10	F83	Compulsor	Malnutrition	0.2
Map72	F10	F01	Compulsor	Bad experi	0.2
Map72	F10	F64	Compulsor	Increased p	0.2
Map72	F10	F63	Compulsor	Increased c	0.2
Map72	F10	F152	Compulsor	Unable to b	0.2
Map72	F10	F96	Compulsor	More hung	0.2
Map72	F10	F74	Compulsor	Lack of fac	0.2
Map72	F10	F147	Compulsor	Stress and	0.2
Map72	F70	F83	Jobs lost	Malnutrition	0.6
Map72	F70	F01	Jobs lost	Bad experi	0.6
Map72	F70	F64	Jobs lost	Increased p	0.6
Map72	F70	F63	Jobs lost	Increased c	0.6
Map72	F70	F152	Jobs lost	Unable to b	0.6

For Peer Review

Map72	F70	F96	Jobs lost	More hung	0.6
Map72	F70	F147	Jobs lost	Stress and	0.4
Map72	F146	F83	Street haw	Malnutritior	0.6
Map72	F146	F01	Street haw	Bad experi	0.6
Map72	F146	F64	Street haw	Increased	0.6
Map72	F146	F63	Street haw	Increased	0.6
Map72	F146	F152	Street haw	Unable to	0.6
Map72	F146	F96	Street haw	More hung	0.6
Map72	F146	F147	Street haw	Stress and	0.4
Map72	F143	F83	Shortage o	Malnutritior	0.6
Map72	F143	F01	Shortage o	Bad experi	0.6
Map72	F143	F63	Shortage o	Increased	0.6
Map72	F143	F96	Shortage o	More hung	1
Map72	F140	F83	Restriction	Malnutritior	0.6
Map72	F140	F01	Restriction	Bad experi	0.6
Map72	F140	F64	Restriction	Increased	0.6
Map72	F140	F63	Restriction	Increased	0.6
Map72	F140	F62	Restriction	Increased	0.6
Map72	F140	F152	Restriction	Unable to	0.6
Map72	F140	F118	Restriction	No social g	0.6
Map72	F140	F96	Restriction	More hung	0.6
Map72	F140	F110	Restriction	No funerals	0.6
Map72	F140	F112	Restriction	No home/p	0.4
Map72	F140	F147	Restriction	Stress and	0.4
Map72	F140	F70	Restriction	Jobs lost	0.6
Map72	F140	F143	Restriction	Shortage o	0.6
Map72	F140	F121	Restriction	No weddin	0.6
Map72	F140	F123	Restriction	Not going t	0.6
Map72	F140	F104	Restriction	No buyers	0.4
Map72	F121	F01	No weddin	Bad experi	0.6
Map72	F121	F62	No weddin	Increased	0.6
Map72	F120	F83	No transpo	Malnutritior	0.6
Map72	F120	F01	No transpo	Bad experi	0.6
Map72	F120	F64	No transpo	Increased	0.6
Map72	F120	F63	No transpo	Increased	0.6
Map72	F120	F152	No transpo	Unable to	0.6
Map72	F120	F96	No transpo	More hung	0.6
Map72	F120	F147	No transpo	Stress and	0.4
Map72	F120	F146	No transpo	Street haw	0.6
Map72	F123	F83	Not going t	Malnutritior	0.6
Map72	F123	F01	Not going t	Bad experi	0.6
Map72	F123	F63	Not going t	Increased	0.6
Map72	F123	F96	Not going t	More hung	0.8
Map72	F123	F143	Not going t	Shortage o	0.8
Map72	F144	F01	Social dista	Bad experi	0.8
Map72	F144	F147	Social dista	Stress and	0.8
Map72	F144	F106	Social dista	No congre	0.8
Map72	F106	F01	No congre	Bad experi	0.8
Map72	F106	F147	No congre	Stress and	0.8
Map72	F104	F83	No buyers	Malnutritior	0.2
Map72	F104	F01	No buyers	Bad experi	0.2
Map72	F104	F64	No buyers	Increased	0.2
Map72	F104	F63	No buyers	Increased	0.2
Map72	F104	F152	No buyers	Unable to	0.2
Map72	F104	F96	No buyers	More hung	0.2
Map72	F104	F147	No buyers	Stress and	0.2

For Peer Review

Map72	F100	F01	Mosques/c	Bad experi	0.6
Map72	F100	F147	Mosques/c	Stress and	0.6
Map72	F100	F106	Mosques/c	No congreg	0.6
Map73	F47	F01	Increase in	Bad experi	0.4
Map73	F49	F01	Increase in	Bad experi	0.8
Map73	F87	F47	Men stayin	Increase in	0.6
Map73	F87	F01	Men stayin	Bad experi	0.4
Map73	F87	F72	Men stayin	Lack of bas	0.8
Map73	F80	F47	Lockdown	Increase in	0.6
Map73	F80	F49	Lockdown	Increase in	0.2
Map73	F80	F87	Lockdown	Men stayin	0.6
Map73	F80	F82	Lockdown	Lost invest	0.6
Map73	F80	F07	Lockdown	Businesses	0.6
Map73	F80	F01	Lockdown	Bad experi	0.6
Map73	F80	F67	Lockdown	Increased s	0.4
Map73	F80	F64	Lockdown	Increased p	0.6
Map73	F80	F63	Lockdown	Increased c	0.4
Map73	F80	F60	Lockdown	Increased i	0.4
Map73	F80	F117	Lockdown	No religiou	0.6
Map73	F80	F158	Lockdown	Youth/child	0.6
Map73	F80	F96	Lockdown	More hung	0.6
Map73	F80	F14	Lockdown	Could not g	0.4
Map73	F80	F115	Lockdown	No preachi	0.6
Map73	F80	F131	Lockdown	Physical pu	0.4
Map73	F80	F142	Lockdown	School/mad	0.8
Map73	F80	F58	Lockdown	Increased c	0.4
Map73	F80	F31	Lockdown	Fear of eac	1
Map73	F80	F38	Lockdown	Health wor	0.4
Map73	F80	F147	Lockdown	Stress and	0.8
Map73	F80	F16	Lockdown	Delay in ec	0.6
Map73	F80	F72	Lockdown	Lack of bas	0.6
Map73	F80	F124	Lockdown	Not visiting	0.4
Map73	F80	F141	Lockdown	Rumors on	0.8
Map73	F80	F140	Lockdown	Restriction	0.6
Map73	F80	F121	Lockdown	No wedding	0.2
Map73	F80	F106	Lockdown	No congreg	0.8
Map73	F80	F100	Lockdown	Mosques/c	0.8
Map73	F82	F47	Lost invest	Increase in	0.6
Map73	F82	F87	Lost invest	Men stayin	0.6
Map73	F82	F01	Lost invest	Bad experi	1
Map73	F82	F64	Lost invest	Increased p	1
Map73	F82	F96	Lost invest	More hung	1
Map73	F82	F72	Lost invest	Lack of bas	0.6
Map73	F07	F47	Businesses	Increase in	0.6
Map73	F07	F87	Businesses	Men stayin	0.6
Map73	F07	F82	Businesses	Lost invest	0.6
Map73	F07	F01	Businesses	Bad experi	0.6
Map73	F07	F64	Businesses	Increased p	0.6
Map73	F07	F96	Businesses	More hung	0.6
Map73	F07	F72	Businesses	Lack of bas	0.6
Map73	F67	F01	Increased s	Bad experi	0.6
Map73	F67	F131	Increased s	Physical pu	0.6
Map73	F64	F01	Increased p	Bad experi	1
Map73	F64	F96	Increased p	More hung	1
Map73	F63	F01	Increased c	Bad experi	0.6
Map73	F60	F01	Increased i	Bad experi	0.6

For Peer Review

Map73	F60	F67	Increased i	Increased s	0.6
Map73	F60	F131	Increased i	Physical pu	0.6
Map73	F60	F58	Increased i	Increased (	0.6
Map73	F117	F01	No religiou	Bad experi	0.6
Map73	F117	F147	No religiou	Stress and	0.6
Map73	F158	F01	Youth/child	Bad experi	0.4
Map73	F158	F67	Youth/child	Increased s	0.4
Map73	F158	F60	Youth/child	Increased i	0.4
Map73	F158	F131	Youth/child	Physical pu	0.4
Map73	F158	F58	Youth/child	Increased (	0.4
Map73	F96	F01	More hung	Bad experi	1
Map73	F14	F01	Could not g	Bad experi	0.6
Map73	F14	F63	Could not g	Increased (	0.8
Map73	F115	F01	No preachi	Bad experi	0.4
Map73	F115	F147	No preachi	Stress and	0.4
Map73	F131	F01	Physical pu	Bad experi	0.6
Map73	F142	F47	School/ma	Increase in	0.6
Map73	F142	F87	School/ma	Men stayin	0.6
Map73	F142	F82	School/ma	Lost invest	0.6
Map73	F142	F01	School/ma	Bad experi	0.6
Map73	F142	F64	School/ma	Increased p	0.6
Map73	F142	F96	School/ma	More hung	0.6
Map73	F142	F16	School/ma	Delay in ec	0.6
Map73	F142	F72	School/ma	Lack of bas	0.6
Map73	F58	F01	Increased (	Bad experi	0.4
Map73	F58	F131	Increased (	Physical pu	0.4
Map73	F31	F49	Fear of eac	Increase in	0.2
Map73	F31	F01	Fear of eac	Bad experi	0.4
Map73	F31	F63	Fear of eac	Increased (	0.4
Map73	F31	F14	Fear of eac	Could not g	0.4
Map73	F31	F38	Fear of eac	Health wor	0.4
Map73	F31	F124	Fear of eac	Not visiting	0.4
Map73	F31	F141	Fear of eac	Rumors on	0.8
Map73	F31	F121	Fear of eac	No wedding	0.2
Map73	F38	F49	Health wor	Increase in	0.2
Map73	F38	F01	Health wor	Bad experi	0.6
Map73	F38	F63	Health wor	Increased (	0.6
Map73	F38	F14	Health wor	Could not g	0.6
Map73	F38	F124	Health wor	Not visiting	0.8
Map73	F38	F121	Health wor	No wedding	0.2
Map73	F147	F01	Stress and	Bad experi	0.6
Map73	F16	F01	Delay in ec	Bad experi	0.8
Map73	F72	F47	Lack of bas	Increase in	0.6
Map73	F72	F01	Lack of bas	Bad experi	0.4
Map73	F124	F49	Not visiting	Increase in	0.2
Map73	F124	F01	Not visiting	Bad experi	0.6
Map73	F124	F63	Not visiting	Increased (	0.6
Map73	F124	F14	Not visiting	Could not g	0.6
Map73	F124	F121	Not visiting	No wedding	0.2
Map73	F141	F49	Rumors on	Increase in	0.2
Map73	F141	F01	Rumors on	Bad experi	0.4
Map73	F141	F63	Rumors on	Increased (	0.4
Map73	F141	F14	Rumors on	Could not g	0.4
Map73	F141	F38	Rumors on	Health wor	0.4
Map73	F141	F124	Rumors on	Not visiting	0.4
Map73	F141	F121	Rumors on	No wedding	0.2

For Peer Review

Map73	F140	F47	Restriction	Increase in	0.6
Map73	F140	F87	Restriction	Men stayin	0.6
Map73	F140	F82	Restriction	Lost invest	0.6
Map73	F140	F07	Restriction	Businesses	0.8
Map73	F140	F01	Restriction	Bad experi	0.6
Map73	F140	F67	Restriction	Increased s	0.4
Map73	F140	F64	Restriction	Increased p	0.6
Map73	F140	F60	Restriction	Increased i	0.4
Map73	F140	F158	Restriction	Youth/child	0.6
Map73	F140	F96	Restriction	More hung	0.6
Map73	F140	F131	Restriction	Physical pu	0.4
Map73	F140	F58	Restriction	Increased c	0.4
Map73	F140	F72	Restriction	Lack of bas	0.6
Map73	F121	F49	No wedding	Increase in	0.4
Map73	F121	F01	No wedding	Bad experi	0.4
Map73	F121	F121	No wedding	No wedding	0.2
Map73	F106	F01	No congreg	Bad experi	0.6
Map73	F106	F147	No congreg	Stress and	0.8
Map73	F100	F01	Mosques/c	Bad experi	0.6
Map73	F100	F117	Mosques/c	No religiou	0.6
Map73	F100	F115	Mosques/c	No preachi	0.6
Map73	F100	F147	Mosques/c	Stress and	0.8
Map73	F100	F106	Mosques/c	No congreg	1
Map74	F40	F01	Household	Bad experi	0.6
Map74	F40	F131	Household	Physical pu	0.6
Map74	F85	F83	Markets/sh	Malnutrition	0.6
Map74	F85	F82	Markets/sh	Lost invest	0.6
Map74	F85	F07	Markets/sh	Businesses	0.8
Map74	F85	F01	Markets/sh	Bad experi	0.6
Map74	F85	F64	Markets/sh	Increased p	0.6
Map74	F85	F96	Markets/sh	More hung	0.6
Map74	F85	F78	Markets/sh	Less food t	0.6
Map74	F85	F143	Markets/sh	Shortage o	0.6
Map74	F80	F40	Lockdown	Household	0.6
Map74	F80	F85	Lockdown	Markets/sh	0.8
Map74	F80	F83	Lockdown	Malnutrition	0.6
Map74	F80	F82	Lockdown	Lost invest	0.6
Map74	F80	F07	Lockdown	Businesses	0.8
Map74	F80	F01	Lockdown	Bad experi	0.8
Map74	F80	F64	Lockdown	Increased p	0.6
Map74	F80	F63	Lockdown	Increased c	0.6
Map74	F80	F158	Lockdown	Youth/child	0.8
Map74	F80	F96	Lockdown	More hung	0.6
Map74	F80	F133	Lockdown	Poor perfor	0.6
Map74	F80	F131	Lockdown	Physical pu	0.6
Map74	F80	F142	Lockdown	School/mac	0.8
Map74	F80	F118	Lockdown	No social g	0.6
Map74	F80	F78	Lockdown	Less food t	0.6
Map74	F80	F16	Lockdown	Delay in ec	0.6
Map74	F80	F143	Lockdown	Shortage o	0.6
Map74	F80	F124	Lockdown	Not visiting	0.6
Map74	F80	F140	Lockdown	Restriction	1
Map74	F80	F147	Lockdown	Stress and	0.8
Map74	F80	F120	Lockdown	No transpo	0.6
Map74	F80	F144	Lockdown	Social dista	0.8
Map74	F80	F106	Lockdown	No congreg	0.8

For Peer Review

Map74	F80	F100	Lockdown	Mosques/c	0.8
Map74	F83	F01	Malnutritior	Bad experi	0.6
Map74	F82	F01	Lost invest	Bad experi	0.6
Map74	F82	F64	Lost invest	Increased	0.6
Map74	F07	F82	Businesses	Lost invest	0.6
Map74	F07	F01	Businesses	Bad experi	0.6
Map74	F07	F64	Businesses	Increased	0.6
Map74	F20	F01	Difficult bre	Bad experi	0.4
Map74	F29	F01	Fear of cor	Bad experi	0.4
Map74	F29	F31	Fear of cor	Fear of eac	0.4
Map74	F64	F01	Increased	Bad experi	0.8
Map74	F63	F01	Increased	Bad experi	0.8
Map74	F158	F40	Youth/child	Household	0.6
Map74	F158	F01	Youth/child	Bad experi	0.6
Map74	F158	F131	Youth/child	Physical pu	0.6
Map74	F96	F83	More hung	Malnutritior	0.6
Map74	F96	F01	More hung	Bad experi	1
Map74	F133	F01	Poor perfor	Bad experi	0.8
Map74	F133	F16	Poor perfor	Delay in ec	0.8
Map74	F131	F01	Physical pu	Bad experi	0.8
Map74	F142	F40	School/ma	Household	0.6
Map74	F142	F01	School/ma	Bad experi	0.6
Map74	F142	F158	School/ma	Youth/child	0.8
Map74	F142	F133	School/ma	Poor perfor	0.6
Map74	F142	F131	School/ma	Physical pu	0.6
Map74	F142	F16	School/ma	Delay in ec	0.6
Map74	F30	F01	Fear of dea	Bad experi	0.4
Map74	F30	F29	Fear of dea	Fear of cor	0.4
Map74	F30	F31	Fear of dea	Fear of eac	0.4
Map74	F31	F01	Fear of eac	Bad experi	0.4
Map74	F118	F01	No social g	Bad experi	0.4
Map74	F118	F147	No social g	Stress and	0.4
Map74	F78	F83	Less food t	Malnutritior	0.6
Map74	F78	F01	Less food t	Bad experi	0.8
Map74	F78	F96	Less food t	More hung	0.8
Map74	F10	F20	Compulsor	Difficult bre	0.2
Map74	F10	F01	Compulsor	Bad experi	0.2
Map74	F16	F01	Delay in ec	Bad experi	0.8
Map74	F143	F83	Shortage o	Malnutritior	0.6
Map74	F143	F01	Shortage o	Bad experi	0.6
Map74	F143	F96	Shortage o	More hung	0.6
Map74	F143	F78	Shortage o	Less food t	0.6
Map74	F124	F01	Not visiting	Bad experi	0.8
Map74	F124	F63	Not visiting	Increased	0.8
Map74	F140	F01	Restriction	Bad experi	0.6
Map74	F140	F63	Restriction	Increased	0.6
Map74	F140	F124	Restriction	Not visiting	0.6
Map74	F140	F120	Restriction	No transpo	0.6
Map74	F147	F01	Stress and	Bad experi	0.8
Map74	F120	F01	No transpo	Bad experi	0.8
Map74	F120	F63	No transpo	Increased	0.8
Map74	F120	F124	No transpo	Not visiting	0.8
Map74	F144	F01	Social dista	Bad experi	0.8
Map74	F144	F118	Social dista	No social g	0.6
Map74	F144	F147	Social dista	Stress and	0.8
Map74	F144	F106	Social dista	No congreg	0.8

For Peer Review

Map74	F144	F100	Social dista	Mosques/c	1
Map74	F106	F01	No congreg	Bad experi	0.8
Map74	F106	F147	No congreg	Stress and	0.8
Map74	F100	F01	Mosques/c	Bad experi	0.8
Map74	F100	F147	Mosques/c	Stress and	0.8
Map74	F100	F106	Mosques/c	No congreg	0.8
Map75	F40	F47	Household	Increase in	0.2
Map75	F40	F01	Household	Bad experi	0.2
Map75	F88	F40	Men unable	Household	0.4
Map75	F88	F47	Men unable	Increase in	0.2
Map75	F88	F01	Men unable	Bad experi	0.2
Map75	F47	F01	Increase in	Bad experi	0.4
Map75	F85	F82	Markets/sh	Lost invest	0.6
Map75	F85	F07	Markets/sh	Businesses	1
Map75	F85	F01	Markets/sh	Bad experi	0.6
Map75	F85	F64	Markets/sh	Increased	0.6
Map75	F85	F61	Markets/sh	Increased	0.2
Map75	F87	F40	Men stayin	Household	0.4
Map75	F87	F88	Men stayin	Men unable	0.6
Map75	F87	F47	Men stayin	Increase in	0.2
Map75	F87	F01	Men stayin	Bad experi	0.2
Map75	F80	F40	Lockdown	Household	0.4
Map75	F80	F88	Lockdown	Men unable	0.6
Map75	F80	F47	Lockdown	Increase in	0.2
Map75	F80	F85	Lockdown	Markets/sh	0.6
Map75	F80	F87	Lockdown	Men stayin	0.8
Map75	F80	F83	Lockdown	Malnutrition	0.4
Map75	F80	F82	Lockdown	Lost invest	0.6
Map75	F80	F07	Lockdown	Businesses	0.6
Map75	F80	F01	Lockdown	Bad experi	0.6
Map75	F80	F67	Lockdown	Increased	0.6
Map75	F80	F64	Lockdown	Increased	0.6
Map75	F80	F63	Lockdown	Increased	0.4
Map75	F80	F62	Lockdown	Increased	0.4
Map75	F80	F61	Lockdown	Increased	0.2
Map75	F80	F158	Lockdown	Youth/child	0.6
Map75	F80	F96	Lockdown	More hung	0.6
Map75	F80	F130	Lockdown	Physical in	0.4
Map75	F80	F110	Lockdown	No funerals	0.4
Map75	F80	F135	Lockdown	Price hike	0.6
Map75	F80	F115	Lockdown	No preachi	0.6
Map75	F80	F13	Lockdown	Could not	0.4
Map75	F80	F16	Lockdown	Delay in ec	0.8
Map75	F80	F143	Lockdown	Shortage o	0.6
Map75	F80	F142	Lockdown	School/ma	1
Map75	F80	F147	Lockdown	Stress and	0.4
Map75	F80	F120	Lockdown	No transpo	0.6
Map75	F80	F106	Lockdown	No congreg	0.6
Map75	F80	F100	Lockdown	Mosques/c	1
Map75	F83	F01	Malnutrition	Bad experi	0.4
Map75	F82	F01	Lost invest	Bad experi	0.6
Map75	F82	F64	Lost invest	Increased	0.8
Map75	F82	F61	Lost invest	Increased	0.2
Map75	F07	F82	Businesses	Lost invest	0.6
Map75	F07	F01	Businesses	Bad experi	0.6
Map75	F07	F64	Businesses	Increased	0.6

For Peer Review

Map75	F07	F61	Businesses	Increased l	0.2
Map75	F20	F01	Difficult bre	Bad experi	0.4
Map75	F67	F01	Increased s	Bad experi	0.6
Map75	F29	F20	Fear of cor	Difficult bre	0.4
Map75	F29	F01	Fear of cor	Bad experi	0.4
Map75	F29	F10	Fear of cor	Compulsor	0.5
Map75	F29	F126	Fear of cor	Odour of sa	0.4
Map75	F64	F01	Increased p	Bad experi	0.6
Map75	F64	F61	Increased p	Increased l	0.2
Map75	F63	F01	Increased c	Bad experi	0.4
Map75	F62	F01	Increased r	Bad experi	0.4
Map75	F61	F01	Increased l	Bad experi	0.4
Map75	F158	F01	Youth/child	Bad experi	0.6
Map75	F158	F67	Youth/child	Increased s	0.8
Map75	F96	F83	More hung	Malnutritior	0.4
Map75	F96	F01	More hung	Bad experi	0.4
Map75	F96	F63	More hung	Increased c	0.4
Map75	F118	F01	No social g	Bad experi	0.6
Map75	F118	F62	No social g	Increased r	0.4
Map75	F118	F147	No social g	Stress and	0.6
Map75	F130	F01	Physical inj	Bad experi	0.6
Map75	F110	F01	No funerals	Bad experi	0.8
Map75	F110	F62	No funerals	Increased r	0.4
Map75	F110	F147	No funerals	Stress and	0.8
Map75	F135	F83	Price hike	Malnutritior	0.4
Map75	F135	F01	Price hike	Bad experi	0.4
Map75	F135	F63	Price hike	Increased c	0.4
Map75	F135	F96	Price hike	More hung	0.6
Map75	F32	F01	Feeling soc	Bad experi	0.4
Map75	F32	F62	Feeling soc	Increased r	0.4
Map75	F115	F01	No preachi	Bad experi	0.8
Map75	F13	F01	Could not c	Bad experi	0.4
Map75	F13	F130	Could not c	Physical inj	0.4
Map75	F10	F20	Compulsor	Difficult bre	0.4
Map75	F10	F01	Compulsor	Bad experi	0.4
Map75	F10	F126	Compulsor	Odour of sa	0.4
Map75	F16	F01	Delay in ed	Bad experi	0.6
Map75	F143	F83	Shortage o	Malnutritior	0.4
Map75	F143	F01	Shortage o	Bad experi	0.4
Map75	F143	F63	Shortage o	Increased c	0.4
Map75	F143	F96	Shortage o	More hung	0.6
Map75	F143	F135	Shortage o	Price hike	0.8
Map75	F142	F01	School/ma	Bad experi	0.6
Map75	F142	F67	School/ma	Increased s	0.6
Map75	F142	F158	School/ma	Youth/child	0.6
Map75	F142	F130	School/ma	Physical inj	0.4
Map75	F142	F13	School/ma	Could not c	0.4
Map75	F142	F16	School/ma	Delay in ec	0.8
Map75	F126	F20	Odour of sa	Difficult bre	0.2
Map75	F126	F01	Odour of sa	Bad experi	0.2
Map75	F147	F01	Stress and	Bad experi	0.8
Map75	F147	F62	Stress and	Increased r	0.4
Map75	F120	F83	No transpo	Malnutritior	0.4
Map75	F120	F01	No transpo	Bad experi	0.4
Map75	F120	F63	No transpo	Increased c	0.4
Map75	F120	F96	No transpo	More hung	0.6

For Peer Review

Map75	F120	F135	No transpo	Price hike	0.8
Map75	F120	F143	No transpo	Shortage o	0.8
Map75	F144	F01	Social dista	Bad experi	0.6
Map75	F144	F62	Social dista	Increased r	0.4
Map75	F144	F118	Social dista	No social g	0.6
Map75	F144	F32	Social dista	Feeling soc	0.4
Map75	F144	F147	Social dista	Stress and	0.6
Map75	F106	F01	No congreg	Bad experi	0.8
Map75	F106	F62	No congreg	Increased r	0.4
Map75	F106	F110	No congreg	No funerals	0.4
Map75	F106	F115	No congreg	No preachi	0.8
Map75	F106	F147	No congreg	Stress and	0.4
Map75	F100	F01	Mosques/c	Bad experi	0.6
Map75	F100	F62	Mosques/c	Increased r	0.4
Map75	F100	F110	Mosques/c	No funerals	0.4
Map75	F100	F115	Mosques/c	No preachi	0.6
Map75	F100	F147	Mosques/c	Stress and	0.4
Map75	F100	F106	Mosques/c	No congreg	0.6
Map76	F89	F62	Misconcep	Increased r	0.6
Map76	F89	F01	Misconcep	Bad experi	0.6
Map76	F49	F01	Increase in	Bad experi	0.4
Map76	F49	F99	Increase in	More unwa	1
Map76	F80	F49	Lockdown	Increase in	0.6
Map76	F80	F05	Lockdown	Banks clos	0.6
Map76	F80	F07	Lockdown	Businesses	0.6
Map76	F80	F27	Lockdown	Fear of bei	0.6
Map76	F80	F64	Lockdown	Increased p	0.6
Map76	F80	F01	Lockdown	Bad experi	0.6
Map76	F80	F158	Lockdown	Youth/child	0.6
Map76	F80	F16	Lockdown	Delay in ec	0.6
Map76	F80	F93	Lockdown	More dema	0.6
Map76	F80	F96	Lockdown	More hung	0.6
Map76	F80	F94	Lockdown	More hous	0.6
Map76	F80	F99	Lockdown	More unwa	0.6
Map76	F80	F135	Lockdown	Price hike	0.6
Map76	F80	F56	Lockdown	Increased c	0.4
Map76	F80	F85	Lockdown	Markets/sh	0.8
Map76	F80	F78	Lockdown	Less food t	0.6
Map76	F80	F115	Lockdown	No preachi	0.6
Map76	F80	F70	Lockdown	Jobs lost	0.6
Map76	F80	F72	Lockdown	Lack of bas	0.6
Map76	F80	F142	Lockdown	School/mac	0.6
Map76	F80	F140	Lockdown	Restriction	0.6
Map76	F80	F147	Lockdown	Stress and	0.6
Map76	F80	F120	Lockdown	No transpo	0.6
Map76	F80	F106	Lockdown	No congreg	0.6
Map76	F80	F100	Lockdown	Mosques/c	0.6
Map76	F05	F05	Banks clos	Banks clos	0.6
Map76	F05	F01	Banks clos	Bad experi	0.8
Map76	F05	F96	Banks clos	More hung	0.8
Map76	F05	F78	Banks clos	Less food t	0.8
Map76	F07	F01	Businesses	Bad experi	0.6
Map76	F07	F96	Businesses	More hung	0.6
Map76	F07	F135	Businesses	Price hike	0.6
Map76	F07	F78	Businesses	Less food t	0.6
Map76	F07	F72	Businesses	Lack of bas	0.6

For Peer Review

Map76	F27	F64	Fear of bei	Increased	0.2
Map76	F27	F01	Fear of bei	Bad experi	0.2
Map76	F27	F56	Fear of bei	Increased	0.4
Map76	F27	F72	Fear of bei	Lack of bas	0.2
Map76	F25	F89	Enforced C	Misconcep	0.4
Map76	F25	F62	Enforced C	Increased	0.4
Map76	F25	F01	Enforced C	Bad experi	0.4
Map76	F29	F89	Fear of cor	Misconcep	0.4
Map76	F29	F25	Fear of cor	Enforced C	0.6
Map76	F29	F62	Fear of cor	Increased	0.4
Map76	F29	F01	Fear of cor	Bad experi	0.4
Map76	F29	F154	Fear of cor	Wearing fa	0.6
Map76	F29	F31	Fear of cor	Fear of eac	0.6
Map76	F29	F74	Fear of cor	Lack of fac	0.4
Map76	F64	F01	Increased	Bad experi	0.8
Map76	F64	F72	Increased	Lack of bas	0.8
Map76	F62	F01	Increased	Bad experi	0.6
Map76	F154	F01	Wearing fa	Bad experi	0.2
Map76	F154	F74	Wearing fa	Lack of fac	0.4
Map76	F158	F49	Youth/child	Increase in	0.6
Map76	F158	F01	Youth/child	Bad experi	0.6
Map76	F158	F93	Youth/child	More dema	0.6
Map76	F158	F94	Youth/child	More hous	0.6
Map76	F158	F99	Youth/child	More unwa	0.6
Map76	F16	F01	Delay in ed	Bad experi	0.8
Map76	F93	F01	More dema	Bad experi	0.6
Map76	F93	F94	More dema	More hous	0.6
Map76	F96	F01	More hung	Bad experi	0.8
Map76	F94	F01	More hous	Bad experi	0.6
Map76	F114	F01	No pilgrima	Bad experi	0.4
Map76	F114	F147	No pilgrima	Stress and	0.4
Map76	F118	F49	No social g	Increase in	0.4
Map76	F118	F01	No social g	Bad experi	0.4
Map76	F118	F99	No social g	More unwa	0.4
Map76	F118	F121	No social g	No weddin	0.8
Map76	F99	F01	More unwa	Bad experi	0.4
Map76	F111	F01	No governr	Bad experi	0.4
Map76	F111	F94	No governr	More hous	0.4
Map76	F135	F01	Price hike	Bad experi	0.8
Map76	F135	F96	Price hike	More hung	0.8
Map76	F135	F78	Price hike	Less food t	0.8
Map76	F135	F72	Price hike	Lack of bas	1
Map76	F56	F64	Increased	Increased	0.2
Map76	F56	F01	Increased	Bad experi	0.2
Map76	F56	F72	Increased	Lack of bas	0.2
Map76	F85	F07	Markets/sh	Businesses	0.6
Map76	F85	F01	Markets/sh	Bad experi	0.6
Map76	F85	F96	Markets/sh	More hung	0.6
Map76	F85	F135	Markets/sh	Price hike	0.6
Map76	F85	F78	Markets/sh	Less food t	0.6
Map76	F85	F72	Markets/sh	Lack of bas	0.6
Map76	F31	F89	Fear of eac	Misconcep	0.4
Map76	F31	F25	Fear of eac	Enforced C	0.6
Map76	F31	F62	Fear of eac	Increased	0.4
Map76	F31	F01	Fear of eac	Bad experi	0.4
Map76	F78	F01	Less food t	Bad experi	0.8

For Peer Review

Map76	F78	F96	Less food t	More hung	0.8
Map76	F115	F01	No preachi	Bad experi	0.8
Map76	F115	F147	No preachi	Stress and	0.8
Map76	F74	F01	Lack of fac	Bad experi	0.2
Map76	F121	F49	No wedding	Increase in	0.4
Map76	F121	F01	No wedding	Bad experi	0.4
Map76	F121	F99	No wedding	More unwa	0.4
Map76	F70	F64	Jobs lost	Increased p	1
Map76	F70	F01	Jobs lost	Bad experi	0.8
Map76	F70	F72	Jobs lost	Lack of bas	0.8
Map76	F72	F01	Lack of bas	Bad experi	0.8
Map76	F142	F01	School/ma	Bad experi	0.8
Map76	F142	F16	School/ma	Delay in ec	0.8
Map76	F141	F89	Rumors on	Misconcep	0.4
Map76	F141	F25	Rumors on	Enforced C	0.6
Map76	F141	F29	Rumors on	Fear of cor	1
Map76	F141	F62	Rumors on	Increased r	0.4
Map76	F141	F01	Rumors on	Bad experi	0.4
Map76	F141	F154	Rumors on	Wearing fa	0.6
Map76	F141	F31	Rumors on	Fear of eac	0.6
Map76	F141	F74	Rumors on	Lack of fac	0.4
Map76	F140	F49	Restriction	Increase in	0.6
Map76	F140	F27	Restriction	Fear of bei	0.6
Map76	F140	F64	Restriction	Increased p	0.6
Map76	F140	F01	Restriction	Bad experi	0.6
Map76	F140	F158	Restriction	Youth/child	0.6
Map76	F140	F16	Restriction	Delay in ec	0.6
Map76	F140	F93	Restriction	More dema	0.6
Map76	F140	F94	Restriction	More hous	0.6
Map76	F140	F99	Restriction	More unwa	0.6
Map76	F140	F56	Restriction	Increased c	0.4
Map76	F140	F115	Restriction	No preachi	0.6
Map76	F140	F70	Restriction	Jobs lost	0.6
Map76	F140	F72	Restriction	Lack of bas	0.6
Map76	F140	F142	Restriction	School/ma	0.6
Map76	F140	F147	Restriction	Stress and	0.6
Map76	F140	F120	Restriction	No transpo	0.6
Map76	F140	F106	Restriction	No congreg	0.6
Map76	F140	F100	Restriction	Mosques/c	0.8
Map76	F147	F01	Stress and	Bad experi	1
Map76	F120	F27	No transpo	Fear of bei	0.6
Map76	F120	F64	No transpo	Increased p	1
Map76	F120	F01	No transpo	Bad experi	0.8
Map76	F120	F56	No transpo	Increased c	0.4
Map76	F120	F70	No transpo	Jobs lost	1
Map76	F120	F72	No transpo	Lack of bas	0.8
Map76	F144	F49	Social dista	Increase in	0.4
Map76	F144	F01	Social dista	Bad experi	0.4
Map76	F144	F114	Social dista	No pilgrima	0.6
Map76	F144	F118	Social dista	No social g	0.8
Map76	F144	F99	Social dista	More unwa	0.4
Map76	F144	F121	Social dista	No wedding	0.8
Map76	F144	F147	Social dista	Stress and	0.4
Map76	F106	F01	No congreg	Bad experi	0.6
Map76	F106	F147	No congreg	Stress and	0.6
Map76	F100	F01	Mosques/c	Bad experi	0.6

For Peer Review

Map76	F100	F115	Mosques/c	No preachi	0.6
Map76	F100	F147	Mosques/c	Stress and	0.6
Map76	F100	F106	Mosques/c	No congreg	0.6
Map77	F40	F47	Household	Increase in	0.4
Map77	F40	F01	Household	Bad experi	0.2
Map77	F40	F62	Household	Increased r	0.4
Map77	F88	F40	Men unable	Household	0.4
Map77	F88	F47	Men unable	Increase in	0.4
Map77	F88	F01	Men unable	Bad experi	0.2
Map77	F88	F62	Men unable	Increased r	0.4
Map77	F47	F01	Increase in	Bad experi	0.2
Map77	F47	F62	Increase in	Increased r	0.4
Map77	F87	F40	Men stayin	Household	0.4
Map77	F87	F88	Men stayin	Men unable	0.8
Map77	F87	F47	Men stayin	Increase in	0.4
Map77	F87	F01	Men stayin	Bad experi	0.6
Map77	F87	F64	Men stayin	Increased p	0.6
Map77	F87	F63	Men stayin	Increased c	0.4
Map77	F87	F62	Men stayin	Increased r	0.4
Map77	F87	F96	Men stayin	More hung	0.6
Map77	F87	F134	Men stayin	Poverty	0.4
Map77	F87	F78	Men stayin	Less food t	0.6
Map77	F87	F38	Men stayin	Health wor	0.4
Map77	F87	F70	Men stayin	Jobs lost	0.6
Map77	F87	F14	Men stayin	Could not g	0.4
Map77	F87	F147	Men stayin	Stress and	0.2
Map77	F80	F40	Lockdown	Household	0.4
Map77	F80	F88	Lockdown	Men unable	0.8
Map77	F80	F47	Lockdown	Increase in	0.4
Map77	F80	F87	Lockdown	Men stayin	0.8
Map77	F80	F01	Lockdown	Bad experi	0.8
Map77	F80	F68	Lockdown	Increased s	0.8
Map77	F80	F64	Lockdown	Increased p	0.8
Map77	F80	F63	Lockdown	Increased c	0.4
Map77	F80	F62	Lockdown	Increased r	0.4
Map77	F80	F157	Lockdown	Women un	0.8
Map77	F80	F16	Lockdown	Delay in ec	0.5
Map77	F80	F96	Lockdown	More hung	0.6
Map77	F80	F134	Lockdown	Poverty	0.4
Map77	F80	F78	Lockdown	Less food t	0.6
Map77	F80	F133	Lockdown	Poor perfor	0.4
Map77	F80	F38	Lockdown	Health wor	0.4
Map77	F80	F70	Lockdown	Jobs lost	0.6
Map77	F80	F14	Lockdown	Could not g	0.4
Map77	F80	F142	Lockdown	School/mad	0.8
Map77	F80	F140	Lockdown	Restriction	0.8
Map77	F80	F147	Lockdown	Stress and	0.4
Map77	F80	F120	Lockdown	No transpo	0.8
Map77	F80	F115	Lockdown	No preachi	0.6
Map77	F80	F106	Lockdown	No congreg	0.8
Map77	F80	F100	Lockdown	Mosques/c	1
Map77	F20	F01	Difficult bre	Bad experi	0.4
Map77	F68	F01	Increased s	Bad experi	0.8
Map77	F68	F64	Increased s	Increased p	1
Map77	F68	F63	Increased s	Increased c	0.4
Map77	F68	F134	Increased s	Poverty	0.4

For Peer Review

Map77	F68	F38	Increased s	Health wor	0.4
Map77	F68	F14	Increased s	Could not g	0.4
Map77	F68	F147	Increased s	Stress and	0.2
Map77	F29	F20	Fear of cor	Difficult bre	0.4
Map77	F29	F01	Fear of cor	Bad experi	0.4
Map77	F29	F64	Fear of cor	Increased p	0.4
Map77	F29	F63	Fear of cor	Increased c	0.4
Map77	F29	F134	Fear of cor	Poverty	0.4
Map77	F29	F30	Fear of cor	Fear of dea	0.4
Map77	F29	F31	Fear of cor	Fear of eac	0.4
Map77	F29	F38	Fear of cor	Health wor	0.4
Map77	F29	F10	Fear of cor	Compulsor	0.4
Map77	F29	F14	Fear of cor	Could not g	0.4
Map77	F29	F147	Fear of cor	Stress and	0.2
Map77	F64	F01	Increased p	Bad experi	0.8
Map77	F64	F63	Increased p	Increased c	0.4
Map77	F64	F134	Increased p	Poverty	0.4
Map77	F64	F38	Increased p	Health wor	0.4
Map77	F64	F14	Increased p	Could not g	0.4
Map77	F64	F147	Increased p	Stress and	0.2
Map77	F63	F01	Increased c	Bad experi	0.6
Map77	F62	F01	Increased r	Bad experi	0.2
Map77	F157	F01	Women un	Bad experi	0.6
Map77	F157	F64	Women un	Increased p	0.6
Map77	F157	F63	Women un	Increased c	0.4
Map77	F157	F96	Women un	More hung	0.6
Map77	F157	F134	Women un	Poverty	0.4
Map77	F157	F78	Women un	Less food t	0.6
Map77	F157	F38	Women un	Health wor	0.4
Map77	F157	F70	Women un	Jobs lost	0.6
Map77	F157	F14	Women un	Could not g	0.4
Map77	F157	F147	Women un	Stress and	0.2
Map77	F16	F01	Delay in ed	Bad experi	0.4
Map77	F16	F133	Delay in ed	Poor perfor	0.4
Map77	F16	F147	Delay in ed	Stress and	0.4
Map77	F96	F01	More hung	Bad experi	0.4
Map77	F96	F63	More hung	Increased c	0.4
Map77	F95	F40	More hous	Household	0.6
Map77	F95	F47	More hous	Increase in	0.4
Map77	F95	F01	More hous	Bad experi	0.2
Map77	F95	F62	More hous	Increased r	0.4
Map77	F118	F01	No social g	Bad experi	0.4
Map77	F118	F32	No social g	Feeling soc	0.6
Map77	F118	F147	No social g	Stress and	0.4
Map77	F111	F01	No governr	Bad experi	0.4
Map77	F111	F63	No governr	Increased c	0.4
Map77	F111	F96	No governr	More hung	0.4
Map77	F134	F01	Poverty	Bad experi	0.4
Map77	F134	F63	Poverty	Increased c	0.4
Map77	F134	F38	Poverty	Health wor	0.4
Map77	F134	F14	Poverty	Could not g	0.4
Map77	F30	F01	Fear of dea	Bad experi	0.4
Map77	F30	F31	Fear of dea	Fear of eac	0.4
Map77	F31	F01	Fear of eac	Bad experi	0.4
Map77	F32	F01	Feeling soc	Bad experi	0.4
Map77	F32	F147	Feeling soc	Stress and	0.4

For Peer Review

Map77	F78	F01	Less food t	Bad experi	0.4
Map77	F78	F63	Less food t	Increased	0.4
Map77	F78	F96	Less food t	More hung	1
Map77	F133	F01	Poor perfor	Bad experi	0.4
Map77	F133	F147	Poor perfor	Stress and	0.4
Map77	F38	F01	Health wor	Bad experi	0.6
Map77	F38	F63	Health wor	Increased	0.6
Map77	F38	F14	Health wor	Could not g	0.6
Map77	F10	F20	Compulsor	Difficult bre	0.4
Map77	F10	F01	Compulsor	Bad experi	0.6
Map77	F10	F64	Compulsor	Increased p	0.6
Map77	F10	F63	Compulsor	Increased	0.4
Map77	F10	F134	Compulsor	Poverty	0.4
Map77	F10	F38	Compulsor	Health wor	0.4
Map77	F10	F14	Compulsor	Could not g	0.4
Map77	F10	F147	Compulsor	Stress and	0.2
Map77	F70	F01	Jobs lost	Bad experi	0.8
Map77	F70	F64	Jobs lost	Increased p	0.8
Map77	F70	F63	Jobs lost	Increased	0.4
Map77	F70	F96	Jobs lost	More hung	0.8
Map77	F70	F134	Jobs lost	Poverty	0.4
Map77	F70	F78	Jobs lost	Less food t	0.8
Map77	F70	F38	Jobs lost	Health wor	0.4
Map77	F70	F14	Jobs lost	Could not g	0.4
Map77	F70	F147	Jobs lost	Stress and	0.2
Map77	F14	F01	Could not g	Bad experi	0.6
Map77	F14	F63	Could not g	Increased	0.8
Map77	F142	F01	School/ma	Bad experi	0.4
Map77	F142	F16	School/ma	Delay in ec	0.5
Map77	F142	F133	School/ma	Poor perfor	0.4
Map77	F142	F147	School/ma	Stress and	0.4
Map77	F141	F20	Rumors on	Difficult bre	0.4
Map77	F141	F01	Rumors on	Bad experi	0.4
Map77	F141	F29	Rumors on	Fear of cor	0.4
Map77	F141	F64	Rumors on	Increased p	0.4
Map77	F141	F63	Rumors on	Increased	0.4
Map77	F141	F134	Rumors on	Poverty	0.4
Map77	F141	F30	Rumors on	Fear of dea	0.4
Map77	F141	F31	Rumors on	Fear of eac	0.4
Map77	F141	F38	Rumors on	Health wor	0.4
Map77	F141	F10	Rumors on	Compulsor	0.4
Map77	F141	F14	Rumors on	Could not g	0.4
Map77	F141	F147	Rumors on	Stress and	0.2
Map77	F140	F40	Restriction	Household	0.4
Map77	F140	F88	Restriction	Men unable	0.8
Map77	F140	F47	Restriction	Increase in	0.4
Map77	F140	F87	Restriction	Men stayin	0.8
Map77	F140	F01	Restriction	Bad experi	0.6
Map77	F140	F64	Restriction	Increased p	0.6
Map77	F140	F63	Restriction	Increased	0.4
Map77	F140	F62	Restriction	Increased r	0.4
Map77	F140	F157	Restriction	Women un	0.8
Map77	F140	F96	Restriction	More hung	0.6
Map77	F140	F134	Restriction	Poverty	0.4
Map77	F140	F78	Restriction	Less food t	0.6
Map77	F140	F38	Restriction	Health wor	0.4

For Peer Review

Map77	F140	F70	Restriction	Jobs lost	0.6
Map77	F140	F14	Restriction	Could not g	0.4
Map77	F140	F147	Restriction	Stress and	0.2
Map77	F140	F120	Restriction	No transpo	0.8
Map77	F147	F01	Stress and	Bad experi	0.4
Map77	F120	F40	No transpo	Household	0.4
Map77	F120	F88	No transpo	Men unable	0.8
Map77	F120	F47	No transpo	Increase in	0.4
Map77	F120	F87	No transpo	Men stayin	0.8
Map77	F120	F01	No transpo	Bad experi	0.6
Map77	F120	F64	No transpo	Increased p	0.6
Map77	F120	F63	No transpo	Increased c	0.4
Map77	F120	F62	No transpo	Increased r	0.4
Map77	F120	F157	No transpo	Women un	0.8
Map77	F120	F96	No transpo	More hung	0.6
Map77	F120	F134	No transpo	Poverty	0.4
Map77	F120	F78	No transpo	Less food t	0.6
Map77	F120	F38	No transpo	Health wor	0.4
Map77	F120	F70	No transpo	Jobs lost	0.6
Map77	F120	F14	No transpo	Could not g	0.4
Map77	F120	F147	No transpo	Stress and	0.2
Map77	F144	F01	Social dista	Bad experi	0.4
Map77	F144	F118	Social dista	No social g	0.8
Map77	F144	F32	Social dista	Feeling soc	0.6
Map77	F144	F147	Social dista	Stress and	0.4
Map77	F115	F01	No preachi	Bad experi	0.2
Map77	F115	F147	No preachi	Stress and	0.2
Map77	F106	F01	No congreg	Bad experi	0.8
Map77	F106	F68	No congreg	Increased s	0.8
Map77	F106	F64	No congreg	Increased p	0.8
Map77	F106	F63	No congreg	Increased c	0.4
Map77	F106	F134	No congreg	Poverty	0.4
Map77	F106	F38	No congreg	Health wor	0.4
Map77	F106	F14	No congreg	Could not g	0.4
Map77	F106	F147	No congreg	Stress and	0.2
Map77	F106	F115	No congreg	No preachi	0.6
Map77	F100	F01	Mosques/c	Bad experi	0.8
Map77	F100	F68	Mosques/c	Increased s	0.8
Map77	F100	F64	Mosques/c	Increased p	0.8
Map77	F100	F63	Mosques/c	Increased c	0.4
Map77	F100	F134	Mosques/c	Poverty	0.4
Map77	F100	F38	Mosques/c	Health wor	0.4
Map77	F100	F14	Mosques/c	Could not g	0.4
Map77	F100	F147	Mosques/c	Stress and	0.2
Map77	F100	F115	Mosques/c	No preachi	0.6
Map77	F100	F106	Mosques/c	No congreg	0.8
Map78	F40	F47	Household	Increase in	1
Map78	F40	F01	Household	Bad experi	0.6
Map78	F47	F01	Increase in	Bad experi	0.6
Map78	F85	F40	Markets/sh	Household	0.6
Map78	F85	F47	Markets/sh	Increase in	0.6
Map78	F85	F01	Markets/sh	Bad experi	1
Map78	F85	F68	Markets/sh	Increased s	1
Map78	F85	F64	Markets/sh	Increased p	1
Map78	F85	F63	Markets/sh	Increased c	0.6
Map78	F85	F96	Markets/sh	More hung	0.6

For Peer Review

Map78	F85	F135	Markets/sh	Price hike	0.6
Map78	F85	F78	Markets/sh	Less food t	0.6
Map78	F85	F143	Markets/sh	Shortage o	0.6
Map78	F80	F40	Lockdown	Household	0.6
Map78	F80	F47	Lockdown	Increase in	0.6
Map78	F80	F85	Lockdown	Markets/sh	0.8
Map78	F80	F01	Lockdown	Bad experi	1
Map78	F80	F68	Lockdown	Increased s	0.8
Map78	F80	F64	Lockdown	Increased p	0.8
Map78	F80	F63	Lockdown	Increased c	0.8
Map78	F80	F158	Lockdown	Youth/child	0.6
Map78	F80	F96	Lockdown	More hung	0.6
Map78	F80	F133	Lockdown	Poor perfor	0.6
Map78	F80	F135	Lockdown	Price hike	0.8
Map78	F80	F19	Lockdown	Difficulty ac	0.8
Map78	F80	F78	Lockdown	Less food t	0.6
Map78	F80	F143	Lockdown	Shortage o	0.8
Map78	F80	F142	Lockdown	School/ma	0.6
Map78	F80	F147	Lockdown	Stress and	1
Map78	F80	F120	Lockdown	No transpo	1
Map78	F80	F106	Lockdown	No congreg	1
Map78	F23	F01	Difficulty w	Bad experi	1
Map78	F23	F63	Difficulty w	Increased c	0.8
Map78	F23	F147	Difficulty w	Stress and	1
Map78	F21	F01	Difficulty in	Bad experi	0.6
Map78	F20	F01	Difficult bre	Bad experi	0.2
Map78	F68	F01	Increased s	Bad experi	1
Map78	F68	F68	Increased s	Increased s	0.8
Map78	F68	F64	Increased s	Increased p	1
Map78	F29	F23	Fear of cor	Difficulty w	0.6
Map78	F29	F21	Fear of cor	Difficulty in	0.4
Map78	F29	F20	Fear of cor	Difficult bre	0.4
Map78	F29	F01	Fear of cor	Bad experi	0.8
Map78	F29	F68	Fear of cor	Increased s	0.8
Map78	F29	F64	Fear of cor	Increased p	0.8
Map78	F29	F63	Fear of cor	Increased c	0.6
Map78	F29	F30	Fear of cor	Fear of dea	0.4
Map78	F29	F32	Fear of cor	Feeling soc	0.6
Map78	F29	F10	Fear of cor	Compulsor	0.4
Map78	F29	F77	Fear of cor	Less careg	0.6
Map78	F29	F147	Fear of cor	Stress and	0.6
Map78	F29	F144	Fear of cor	Social dista	0.6
Map78	F64	F01	Increased p	Bad experi	1
Map78	F64	F68	Increased p	Increased s	0.6
Map78	F64	F64	Increased p	Increased p	0.6
Map78	F63	F01	Increased c	Bad experi	0.6
Map78	F158	F01	Youth/child	Bad experi	0.6
Map78	F158	F133	Youth/child	Poor perfor	0.6
Map78	F96	F40	More hung	Household	0.8
Map78	F96	F47	More hung	Increase in	0.8
Map78	F96	F01	More hung	Bad experi	1
Map78	F96	F63	More hung	Increased c	1
Map78	F133	F01	Poor perfor	Bad experi	0.8
Map78	F111	F01	No governr	Bad experi	1
Map78	F111	F68	No governr	Increased s	1
Map78	F111	F64	No governr	Increased p	1

For Peer Review

Map78	F135	F40	Price hike	Household	0.6
Map78	F135	F47	Price hike	Increase in	0.6
Map78	F135	F01	Price hike	Bad experi	0.6
Map78	F135	F63	Price hike	Increased (	0.6
Map78	F135	F96	Price hike	More hung	0.6
Map78	F135	F78	Price hike	Less food t	0.6
Map78	F30	F01	Fear of dea	Bad experi	0.6
Map78	F31	F01	Fear of eac	Bad experi	0.6
Map78	F32	F01	Feeling soc	Bad experi	1
Map78	F32	F63	Feeling soc	Increased (	0.8
Map78	F32	F147	Feeling soc	Stress and	1
Map78	F19	F01	Difficulty ac	Bad experi	0.6
Map78	F19	F63	Difficulty ac	Increased (	1
Map78	F78	F40	Less food t	Household	0.6
Map78	F78	F47	Less food t	Increase in	0.6
Map78	F78	F01	Less food t	Bad experi	0.6
Map78	F78	F63	Less food t	Increased (	0.6
Map78	F78	F96	Less food t	More hung	0.6
Map78	F10	F21	Compulsor	Difficulty in	0.6
Map78	F10	F20	Compulsor	Difficult bre	0.4
Map78	F10	F01	Compulsor	Bad experi	0.6
Map78	F77	F23	Less careg	Difficulty w	0.6
Map78	F77	F01	Less careg	Bad experi	0.6
Map78	F77	F63	Less careg	Increased (	0.6
Map78	F77	F147	Less careg	Stress and	0.6
Map78	F143	F40	Shortage o	Household	0.6
Map78	F143	F47	Shortage o	Increase in	0.6
Map78	F143	F01	Shortage o	Bad experi	0.6
Map78	F143	F63	Shortage o	Increased (	0.6
Map78	F143	F96	Shortage o	More hung	0.6
Map78	F143	F135	Shortage o	Price hike	0.8
Map78	F143	F78	Shortage o	Less food t	0.6
Map78	F142	F01	School/ma	Bad experi	0.6
Map78	F142	F158	School/ma	Youth/child	0.8
Map78	F142	F133	School/ma	Poor perfor	0.6
Map78	F147	F01	Stress and	Bad experi	1
Map78	F147	F63	Stress and	Increased (	0.8
Map78	F120	F40	No transpo	Household	0.6
Map78	F120	F47	No transpo	Increase in	0.6
Map78	F120	F01	No transpo	Bad experi	0.6
Map78	F120	F68	No transpo	Increased s	0.6
Map78	F120	F64	No transpo	Increased p	0.6
Map78	F120	F63	No transpo	Increased (	0.8
Map78	F120	F96	No transpo	More hung	0.6
Map78	F120	F135	No transpo	Price hike	0.8
Map78	F120	F19	No transpo	Difficulty ac	0.8
Map78	F120	F78	No transpo	Less food t	0.6
Map78	F120	F143	No transpo	Shortage o	0.8
Map78	F144	F23	Social dista	Difficulty w	0.6
Map78	F144	F01	Social dista	Bad experi	0.8
Map78	F144	F63	Social dista	Increased (	0.8
Map78	F144	F32	Social dista	Feeling soc	0.8
Map78	F144	F77	Social dista	Less careg	0.8
Map78	F144	F147	Social dista	Stress and	0.8
Map78	F106	F01	No congreg	Bad experi	1
Map78	F106	F63	No congreg	Increased (	0.8

For Peer Review

Map78	F106	F147	No congreg	Stress and	1
Map79	F40	F47	Household	Increase in	0.8
Map79	F40	F01	Household	Bad experi	0.6
Map79	F47	F01	Increase in	Bad experi	0.6
Map79	F87	F40	Men stayin	Household	0.8
Map79	F87	F47	Men stayin	Increase in	0.8
Map79	F87	F64	Men stayin	Increased p	0.8
Map79	F87	F01	Men stayin	Bad experi	0.6
Map79	F87	F96	Men stayin	More hung	0.6
Map79	F87	F78	Men stayin	Less food t	0.6
Map79	F87	F72	Men stayin	Lack of bas	0.8
Map79	F81	F40	Loss of Ha	Household	0.4
Map79	F81	F47	Loss of Ha	Increase in	0.4
Map79	F81	F64	Loss of Ha	Increased p	0.4
Map79	F81	F01	Loss of Ha	Bad experi	0.4
Map79	F81	F96	Loss of Ha	More hung	0.4
Map79	F81	F78	Loss of Ha	Less food t	0.4
Map79	F81	F72	Loss of Ha	Lack of bas	0.4
Map79	F80	F40	Lockdown	Household	0.4
Map79	F80	F47	Lockdown	Increase in	0.4
Map79	F80	F85	Lockdown	Markets/sh	0.6
Map79	F80	F81	Lockdown	Loss of Ha	0.4
Map79	F80	F27	Lockdown	Fear of bei	0.8
Map79	F80	F64	Lockdown	Increased p	0.4
Map79	F80	F01	Lockdown	Bad experi	0.8
Map79	F80	F114	Lockdown	No pilgrima	0.6
Map79	F80	F96	Lockdown	More hung	0.4
Map79	F80	F142	Lockdown	School/ma	1
Map79	F80	F78	Lockdown	Less food t	0.4
Map79	F80	F16	Lockdown	Delay in ec	0.6
Map79	F80	F72	Lockdown	Lack of bas	0.4
Map79	F80	F140	Lockdown	Restriction	0.8
Map79	F80	F147	Lockdown	Stress and	0.8
Map79	F80	F120	Lockdown	No transpo	0.8
Map79	F80	F106	Lockdown	No congreg	0.6
Map79	F80	F100	Lockdown	Mosques/c	0.8
Map79	F82	F01	Lost invest	Bad experi	0.4
Map79	F82	F147	Lost invest	Stress and	0.4
Map79	F07	F40	Businesses	Household	0.8
Map79	F07	F47	Businesses	Increase in	0.8
Map79	F07	F85	Businesses	Markets/sh	1
Map79	F07	F87	Businesses	Men stayin	0.8
Map79	F07	F82	Businesses	Lost invest	0.4
Map79	F07	F64	Businesses	Increased p	0.8
Map79	F07	F01	Businesses	Bad experi	0.6
Map79	F07	F96	Businesses	More hung	0.6
Map79	F07	F78	Businesses	Less food t	0.6
Map79	F07	F72	Businesses	Lack of bas	0.8
Map79	F07	F147	Businesses	Stress and	0.4
Map79	F27	F40	Fear of bei	Household	0.4
Map79	F27	F47	Fear of bei	Increase in	0.4
Map79	F27	F81	Fear of bei	Loss of Ha	0.4
Map79	F27	F64	Fear of bei	Increased p	0.4
Map79	F27	F01	Fear of bei	Bad experi	0.6
Map79	F27	F114	Fear of bei	No pilgrima	0.6
Map79	F27	F96	Fear of bei	More hung	0.4

For Peer Review

Map79	F27	F78	Fear of bei	Less food t	0.4
Map79	F27	F72	Fear of bei	Lack of bas	0.4
Map79	F27	F140	Fear of bei	Restriction	0.6
Map79	F27	F147	Fear of bei	Stress and	0.6
Map79	F29	F63	Fear of cor	Increased (	0.6
Map79	F29	F01	Fear of cor	Bad experi	0.6
Map79	F29	F118	Fear of cor	No social g	0.4
Map79	F29	F31	Fear of cor	Fear of eac	0.6
Map79	F29	F38	Fear of cor	Health wor	0.8
Map79	F29	F76	Fear of cor	Less affect	0.4
Map79	F29	F124	Fear of cor	Not visiting	0.6
Map79	F64	F40	Increased p	Household	0.8
Map79	F64	F47	Increased p	Increase in	0.8
Map79	F64	F01	Increased p	Bad experi	0.6
Map79	F64	F96	Increased p	More hung	0.6
Map79	F64	F78	Increased p	Less food t	0.6
Map79	F64	F72	Increased p	Lack of bas	1
Map79	F63	F01	Increased (	Bad experi	1
Map79	F118	F01	No social g	Bad experi	0.2
Map79	F118	F76	No social g	Less affect	0.4
Map79	F114	F40	No pilgrima	Household	0.4
Map79	F114	F47	No pilgrima	Increase in	0.4
Map79	F114	F81	No pilgrima	Loss of Ha	0.4
Map79	F114	F64	No pilgrima	Increased p	0.4
Map79	F114	F01	No pilgrima	Bad experi	0.4
Map79	F114	F96	No pilgrima	More hung	0.4
Map79	F114	F78	No pilgrima	Less food t	0.4
Map79	F114	F72	No pilgrima	Lack of bas	0.4
Map79	F96	F01	More hung	Bad experi	1
Map79	F142	F01	School/ma	Bad experi	0.6
Map79	F142	F16	School/ma	Delay in ec	0.6
Map79	F30	F29	Fear of dea	Fear of cor	1
Map79	F30	F63	Fear of dea	Increased (	0.6
Map79	F30	F01	Fear of dea	Bad experi	0.6
Map79	F30	F118	Fear of dea	No social g	0.4
Map79	F30	F31	Fear of dea	Fear of eac	0.6
Map79	F30	F38	Fear of dea	Health wor	0.8
Map79	F30	F76	Fear of dea	Less affect	0.4
Map79	F30	F124	Fear of dea	Not visiting	0.6
Map79	F31	F01	Fear of eac	Bad experi	0.2
Map79	F31	F118	Fear of eac	No social g	0.4
Map79	F31	F76	Fear of eac	Less affect	0.4
Map79	F78	F01	Less food t	Bad experi	0.6
Map79	F78	F96	Less food t	More hung	0.6
Map79	F38	F63	Health wor	Increased (	0.6
Map79	F38	F01	Health wor	Bad experi	0.6
Map79	F38	F124	Health wor	Not visiting	0.6
Map79	F76	F01	Less affect	Bad experi	0.2
Map79	F16	F01	Delay in ec	Bad experi	0.8
Map79	F72	F40	Lack of bas	Household	0.8
Map79	F72	F47	Lack of bas	Increase in	0.8
Map79	F72	F01	Lack of bas	Bad experi	0.6
Map79	F72	F96	Lack of bas	More hung	0.6
Map79	F72	F78	Lack of bas	Less food t	0.6
Map79	F124	F63	Not visiting	Increased (	0.6
Map79	F124	F01	Not visiting	Bad experi	0.6

For Peer Review

Map79	F140	F40	Restriction	Household	0.4
Map79	F140	F47	Restriction	Increase in	0.4
Map79	F140	F81	Restriction	Loss of Ha	0.4
Map79	F140	F64	Restriction	Increased	0.4
Map79	F140	F01	Restriction	Bad experi	0.8
Map79	F140	F114	Restriction	No pilgrima	0.6
Map79	F140	F96	Restriction	More hung	0.4
Map79	F140	F78	Restriction	Less food t	0.4
Map79	F140	F72	Restriction	Lack of bas	0.4
Map79	F140	F147	Restriction	Stress and	0.8
Map79	F147	F01	Stress and	Bad experi	1
Map79	F120	F40	No transpo	Household	0.4
Map79	F120	F47	No transpo	Increase in	0.4
Map79	F120	F81	No transpo	Loss of Ha	0.4
Map79	F120	F27	No transpo	Fear of bei	0.8
Map79	F120	F64	No transpo	Increased	0.4
Map79	F120	F01	No transpo	Bad experi	0.6
Map79	F120	F114	No transpo	No pilgrima	0.6
Map79	F120	F96	No transpo	More hung	0.4
Map79	F120	F78	No transpo	Less food t	0.4
Map79	F120	F72	No transpo	Lack of bas	0.4
Map79	F120	F140	No transpo	Restriction	0.6
Map79	F120	F147	No transpo	Stress and	0.6
Map79	F106	F01	No congreg	Bad experi	0.8
Map79	F106	F147	No congreg	Stress and	0.8
Map79	F100	F01	Mosques/c	Bad experi	0.6
Map79	F100	F147	Mosques/c	Stress and	0.6
Map79	F100	F106	Mosques/c	No congreg	0.6
Map80	F88	F01	Men unable	Bad experi	1
Map80	F88	F63	Men unable	Increased	0.8
Map80	F88	F96	Men unable	More hung	1
Map80	F85	F01	Markets/sh	Bad experi	0.6
Map80	F85	F64	Markets/sh	Increased	0.8
Map80	F85	F63	Markets/sh	Increased	0.6
Map80	F85	F96	Markets/sh	More hung	0.6
Map80	F85	F112	Markets/sh	No home/p	0.8
Map80	F85	F78	Markets/sh	Less food t	0.6
Map80	F80	F88	Lockdown	Men unable	0.6
Map80	F80	F85	Lockdown	Markets/sh	1
Map80	F80	F01	Lockdown	Bad experi	0.6
Map80	F80	F64	Lockdown	Increased	0.8
Map80	F80	F63	Lockdown	Increased	0.6
Map80	F80	F96	Lockdown	More hung	0.6
Map80	F80	F112	Lockdown	No home/p	0.8
Map80	F80	F78	Lockdown	Less food t	0.6
Map80	F80	F16	Lockdown	Delay in ec	0.6
Map80	F80	F142	Lockdown	School/ma	0.8
Map80	F80	F140	Lockdown	Restriction	0.6
Map80	F80	F147	Lockdown	Stress and	0.6
Map80	F80	F106	Lockdown	No congreg	0.8
Map80	F80	F100	Lockdown	Mosques/c	1
Map80	F20	F01	Difficult bre	Bad experi	0.4
Map80	F29	F01	Fear of cor	Bad experi	0.4
Map80	F29	F118	Fear of cor	No social g	0.6
Map80	F29	F30	Fear of cor	Fear of dea	0.4
Map80	F29	F31	Fear of cor	Fear of eac	0.6

For Peer Review

Map80	F29	F32	Fear of cor	Feeling soc	0.6
Map80	F29	F144	Fear of cor	Social dista	0.6
Map80	F64	F01	Increased p	Bad experi	0.6
Map80	F63	F01	Increased c	Bad experi	0.8
Map80	F154	F20	Wearing fa	Difficult bre	0.2
Map80	F154	F01	Wearing fa	Bad experi	0.2
Map80	F118	F01	No social g	Bad experi	0.4
Map80	F118	F32	No social g	Feeling soc	0.6
Map80	F96	F01	More hung	Bad experi	1
Map80	F96	F63	More hung	Increased c	0.8
Map80	F112	F01	No home/p	Bad experi	0.6
Map80	F112	F64	No home/p	Increased p	0.8
Map80	F112	F63	No home/p	Increased c	0.6
Map80	F112	F96	No home/p	More hung	0.6
Map80	F112	F78	No home/p	Less food t	0.6
Map80	F30	F01	Fear of dea	Bad experi	0.6
Map80	F31	F01	Fear of eac	Bad experi	0.4
Map80	F32	F01	Feeling soc	Bad experi	0.4
Map80	F78	F01	Less food t	Bad experi	0.8
Map80	F78	F63	Less food t	Increased c	0.8
Map80	F78	F96	Less food t	More hung	0.8
Map80	F37	F01	Health facil	Bad experi	0.8
Map80	F37	F63	Health facil	Increased c	0.8
Map80	F37	F14	Health facil	Could not g	0.8
Map80	F16	F01	Delay in ec	Bad experi	0.6
Map80	F16	F147	Delay in ec	Stress and	0.6
Map80	F14	F01	Could not g	Bad experi	0.8
Map80	F14	F63	Could not g	Increased c	1
Map80	F142	F01	School/ma	Bad experi	0.6
Map80	F142	F16	School/ma	Delay in ec	0.6
Map80	F142	F147	School/ma	Stress and	0.6
Map80	F140	F88	Restriction	Men unable	1
Map80	F140	F01	Restriction	Bad experi	1
Map80	F140	F64	Restriction	Increased p	0.6
Map80	F140	F63	Restriction	Increased c	0.8
Map80	F140	F96	Restriction	More hung	1
Map80	F140	F112	Restriction	No home/p	0.6
Map80	F140	F78	Restriction	Less food t	0.6
Map80	F147	F01	Stress and	Bad experi	0.6
Map80	F144	F01	Social dista	Bad experi	0.4
Map80	F144	F118	Social dista	No social g	0.6
Map80	F144	F32	Social dista	Feeling soc	0.6
Map80	F106	F01	No congreg	Bad experi	0.6
Map80	F106	F147	No congreg	Stress and	0.6
Map80	F100	F01	Mosques/c	Bad experi	0.6
Map80	F100	F147	Mosques/c	Stress and	0.6
Map80	F100	F106	Mosques/c	No congreg	0.8
Map81	F40	F47	Household	Increase in	0.4
Map81	F40	F01	Household	Bad experi	0.4
Map81	F47	F01	Increase in	Bad experi	0.8
Map81	F85	F82	Markets/sh	Lost invest	0.6
Map81	F85	F07	Markets/sh	Businesses	0.6
Map81	F85	F01	Markets/sh	Bad experi	0.6
Map81	F85	F64	Markets/sh	Increased p	0.6
Map81	F87	F40	Men stayin	Household	0.4
Map81	F87	F47	Men stayin	Increase in	0.4

For Peer Review

Map81	F87	F01	Men stayin	Bad experi	0.4
Map81	F81	F01	Loss of Ha	Bad experi	0.4
Map81	F81	F62	Loss of Ha	Increased r	0.4
Map81	F80	F40	Lockdown	Household	0.4
Map81	F80	F47	Lockdown	Increase in	0.4
Map81	F80	F85	Lockdown	Markets/sh	0.4
Map81	F80	F87	Lockdown	Men stayin	0.6
Map81	F80	F81	Lockdown	Loss of Ha	0.6
Map81	F80	F82	Lockdown	Lost invest	0.4
Map81	F80	F07	Lockdown	Businesses	0.4
Map81	F80	F01	Lockdown	Bad experi	0.4
Map81	F80	F67	Lockdown	Increased s	0.4
Map81	F80	F64	Lockdown	Increased p	0.4
Map81	F80	F63	Lockdown	Increased c	0.4
Map81	F80	F62	Lockdown	Increased r	0.4
Map81	F80	F155	Lockdown	Wives ran a	0.4
Map81	F80	F158	Lockdown	Youth/child	0.4
Map81	F80	F96	Lockdown	More hung	0.4
Map81	F80	F114	Lockdown	No pilgrima	0.6
Map81	F80	F110	Lockdown	No funerals	0.6
Map81	F80	F135	Lockdown	Price hike	0.4
Map81	F80	F78	Lockdown	Less food t	0.4
Map81	F80	F70	Lockdown	Jobs lost	0.4
Map81	F80	F143	Lockdown	Shortage o	0.4
Map81	F80	F140	Lockdown	Restriction	0.4
Map81	F80	F147	Lockdown	Stress and	0.4
Map81	F80	F120	Lockdown	No transpo	0.4
Map81	F80	F106	Lockdown	No congreg	0.6
Map81	F80	F100	Lockdown	Mosques/c	1
Map81	F82	F01	Lost invest	Bad experi	0.6
Map81	F82	F64	Lost invest	Increased p	0.6
Map81	F07	F82	Businesses	Lost invest	0.8
Map81	F07	F01	Businesses	Bad experi	0.6
Map81	F07	F64	Businesses	Increased p	0.6
Map81	F20	F01	Difficult bre	Bad experi	0.2
Map81	F67	F47	Increased s	Increase in	0.6
Map81	F67	F01	Increased s	Bad experi	0.6
Map81	F29	F20	Fear of cor	Difficult bre	0.4
Map81	F29	F01	Fear of cor	Bad experi	0.2
Map81	F29	F30	Fear of cor	Fear of dea	0.4
Map81	F29	F31	Fear of cor	Fear of eac	0.4
Map81	F29	F10	Fear of cor	Compulsor	0.4
Map81	F64	F01	Increased p	Bad experi	1
Map81	F63	F01	Increased c	Bad experi	0.6
Map81	F62	F01	Increased r	Bad experi	0.8
Map81	F155	F01	Wives ran a	Bad experi	0.6
Map81	F158	F47	Youth/child	Increase in	0.4
Map81	F158	F01	Youth/child	Bad experi	0.4
Map81	F158	F67	Youth/child	Increased s	0.4
Map81	F96	F01	More hung	Bad experi	0.6
Map81	F96	F155	More hung	Wives ran a	0.8
Map81	F114	F81	No pilgrima	Loss of Ha	0.6
Map81	F114	F01	No pilgrima	Bad experi	0.4
Map81	F114	F62	No pilgrima	Increased r	0.4
Map81	F110	F01	No funerals	Bad experi	0.4
Map81	F110	F147	No funerals	Stress and	0.4

For Peer Review

Map81	F135	F01	Price hike	Bad experi	0.6
Map81	F135	F63	Price hike	Increased	0.6
Map81	F135	F155	Price hike	Wives ran a	0.6
Map81	F135	F96	Price hike	More hung	0.6
Map81	F135	F78	Price hike	Less food t	0.6
Map81	F30	F01	Fear of dea	Bad experi	0.2
Map81	F31	F01	Fear of eac	Bad experi	0.2
Map81	F31	F30	Fear of eac	Fear of dea	0.6
Map81	F32	F01	Feeling soc	Bad experi	0.4
Map81	F32	F62	Feeling soc	Increased r	0.4
Map81	F78	F01	Less food t	Bad experi	0.6
Map81	F78	F63	Less food t	Increased	0.6
Map81	F78	F155	Less food t	Wives ran a	0.6
Map81	F78	F96	Less food t	More hung	0.6
Map81	F10	F20	Compulsor	Difficult bre	0.4
Map81	F10	F01	Compulsor	Bad experi	0.2
Map81	F70	F01	Jobs lost	Bad experi	0.6
Map81	F70	F64	Jobs lost	Increased p	0.6
Map81	F143	F01	Shortage o	Bad experi	0.6
Map81	F143	F63	Shortage o	Increased	0.6
Map81	F143	F155	Shortage o	Wives ran a	0.6
Map81	F143	F96	Shortage o	More hung	0.6
Map81	F143	F135	Shortage o	Price hike	0.6
Map81	F143	F78	Shortage o	Less food t	0.6
Map81	F140	F01	Restriction	Bad experi	0.4
Map81	F140	F63	Restriction	Increased	0.4
Map81	F140	F155	Restriction	Wives ran a	0.4
Map81	F140	F96	Restriction	More hung	0.4
Map81	F140	F135	Restriction	Price hike	0.4
Map81	F140	F78	Restriction	Less food t	0.4
Map81	F140	F143	Restriction	Shortage o	0.4
Map81	F140	F120	Restriction	No transpo	0.4
Map81	F147	F01	Stress and	Bad experi	0.6
Map81	F120	F01	No transpo	Bad experi	0.6
Map81	F120	F63	No transpo	Increased	0.6
Map81	F120	F155	No transpo	Wives ran a	0.6
Map81	F120	F96	No transpo	More hung	0.6
Map81	F120	F135	No transpo	Price hike	0.6
Map81	F120	F78	No transpo	Less food t	0.6
Map81	F120	F143	No transpo	Shortage o	0.8
Map81	F144	F01	Social dista	Bad experi	0.4
Map81	F144	F62	Social dista	Increased r	0.4
Map81	F144	F32	Social dista	Feeling soc	0.6
Map81	F144	F147	Social dista	Stress and	0.4
Map81	F144	F106	Social dista	No congreg	0.4
Map81	F106	F01	No congreg	Bad experi	0.4
Map81	F106	F147	No congreg	Stress and	0.4
Map81	F100	F01	Mosques/c	Bad experi	0.4
Map81	F100	F147	Mosques/c	Stress and	0.4
Map81	F100	F106	Mosques/c	No congreg	0.6
Map82	F40	F01	Household	Bad experi	0.4
Map82	F88	F40	Men unable	Household	0.6
Map82	F88	F01	Men unable	Bad experi	0.4
Map82	F88	F96	Men unable	More hung	0.4
Map82	F88	F78	Men unable	Less food t	0.4
Map82	F85	F40	Markets/sh	Household	0.6

For Peer Review

Map82	F85	F88	Markets/sh	Men unable	0.6
Map82	F85	F07	Markets/sh	Businesses	0.8
Map82	F85	F01	Markets/sh	Bad experi	0.4
Map82	F85	F96	Markets/sh	More hung	0.4
Map82	F85	F78	Markets/sh	Less food t	0.4
Map82	F87	F40	Men stayin	Household	0.6
Map82	F87	F88	Men stayin	Men unable	0.6
Map82	F87	F01	Men stayin	Bad experi	0.4
Map82	F87	F96	Men stayin	More hung	0.4
Map82	F87	F78	Men stayin	Less food t	0.4
Map82	F87	F70	Men stayin	Jobs lost	0.6
Map82	F80	F40	Lockdown	Household	0.6
Map82	F80	F88	Lockdown	Men unable	0.6
Map82	F80	F85	Lockdown	Markets/sh	1
Map82	F80	F87	Lockdown	Men stayin	0.6
Map82	F80	F82	Lockdown	Lost invest	0.6
Map82	F80	F05	Lockdown	Banks clos	0.6
Map82	F80	F07	Lockdown	Businesses	0.8
Map82	F80	F01	Lockdown	Bad experi	0.6
Map82	F80	F64	Lockdown	Increased p	0.6
Map82	F80	F62	Lockdown	Increased r	0.4
Map82	F80	F157	Lockdown	Women un	0.2
Map82	F80	F16	Lockdown	Delay in ec	0.6
Map82	F80	F96	Lockdown	More hung	0.4
Map82	F80	F112	Lockdown	No home/p	0.6
Map82	F80	F78	Lockdown	Less food t	0.4
Map82	F80	F133	Lockdown	Poor perfor	0.6
Map82	F80	F13	Lockdown	Could not c	0.6
Map82	F80	F70	Lockdown	Jobs lost	0.6
Map82	F80	F142	Lockdown	School/ma	1
Map82	F80	F140	Lockdown	Restriction	1
Map82	F80	F147	Lockdown	Stress and	0.6
Map82	F80	F120	Lockdown	No transpo	1
Map82	F80	F106	Lockdown	No congreg	0.4
Map82	F80	F104	Lockdown	No buyers	0.6
Map82	F80	F100	Lockdown	Mosques/c	0.4
Map82	F82	F01	Lost invest	Bad experi	0.8
Map82	F82	F64	Lost invest	Increased p	0.8
Map82	F05	F01	Banks clos	Bad experi	0.4
Map82	F05	F62	Banks clos	Increased r	0.4
Map82	F07	F40	Businesses	Household	0.6
Map82	F07	F88	Businesses	Men unable	0.6
Map82	F07	F01	Businesses	Bad experi	0.4
Map82	F07	F96	Businesses	More hung	0.4
Map82	F07	F78	Businesses	Less food t	0.4
Map82	F20	F01	Difficult bre	Bad experi	0.4
Map82	F02	F01	Bad experi	Bad experi	0.4
Map82	F29	F01	Fear of cor	Bad experi	0.6
Map82	F29	F02	Fear of cor	Bad experi	0.6
Map82	F29	F63	Fear of cor	Increased c	0.6
Map82	F29	F118	Fear of cor	No social g	0.6
Map82	F29	F124	Fear of cor	Not visiting	0.6
Map82	F29	F38	Fear of cor	Health wor	0.2
Map82	F29	F14	Fear of cor	Could not g	0.6
Map82	F29	F144	Fear of cor	Social dista	1
Map82	F29	F109	Fear of cor	No financia	0.6

For Peer Review

Map82	F64	F01	Increased p	Bad experi	1
Map82	F63	F01	Increased c	Bad experi	0.8
Map82	F62	F01	Increased r	Bad experi	0.6
Map82	F154	F20	Wearing fa	Difficult bre	0.2
Map82	F154	F01	Wearing fa	Bad experi	0.2
Map82	F157	F01	Women un	Bad experi	0.6
Map82	F157	F147	Women un	Stress and	0.6
Map82	F16	F01	Delay in ed	Bad experi	0.6
Map82	F16	F133	Delay in ed	Poor perfor	0.6
Map82	F16	F147	Delay in ed	Stress and	0.6
Map82	F96	F01	More hung	Bad experi	1
Map82	F118	F01	No social g	Bad experi	0.8
Map82	F118	F109	No social g	No financia	0.8
Map82	F112	F82	No home/p	Lost invest	1
Map82	F112	F01	No home/p	Bad experi	0.8
Map82	F112	F64	No home/p	Increased p	0.8
Map82	F124	F01	Not visiting	Bad experi	0.6
Map82	F124	F02	Not visiting	Bad experi	0.6
Map82	F124	F63	Not visiting	Increased c	0.6
Map82	F124	F14	Not visiting	Could not g	0.6
Map82	F78	F01	Less food t	Bad experi	1
Map82	F78	F96	Less food t	More hung	1
Map82	F133	F01	Poor perfor	Bad experi	0.8
Map82	F133	F147	Poor perfor	Stress and	0.8
Map82	F38	F01	Health wor	Bad experi	0.4
Map82	F38	F63	Health wor	Increased c	0.4
Map82	F38	F14	Health wor	Could not g	0.4
Map82	F13	F01	Could not c	Bad experi	0.4
Map82	F13	F147	Could not c	Stress and	0.4
Map82	F70	F40	Jobs lost	Household	0.6
Map82	F70	F88	Jobs lost	Men unabl	0.8
Map82	F70	F01	Jobs lost	Bad experi	0.4
Map82	F70	F96	Jobs lost	More hung	0.4
Map82	F70	F78	Jobs lost	Less food t	0.4
Map82	F14	F01	Could not g	Bad experi	0.6
Map82	F14	F63	Could not g	Increased c	0.6
Map82	F142	F01	School/ma	Bad experi	0.6
Map82	F142	F16	School/ma	Delay in ed	0.6
Map82	F142	F133	School/ma	Poor perfor	0.6
Map82	F142	F13	School/ma	Could not c	0.6
Map82	F142	F147	School/ma	Stress and	0.6
Map82	F140	F82	Restriction	Lost invest	0.6
Map82	F140	F01	Restriction	Bad experi	0.6
Map82	F140	F64	Restriction	Increased p	0.6
Map82	F140	F112	Restriction	No home/p	0.6
Map82	F140	F104	Restriction	No buyers	0.6
Map82	F147	F01	Stress and	Bad experi	1
Map82	F120	F05	No transpo	Banks clos	0.6
Map82	F120	F01	No transpo	Bad experi	0.4
Map82	F120	F62	No transpo	Increased r	0.4
Map82	F120	F157	No transpo	Women un	0.2
Map82	F120	F147	No transpo	Stress and	0.2
Map82	F144	F01	Social dista	Bad experi	0.6
Map82	F144	F118	Social dista	No social g	0.6
Map82	F144	F109	Social dista	No financia	0.6
Map82	F109	F01	No financia	Bad experi	0.8

For Peer Review

Map82	F106	F01	No congreg	Bad experi	0.6
Map82	F106	F147	No congreg	Stress and	0.6
Map82	F104	F82	No buyers	Lost invest	0.6
Map82	F104	F01	No buyers	Bad experi	0.6
Map82	F104	F64	No buyers	Increased p	0.6
Map82	F104	F112	No buyers	No home/p	0.6
Map82	F100	F01	Mosques/c	Bad experi	0.4
Map82	F100	F147	Mosques/c	Stress and	0.4
Map82	F100	F106	Mosques/c	No congreg	0.4
Map83	F89	F63	Misconcep	Increased c	0.6
Map83	F89	F01	Misconcep	Bad experi	0.4
Map83	F85	F82	Markets/sh	Lost invest	0.6
Map83	F85	F64	Markets/sh	Increased p	0.6
Map83	F85	F01	Markets/sh	Bad experi	0.6
Map83	F85	F96	Markets/sh	More hung	0.6
Map83	F85	F78	Markets/sh	Less food t	0.6
Map83	F81	F01	Loss of Ha	Bad experi	0.8
Map83	F81	F147	Loss of Ha	Stress and	0.8
Map83	F80	F85	Lockdown	Markets/sh	1
Map83	F80	F81	Lockdown	Loss of Ha	0.6
Map83	F80	F82	Lockdown	Lost invest	0.6
Map83	F80	F05	Lockdown	Banks clos	0.2
Map83	F80	F27	Lockdown	Fear of bei	0.2
Map83	F80	F64	Lockdown	Increased p	0.6
Map83	F80	F01	Lockdown	Bad experi	0.6
Map83	F80	F158	Lockdown	Youth/child	0.4
Map83	F80	F138	Lockdown	Restricted	0.6
Map83	F80	F96	Lockdown	More hung	0.6
Map83	F80	F114	Lockdown	No pilgrima	0.6
Map83	F80	F133	Lockdown	Poor perfor	0.4
Map83	F80	F142	Lockdown	School/mad	0.4
Map83	F80	F78	Lockdown	Less food t	0.6
Map83	F80	F115	Lockdown	No preachi	0.4
Map83	F80	F16	Lockdown	Delay in ed	0.4
Map83	F80	F140	Lockdown	Restriction	0.6
Map83	F80	F147	Lockdown	Stress and	0.6
Map83	F80	F120	Lockdown	No transpo	0.6
Map83	F80	F144	Lockdown	Social dista	0.8
Map83	F80	F06	Lockdown	Borders clo	0.6
Map83	F80	F106	Lockdown	No congreg	0.6
Map83	F80	F100	Lockdown	Mosques/c	0.8
Map83	F82	F64	Lost invest	Increased p	0.6
Map83	F82	F01	Lost invest	Bad experi	0.6
Map83	F82	F96	Lost invest	More hung	0.6
Map83	F82	F78	Lost invest	Less food t	0.6
Map83	F05	F64	Banks clos	Increased p	0.4
Map83	F05	F01	Banks clos	Bad experi	0.4
Map83	F05	F96	Banks clos	More hung	0.4
Map83	F05	F78	Banks clos	Less food t	0.4
Map83	F20	F63	Difficult bre	Increased c	0.2
Map83	F20	F01	Difficult bre	Bad experi	0.2
Map83	F27	F01	Fear of bei	Bad experi	0.6
Map83	F27	F147	Fear of bei	Stress and	0.6
Map83	F02	F01	Bad experi	Bad experi	0.6
Map83	F02	F147	Bad experi	Stress and	0.6
Map83	F29	F20	Fear of cor	Difficult bre	0.4

For Peer Review

Map83	F29	F02	Fear of cor	Bad experi	0.6
Map83	F29	F63	Fear of cor	Increased	0.2
Map83	F29	F01	Fear of cor	Bad experi	0.6
Map83	F29	F31	Fear of cor	Fear of eac	0.6
Map83	F29	F10	Fear of cor	Compulsor	0.6
Map83	F29	F124	Fear of cor	Not visiting	0.6
Map83	F29	F147	Fear of cor	Stress and	0.6
Map83	F64	F01	Increased	Bad experi	0.8
Map83	F64	F96	Increased	More hung	0.8
Map83	F64	F78	Increased	Less food t	0.8
Map83	F63	F01	Increased	Bad experi	0.4
Map83	F158	F01	Youth/child	Bad experi	0.4
Map83	F158	F133	Youth/child	Poor perfor	0.4
Map83	F158	F16	Youth/child	Delay in ec	0.4
Map83	F138	F64	Restricted	Increased	0.8
Map83	F138	F01	Restricted	Bad experi	0.8
Map83	F138	F96	Restricted	More hung	0.8
Map83	F138	F78	Restricted	Less food t	0.8
Map83	F96	F01	More hung	Bad experi	0.6
Map83	F114	F81	No pilgrima	Loss of Ha	0.8
Map83	F114	F01	No pilgrima	Bad experi	0.8
Map83	F114	F147	No pilgrima	Stress and	0.8
Map83	F133	F01	Poor perfor	Bad experi	0.4
Map83	F142	F01	School/ma	Bad experi	0.4
Map83	F142	F158	School/ma	Youth/child	0.8
Map83	F142	F133	School/ma	Poor perfor	0.4
Map83	F142	F16	School/ma	Delay in ec	0.4
Map83	F31	F01	Fear of eac	Bad experi	0.6
Map83	F31	F147	Fear of eac	Stress and	0.6
Map83	F78	F01	Less food t	Bad experi	0.6
Map83	F78	F96	Less food t	More hung	0.8
Map83	F115	F27	No preachi	Fear of bei	0.2
Map83	F115	F01	No preachi	Bad experi	0.2
Map83	F115	F147	No preachi	Stress and	0.2
Map83	F10	F20	Compulsor	Difficult bre	0.4
Map83	F10	F63	Compulsor	Increased	0.2
Map83	F10	F01	Compulsor	Bad experi	0.2
Map83	F16	F01	Delay in ec	Bad experi	0.4
Map83	F16	F133	Delay in ec	Poor perfor	0.6
Map83	F124	F02	Not visiting	Bad experi	0.6
Map83	F124	F01	Not visiting	Bad experi	0.6
Map83	F124	F147	Not visiting	Stress and	0.6
Map83	F140	F64	Restriction	Increased	0.6
Map83	F140	F01	Restriction	Bad experi	0.6
Map83	F140	F138	Restriction	Restricted	0.6
Map83	F140	F96	Restriction	More hung	0.6
Map83	F140	F78	Restriction	Less food t	0.6
Map83	F140	F120	Restriction	No transpo	0.6
Map83	F147	F01	Stress and	Bad experi	1
Map83	F120	F64	No transpo	Increased	0.8
Map83	F120	F01	No transpo	Bad experi	0.8
Map83	F120	F138	No transpo	Restricted	0.8
Map83	F120	F96	No transpo	More hung	0.8
Map83	F120	F78	No transpo	Less food t	0.8
Map83	F144	F27	Social dista	Fear of bei	0.2
Map83	F144	F01	Social dista	Bad experi	0.6

For Peer Review

Map83	F144	F115	Social dista	No preachi	0.4
Map83	F144	F147	Social dista	Stress and	0.6
Map83	F144	F106	Social dista	No congreg	0.6
Map83	F144	F100	Social dista	Mosques/c	0.8
Map83	F06	F81	Borders clc	Loss of Ha	0.8
Map83	F06	F01	Borders clc	Bad experi	0.8
Map83	F06	F114	Borders clc	No pilgrima	1
Map83	F06	F147	Borders clc	Stress and	0.8
Map83	F106	F01	No congreg	Bad experi	0.8
Map83	F106	F147	No congreg	Stress and	0.8
Map83	F100	F01	Mosques/c	Bad experi	0.6
Map83	F100	F147	Mosques/c	Stress and	0.6
Map83	F100	F106	Mosques/c	No congreg	0.6
Map84	F85	F87	Markets/sh	Men stayin	0.6
Map84	F85	F83	Markets/sh	Malnutritior	0.6
Map84	F85	F07	Markets/sh	Businesses	0.6
Map84	F85	F01	Markets/sh	Bad experi	0.6
Map84	F85	F64	Markets/sh	Increased p	0.4
Map84	F85	F96	Markets/sh	More hung	0.6
Map84	F85	F135	Markets/sh	Price hike	0.4
Map84	F85	F78	Markets/sh	Less food t	0.6
Map84	F87	F83	Men stayin	Malnutritior	0.6
Map84	F87	F01	Men stayin	Bad experi	0.6
Map84	F87	F96	Men stayin	More hung	0.8
Map84	F87	F78	Men stayin	Less food t	0.8
Map84	F80	F85	Lockdown	Markets/sh	0.8
Map84	F80	F87	Lockdown	Men stayin	0.6
Map84	F80	F83	Lockdown	Malnutritior	0.6
Map84	F80	F07	Lockdown	Businesses	0.6
Map84	F80	F01	Lockdown	Bad experi	0.6
Map84	F80	F67	Lockdown	Increased s	0.4
Map84	F80	F64	Lockdown	Increased p	0.6
Map84	F80	F158	Lockdown	Youth/child	0.6
Map84	F80	F70	Lockdown	Jobs lost	0.6
Map84	F80	F96	Lockdown	More hung	0.6
Map84	F80	F133	Lockdown	Poor perfor	0.8
Map84	F80	F131	Lockdown	Physical pu	0.4
Map84	F80	F135	Lockdown	Price hike	0.4
Map84	F80	F118	Lockdown	No social g	0.4
Map84	F80	F78	Lockdown	Less food t	0.6
Map84	F80	F16	Lockdown	Delay in ec	0.6
Map84	F80	F142	Lockdown	School/ma	0.8
Map84	F80	F140	Lockdown	Restriction	0.6
Map84	F80	F147	Lockdown	Stress and	0.6
Map84	F80	F120	Lockdown	No transpo	0.6
Map84	F80	F144	Lockdown	Social dista	0.6
Map84	F80	F106	Lockdown	No congreg	0.6
Map84	F80	F100	Lockdown	Mosques/c	0.8
Map84	F83	F01	Malnutritior	Bad experi	0.8
Map84	F07	F83	Businesses	Malnutritior	0.4
Map84	F07	F01	Businesses	Bad experi	0.4
Map84	F07	F64	Businesses	Increased p	0.4
Map84	F07	F96	Businesses	More hung	0.4
Map84	F07	F135	Businesses	Price hike	0.4
Map84	F07	F78	Businesses	Less food t	0.4
Map84	F20	F01	Difficult bre	Bad experi	0.4

For Peer Review

Map84	F67	F01	Increased s	Bad experi	0.6
Map84	F67	F131	Increased s	Physical pu	0.6
Map84	F29	F20	Fear of cor	Difficult bre	0.2
Map84	F29	F01	Fear of cor	Bad experi	0.2
Map84	F29	F10	Fear of cor	Compulsor	0.6
Map84	F64	F01	Increased p	Bad experi	0.6
Map84	F158	F01	Youth/child	Bad experi	0.4
Map84	F158	F67	Youth/child	Increased s	0.4
Map84	F158	F131	Youth/child	Physical pu	0.4
Map84	F70	F01	Jobs lost	Bad experi	0.6
Map84	F70	F64	Jobs lost	Increased p	0.8
Map84	F96	F83	More hung	Malnutrition	0.6
Map84	F96	F01	More hung	Bad experi	0.6
Map84	F133	F01	Poor perfor	Bad experi	0.6
Map84	F133	F16	Poor perfor	Delay in ec	0.6
Map84	F131	F01	Physical pu	Bad experi	0.8
Map84	F135	F83	Price hike	Malnutrition	0.6
Map84	F135	F01	Price hike	Bad experi	0.6
Map84	F135	F64	Price hike	Increased p	0.8
Map84	F135	F96	Price hike	More hung	0.6
Map84	F135	F78	Price hike	Less food t	0.6
Map84	F118	F01	No social g	Bad experi	0.6
Map84	F118	F147	No social g	Stress and	0.6
Map84	F78	F83	Less food t	Malnutrition	0.6
Map84	F78	F01	Less food t	Bad experi	0.6
Map84	F78	F96	Less food t	More hung	1
Map84	F10	F20	Compulsor	Difficult bre	0.2
Map84	F10	F01	Compulsor	Bad experi	0.2
Map84	F16	F01	Delay in ec	Bad experi	0.8
Map84	F142	F01	School/ma	Bad experi	0.6
Map84	F142	F67	School/ma	Increased s	0.4
Map84	F142	F158	School/ma	Youth/child	0.6
Map84	F142	F133	School/ma	Poor perfor	0.8
Map84	F142	F131	School/ma	Physical pu	0.4
Map84	F142	F16	School/ma	Delay in ec	0.6
Map84	F141	F20	Rumors on	Difficult bre	0.2
Map84	F141	F01	Rumors on	Bad experi	0.2
Map84	F141	F29	Rumors on	Fear of cor	0.6
Map84	F141	F10	Rumors on	Compulsor	0.6
Map84	F141	F141	Rumors on	Rumors on	0.8
Map84	F140	F01	Restriction	Bad experi	0.6
Map84	F140	F64	Restriction	Increased p	0.6
Map84	F140	F70	Restriction	Jobs lost	0.6
Map84	F140	F120	Restriction	No transpo	0.6
Map84	F147	F01	Stress and	Bad experi	1
Map84	F120	F01	No transpo	Bad experi	0.6
Map84	F120	F64	No transpo	Increased p	0.8
Map84	F120	F70	No transpo	Jobs lost	1
Map84	F144	F01	Social dista	Bad experi	0.8
Map84	F144	F118	Social dista	No social g	0.4
Map84	F144	F147	Social dista	Stress and	0.8
Map84	F144	F106	Social dista	No congreg	0.8
Map84	F106	F01	No congreg	Bad experi	1
Map84	F106	F147	No congreg	Stress and	1
Map84	F100	F01	Mosques/c	Bad experi	0.6
Map84	F100	F118	Mosques/c	No social g	0.4

For Peer Review

Map84	F100	F147	Mosques/c	Stress and	0.6
Map84	F100	F144	Mosques/c	Social dista	0.6
Map84	F100	F106	Mosques/c	No congreg	0.6
Map85	F40	F47	Household	Increase in	1
Map85	F40	F01	Household	Bad experi	1
Map85	F45	F40	Increase in	Household	0.6
Map85	F45	F88	Increase in	Men unable	0.8
Map85	F45	F47	Increase in	Increase in	0.6
Map85	F45	F82	Increase in	Lost invest	1
Map85	F45	F01	Increase in	Bad experi	0.8
Map85	F45	F64	Increase in	Increased p	0.8
Map85	F45	F155	Increase in	Wives ran a	0.8
Map85	F45	F94	Increase in	More hous	0.6
Map85	F45	F96	Increase in	More hung	0.6
Map85	F45	F78	Increase in	Less food t	0.6
Map85	F45	F143	Increase in	Shortage o	0.6
Map85	F45	F147	Increase in	Stress and	0.6
Map85	F45	F109	Increase in	No financia	0.6
Map85	F88	F40	Men unable	Household	0.6
Map85	F88	F47	Men unable	Increase in	0.6
Map85	F88	F01	Men unable	Bad experi	0.6
Map85	F88	F155	Men unable	Wives ran a	0.8
Map85	F88	F94	Men unable	More hous	0.6
Map85	F88	F147	Men unable	Stress and	0.6
Map85	F47	F01	Increase in	Bad experi	1
Map85	F85	F40	Markets/sh	Household	0.6
Map85	F85	F88	Markets/sh	Men unable	0.8
Map85	F85	F47	Markets/sh	Increase in	0.6
Map85	F85	F82	Markets/sh	Lost invest	0.4
Map85	F85	F07	Markets/sh	Businesses	0.8
Map85	F85	F01	Markets/sh	Bad experi	0.8
Map85	F85	F64	Markets/sh	Increased p	0.8
Map85	F85	F155	Markets/sh	Wives ran a	0.8
Map85	F85	F94	Markets/sh	More hous	0.6
Map85	F85	F132	Markets/sh	Poor harve	0.6
Map85	F85	F96	Markets/sh	More hung	0.6
Map85	F85	F78	Markets/sh	Less food t	0.6
Map85	F85	F70	Markets/sh	Jobs lost	0.8
Map85	F85	F143	Markets/sh	Shortage o	0.6
Map85	F85	F147	Markets/sh	Stress and	0.6
Map85	F85	F109	Markets/sh	No financia	0.4
Map85	F85	F101	Markets/sh	No access	0.8
Map85	F80	F40	Lockdown	Household	0.6
Map85	F80	F45	Lockdown	Increase in	0.6
Map85	F80	F88	Lockdown	Men unable	0.8
Map85	F80	F47	Lockdown	Increase in	0.6
Map85	F80	F85	Lockdown	Markets/sh	0.8
Map85	F80	F82	Lockdown	Lost invest	0.6
Map85	F80	F07	Lockdown	Businesses	0.8
Map85	F80	F01	Lockdown	Bad experi	0.8
Map85	F80	F67	Lockdown	Increased s	0.4
Map85	F80	F64	Lockdown	Increased p	0.8
Map85	F80	F153	Lockdown	Veterinary	0.6
Map85	F80	F155	Lockdown	Wives ran a	0.8
Map85	F80	F16	Lockdown	Delay in ec	0.8
Map85	F80	F118	Lockdown	No social g	0.6

For Peer Review

Map85	F80	F94	Lockdown	More hous	0.6
Map85	F80	F132	Lockdown	Poor harve	0.6
Map85	F80	F96	Lockdown	More hung	0.6
Map85	F80	F51	Lockdown	Increase in	0.6
Map85	F80	F58	Lockdown	Increased	0.4
Map85	F80	F78	Lockdown	Less food t	0.6
Map85	F80	F70	Lockdown	Jobs lost	0.8
Map85	F80	F143	Lockdown	Shortage o	0.6
Map85	F80	F142	Lockdown	School/ma	1
Map85	F80	F140	Lockdown	Restriction	0.8
Map85	F80	F147	Lockdown	Stress and	0.6
Map85	F80	F120	Lockdown	No transpo	0.8
Map85	F80	F144	Lockdown	Social dista	0.6
Map85	F80	F109	Lockdown	No financia	0.6
Map85	F80	F106	Lockdown	No congreg	0.6
Map85	F80	F101	Lockdown	No access	0.8
Map85	F80	F100	Lockdown	Mosques/c	1
Map85	F82	F40	Lost invest	Household	0.6
Map85	F82	F88	Lost invest	Men unable	0.8
Map85	F82	F47	Lost invest	Increase in	0.6
Map85	F82	F01	Lost invest	Bad experi	0.8
Map85	F82	F64	Lost invest	Increased	0.8
Map85	F82	F155	Lost invest	Wives ran	0.8
Map85	F82	F94	Lost invest	More hous	0.6
Map85	F82	F96	Lost invest	More hung	0.6
Map85	F82	F78	Lost invest	Less food t	0.6
Map85	F82	F143	Lost invest	Shortage o	0.6
Map85	F82	F147	Lost invest	Stress and	0.6
Map85	F82	F109	Lost invest	No financia	0.6
Map85	F07	F40	Businesses	Household	0.6
Map85	F07	F88	Businesses	Men unable	0.8
Map85	F07	F47	Businesses	Increase in	0.6
Map85	F07	F01	Businesses	Bad experi	0.8
Map85	F07	F64	Businesses	Increased	0.8
Map85	F07	F155	Businesses	Wives ran	0.8
Map85	F07	F94	Businesses	More hous	0.6
Map85	F07	F96	Businesses	More hung	0.6
Map85	F07	F78	Businesses	Less food t	0.6
Map85	F07	F70	Businesses	Jobs lost	0.8
Map85	F07	F143	Businesses	Shortage o	0.6
Map85	F07	F147	Businesses	Stress and	0.6
Map85	F20	F01	Difficult bre	Bad experi	0.4
Map85	F67	F01	Increased s	Bad experi	0.6
Map85	F67	F58	Increased s	Increased	0.6
Map85	F67	F147	Increased s	Stress and	0.6
Map85	F29	F20	Fear of cor	Difficult bre	0.2
Map85	F29	F01	Fear of cor	Bad experi	0.2
Map85	F29	F31	Fear of cor	Fear of eac	0.6
Map85	F29	F10	Fear of cor	Compulsor	0.2
Map85	F64	F40	Increased	Household	0.6
Map85	F64	F88	Increased	Men unable	0.8
Map85	F64	F47	Increased	Increase in	0.6
Map85	F64	F01	Increased	Bad experi	1
Map85	F64	F155	Increased	Wives ran	0.8
Map85	F64	F94	Increased	More hous	0.6
Map85	F64	F96	Increased	More hung	0.6

For Peer Review

Map85	F64	F78	Increased	Less food t	0.6
Map85	F64	F143	Increased	Shortage o	0.6
Map85	F64	F147	Increased	Stress and	0.6
Map85	F63	F01	Increased	Bad experi	1
Map85	F153	F40	Veterinary	Household	0.6
Map85	F153	F45	Veterinary	Increase in	0.8
Map85	F153	F88	Veterinary	Men unabl	0.8
Map85	F153	F47	Veterinary	Increase in	0.6
Map85	F153	F82	Veterinary	Lost invest	0.8
Map85	F153	F01	Veterinary	Bad experi	0.8
Map85	F153	F64	Veterinary	Increased	0.8
Map85	F153	F155	Veterinary	Wives ran	0.8
Map85	F153	F94	Veterinary	More hous	0.6
Map85	F153	F96	Veterinary	More hung	0.6
Map85	F153	F78	Veterinary	Less food t	0.6
Map85	F153	F143	Veterinary	Shortage o	0.6
Map85	F153	F147	Veterinary	Stress and	0.6
Map85	F153	F109	Veterinary	No financia	0.6
Map85	F155	F01	Wives ran	Bad experi	0.6
Map85	F155	F94	Wives ran	More hous	0.6
Map85	F155	F147	Wives ran	Stress and	0.6
Map85	F16	F01	Delay in ed	Bad experi	0.4
Map85	F16	F67	Delay in ed	Increased	0.4
Map85	F16	F51	Delay in ed	Increase in	0.6
Map85	F16	F58	Delay in ed	Increased	0.4
Map85	F16	F147	Delay in ed	Stress and	0.4
Map85	F118	F01	No social g	Bad experi	1
Map85	F118	F147	No social g	Stress and	1
Map85	F94	F01	More hous	Bad experi	0.8
Map85	F94	F147	More hous	Stress and	0.6
Map85	F132	F40	Poor harve	Household	0.4
Map85	F132	F88	Poor harve	Men unabl	0.4
Map85	F132	F47	Poor harve	Increase in	0.4
Map85	F132	F82	Poor harve	Lost invest	0.4
Map85	F132	F01	Poor harve	Bad experi	0.6
Map85	F132	F64	Poor harve	Increased	0.4
Map85	F132	F155	Poor harve	Wives ran	0.4
Map85	F132	F94	Poor harve	More hous	0.4
Map85	F132	F96	Poor harve	More hung	0.6
Map85	F132	F78	Poor harve	Less food t	0.6
Map85	F132	F143	Poor harve	Shortage o	0.8
Map85	F132	F147	Poor harve	Stress and	0.4
Map85	F132	F109	Poor harve	No financia	0.4
Map85	F96	F01	More hung	Bad experi	0.8
Map85	F51	F01	Increase in	Bad experi	0.4
Map85	F51	F67	Increase in	Increased	0.4
Map85	F51	F58	Increase in	Increased	0.4
Map85	F51	F147	Increase in	Stress and	0.4
Map85	F58	F01	Increased	Bad experi	0.8
Map85	F58	F147	Increased	Stress and	0.8
Map85	F31	F20	Fear of eac	Difficult bre	0.2
Map85	F31	F01	Fear of eac	Bad experi	0.2
Map85	F31	F10	Fear of eac	Compulsor	0.2
Map85	F78	F01	Less food t	Bad experi	0.6
Map85	F78	F96	Less food t	More hung	0.6
Map85	F38	F01	Health wor	Bad experi	0.8

For Peer Review

Map85	F38	F63	Health worl	Increased (	0.8
Map85	F10	F20	Compulsor	Difficult bre	0.4
Map85	F10	F01	Compulsor	Bad experi	0.4
Map85	F70	F40	Jobs lost	Household	0.6
Map85	F70	F88	Jobs lost	Men unable	0.8
Map85	F70	F47	Jobs lost	Increase in	0.6
Map85	F70	F01	Jobs lost	Bad experi	1
Map85	F70	F64	Jobs lost	Increased p	1
Map85	F70	F155	Jobs lost	Wives ran a	0.8
Map85	F70	F94	Jobs lost	More house	0.6
Map85	F70	F96	Jobs lost	More hung	0.6
Map85	F70	F78	Jobs lost	Less food t	0.6
Map85	F70	F143	Jobs lost	Shortage o	0.6
Map85	F70	F147	Jobs lost	Stress and	0.6
Map85	F143	F01	Shortage o	Bad experi	0.6
Map85	F143	F96	Shortage o	More hung	0.6
Map85	F143	F78	Shortage o	Less food t	0.6
Map85	F142	F01	School/ma	Bad experi	0.4
Map85	F142	F67	School/ma	Increased s	0.4
Map85	F142	F16	School/ma	Delay in ec	0.8
Map85	F142	F51	School/ma	Increase in	0.6
Map85	F142	F58	School/ma	Increased (	0.4
Map85	F142	F147	School/ma	Stress and	0.4
Map85	F140	F40	Restriction	Household	0.6
Map85	F140	F45	Restriction	Increase in	0.6
Map85	F140	F88	Restriction	Men unable	0.6
Map85	F140	F47	Restriction	Increase in	0.6
Map85	F140	F82	Restriction	Lost invest	0.6
Map85	F140	F01	Restriction	Bad experi	0.6
Map85	F140	F64	Restriction	Increased p	0.6
Map85	F140	F153	Restriction	Veterinary	0.6
Map85	F140	F155	Restriction	Wives ran a	0.6
Map85	F140	F94	Restriction	More house	0.6
Map85	F140	F96	Restriction	More hung	0.6
Map85	F140	F78	Restriction	Less food t	0.6
Map85	F140	F143	Restriction	Shortage o	0.6
Map85	F140	F147	Restriction	Stress and	0.6
Map85	F140	F109	Restriction	No financia	0.6
Map85	F147	F01	Stress and	Bad experi	1
Map85	F120	F40	No transpo	Household	0.6
Map85	F120	F88	No transpo	Men unable	0.8
Map85	F120	F47	No transpo	Increase in	0.6
Map85	F120	F85	No transpo	Markets/sh	1
Map85	F120	F82	No transpo	Lost invest	0.4
Map85	F120	F07	No transpo	Businesses	0.8
Map85	F120	F01	No transpo	Bad experi	0.8
Map85	F120	F64	No transpo	Increased p	0.8
Map85	F120	F155	No transpo	Wives ran a	0.8
Map85	F120	F94	No transpo	More house	0.6
Map85	F120	F132	No transpo	Poor harve	0.6
Map85	F120	F96	No transpo	More hung	0.6
Map85	F120	F78	No transpo	Less food t	0.6
Map85	F120	F70	No transpo	Jobs lost	0.8
Map85	F120	F143	No transpo	Shortage o	0.6
Map85	F120	F147	No transpo	Stress and	0.6
Map85	F120	F109	No transpo	No financia	0.4

For Peer Review

Map85	F120	F101	No transpo	No access	0.8
Map85	F144	F01	Social dista	Bad experi	0.8
Map85	F144	F118	Social dista	No social g	0.8
Map85	F144	F147	Social dista	Stress and	0.8
Map85	F144	F106	Social dista	No congreg	0.8
Map85	F109	F01	No financia	Bad experi	1
Map85	F109	F109	No financia	No financia	0.6
Map85	F106	F01	No congreg	Bad experi	0.8
Map85	F106	F147	No congreg	Stress and	0.8
Map85	F101	F40	No access	Household	0.4
Map85	F101	F88	No access	Men unable	0.4
Map85	F101	F47	No access	Increase in	0.4
Map85	F101	F82	No access	Lost invest	0.4
Map85	F101	F01	No access	Bad experi	0.6
Map85	F101	F64	No access	Increased p	0.4
Map85	F101	F155	No access	Wives ran a	0.4
Map85	F101	F94	No access	More house	0.4
Map85	F101	F132	No access	Poor harve	0.6
Map85	F101	F96	No access	More hung	0.6
Map85	F101	F78	No access	Less food t	0.6
Map85	F101	F143	No access	Shortage o	0.6
Map85	F101	F147	No access	Stress and	0.4
Map85	F101	F109	No access	No financia	0.4
Map85	F100	F01	Mosques/c	Bad experi	0.6
Map85	F100	F118	Mosques/c	No social g	0.6
Map85	F100	F147	Mosques/c	Stress and	0.6
Map85	F100	F144	Mosques/c	Social dista	0.6
Map85	F100	F106	Mosques/c	No congreg	0.6
Map86	F40	F47	Household	Increase in	0.4
Map86	F40	F01	Household	Bad experi	0.4
Map86	F88	F40	Men unable	Household	0.4
Map86	F88	F47	Men unable	Increase in	0.4
Map86	F88	F01	Men unable	Bad experi	0.6
Map86	F88	F63	Men unable	Increased c	0.6
Map86	F88	F96	Men unable	More hung	0.6
Map86	F88	F78	Men unable	Less food t	0.6
Map86	F47	F01	Increase in	Bad experi	0.4
Map86	F85	F07	Markets/sh	Businesses	0.8
Map86	F85	F01	Markets/sh	Bad experi	0.6
Map86	F85	F64	Markets/sh	Increased p	0.8
Map86	F85	F63	Markets/sh	Increased c	0.6
Map86	F85	F96	Markets/sh	More hung	0.8
Map86	F85	F78	Markets/sh	Less food t	0.8
Map86	F87	F40	Men stayin	Household	0.4
Map86	F87	F88	Men stayin	Men unable	0.6
Map86	F87	F47	Men stayin	Increase in	0.4
Map86	F87	F01	Men stayin	Bad experi	0.6
Map86	F87	F63	Men stayin	Increased c	0.6
Map86	F87	F96	Men stayin	More hung	0.6
Map86	F87	F78	Men stayin	Less food t	0.6
Map86	F37	F01	Health facil	Bad experi	0.4
Map86	F37	F63	Health facil	Increased c	0.6
Map86	F37	F14	Health facil	Could not g	0.8
Map86	F81	F01	Loss of Ha	Bad experi	0.6
Map86	F81	F147	Loss of Ha	Stress and	0.6
Map86	F80	F40	Lockdown	Household	0.4

For Peer Review

Map86	F80	F88	Lockdown	Men unable	0.6
Map86	F80	F47	Lockdown	Increase in	0.4
Map86	F80	F85	Lockdown	Markets/sh	1
Map86	F80	F87	Lockdown	Men stayin	0.8
Map86	F80	F81	Lockdown	Loss of Ha	0.6
Map86	F80	F07	Lockdown	Businesses	0.8
Map86	F80	F01	Lockdown	Bad experi	0.6
Map86	F80	F64	Lockdown	Increased p	0.8
Map86	F80	F63	Lockdown	Increased c	0.6
Map86	F80	F158	Lockdown	Youth/child	0.8
Map86	F80	F16	Lockdown	Delay in ec	0.6
Map86	F80	F96	Lockdown	More hung	0.8
Map86	F80	F114	Lockdown	No pilgrima	0.6
Map86	F80	F130	Lockdown	Physical in	0.4
Map86	F80	F58	Lockdown	Increased c	0.6
Map86	F80	F78	Lockdown	Less food t	0.8
Map86	F80	F133	Lockdown	Poor perfor	0.4
Map86	F80	F142	Lockdown	School/mac	1
Map86	F80	F140	Lockdown	Restriction	0.8
Map86	F80	F147	Lockdown	Stress and	0.6
Map86	F80	F120	Lockdown	No transpo	0.8
Map86	F80	F06	Lockdown	Borders clo	1
Map86	F80	F106	Lockdown	No congreg	0.8
Map86	F80	F100	Lockdown	Mosques/c	1
Map86	F07	F01	Businesses	Bad experi	0.6
Map86	F07	F64	Businesses	Increased p	0.8
Map86	F07	F63	Businesses	Increased c	0.6
Map86	F07	F96	Businesses	More hung	0.8
Map86	F07	F78	Businesses	Less food t	0.8
Map86	F20	F01	Difficult bre	Bad experi	0.4
Map86	F64	F01	Increased p	Bad experi	0.6
Map86	F64	F63	Increased p	Increased c	0.6
Map86	F64	F96	Increased p	More hung	0.8
Map86	F64	F78	Increased p	Less food t	1
Map86	F63	F01	Increased c	Bad experi	0.4
Map86	F158	F01	Youth/child	Bad experi	0.4
Map86	F158	F16	Youth/child	Delay in ec	0.6
Map86	F158	F130	Youth/child	Physical in	0.4
Map86	F158	F58	Youth/child	Increased c	0.6
Map86	F158	F133	Youth/child	Poor perfor	0.4
Map86	F158	F147	Youth/child	Stress and	0.4
Map86	F16	F01	Delay in ec	Bad experi	0.4
Map86	F16	F133	Delay in ec	Poor perfor	0.4
Map86	F16	F147	Delay in ec	Stress and	0.4
Map86	F96	F01	More hung	Bad experi	0.6
Map86	F96	F63	More hung	Increased c	0.6
Map86	F114	F81	No pilgrima	Loss of Ha	0.6
Map86	F114	F01	No pilgrima	Bad experi	0.6
Map86	F114	F147	No pilgrima	Stress and	0.6
Map86	F118	F01	No social g	Bad experi	0.4
Map86	F118	F32	No social g	Feeling soc	0.4
Map86	F118	F147	No social g	Stress and	0.4
Map86	F130	F01	Physical in	Bad experi	0.4
Map86	F130	F130	Physical in	Physical in	0.2
Map86	F130	F147	Physical in	Stress and	0.4
Map86	F58	F01	Increased c	Bad experi	0.4

For Peer Review

Map86	F58	F130	Increased (	Physical in	0.4
Map86	F58	F147	Increased (	Stress and	0.4
Map86	F32	F01	Feeling soc	Bad experi	0.4
Map86	F32	F147	Feeling soc	Stress and	0.4
Map86	F78	F01	Less food t	Bad experi	0.6
Map86	F78	F63	Less food t	Increased (	0.6
Map86	F78	F96	Less food t	More hung	0.8
Map86	F133	F01	Poor perfor	Bad experi	0.6
Map86	F133	F147	Poor perfor	Stress and	0.6
Map86	F10	F20	Compulsor	Difficult bre	0.2
Map86	F10	F01	Compulsor	Bad experi	0.2
Map86	F70	F40	Jobs lost	Household	0.4
Map86	F70	F88	Jobs lost	Men unable	0.6
Map86	F70	F47	Jobs lost	Increase in	0.4
Map86	F70	F01	Jobs lost	Bad experi	0.6
Map86	F70	F63	Jobs lost	Increased (	0.6
Map86	F70	F96	Jobs lost	More hung	0.6
Map86	F70	F78	Jobs lost	Less food t	0.6
Map86	F14	F01	Could not g	Bad experi	0.4
Map86	F14	F63	Could not g	Increased (	0.6
Map86	F142	F01	School/ma	Bad experi	0.4
Map86	F142	F158	School/ma	Youth/child	0.8
Map86	F142	F16	School/ma	Delay in ec	0.6
Map86	F142	F130	School/ma	Physical in	0.4
Map86	F142	F58	School/ma	Increased (	0.6
Map86	F142	F133	School/ma	Poor perfor	0.4
Map86	F142	F147	School/ma	Stress and	0.4
Map86	F140	F40	Restriction	Household	0.4
Map86	F140	F88	Restriction	Men unable	0.6
Map86	F140	F47	Restriction	Increase in	0.4
Map86	F140	F87	Restriction	Men stayin	1
Map86	F140	F01	Restriction	Bad experi	0.6
Map86	F140	F63	Restriction	Increased (	0.6
Map86	F140	F96	Restriction	More hung	0.6
Map86	F140	F78	Restriction	Less food t	0.6
Map86	F147	F01	Stress and	Bad experi	0.6
Map86	F144	F01	Social dista	Bad experi	0.4
Map86	F144	F118	Social dista	No social g	0.8
Map86	F144	F32	Social dista	Feeling soc	0.4
Map86	F144	F147	Social dista	Stress and	0.4
Map86	F06	F81	Borders clo	Loss of Ha	0.6
Map86	F06	F01	Borders clo	Bad experi	0.6
Map86	F06	F114	Borders clo	No pilgrima	0.6
Map86	F06	F147	Borders clo	Stress and	0.6
Map86	F106	F01	No congreg	Bad experi	0.6
Map86	F106	F147	No congreg	Stress and	0.6
Map86	F100	F01	Mosques/c	Bad experi	0.6
Map86	F100	F147	Mosques/c	Stress and	0.6
Map86	F100	F106	Mosques/c	No congreg	0.8
Map87	F41	F01	Husbands	Bad experi	0.4
Map87	F41	F95	Husbands	More hous	0.4
Map87	F40	F41	Household	Husbands	0.6
Map87	F40	F47	Household	Increase in	0.6
Map87	F40	F01	Household	Bad experi	0.6
Map87	F40	F95	Household	More hous	0.4
Map87	F42	F01	Immunizati	Bad experi	0.8

For Peer Review

Map87	F42	F63	Immunizati	Increased c	0.8
Map87	F47	F01	Increase in	Bad experi	0.6
Map87	F85	F41	Markets/sh	Husbands	0.6
Map87	F85	F40	Markets/sh	Household	0.6
Map87	F85	F47	Markets/sh	Increase in	0.6
Map87	F85	F82	Markets/sh	Lost invest	0.6
Map87	F85	F07	Markets/sh	Businesses	0.6
Map87	F85	F01	Markets/sh	Bad experi	0.6
Map87	F85	F64	Markets/sh	Increased p	0.6
Map87	F85	F96	Markets/sh	More hung	0.6
Map87	F85	F95	Markets/sh	More hous	0.4
Map87	F85	F135	Markets/sh	Price hike	0.6
Map87	F85	F143	Markets/sh	Shortage o	0.6
Map87	F85	F147	Markets/sh	Stress and	0.6
Map87	F87	F41	Men stayin	Husbands	0.6
Map87	F87	F40	Men stayin	Household	0.6
Map87	F87	F47	Men stayin	Increase in	0.6
Map87	F87	F01	Men stayin	Bad experi	0.6
Map87	F87	F95	Men stayin	More hous	0.4
Map87	F80	F41	Lockdown	Husbands	0.6
Map87	F80	F40	Lockdown	Household	0.6
Map87	F80	F47	Lockdown	Increase in	0.6
Map87	F80	F85	Lockdown	Markets/sh	0.6
Map87	F80	F87	Lockdown	Men stayin	0.8
Map87	F80	F82	Lockdown	Lost invest	0.6
Map87	F80	F07	Lockdown	Businesses	0.6
Map87	F80	F01	Lockdown	Bad experi	0.8
Map87	F80	F64	Lockdown	Increased p	0.6
Map87	F80	F158	Lockdown	Youth/child	0.6
Map87	F80	F96	Lockdown	More hung	0.6
Map87	F80	F95	Lockdown	More hous	0.4
Map87	F80	F130	Lockdown	Physical in	0.4
Map87	F80	F135	Lockdown	Price hike	0.6
Map87	F80	F133	Lockdown	Poor perfo	0.6
Map87	F80	F13	Lockdown	Could not c	0.4
Map87	F80	F16	Lockdown	Delay in ed	0.6
Map87	F80	F143	Lockdown	Shortage o	0.6
Map87	F80	F142	Lockdown	School/ma	0.6
Map87	F80	F147	Lockdown	Stress and	0.8
Map87	F80	F115	Lockdown	No preachi	0.8
Map87	F80	F100	Lockdown	Mosques/c	1
Map87	F82	F41	Lost invest	Husbands	0.6
Map87	F82	F40	Lost invest	Household	0.6
Map87	F82	F47	Lost invest	Increase in	0.6
Map87	F82	F01	Lost invest	Bad experi	0.8
Map87	F82	F64	Lost invest	Increased p	0.8
Map87	F82	F96	Lost invest	More hung	0.8
Map87	F82	F95	Lost invest	More hous	0.4
Map87	F07	F41	Businesses	Husbands	0.6
Map87	F07	F40	Businesses	Household	0.6
Map87	F07	F47	Businesses	Increase in	0.6
Map87	F07	F82	Businesses	Lost invest	0.8
Map87	F07	F01	Businesses	Bad experi	0.8
Map87	F07	F64	Businesses	Increased p	0.8
Map87	F07	F96	Businesses	More hung	0.8
Map87	F07	F95	Businesses	More hous	0.4

Map87	F20	F01	Difficult bre	Bad experi	0.4
Map87	F67	F01	Increased s	Bad experi	1
Map87	F67	F158	Increased s	Youth/child	0.8
Map87	F67	F130	Increased s	Physical in	0.4
Map87	F67	F13	Increased s	Could not c	0.4
Map87	F29	F20	Fear of cor	Difficult bre	0.2
Map87	F29	F01	Fear of cor	Bad experi	0.6
Map87	F29	F31	Fear of cor	Fear of eac	0.6
Map87	F29	F10	Fear of cor	Compulsor	0.6
Map87	F64	F41	Increased p	Husbands	0.6
Map87	F64	F40	Increased p	Household	0.6
Map87	F64	F47	Increased p	Increase in	0.6
Map87	F64	F01	Increased p	Bad experi	0.8
Map87	F64	F96	Increased p	More hung	0.8
Map87	F64	F95	Increased p	More hous	0.4
Map87	F63	F01	Increased c	Bad experi	0.8
Map87	F62	F01	Increased r	Bad experi	0.6
Map87	F158	F01	Youth/child	Bad experi	0.4
Map87	F158	F130	Youth/child	Physical in	0.4
Map87	F158	F13	Youth/child	Could not c	0.4
Map87	F96	F41	More hung	Husbands	0.6
Map87	F96	F40	More hung	Household	0.6
Map87	F96	F47	More hung	Increase in	0.6
Map87	F96	F01	More hung	Bad experi	0.8
Map87	F96	F95	More hung	More hous	0.4
Map87	F95	F01	More hous	Bad experi	0.4
Map87	F118	F01	No social g	Bad experi	0.6
Map87	F118	F62	No social g	Increased r	0.6
Map87	F130	F01	Physical in	Bad experi	0.6
Map87	F135	F01	Price hike	Bad experi	0.8
Map87	F31	F01	Fear of eac	Bad experi	0.6
Map87	F133	F01	Poor perfor	Bad experi	0.8
Map87	F13	F01	Could not c	Bad experi	0.4
Map87	F13	F130	Could not c	Physical in	0.4
Map87	F10	F20	Compulsor	Difficult bre	0.2
Map87	F10	F01	Compulsor	Bad experi	0.2
Map87	F16	F01	Delay in ec	Bad experi	0.6
Map87	F16	F133	Delay in ec	Poor perfor	0.6
Map87	F73	F01	Lack of del	Bad experi	0.8
Map87	F73	F63	Lack of del	Increased c	0.8
Map87	F143	F01	Shortage o	Bad experi	0.8
Map87	F143	F135	Shortage o	Price hike	0.8
Map87	F143	F147	Shortage o	Stress and	0.6
Map87	F142	F01	School/ma	Bad experi	0.6
Map87	F142	F158	School/ma	Youth/child	0.6
Map87	F142	F130	School/ma	Physical in	0.4
Map87	F142	F133	School/ma	Poor perfor	0.6
Map87	F142	F13	School/ma	Could not c	0.4
Map87	F142	F16	School/ma	Delay in ec	0.8
Map87	F147	F01	Stress and	Bad experi	0.8
Map87	F120	F42	No transpo	Immunizati	0.6
Map87	F120	F01	No transpo	Bad experi	0.6
Map87	F120	F63	No transpo	Increased c	0.6
Map87	F120	F73	No transpo	Lack of del	0.6
Map87	F144	F01	Social dista	Bad experi	0.6
Map87	F144	F62	Social dista	Increased r	0.6

For Peer Review

Map87	F144	F118	Social dista	No social g	0.6
Map87	F115	F01	No preachi	Bad experi	0.8
Map87	F115	F147	No preachi	Stress and	0.8
Map87	F100	F01	Mosques/c	Bad experi	0.8
Map87	F100	F147	Mosques/c	Stress and	0.8
Map87	F100	F115	Mosques/c	No preachi	0.8
Map88	F85	F82	Markets/sh	Lost invest	0.6
Map88	F85	F07	Markets/sh	Businesses	0.6
Map88	F85	F01	Markets/sh	Bad experi	0.6
Map88	F85	F64	Markets/sh	Increased p	0.6
Map88	F85	F70	Markets/sh	Jobs lost	0.8
Map88	F85	F96	Markets/sh	More hung	0.6
Map88	F85	F135	Markets/sh	Price hike	0.6
Map88	F85	F78	Markets/sh	Less food t	0.6
Map88	F85	F143	Markets/sh	Shortage o	0.8
Map88	F80	F85	Lockdown	Markets/sh	0.8
Map88	F80	F82	Lockdown	Lost invest	0.6
Map88	F80	F07	Lockdown	Businesses	0.6
Map88	F80	F01	Lockdown	Bad experi	0.6
Map88	F80	F67	Lockdown	Increased s	0.6
Map88	F80	F64	Lockdown	Increased p	0.6
Map88	F80	F63	Lockdown	Increased c	0.6
Map88	F80	F158	Lockdown	Youth/child	0.6
Map88	F80	F70	Lockdown	Jobs lost	0.8
Map88	F80	F96	Lockdown	More hung	0.6
Map88	F80	F133	Lockdown	Poor perfor	0.8
Map88	F80	F131	Lockdown	Physical pu	0.6
Map88	F80	F135	Lockdown	Price hike	0.6
Map88	F80	F142	Lockdown	School/ma	0.8
Map88	F80	F118	Lockdown	No social g	0.6
Map88	F80	F78	Lockdown	Less food t	0.6
Map88	F80	F16	Lockdown	Delay in ec	0.6
Map88	F80	F143	Lockdown	Shortage o	0.8
Map88	F80	F124	Lockdown	Not visiting	0.6
Map88	F80	F140	Lockdown	Restriction	0.8
Map88	F80	F147	Lockdown	Stress and	0.6
Map88	F80	F120	Lockdown	No transpo	0.6
Map88	F80	F144	Lockdown	Social dista	0.6
Map88	F80	F100	Lockdown	Mosques/c	0.6
Map88	F82	F01	Lost invest	Bad experi	0.8
Map88	F82	F64	Lost invest	Increased p	0.8
Map88	F07	F82	Businesses	Lost invest	0.6
Map88	F07	F01	Businesses	Bad experi	0.6
Map88	F07	F64	Businesses	Increased p	0.6
Map88	F07	F70	Businesses	Jobs lost	0.8
Map88	F20	F01	Difficult bre	Bad experi	0.6
Map88	F67	F01	Increased s	Bad experi	0.6
Map88	F67	F131	Increased s	Physical pu	0.8
Map88	F29	F20	Fear of cor	Difficult bre	0.2
Map88	F29	F01	Fear of cor	Bad experi	0.2
Map88	F29	F154	Fear of cor	Wearing fa	0.4
Map88	F64	F01	Increased p	Bad experi	1
Map88	F63	F01	Increased c	Bad experi	0.8
Map88	F154	F20	Wearing fa	Difficult bre	0.2
Map88	F154	F01	Wearing fa	Bad experi	0.2
Map88	F158	F01	Youth/child	Bad experi	0.6

For Peer Review

Map88	F158	F67	Youth/child	Increased s	0.8
Map88	F158	F131	Youth/child	Physical pu	0.8
Map88	F70	F01	Jobs lost	Bad experi	0.6
Map88	F70	F64	Jobs lost	Increased p	0.6
Map88	F96	F01	More hung	Bad experi	1
Map88	F133	F01	Poor perfor	Bad experi	0.6
Map88	F133	F16	Poor perfor	Delay in ec	0.6
Map88	F131	F01	Physical pu	Bad experi	0.6
Map88	F135	F01	Price hike	Bad experi	0.6
Map88	F135	F96	Price hike	More hung	0.6
Map88	F135	F78	Price hike	Less food t	0.6
Map88	F142	F01	School/ma	Bad experi	0.6
Map88	F142	F67	School/ma	Increased s	0.6
Map88	F142	F158	School/ma	Youth/child	0.6
Map88	F142	F133	School/ma	Poor perfor	0.8
Map88	F142	F131	School/ma	Physical pu	0.6
Map88	F142	F16	School/ma	Delay in ec	0.6
Map88	F118	F01	No social g	Bad experi	0.6
Map88	F118	F147	No social g	Stress and	0.6
Map88	F78	F01	Less food t	Bad experi	0.8
Map88	F78	F96	Less food t	More hung	0.8
Map88	F16	F01	Delay in ec	Bad experi	0.8
Map88	F143	F01	Shortage o	Bad experi	0.6
Map88	F143	F96	Shortage o	More hung	0.6
Map88	F143	F135	Shortage o	Price hike	0.6
Map88	F143	F78	Shortage o	Less food t	0.6
Map88	F124	F01	Not visiting	Bad experi	0.8
Map88	F124	F63	Not visiting	Increased c	0.8
Map88	F140	F01	Restriction	Bad experi	0.6
Map88	F140	F63	Restriction	Increased c	0.6
Map88	F140	F124	Restriction	Not visiting	0.6
Map88	F140	F120	Restriction	No transpo	0.6
Map88	F147	F01	Stress and	Bad experi	0.8
Map88	F120	F01	No transpo	Bad experi	0.8
Map88	F120	F63	No transpo	Increased c	0.8
Map88	F120	F124	No transpo	Not visiting	0.8
Map88	F144	F01	Social dista	Bad experi	0.6
Map88	F144	F118	Social dista	No social g	0.6
Map88	F144	F147	Social dista	Stress and	0.6
Map88	F144	F100	Social dista	Mosques/c	0.8
Map88	F106	F01	No congreg	Bad experi	0.8
Map88	F106	F147	No congreg	Stress and	0.8
Map88	F106	F100	No congreg	Mosques/c	1
Map89	F40	F47	Household	Increase in	0.4
Map89	F40	F01	Household	Bad experi	0.4
Map89	F88	F40	Men unable	Household	0.6
Map89	F88	F47	Men unable	Increase in	0.4
Map89	F88	F01	Men unable	Bad experi	0.4
Map89	F47	F01	Increase in	Bad experi	0.4
Map89	F85	F82	Markets/sh	Lost invest	0.6
Map89	F85	F07	Markets/sh	Businesses	0.8
Map89	F85	F01	Markets/sh	Bad experi	0.6
Map89	F85	F64	Markets/sh	Increased p	0.6
Map89	F85	F78	Markets/sh	Less food t	0.6
Map89	F87	F40	Men stayin	Household	0.6
Map89	F87	F88	Men stayin	Men unable	0.8

For Peer Review

Map89	F87	F47	Men stayin	Increase in	0.4
Map89	F87	F01	Men stayin	Bad experi	0.4
Map89	F87	F63	Men stayin	Increased	0.4
Map89	F87	F98	Men stayin	More mate	0.4
Map89	F87	F99	Men stayin	More unwa	0.4
Map89	F80	F40	Lockdown	Household	0.6
Map89	F80	F88	Lockdown	Men unable	0.8
Map89	F80	F47	Lockdown	Increase in	0.4
Map89	F80	F87	Lockdown	Men stayin	0.8
Map89	F80	F01	Lockdown	Bad experi	0.4
Map89	F80	F63	Lockdown	Increased	0.4
Map89	F80	F158	Lockdown	Youth/child	0.8
Map89	F80	F95	Lockdown	More hous	0.6
Map89	F80	F98	Lockdown	More mate	0.4
Map89	F80	F99	Lockdown	More unwa	0.4
Map89	F80	F13	Lockdown	Could not	0.6
Map89	F80	F142	Lockdown	School/ma	1
Map89	F80	F147	Lockdown	Stress and	0.6
Map89	F80	F106	Lockdown	No congreg	0.6
Map89	F80	F100	Lockdown	Mosques/c	1
Map89	F82	F01	Lost invest	Bad experi	0.6
Map89	F82	F64	Lost invest	Increased	0.6
Map89	F82	F78	Lost invest	Less food t	0.6
Map89	F07	F82	Businesses	Lost invest	0.6
Map89	F07	F01	Businesses	Bad experi	0.6
Map89	F07	F64	Businesses	Increased	0.6
Map89	F07	F78	Businesses	Less food t	0.6
Map89	F20	F01	Difficult bre	Bad experi	0.2
Map89	F29	F01	Fear of cor	Bad experi	0.4
Map89	F29	F118	Fear of cor	No social g	0.4
Map89	F29	F30	Fear of cor	Fear of dea	0.6
Map89	F29	F31	Fear of cor	Fear of eac	0.4
Map89	F29	F147	Fear of cor	Stress and	0.2
Map89	F29	F144	Fear of cor	Social dista	0.4
Map89	F64	F01	Increased	Bad experi	0.6
Map89	F64	F78	Increased	Less food t	0.8
Map89	F63	F01	Increased	Bad experi	0.4
Map89	F158	F01	Youth/child	Bad experi	0.4
Map89	F158	F95	Youth/child	More hous	0.6
Map89	F158	F13	Youth/child	Could not	0.6
Map89	F158	F147	Youth/child	Stress and	0.6
Map89	F118	F01	No social g	Bad experi	0.2
Map89	F118	F147	No social g	Stress and	0.2
Map89	F95	F01	More hous	Bad experi	0.4
Map89	F95	F147	More hous	Stress and	0.4
Map89	F98	F01	More mate	Bad experi	0.4
Map89	F98	F63	More mate	Increased	0.8
Map89	F99	F01	More unwa	Bad experi	0.4
Map89	F99	F63	More unwa	Increased	0.6
Map89	F99	F98	More unwa	More mate	0.6
Map89	F30	F01	Fear of dea	Bad experi	0.4
Map89	F31	F01	Fear of eac	Bad experi	0.2
Map89	F31	F118	Fear of eac	No social g	0.6
Map89	F31	F147	Fear of eac	Stress and	0.2
Map89	F31	F144	Fear of eac	Social dista	0.6
Map89	F78	F01	Less food t	Bad experi	0.6

For Peer Review

Map89	F37	F01	Health facil	Bad experi	0.4
Map89	F37	F63	Health facil	Increased c	0.6
Map89	F37	F14	Health facil	Could not g	0.8
Map89	F13	F01	Could not c	Bad experi	0.4
Map89	F13	F147	Could not c	Stress and	0.6
Map89	F10	F20	Compulsor	Difficult bre	0.4
Map89	F10	F01	Compulsor	Bad experi	0.2
Map89	F14	F01	Could not g	Bad experi	0.4
Map89	F14	F63	Could not g	Increased c	0.6
Map89	F142	F01	School/ma	Bad experi	0.4
Map89	F142	F158	School/ma	Youth/child	0.8
Map89	F142	F95	School/ma	More hous	0.6
Map89	F142	F13	School/ma	Could not c	0.6
Map89	F142	F147	School/ma	Stress and	0.6
Map89	F147	F01	Stress and	Bad experi	0.4
Map89	F144	F01	Social dista	Bad experi	0.2
Map89	F144	F118	Social dista	No social g	0.6
Map89	F144	F147	Social dista	Stress and	0.2
Map89	F106	F01	No congreg	Bad experi	0.2
Map89	F100	F01	Mosques/c	Bad experi	0.2
Map89	F100	F106	Mosques/c	No congreg	0.6
Map90	F40	F47	Household	Increase in	0.4
Map90	F40	F01	Household	Bad experi	0.4
Map90	F88	F40	Men unable	Household	0.2
Map90	F88	F47	Men unable	Increase in	0.2
Map90	F88	F01	Men unable	Bad experi	0.8
Map90	F88	F63	Men unable	Increased c	0.8
Map90	F88	F96	Men unable	More hung	0.8
Map90	F47	F01	Increase in	Bad experi	0.4
Map90	F85	F82	Markets/sh	Lost invest	0.6
Map90	F85	F07	Markets/sh	Businesses	0.8
Map90	F85	F01	Markets/sh	Bad experi	0.4
Map90	F85	F135	Markets/sh	Price hike	0.8
Map90	F87	F40	Men stayin	Household	0.2
Map90	F87	F88	Men stayin	Men unable	0.8
Map90	F87	F47	Men stayin	Increase in	0.2
Map90	F87	F01	Men stayin	Bad experi	0.8
Map90	F87	F63	Men stayin	Increased c	0.8
Map90	F87	F96	Men stayin	More hung	0.8
Map90	F87	F98	Men stayin	More mate	0.2
Map90	F87	F99	Men stayin	More unwa	0.2
Map90	F87	F36	Men stayin	Frequent s	0.6
Map90	F80	F40	Lockdown	Household	0.2
Map90	F80	F88	Lockdown	Men unable	0.4
Map90	F80	F47	Lockdown	Increase in	0.2
Map90	F80	F85	Lockdown	Markets/sh	0.8
Map90	F80	F87	Lockdown	Men stayin	0.4
Map90	F80	F82	Lockdown	Lost invest	0.6
Map90	F80	F07	Lockdown	Businesses	0.8
Map90	F80	F01	Lockdown	Bad experi	0.6
Map90	F80	F63	Lockdown	Increased c	0.4
Map90	F80	F158	Lockdown	Youth/child	1
Map90	F80	F70	Lockdown	Jobs lost	0.6
Map90	F80	F96	Lockdown	More hung	0.4
Map90	F80	F133	Lockdown	Poor perfor	0.4
Map90	F80	F98	Lockdown	More mate	0.2

For Peer Review

Map90	F80	F99	Lockdown	More unwa	0.2
Map90	F80	F135	Lockdown	Price hike	0.8
Map90	F80	F118	Lockdown	No social g	0.4
Map90	F80	F36	Lockdown	Frequent s	0.4
Map90	F80	F13	Lockdown	Could not	0.4
Map90	F80	F16	Lockdown	Delay in ec	0.8
Map90	F80	F142	Lockdown	School/ma	1
Map90	F80	F140	Lockdown	Restriction	0.6
Map90	F80	F147	Lockdown	Stress and	0.6
Map90	F80	F120	Lockdown	No transpo	0.8
Map90	F80	F100	Lockdown	Mosques/c	0.6
Map90	F82	F01	Lost invest	Bad experi	0.4
Map90	F07	F82	Businesses	Lost invest	0.6
Map90	F07	F01	Businesses	Bad experi	0.4
Map90	F29	F01	Fear of cor	Bad experi	0.4
Map90	F29	F30	Fear of cor	Fear of dea	0.6
Map90	F29	F31	Fear of cor	Fear of eac	0.8
Map90	F63	F01	Increased	Bad experi	0.6
Map90	F158	F01	Youth/child	Bad experi	0.4
Map90	F158	F133	Youth/child	Poor perfor	0.4
Map90	F158	F13	Youth/child	Could not	0.4
Map90	F158	F16	Youth/child	Delay in ec	0.8
Map90	F158	F147	Youth/child	Stress and	0.4
Map90	F70	F40	Jobs lost	Household	0.2
Map90	F70	F88	Jobs lost	Men unabl	0.4
Map90	F70	F47	Jobs lost	Increase in	0.2
Map90	F70	F87	Jobs lost	Men stayin	0.4
Map90	F70	F01	Jobs lost	Bad experi	0.4
Map90	F70	F63	Jobs lost	Increased	0.4
Map90	F70	F96	Jobs lost	More hung	0.4
Map90	F70	F98	Jobs lost	More mate	0.2
Map90	F70	F99	Jobs lost	More unwa	0.2
Map90	F70	F36	Jobs lost	Frequent s	0.4
Map90	F96	F01	More hung	Bad experi	1
Map90	F96	F63	More hung	Increased	0.8
Map90	F133	F01	Poor perfor	Bad experi	0.4
Map90	F133	F147	Poor perfor	Stress and	0.4
Map90	F98	F01	More mate	Bad experi	0.6
Map90	F99	F01	More unwa	Bad experi	0.4
Map90	F99	F98	More unwa	More mate	0.4
Map90	F135	F82	Price hike	Lost invest	0.6
Map90	F135	F01	Price hike	Bad experi	0.4
Map90	F30	F01	Fear of dea	Bad experi	0.4
Map90	F31	F01	Fear of eac	Bad experi	0.4
Map90	F31	F30	Fear of eac	Fear of dea	0.6
Map90	F118	F01	No social g	Bad experi	0.6
Map90	F118	F147	No social g	Stress and	0.4
Map90	F36	F01	Frequent s	Bad experi	0.2
Map90	F36	F98	Frequent s	More mate	0.2
Map90	F36	F99	Frequent s	More unwa	0.2
Map90	F37	F01	Health facil	Bad experi	0.4
Map90	F37	F63	Health facil	Increased	0.4
Map90	F37	F38	Health facil	Health wor	0.6
Map90	F37	F14	Health facil	Could not	0.6
Map90	F38	F01	Health wor	Bad experi	0.4
Map90	F38	F63	Health wor	Increased	0.4

For Peer Review

Map90	F38	F14	Health wor	Could not g	0.6
Map90	F13	F01	Could not c	Bad experi	0.8
Map90	F13	F147	Could not c	Stress and	0.8
Map90	F16	F01	Delay in ec	Bad experi	0.4
Map90	F16	F133	Delay in ec	Poor perfor	0.4
Map90	F16	F147	Delay in ec	Stress and	0.4
Map90	F14	F01	Could not g	Bad experi	0.4
Map90	F14	F63	Could not g	Increased c	0.4
Map90	F142	F01	School/ma	Bad experi	0.4
Map90	F142	F158	School/ma	Youth/child	1
Map90	F142	F133	School/ma	Poor perfor	0.4
Map90	F142	F13	School/ma	Could not c	0.4
Map90	F142	F16	School/ma	Delay in ec	0.8
Map90	F142	F147	School/ma	Stress and	0.4
Map90	F140	F01	Restriction	Bad experi	0.4
Map90	F140	F118	Restriction	No social g	0.4
Map90	F140	F147	Restriction	Stress and	0.4
Map90	F147	F01	Stress and	Bad experi	0.8
Map90	F120	F40	No transpo	Household	0.2
Map90	F120	F88	No transpo	Men unable	0.4
Map90	F120	F47	No transpo	Increase in	0.2
Map90	F120	F87	No transpo	Men stayin	0.4
Map90	F120	F01	No transpo	Bad experi	0.4
Map90	F120	F63	No transpo	Increased c	0.4
Map90	F120	F70	No transpo	Jobs lost	0.6
Map90	F120	F96	No transpo	More hung	0.4
Map90	F120	F98	No transpo	More mate	0.2
Map90	F120	F99	No transpo	More unwa	0.2
Map90	F120	F36	No transpo	Frequent s	0.4
Map90	F100	F01	Mosques/c	Bad experi	0.8
Map90	F100	F147	Mosques/c	Stress and	0.8
Map91	F40	F47	Household	Increase in	0.6
Map91	F40	F01	Household	Bad experi	0.6
Map91	F88	F40	Men unable	Household	0.6
Map91	F88	F47	Men unable	Increase in	0.6
Map91	F88	F01	Men unable	Bad experi	0.6
Map91	F47	F01	Increase in	Bad experi	0.8
Map91	F85	F83	Markets/sh	Malnutrition	0.6
Map91	F85	F01	Markets/sh	Bad experi	0.8
Map91	F85	F63	Markets/sh	Increased c	0.8
Map91	F85	F96	Markets/sh	More hung	0.8
Map91	F85	F135	Markets/sh	Price hike	1
Map91	F85	F78	Markets/sh	Less food t	0.8
Map91	F85	F143	Markets/sh	Shortage o	1
Map91	F87	F40	Men stayin	Household	0.6
Map91	F87	F88	Men stayin	Men unable	0.6
Map91	F87	F47	Men stayin	Increase in	0.6
Map91	F87	F01	Men stayin	Bad experi	0.6
Map91	F80	F40	Lockdown	Household	0.6
Map91	F80	F88	Lockdown	Men unable	0.8
Map91	F80	F47	Lockdown	Increase in	0.6
Map91	F80	F85	Lockdown	Markets/sh	1
Map91	F80	F87	Lockdown	Men stayin	0.6
Map91	F80	F83	Lockdown	Malnutrition	0.6
Map91	F80	F01	Lockdown	Bad experi	1
Map91	F80	F64	Lockdown	Increased p	1

For Peer Review

Map91	F80	F63	Lockdown	Increased (	0.8
Map91	F80	F158	Lockdown	Youth/child	0.8
Map91	F80	F118	Lockdown	No social g	0.6
Map91	F80	F132	Lockdown	Poor harve	0.8
Map91	F80	F96	Lockdown	More hung	0.8
Map91	F80	F130	Lockdown	Physical in	0.6
Map91	F80	F135	Lockdown	Price hike	1
Map91	F80	F142	Lockdown	School/ma	1
Map91	F80	F78	Lockdown	Less food t	0.8
Map91	F80	F79	Lockdown	LGA secret	0.4
Map91	F80	F70	Lockdown	Jobs lost	1
Map91	F80	F143	Lockdown	Shortage o	1
Map91	F80	F147	Lockdown	Stress and	0.6
Map91	F80	F120	Lockdown	No transpo	1
Map91	F80	F123	Lockdown	Not going t	0.8
Map91	F80	F106	Lockdown	No congreg	0.6
Map91	F80	F100	Lockdown	Mosques/c	0.8
Map91	F83	F01	Malnutritior	Bad experi	0.6
Map91	F20	F01	Difficult bre	Bad experi	0.4
Map91	F29	F01	Fear of cor	Bad experi	0.6
Map91	F29	F31	Fear of cor	Fear of eac	0.6
Map91	F64	F40	Increased	Household	0.6
Map91	F64	F88	Increased	Men unable	0.8
Map91	F64	F47	Increased	Increase in	0.6
Map91	F64	F01	Increased	Bad experi	1
Map91	F63	F01	Increased (	Bad experi	1
Map91	F158	F01	Youth/child	Bad experi	0.6
Map91	F158	F130	Youth/child	Physical in	0.6
Map91	F158	F147	Youth/child	Stress and	0.6
Map91	F118	F01	No social g	Bad experi	0.8
Map91	F118	F147	No social g	Stress and	0.8
Map91	F118	F106	No social g	No congreg	0.8
Map91	F132	F83	Poor harve	Malnutritior	0.6
Map91	F132	F01	Poor harve	Bad experi	0.8
Map91	F132	F63	Poor harve	Increased (	0.8
Map91	F132	F96	Poor harve	More hung	0.8
Map91	F132	F135	Poor harve	Price hike	0.8
Map91	F132	F78	Poor harve	Less food t	0.8
Map91	F132	F143	Poor harve	Shortage o	0.6
Map91	F96	F01	More hung	Bad experi	1
Map91	F96	F63	More hung	Increased (	0.8
Map91	F130	F01	Physical in	Bad experi	0.6
Map91	F130	F147	Physical in	Stress and	0.6
Map91	F135	F83	Price hike	Malnutritior	0.6
Map91	F135	F01	Price hike	Bad experi	0.8
Map91	F135	F63	Price hike	Increased (	0.8
Map91	F135	F96	Price hike	More hung	0.8
Map91	F135	F78	Price hike	Less food t	0.8
Map91	F142	F01	School/ma	Bad experi	0.6
Map91	F142	F158	School/ma	Youth/child	0.8
Map91	F142	F130	School/ma	Physical in	0.6
Map91	F142	F147	School/ma	Stress and	0.6
Map91	F31	F01	Fear of eac	Bad experi	0.8
Map91	F78	F83	Less food t	Malnutritior	0.6
Map91	F78	F01	Less food t	Bad experi	0.8
Map91	F78	F63	Less food t	Increased (	0.8

For Peer Review

Map91	F78	F96	Less food t	More hung	0.8
Map91	F79	F40	LGA secret	Household	0.6
Map91	F79	F88	LGA secret	Men unable	0.6
Map91	F79	F47	LGA secret	Increase in	0.6
Map91	F79	F87	LGA secret	Men stayin	0.6
Map91	F79	F01	LGA secret	Bad experi	0.6
Map91	F38	F01	Health wor	Bad experi	0.8
Map91	F38	F63	Health wor	Increased	0.8
Map91	F38	F124	Health wor	Not visiting	0.8
Map91	F10	F20	Compulsor	Difficult bre	0.2
Map91	F10	F01	Compulsor	Bad experi	0.2
Map91	F70	F40	Jobs lost	Household	0.6
Map91	F70	F88	Jobs lost	Men unable	0.8
Map91	F70	F47	Jobs lost	Increase in	0.6
Map91	F70	F87	Jobs lost	Men stayin	0.6
Map91	F70	F01	Jobs lost	Bad experi	1
Map91	F70	F64	Jobs lost	Increased	1
Map91	F143	F83	Shortage o	Malnutrition	0.6
Map91	F143	F01	Shortage o	Bad experi	0.8
Map91	F143	F63	Shortage o	Increased	0.8
Map91	F143	F96	Shortage o	More hung	0.8
Map91	F143	F135	Shortage o	Price hike	1
Map91	F143	F78	Shortage o	Less food t	0.8
Map91	F124	F01	Not visiting	Bad experi	0.8
Map91	F124	F63	Not visiting	Increased	0.8
Map91	F147	F01	Stress and	Bad experi	1
Map91	F120	F85	No transpo	Markets/sh	1
Map91	F120	F83	No transpo	Malnutrition	0.6
Map91	F120	F01	No transpo	Bad experi	0.8
Map91	F120	F63	No transpo	Increased	0.8
Map91	F120	F132	No transpo	Poor harve	0.8
Map91	F120	F96	No transpo	More hung	0.8
Map91	F120	F135	No transpo	Price hike	1
Map91	F120	F78	No transpo	Less food t	0.8
Map91	F120	F143	No transpo	Shortage o	1
Map91	F120	F123	No transpo	Not going t	0.8
Map91	F123	F83	Not going t	Malnutrition	0.6
Map91	F123	F01	Not going t	Bad experi	0.8
Map91	F123	F63	Not going t	Increased	0.8
Map91	F123	F132	Not going t	Poor harve	0.8
Map91	F123	F96	Not going t	More hung	0.8
Map91	F123	F135	Not going t	Price hike	0.8
Map91	F123	F78	Not going t	Less food t	0.8
Map91	F123	F143	Not going t	Shortage o	0.6
Map91	F106	F01	No congreg	Bad experi	1
Map91	F106	F147	No congreg	Stress and	1
Map91	F100	F01	Mosques/c	Bad experi	0.6
Map91	F100	F118	Mosques/c	No social g	0.6
Map91	F100	F147	Mosques/c	Stress and	0.6
Map91	F100	F106	Mosques/c	No congreg	0.6
Map92	F40	F01	Household	Bad experi	0.4
Map92	F85	F82	Markets/sh	Lost invest	0.6
Map92	F85	F07	Markets/sh	Businesses	0.6
Map92	F85	F01	Markets/sh	Bad experi	0.8
Map92	F85	F64	Markets/sh	Increased	0.6
Map92	F85	F63	Markets/sh	Increased	0.8

For Peer Review

Map92	F85	F96	Markets/sh	More hung	0.8
Map92	F85	F135	Markets/sh	Price hike	0.8
Map92	F85	F78	Markets/sh	Less food t	0.8
Map92	F87	F40	Men stayin	Household	0.8
Map92	F87	F01	Men stayin	Bad experi	0.4
Map92	F80	F40	Lockdown	Household	0.6
Map92	F80	F85	Lockdown	Markets/sh	0.8
Map92	F80	F87	Lockdown	Men stayin	0.6
Map92	F80	F82	Lockdown	Lost invest	0.6
Map92	F80	F05	Lockdown	Banks clos	0.6
Map92	F80	F07	Lockdown	Businesses	0.6
Map92	F80	F01	Lockdown	Bad experi	0.8
Map92	F80	F64	Lockdown	Increased p	0.6
Map92	F80	F63	Lockdown	Increased c	0.8
Map92	F80	F62	Lockdown	Increased r	0.6
Map92	F80	F158	Lockdown	Youth/child	0.4
Map92	F80	F96	Lockdown	More hung	0.8
Map92	F80	F114	Lockdown	No pilgrima	1
Map92	F80	F130	Lockdown	Physical in	0.4
Map92	F80	F135	Lockdown	Price hike	0.8
Map92	F80	F78	Lockdown	Less food t	0.8
Map92	F80	F13	Lockdown	Could not c	0.4
Map92	F80	F70	Lockdown	Jobs lost	0.4
Map92	F80	F143	Lockdown	Shortage o	0.6
Map92	F80	F142	Lockdown	School/mac	0.4
Map92	F80	F140	Lockdown	Restriction	0.8
Map92	F80	F147	Lockdown	Stress and	0.8
Map92	F80	F120	Lockdown	No transpo	0.6
Map92	F80	F106	Lockdown	No congreg	0.8
Map92	F80	F100	Lockdown	Mosques/c	1
Map92	F82	F01	Lost invest	Bad experi	0.6
Map92	F82	F64	Lost invest	Increased p	0.6
Map92	F05	F01	Banks clos	Bad experi	0.4
Map92	F05	F63	Banks clos	Increased c	0.4
Map92	F05	F62	Banks clos	Increased r	0.4
Map92	F05	F147	Banks clos	Stress and	0.4
Map92	F07	F82	Businesses	Lost invest	0.8
Map92	F07	F01	Businesses	Bad experi	0.6
Map92	F07	F64	Businesses	Increased p	0.6
Map92	F20	F01	Difficult bre	Bad experi	0.6
Map92	F29	F20	Fear of cor	Difficult bre	0.2
Map92	F29	F01	Fear of cor	Bad experi	0.2
Map92	F29	F31	Fear of cor	Fear of eac	0.4
Map92	F29	F10	Fear of cor	Compulsor	0.2
Map92	F64	F01	Increased p	Bad experi	0.6
Map92	F63	F01	Increased c	Bad experi	0.8
Map92	F62	F01	Increased r	Bad experi	0.6
Map92	F62	F63	Increased r	Increased c	0.4
Map92	F158	F01	Youth/child	Bad experi	0.4
Map92	F158	F63	Youth/child	Increased c	0.4
Map92	F158	F62	Youth/child	Increased r	0.4
Map92	F158	F130	Youth/child	Physical in	0.4
Map92	F158	F13	Youth/child	Could not c	0.4
Map92	F158	F147	Youth/child	Stress and	0.4
Map92	F96	F01	More hung	Bad experi	1
Map92	F96	F63	More hung	Increased c	0.8

For Peer Review

Map92	F114	F01	No pilgrima	Bad experi	0.6
Map92	F114	F63	No pilgrima	Increased	0.4
Map92	F114	F62	No pilgrima	Increased	0.6
Map92	F114	F147	No pilgrima	Stress and	0.6
Map92	F130	F01	Physical in	Bad experi	0.6
Map92	F130	F63	Physical in	Increased	0.4
Map92	F130	F62	Physical in	Increased	0.6
Map92	F130	F13	Physical in	Could not	0.6
Map92	F130	F147	Physical in	Stress and	0.6
Map92	F111	F01	No governr	Bad experi	0.8
Map92	F111	F63	No governr	Increased	0.8
Map92	F111	F96	No governr	More hung	0.8
Map92	F111	F78	No governr	Less food t	0.8
Map92	F135	F01	Price hike	Bad experi	0.8
Map92	F135	F63	Price hike	Increased	0.8
Map92	F135	F96	Price hike	More hung	0.8
Map92	F135	F78	Price hike	Less food t	0.8
Map92	F31	F01	Fear of eac	Bad experi	0.2
Map92	F78	F01	Less food t	Bad experi	0.8
Map92	F78	F63	Less food t	Increased	0.8
Map92	F78	F96	Less food t	More hung	0.8
Map92	F13	F01	Could not c	Bad experi	0.6
Map92	F13	F63	Could not c	Increased	0.4
Map92	F13	F62	Could not c	Increased	0.6
Map92	F13	F147	Could not c	Stress and	0.6
Map92	F10	F20	Compulsor	Difficult bre	0.4
Map92	F10	F01	Compulsor	Bad experi	0.4
Map92	F70	F01	Jobs lost	Bad experi	0.6
Map92	F70	F63	Jobs lost	Increased	0.4
Map92	F70	F62	Jobs lost	Increased	0.6
Map92	F70	F147	Jobs lost	Stress and	0.6
Map92	F143	F01	Shortage o	Bad experi	0.6
Map92	F143	F63	Shortage o	Increased	0.6
Map92	F143	F96	Shortage o	More hung	0.6
Map92	F143	F78	Shortage o	Less food t	0.6
Map92	F142	F01	School/ma	Bad experi	0.4
Map92	F142	F63	School/ma	Increased	0.4
Map92	F142	F62	School/ma	Increased	0.4
Map92	F142	F70	School/ma	Jobs lost	0.4
Map92	F142	F147	School/ma	Stress and	0.4
Map92	F140	F40	Restriction	Household	0.6
Map92	F140	F87	Restriction	Men stayin	0.6
Map92	F140	F01	Restriction	Bad experi	0.6
Map92	F140	F63	Restriction	Increased	0.6
Map92	F140	F96	Restriction	More hung	0.6
Map92	F140	F78	Restriction	Less food t	0.6
Map92	F140	F143	Restriction	Shortage o	0.6
Map92	F140	F120	Restriction	No transpo	0.6
Map92	F147	F01	Stress and	Bad experi	0.6
Map92	F147	F63	Stress and	Increased	0.4
Map92	F147	F62	Stress and	Increased	0.6
Map92	F120	F01	No transpo	Bad experi	0.6
Map92	F120	F63	No transpo	Increased	0.6
Map92	F120	F96	No transpo	More hung	0.6
Map92	F120	F78	No transpo	Less food t	0.6
Map92	F120	F143	No transpo	Shortage o	0.8

For Peer Review

Map92	F106	F01	No congreg	Bad experi	0.6
Map92	F106	F63	No congreg	Increased c	0.4
Map92	F106	F62	No congreg	Increased r	0.6
Map92	F106	F147	No congreg	Stress and	1
Map92	F100	F01	Mosques/c	Bad experi	0.6
Map92	F100	F63	Mosques/c	Increased c	0.4
Map92	F100	F62	Mosques/c	Increased r	0.6
Map92	F100	F147	Mosques/c	Stress and	0.8
Map92	F100	F106	Mosques/c	No congreg	0.8
Map93	F47	F49	Increase in	Increase in	0.4
Map93	F47	F01	Increase in	Bad experi	0.4
Map93	F47	F63	Increase in	Increased c	0.4
Map93	F49	F01	Increase in	Bad experi	0.8
Map93	F49	F63	Increase in	Increased c	0.8
Map93	F87	F47	Men stayin	Increase in	0.4
Map93	F87	F49	Men stayin	Increase in	0.4
Map93	F87	F01	Men stayin	Bad experi	0.4
Map93	F87	F63	Men stayin	Increased c	0.4
Map93	F87	F129	Men stayin	Physical ab	0.4
Map93	F81	F01	Loss of Ha	Bad experi	0.6
Map93	F81	F63	Loss of Ha	Increased c	0.6
Map93	F81	F147	Loss of Ha	Stress and	0.6
Map93	F80	F47	Lockdown	Increase in	0.4
Map93	F80	F49	Lockdown	Increase in	0.4
Map93	F80	F87	Lockdown	Men stayin	0.6
Map93	F80	F82	Lockdown	Lost invest	0.8
Map93	F80	F01	Lockdown	Bad experi	0.6
Map93	F80	F29	Lockdown	Fear of cor	0.6
Map93	F80	F64	Lockdown	Increased p	0.6
Map93	F80	F63	Lockdown	Increased c	0.6
Map93	F80	F60	Lockdown	Increased i	0.6
Map93	F80	F85	Lockdown	Markets/sh	0.8
Map93	F80	F118	Lockdown	No social g	0.6
Map93	F80	F96	Lockdown	More hung	0.6
Map93	F80	F135	Lockdown	Price hike	0.6
Map93	F80	F31	Lockdown	Fear of eac	0.6
Map93	F80	F78	Lockdown	Less food t	0.6
Map93	F80	F16	Lockdown	Delay in ec	0.6
Map93	F80	F142	Lockdown	School/ma	0.6
Map93	F80	F140	Lockdown	Restriction	0.8
Map93	F80	F147	Lockdown	Stress and	0.6
Map93	F80	F120	Lockdown	No transpo	0.6
Map93	F80	F122	Lockdown	Non compl	0.6
Map93	F80	F129	Lockdown	Physical ab	0.4
Map93	F80	F128	Lockdown	People fee	0.4
Map93	F80	F106	Lockdown	No congreg	0.4
Map93	F82	F01	Lost invest	Bad experi	0.6
Map93	F82	F64	Lost invest	Increased p	0.6
Map93	F82	F96	Lost invest	More hung	0.6
Map93	F82	F135	Lost invest	Price hike	0.6
Map93	F82	F78	Lost invest	Less food t	0.6
Map93	F06	F81	Borders clo	Loss of Ha	0.4
Map93	F06	F01	Borders clo	Bad experi	0.6
Map93	F06	F29	Borders clo	Fear of cor	0.8
Map93	F06	F63	Borders clo	Increased c	0.6
Map93	F06	F118	Borders clo	No social g	0.6

For Peer Review

Map93	F06	F114	Borders clc	No pilgrima	0.8
Map93	F06	F31	Borders clc	Fear of eac	0.6
Map93	F06	F147	Borders clc	Stress and	0.6
Map93	F06	F106	Borders clc	No congreg	0.4
Map93	F29	F01	Fear of cor	Bad experi	0.6
Map93	F29	F63	Fear of cor	Increased (	0.6
Map93	F29	F118	Fear of cor	No social g	0.6
Map93	F29	F31	Fear of cor	Fear of eac	0.6
Map93	F29	F147	Fear of cor	Stress and	0.6
Map93	F29	F106	Fear of cor	No congreg	0.4
Map93	F64	F01	Increased p	Bad experi	1
Map93	F64	F96	Increased p	More hung	1
Map93	F63	F01	Increased (	Bad experi	1
Map93	F60	F01	Increased i	Bad experi	0.4
Map93	F60	F63	Increased i	Increased (	0.4
Map93	F60	F147	Increased i	Stress and	0.4
Map93	F60	F128	Increased i	People fee	0.4
Map93	F85	F47	Markets/sh	Increase in	0.4
Map93	F85	F49	Markets/sh	Increase in	0.4
Map93	F85	F87	Markets/sh	Men stayin	0.6
Map93	F85	F82	Markets/sh	Lost invest	0.8
Map93	F85	F01	Markets/sh	Bad experi	0.6
Map93	F85	F64	Markets/sh	Increased p	0.6
Map93	F85	F63	Markets/sh	Increased (	0.4
Map93	F85	F96	Markets/sh	More hung	0.6
Map93	F85	F135	Markets/sh	Price hike	0.6
Map93	F85	F78	Markets/sh	Less food t	0.6
Map93	F85	F129	Markets/sh	Physical ab	0.4
Map93	F118	F01	No social g	Bad experi	0.6
Map93	F118	F63	No social g	Increased (	0.6
Map93	F118	F147	No social g	Stress and	0.6
Map93	F114	F81	No pilgrima	Loss of Ha	0.4
Map93	F114	F01	No pilgrima	Bad experi	0.4
Map93	F114	F63	No pilgrima	Increased (	0.4
Map93	F114	F147	No pilgrima	Stress and	0.4
Map93	F96	F01	More hung	Bad experi	1
Map93	F135	F01	Price hike	Bad experi	0.8
Map93	F135	F64	Price hike	Increased p	0.8
Map93	F135	F96	Price hike	More hung	0.8
Map93	F135	F78	Price hike	Less food t	0.6
Map93	F31	F01	Fear of eac	Bad experi	0.6
Map93	F31	F63	Fear of eac	Increased (	0.6
Map93	F31	F118	Fear of eac	No social g	0.8
Map93	F31	F147	Fear of eac	Stress and	0.6
Map93	F78	F01	Less food t	Bad experi	0.6
Map93	F78	F96	Less food t	More hung	0.6
Map93	F16	F01	Delay in ec	Bad experi	0.8
Map93	F142	F01	School/ma	Bad experi	0.8
Map93	F142	F16	School/ma	Delay in ec	1
Map93	F140	F47	Restriction	Increase in	0.4
Map93	F140	F49	Restriction	Increase in	0.4
Map93	F140	F87	Restriction	Men stayin	0.4
Map93	F140	F01	Restriction	Bad experi	0.4
Map93	F140	F63	Restriction	Increased (	0.4
Map93	F140	F120	Restriction	No transpo	0.6
Map93	F140	F129	Restriction	Physical ab	0.4

For Peer Review

Map93	F147	F01	Stress and	Bad experi	0.8
Map93	F147	F63	Stress and	Increased (	0.8
Map93	F120	F47	No transpo	Increase in	0.4
Map93	F120	F49	No transpo	Increase in	0.4
Map93	F120	F87	No transpo	Men stayin	0.4
Map93	F120	F01	No transpo	Bad experi	0.4
Map93	F120	F63	No transpo	Increased (	0.4
Map93	F120	F129	No transpo	Physical ab	0.4
Map93	F122	F01	Non compl	Bad experi	0.4
Map93	F122	F63	Non compl	Increased (	0.4
Map93	F122	F60	Non compl	Increased i	0.6
Map93	F122	F147	Non compl	Stress and	0.4
Map93	F122	F128	Non compl	People fee	0.4
Map93	F129	F47	Physical ab	Increase in	0.6
Map93	F129	F49	Physical ab	Increase in	0.4
Map93	F129	F01	Physical ab	Bad experi	0.4
Map93	F129	F63	Physical ab	Increased (	0.4
Map93	F128	F01	People feel	Bad experi	0.8
Map93	F128	F63	People feel	Increased (	0.8
Map93	F128	F147	People feel	Stress and	0.8
Map93	F106	F01	No congreg	Bad experi	0.8
Map93	F106	F63	No congreg	Increased (	0.8
Map93	F106	F147	No congreg	Stress and	1
Map94	F45	F82	Increase in	Lost invest	0.8
Map94	F45	F01	Increase in	Bad experi	0.8
Map94	F45	F64	Increase in	Increased p	0.8
Map94	F85	F82	Markets/sh	Lost invest	0.6
Map94	F85	F07	Markets/sh	Businesses	0.8
Map94	F85	F01	Markets/sh	Bad experi	0.6
Map94	F85	F64	Markets/sh	Increased p	0.6
Map94	F85	F70	Markets/sh	Jobs lost	0.6
Map94	F84	F45	Malnutritior	Increase in	0.6
Map94	F84	F82	Malnutritior	Lost invest	0.6
Map94	F84	F01	Malnutritior	Bad experi	0.6
Map94	F84	F64	Malnutritior	Increased p	0.6
Map94	F80	F45	Lockdown	Increase in	0.6
Map94	F80	F85	Lockdown	Markets/sh	0.8
Map94	F80	F84	Lockdown	Malnutritior	0.6
Map94	F80	F82	Lockdown	Lost invest	0.6
Map94	F80	F07	Lockdown	Businesses	0.8
Map94	F80	F01	Lockdown	Bad experi	0.6
Map94	F80	F67	Lockdown	Increased s	0.2
Map94	F80	F64	Lockdown	Increased p	0.6
Map94	F80	F63	Lockdown	Increased (	0.4
Map94	F80	F158	Lockdown	Youth/child	0.4
Map94	F80	F16	Lockdown	Delay in ec	0.6
Map94	F80	F96	Lockdown	More hung	0.6
Map94	F80	F114	Lockdown	No pilgrima	0.4
Map94	F80	F118	Lockdown	No social g	0.4
Map94	F80	F131	Lockdown	Physical pu	0.2
Map94	F80	F136	Lockdown	Reduced p	0.4
Map94	F80	F78	Lockdown	Less food t	0.6
Map94	F80	F133	Lockdown	Poor perfor	0.8
Map94	F80	F38	Lockdown	Health wor	0.4
Map94	F80	F70	Lockdown	Jobs lost	0.6
Map94	F80	F142	Lockdown	School/mac	1

For Peer Review

Map94	F80	F140	Lockdown	Restriction	0.4
Map94	F80	F147	Lockdown	Stress and	0.4
Map94	F80	F120	Lockdown	No transpo	0.4
Map94	F80	F123	Lockdown	Not going t	0.8
Map94	F80	F144	Lockdown	Social dista	0.4
Map94	F80	F109	Lockdown	No financia	0.4
Map94	F80	F106	Lockdown	No congreg	0.4
Map94	F80	F100	Lockdown	Mosques/c	0.4
Map94	F82	F01	Lost invest	Bad experi	0.8
Map94	F82	F64	Lost invest	Increased p	0.8
Map94	F07	F82	Businesses	Lost invest	0.6
Map94	F07	F07	Businesses	Businesses	0.4
Map94	F07	F01	Businesses	Bad experi	0.6
Map94	F07	F64	Businesses	Increased p	0.6
Map94	F07	F70	Businesses	Jobs lost	0.6
Map94	F20	F01	Difficult bre	Bad experi	0.4
Map94	F67	F01	Increased s	Bad experi	0.2
Map94	F67	F131	Increased s	Physical pu	0.2
Map94	F67	F147	Increased s	Stress and	0.2
Map94	F29	F01	Fear of cor	Bad experi	0.4
Map94	F29	F31	Fear of cor	Fear of eac	0.6
Map94	F29	F147	Fear of cor	Stress and	0.4
Map94	F64	F01	Increased p	Bad experi	1
Map94	F63	F01	Increased c	Bad experi	0.8
Map94	F158	F01	Youth/child	Bad experi	0.2
Map94	F158	F67	Youth/child	Increased s	0.2
Map94	F158	F131	Youth/child	Physical pu	0.2
Map94	F158	F147	Youth/child	Stress and	0.2
Map94	F16	F01	Delay in ed	Bad experi	0.8
Map94	F96	F01	More hung	Bad experi	0.8
Map94	F114	F01	No pilgrima	Bad experi	0.4
Map94	F114	F147	No pilgrima	Stress and	0.4
Map94	F114	F109	No pilgrima	No financia	0.4
Map94	F118	F01	No social g	Bad experi	0.6
Map94	F118	F136	No social g	Reduced p	0.4
Map94	F118	F147	No social g	Stress and	0.6
Map94	F131	F01	Physical pu	Bad experi	0.6
Map94	F136	F01	Reduced p	Bad experi	0.2
Map94	F136	F147	Reduced p	Stress and	0.2
Map94	F135	F01	Price hike	Bad experi	0.8
Map94	F135	F96	Price hike	More hung	0.8
Map94	F135	F78	Price hike	Less food t	0.8
Map94	F31	F01	Fear of eac	Bad experi	0.4
Map94	F31	F147	Fear of eac	Stress and	0.4
Map94	F78	F01	Less food t	Bad experi	0.8
Map94	F78	F96	Less food t	More hung	0.8
Map94	F133	F01	Poor perfor	Bad experi	0.6
Map94	F133	F16	Poor perfor	Delay in ed	0.6
Map94	F38	F01	Health wor	Bad experi	0.6
Map94	F38	F63	Health wor	Increased c	0.6
Map94	F10	F20	Compulsor	Difficult bre	0.2
Map94	F10	F01	Compulsor	Bad experi	0.2
Map94	F70	F82	Jobs lost	Lost invest	0.8
Map94	F70	F01	Jobs lost	Bad experi	0.8
Map94	F70	F64	Jobs lost	Increased p	0.8
Map94	F142	F01	School/ma	Bad experi	0.6

For Peer Review

Map94	F142	F67	School/ma	Increased s	0.2
Map94	F142	F158	School/ma	Youth/child	0.4
Map94	F142	F16	School/ma	Delay in ec	0.6
Map94	F142	F131	School/ma	Physical pu	0.2
Map94	F142	F133	School/ma	Poor perfor	0.8
Map94	F142	F147	School/ma	Stress and	0.2
Map94	F140	F01	Restriction	Bad experi	0.6
Map94	F140	F63	Restriction	Increased c	0.6
Map94	F140	F114	Restriction	No pilgrima	0.4
Map94	F140	F38	Restriction	Health wor	0.6
Map94	F140	F147	Restriction	Stress and	0.4
Map94	F140	F120	Restriction	No transpo	0.6
Map94	F140	F109	Restriction	No financia	0.4
Map94	F147	F01	Stress and	Bad experi	0.6
Map94	F120	F01	No transpo	Bad experi	0.6
Map94	F120	F63	No transpo	Increased c	0.6
Map94	F120	F114	No transpo	No pilgrima	0.4
Map94	F120	F38	No transpo	Health wor	0.8
Map94	F120	F147	No transpo	Stress and	0.4
Map94	F120	F109	No transpo	No financia	0.4
Map94	F123	F01	Not going t	Bad experi	0.6
Map94	F123	F96	Not going t	More hung	0.6
Map94	F123	F78	Not going t	Less food t	0.6
Map94	F144	F01	Social dista	Bad experi	0.6
Map94	F144	F118	Social dista	No social g	0.8
Map94	F144	F136	Social dista	Reduced p	0.4
Map94	F144	F147	Social dista	Stress and	0.6
Map94	F144	F106	Social dista	No congreg	0.8
Map94	F144	F100	Social dista	Mosques/c	0.8
Map94	F109	F01	No financia	Bad experi	0.4
Map94	F109	F147	No financia	Stress and	0.4
Map94	F106	F01	No congreg	Bad experi	0.6
Map94	F106	F118	No congreg	No social g	0.8
Map94	F106	F136	No congreg	Reduced p	0.4
Map94	F106	F147	No congreg	Stress and	0.6
Map94	F100	F01	Mosques/c	Bad experi	0.6
Map94	F100	F118	Mosques/c	No social g	0.8
Map94	F100	F136	Mosques/c	Reduced p	0.4
Map94	F100	F147	Mosques/c	Stress and	0.6
Map94	F100	F106	Mosques/c	No congreg	1
Map95	F89	F01	Misconcep	Bad experi	0.4
Map95	F85	F82	Markets/sh	Lost invest	0.4
Map95	F85	F07	Markets/sh	Businesses	0.6
Map95	F85	F01	Markets/sh	Bad experi	0.4
Map95	F85	F147	Markets/sh	Stress and	0.4
Map95	F80	F85	Lockdown	Markets/sh	0.6
Map95	F80	F82	Lockdown	Lost invest	0.4
Map95	F80	F07	Lockdown	Businesses	0.6
Map95	F80	F01	Lockdown	Bad experi	0.6
Map95	F80	F69	Lockdown	Increased t	0.6
Map95	F80	F67	Lockdown	Increased s	0.2
Map95	F80	F64	Lockdown	Increased p	0.4
Map95	F80	F62	Lockdown	Increased r	0.6
Map95	F80	F16	Lockdown	Delay in ec	0.6
Map95	F80	F96	Lockdown	More hung	0.4
Map95	F80	F130	Lockdown	Physical in	0.4

For Peer Review

Map95	F80	F51	Lockdown	Increase in	0.6
Map95	F80	F58	Lockdown	Increased (	0.6
Map95	F80	F13	Lockdown	Could not (	0.4
Map95	F80	F11	Lockdown	Compulsor	1
Map95	F80	F70	Lockdown	Jobs lost	0.4
Map95	F80	F143	Lockdown	Shortage o	0.4
Map95	F80	F142	Lockdown	School/ma	0.8
Map95	F80	F147	Lockdown	Stress and	0.4
Map95	F80	F106	Lockdown	No congreg	0.6
Map95	F80	F100	Lockdown	Mosques/c	0.8
Map95	F82	F01	Lost invest	Bad experi	0.8
Map95	F82	F147	Lost invest	Stress and	0.8
Map95	F07	F82	Businesses	Lost invest	0.4
Map95	F07	F01	Businesses	Bad experi	0.4
Map95	F07	F147	Businesses	Stress and	0.4
Map95	F20	F01	Difficult bre	Bad experi	0.4
Map95	F69	F01	Increased t	Bad experi	0.6
Map95	F69	F58	Increased t	Increased (	0.6
Map95	F02	F01	Bad experi	Bad experi	0.6
Map95	F67	F01	Increased s	Bad experi	0.6
Map95	F29	F01	Fear of cor	Bad experi	0.4
Map95	F29	F147	Fear of cor	Stress and	0.4
Map95	F64	F01	Increased p	Bad experi	0.6
Map95	F64	F67	Increased p	Increased s	0.2
Map95	F64	F96	Increased p	More hung	0.6
Map95	F64	F143	Increased p	Shortage o	0.8
Map95	F63	F01	Increased (	Bad experi	0.4
Map95	F62	F01	Increased r	Bad experi	0.6
Map95	F151	F151	Two shifts	Two shifts	0.6
Map95	F154	F20	Wearing fa	Difficult bre	0.2
Map95	F154	F01	Wearing fa	Bad experi	0.2
Map95	F16	F01	Delay in ed	Bad experi	0.6
Map95	F16	F69	Delay in ed	Increased t	0.8
Map95	F16	F51	Delay in ed	Increase in	0.8
Map95	F16	F58	Delay in ed	Increased (	0.6
Map95	F96	F01	More hung	Bad experi	0.8
Map95	F130	F01	Physical in	Bad experi	0.8
Map95	F130	F130	Physical in	Physical in	0.6
Map95	F51	F01	Increase in	Bad experi	0.6
Map95	F51	F69	Increase in	Increased t	0.8
Map95	F51	F58	Increase in	Increased (	0.6
Map95	F58	F01	Increased (	Bad experi	0.8
Map95	F13	F01	Could not (	Bad experi	0.6
Map95	F13	F130	Could not (	Physical in	0.6
Map95	F11	F01	Compulsor	Bad experi	0.4
Map95	F11	F67	Compulsor	Increased s	0.2
Map95	F11	F64	Compulsor	Increased p	0.4
Map95	F11	F96	Compulsor	More hung	0.4
Map95	F11	F130	Compulsor	Physical in	0.4
Map95	F11	F13	Compulsor	Could not (	0.4
Map95	F11	F70	Compulsor	Jobs lost	0.4
Map95	F11	F143	Compulsor	Shortage o	0.4
Map95	F70	F01	Jobs lost	Bad experi	0.6
Map95	F70	F67	Jobs lost	Increased s	0.2
Map95	F70	F64	Jobs lost	Increased p	0.6
Map95	F70	F96	Jobs lost	More hung	0.6

For Peer Review

Map95	F70	F143	Jobs lost	Shortage o	0.6
Map95	F143	F01	Shortage o	Bad experi	0.6
Map95	F143	F96	Shortage o	More hung	0.6
Map95	F142	F01	School/ma	Bad experi	0.6
Map95	F142	F69	School/ma	Increased t	0.6
Map95	F142	F16	School/ma	Delay in ec	0.6
Map95	F142	F51	School/ma	Increase in	0.6
Map95	F142	F58	School/ma	Increased	0.6
Map95	F141	F89	Rumors on	Misconcep	0.4
Map95	F141	F01	Rumors on	Bad experi	0.4
Map95	F147	F01	Stress and	Bad experi	0.8
Map95	F106	F01	No congreg	Bad experi	0.6
Map95	F106	F62	No congreg	Increased r	0.6
Map95	F100	F01	Mosques/c	Bad experi	0.6
Map95	F100	F62	Mosques/c	Increased r	0.6
Map95	F100	F106	Mosques/c	No congreg	0.6
Map96	F20	F63	Difficult bre	Increased	0.4
Map96	F20	F01	Difficult bre	Bad experi	0.4
Map96	F64	F60	Increased	Increased i	0.6
Map96	F64	F49	Increased	Increase in	0.6
Map96	F64	F01	Increased	Bad experi	1
Map96	F63	F01	Increased	Bad experi	0.8
Map96	F62	F01	Increased	Bad experi	0.6
Map96	F60	F60	Increased i	Increased i	0.6
Map96	F60	F49	Increased i	Increase in	0.8
Map96	F60	F01	Increased i	Bad experi	0.6
Map96	F94	F64	More hous	Increased	0.8
Map96	F94	F60	More hous	Increased i	0.6
Map96	F94	F49	More hous	Increase in	0.6
Map96	F94	F01	More hous	Bad experi	0.8
Map96	F98	F01	More mate	Bad experi	0.4
Map96	F99	F64	More unwa	Increased	0.6
Map96	F99	F60	More unwa	Increased i	0.6
Map96	F99	F94	More unwa	More hous	0.6
Map96	F99	F98	More unwa	More mate	0.6
Map96	F99	F49	More unwa	Increase in	0.6
Map96	F99	F01	More unwa	Bad experi	0.6
Map96	F135	F64	Price hike	Increased	0.6
Map96	F135	F60	Price hike	Increased i	0.6
Map96	F135	F49	Price hike	Increase in	0.6
Map96	F135	F01	Price hike	Bad experi	0.6
Map96	F30	F20	Fear of dea	Difficult bre	0.4
Map96	F30	F63	Fear of dea	Increased	0.4
Map96	F30	F62	Fear of dea	Increased r	0.4
Map96	F30	F31	Fear of dea	Fear of eac	0.4
Map96	F30	F124	Fear of dea	Not visiting	0.4
Map96	F30	F01	Fear of dea	Bad experi	0.4
Map96	F30	F02	Fear of dea	Bad experi	0.4
Map96	F30	F10	Fear of dea	Compulsor	0.4
Map96	F30	F145	Fear of dea	Stigma aga	0.4
Map96	F31	F20	Fear of eac	Difficult bre	0.6
Map96	F31	F63	Fear of eac	Increased	0.4
Map96	F31	F62	Fear of eac	Increased r	0.4
Map96	F31	F124	Fear of eac	Not visiting	0.6
Map96	F31	F01	Fear of eac	Bad experi	0.4
Map96	F31	F02	Fear of eac	Bad experi	0.4

For Peer Review

Map96	F31	F10	Fear of eac	Compulsor	0.6
Map96	F31	F145	Fear of eac	Stigma aga	0.4
Map96	F32	F62	Feeling soc	Increased r	0.8
Map96	F32	F76	Feeling soc	Less affect	0.8
Map96	F32	F01	Feeling soc	Bad experi	0.6
Map96	F76	F62	Less affect	Increased r	1
Map96	F76	F01	Less affect	Bad experi	0.6
Map96	F70	F64	Jobs lost	Increased p	0.6
Map96	F70	F60	Jobs lost	Increased i	0.6
Map96	F70	F49	Jobs lost	Increase in	0.6
Map96	F70	F01	Jobs lost	Bad experi	0.6
Map96	F71	F64	Lack of aw	Increased p	0.4
Map96	F71	F60	Lack of aw	Increased i	0.4
Map96	F71	F70	Lack of aw	Jobs lost	0.4
Map96	F71	F49	Lack of aw	Increase in	0.4
Map96	F71	F01	Lack of aw	Bad experi	0.4
Map96	F72	F40	Lack of bas	Household	0.8
Map96	F72	F47	Lack of bas	Increase in	0.6
Map96	F72	F01	Lack of bas	Bad experi	0.2
Map96	F124	F62	Not visiting	Increased r	0.4
Map96	F124	F124	Not visiting	Not visiting	0.4
Map96	F124	F01	Not visiting	Bad experi	0.4
Map96	F124	F02	Not visiting	Bad experi	0.4
Map96	F124	F145	Not visiting	Stigma aga	0.4
Map96	F121	F62	No wedding	Increased r	0.4
Map96	F121	F32	No wedding	Feeling soc	0.4
Map96	F121	F76	No wedding	Less affect	0.4
Map96	F121	F01	No wedding	Bad experi	0.4
Map96	F120	F64	No transpo	Increased p	0.6
Map96	F120	F60	No transpo	Increased i	0.6
Map96	F120	F135	No transpo	Price hike	0.6
Map96	F120	F123	No transpo	Not going t	0.6
Map96	F120	F49	No transpo	Increase in	0.6
Map96	F120	F01	No transpo	Bad experi	0.6
Map96	F120	F143	No transpo	Shortage o	0.6
Map96	F123	F64	Not going t	Increased p	0.6
Map96	F123	F60	Not going t	Increased i	0.6
Map96	F123	F135	Not going t	Price hike	0.6
Map96	F123	F49	Not going t	Increase in	0.6
Map96	F123	F01	Not going t	Bad experi	0.6
Map96	F123	F143	Not going t	Shortage o	0.6
Map96	F40	F47	Household	Increase in	0.6
Map96	F40	F01	Household	Bad experi	0.2
Map96	F43	F01	Improper ir	Bad experi	0.6
Map96	F47	F01	Increase in	Bad experi	0.2
Map96	F49	F01	Increase in	Bad experi	0.6
Map96	F87	F64	Men stayin	Increased p	0.6
Map96	F87	F60	Men stayin	Increased i	0.6
Map96	F87	F94	Men stayin	More hous	0.6
Map96	F87	F98	Men stayin	More mate	0.6
Map96	F87	F99	Men stayin	More unwa	0.8
Map96	F87	F72	Men stayin	Lack of bas	0.6
Map96	F87	F40	Men stayin	Household	0.6
Map96	F87	F47	Men stayin	Increase in	0.6
Map96	F87	F49	Men stayin	Increase in	0.6
Map96	F87	F01	Men stayin	Bad experi	0.6

For Peer Review

Map96	F80	F64	Lockdown	Increased	0.6
Map96	F80	F62	Lockdown	Increased	0.6
Map96	F80	F60	Lockdown	Increased i	0.6
Map96	F80	F94	Lockdown	More hous	0.6
Map96	F80	F98	Lockdown	More mate	0.6
Map96	F80	F99	Lockdown	More unwa	0.8
Map96	F80	F135	Lockdown	Price hike	0.6
Map96	F80	F70	Lockdown	Jobs lost	0.4
Map96	F80	F71	Lockdown	Lack of aw	0.4
Map96	F80	F72	Lockdown	Lack of bas	0.6
Map96	F80	F120	Lockdown	No transpo	0.62
Map96	F80	F123	Lockdown	Not going t	0.6
Map96	F80	F40	Lockdown	Household	0.6
Map96	F80	F43	Lockdown	Improper ir	0.2
Map96	F80	F47	Lockdown	Increase in	0.6
Map96	F80	F49	Lockdown	Increase in	0.6
Map96	F80	F87	Lockdown	Men stayin	0.8
Map96	F80	F07	Lockdown	Businesses	0.8
Map96	F80	F01	Lockdown	Bad experi	0.6
Map96	F80	F158	Lockdown	Youth/child	0.8
Map96	F80	F119	Lockdown	No tourism	0.4
Map96	F80	F56	Lockdown	Increased	0.2
Map96	F80	F58	Lockdown	Increased	0.6
Map96	F80	F18	Lockdown	Developme	0.4
Map96	F80	F16	Lockdown	Delay in ec	0.8
Map96	F80	F15	Lockdown	Decreased	0.2
Map96	F80	F143	Lockdown	Shortage o	0.6
Map96	F80	F142	Lockdown	School/ma	0.8
Map96	F80	F140	Lockdown	Restriction	0.62
Map96	F80	F147	Lockdown	Stress and	0.62
Map96	F80	F106	Lockdown	No congreg	0.62
Map96	F80	F100	Lockdown	Mosques/c	0.62
Map96	F07	F64	Businesses	Increased	0.6
Map96	F07	F60	Businesses	Increased i	0.6
Map96	F07	F94	Businesses	More hous	0.6
Map96	F07	F98	Businesses	More mate	0.6
Map96	F07	F99	Businesses	More unwa	0.8
Map96	F07	F72	Businesses	Lack of bas	0.6
Map96	F07	F40	Businesses	Household	0.6
Map96	F07	F47	Businesses	Increase in	0.6
Map96	F07	F49	Businesses	Increase in	0.6
Map96	F07	F87	Businesses	Men stayin	0.8
Map96	F07	F01	Businesses	Bad experi	0.6
Map96	F02	F62	Bad experi	Increased	0.6
Map96	F02	F124	Bad experi	Not visiting	0.6
Map96	F02	F01	Bad experi	Bad experi	0.6
Map96	F02	F02	Bad experi	Bad experi	0.4
Map96	F02	F145	Bad experi	Stigma aga	0.8
Map96	F158	F62	Youth/child	Increased	0.6
Map96	F158	F01	Youth/child	Bad experi	0.6
Map96	F158	F58	Youth/child	Increased	0.6
Map96	F158	F16	Youth/child	Delay in ec	1
Map96	F118	F62	No social g	Increased	0.4
Map96	F118	F32	No social g	Feeling soc	0.4
Map96	F118	F76	No social g	Less affect	0.4
Map96	F118	F121	No social g	No weddin	0.6

For Peer Review

Map96	F118	F01	No social g	Bad experi	0.4
Map96	F118	F110	No social g	No funerals	0.4
Map96	F119	F43	No tourism	Improper ir	0.2
Map96	F119	F01	No tourism	Bad experi	0.2
Map96	F119	F15	No tourism	Decreased	0.2
Map96	F110	F62	No funerals	Increased r	0.2
Map96	F110	F32	No funerals	Feeling soc	0.2
Map96	F110	F76	No funerals	Less affect	0.2
Map96	F110	F01	No funerals	Bad experi	0.2
Map96	F56	F64	Increased (	Increased p	0.8
Map96	F56	F60	Increased (	Increased i	0.6
Map96	F56	F49	Increased (	Increase in	0.6
Map96	F56	F01	Increased (	Bad experi	0.8
Map96	F56	F56	Increased (	Increased (	0.4
Map96	F58	F62	Increased (	Increased r	0.8
Map96	F58	F01	Increased (	Bad experi	0.6
Map96	F18	F64	Developme	Increased p	0.4
Map96	F18	F60	Developme	Increased i	0.4
Map96	F18	F70	Developme	Jobs lost	0.4
Map96	F18	F71	Developme	Lack of aw	0.6
Map96	F18	F49	Developme	Increase in	0.4
Map96	F18	F01	Developme	Bad experi	0.4
Map96	F18	F56	Developme	Increased (	0.2
Map96	F10	F20	Compulsor	Difficult bre	0.6
Map96	F10	F63	Compulsor	Increased (	0.4
Map96	F10	F01	Compulsor	Bad experi	0.4
Map96	F16	F01	Delay in ec	Bad experi	0.6
Map96	F15	F43	Decreased	Improper ir	0.4
Map96	F15	F01	Decreased	Bad experi	0.4
Map96	F143	F64	Shortage o	Increased p	0.6
Map96	F143	F60	Shortage o	Increased i	0.6
Map96	F143	F135	Shortage o	Price hike	0.6
Map96	F143	F49	Shortage o	Increase in	0.6
Map96	F143	F01	Shortage o	Bad experi	0.6
Map96	F142	F62	School/ma	Increased r	0.6
Map96	F142	F01	School/ma	Bad experi	0.6
Map96	F142	F158	School/ma	Youth/child	0.8
Map96	F142	F58	School/ma	Increased (	0.6
Map96	F142	F16	School/ma	Delay in ec	0.8
Map96	F140	F64	Restriction	Increased p	0.6
Map96	F140	F60	Restriction	Increased i	0.6
Map96	F140	F94	Restriction	More hous	0.6
Map96	F140	F98	Restriction	More mate	0.6
Map96	F140	F99	Restriction	More unwa	0.8
Map96	F140	F135	Restriction	Price hike	0.6
Map96	F140	F70	Restriction	Jobs lost	0.4
Map96	F140	F71	Restriction	Lack of aw	0.4
Map96	F140	F72	Restriction	Lack of bas	0.6
Map96	F140	F120	Restriction	No transpo	0.8
Map96	F140	F123	Restriction	Not going t	0.6
Map96	F140	F40	Restriction	Household	0.6
Map96	F140	F47	Restriction	Increase in	0.6
Map96	F140	F49	Restriction	Increase in	0.6
Map96	F140	F87	Restriction	Men stayin	0.8
Map96	F140	F07	Restriction	Businesses	0.8
Map96	F140	F01	Restriction	Bad experi	0.6

For Peer Review

Map96	F140	F56	Restriction	Increased (	0.2
Map96	F140	F18	Restriction	Developme	0.4
Map96	F140	F143	Restriction	Shortage o	0.6
Map96	F140	F147	Restriction	Stress and	0.8
Map96	F140	F106	Restriction	No congreg	0.8
Map96	F140	F100	Restriction	Mosques/c	0.8
Map96	F147	F01	Stress and	Bad experi	0.6
Map96	F145	F62	Stigma aga	Increased r	0.6
Map96	F145	F01	Stigma aga	Bad experi	0.6
Map96	F106	F01	No congreg	Bad experi	0.6
Map96	F106	F147	No congreg	Stress and	0.8
Map96	F100	F01	Mosques/c	Bad experi	0.6
Map96	F100	F147	Mosques/c	Stress and	0.8
Map96	F100	F106	Mosques/c	No congreg	0.8

For Peer Review

Supplementary material 4. Categories identified as causes of worse experience of COVID-19 across groups

Category	All maps	Female	Male	Urban	Rural	Remote	Com.	High L*	Disab.	IDP
**LOCKDOWN**	Yes	Yes	Yes	Yes	Yes	Yes	Yes	Yes	Yes	Yes
**REDUCED BUSINESSES/JOBS**	Yes	Yes	Yes	Yes	Yes	Yes	Yes	Yes	Yes	Yes
**REDUCED SOCIAL CONTACT/ACTIVITIES**	Yes	Yes	Yes	Yes	Yes	Yes	Yes	Yes	Yes	Yes
**REDUCED HEALTH SERVICES**	Yes	Yes	Yes	Yes	Yes	Yes	Yes	Yes	Yes	Yes
**PROBLEMS WITH PPE**	Yes	Yes	Yes	Yes	Yes	Yes	Yes	Yes	Yes	Yes
**FEAR AND MISCONCEPTION OF COVID-19**	Yes	Yes	Yes	Yes	Yes	Yes	Yes	Yes	Yes	Yes
**INCREASE IN HOUSEHOLD CONFLICTS**	Yes	Yes	Yes	Yes	Yes	Yes	Yes	Yes	Yes	Yes
**REDUCED EDUCATION**	Yes	Yes	Yes	Yes	Yes	Yes	Yes	Yes	Yes	Yes
**STRESS AND MENTAL HEALTH PROBLEMS**	Yes	Yes	Yes	Yes	Yes	Yes	Yes	Yes	Yes	Yes
**INCREASED ILLNESS/DEATHS**	Yes	Yes	Yes	Yes	Yes	Yes	Yes	Yes	Yes	Yes
**HUNGER AND LACK OF FOOD**	Yes	Yes	Yes	Yes	Yes	Yes	Yes	Yes	Yes	Yes
**REDUCED RELIGIOUS ACTIVITIES**	Yes	Yes	Yes	Yes	Yes	Yes	Yes	Yes	Yes	Yes
**BAD EXPERIENCE OF COVID-19 PANDEMIC**	Yes	Yes	Yes	Yes	Yes	Yes	Yes	Yes	Yes	Yes
INCREASE IN SOCIAL VICES	Yes	Yes	Yes	Yes	Yes	Yes	Yes	Yes	-	Yes
LACK OF ACCESS TO MONEY	Yes	Yes	Yes	Yes	Yes	Yes	Yes	Yes	-	Yes
PENALTIES/PUNISHMENT	Yes	Yes	Yes	Yes	Yes	Yes	Yes	Yes	-	Yes
CONFLICT BETWEEN GOVERNMENT AND CITIZENS	Yes	-	Yes	Yes	-	-	Yes	Yes	-	-
ANIMAL/HERDERS PROBLEMS INCREASED	Yes	-	Yes	Yes	Yes	Yes	Yes	-	-	-
REDUCED GOVERNMENT SUPPORT	Yes	Yes	Yes	Yes	Yes	Yes	Yes	Yes	Yes	-
INCREASE IN EARLY MARRIAGE	Yes	-	Yes	Yes	-	-	Yes	-	-	-
LACK OF COVID-19 COMMODITIES	Yes	-	Yes	-	Yes	Yes	Yes	-	-	-
PRICE HIKE	Yes	Yes	Yes	Yes	Yes	Yes	Yes	Yes	Yes	-
REDUCED PHYSICAL FITNESS	Yes	-	Yes	Yes	-	-	Yes	Yes	-	-
ENFORCED SOCIAL DISTANCE	Yes	Yes	Yes	Yes	Yes	Yes	Yes	Yes	Yes	-
FAULTY CLINICAL ISOLATION POLICIES	Yes	Yes	Yes	Yes	Yes	Yes	Yes	Yes	-	-

Legend: P = position in the ranking of the five strongest influences. W = Cumulative net influence. Com. = Community maps. High L. = Maps of wards, LGA and state levels. PPE= Personal protective equipment. Bolded categories were mentioned across all the stakeholder groups.

For Peer Review

Supplementary material 5. Number of new nodes in each map by group

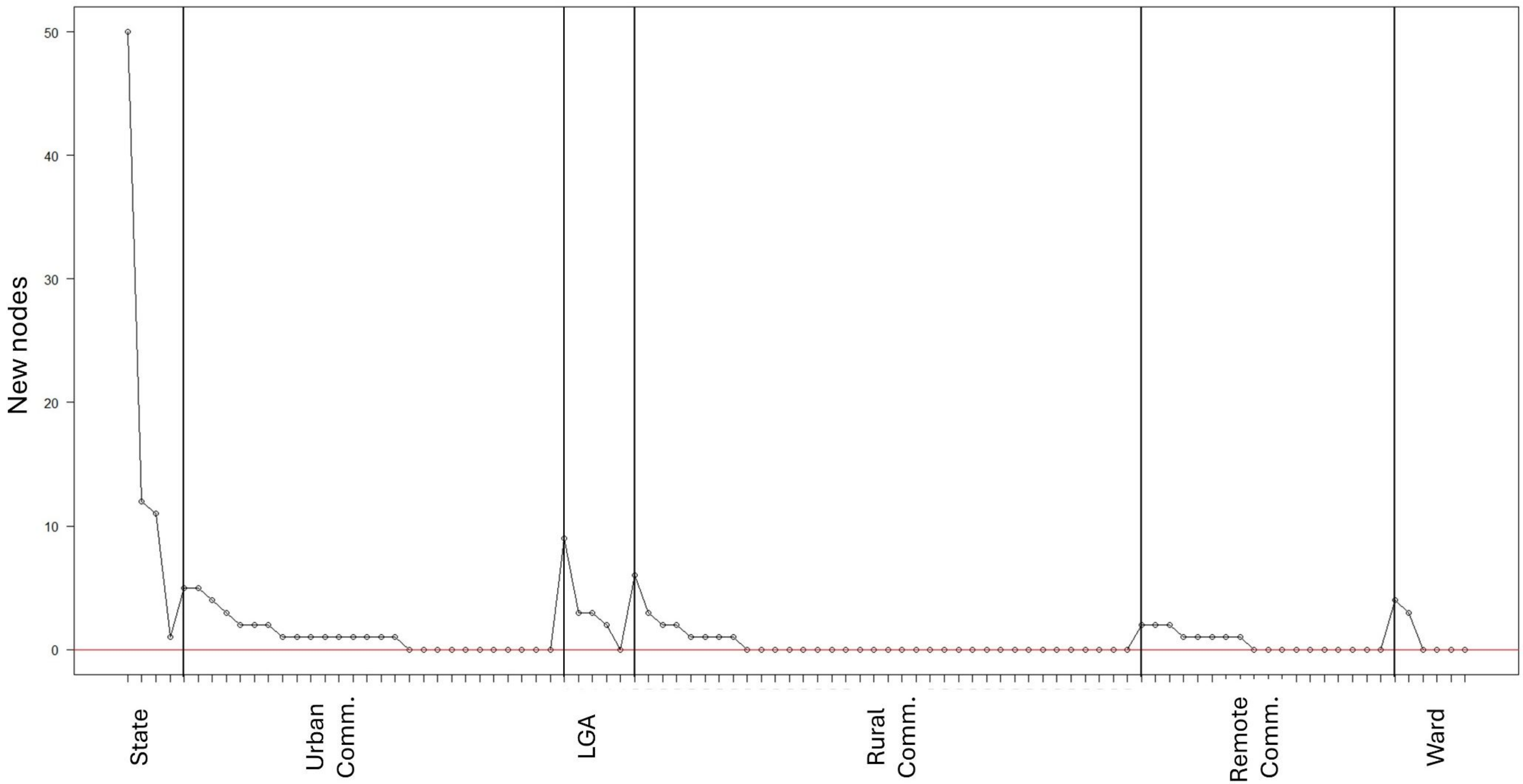

Supplementary Material 6. Adjacency matrices of category maps by participant groups

Legend. The tables in each sheet illustrate the relationships between categories for the overall maps and each stakeholder group. The format is an adjacency matrix depicting relationships from row to column nodes. Cell values are the relationship's weight.

Group
All maps
All community members
People living with disabilities
Female participants
Internally displaced persons
Male participants
Remote communities
Rural communities
Urban communities

For Peer Review

Sheet
All maps
Communities
Disabled
Female
IDPS
Male
Remote
Rural
Urban

For Peer Review

Category	Code		K01	K02	K03	K04	K05	K06
BAD EXPERIENCE OF COVID-19 PANDEMIC	K01		0.000	0.00	0.00	0.00	0.00	0.00
INCREASE IN SOCIAL VICES	K02		0.114	0.07	0.00	0.00	0.00	0.00
LACK OF ACCESS TO MONEY	K03		0.137	0.00	0.00	0.00	0.00	0.01
LOCKDOWN	K04	4	0.337	0.13	0.17	0.29	0.55	0.15
REDUCED BUSINESSES/JOBS	K05	2	0.401	0.03	0.21	0.00	0.24	0.02
REDUCED SOCIAL CONTACT/ACTIVITIES	K06		0.154	0.00	0.00	0.00	0.01	0.04
REDUCED HEALTH SERVICES	K07		0.166	0.00	0.00	0.00	0.00	0.01
PROBLEMS WITH PPE	K08		0.130	0.00	0.00	0.00	0.00	0.00
FEAR AND MISCONCEPTION OF COVID-19	K09		0.229	0.01	0.00	0.00	0.01	0.07
INCREASE IN HOUSEHOLD CONFLICTS	K10	3	0.351	0.11	0.01	0.00	0.01	0.00
REDUCED EDUCATION	K11		0.286	0.12	0.00	0.00	0.00	0.00
PENALTIES/PUNISHMENT	K12		0.018	0.00	0.00	0.00	0.00	0.00
CONFLICT BETWEEN GOVERNMENT AND CITIZ	K13		0.002	0.00	0.00	0.00	0.00	0.00
ANIMAL/HERDERS PROBLEMS INCREASED	K14		0.013	0.00	0.01	0.00	0.00	0.00
STRESS AND MENTAL HEALTH PROBLEMS	K15	5	0.300	0.00	0.00	0.00	0.00	0.00
INCREASED ILLNESS/DEATH	K16		0.188	0.00	0.00	0.00	0.00	0.00
REDUCED GOVERNMENT SUPPORT	K17		0.023	0.00	0.00	0.00	0.00	0.00
INCREASE IN EARLY MARRIAGE	K18		0.002	0.00	0.00	0.00	0.00	0.00
HUNGER AND LACK OF FOOD	K19	1	0.635	0.02	0.00	0.00	0.01	0.01
LACK OF COVID-19 COMMODITIES	K20		0.003	0.00	0.00	0.00	0.00	0.00
REDUCED RELIGIOUS ACTIVITIES	K21		0.275	0.01	0.02	0.00	0.00	0.02
PRICE HIKE	K22		0.069	0.00	0.00	0.00	0.01	0.00
REDUCED PHYSICAL FITNESS	K23		0.003	0.00	0.00	0.00	0.00	0.00
ENFORCED SOCIAL DISTANCE	K24		0.087	0.01	0.01	0.00	0.01	0.08
FAULTY CLINICAL ISOLATION POLICIES	K25		0.019	0.00	0.00	0.00	0.00	0.00
	K26		0.000	0.00	0.00	0.00	0.00	0.00
Indegree			3.94	0.52	0.44	0.30	0.85	0.41
Relative			1.00	0.13	0.11	0.08	0.22	0.10
Rank			1	9	10	13	6	11

For Peer Review

K07	K08	K09	K10	K11	K12	K13	K14	K15	K16	K17	K18	K19	K20	K21	K22	K23
0.00	0.00	0.00	0.00	0.00	0.00	0.00	0.00	0.00	0.00	0.00	0.00	0.00	0.00	0.00	0.00	0.00
0.00	0.00	0.00	0.01	0.00	0.02	0.00	0.00	0.03	0.03	0.00	0.00	0.00	0.00	0.00	0.00	0.00
0.00	0.00	0.00	0.02	0.00	0.00	0.00	0.00	0.04	0.02	0.00	0.00	0.22	0.00	0.00	0.00	0.00
0.16	0.00	0.02	0.52	0.36	0.03	0.00	0.02	0.30	0.22	0.00	0.00	0.93	0.00	0.36	0.11	0.00
0.03	0.00	0.00	0.32	0.00	0.00	0.00	0.00	0.08	0.11	0.00	0.00	1.00	0.00	0.00	0.06	0.00
0.01	0.01	0.00	0.01	0.00	0.00	0.00	0.00	0.15	0.02	0.00	0.00	0.03	0.00	0.02	0.00	0.00
0.07	0.00	0.00	0.00	0.00	0.00	0.00	0.00	0.01	0.18	0.00	0.00	0.00	0.00	0.00	0.00	0.00
0.01	0.08	0.00	0.00	0.00	0.00	0.00	0.00	0.01	0.02	0.00	0.00	0.01	0.00	0.00	0.00	0.00
0.08	0.12	0.19	0.00	0.00	0.00	0.00	0.00	0.08	0.07	0.00	0.00	0.02	0.00	0.01	0.00	0.00
0.00	0.00	0.00	0.24	0.07	0.01	0.00	0.00	0.13	0.06	0.00	0.00	0.11	0.00	0.00	0.00	0.00
0.00	0.00	0.00	0.18	0.25	0.01	0.00	0.00	0.15	0.02	0.00	0.00	0.02	0.00	0.00	0.00	0.00
0.00	0.00	0.00	0.00	0.00	0.00	0.00	0.00	0.00	0.00	0.00	0.00	0.00	0.00	0.00	0.00	0.00
0.00	0.00	0.00	0.00	0.00	0.00	0.00	0.00	0.00	0.00	0.00	0.00	0.00	0.00	0.00	0.00	0.00
0.00	0.00	0.00	0.01	0.00	0.00	0.00	0.00	0.01	0.00	0.00	0.00	0.02	0.00	0.00	0.00	0.00
0.00	0.00	0.00	0.01	0.00	0.00	0.00	0.00	0.07	0.01	0.00	0.00	0.01	0.00	0.00	0.00	0.00
0.00	0.00	0.00	0.00	0.00	0.00	0.00	0.00	0.01	0.01	0.00	0.00	0.00	0.00	0.00	0.00	0.00
0.00	0.00	0.00	0.00	0.00	0.00	0.00	0.00	0.01	0.01	0.00	0.00	0.02	0.00	0.00	0.00	0.00
0.00	0.00	0.00	0.00	0.00	0.00	0.00	0.00	0.00	0.00	0.00	0.00	0.00	0.00	0.00	0.00	0.00
0.01	0.00	0.00	0.22	0.00	0.00	0.00	0.00	0.05	0.19	0.00	0.00	0.54	0.00	0.00	0.05	0.00
0.00	0.00	0.00	0.00	0.00	0.00	0.00	0.00	0.00	0.00	0.00	0.00	0.00	0.00	0.00	0.00	0.00
0.00	0.00	0.00	0.00	0.00	0.00	0.00	0.00	0.30	0.02	0.00	0.00	0.03	0.00	0.16	0.00	0.00
0.00	0.00	0.00	0.03	0.00	0.00	0.00	0.00	0.00	0.03	0.00	0.00	0.14	0.00	0.00	0.00	0.00
0.00	0.00	0.00	0.00	0.00	0.00	0.00	0.00	0.00	0.00	0.00	0.00	0.00	0.00	0.00	0.00	0.00
0.00	0.00	0.00	0.01	0.01	0.00	0.00	0.00	0.12	0.01	0.00	0.00	0.03	0.00	0.07	0.01	0.00
0.01	0.00	0.00	0.00	0.00	0.00	0.00	0.00	0.00	0.01	0.00	0.00	0.00	0.00	0.00	0.00	0.00
0.00	0.00	0.00	0.00	0.00	0.00	0.00	0.00	0.00	0.00	0.00	0.00	0.00	0.00	0.00	0.00	0.00
0.40	0.23	0.22	1.59	0.70	0.08	0.01	0.03	1.57	1.03	0.02	0.01	3.12	0.00	0.63	0.22	0.01
0.10	0.06	0.06	0.40	0.18	0.02	0.00	0.01	0.40	0.26	0.00	0.00	0.79	0.00	0.16	0.06	0.00
12	14	16	3	7	18	24	20	4	5	21	23	2	25	8	15	22

For Peer Review

K24	K25	K26
0.00	0.00	0.00
0.00	0.00	0.00
0.00	0.00	0.00
0.05	0.00	0.00
0.00	0.00	0.00
0.00	0.00	0.00
0.00	0.01	0.00
0.00	0.00	0.00
0.02	0.01	0.00
0.00	0.00	0.00
0.00	0.00	0.00
0.00	0.00	0.00
0.00	0.00	0.00
0.00	0.00	0.00
0.00	0.00	0.00
0.00	0.00	0.00
0.00	0.00	0.00
0.00	0.00	0.00
0.00	0.00	0.00
0.00	0.00	0.00
0.02	0.00	0.00
0.00	0.00	0.00
0.00	0.00	0.00
0.00	0.00	0.00
0.00	0.00	0.00
0.00	0.00	0.00

0.10	0.03	0.00
0.02	0.01	0.00
17	19	26

Outdegree	Relative	Rank
0.00	0.00	25
0.28	0.06	14
0.47	0.10	8
4.70	1.00	1
2.51	0.53	2
0.45	0.10	10
0.45	0.10	11
0.26	0.06	15
0.93	0.20	6
1.10	0.23	4
1.06	0.22	5
0.03	0.01	20
0.01	0.00	22
0.07	0.01	18
0.41	0.09	12
0.22	0.05	16
0.08	0.02	17
0.00	0.00	24
1.74	0.37	3
0.01	0.00	21
0.86	0.18	7
0.29	0.06	13
0.01	0.00	23
0.45	0.10	9
0.05	0.01	19
0.00	0.00	25

For Peer Review

Category	Code		K01	K02	K03	K04	K05	K06
BAD EXPERIENCE OF COVID-19 PANDEMIC	K01		0.000	0.00	0.00	0.00	0.00	0.00
INCREASE IN SOCIAL VICES	K02		0.095	0.05	0.00	0.00	0.00	0.00
LACK OF ACCESS TO MONEY	K03		0.134	0.00	0.00	0.00	0.00	0.00
LOCKDOWN	K04	4	0.326	0.10	0.16	0.27	0.55	0.15
REDUCED BUSINESSES/JOBS	K05	2	0.403	0.02	0.22	0.00	0.24	0.01
REDUCED SOCIAL CONTACT/ACTIVITIES	K06		0.143	0.00	0.00	0.00	0.01	0.03
REDUCED HEALTH SERVICES	K07		0.164	0.00	0.00	0.00	0.00	0.01
PROBLEMS WITH PPE	K08		0.123	0.00	0.00	0.00	0.00	0.00
FEAR AND MISCONCEPTION OF COVID-19	K09		0.204	0.01	0.00	0.00	0.01	0.06
INCREASE IN HOUSEHOLD CONFLICTS	K10	3	0.327	0.08	0.01	0.00	0.01	0.00
REDUCED EDUCATION	K11		0.277	0.10	0.00	0.00	0.01	0.01
PENALTIES/PUNISHMENT	K12		0.018	0.00	0.00	0.00	0.00	0.00
CONFLICT BETWEEN GOVERNMENT AND CITIZ	K13		0.001	0.00	0.00	0.00	0.00	0.00
ANIMAL/HERDERS PROBLEMS INCREASED	K14		0.015	0.00	0.01	0.00	0.00	0.00
STRESS AND MENTAL HEALTH PROBLEMS	K15	5	0.280	0.00	0.00	0.00	0.00	0.00
INCREASED ILLNESS/DEATH	K16		0.181	0.00	0.00	0.00	0.00	0.00
REDUCED GOVERNMENT SUPPORT	K17		0.019	0.00	0.00	0.00	0.00	0.00
INCREASE IN EARLY MARRIAGE	K18		0.002	0.00	0.00	0.00	0.00	0.00
HUNGER AND LACK OF FOOD	K19	1	0.615	0.01	0.01	0.00	0.01	0.01
LACK OF COVID-19 COMMODITIES	K20		0.004	0.00	0.00	0.00	0.00	0.00
REDUCED RELIGIOUS ACTIVITIES	K21		0.263	0.00	0.02	0.00	0.00	0.02
PRICE HIKE	K22		0.064	0.00	0.00	0.00	0.01	0.00
REDUCED PHYSICAL FITNESS	K23		0.002	0.00	0.00	0.00	0.00	0.00
ENFORCED SOCIAL DISTANCE	K24		0.086	0.00	0.01	0.01	0.01	0.07
FAULTY CLINICAL ISOLATION POLICIES	K25		0.013	0.00	0.00	0.00	0.00	0.00
	K26		0.000	0.00	0.00	0.00	0.00	0.00
Indegree			3.75	0.38	0.44	0.28	0.86	0.38
Relative			1.00	0.10	0.12	0.07	0.23	0.10
Rank								

For Peer Review

K07	K08	K09	K10	K11	K12	K13	K14	K15	K16	K17	K18	K19	K20	K21	K22	K23
0.00	0.00	0.00	0.00	0.00	0.00	0.00	0.00	0.00	0.00	0.00	0.00	0.00	0.00	0.00	0.00	0.00
0.00	0.00	0.00	0.01	0.00	0.02	0.00	0.00	0.02	0.02	0.00	0.00	0.00	0.00	0.00	0.00	0.00
0.00	0.00	0.00	0.02	0.00	0.00	0.00	0.00	0.04	0.02	0.00	0.00	0.22	0.00	0.00	0.00	0.00
0.15	0.01	0.02	0.46	0.36	0.02	0.00	0.03	0.28	0.21	0.00	0.00	0.85	0.00	0.32	0.09	0.00
0.03	0.00	0.00	0.27	0.01	0.00	0.00	0.00	0.08	0.11	0.00	0.00	1.00	0.00	0.00	0.05	0.00
0.00	0.01	0.00	0.00	0.00	0.00	0.00	0.00	0.14	0.02	0.00	0.00	0.02	0.00	0.01	0.00	0.00
0.07	0.00	0.00	0.00	0.00	0.00	0.00	0.00	0.01	0.17	0.00	0.00	0.00	0.00	0.00	0.00	0.00
0.00	0.07	0.00	0.00	0.00	0.01	0.00	0.00	0.01	0.02	0.00	0.00	0.00	0.00	0.00	0.00	0.00
0.06	0.10	0.16	0.00	0.00	0.00	0.00	0.00	0.07	0.06	0.00	0.00	0.01	0.00	0.01	0.00	0.00
0.00	0.00	0.00	0.21	0.06	0.01	0.00	0.00	0.13	0.05	0.00	0.00	0.09	0.00	0.00	0.00	0.00
0.00	0.00	0.00	0.17	0.25	0.01	0.00	0.00	0.15	0.02	0.00	0.00	0.02	0.00	0.00	0.00	0.00
0.00	0.00	0.00	0.00	0.00	0.00	0.00	0.00	0.00	0.00	0.00	0.00	0.00	0.00	0.00	0.00	0.00
0.00	0.00	0.00	0.00	0.00	0.00	0.00	0.00	0.00	0.00	0.00	0.00	0.00	0.00	0.00	0.00	0.00
0.00	0.00	0.00	0.01	0.00	0.00	0.00	0.01	0.01	0.00	0.00	0.00	0.03	0.00	0.00	0.00	0.00
0.00	0.00	0.00	0.01	0.00	0.00	0.00	0.00	0.06	0.01	0.00	0.00	0.00	0.00	0.00	0.00	0.00
0.00	0.00	0.00	0.00	0.00	0.00	0.00	0.00	0.01	0.01	0.00	0.00	0.00	0.00	0.00	0.00	0.00
0.00	0.00	0.00	0.00	0.00	0.00	0.00	0.00	0.01	0.01	0.00	0.00	0.02	0.00	0.00	0.00	0.00
0.00	0.00	0.00	0.00	0.00	0.00	0.00	0.00	0.00	0.00	0.00	0.00	0.00	0.00	0.00	0.00	0.00
0.01	0.00	0.00	0.18	0.00	0.00	0.00	0.00	0.05	0.19	0.00	0.00	0.52	0.00	0.00	0.04	0.00
0.00	0.00	0.00	0.00	0.00	0.00	0.00	0.00	0.00	0.00	0.00	0.00	0.00	0.00	0.00	0.00	0.00
0.00	0.00	0.00	0.00	0.00	0.00	0.00	0.00	0.29	0.01	0.00	0.00	0.02	0.00	0.15	0.00	0.00
0.00	0.00	0.00	0.02	0.00	0.00	0.00	0.00	0.01	0.03	0.00	0.00	0.13	0.00	0.00	0.00	0.00
0.00	0.00	0.00	0.00	0.00	0.00	0.00	0.00	0.00	0.00	0.00	0.00	0.00	0.00	0.00	0.00	0.00
0.00	0.00	0.00	0.01	0.01	0.00	0.00	0.00	0.11	0.01	0.00	0.00	0.04	0.00	0.07	0.01	0.00
0.00	0.00	0.00	0.00	0.00	0.00	0.00	0.00	0.00	0.01	0.00	0.00	0.00	0.00	0.00	0.00	0.00
0.00	0.00	0.00	0.00	0.00	0.00	0.00	0.00	0.00	0.00	0.00	0.00	0.00	0.00	0.00	0.00	0.00

0.35	0.20	0.19	1.38	0.68	0.08	0.00	0.03	1.45	0.96	0.01	0.01	2.98	0.01	0.57	0.19	0.01
0.09	0.05	0.05	0.37	0.18	0.02	0.00	0.01	0.39	0.26	0.00	0.00	0.79	0.00	0.15	0.05	0.00

For Peer Review

K24	K25	K26
0.00	0.00	0.00
0.00	0.00	0.00
0.00	0.00	0.00
0.06	0.00	0.00
0.00	0.00	0.00
0.00	0.00	0.00
0.00	0.01	0.00
0.00	0.00	0.00
0.02	0.01	0.00
0.00	0.00	0.00
0.00	0.00	0.00
0.00	0.00	0.00
0.00	0.00	0.00
0.00	0.00	0.00
0.00	0.00	0.00
0.00	0.00	0.00
0.00	0.00	0.00
0.00	0.00	0.00
0.00	0.00	0.00
0.00	0.00	0.00
0.02	0.00	0.00
0.00	0.00	0.00
0.00	0.00	0.00
0.00	0.00	0.00
0.00	0.00	0.00
0.00	0.00	0.00

0.10	0.02	0.00
0.03	0.01	0.00

Outdegree	
0.00	0.00
0.22	0.05
0.46	0.10
4.41	1.00
2.45	0.56
0.40	0.09
0.43	0.10
0.24	0.05
0.80	0.18
0.96	0.22
1.03	0.23
0.02	0.01
0.01	0.00
0.08	0.02
0.36	0.08
0.21	0.05
0.08	0.02
0.00	0.00
1.64	0.37
0.01	0.00
0.80	0.18
0.26	0.06
0.00	0.00
0.42	0.10
0.03	0.01
0.00	0.00

For Peer Review

Category	Code		K01	K02	K03	K04	K05	K06
BAD EXPERIENCE OF COVID-19 PANDEMIC	K01		0.000	0.00	0.00	0.00	0.00	0.00
INCREASE IN SOCIAL VICES	K02		0.000	0.00	0.00	0.00	0.00	0.00
LACK OF ACCESS TO MONEY	K03		0.000	0.00	0.00	0.00	0.00	0.00
LOCKDOWN	K04	2	0.247	0.00	0.00	0.23	0.18	0.32
REDUCED BUSINESSES/JOBS	K05		0.123	0.00	0.00	0.00	0.00	0.07
REDUCED SOCIAL CONTACT/ACTIVITIES	K06	4	0.233	0.00	0.00	0.00	0.04	0.05
REDUCED HEALTH SERVICES	K07		0.123	0.00	0.00	0.00	0.00	0.00
PROBLEMS WITH PPE	K08	4	0.233	0.00	0.00	0.00	0.00	0.00
FEAR AND MISCONCEPTION OF COVID-19	K09	2	0.247	0.00	0.00	0.00	0.00	0.10
INCREASE IN HOUSEHOLD CONFLICTS	K10		0.192	0.00	0.00	0.00	0.04	0.00
REDUCED EDUCATION	K11		0.178	0.00	0.00	0.00	0.00	0.00
PENALTIES/PUNISHMENT	K12		0.000	0.00	0.00	0.00	0.00	0.00
CONFLICT BETWEEN GOVERNMENT AND CITIZ	K13		0.000	0.00	0.00	0.00	0.00	0.00
ANIMAL/HERDERS PROBLEMS INCREASED	K14		0.000	0.00	0.00	0.00	0.00	0.00
STRESS AND MENTAL HEALTH PROBLEMS	K15		0.219	0.00	0.00	0.00	0.00	0.00
INCREASED ILLNESS/DEATH	K16		0.082	0.00	0.00	0.00	0.00	0.00
REDUCED GOVERNMENT SUPPORT	K17		0.096	0.00	0.00	0.00	0.00	0.07
INCREASE IN EARLY MARRIAGE	K18		0.000	0.00	0.00	0.00	0.00	0.00
HUNGER AND LACK OF FOOD	K19	1	0.370	0.00	0.00	0.00	0.00	0.04
LACK OF COVID-19 COMMODITIES	K20		0.000	0.00	0.00	0.00	0.00	0.00
REDUCED RELIGIOUS ACTIVITIES	K21		0.192	0.00	0.00	0.00	0.00	0.11
PRICE HIKE	K22		0.041	0.00	0.00	0.00	0.00	0.00
REDUCED PHYSICAL FITNESS	K23		0.000	0.00	0.00	0.00	0.00	0.00
ENFORCED SOCIAL DISTANCE	K24		0.082	0.00	0.00	0.00	0.00	0.11
FAULTY CLINICAL ISOLATION POLICIES	K25		0.000	0.00	0.00	0.00	0.00	0.00
	K26		0.000	0.00	0.00	0.00	0.00	0.00

	K01	K02	K03	K04	K05	K06
Indegree	2.66	0.00	0.00	0.23	0.26	0.86
Relative	0.97	0.00	0.00	0.09	0.10	0.32
Rank						

For Peer Review

K07	K08	K09	K10	K11	K12	K13	K14	K15	K16	K17	K18	K19	K20	K21	K22	K23
0.00	0.00	0.00	0.00	0.00	0.00	0.00	0.00	0.00	0.00	0.00	0.00	0.00	0.00	0.00	0.00	0.00
0.00	0.00	0.00	0.00	0.00	0.00	0.00	0.00	0.00	0.00	0.00	0.00	0.00	0.00	0.00	0.00	0.00
0.00	0.00	0.00	0.00	0.00	0.00	0.00	0.00	0.00	0.00	0.00	0.00	0.00	0.00	0.00	0.00	0.00
0.27	0.00	0.00	0.53	0.20	0.00	0.00	0.00	0.21	0.19	0.00	0.00	1.00	0.00	0.23	0.11	0.00
0.05	0.00	0.00	0.08	0.00	0.00	0.00	0.00	0.01	0.07	0.00	0.00	0.38	0.00	0.00	0.04	0.00
0.11	0.04	0.00	0.00	0.00	0.00	0.00	0.00	0.14	0.10	0.00	0.00	0.32	0.00	0.00	0.00	0.00
0.04	0.00	0.00	0.00	0.00	0.00	0.00	0.00	0.00	0.16	0.00	0.00	0.00	0.00	0.00	0.00	0.00
0.05	0.10	0.00	0.00	0.00	0.00	0.00	0.00	0.08	0.08	0.00	0.00	0.07	0.00	0.00	0.00	0.00
0.11	0.23	0.19	0.00	0.00	0.00	0.00	0.00	0.11	0.10	0.00	0.00	0.16	0.00	0.00	0.00	0.00
0.05	0.00	0.00	0.15	0.04	0.00	0.00	0.00	0.10	0.03	0.00	0.00	0.21	0.00	0.00	0.00	0.00
0.00	0.00	0.00	0.05	0.13	0.00	0.00	0.00	0.08	0.00	0.00	0.00	0.00	0.00	0.00	0.00	0.00
0.00	0.00	0.00	0.00	0.00	0.00	0.00	0.00	0.00	0.00	0.00	0.00	0.00	0.00	0.00	0.00	0.00
0.00	0.00	0.00	0.00	0.00	0.00	0.00	0.00	0.00	0.00	0.00	0.00	0.00	0.00	0.00	0.00	0.00
0.00	0.00	0.00	0.00	0.00	0.00	0.00	0.00	0.00	0.00	0.00	0.00	0.00	0.00	0.00	0.00	0.00
0.00	0.00	0.00	0.07	0.00	0.00	0.00	0.00	0.12	0.11	0.00	0.00	0.00	0.00	0.00	0.00	0.00
0.00	0.00	0.00	0.00	0.00	0.00	0.00	0.00	0.00	0.00	0.00	0.00	0.00	0.00	0.00	0.00	0.00
0.00	0.00	0.00	0.00	0.00	0.00	0.00	0.00	0.00	0.03	0.00	0.00	0.10	0.00	0.00	0.00	0.00
0.00	0.00	0.00	0.00	0.00	0.00	0.00	0.00	0.00	0.00	0.00	0.00	0.00	0.00	0.00	0.00	0.00
0.11	0.00	0.00	0.33	0.00	0.00	0.00	0.00	0.04	0.26	0.00	0.00	0.26	0.00	0.00	0.05	0.00
0.00	0.00	0.00	0.00	0.00	0.00	0.00	0.00	0.00	0.00	0.00	0.00	0.00	0.00	0.00	0.00	0.00
0.11	0.00	0.00	0.00	0.00	0.00	0.00	0.00	0.11	0.11	0.00	0.00	0.16	0.00	0.14	0.00	0.00
0.00	0.00	0.00	0.08	0.00	0.00	0.00	0.00	0.00	0.04	0.00	0.00	0.08	0.00	0.00	0.00	0.00
0.00	0.00	0.00	0.00	0.00	0.00	0.00	0.00	0.00	0.00	0.00	0.00	0.00	0.00	0.00	0.00	0.00
0.00	0.04	0.00	0.00	0.00	0.00	0.00	0.00	0.18	0.05	0.00	0.00	0.00	0.00	0.00	0.00	0.00
0.00	0.00	0.00	0.00	0.00	0.00	0.00	0.00	0.00	0.00	0.00	0.00	0.00	0.00	0.00	0.00	0.00
0.00	0.00	0.00	0.00	0.00	0.00	0.00	0.00	0.00	0.00	0.00	0.00	0.00	0.00	0.00	0.00	0.00

0.92	0.41	0.19	1.30	0.37	0.00	0.00	0.00	1.18	1.33	0.00	0.00	2.74	0.00	0.37	0.21	0.00
0.34	0.15	0.07	0.48	0.14	0.00	0.00	0.00	0.43	0.49	0.00	0.00	1.00	0.00	0.14	0.08	0.00

For Peer Review

K24	K25	K26
0.00	0.00	0.00
0.00	0.00	0.00
0.00	0.00	0.00
0.00	0.00	0.00
0.00	0.00	0.00
0.00	0.00	0.00
0.00	0.00	0.00
0.00	0.00	0.00
0.04	0.00	0.00
0.00	0.00	0.00
0.00	0.00	0.00
0.00	0.00	0.00
0.00	0.00	0.00
0.00	0.00	0.00
0.00	0.00	0.00
0.00	0.00	0.00
0.00	0.00	0.00
0.00	0.00	0.00
0.00	0.00	0.00
0.00	0.00	0.00
0.00	0.00	0.00
0.00	0.00	0.00
0.00	0.00	0.00
0.00	0.00	0.00
0.00	0.00	0.00
0.00	0.00	0.00
0.04	0.00	0.00
0.02	0.00	0.00

Outdegree	
0.00	0.00
0.00	0.00
0.00	0.00
3.72	1.00
0.84	0.22
1.03	0.28
0.33	0.09
0.62	0.17
1.29	0.35
0.81	0.22
0.45	0.12
0.00	0.00
0.00	0.00
0.00	0.00
0.52	0.14
0.08	0.02
0.29	0.08
0.00	0.00
1.47	0.39
0.00	0.00
0.93	0.25
0.25	0.07
0.00	0.00
0.47	0.13
0.00	0.00
0.00	0.00

For Peer Review

Category	Code		K01	K02	K03	K04	K05	K06
BAD EXPERIENCE OF COVID-19 PANDEMIC	K01		0.000	0.00	0.00	0.00	0.00	0.00
INCREASE IN SOCIAL VICES	K02		0.036	0.03	0.00	0.00	0.00	0.00
LACK OF ACCESS TO MONEY	K03		0.115	0.00	0.00	0.00	0.00	0.01
LOCKDOWN	K04	4	0.310	0.04	0.14	0.29	0.50	0.20
REDUCED BUSINESSES/JOBS	K05	2	0.364	0.01	0.22	0.00	0.21	0.02
REDUCED SOCIAL CONTACT/ACTIVITIES	K06		0.141	0.00	0.00	0.00	0.01	0.04
REDUCED HEALTH SERVICES	K07		0.181	0.00	0.00	0.00	0.00	0.01
PROBLEMS WITH PPE	K08		0.097	0.00	0.00	0.00	0.00	0.00
FEAR AND MISCONCEPTION OF COVID-19	K09		0.216	0.00	0.00	0.00	0.00	0.03
INCREASE IN HOUSEHOLD CONFLICTS	K10	3	0.327	0.05	0.00	0.00	0.01	0.00
REDUCED EDUCATION	K11		0.223	0.04	0.00	0.00	0.00	0.00
PENALTIES/PUNISHMENT	K12		0.002	0.00	0.00	0.00	0.00	0.00
CONFLICT BETWEEN GOVERNMENT AND CITIZ	K13		0.000	0.00	0.00	0.00	0.00	0.00
ANIMAL/HERDERS PROBLEMS INCREASED	K14		0.000	0.00	0.00	0.00	0.00	0.00
STRESS AND MENTAL HEALTH PROBLEMS	K15	5	0.247	0.00	0.00	0.00	0.00	0.00
INCREASED ILLNESS/DEATH	K16		0.148	0.00	0.00	0.00	0.00	0.00
REDUCED GOVERNMENT SUPPORT	K17		0.004	0.00	0.00	0.00	0.00	0.00
INCREASE IN EARLY MARRIAGE	K18		0.000	0.00	0.00	0.00	0.00	0.00
HUNGER AND LACK OF FOOD	K19	1	0.543	0.01	0.01	0.00	0.00	0.02
LACK OF COVID-19 COMMODITIES	K20		0.000	0.00	0.00	0.00	0.00	0.00
REDUCED RELIGIOUS ACTIVITIES	K21		0.203	0.00	0.02	0.00	0.00	0.01
PRICE HIKE	K22		0.047	0.00	0.00	0.00	0.01	0.00
REDUCED PHYSICAL FITNESS	K23		0.000	0.00	0.00	0.00	0.00	0.00
ENFORCED SOCIAL DISTANCE	K24		0.060	0.00	0.01	0.01	0.01	0.07
FAULTY CLINICAL ISOLATION POLICIES	K25		0.013	0.00	0.00	0.00	0.00	0.00
	K26		0.000	0.00	0.00	0.00	0.00	0.00

	K01	K02	K03	K04	K05	K06
Indegree	3.28	0.17	0.40	0.30	0.75	0.42
Relative	1.00	0.05	0.12	0.09	0.23	0.13
Rank						

For Peer Review

K07	K08	K09	K10	K11	K12	K13	K14	K15	K16	K17	K18	K19	K20	K21	K22	K23
0.00	0.00	0.00	0.00	0.00	0.00	0.00	0.00	0.00	0.00	0.00	0.00	0.00	0.00	0.00	0.00	0.00
0.00	0.00	0.00	0.00	0.00	0.01	0.00	0.00	0.01	0.01	0.00	0.00	0.00	0.00	0.00	0.00	0.00
0.01	0.00	0.00	0.01	0.00	0.00	0.00	0.00	0.03	0.03	0.00	0.00	0.23	0.00	0.00	0.00	0.00
0.18	0.00	0.01	0.49	0.34	0.00	0.00	0.00	0.31	0.21	0.00	0.00	0.83	0.00	0.27	0.08	0.00
0.06	0.00	0.00	0.23	0.00	0.00	0.00	0.00	0.08	0.18	0.00	0.00	1.00	0.00	0.00	0.04	0.00
0.01	0.00	0.00	0.00	0.00	0.00	0.00	0.00	0.14	0.02	0.00	0.00	0.05	0.00	0.00	0.00	0.00
0.10	0.00	0.00	0.00	0.00	0.00	0.00	0.00	0.02	0.20	0.00	0.00	0.00	0.00	0.00	0.00	0.00
0.01	0.07	0.00	0.00	0.00	0.00	0.00	0.00	0.01	0.01	0.00	0.00	0.01	0.00	0.00	0.00	0.00
0.08	0.06	0.19	0.00	0.00	0.00	0.00	0.00	0.05	0.05	0.00	0.00	0.02	0.00	0.00	0.00	0.00
0.00	0.00	0.00	0.25	0.06	0.00	0.00	0.00	0.18	0.05	0.00	0.00	0.11	0.00	0.00	0.00	0.00
0.00	0.00	0.01	0.21	0.19	0.00	0.00	0.00	0.19	0.01	0.00	0.00	0.02	0.00	0.00	0.00	0.00
0.00	0.00	0.00	0.00	0.00	0.00	0.00	0.00	0.00	0.00	0.00	0.00	0.00	0.00	0.00	0.00	0.00
0.00	0.00	0.00	0.00	0.00	0.00	0.00	0.00	0.00	0.00	0.00	0.00	0.00	0.00	0.00	0.00	0.00
0.00	0.00	0.00	0.00	0.00	0.00	0.00	0.00	0.00	0.00	0.00	0.00	0.00	0.00	0.00	0.00	0.00
0.00	0.01	0.00	0.02	0.00	0.00	0.00	0.00	0.03	0.00	0.00	0.00	0.01	0.00	0.00	0.00	0.00
0.00	0.00	0.00	0.00	0.00	0.00	0.00	0.00	0.01	0.00	0.00	0.00	0.00	0.00	0.00	0.00	0.00
0.00	0.00	0.00	0.00	0.00	0.00	0.00	0.00	0.00	0.00	0.00	0.00	0.01	0.00	0.00	0.00	0.00
0.00	0.00	0.00	0.00	0.00	0.00	0.00	0.00	0.00	0.00	0.00	0.00	0.00	0.00	0.00	0.00	0.00
0.03	0.00	0.00	0.10	0.00	0.00	0.00	0.00	0.06	0.24	0.00	0.00	0.52	0.00	0.00	0.02	0.00
0.00	0.00	0.00	0.00	0.00	0.00	0.00	0.00	0.00	0.00	0.00	0.00	0.00	0.00	0.00	0.00	0.00
0.01	0.00	0.00	0.00	0.00	0.00	0.00	0.00	0.21	0.00	0.00	0.00	0.04	0.00	0.14	0.00	0.00
0.00	0.00	0.00	0.01	0.00	0.00	0.00	0.00	0.00	0.02	0.00	0.00	0.10	0.00	0.00	0.00	0.00
0.00	0.00	0.00	0.00	0.00	0.00	0.00	0.00	0.00	0.00	0.00	0.00	0.00	0.00	0.00	0.00	0.00
0.00	0.00	0.00	0.00	0.01	0.00	0.00	0.00	0.07	0.01	0.00	0.00	0.04	0.00	0.03	0.01	0.00
0.00	0.00	0.00	0.00	0.00	0.00	0.00	0.00	0.00	0.01	0.00	0.00	0.00	0.00	0.00	0.00	0.00
0.00	0.00	0.00	0.00	0.00	0.00	0.00	0.00	0.00	0.00	0.00	0.00	0.00	0.00	0.00	0.00	0.00

0.48	0.14	0.21	1.32	0.60	0.01	0.00	0.00	1.39	1.06	0.00	0.00	3.00	0.00	0.45	0.15	0.00
0.15	0.04	0.06	0.40	0.18	0.00	0.00	0.00	0.42	0.32	0.00	0.00	0.91	0.00	0.14	0.05	0.00

For Peer Review

K24	K25	K26
0.00	0.00	0.00
0.00	0.00	0.00
0.00	0.00	0.00
0.01	0.00	0.00
0.00	0.00	0.00
0.00	0.00	0.00
0.00	0.01	0.00
0.00	0.00	0.00
0.02	0.02	0.00
0.00	0.00	0.00
0.00	0.00	0.00
0.00	0.00	0.00
0.00	0.00	0.00
0.00	0.00	0.00
0.00	0.00	0.00
0.00	0.00	0.00
0.00	0.00	0.00
0.00	0.00	0.00
0.00	0.00	0.00
0.00	0.00	0.00
0.01	0.00	0.00
0.00	0.00	0.00
0.00	0.00	0.00
0.00	0.00	0.00
0.00	0.00	0.00
0.00	0.00	0.00

0.05	0.03	0.00
0.02	0.01	0.00

Outdegree	
0.00	0.00
0.08	0.02
0.43	0.10
4.21	1.00
2.40	0.57
0.42	0.10
0.51	0.12
0.21	0.05
0.74	0.18
1.06	0.25
0.89	0.21
0.00	0.00
0.00	0.00
0.00	0.00
0.33	0.08
0.17	0.04
0.03	0.01
0.00	0.00
1.54	0.37
0.00	0.00
0.64	0.15
0.20	0.05
0.00	0.00
0.32	0.08
0.03	0.01
0.00	0.00

For Peer Review

Category	Code		K01	K02	K03	K04	K05	K06
BAD EXPERIENCE OF COVID-19 PANDEMIC	K01		0.000	0.00	0.00	0.00	0.00	0.00
INCREASE IN SOCIAL VICES	K02		0.154	0.23	0.00	0.00	0.00	0.00
LACK OF ACCESS TO MONEY	K03		0.077	0.00	0.00	0.00	0.00	0.00
LOCKDOWN	K04	3	0.308	0.23	0.12	0.29	0.31	0.19
REDUCED BUSINESSES/JOBS	K05		0.212	0.00	0.04	0.00	0.25	0.00
REDUCED SOCIAL CONTACT/ACTIVITIES	K06		0.115	0.00	0.00	0.00	0.00	0.04
REDUCED HEALTH SERVICES	K07		0.231	0.00	0.00	0.00	0.00	0.00
PROBLEMS WITH PPE	K08		0.058	0.00	0.00	0.00	0.00	0.00
FEAR AND MISCONCEPTION OF COVID-19	K09	5	0.288	0.00	0.00	0.00	0.00	0.23
INCREASE IN HOUSEHOLD CONFLICTS	K10	2	0.385	0.23	0.00	0.00	0.00	0.00
REDUCED EDUCATION	K11	3	0.308	0.23	0.00	0.00	0.00	0.00
PENALTIES/PUNISHMENT	K12		0.096	0.00	0.04	0.06	0.00	0.00
CONFLICT BETWEEN GOVERNMENT AND CITIZ	K13		0.000	0.00	0.00	0.00	0.00	0.00
ANIMAL/HERDERS PROBLEMS INCREASED	K14		0.000	0.00	0.00	0.00	0.00	0.00
STRESS AND MENTAL HEALTH PROBLEMS	K15		0.231	0.00	0.00	0.00	0.00	0.00
INCREASED ILLNESS/DEATH	K16		0.231	0.00	0.00	0.00	0.00	0.00
REDUCED GOVERNMENT SUPPORT	K17		0.000	0.00	0.00	0.00	0.00	0.00
INCREASE IN EARLY MARRIAGE	K18		0.000	0.00	0.00	0.00	0.00	0.00
HUNGER AND LACK OF FOOD	K19	1	0.538	0.00	0.00	0.00	0.00	0.00
LACK OF COVID-19 COMMODITIES	K20		0.000	0.00	0.00	0.00	0.00	0.00
REDUCED RELIGIOUS ACTIVITIES	K21	5	0.288	0.00	0.04	0.00	0.00	0.00
PRICE HIKE	K22		0.000	0.00	0.00	0.00	0.00	0.00
REDUCED PHYSICAL FITNESS	K23		0.000	0.00	0.00	0.00	0.00	0.00
ENFORCED SOCIAL DISTANCE	K24		0.000	0.00	0.00	0.00	0.00	0.00
FAULTY CLINICAL ISOLATION POLICIES	K25		0.000	0.00	0.00	0.00	0.00	0.00
	K26		0.000	0.00	0.00	0.00	0.00	0.00
Indegree			3.52	0.92	0.23	0.35	0.56	0.46
Relative			1.00	0.26	0.07	0.10	0.16	0.13
Rank								

For Peer Review

K07	K08	K09	K10	K11	K12	K13	K14	K15	K16	K17	K18	K19	K20	K21	K22	K23
0.00	0.00	0.00	0.00	0.00	0.00	0.00	0.00	0.00	0.00	0.00	0.00	0.00	0.00	0.00	0.00	0.00
0.00	0.00	0.00	0.00	0.00	0.12	0.00	0.00	0.08	0.08	0.00	0.00	0.00	0.00	0.00	0.00	0.00
0.00	0.00	0.00	0.08	0.00	0.00	0.00	0.00	0.04	0.00	0.00	0.00	0.15	0.00	0.00	0.00	0.00
0.00	0.00	0.00	0.54	0.38	0.19	0.00	0.00	0.46	0.13	0.00	0.00	0.83	0.00	0.48	0.00	0.00
0.00	0.00	0.00	0.69	0.00	0.00	0.00	0.00	0.04	0.19	0.00	0.00	1.00	0.00	0.00	0.00	0.00
0.00	0.00	0.00	0.00	0.00	0.00	0.00	0.00	0.19	0.00	0.00	0.00	0.00	0.00	0.00	0.00	0.00
0.12	0.00	0.00	0.00	0.00	0.00	0.00	0.00	0.00	0.23	0.00	0.00	0.00	0.00	0.00	0.00	0.00
0.00	0.02	0.00	0.00	0.00	0.00	0.00	0.00	0.00	0.00	0.00	0.00	0.00	0.00	0.00	0.00	0.00
0.50	0.00	0.40	0.00	0.00	0.00	0.00	0.00	0.00	0.23	0.00	0.00	0.00	0.00	0.00	0.00	0.00
0.00	0.00	0.00	0.42	0.13	0.04	0.00	0.00	0.08	0.12	0.00	0.00	0.37	0.00	0.00	0.00	0.00
0.00	0.00	0.00	0.08	0.25	0.04	0.00	0.00	0.15	0.04	0.00	0.00	0.00	0.00	0.00	0.00	0.00
0.00	0.00	0.00	0.08	0.00	0.00	0.00	0.00	0.06	0.00	0.00	0.00	0.15	0.00	0.06	0.00	0.00
0.00	0.00	0.00	0.00	0.00	0.00	0.00	0.00	0.00	0.00	0.00	0.00	0.00	0.00	0.00	0.00	0.00
0.00	0.00	0.00	0.00	0.00	0.00	0.00	0.00	0.00	0.00	0.00	0.00	0.00	0.00	0.00	0.00	0.00
0.00	0.00	0.00	0.00	0.00	0.00	0.00	0.00	0.06	0.00	0.00	0.00	0.00	0.00	0.00	0.00	0.00
0.00	0.00	0.00	0.00	0.00	0.00	0.00	0.00	0.04	0.00	0.00	0.00	0.00	0.00	0.00	0.00	0.00
0.00	0.00	0.00	0.00	0.00	0.00	0.00	0.00	0.00	0.00	0.00	0.00	0.00	0.00	0.00	0.00	0.00
0.00	0.00	0.00	0.00	0.00	0.00	0.00	0.00	0.00	0.00	0.00	0.00	0.00	0.00	0.00	0.00	0.00
0.00	0.00	0.00	0.40	0.00	0.00	0.00	0.00	0.00	0.23	0.00	0.00	0.81	0.00	0.00	0.00	0.00
0.00	0.00	0.00	0.00	0.00	0.00	0.00	0.00	0.00	0.00	0.00	0.00	0.00	0.00	0.00	0.00	0.00
0.00	0.00	0.00	0.08	0.00	0.00	0.00	0.00	0.25	0.00	0.00	0.00	0.15	0.00	0.13	0.00	0.00
0.00	0.00	0.00	0.00	0.00	0.00	0.00	0.00	0.00	0.00	0.00	0.00	0.00	0.00	0.00	0.00	0.00
0.00	0.00	0.00	0.00	0.00	0.00	0.00	0.00	0.00	0.00	0.00	0.00	0.00	0.00	0.00	0.00	0.00
0.00	0.00	0.00	0.00	0.00	0.00	0.00	0.00	0.00	0.00	0.00	0.00	0.00	0.00	0.00	0.00	0.00
0.00	0.00	0.00	0.00	0.00	0.00	0.00	0.00	0.00	0.00	0.00	0.00	0.00	0.00	0.00	0.00	0.00
0.00	0.00	0.00	0.00	0.00	0.00	0.00	0.00	0.00	0.00	0.00	0.00	0.00	0.00	0.00	0.00	0.00

0.62	0.02	0.40	2.37	0.77	0.38	0.00	0.00	1.44	1.25	0.00	0.00	3.46	0.00	0.67	0.00	0.00
0.17	0.01	0.11	0.67	0.22	0.11	0.00	0.00	0.41	0.36	0.00	0.00	0.98	0.00	0.19	0.00	0.00

K24	K25	K26
0.00	0.00	0.00
0.00	0.00	0.00
0.00	0.00	0.00
0.00	0.00	0.00
0.00	0.00	0.00
0.00	0.00	0.00
0.00	0.00	0.00
0.00	0.00	0.00
0.00	0.00	0.00
0.00	0.00	0.00
0.00	0.00	0.00
0.00	0.00	0.00
0.00	0.00	0.00
0.00	0.00	0.00
0.00	0.00	0.00
0.00	0.00	0.00
0.00	0.00	0.00
0.00	0.00	0.00
0.00	0.00	0.00
0.00	0.00	0.00
0.00	0.00	0.00
0.00	0.00	0.00
0.00	0.00	0.00
0.00	0.00	0.00
0.00	0.00	0.00
0.00	0.00	0.00
0.00	0.00	0.00
0.00	0.00	0.00

Outdegree	
0.00	0.00
0.65	0.15
0.35	0.08
4.46	1.00
2.42	0.54
0.35	0.08
0.58	0.13
0.08	0.02
1.65	0.37
1.77	0.40
1.10	0.25
0.54	0.12
0.00	0.00
0.00	0.00
0.29	0.06
0.27	0.06
0.00	0.00
0.00	0.00
1.98	0.44
0.00	0.00
0.94	0.21
0.00	0.00
0.00	0.00
0.00	0.00
0.00	0.00
0.00	0.00

For Peer Review

Category	Code		K01	K02	K03	K04	K05	K06
BAD EXPERIENCE OF COVID-19 PANDEMIC	K01		0.000	0.00	0.00	0.00	0.00	0.00
INCREASE IN SOCIAL VICES	K02		0.124	0.04	0.00	0.00	0.00	0.00
LACK OF ACCESS TO MONEY	K03		0.151	0.01	0.00	0.00	0.00	0.01
LOCKDOWN	K04	3	0.362	0.14	0.19	0.29	0.60	0.11
REDUCED BUSINESSES/JOBS	K05	2	0.426	0.04	0.19	0.00	0.28	0.02
REDUCED SOCIAL CONTACT/ACTIVITIES	K06		0.151	0.01	0.00	0.00	0.00	0.03
REDUCED HEALTH SERVICES	K07		0.130	0.00	0.00	0.00	0.00	0.00
PROBLEMS WITH PPE	K08		0.143	0.00	0.00	0.00	0.00	0.00
FEAR AND MISCONCEPTION OF COVID-19	K09		0.218	0.01	0.01	0.00	0.01	0.11
INCREASE IN HOUSEHOLD CONFLICTS	K10	5	0.328	0.08	0.01	0.00	0.01	0.00
REDUCED EDUCATION	K11		0.326	0.13	0.01	0.00	0.01	0.01
PENALTIES/PUNISHMENT	K12		0.039	0.00	0.00	0.00	0.00	0.00
CONFLICT BETWEEN GOVERNMENT AND CITIZ	K13		0.004	0.00	0.00	0.00	0.00	0.00
ANIMAL/HERDERS PROBLEMS INCREASED	K14		0.032	0.00	0.02	0.00	0.00	0.00
STRESS AND MENTAL HEALTH PROBLEMS	K15		0.321	0.00	0.00	0.00	0.00	0.00
INCREASED ILLNESS/DEATH	K16		0.200	0.00	0.00	0.00	0.00	0.00
REDUCED GOVERNMENT SUPPORT	K17		0.049	0.01	0.00	0.00	0.01	0.01
INCREASE IN EARLY MARRIAGE	K18		0.004	0.00	0.00	0.00	0.00	0.00
HUNGER AND LACK OF FOOD	K19	1	0.715	0.02	0.00	0.00	0.01	0.01
LACK OF COVID-19 COMMODITIES	K20		0.004	0.00	0.00	0.00	0.00	0.00
REDUCED RELIGIOUS ACTIVITIES	K21	4	0.335	0.00	0.02	0.00	0.00	0.03
PRICE HIKE	K22		0.079	0.00	0.00	0.00	0.00	0.00
REDUCED PHYSICAL FITNESS	K23		0.003	0.00	0.00	0.00	0.00	0.00
ENFORCED SOCIAL DISTANCE	K24		0.106	0.00	0.00	0.00	0.01	0.07
FAULTY CLINICAL ISOLATION POLICIES	K25		0.009	0.00	0.00	0.00	0.00	0.00
	K26		0.000	0.00	0.00	0.00	0.00	0.00
Indegree			4.26	0.49	0.45	0.30	0.94	0.39
Relative			1.00	0.12	0.11	0.07	0.22	0.09
Rank								

For Peer Review

K07	K08	K09	K10	K11	K12	K13	K14	K15	K16	K17	K18	K19	K20	K21	K22	K23
0.00	0.00	0.00	0.00	0.00	0.00	0.00	0.00	0.00	0.00	0.00	0.00	0.00	0.00	0.00	0.00	0.00
0.00	0.00	0.00	0.01	0.00	0.04	0.00	0.00	0.05	0.02	0.00	0.00	0.00	0.00	0.01	0.00	0.00
0.00	0.01	0.00	0.03	0.00	0.01	0.00	0.00	0.06	0.01	0.00	0.00	0.20	0.00	0.00	0.00	0.00
0.10	0.01	0.03	0.46	0.35	0.06	0.00	0.05	0.28	0.20	0.01	0.00	0.98	0.00	0.40	0.11	0.01
0.00	0.00	0.00	0.36	0.01	0.01	0.00	0.00	0.08	0.05	0.01	0.00	1.00	0.00	0.01	0.06	0.00
0.00	0.02	0.00	0.00	0.00	0.00	0.00	0.00	0.15	0.02	0.00	0.00	0.01	0.00	0.03	0.00	0.00
0.05	0.00	0.00	0.00	0.00	0.00	0.00	0.00	0.00	0.13	0.00	0.00	0.00	0.00	0.00	0.00	0.00
0.00	0.07	0.00	0.00	0.00	0.00	0.00	0.00	0.01	0.03	0.00	0.00	0.00	0.00	0.00	0.00	0.00
0.07	0.17	0.16	0.01	0.01	0.01	0.01	0.00	0.12	0.07	0.00	0.00	0.01	0.00	0.01	0.00	0.00
0.00	0.00	0.00	0.17	0.07	0.03	0.00	0.00	0.07	0.03	0.00	0.00	0.09	0.00	0.00	0.00	0.00
0.00	0.00	0.00	0.11	0.27	0.01	0.00	0.00	0.13	0.03	0.00	0.01	0.02	0.00	0.01	0.00	0.00
0.00	0.00	0.00	0.00	0.00	0.00	0.00	0.00	0.01	0.00	0.00	0.00	0.01	0.00	0.00	0.00	0.00
0.00	0.00	0.00	0.00	0.00	0.00	0.00	0.00	0.00	0.00	0.00	0.00	0.00	0.00	0.00	0.00	0.00
0.00	0.00	0.00	0.02	0.00	0.01	0.00	0.01	0.01	0.00	0.00	0.00	0.06	0.00	0.00	0.00	0.00
0.00	0.00	0.00	0.00	0.00	0.00	0.00	0.00	0.09	0.02	0.00	0.00	0.00	0.00	0.00	0.00	0.00
0.00	0.00	0.00	0.01	0.00	0.00	0.00	0.00	0.02	0.01	0.00	0.00	0.00	0.00	0.00	0.00	0.00
0.00	0.00	0.00	0.01	0.01	0.00	0.00	0.00	0.01	0.01	0.00	0.00	0.04	0.00	0.00	0.00	0.00
0.00	0.00	0.00	0.00	0.00	0.00	0.00	0.00	0.00	0.00	0.00	0.00	0.00	0.00	0.00	0.00	0.00
0.00	0.00	0.00	0.31	0.00	0.01	0.00	0.00	0.04	0.15	0.00	0.00	0.56	0.00	0.00	0.07	0.00
0.00	0.00	0.00	0.00	0.00	0.00	0.00	0.00	0.00	0.00	0.00	0.00	0.00	0.00	0.00	0.00	0.00
0.00	0.00	0.00	0.00	0.01	0.00	0.00	0.00	0.37	0.02	0.00	0.00	0.01	0.00	0.16	0.00	0.00
0.00	0.00	0.00	0.04	0.00	0.00	0.00	0.00	0.00	0.03	0.00	0.00	0.17	0.00	0.00	0.00	0.00
0.00	0.00	0.00	0.00	0.00	0.00	0.00	0.00	0.00	0.00	0.00	0.00	0.00	0.00	0.00	0.00	0.00
0.00	0.01	0.00	0.00	0.00	0.00	0.00	0.00	0.15	0.01	0.00	0.00	0.02	0.00	0.12	0.00	0.00
0.00	0.00	0.00	0.00	0.00	0.00	0.00	0.00	0.00	0.01	0.00	0.00	0.00	0.00	0.00	0.00	0.00
0.00	0.00	0.00	0.00	0.00	0.00	0.00	0.00	0.00	0.00	0.00	0.00	0.00	0.00	0.00	0.00	0.00

0.22	0.28	0.20	1.56	0.73	0.18	0.01	0.06	1.68	0.88	0.03	0.02	3.20	0.00	0.76	0.25	0.01
0.05	0.07	0.05	0.37	0.17	0.04	0.00	0.02	0.40	0.21	0.01	0.00	0.75	0.00	0.18	0.06	0.00

For peer review

K24	K25	K26
0.00	0.00	0.00
0.00	0.00	0.00
0.00	0.00	0.00
0.09	0.00	0.00
0.00	0.00	0.00
0.00	0.00	0.00
0.00	0.00	0.00
0.00	0.00	0.00
0.02	0.01	0.00
0.00	0.00	0.00
0.00	0.00	0.00
0.00	0.00	0.00
0.00	0.00	0.00
0.00	0.00	0.00
0.00	0.00	0.00
0.00	0.00	0.00
0.00	0.00	0.00
0.00	0.00	0.00
0.00	0.00	0.00
0.00	0.00	0.00
0.03	0.00	0.00
0.00	0.00	0.00
0.00	0.00	0.00
0.00	0.00	0.00
0.00	0.00	0.00
0.00	0.00	0.00

0.15	0.02	0.00
0.03	0.00	0.00

Outdegree	
0.00	0.00
0.31	0.06
0.49	0.10
4.84	1.00
2.55	0.53
0.41	0.09
0.32	0.07
0.25	0.05
1.05	0.22
0.88	0.18
1.08	0.22
0.08	0.02
0.02	0.00
0.17	0.03
0.44	0.09
0.24	0.05
0.17	0.03
0.00	0.00
1.89	0.39
0.01	0.00
1.00	0.21
0.34	0.07
0.01	0.00
0.52	0.11
0.02	0.00
0.00	0.00

For Peer Review

Category	Code		K01	K02	K03	K04	K05	K06
BAD EXPERIENCE OF COVID-19 PANDEMIC	K01		0.000	0.00	0.00	0.00	0.00	0.00
INCREASE IN SOCIAL VICES	K02		0.206	0.17	0.00	0.00	0.00	0.00
LACK OF ACCESS TO MONEY	K03		0.138	0.00	0.01	0.00	0.00	0.02
LOCKDOWN	K04	5	0.291	0.28	0.18	0.31	0.44	0.06
REDUCED BUSINESSES/JOBS	K05	4	0.316	0.06	0.14	0.00	0.18	0.04
REDUCED SOCIAL CONTACT/ACTIVITIES	K06		0.153	0.01	0.00	0.00	0.00	0.04
REDUCED HEALTH SERVICES	K07		0.116	0.02	0.00	0.00	0.01	0.01
PROBLEMS WITH PPE	K08		0.109	0.00	0.00	0.00	0.00	0.00
FEAR AND MISCONCEPTION OF COVID-19	K09		0.278	0.01	0.01	0.00	0.01	0.03
INCREASE IN HOUSEHOLD CONFLICTS	K10	2	0.388	0.30	0.00	0.00	0.00	0.00
REDUCED EDUCATION	K11		0.250	0.17	0.01	0.01	0.00	0.00
PENALTIES/PUNISHMENT	K12		0.003	0.01	0.00	0.00	0.00	0.00
CONFLICT BETWEEN GOVERNMENT AND CITIZ	K13		0.006	0.00	0.00	0.00	0.00	0.00
ANIMAL/HERDERS PROBLEMS INCREASED	K14		0.000	0.00	0.00	0.00	0.00	0.00
STRESS AND MENTAL HEALTH PROBLEMS	K15	3	0.334	0.02	0.00	0.00	0.00	0.01
INCREASED ILLNESS/DEATH	K16		0.172	0.01	0.00	0.00	0.00	0.00
REDUCED GOVERNMENT SUPPORT	K17		0.028	0.00	0.00	0.00	0.00	0.00
INCREASE IN EARLY MARRIAGE	K18		0.000	0.00	0.00	0.00	0.00	0.00
HUNGER AND LACK OF FOOD	K19	1	0.575	0.04	0.00	0.00	0.00	0.02
LACK OF COVID-19 COMMODITIES	K20		0.000	0.00	0.00	0.00	0.00	0.00
REDUCED RELIGIOUS ACTIVITIES	K21		0.256	0.09	0.04	0.00	0.00	0.00
PRICE HIKE	K22		0.091	0.02	0.00	0.00	0.00	0.00
REDUCED PHYSICAL FITNESS	K23		0.013	0.00	0.00	0.00	0.00	0.00
ENFORCED SOCIAL DISTANCE	K24		0.072	0.04	0.02	0.00	0.00	0.11
FAULTY CLINICAL ISOLATION POLICIES	K25		0.053	0.00	0.00	0.00	0.00	0.00
	K26		0.000	0.00	0.00	0.00	0.00	0.00
Indegree			3.85	1.25	0.41	0.31	0.63	0.33
Relative			1.00	0.32	0.11	0.08	0.16	0.09
Rank								

For Peer Review

K07	K08	K09	K10	K11	K12	K13	K14	K15	K16	K17	K18	K19	K20	K21	K22	K23
0.00	0.00	0.00	0.00	0.00	0.00	0.00	0.00	0.00	0.00	0.00	0.00	0.00	0.00	0.00	0.00	0.00
0.00	0.00	0.01	0.03	0.00	0.00	0.00	0.00	0.06	0.05	0.00	0.00	0.02	0.00	0.02	0.00	0.00
0.00	0.00	0.00	0.01	0.00	0.00	0.00	0.00	0.08	0.01	0.01	0.00	0.14	0.00	0.01	0.00	0.00
0.13	0.00	0.02	0.69	0.30	0.03	0.00	0.00	0.27	0.20	0.01	0.00	1.00	0.00	0.43	0.20	0.00
0.00	0.00	0.00	0.46	0.00	0.00	0.00	0.00	0.12	0.06	0.03	0.00	0.73	0.00	0.03	0.08	0.00
0.00	0.00	0.00	0.01	0.00	0.00	0.00	0.00	0.21	0.02	0.00	0.00	0.00	0.00	0.05	0.00	0.00
0.06	0.00	0.02	0.00	0.00	0.00	0.00	0.00	0.01	0.12	0.00	0.00	0.01	0.00	0.00	0.00	0.00
0.00	0.11	0.00	0.00	0.00	0.00	0.00	0.00	0.00	0.03	0.00	0.00	0.00	0.00	0.00	0.00	0.00
0.09	0.15	0.28	0.00	0.00	0.00	0.03	0.00	0.13	0.08	0.00	0.00	0.01	0.00	0.00	0.00	0.00
0.00	0.00	0.00	0.34	0.13	0.00	0.00	0.00	0.11	0.09	0.00	0.00	0.11	0.00	0.01	0.00	0.00
0.00	0.00	0.00	0.17	0.17	0.00	0.00	0.00	0.09	0.03	0.00	0.00	0.00	0.00	0.01	0.00	0.00
0.00	0.00	0.00	0.00	0.00	0.00	0.00	0.00	0.00	0.00	0.00	0.00	0.01	0.00	0.00	0.00	0.00
0.00	0.00	0.01	0.00	0.00	0.00	0.00	0.00	0.01	0.00	0.00	0.00	0.00	0.00	0.00	0.00	0.00
0.00	0.00	0.00	0.00	0.00	0.00	0.00	0.00	0.00	0.00	0.00	0.00	0.00	0.00	0.00	0.00	0.00
0.00	0.00	0.00	0.03	0.00	0.00	0.00	0.00	0.13	0.01	0.00	0.00	0.01	0.00	0.00	0.00	0.00
0.00	0.00	0.00	0.00	0.00	0.00	0.00	0.00	0.02	0.00	0.00	0.00	0.00	0.00	0.01	0.00	0.00
0.00	0.00	0.00	0.00	0.00	0.00	0.00	0.00	0.01	0.00	0.00	0.00	0.00	0.00	0.00	0.00	0.00
0.00	0.00	0.00	0.00	0.00	0.00	0.00	0.00	0.00	0.00	0.00	0.00	0.00	0.00	0.00	0.00	0.00
0.00	0.00	0.00	0.33	0.00	0.00	0.00	0.00	0.08	0.11	0.01	0.00	0.47	0.00	0.01	0.08	0.00
0.00	0.00	0.00	0.00	0.00	0.00	0.00	0.00	0.00	0.00	0.00	0.00	0.00	0.00	0.00	0.00	0.00
0.00	0.00	0.00	0.02	0.00	0.00	0.00	0.00	0.27	0.05	0.00	0.00	0.00	0.00	0.17	0.00	0.00
0.00	0.00	0.00	0.06	0.00	0.00	0.00	0.00	0.00	0.02	0.00	0.00	0.23	0.00	0.00	0.00	0.00
0.00	0.00	0.00	0.00	0.00	0.00	0.00	0.00	0.00	0.01	0.00	0.00	0.00	0.00	0.00	0.00	0.01
0.00	0.00	0.00	0.01	0.00	0.00	0.00	0.00	0.17	0.02	0.00	0.00	0.01	0.00	0.08	0.00	0.00
0.07	0.00	0.01	0.00	0.00	0.00	0.00	0.00	0.01	0.04	0.00	0.00	0.00	0.00	0.00	0.00	0.00
0.00	0.00	0.00	0.00	0.00	0.00	0.00	0.00	0.00	0.00	0.00	0.00	0.00	0.00	0.00	0.00	0.00
0.35	0.26	0.34	2.17	0.60	0.03	0.03	0.00	1.78	0.95	0.06	0.00	2.74	0.00	0.81	0.37	0.01
0.09	0.07	0.09	0.56	0.16	0.01	0.01	0.00	0.46	0.25	0.02	0.00	0.71	0.00	0.21	0.10	0.00

K24	K25	K26
0.00	0.00	0.00
0.00	0.00	0.00
0.00	0.00	0.00
0.00	0.00	0.00
0.00	0.00	0.00
0.00	0.00	0.00
0.00	0.01	0.00
0.00	0.00	0.00
0.04	0.03	0.00
0.00	0.00	0.00
0.00	0.00	0.00
0.00	0.00	0.00
0.00	0.00	0.00
0.00	0.00	0.00
0.00	0.00	0.00
0.00	0.00	0.00
0.00	0.00	0.00
0.00	0.00	0.00
0.00	0.00	0.00
0.00	0.00	0.00
0.00	0.00	0.00
0.00	0.00	0.00
0.00	0.00	0.00
0.00	0.00	0.00
0.00	0.02	0.00
0.00	0.00	0.00

0.04	0.06	0.00
0.01	0.01	0.00

Outdegree	
0.00	0.00
0.55	0.11
0.43	0.09
4.84	1.00
2.24	0.46
0.50	0.10
0.36	0.07
0.24	0.05
1.17	0.24
1.47	0.30
0.92	0.19
0.02	0.00
0.02	0.00
0.00	0.00
0.55	0.11
0.21	0.04
0.03	0.01
0.00	0.00
1.73	0.36
0.00	0.00
0.90	0.19
0.41	0.09
0.03	0.01
0.53	0.11
0.20	0.04
0.00	0.00

For Peer Review

Category	Code		K01	K02	K03	K04	K05	K06
BAD EXPERIENCE OF COVID-19 PANDEMIC	K01		0.000	0.00	0.00	0.00	0.00	0.00
INCREASE IN SOCIAL VICES	K02		0.059	0.03	0.00	0.00	0.00	0.00
LACK OF ACCESS TO MONEY	K03		0.156	0.01	0.00	0.00	0.00	0.00
LOCKDOWN	K04	4	0.358	0.07	0.17	0.28	0.51	0.13
REDUCED BUSINESSES/JOBS	K05	2	0.449	0.02	0.21	0.00	0.29	0.01
REDUCED SOCIAL CONTACT/ACTIVITIES	K06		0.170	0.00	0.00	0.00	0.01	0.03
REDUCED HEALTH SERVICES	K07		0.151	0.00	0.00	0.00	0.00	0.00
PROBLEMS WITH PPE	K08		0.146	0.00	0.00	0.00	0.00	0.00
FEAR AND MISCONCEPTION OF COVID-19	K09		0.217	0.00	0.00	0.00	0.01	0.11
INCREASE IN HOUSEHOLD CONFLICTS	K10		0.314	0.07	0.03	0.00	0.03	0.00
REDUCED EDUCATION	K11	5	0.327	0.09	0.01	0.00	0.01	0.02
PENALTIES/PUNISHMENT	K12		0.022	0.00	0.00	0.00	0.00	0.00
CONFLICT BETWEEN GOVERNMENT AND CITIZ	K13		0.000	0.00	0.00	0.00	0.00	0.00
ANIMAL/HERDERS PROBLEMS INCREASED	K14		0.037	0.00	0.02	0.00	0.00	0.00
STRESS AND MENTAL HEALTH PROBLEMS	K15	3	0.363	0.00	0.00	0.00	0.00	0.00
INCREASED ILLNESS/DEATH	K16		0.178	0.00	0.00	0.00	0.00	0.00
REDUCED GOVERNMENT SUPPORT	K17		0.030	0.00	0.00	0.00	0.02	0.00
INCREASE IN EARLY MARRIAGE	K18		0.000	0.00	0.00	0.00	0.00	0.00
HUNGER AND LACK OF FOOD	K19	1	0.681	0.01	0.00	0.00	0.03	0.00
LACK OF COVID-19 COMMODITIES	K20		0.010	0.00	0.00	0.00	0.00	0.00
REDUCED RELIGIOUS ACTIVITIES	K21		0.244	0.00	0.01	0.00	0.00	0.03
PRICE HIKE	K22		0.084	0.00	0.00	0.00	0.03	0.00
REDUCED PHYSICAL FITNESS	K23		0.000	0.00	0.00	0.00	0.00	0.00
ENFORCED SOCIAL DISTANCE	K24		0.096	0.00	0.00	0.00	0.01	0.09
FAULTY CLINICAL ISOLATION POLICIES	K25		0.002	0.00	0.00	0.00	0.00	0.00
	K26		0.000	0.00	0.00	0.00	0.00	0.00

	K01	K02	K03	K04	K05	K06
Indegree	4.10	0.29	0.46	0.28	0.96	0.42
Relative	1.00	0.07	0.11	0.07	0.23	0.10
Rank						

For Peer Review

K07	K08	K09	K10	K11	K12	K13	K14	K15	K16	K17	K18	K19	K20	K21	K22	K23
0.00	0.00	0.00	0.00	0.00	0.00	0.00	0.00	0.00	0.00	0.00	0.00	0.00	0.00	0.00	0.00	0.00
0.00	0.00	0.00	0.00	0.00	0.01	0.00	0.00	0.01	0.01	0.00	0.00	0.00	0.00	0.00	0.00	0.00
0.00	0.00	0.00	0.00	0.00	0.00	0.00	0.00	0.02	0.03	0.00	0.00	0.21	0.00	0.00	0.00	0.00
0.14	0.00	0.01	0.48	0.47	0.03	0.00	0.08	0.32	0.18	0.00	0.00	0.88	0.00	0.28	0.12	0.00
0.00	0.00	0.00	0.24	0.02	0.01	0.00	0.00	0.08	0.07	0.00	0.00	1.00	0.00	0.00	0.08	0.00
0.00	0.02	0.00	0.01	0.00	0.00	0.00	0.00	0.16	0.03	0.01	0.00	0.01	0.00	0.02	0.00	0.00
0.05	0.00	0.00	0.00	0.00	0.00	0.00	0.00	0.02	0.15	0.00	0.00	0.00	0.00	0.00	0.00	0.00
0.00	0.10	0.00	0.00	0.00	0.00	0.00	0.00	0.01	0.02	0.00	0.00	0.00	0.00	0.00	0.00	0.00
0.11	0.14	0.15	0.02	0.01	0.00	0.00	0.00	0.12	0.08	0.01	0.00	0.01	0.00	0.03	0.00	0.00
0.00	0.00	0.00	0.18	0.04	0.02	0.00	0.00	0.14	0.02	0.00	0.00	0.11	0.00	0.00	0.00	0.00
0.01	0.00	0.01	0.29	0.28	0.01	0.00	0.00	0.20	0.03	0.00	0.00	0.03	0.00	0.01	0.00	0.00
0.00	0.00	0.00	0.00	0.00	0.00	0.00	0.00	0.00	0.00	0.00	0.00	0.00	0.00	0.00	0.00	0.00
0.00	0.00	0.00	0.00	0.00	0.00	0.00	0.00	0.00	0.00	0.00	0.00	0.00	0.00	0.00	0.00	0.00
0.00	0.00	0.00	0.00	0.00	0.02	0.00	0.01	0.00	0.00	0.00	0.00	0.02	0.00	0.00	0.00	0.00
0.00	0.00	0.00	0.00	0.00	0.00	0.00	0.00	0.08	0.01	0.00	0.00	0.00	0.00	0.00	0.00	0.00
0.00	0.00	0.00	0.00	0.00	0.00	0.00	0.00	0.01	0.00	0.00	0.00	0.00	0.00	0.00	0.00	0.00
0.00	0.00	0.00	0.00	0.00	0.00	0.00	0.00	0.01	0.01	0.00	0.00	0.02	0.00	0.00	0.00	0.00
0.00	0.00	0.00	0.00	0.00	0.00	0.00	0.00	0.00	0.00	0.00	0.00	0.00	0.00	0.00	0.00	0.00
0.00	0.00	0.00	0.17	0.00	0.00	0.00	0.00	0.04	0.16	0.00	0.00	0.55	0.00	0.00	0.07	0.00
0.00	0.00	0.00	0.00	0.00	0.00	0.00	0.00	0.01	0.00	0.00	0.00	0.00	0.00	0.00	0.00	0.00
0.00	0.00	0.00	0.00	0.00	0.00	0.00	0.00	0.23	0.01	0.00	0.00	0.00	0.00	0.14	0.00	0.00
0.00	0.00	0.00	0.03	0.00	0.00	0.00	0.00	0.02	0.03	0.00	0.00	0.17	0.00	0.00	0.00	0.00
0.00	0.00	0.00	0.00	0.00	0.00	0.00	0.00	0.00	0.00	0.00	0.00	0.00	0.00	0.00	0.00	0.00
0.00	0.00	0.00	0.01	0.00	0.00	0.00	0.00	0.14	0.00	0.00	0.00	0.04	0.00	0.06	0.01	0.00
0.00	0.00	0.00	0.00	0.00	0.00	0.00	0.00	0.00	0.00	0.00	0.00	0.00	0.00	0.00	0.00	0.00
0.00	0.00	0.00	0.00	0.00	0.00	0.00	0.00	0.00	0.00	0.00	0.00	0.00	0.00	0.00	0.00	0.00

0.30	0.26	0.18	1.44	0.82	0.12	0.00	0.09	1.61	0.84	0.01	0.00	3.04	0.00	0.55	0.29	0.00
0.07	0.06	0.04	0.35	0.20	0.03	0.00	0.02	0.39	0.20	0.00	0.00	0.74	0.00	0.14	0.07	0.00

For Peer Review

K24	K25	K26
0.00	0.00	0.00
0.00	0.00	0.00
0.00	0.00	0.00
0.06	0.00	0.00
0.01	0.00	0.00
0.00	0.00	0.00
0.00	0.00	0.00
0.00	0.00	0.00
0.03	0.00	0.00
0.00	0.00	0.00
0.01	0.00	0.00
0.00	0.00	0.00
0.00	0.00	0.00
0.00	0.00	0.00
0.00	0.00	0.00
0.00	0.00	0.00
0.00	0.01	0.00
0.00	0.00	0.00
0.00	0.00	0.00
0.00	0.00	0.00
0.02	0.00	0.00
0.00	0.00	0.00
0.00	0.00	0.00
0.00	0.00	0.00
0.00	0.00	0.00
0.00	0.00	0.00

0.13	0.01	0.00
0.03	0.00	0.00

Outdegree	
0.00	0.00
0.13	0.03
0.42	0.09
4.55	1.00
2.49	0.55
0.48	0.11
0.37	0.08
0.27	0.06
1.03	0.23
0.95	0.21
1.35	0.30
0.02	0.00
0.00	0.00
0.10	0.02
0.45	0.10
0.19	0.04
0.10	0.02
0.00	0.00
1.72	0.38
0.02	0.00
0.69	0.15
0.37	0.08
0.00	0.00
0.46	0.10
0.01	0.00
0.00	0.00

For Peer Review

Category	Code		K01	K02	K03	K04	K05	K06
BAD EXPERIENCE OF COVID-19 PANDEMIC	K01		0.000	0.00	0.00	0.00	0.00	0.00
INCREASE IN SOCIAL VICES	K02		0.120	0.05	0.00	0.00	0.00	0.00
LACK OF ACCESS TO MONEY	K03		0.125	0.01	0.01	0.00	0.00	0.01
LOCKDOWN	K04	4	0.317	0.11	0.16	0.26	0.56	0.14
REDUCED BUSINESSES/JOBS	K05	2	0.384	0.03	0.22	0.00	0.24	0.01
REDUCED SOCIAL CONTACT/ACTIVITIES	K06		0.144	0.00	0.00	0.00	0.00	0.02
REDUCED HEALTH SERVICES	K07		0.170	0.00	0.00	0.00	0.00	0.01
PROBLEMS WITH PPE	K08		0.120	0.00	0.00	0.00	0.00	0.01
FEAR AND MISCONCEPTION OF COVID-19	K09		0.197	0.00	0.00	0.00	0.00	0.06
INCREASE IN HOUSEHOLD CONFLICTS	K10	3	0.352	0.07	0.00	0.00	0.01	0.00
REDUCED EDUCATION	K11		0.247	0.11	0.00	0.00	0.00	0.00
PENALTIES/PUNISHMENT	K12		0.022	0.00	0.00	0.00	0.00	0.00
CONFLICT BETWEEN GOVERNMENT AND CITIZ	K13		0.000	0.00	0.00	0.00	0.00	0.00
ANIMAL/HERDERS PROBLEMS INCREASED	K14		0.009	0.00	0.01	0.00	0.00	0.00
STRESS AND MENTAL HEALTH PROBLEMS	K15		0.272	0.00	0.00	0.00	0.00	0.00
INCREASED ILLNESS/DEATH	K16		0.178	0.00	0.00	0.00	0.00	0.00
REDUCED GOVERNMENT SUPPORT	K17		0.012	0.01	0.00	0.00	0.00	0.00
INCREASE IN EARLY MARRIAGE	K18		0.000	0.00	0.00	0.00	0.00	0.00
HUNGER AND LACK OF FOOD	K19	1	0.640	0.02	0.01	0.00	0.00	0.01
LACK OF COVID-19 COMMODITIES	K20		0.003	0.00	0.00	0.00	0.00	0.00
REDUCED RELIGIOUS ACTIVITIES	K21	5	0.274	0.00	0.01	0.00	0.00	0.02
PRICE HIKE	K22		0.049	0.00	0.00	0.00	0.00	0.00
REDUCED PHYSICAL FITNESS	K23		0.000	0.00	0.00	0.00	0.00	0.00
ENFORCED SOCIAL DISTANCE	K24		0.100	0.00	0.01	0.01	0.02	0.08
FAULTY CLINICAL ISOLATION POLICIES	K25		0.009	0.00	0.00	0.00	0.00	0.00
	K26		0.000	0.00	0.00	0.00	0.00	0.00

Indegree	3.74	0.40	0.43	0.28	0.84	0.39
Relative	1.00	0.11	0.11	0.07	0.23	0.10
Rank						

For Peer Review

K07	K08	K09	K10	K11	K12	K13	K14	K15	K16	K17	K18	K19	K20	K21	K22	K23
0.00	0.00	0.00	0.00	0.00	0.00	0.00	0.00	0.00	0.00	0.00	0.00	0.00	0.00	0.00	0.00	0.00
0.01	0.00	0.00	0.01	0.00	0.02	0.00	0.00	0.04	0.03	0.00	0.00	0.00	0.00	0.00	0.00	0.00
0.01	0.01	0.00	0.04	0.00	0.00	0.00	0.00	0.03	0.02	0.00	0.00	0.25	0.00	0.00	0.00	0.00
0.15	0.01	0.01	0.48	0.31	0.02	0.00	0.01	0.27	0.20	0.00	0.00	0.89	0.01	0.31	0.06	0.00
0.05	0.00	0.00	0.36	0.00	0.00	0.00	0.00	0.09	0.11	0.00	0.00	1.00	0.00	0.00	0.03	0.00
0.01	0.01	0.00	0.00	0.00	0.00	0.00	0.00	0.12	0.02	0.00	0.00	0.01	0.00	0.00	0.00	0.00
0.07	0.00	0.00	0.00	0.00	0.00	0.00	0.00	0.02	0.17	0.00	0.00	0.00	0.00	0.00	0.00	0.00
0.01	0.07	0.00	0.00	0.00	0.00	0.00	0.00	0.00	0.02	0.00	0.00	0.01	0.00	0.00	0.00	0.00
0.06	0.12	0.18	0.00	0.00	0.00	0.00	0.00	0.04	0.04	0.00	0.00	0.01	0.00	0.00	0.00	0.00
0.00	0.00	0.00	0.22	0.05	0.01	0.00	0.00	0.11	0.03	0.00	0.00	0.08	0.00	0.00	0.00	0.00
0.00	0.00	0.00	0.14	0.22	0.01	0.00	0.00	0.13	0.01	0.00	0.00	0.01	0.00	0.00	0.00	0.00
0.00	0.00	0.00	0.00	0.00	0.00	0.00	0.00	0.01	0.00	0.00	0.00	0.00	0.00	0.00	0.00	0.00
0.00	0.00	0.00	0.00	0.00	0.00	0.00	0.00	0.00	0.00	0.00	0.00	0.00	0.00	0.00	0.00	0.00
0.00	0.00	0.00	0.02	0.00	0.00	0.00	0.00	0.01	0.00	0.00	0.00	0.04	0.00	0.00	0.00	0.00
0.00	0.00	0.00	0.01	0.00	0.00	0.00	0.00	0.04	0.00	0.00	0.00	0.00	0.00	0.00	0.00	0.00
0.00	0.00	0.00	0.00	0.00	0.00	0.00	0.00	0.01	0.01	0.00	0.00	0.00	0.00	0.00	0.00	0.00
0.00	0.00	0.00	0.01	0.01	0.00	0.00	0.00	0.01	0.00	0.00	0.00	0.02	0.00	0.00	0.00	0.00
0.00	0.00	0.00	0.00	0.00	0.00	0.00	0.00	0.00	0.00	0.00	0.00	0.00	0.00	0.00	0.00	0.00
0.02	0.00	0.00	0.23	0.00	0.00	0.00	0.00	0.06	0.19	0.00	0.00	0.56	0.00	0.00	0.03	0.00
0.01	0.00	0.00	0.00	0.00	0.00	0.00	0.00	0.00	0.00	0.00	0.00	0.00	0.00	0.00	0.00	0.00
0.00	0.00	0.00	0.00	0.00	0.00	0.00	0.00	0.30	0.00	0.00	0.00	0.00	0.00	0.16	0.00	0.00
0.00	0.00	0.00	0.03	0.00	0.00	0.00	0.00	0.00	0.03	0.00	0.00	0.11	0.00	0.00	0.00	0.00
0.00	0.00	0.00	0.00	0.00	0.00	0.00	0.00	0.00	0.00	0.00	0.00	0.00	0.00	0.00	0.00	0.00
0.00	0.01	0.00	0.01	0.01	0.00	0.00	0.00	0.11	0.01	0.00	0.00	0.06	0.00	0.08	0.01	0.00
0.00	0.00	0.00	0.00	0.00	0.00	0.00	0.00	0.00	0.00	0.00	0.00	0.00	0.00	0.00	0.00	0.00
0.00	0.00	0.00	0.00	0.00	0.00	0.00	0.00	0.00	0.00	0.00	0.00	0.00	0.00	0.00	0.00	0.00

0.40	0.23	0.19	1.57	0.61	0.07	0.00	0.02	1.41	0.89	0.00	0.00	3.06	0.01	0.56	0.13	0.00
0.11	0.06	0.05	0.42	0.16	0.02	0.00	0.00	0.38	0.24	0.00	0.00	0.82	0.00	0.15	0.03	0.00

For Peer Review

K24	K25	K26
0.00	0.00	0.00
0.00	0.00	0.00
0.00	0.00	0.00
0.07	0.00	0.00
0.00	0.00	0.00
0.00	0.00	0.00
0.00	0.01	0.00
0.00	0.00	0.00
0.01	0.01	0.00
0.00	0.00	0.00
0.00	0.00	0.00
0.00	0.00	0.00
0.00	0.00	0.00
0.00	0.00	0.00
0.00	0.00	0.00
0.00	0.00	0.00
0.00	0.00	0.00
0.00	0.00	0.00
0.00	0.00	0.00
0.00	0.00	0.00
0.02	0.00	0.00
0.00	0.00	0.00
0.00	0.00	0.00
0.00	0.00	0.00
0.00	0.00	0.00
0.00	0.00	0.00

0.10	0.02	0.00
0.03	0.00	0.00

Outdegree	
0.00	0.00
0.27	0.06
0.50	0.12
4.35	1.00
2.52	0.58
0.35	0.08
0.45	0.10
0.24	0.06
0.74	0.17
0.94	0.22
0.88	0.20
0.03	0.01
0.00	0.00
0.10	0.02
0.32	0.07
0.20	0.05
0.08	0.02
0.00	0.00
1.79	0.41
0.01	0.00
0.79	0.18
0.22	0.05
0.00	0.00
0.53	0.12
0.01	0.00
0.00	0.00

For Peer Review

Category	Code		K01	K02	K03	K04	K05	K06
BAD EXPERIENCE OF COVID-19 PANDEMIC	K01		0.000	0.00	0.00	0.00	0.00	0.00
INCREASE IN SOCIAL VICES	K02		0.083	0.06	0.00	0.00	0.00	0.00
LACK OF ACCESS TO MONEY	K03		0.133	0.00	0.00	0.00	0.00	0.01
LOCKDOWN	K04	3	0.319	0.11	0.16	0.28	0.56	0.16
REDUCED BUSINESSES/JOBS	K05	2	0.401	0.02	0.22	0.00	0.22	0.02
REDUCED SOCIAL CONTACT/ACTIVITIES	K06		0.124	0.00	0.01	0.00	0.01	0.04
REDUCED HEALTH SERVICES	K07		0.163	0.00	0.00	0.00	0.00	0.01
PROBLEMS WITH PPE	K08		0.113	0.00	0.00	0.00	0.00	0.00
FEAR AND MISCONCEPTION OF COVID-19	K09		0.205	0.02	0.01	0.00	0.01	0.04
INCREASE IN HOUSEHOLD CONFLICTS	K10	4	0.302	0.09	0.00	0.00	0.00	0.00
REDUCED EDUCATION	K11	5	0.285	0.10	0.00	0.00	0.01	0.00
PENALTIES/PUNISHMENT	K12		0.010	0.00	0.00	0.00	0.00	0.00
CONFLICT BETWEEN GOVERNMENT AND CITIZ	K13		0.003	0.00	0.00	0.00	0.00	0.00
ANIMAL/HERDERS PROBLEMS INCREASED	K14		0.010	0.00	0.01	0.00	0.00	0.00
STRESS AND MENTAL HEALTH PROBLEMS	K15		0.243	0.00	0.00	0.00	0.00	0.00
INCREASED ILLNESS/DEATH	K16		0.185	0.00	0.00	0.00	0.00	0.00
REDUCED GOVERNMENT SUPPORT	K17		0.021	0.00	0.00	0.00	0.00	0.00
INCREASE IN EARLY MARRIAGE	K18		0.006	0.00	0.00	0.00	0.00	0.00
HUNGER AND LACK OF FOOD	K19	1	0.544	0.01	0.00	0.00	0.00	0.01
LACK OF COVID-19 COMMODITIES	K20		0.000	0.00	0.00	0.00	0.00	0.00
REDUCED RELIGIOUS ACTIVITIES	K21		0.258	0.00	0.02	0.00	0.00	0.02
PRICE HIKE	K22		0.071	0.00	0.00	0.00	0.01	0.00
REDUCED PHYSICAL FITNESS	K23		0.004	0.00	0.00	0.00	0.00	0.00
ENFORCED SOCIAL DISTANCE	K24		0.063	0.00	0.00	0.00	0.00	0.05
FAULTY CLINICAL ISOLATION POLICIES	K25		0.024	0.00	0.00	0.00	0.00	0.00
	K26		0.000	0.00	0.00	0.00	0.00	0.00
Indegree			3.57	0.41	0.43	0.28	0.82	0.34
Relative			1.00	0.11	0.12	0.08	0.23	0.10
Rank								

For Peer Review

K07	K08	K09	K10	K11	K12	K13	K14	K15	K16	K17	K18	K19	K20	K21	K22	K23
0.00	0.00	0.00	0.00	0.00	0.00	0.00	0.00	0.00	0.00	0.00	0.00	0.00	0.00	0.00	0.00	0.00
0.00	0.00	0.00	0.00	0.00	0.03	0.00	0.00	0.01	0.02	0.00	0.00	0.00	0.00	0.00	0.00	0.00
0.00	0.00	0.00	0.00	0.00	0.00	0.00	0.00	0.06	0.02	0.00	0.00	0.20	0.00	0.00	0.00	0.00
0.16	0.00	0.03	0.42	0.35	0.02	0.00	0.01	0.26	0.23	0.01	0.00	0.80	0.00	0.36	0.11	0.01
0.02	0.00	0.00	0.18	0.00	0.00	0.00	0.00	0.06	0.13	0.00	0.00	1.00	0.00	0.00	0.06	0.00
0.00	0.00	0.00	0.00	0.00	0.00	0.00	0.00	0.14	0.01	0.00	0.00	0.04	0.00	0.02	0.00	0.00
0.09	0.00	0.00	0.00	0.00	0.00	0.00	0.00	0.00	0.19	0.00	0.00	0.00	0.00	0.00	0.00	0.00
0.00	0.06	0.00	0.00	0.00	0.01	0.00	0.00	0.01	0.02	0.00	0.00	0.00	0.00	0.00	0.00	0.00
0.05	0.06	0.14	0.00	0.00	0.01	0.00	0.00	0.07	0.07	0.00	0.00	0.01	0.00	0.00	0.00	0.00
0.00	0.00	0.00	0.21	0.07	0.00	0.00	0.00	0.15	0.08	0.00	0.00	0.08	0.00	0.00	0.00	0.00
0.00	0.00	0.00	0.15	0.26	0.00	0.00	0.00	0.16	0.03	0.00	0.01	0.03	0.00	0.00	0.00	0.00
0.00	0.00	0.00	0.00	0.00	0.00	0.00	0.00	0.00	0.00	0.00	0.00	0.00	0.00	0.00	0.00	0.00
0.00	0.00	0.00	0.00	0.00	0.00	0.00	0.00	0.00	0.00	0.00	0.00	0.00	0.00	0.00	0.00	0.00
0.00	0.00	0.00	0.00	0.00	0.00	0.00	0.00	0.00	0.00	0.00	0.00	0.01	0.00	0.00	0.00	0.00
0.00	0.01	0.01	0.01	0.00	0.00	0.00	0.00	0.06	0.02	0.00	0.00	0.01	0.00	0.00	0.00	0.00
0.00	0.00	0.00	0.01	0.00	0.00	0.00	0.00	0.02	0.01	0.00	0.00	0.00	0.00	0.00	0.00	0.00
0.00	0.00	0.00	0.00	0.00	0.00	0.00	0.00	0.00	0.01	0.01	0.00	0.03	0.00	0.00	0.00	0.00
0.00	0.00	0.00	0.00	0.00	0.00	0.00	0.00	0.00	0.00	0.00	0.00	0.00	0.00	0.00	0.00	0.00
0.01	0.00	0.00	0.13	0.00	0.00	0.00	0.00	0.03	0.20	0.00	0.00	0.44	0.00	0.00	0.04	0.00
0.00	0.00	0.00	0.00	0.00	0.00	0.00	0.00	0.00	0.00	0.00	0.00	0.00	0.00	0.00	0.00	0.00
0.00	0.00	0.00	0.00	0.00	0.00	0.00	0.00	0.31	0.02	0.00	0.00	0.05	0.00	0.14	0.00	0.01
0.00	0.00	0.00	0.01	0.00	0.00	0.00	0.00	0.00	0.03	0.00	0.00	0.13	0.00	0.00	0.00	0.00
0.00	0.00	0.00	0.00	0.00	0.00	0.00	0.00	0.00	0.00	0.00	0.00	0.00	0.00	0.00	0.00	0.00
0.00	0.00	0.00	0.00	0.00	0.00	0.00	0.00	0.08	0.00	0.00	0.00	0.00	0.00	0.06	0.00	0.00
0.00	0.00	0.00	0.00	0.00	0.00	0.00	0.00	0.00	0.02	0.00	0.00	0.00	0.00	0.00	0.00	0.00
0.00	0.00	0.00	0.00	0.00	0.00	0.00	0.00	0.00	0.00	0.00	0.00	0.00	0.00	0.00	0.00	0.00

0.32	0.15	0.19	1.11	0.68	0.07	0.00	0.01	1.41	1.13	0.01	0.02	2.84	0.00	0.59	0.22	0.02
0.09	0.04	0.05	0.31	0.19	0.02	0.00	0.00	0.40	0.32	0.00	0.01	0.80	0.00	0.17	0.06	0.01

K24	K25	K26
0.00	0.00	0.00
0.00	0.00	0.00
0.00	0.00	0.00
0.04	0.00	0.00
0.00	0.00	0.00
0.00	0.00	0.00
0.00	0.00	0.00
0.00	0.00	0.00
0.01	0.03	0.00
0.00	0.00	0.00
0.00	0.00	0.00
0.00	0.00	0.00
0.00	0.00	0.00
0.00	0.00	0.00
0.00	0.00	0.00
0.00	0.00	0.00
0.00	0.00	0.00
0.00	0.00	0.00
0.00	0.00	0.00
0.00	0.00	0.00
0.03	0.00	0.00
0.00	0.00	0.00
0.00	0.00	0.00
0.00	0.00	0.00
0.00	0.01	0.00
0.00	0.00	0.00

0.09	0.04	0.00
0.03	0.01	0.00

Outdegree	
0.00	0.00
0.20	0.05
0.43	0.10
4.40	1.00
2.34	0.53
0.41	0.09
0.44	0.10
0.21	0.05
0.74	0.17
0.99	0.23
1.03	0.23
0.01	0.00
0.02	0.00
0.03	0.01
0.36	0.08
0.22	0.05
0.07	0.01
0.01	0.00
1.40	0.32
0.00	0.00
0.88	0.20
0.26	0.06
0.01	0.00
0.27	0.06
0.05	0.01
0.00	0.00

For Peer Review
